# A disease-related essential protein prediction model based on the transfer neural network

**DOI:** 10.3389/fgene.2022.1087294

**Published:** 2023-01-04

**Authors:** Sisi Chen, Chiguo Huang, Lei Wang, Shunxian Zhou

**Affiliations:** ^1^ The First Hospital of Hunan University of Chinese Medicine, Changsha, Hunan, China; ^2^ Big Data Innovation and Entrepreneurship Education Center of Hunan Province, Changsha University, Changsha, China; ^3^ College of Information Science and Engineering, Hunan Women’s University, Changsha, Hunan, China

**Keywords:** essential protein, prediction model, transfer neural network, biological information, internal disease, protein-protein interaction network

## Abstract

Essential proteins play important roles in the development and survival of organisms whose mutations are proven to be the drivers of common internal diseases having higher prevalence rates. Due to high costs of traditional biological experiments, an improved Transfer Neural Network (TNN) was designed to extract raw features from multiple biological information of proteins first, and then, based on the newly-constructed Transfer Neural Network, a novel computational model called TNNM was designed to infer essential proteins in this paper. Different from traditional Markov chain, since Transfer Neural Network adopted the gradient descent algorithm to automatically obtain the transition probability matrix, the prediction accuracy of TNNM was greatly improved. Moreover, additional antecedent memory coefficient and bias term were introduced in Transfer Neural Network, which further enhanced both the robustness and the non-linear expression ability of TNNM as well. Finally, in order to evaluate the identification performance of TNNM, intensive experiments have been executed based on two well-known public databases separately, and experimental results show that TNNM can achieve better performance than representative state-of-the-art prediction models in terms of both predictive accuracies and decline rate of accuracies. Therefore, TNNM may play an important role in key protein prediction in the future.

## 1 Introduction

Essential proteins refer to proteins that removal of them will lead to cell death or infertility (Acencio and Lemke, 2009). Identification of essential proteins can help us understand the basic requirements for maintaining life forms. However, considering that it is very costly and time-consuming to identify essential proteins by adopting traditional biological experimental techniques such as gene knock-out (Maliackal et al., 2005), RNA interference (Jeong et al., 2001) and conditional knockout (Hahn and Kern, 2005), more and more computational models have been proposed to infer essential proteins in recent years based on the topological characteristics of Protein-Protein Interaction (PPI) networks, in which, proteins are the vertices of PPI networks, while the interactions between proteins constitute the edges. Researches demonstrated that the topological position of a protein in the protein network has a strong correlation with whether the protein is critical (Jeong et al., 2001; Maslov and Sneppen, 2002; Pržulj et al., 2004). Based on the topological characteristics of proteins in PPI networks, a series of essential protein recognition methods have been proposed. For instance, based on the centrality of PPI networks (Stephenson and Zelen, 1989; Jeong et al., 2001), different computational models including CC (Closeness Centrality) (Stefan and Stadler, 2003), DC (Degree Centrality) (Hahn and Kern, 2005), BC (Between Centrality) (Wang et al., 2012), SC (Graph Centrality) (Maliackal et al., 2005) and NC (Neighbor Centrality) (Wang et al., 2012) have been proposed in succession. In addition, Li M et al. designed a recognition model named LAC (Li et al., 2011) to detect essential proteins (Li et al., 2015) based on the local average connectivity of protein nodes in the PPI network. Qi Yi et al. (Qi and Luo, 2016) introduced a prediction model based on the local interaction density (LID) of protein nodes in the PPI network to infer essential proteins. Chen B et al. (Chen and Wu, 2013) proposed an essential protein recognition method based on multiple topological features of the PPI network. In all these above methods, only topological characteristics of the PPI network were considered to identify essential proteins, however, since there is a large amount of noise data in PPI networks, then the predictive accuracy of these methods is not very satisfactory.

In order to break through the inherent limitations of existing PPI data, in the past few years, people proposed novel models by combining the topological characteristics of PPI networks with biological information of proteins. For example, M Li et al. and Xiwei Tang et al. put forward prediction models called Pec (Li et al., 2012) and WDC (Tang et al., 2014) by integrating PPI network and gene expression data of proteins respectively. W Peng et al. designed a prediction model (Peng et al., 2012) by integrating protein homology information with PPI networks, and a prediction model (Peng et al., 2015) through combining protein domain information with PPI network, simultaneously. X Zhang et al. (Zhang et al., 2013) introduced a recognition method called CoEWC by merging topological characteristics of the PPI network with the co expression characteristics of proteins. BH Zhao et al. designed a prediction model named POEM (Zhao et al., 2014) by combining gene expression data of proteins with the topological characteristics of PPI networks. J Luo et al. proposed a identification method based on local interaction density of PPI networks and biological characteristics of protein complexes (Jiawei et al., 2015). Seketoulie Keretsu et al. presented a protein complex recognition model (Li et al., 2015) based on clustering weighted edges and gene expression profile of proteins. M Li et al. designed two essential protein recognition methods by combining PPI networks with subcellular location information and complex centrality of proteins respectively (Keretsu and Sarmah, 2016; Li et al., 2017; Chen et al., 2020). J Luo et al. introduced a prediction method ECC (edge clustering coefficient) based on the complex co expression data of proteins and PPI networks (Luo and Wu, 2015). Bihai Zhao et al. proposed a model based on multiple biological networks (Zhao et al., 2020a) and a model based on diffusion distance network (Zhao et al., 2020b) to predict essential proteins respectively. S. Li et al. designed an iterative method called CVIM (Li et al., 2020) based on topological and functional characteristics of proteins to predict key proteins. Lei X et al. proposed a necessary protein prediction method AFSOEP (Lei et al., 2018) to infer protein complexes through AFSO (Artificial Fish Swarm Optimization). BH Zhao et al. designed an iterative method to identify potential essential proteins (Zhao et al., 2019) based on heterogeneous PPI networks. Dai W et al. proposed a method to discover key genes based on protein-protein interaction network embedding (Dai et al., 2020). Fengyu Zhang et al. predicted the key gene (Zhang et al., 2019) by fusing the dynamic PPI network. Chen Z et al. proposed an essential protein prediction model NPRI based on heterogeneous network, and established heterogeneous protein domain network (Chen et al., 2020) according to initial PPI network, protein domain network and gene expression data.

All these above methods show that the identification accuracy of models can be significantly improved by combining biological information of proteins with topological features of PPI networks. However, through analyzing results achieved by these existing methods, it is not difficult to find that the prediction accuracies of these algorithms decline fast with the increasing of predicted essential proteins. Hence, inspired by recognition models based on the Markov chain and the Transfer algorithm, we designed a new neural network called TNN in this manuscript, based on which, a novel model named TNNM was proposed to predict essential proteins. TNN can be divided into three parts, namely, probability transfer matrix, antecedent output and bias term. In addition, in order to evaluate the performance of TNNM, we compared it with existing representative models such as IC (Stephenson and Zelen, 1989), DC (Hahn and Kern, 2005), SC (Maliackal et al., 2005), NC (Wang et al., 2012), PeC (Li et al., 2012), ION (Peng et al., 2012), CoEWC (Zhang et al., 2013), POEM (Zhao et al., 2014), CVIM (Li et al., 2020), NPRI (Chen et al., 2020) and RWHN (Zhao et al., 2014) separately. Experimental results show that TNNM is far superior to these traditional models in terms of both predictive accuracy and decline rate of accuracy.

The rest of this paper is organized as follows: The experimental data and specific steps are organized in Section 2. In Section 3, the influence of parameters and comparison with other methods are shown. Section 4 describes the shortcomings of the model and future improvement goals. Finally, a summary is made in Part 5.

## 2 Method and materials

**FIGURE 1:**
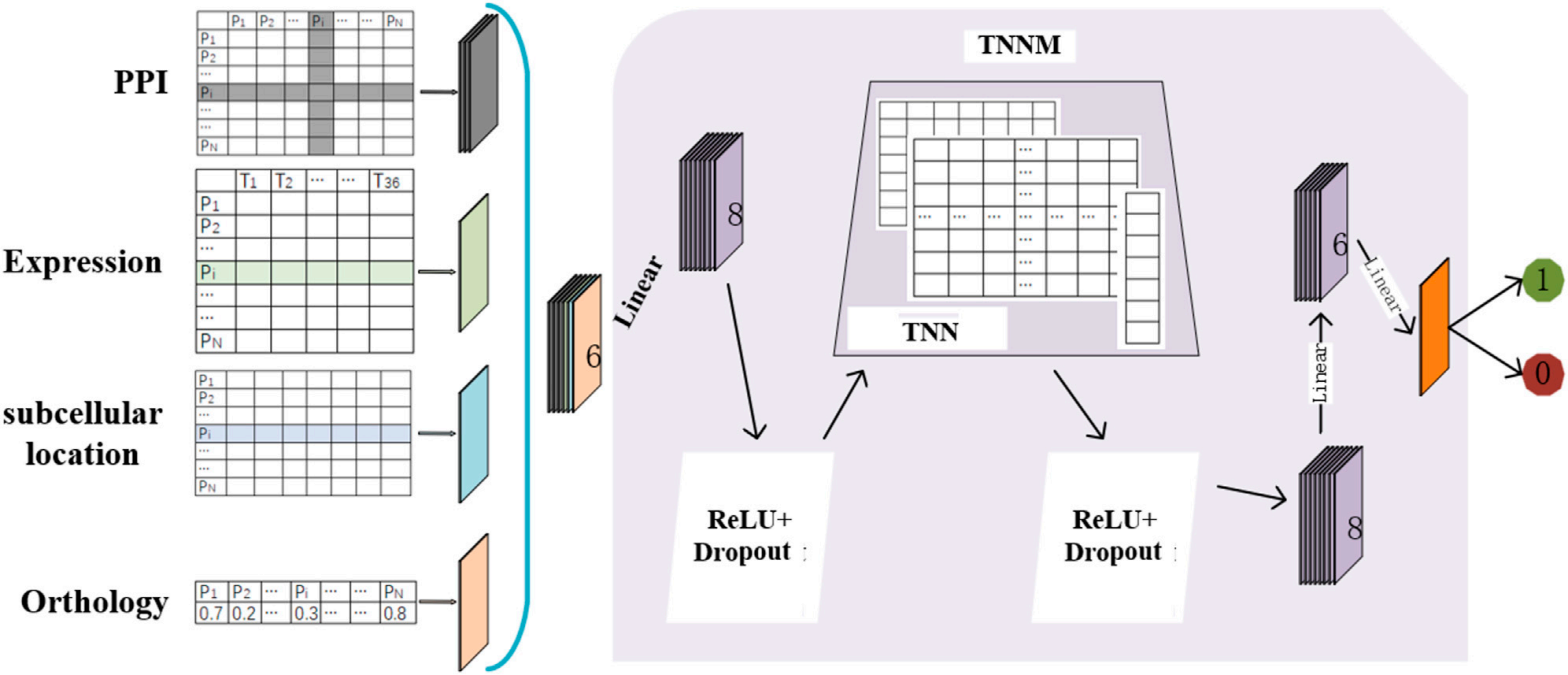
Flowchart of TNNM.

The flow chart of TNNM is shown in Figure 1. Through observing Figure 1, it is easy to see that TNNM consists of the following three major parts. Firstly, based on prior knowledge, topological features and biological features of each protein will be extracted from PPI networks, gene expression data, subcellular localization and ortholog data of proteins separately. And then, the Transfer Neural Network (TNN) will be designed. Finally, through adopting TNN, the prediction model TNNM will be constructed to infer essential proteins based on these newly extracted features.

### 2.1 Experimental data

In order to evaluate the prediction performance of TNNM, during experiments, we first downloaded known PPIs from different benchmark databases such as DIP 2010 (Xenarios et al., 2002) and Gavin (Gavin et al., 2006) respectively. After preprocessing, a dataset containing 5093 proteins and 24,743 known PPIs was finally obtained from the DIP2010 database, and a dataset containing 1855 proteins and 7669 known PPIs was obtained from the Gavin database. In addition, based on databases including MIPS (Mewes et al., 2006), SGD (Cherry et al., 1998), DEG (Zhang and Lin, 2009) and SGDP (StanfordMedicine, 2012), a benchmark dataset containing 1285 essential proteins was constructed, based on which, 1167 and 714 essential proteins were screened from the DIP2010 and Gavin databases respectively. Moreover, based on the dataset provided by Tu BP et al. (Tu et al., 2005), a dataset containing the gene expression data of 6776 proteins was obtained, which consists of the gene expression level data of proteins in the continuous metabolic cycle. Simultaneously, the homologous information of proteins was downloaded from the Inparanoid database (seventh edition), including paired comparison (Gabriel et al., 2010) between 100 whole genomes, and the number of times that proteins have homologous information in the reference organism. Finally, we downloaded the dataset containing subcellular localization information of proteins from the COMPART-MENTS database (Binder et al., 2014) (2014 version), and retained only 11 types of subcellular localization data closely related to essential proteins, such as cytoplasm, cytoskeleton, Golgi apparatus, cytoplasm, vacuoles, mitochondria, endosomes, plasma, nucleus, peroxisomes and extracellular enzymes, etc.

Based on above newly-downloaded datasets, firstly, we constructed an original PPI network. And then, through combining with the existing complex network topological features including degree centrality, closeness centrality, node betweenness centrality and edge betweenness centrality, some new important protein topological features are extracted from the PPI network, including the degree of contact between the protein node and the neighborhood nodes, the importance of the protein node relative to the total distance, and the importance of the protein node relative to the carrying capacity. Simultaneously, we would further extract some biological features for proteins, including the importance of protein node relative to the Pearson correlation coefficient, the importance of protein node relative to the subcellular locations, and the importance of protein node relative to the homologous information, from multiple biological information existed in above newly-downloaded datasets.

### 2.2 Topological feature extraction

Let the undirected graph $G=\left(V,E\right)$ represent the original PPI network formed by a dataset downloaded from any given base database, $V=\left\{p_{1},p_{2},\cdots ,p_{N}\right\}$ denote the set of different proteins in the downloaded dataset, then, for any two given proteins $p_{i}$ and $p_{j}$ in *V*, we define that there is an edge $e\left(p_{i},p_{j}\right)$ between $p_{i}$ and $p_{j}$ , if and only if there is a known interaction between them. And for convenience, we define that $E=\left\{e\left(p_{i},p_{j}\right)|p_{i},p_{j}\in V\right\}$ represents the set of edges in *G*. Hence, we can obtain the adjacency matrix $A={\left[a_{ij}\right]}_{N\times N}$ corresponding to G as follows: if there is $e\left(p_{i},p_{j}\right)\in E$ , then there is $a_{ij}=1$ , otherwise there is $a_{ij}=0$ .

For any given protein p∈V in *G*, let $N\left(P\right)$ be the set of neighboring nodes of *p*, then we have:

$$N\left(p\right)=\left\{q|q\in V,e\left(p,q\right)\in E\right\}$$ (1)

Based on above formula (1), we define that the degree of contact between p and its neighboring nodes as follows:

$${TF}_{1}\left(p\right)=\frac{\sum_{q\in Ng\left(p\right)}Tris\left(p,q\right)}{\left|N\left(p\right)\right|}$$ (2)

Here, $\left|N\left(p\right)\right|$ represents the number of elements in $N\left(p\right)$ , and $Tris\left(p,q\right)$ denotes the number of common neighbors of *p* and *q*, which can be calculated as follows:

$$Tris\left(p,q\right)=\left\{\begin{array}{c} \frac{\left|N\left(p\right)\cap Ng\left(q\right)\right|}{\min\left\{\left|N\left(p\right)\right|,\;\left|N\left(q\right)\right|\right\}},\;p\in N\left(q\right),q\in N\left(p\right)\\ 0,\;otherwise \end{array}\right.$$ (3)

Here, |*N*(*p*)∩*N*(*q*)| represents the number of elements in *N*(*p*)∩*N*(*q*).

It is reasonable to consume that the smaller the total distance between a protein and all other proteins, the more important the protein will be. Hence, let *l* (*p*, *q*) denote the length of the shortest path from protein *p* to the protein *q* in *G*, if there is no path between *p* and *q* in *G*, then we define the length of the shortest path between *p* and *q* is a constant number *N* (>1). Therefore, we can calculate the importance of *p* related to the total distance as follows:

$${TF}_{2}\left(p\right)=\frac{N-1}{\sum_{q\in V}l\left(p,q\right)}$$ (4)

Moreover, it is also reasonable to assume that the more important a protein *p* is, the more proteins that have the shortest path through *p*. This indicator reflects the carrying capacity of *p* between other nodes in *G*. it is obvious that the larger the value, the greater the impact of *p* in the network, which also means that *p* will be more important. Hence, we can calculate the importance of *p* related to the carrying capacity as follows:

$${TF}_{3}\left(p\right)=\sum_{p\neq q\neq q^{\prime }\in V}\frac{k_{qq^{\prime }}\left(p\right)}{k_{qq^{\prime }}}$$ (5)

Here, *k*_*qq’*_ represents the number of shortest paths between *q* and *q′* in *G*, and *k*_*qq′*_ *p*) denotes the number of shortest paths between *q* and *q′* in *G*, which pass through *p*.

### 2.3 Biological feature extraction

Let *ge* (*p*, *t*) represent the gene expression value of the protein *p* at the time point *t*, $ge\left(p\right)$ denote the average expression level of *p* at all *n* time points, and $\sigma \left(p\right)$ be the standard variance of the gene expression level of *p* at all *n* time points, then we can calculate the Pearson correlation coefficient between *p* and *q* as follows:

$$PCC\left(p,q\right)=\frac{1}{n-1}\overset{n}{\underset{t=1}{\sum }}\left[\frac{ge\left(p,t\right)-ge\left(p\right)\;}{\sigma \left(p\right)}\right]\left[\frac{ge\left(q,t\right)-ge\left(q\right)\;}{\sigma \left(q\right)}\right]$$ (6)

Based on above formula (6), we can calculate the importance of *p* related to the Pearson correlation coefficient as follows:

$${BF}_{1}\left(p\right)=\sum_{q\in Ng\left(p\right)}PCC\left(p,q\right)$$ (7)

It is reasonable to consume that essential proteins tend to be connected rather than independent. Therefore, we can believe that proteins closely related to essential proteins are more likely to be essential proteins. Thus, we can obtain another importance indicator of *p* as follows:

$${BF}_{2}\left(p\right)=\frac{\sum_{q\in Ng\left(p\right)}Bsub\left(p,q\right)}{\left|Ng\left(p\right)\right|}$$ (8)

Where $Bsub\left(p,q\right)$ can be obtained as follows:

$$Bsub\left(p,q\right)=\frac{\left|Sub\left(p\right)\cap Sub\left(q\right)\right|}{\left|Sub\left(p\right)\cup Sub\left(q\right)\right|+1}$$ (9)

Here, *Sub*(*p*) represents the set of subcellular locations of the protein *p*, *|Sub*(*p*)*∩Sub*(*q*)*|* denotes the number of elements in *Sub*(*p*)*∩Sub*(*q*), and *|Sub*(*p*)∪*Sub*(*q*)*|* is the number of elements in *Sub*(*p*)∪*Sub*(*q*).

Moreover, based on the reasonable assumption that the evolution of essential proteins is more conservative than that of non-essential proteins, and considering that the homologous information of proteins can objectively reflect the degree of evolutionary conservatism of proteins, let $Os\left(p\right)$ denote the value of homologous score of *p*, then it is obvious that the higher the value of $Os\left(p\right)$ , the more conservative the evolution of *p* will be, i.e., the more important the protein *p* will be. Thus calculate the importance of *p* related to the homologous information as follows:

BF3p=OspOsqq∈Vmax (10)

### 2.4 Construction of the TNN

A Markov chain is a stochastic process, whose characteristic can be summarized as “the future depends on the past only through the present”, that is, the probability distribution of the next state can only be determined by the current state, and the events before it in the time series are independent of it. In a Markov chain, let $T^{n}$ denote the state space at time step *n*, and Q represent the transition probability matrix, then there is:

$$T^{n+1}=QT^{n}$$ (11)

Due to strong predictive ability, Markov chains have been widely used in natural language processing, multivariate factor analysis, time series prediction and other fields. Inspired by the idea of Markov chains, in this manuscript, we designed a novel Transfer Neural Network called TNN, whose destination is being able to learn inherent feature representations from input data just like a Markov chain. In TNN, we introduced three main parameters such as the transition probability matrix *W*, the antecedent memory coefficient α with value between 0 and 1, and a bias term *b*. Let $X^{i}$ denote the input data of the *i*th layer in TNN, then similar to the principle of Markov chains, we define its output $X^{i+1}$ as follows:

$$X^{i+1}=\alpha *W*X^{i}+\left(1-\alpha \right)*X^{i}+b$$ (12)

In the training process, TNN will adopt the gradient descent algorithm to optimize all parameters including *W*, α and *b* in above Eq. 12, and can automatically find a set of optimal values for all these parameters. Thereafter, in the comparative experiments, through a series of complex calculations performed by itself and previous layers in TNNM based on these optimized parameters, TNN is able to assign larger weight values to more important features of proteins, and extract the most important features of proteins from the input data of TNNM, thus achieving satisfactory feature enhancement.

### 2.5 Construction of TNNM

Firstly, as illustrated in Figure 1, let $X^{0}={\left[X^{0}\left(p_{1}\right),\;X^{0}\left(p_{2}\right),\ldots ,X^{0}\left(p_{N}\right)\right]}^{T}$ denote the input data of the input layer in TNNM, then for any given protein $p_{i}\in V$ , there is:

$$X^{0}\left(p_{i}\right)=<\begin{array}{ccc} \begin{array}{cc} {TF}_{1}\left(p_{i}\right) & {TF}_{2}\left(p_{i}\right) \end{array} & \begin{array}{cc} {TF}_{3}\left(p_{i}\right) & {BF}_{1}\left(p_{i}\right) \end{array} & \begin{array}{cc} {BF}_{2}\left(p_{i}\right) & {BF}_{3}\left(p_{i}\right) \end{array} \end{array}>$$ (13)

Secondly, considering that $X^{0}$ is a N×6 dimensional matrix, during experiments, we set the input and output dimensions of the first Linear layer in TNNM as six and 8 separately.

Thirdly, in the ReLU layer of TNNM, we adopt the following activation function:

$$X_{jk}^{i}=\left\{\begin{array}{c} X_{jk}^{i-1}:if\;X_{jk}^{i-1}>0\\ 0:\;otherwise \end{array}\right.$$ (14)

Here, $X_{jk}^{i}$ denotes the element in the *j*th row and *k*th column of $X^{i}$ . And $X^{i}$ and $X^{i-1}$ represent the input and output data of the ReLU layer respectively.

Moreover, in order to solve the problem of over fitting and reduce the training time of TNNM, we introduced two Dropout layers before and after the TNN layer. When each round of samples is inputted into TNNM for training, a probability *p* will be set in the Dropout layer so that each neuron will participate in training with the probability 1-*p*, that is, each neuron has a probability *p* of death. During experiments, we will set 0.7 to *p* in this manuscript.

Next, in the TNN layer, it is obvious that its input data is a N× 8 dimensional matrix, and for each protein, an 8-dimensional feature vector will be extracted by TNN as its output. Hence, in the second Linear layer of TNNM, we will set its input and output dimensions as eight and six respectively.

Finally, in order to estimate the criticality of proteins, we will set the input and output dimensions of the last Linear layer in TNNM as six and 1 separately, that is, TNNM will output 0 or 1 as its final predicted score.

Especially, in each Linear layer of TNNM, we will adopt the following Linear function:

$$X^{i+1}=X^{i}W^{\prime }+b$$ (15)

Here, $W^{\prime }$ is a matrix with *m* rows and *n* columns, where *m* and *n* denote the dimensions of input and output data of the Linear layer respectively. For instance, it is obvious that in these three Linear layers of TNNM, the dimensions of matrix $W^{\prime }$ will be 6 × 8, 8 × 6 and 6 × 1 respectively. And additionally, $X^{i+1}$ and $X^{i}$ represent the input and output data of the Linear layer respectively.

### 2.6 Identification algorithm based on TNNM

Based on above description, we can present the identification algorithm based on TNNM as follows:

Step1: Based on the datasets of known PPIs downloaded from well-known public databases, constructing the original PPI network G and the corresponding adjacency matrix A .

Step2: According to Eqs. 2 and 4, 5, extracting three kinds of important topological features for proteins from G respectively.

Step3: According to Eqs. 7, 8 and 10, extracting three kinds of important biological features for proteins separately.

Step4: According to methods proposed in section 2.4 and section 2.5, constructing the TNN based identification model TNNM first, and then, obtaining the predicted criticality scores for proteins through taking the matrix $X^{0}$ computed by Eq. 13 as the input data of TNNM.

## 3 Experimental results and analysis

During experiments, we will first divide the dataset of downloaded known PPI data into *K* subsets of proteins with the same size and proportion according to the proportion of essential proteins and non-essential proteins. And then, the *K*-fold cross validation will be adopted to evaluate the prediction performance of TNNM in this section.

### 3.1 Value selection of the parameter *K*

According to known results (Jung, 2017), the parameter *K* shall satisfy *K* ≈ log(*N*) and *N*/*K* > 3 *d*ays, where *d* is the number of extracted features. Hence, we can obtain the possible values of *K* as the following Table 1.

**TABLE 1:** Values of *K* in different database.

PPI database	N	3 days	K	N/K	N/K > 3 days
DIP2010	5093	18	9	565.89	True
Gavin	1855	18	8	231.88	True

From observing above table 1, it is easy to see that the value of *K* shall be nine on the DIP2010 database and eight on the Gavin database.

### 3.2 Comparison with representative methods

**FIGURE 2:**
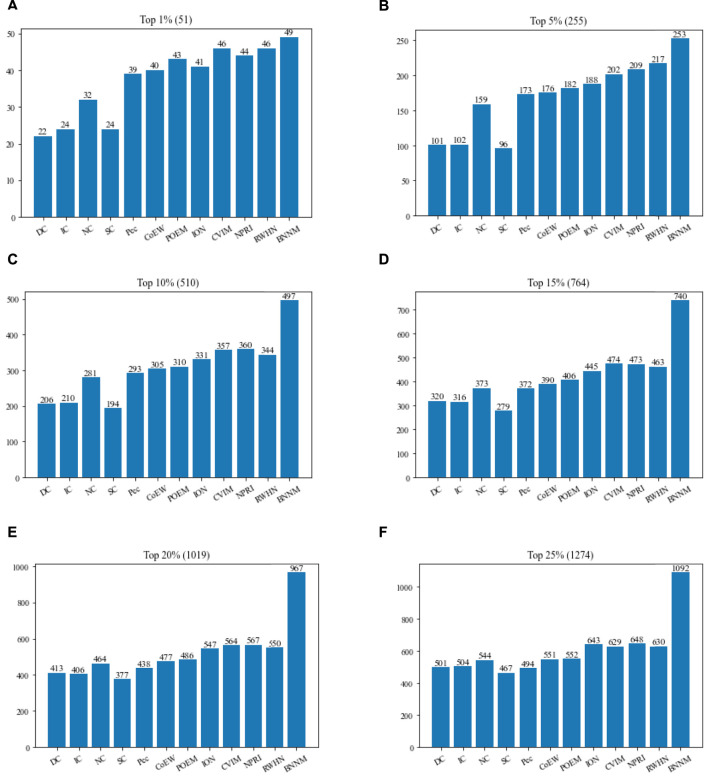
Comparison between TNNM and 11 competitive methods based on the DIP 2010. **(A)** The number of key proteins in the top 1% candidate proteins. **(B)** Number of key proteins in the top 5% candidate proteins. **(C)** Number of key proteins in the top 10% candidate proteins. **(D)** Number of key proteins in the top 15% candidate proteins. **(E)** Number of key proteins in the top 20% candidate proteins. **(F)** Number of key proteins in the top 25% candidate proteins. In above figures, the number in parentheses represents the number of proteins in each interval.

In this section, TNNM will be compared with 11 advanced competitive methods based on the DIP2010 database. Figure 2 shows the comparison results of the numbers of real essential proteins identified by TNNM and 11 recognition methods based on the DIP2010 database. During experiments, proteins will be sorted first in descending order according to predicted scores calculated by each competing methods, such as DC, IC, NC, SC, Pec, POME, CoEWC, ION, CVIM, NPRI, RWHN and TNNM. And then, we will select the top 1%, 5%, 10%, 15%, 20% and 25% proteins as candidate essential proteins. Finally, by comparing with the downloaded dataset of known essential proteins, the number of real essential proteins in the candidate essential proteins identified by each method will be calculated, and used to compare and evaluate the recognition ability of essential proteins of different methods.

From observing Figure 2, it is easy to know that TNNM outperforms all these competitive state-of-the-art prediction methods significantly based on the experimental results on DIP2010 database. And especially, among the top 1%, top 5%, and top 10% candidate key proteins, TNNM can achieve recognition accuracies of 96.07%, 99.21%, and 97.45% separately, which are all higher than 97%. Besides, among the top 15% and 20% candidate key proteins, the recognition accuracy rates of TNNM are all higher than 94%. Even for the top 25% candidate proteins, TNNM can maintain the accuracy rate above 85%.

### 3.3 Evaluation based on the folding knife curve

**FIGURE 3:**
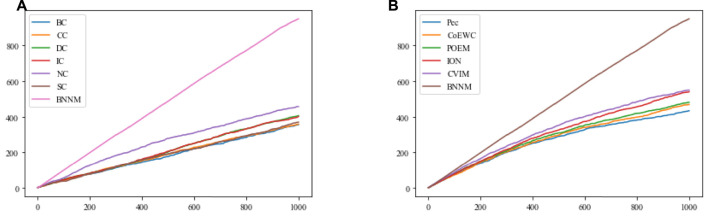
Comparison results between TNNM and 11 competitive methods based on the jackknife method and the DIP2010 database. **(A)** Shows comparison results between BC, CC, DC, IC, NC, SC and TNNM, and **(B)** shows comparison results between Pec, CoEWC, POEM, ION, CVIM and TNNM. The *X*-axis represents the number of potential essential proteins predicted by each method, while the *Y*-axis represents the cumulative count of real essential proteins.

In this section, the Jackknife method (Holman et al., 2009) will be used, based on the top 1000 candidate essential proteins predicted on the DIP2010 database by TNNM and 11 competitive methods, to compare their performance in identifying essential proteins. Comparison results are shown in Figure 3.

From Figure 3A and Figure 3B, it can be seen that with the increasing of the number of predicted proteins, the gap in term of essential protein recognition performance between TNNM and these competitive methods will grow wider and wider, which means that the prediction performance of TNNM is much better than that of these 11 competitive methods.

### 3.4 Comparison between TNN and representative neural networks

**FIGURE 4:**
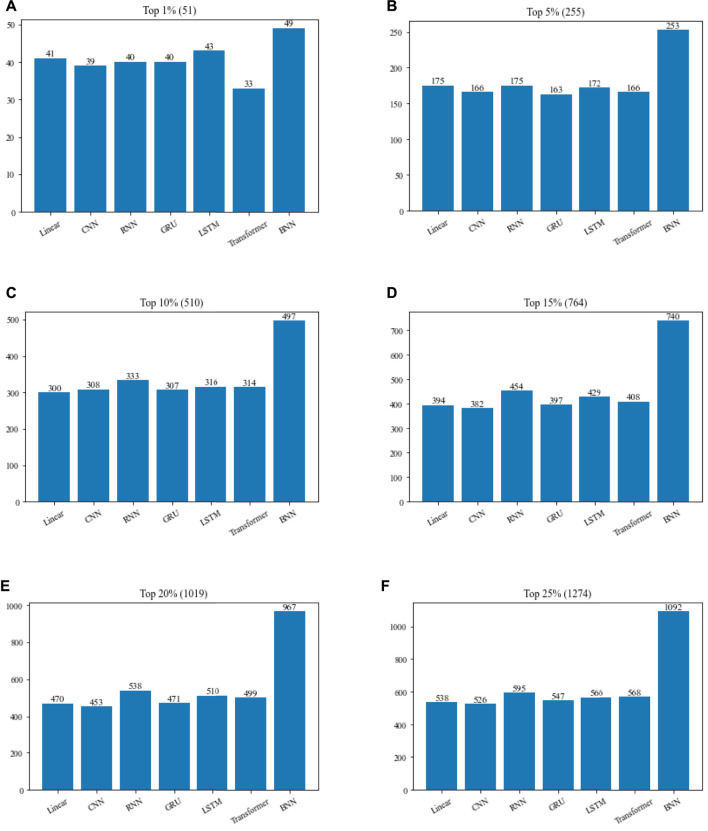
Performance comparison of TNNM by adopting TNN and six representative neural networks based on the DIP2010 database. **(A)** The number of essential proteins in the top 1% candidate proteins. **(B)** The number of essential proteins in the top 5% candidate proteins. **(C)** The number of essential proteins in the top 10% candidate proteins. **(D)** The number of essential proteins in the top 15% candidate proteins. **(E)** The number of essential proteins in the top 20% candidate proteins. **(F)** The number of essential proteins in the top 25% candidate proteins. In above figures, the number in parentheses represents the number of proteins in each interval.

In order to verify the contribution of TNN to TNNM, we will compare TNN with six commonly used neural networks in this section based on the DIP2010 database, and comparison results is illustrated in Figure 4. During experiments, in TNNM, the TNN layer will be replaced by competitive neural networks such as Linear, CNN, RNN, GRU, LSTM and Transformer in turn. And then, the top 1%, 5%, 10%, 15%, 20% and 25% predicted proteins will be compared with downloaded dataset of known essential proteins. Finally, the number of real essential proteins in the candidate essential proteins identified by each method will be calculated, and used to compare and evaluate the recognition ability of essential proteins of different methods.

From Figure 4, it is easy to see that if the TNN in TNNM is replaced by Linear, CNN, RNN, GRU, LSTM or Transformer, the prediction performance of TNNM will turn to be poorer, which reflects that TNN plays a positive role in the prediction performance of TNNM.

### 3.5 Recognition performance based on the gavin database

**FIGURE 5:**
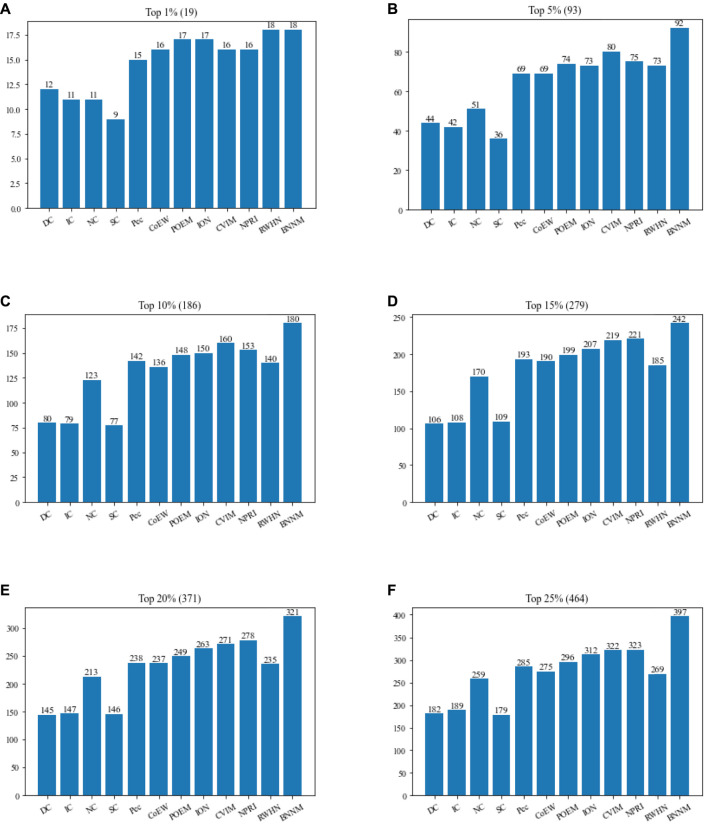
Performance comparison between TNNM and 11 competitive algorithms based on the Gavin database. **(A)** The number of essential proteins in the top 1% candidate proteins. **(B)** The number of essential proteins in the top 5% candidate proteins. **(C)** The number of essential proteins in the top 10% candidate proteins. **(D)** The number of essential proteins in the top 15% candidate proteins. **(E)** The number of essential proteins in the top 20% candidate proteins. **(F)** The number of essential proteins in the top 25% candidate proteins. In above figures, the number in parentheses represents the number of proteins in each interval.

To prove the universal applicability of TNNM, in this section, we further compared TNNM with 11 competitive recognition methods based on the Gavin database, and illustrated comparison results in Figure 5.

From Figure 5, it is obvious that the recognition performance of TNNM is significantly superior to all these 11 competing methods. Especially, among the top 1%, top 5%, and top 10% candidate essential proteins, TNNM can achieve accuracies of 94.73%, 98.92%, and 96.77% respectively, which are all higher than 94%. Besides, among the top 15% and 20% candidate essential proteins, the recognition accuracies of TNNM are higher than 86% as well. Even in the top 25% candidate proteins, TNNM can also maintain its accuracy rate above 85%. Hence, we can say that TNNM has much better universal applicability than all these competitive methods.

## 4 Conclusion

In this manuscript, a novel prediction model named TNNM was designed to identify essential proteins, and through intensive experiments, we demonstrated that TNNM outperformed various advanced algorithms in terms of both prediction accuracies and decline rate of accuracies. The major contributions of TNNM include: 1) we designed a new Transfer Neural Network (TNN), which can extract raw features from multiple biological information of proteins efficiently. 2) we introduced a TNN layer into the prediction model TNNM, which can not only improve the prediction accuracy of TNNM, but also enhance both the robustness and the non-linear expression ability of TNNM. Intensive experiments have demonstrated that TNNM can achieve satisfactory prediction accuracy in different databases, and simultaneously, TNN plays an irreplaceable positive role in TNNM as well.

## Data Availability

The original contributions presented in the study are included in the article/Supplementary Material, further inquiries can be directed to the corresponding authors.
