# Supplementary material for: A disease-related essential protein prediction model based on the transfer neural network
Source: Front Genet. 2023 Jan 4;13:1087294. doi: 10.3389/fgene.2022.1087294 (PMC9845409; doi:10.3389/fgene.2022.1087294)
Supplement: Supplementary file 1 [file DataSheet2.PDF]

snRNA_NME1
snRNA_SNR128
snRNA_SNR14
snRNA_SNR30
snRNA_SNR7-L
snRNA_SNR7-S
snRNA_U3_O1
tQ(CUG)M
YAL001C
YAL003W
YAL012W
YAL025C
YAL032C
YAL033W
YAL034C-B
YAL034W-A
YAL035C-A
YAL035W
YAL038W
YAL041W
YAL043C
YAR007C
YAR008W
YAR019C
YBL003C
YBL004W
YBL014C
YBL018C
YBL020W
YBL023C
YBL026W
YBL030C
YBL034C
YBL035C
YBL040C
YBL041W
YBL050W
YBL073W
YBL074C
YBL076C
YBL077W
YBL084C
YBL092W
YBL097W
YBL102W

YBL105C
YBR002C
YBR004C
YBR009C
YBR011C
YBR024W
YBR029C
YBR038W
YBR049C
YBR055C
YBR058C-A
YBR060C
YBR070C
YBR079C
YBR080C
YBR087W
YBR088C
YBR089C-A
YBR089W
YBR091C
YBR102C
YBR106W
YBR109C
YBR110W
YBR112C
YBR121C
YBR123C
YBR124W
YBR135W
YBR136W
YBR140C
YBR142W
YBR143C
YBR152W
YBR153W
YBR154C
YBR155W
YBR158W
YBR160W
YBR167C
YBR168W
YBR190W
YBR192W
YBR193C
YBR196C

YBR198C
YBR200W
YBR202W
YBR211C
YBR233W-A
YBR234C
YBR236C
YBR237W
YBR243C
YBR247C
YBR252W
YBR253W
YBR254C
YBR256C
YBR257W
YBR265W
YCL003W
YCL004W
YCL017C
YCL028W
YCL031C
YCL041C
YCL043C
YCL052C
YCL053C
YCL054W
YCL059C
YCL067C
YCR012W
YCR013C
YCR035C
YCR039C
YCR042C
YCR052W
YCR054C
YCR057C
YCR072C
YCR088W
YCR093W
YCR096C
YDL003W
YDL004W
YDL007W
YDL008W
YDL013W

YDL014W
YDL015C
YDL016C
YDL017W
YDL028C
YDL029W
YDL030W
YDL031W
YDL043C
YDL045C
YDL055C
YDL058W
YDL060W
YDL064W
YDL067C
YDL084W
YDL087C
YDL089W
YDL092W
YDL097C
YDL098C
YDL102W
YDL103C
YDL105W
YDL108W
YDL111C
YDL120W
YDL126C
YDL132W
YDL139C
YDL140C
YDL141W
YDL143W
YDL145C
YDL147W
YDL148C
YDL150W
YDL152W
YDL153C
YDL155W
YDL163W
YDL164C
YDL165W
YDL166C
YDL193W

YDL195W
YDL196W
YDL205C
YDL207W
YDL208W
YDL209C
YDL212W
YDL217C
YDL220C
YDL221W
YDL232W
YDL235C
YDR002W
YDR013W
YDR016C
YDR021W
YDR023W
YDR037W
YDR041W
YDR044W
YDR045C
YDR047W
YDR050C
YDR052C
YDR053W
YDR054C
YDR060W
YDR062W
YDR064W
YDR081C
YDR082W
YDR086C
YDR087C
YDR088C
YDR091C
YDR113C
YDR118W
YDR123C
YDR130C
YDR141C
YDR145W
YDR151C
YDR160W
YDR164C
YDR166C

YDR167W
YDR168W
YDR170C
YDR172W
YDR177W
YDR180W
YDR182W
YDR187C
YDR188W
YDR189W
YDR190C
YDR196C
YDR201W
YDR208W
YDR211W
YDR212W
YDR224C
YDR226W
YDR228C
YDR231C
YDR232W
YDR233C
YDR235W
YDR236C
YDR238C
YDR240C
YDR241W
YDR243C
YDR246W
YDR267C
YDR280W
YDR288W
YDR292C
YDR299W
YDR301W
YDR302W
YDR303C
YDR308C
YDR311W
YDR320C-A
YDR323C
YDR324C
YDR325W
YDR327W
YDR328C

YDR331W
YDR339C
YDR341C
YDR353W
YDR355C
YDR356W
YDR361C
YDR362C
YDR364C
YDR365C
YDR367W
YDR373W
YDR376W
YDR378C
YDR381W
YDR389W
YDR390C
YDR394W
YDR395W
YDR396W
YDR397C
YDR398W
YDR404C
YDR407C
YDR412W
YDR413C
YDR416W
YDR420W
YDR427W
YDR429C
YDR432W
YDR434W
YDR437W
YDR449C
YDR454C
YDR460W
YDR464W
YDR468C
YDR470C
YDR472W
YDR473C
YDR478W
YDR487C
YDR489W
YDR498C

YDR499W
YDR505C
YDR510W
YDR526C
YDR527W
YDR531W
YDR532C
YEL002C
YEL019C
YEL020W-A
YEL026W
YEL032W
YEL034W
YEL035C
YEL044W
YEL055C
YEL058W
YER003C
YER005W
YER006W
YER008C
YER009W
YER012W
YER013W
YER016W
YER018C
YER019C-A
YER021W
YER022W
YER023W
YER025W
YER026C
YER029C
YER036C
YER038C
YER043C
YER048W-A
YER060W
YER070W
YER074W-A
YER082C
YER093C
YER094C
YER104W
YER106W

YER112W
YER125W
YER126C
YER127W
YER133W
YER136W
YER146W
YER147C
YER148W
YER157W
YER159C
YER165W
YER168C
YER171W
YER172C
YFL002C
YFL005W
YFL008W
YFL009W
YFL017C
YFL017W-A
YFL018W-A
YFL022C
YFL023W
YFL024C
YFL029C
YFL034C-B
YFL035C
YFL035C-A
YFL037W
YFL038C
YFL039C
YFL045C
YFR002W
YFR003C
YFR004W
YFR005C
YFR027W
YFR028C
YFR029W
YFR031C
YFR037C
YFR042W
YFR050C
YFR051C

YFR052W
YGL001C
YGL003C
YGL008C
YGL011C
YGL014W
YGL018C
YGL022W
YGL029W
YGL030W
YGL040C
YGL044C
YGL047W
YGL048C
YGL055W
YGL061C
YGL065C
YGL068W
YGL069C
YGL073W
YGL074C
YGL075C
YGL084C
YGL091C
YGL092W
YGL093W
YGL097W
YGL098W
YGL099W
YGL102C
YGL103W
YGL106W
YGL111W
YGL112C
YGL113W
YGL116W
YGL120C
YGL122C
YGL123W
YGL128C
YGL130W
YGL137W
YGL142C
YGL145W
YGL150C

YGL155W
YGL166W
YGL169W
YGL171W
YGL172W
YGL180W
YGL189C
YGL201C
YGL207W
YGL213C
YGL225W
YGL233W
YGL236C
YGL238W
YGL239C
YGL245W
YGL247W
YGR002C
YGR005C
YGR009C
YGR013W
YGR024C
YGR029W
YGR030C
YGR046W
YGR047C
YGR048W
YGR060W
YGR065C
YGR073C
YGR074W
YGR075C
YGR082W
YGR083C
YGR090W
YGR091W
YGR094W
YGR095C
YGR098C
YGR099W
YGR103W
YGR113W
YGR114C
YGR115C
YGR116W

YGR119C
YGR120C
YGR128C
YGR140W
YGR145W
YGR147C
YGR155W
YGR156W
YGR158C
YGR159C
YGR172C
YGR175C
YGR179C
YGR185C
YGR186W
YGR190C
YGR191W
YGR195W
YGR198W
YGR211W
YGR216C
YGR218W
YGR244C
YGR245C
YGR246C
YGR251W
YGR253C
YGR264C
YGR265W
YGR267C
YGR274C
YGR277C
YGR278W
YGR280C
YGR294W
YHL015W
YHR005C
YHR005C-A
YHR007C
YHR009C
YHR014W
YHR019C
YHR020W
YHR023W
YHR024C

YHR027C
YHR036W
YHR040W
YHR042W
YHR052W
YHR058C
YHR062C
YHR065C
YHR068W
YHR069C
YHR070W
YHR072W
YHR072W-A
YHR074W
YHR082C
YHR083W
YHR084W
YHR085W
YHR086W
YHR088W
YHR089C
YHR098C
YHR099W
YHR101C
YHR102W
YHR107C
YHR118C
YHR122W
YHR128W
YHR143W-A
YHR148W
YHR164C
YHR165C
YHR166C
YHR169W
YHR170W
YHR172W
YHR186C
YHR188C
YHR190W
YHR196W
YHR197W
YHR199C-A
YIL003W
YIL004C

YIL019W
YIL021W
YIL022W
YIL026C
YIL031W
YIL046W
YIL048W
YIL051C
YIL061C
YIL062C
YIL063C
YIL068C
YIL075C
YIL078W
YIL083C
YIL091C
YIL104C
YIL106W
YIL109C
YIL115C
YIL118W
YIL126W
YIL129C
YIL137C
YIL142W
YIL143C
YIL144W
YIL147C
YIL150C
YIL171W
YIR004W
YIR006C
YIR008C
YIR010W
YIR011C
YIR012W
YIR015W
YIR022W
YJL001W
YJL002C
YJL005W
YJL008C
YJL009W
YJL010C
YJL011C

YJL012C
YJL014W
YJL015C
YJL018W
YJL019W
YJL025W
YJL026W
YJL031C
YJL032W
YJL033W
YJL034W
YJL035C
YJL039C
YJL041W
YJL050W
YJL054W
YJL061W
YJL069C
YJL072C
YJL074C
YJL076W
YJL081C
YJL085W
YJL086C
YJL087C
YJL090C
YJL091C
YJL097W
YJL104W
YJL109C
YJL111W
YJL123C
YJL125C
YJL143W
YJL156C
YJL157C
YJL164C
YJL167W
YJL173C
YJL174W
YJL184W
YJL194W
YJL195C
YJL198W
YJL202C

YJL203W
YJR002W
YJR003C
YJR006W
YJR007W
YJR012C
YJR013W
YJR016C
YJR017C
YJR022W
YJR023C
YJR041C
YJR042W
YJR045C
YJR046W
YJR049C
YJR057W
YJR064W
YJR065C
YJR067C
YJR068W
YJR072C
YJR076C
YJR089W
YJR093C
YJR095W
YJR112W
YJR123W
YJR135C
YJR136C
YJR141W
YKL004W
YKL006C-A
YKL012W
YKL013C
YKL014C
YKL018W
YKL019W
YKL021C
YKL022C
YKL024C
YKL028W
YKL033W
YKL035W
YKL036C

YKL037W
YKL042W
YKL045W
YKL049C
YKL052C
YKL058W
YKL059C
YKL060C
YKL078W
YKL082C
YKL083W
YKL088W
YKL089W
YKL095W
YKL099C
YKL101W
YKL104C
YKL108W
YKL111C
YKL112W
YKL122C
YKL125W
YKL138C-A
YKL139W
YKL141W
YKL144C
YKL145W
YKL152C
YKL153W
YKL154W
YKL165C
YKL166C
YKL172W
YKL173W
YKL180W
YKL182W
YKL186C
YKL189W
YKL192C
YKL193C
YKL195W
YKL196C
YKL203C
YKL205W
YKL210W

YKR002W
YKR004C
YKR008W
YKR022C
YKR025W
YKR037C
YKR038C
YKR062W
YKR063C
YKR068C
YKR071C
YKR075C
YKR079C
YKR081C
YKR083C
YKR086W
YLL003W
YLL004W
YLL008W
YLL011W
YLL018C
YLL031C
YLL034C
YLL035W
YLL036C
YLL037W
YLL050C
YLR002C
YLR005W
YLR007W
YLR008C
YLR009W
YLR010C
YLR013W
YLR022C
YLR026C
YLR027C
YLR029C
YLR033W
YLR045C
YLR051C
YLR059C
YLR060W
YLR066W
YLR071C

YLR075W
YLR076C
YLR078C
YLR086W
YLR088W
YLR097C
YLR099W-A
YLR100W
YLR101C
YLR103C
YLR105C
YLR106C
YLR115W
YLR116W
YLR117C
YLR127C
YLR129W
YLR132C
YLR140W
YLR141W
YLR145W
YLR147C
YLR153C
YLR163C
YLR166C
YLR167W
YLR175W
YLR182W
YLR186W
YLR195C
YLR196W
YLR197W
YLR198C
YLR208W
YLR212C
YLR215C
YLR220W
YLR222C
YLR223C
YLR229C
YLR230W
YLR240W
YLR243W
YLR249W
YLR259C

YLR272C
YLR274W
YLR275W
YLR276C
YLR277C
YLR291C
YLR293C
YLR298C
YLR305C
YLR310C
YLR311C
YLR314C
YLR316C
YLR317W
YLR321C
YLR323C
YLR336C
YLR339C
YLR340W
YLR347C
YLR355C
YLR359W
YLR371W
YLR375W
YLR378C
YLR379W
YLR383W
YLR397C
YLR403W
YLR409C
YLR424W
YLR430W
YLR438C-A
YLR440C
YLR454W
YLR457C
YLR458W
YLR459W
YML010W
YML015C
YML023C
YML025C
YML031W
YML043C
YML046W

YML049C
YML064C
YML065W
YML068W
YML069W
YML077W
YML085C
YML091C
YML092C
YML093W
YML098W
YML105C
YML114C
YML125C
YML126C
YML127W
YML128C
YML130C
YMR001C
YMR005W
YMR013C
YMR028W
YMR032W
YMR033W
YMR043W
YMR047C
YMR049C
YMR059W
YMR061W
YMR070W
YMR071C
YMR076C
YMR079W
YMR093W
YMR094W
YMR108W
YMR112C
YMR113W
YMR117C
YMR128W
YMR131C
YMR134W
YMR146C
YMR149W
YMR164C

YMR168C
YMR179W
YMR185W
YMR197C
YMR198W
YMR200W
YMR203W
YMR208W
YMR211W
YMR212C
YMR213W
YMR218C
YMR220W
YMR227C
YMR229C
YMR235C
YMR236W
YMR239C
YMR240C
YMR260C
YMR268C
YMR270C
YMR276W
YMR277W
YMR281W
YMR288W
YMR290C
YMR290W-A
YMR296C
YMR298W
YMR301C
YMR308C
YMR309C
YMR314W
YNL002C
YNL006W
YNL007C
YNL020C
YNL024C-A
YNL026W
YNL028W
YNL036W
YNL038W
YNL039W
YNL044W

YNL048W
YNL059C
YNL061W
YNL062C
YNL075W
YNL088W
YNL102W
YNL103W
YNL110C
YNL112W
YNL113W
YNL114C
YNL118C
YNL124W
YNL126W
YNL131W
YNL132W
YNL137C
YNL138W
YNL138W-A
YNL147W
YNL149C
YNL150W
YNL151C
YNL152W
YNL158W
YNL161W
YNL162W
YNL163C
YNL172W
YNL178W
YNL181W
YNL182C
YNL188W
YNL189W
YNL197C
YNL207W
YNL216W
YNL221C
YNL222W
YNL225C
YNL232W
YNL240C
YNL241C
YNL243W

YNL244C
YNL245C
YNL246W
YNL247W
YNL251C
YNL256W
YNL258C
YNL260C
YNL261W
YNL262W
YNL263C
YNL267W
YNL272C
YNL282W
YNL287W
YNL290W
YNL306W
YNL308C
YNL310C
YNL312W
YNL313C
YNL317W
YNR003C
YNR011C
YNR016C
YNR017W
YNR026C
YNR035C
YNR038W
YNR043W
YNR046W
YNR052C
YNR053C
YNR054C
YOL005C
YOL010W
YOL021C
YOL022C
YOL026C
YOL034W
YOL038W
YOL040C
YOL066C
YOL069W
YOL077C

YOL078W
YOL094C
YOL097C
YOL100W
YOL102C
YOL120C
YOL123W
YOL127W
YOL130W
YOL133W
YOL134C
YOL135C
YOL139C
YOL142W
YOL144W
YOL146W
YOL149W
YOR004W
YOR020C
YOR036W
YOR046C
YOR048C
YOR056C
YOR057W
YOR060C
YOR063W
YOR074C
YOR075W
YOR076C
YOR077W
YOR095C
YOR098C
YOR102W
YOR103C
YOR106W
YOR110W
YOR116C
YOR117W
YOR119C
YOR122C
YOR143C
YOR145C
YOR146W
YOR148C
YOR149C

YOR151C
YOR157C
YOR159C
YOR160W
YOR166C
YOR168W
YOR169C
YOR174W
YOR176W
YOR181W
YOR194C
YOR203W
YOR204W
YOR206W
YOR207C
YOR210W
YOR217W
YOR218C
YOR224C
YOR229W
YOR232W
YOR233W
YOR236W
YOR244W
YOR249C
YOR250C
YOR254C
YOR256C
YOR257W
YOR259C
YOR260W
YOR261C
YOR262W
YOR271C
YOR272W
YOR278W
YOR281C
YOR282W
YOR287C
YOR293W
YOR294W
YOR295W
YOR297C
YOR310C
YOR319W

YOR326W
YOR329C
YOR335C
YOR336W
YOR340C
YOR341W
YOR353C
YOR361C
YOR362C
YOR370C
YOR372C
YOR373W
YPL007C
YPL010W
YPL011C
YPL012W
YPL016W
YPL020C
YPL028W
YPL043W
YPL044C
YPL045W
YPL063W
YPL075W
YPL076W
YPL082C
YPL083C
YPL085W
YPL093W
YPL094C
YPL115C
YPL117C
YPL122C
YPL124W
YPL125W
YPL126W
YPL128C
YPL131W
YPL137C
YPL142C
YPL143W
YPL146C
YPL151C
YPL153C
YPL160W

YPL169C
YPL175W
YPL190C
YPL203W
YPL204W
YPL207W
YPL209C
YPL210C
YPL211W
YPL213W
YPL217C
YPL218W
YPL228W
YPL231W
YPL233W
YPL235W
YPL237W
YPL238C
YPL242C
YPL243W
YPL251W
YPL252C
YPL255W
YPL266W
YPL268W
YPR010C
YPR015C
YPR016C
YPR019W
YPR025C
YPR033C
YPR034W
YPR035W
YPR040W
YPR041W
YPR048W
YPR055W
YPR056W
YPR082C
YPR085C
YPR086W
YPR088C
YPR094W
YPR101W
YPR103W

YPR104C
YPR105C
YPR107C
YPR108W
YPR110C
YPR111W
YPR112C
YPR113W
YPR120C
YPR124W
YPR125W
YPR133C
YPR136C
YPR137W
YPR142C
YPR143W
YPR144C
YPR161C
YPR162C
YPR165W
YPR168W
YPR169W
YPR175W
YPR176C
YPR177C
YPR178W
YPR180W
YPR181C
YPR182W
YPR183W
YPR186C
YPR187W
YPR190C
