# Supplementary material for: A disease-related essential protein prediction model based on the transfer neural network
Source: Front Genet. 2023 Jan 4;13:1087294. doi: 10.3389/fgene.2022.1087294 (PMC9845409; doi:10.3389/fgene.2022.1087294)
Supplement: Supplementary file 2 [file DataSheet4.PDF]

Q0050	6
Q0080	2
Q0085	33
Q0130	55
Q0250	29
Q0275	32
R0020C	0
R0030W	0
YAL001C	6
YAL002W	67
YAL003W	91
YAL004W	0
YAL005C	81
YAL007C	51
YAL009W	12
YAL010C	18
YAL011W	7
YAL012W	86
YAL013W	5
YAL014C	7
YAL015C	38
YAL016W	96
YAL017W	16
YAL018C	4
YAL019W	69
YAL021C	67
YAL022C	80
YAL023C	54
YAL024C	28
YAL025C	90
YAL026C	93
YAL027W	2
YAL028W	2
YAL029C	16
YAL030W	36
YAL031C	4
YAL032C	85
YAL033W	39
YAL034C	9
YAL034W-A	14
YAL035W	92
YAL036C	94
YAL038W	87
YAL040C	11
YAL041W	37

YAL042W	93
YAL043C	36
YAL045C	0
YAL047C	3
YAL048C	86
YAL049C	45
YAL051W	3
YAL053W	13
YAL054C	17
YAL055W	3
YAL056W	0
YAL058W	88
YAL059W	4
YAL060W	6
YAL061W	6
YAL062W	60
YAL063C-A	0
YAL064W	0
YAL064W-B	0
YAL066W	0
YAL067C	11
YAL069W	0
YAR002C-A	26
YAR002W	6
YAR003W	78
YAR007C	94
YAR008W	47
YAR014C	12
YAR018C	65
YAR019C	25
YAR023C	1
YAR027W	0
YAR028W	0
YAR030C	0
YAR031W	0
YAR033W	0
YAR035W	73
YAR042W	59
YAR047C	0
YAR064W	0
YAR066W	0
YAR068W	0
YAR069C	0
YAR071W	20
YAR073W	0

YBL001C	16
YBL002W	40
YBL003C	83
YBL004W	80
YBL005W	0
YBL005W-B	0
YBL006C	2
YBL007C	28
YBL008W	53
YBL009W	12
YBL010C	3
YBL013W	61
YBL014C	5
YBL015W	43
YBL016W	75
YBL017C	34
YBL018C	3
YBL019W	66
YBL020W	82
YBL021C	77
YBL022C	90
YBL023C	96
YBL024W	92
YBL025W	3
YBL026W	77
YBL027W	94
YBL029W	2
YBL030C	52
YBL031W	2
YBL032W	23
YBL033C	26
YBL034C	12
YBL035C	79
YBL036C	94
YBL037W	90
YBL038W	33
YBL039C	71
YBL040C	95
YBL041W	94
YBL042C	2
YBL043W	8
YBL044W	0
YBL045C	4
YBL046W	12
YBL047C	72

YBL049W	84
YBL050W	95
YBL051C	20
YBL052C	21
YBL053W	0
YBL054W	0
YBL056W	39
YBL058W	81
YBL059W	1
YBL061C	26
YBL063W	89
YBL064C	81
YBL065W	0
YBL066C	9
YBL067C	35
YBL068W	59
YBL071C	0
YBL072C	97
YBL074C	44
YBL075C	15
YBL076C	96
YBL078C	93
YBL079W	72
YBL080C	73
YBL081W	1
YBL082C	78
YBL084C	74
YBL085W	16
YBL086C	11
YBL088C	16
YBL089W	20
YBL090W	2
YBL091C	96
YBL092W	92
YBL093C	3
YBL094C	0
YBL095W	12
YBL097W	77
YBL098W	80
YBL099W	91
YBL100C	0
YBL100W-A	0
YBL101C	3
YBL102W	72
YBL103C	23

YBL104C	62
YBL105C	74
YBL106C	45
YBL107C	12
YBL108W	0
YBL112C	0
YBL113C	0
YBR001C	37
YBR003W	91
YBR004C	70
YBR006W	83
YBR007C	14
YBR008C	14
YBR010W	98
YBR011C	95
YBR012C	0
YBR012W-B	0
YBR013C	0
YBR014C	11
YBR015C	14
YBR017C	92
YBR018C	73
YBR019C	30
YBR020W	81
YBR021W	6
YBR023C	27
YBR024W	35
YBR025C	96
YBR027C	0
YBR028C	12
YBR029C	89
YBR033W	0
YBR034C	96
YBR035C	71
YBR036C	2
YBR037C	60
YBR038W	25
YBR039W	92
YBR040W	7
YBR041W	72
YBR042C	62
YBR043C	18
YBR044C	2
YBR045C	1
YBR047W	2

YBR049C	16
YBR050C	0
YBR052C	0
YBR053C	33
YBR054W	8
YBR055C	93
YBR056W	11
YBR057C	5
YBR058C	92
YBR058C-A	2
YBR059C	36
YBR060C	72
YBR061C	90
YBR063C	0
YBR064W	0
YBR065C	22
YBR066C	26
YBR067C	1
YBR068C	5
YBR069C	16
YBR070C	76
YBR072W	16
YBR073W	25
YBR074W	33
YBR076W	1
YBR077C	2
YBR078W	14
YBR079C	90
YBR080C	95
YBR081C	17
YBR082C	59
YBR083W	6
YBR084W	35
YBR085W	48
YBR086C	41
YBR087W	94
YBR088C	92
YBR089C-A	37
YBR089W	0
YBR090C	0
YBR091C	4
YBR093C	21
YBR094W	37
YBR095C	9
YBR096W	19

YBR097W	80
YBR098W	9
YBR099C	0
YBR101C	49
YBR102C	48
YBR103W	70
YBR105C	39
YBR106W	23
YBR107C	0
YBR108W	3
YBR109C	98
YBR109W-A	0
YBR110W	88
YBR111W-A	50
YBR112C	53
YBR113W	0
YBR114W	46
YBR115C	44
YBR116C	0
YBR117C	16
YBR118W	97
YBR119W	15
YBR120C	4
YBR121C	91
YBR122C	12
YBR123C	37
YBR124W	0
YBR125C	8
YBR126C	49
YBR127C	96
YBR128C	2
YBR129C	5
YBR130C	2
YBR131W	3
YBR132C	16
YBR133C	83
YBR134W	0
YBR135W	87
YBR136W	59
YBR137W	11
YBR138C	3
YBR139W	60
YBR140C	20
YBR141C	24
YBR142W	80

YBR143C	93
YBR144C	0
YBR145W	3
YBR146W	36
YBR149W	6
YBR150C	0
YBR152W	0
YBR154C	90
YBR155W	84
YBR156C	7
YBR157C	2
YBR158W	18
YBR159W	92
YBR160W	95
YBR161W	12
YBR162C	13
YBR162W-A	5
YBR163W	11
YBR166C	25
YBR167C	10
YBR168W	2
YBR169C	35
YBR170C	85
YBR171W	15
YBR172C	25
YBR173C	45
YBR174C	0
YBR175W	79
YBR176W	32
YBR177C	10
YBR178W	0
YBR179C	24
YBR180W	14
YBR182C	31
YBR183W	17
YBR184W	0
YBR185C	6
YBR186W	65
YBR187W	81
YBR188C	1
YBR190W	0
YBR192W	78
YBR193C	10
YBR194W	2
YBR195C	6

YBR196C	96
YBR197C	2
YBR198C	90
YBR199W	10
YBR200W	20
YBR201W	14
YBR202W	92
YBR203W	4
YBR204C	3
YBR205W	4
YBR207W	5
YBR208C	62
YBR209W	0
YBR211C	2
YBR212W	21
YBR213W	16
YBR214W	14
YBR215W	11
YBR216C	5
YBR217W	32
YBR218C	34
YBR219C	0
YBR220C	86
YBR221C	93
YBR222C	83
YBR223C	62
YBR225W	2
YBR226C	0
YBR227C	87
YBR228W	62
YBR229C	92
YBR230C	2
YBR231C	57
YBR233W	78
YBR234C	85
YBR235W	76
YBR236C	86
YBR237W	89
YBR238C	4
YBR239C	15
YBR240C	2
YBR241C	76
YBR242W	41
YBR243C	92
YBR244W	62

YBR245C	18
YBR246W	40
YBR247C	92
YBR248C	34
YBR249C	22
YBR250W	1
YBR251W	21
YBR252W	87
YBR253W	3
YBR254C	84
YBR255W	5
YBR257W	38
YBR258C	2
YBR260C	61
YBR261C	88
YBR262C	2
YBR263W	14
YBR264C	1
YBR265W	63
YBR266C	0
YBR267W	88
YBR269C	21
YBR270C	3
YBR271W	18
YBR272C	4
YBR273C	26
YBR274W	60
YBR275C	5
YBR276C	16
YBR277C	0
YBR278W	11
YBR279W	47
YBR280C	13
YBR281C	19
YBR282W	11
YBR283C	5
YBR284W	2
YBR285W	0
YBR286W	19
YBR287W	26
YBR288C	10
YBR289W	40
YBR290W	15
YBR291C	73
YBR293W	14

YBR295W	8
YBR296C	80
YBR298C	8
YBR299W	7
YBR300C	0
YBR301W	0
YBR302C	0
YCL004W	84
YCL005W	1
YCL007C	0
YCL008C	60
YCL009C	21
YCL010C	14
YCL011C	41
YCL014W	8
YCL016C	58
YCL017C	98
YCL018W	31
YCL019W	4
YCL020W	0
YCL023C	0
YCL024W	29
YCL025C	8
YCL026C-A	0
YCL027W	5
YCL028W	1
YCL029C	23
YCL030C	29
YCL031C	40
YCL032W	7
YCL033C	86
YCL034W	16
YCL035C	36
YCL036W	1
YCL037C	21
YCL038C	17
YCL039W	70
YCL040W	18
YCL042W	0
YCL043C	96
YCL044C	4
YCL045C	79
YCL046W	0
YCL048W	5
YCL049C	1

YCL050C	8
YCL051W	3
YCL052C	8
YCL054W	83
YCL055W	47
YCL056C	1
YCL057W	67
YCL058C	0
YCL059C	90
YCL061C	10
YCL063W	2
YCL064C	47
YCL065W	0
YCL069W	1
YCL073C	0
YCL076W	0
YCR001W	0
YCR002C	47
YCR003W	17
YCR004C	22
YCR005C	3
YCR007C	1
YCR008W	22
YCR009C	54
YCR010C	20
YCR011C	90
YCR012W	96
YCR014C	53
YCR015C	10
YCR016W	17
YCR020C-A	8
YCR021C	4
YCR022C	0
YCR023C	57
YCR024C	89
YCR024C-A	1
YCR027C	44
YCR028C	19
YCR028C-A	15
YCR030C	16
YCR033W	28
YCR034W	59
YCR035C	8
YCR036W	88
YCR037C	5

YCR038C	24
YCR039C	9
YCR040W	2
YCR041W	0
YCR042C	73
YCR044C	71
YCR045C	11
YCR046C	13
YCR047C	94
YCR048W	24
YCR050C	0
YCR052W	33
YCR053W	56
YCR054C	12
YCR057C	95
YCR059C	63
YCR060W	8
YCR061W	16
YCR063W	93
YCR065W	39
YCR066W	55
YCR067C	5
YCR068W	19
YCR071C	14
YCR072C	96
YCR073C	27
YCR075C	70
YCR076C	1
YCR077C	40
YCR079W	16
YCR081W	10
YCR082W	2
YCR084C	48
YCR086W	4
YCR087C-A	22
YCR087W	0
YCR088W	70
YCR091W	23
YCR092C	53
YCR093W	92
YCR094W	87
YCR095C	7
YCR096C	1
YCR097W	4
YCR098C	25

YCR099C	0
YCR100C	0
YCR101C	0
YCR105W	10
YCR106W	2
YCR107W	0
YDL001W	49
YDL002C	29
YDL003W	63
YDL004W	84
YDL005C	2
YDL006W	37
YDL007W	96
YDL008W	59
YDL011C	0
YDL012C	2
YDL013W	4
YDL014W	96
YDL015C	94
YDL016C	0
YDL017W	61
YDL019C	30
YDL020C	11
YDL022W	81
YDL023C	0
YDL024C	18
YDL025C	9
YDL026W	0
YDL027C	2
YDL028C	34
YDL029W	87
YDL030W	92
YDL031W	90
YDL035C	10
YDL036C	43
YDL037C	0
YDL040C	93
YDL041W	0
YDL042C	23
YDL043C	79
YDL044C	2
YDL045C	41
YDL045W-A	14
YDL046W	26
YDL047W	67

YDL049C	5
YDL050C	0
YDL051W	71
YDL052C	82
YDL053C	2
YDL054C	17
YDL055C	94
YDL056W	20
YDL058W	52
YDL059C	9
YDL060W	95
YDL061C	14
YDL062W	0
YDL063C	15
YDL064W	90
YDL065C	35
YDL066W	75
YDL067C	17
YDL069C	0
YDL070W	30
YDL071C	0
YDL072C	61
YDL073W	3
YDL074C	73
YDL075W	66
YDL076C	3
YDL077C	30
YDL078C	8
YDL079C	5
YDL080C	1
YDL081C	81
YDL082W	58
YDL084W	94
YDL085W	7
YDL086W	17
YDL087C	88
YDL088C	5
YDL089W	3
YDL090C	88
YDL091C	76
YDL092W	6
YDL093W	1
YDL094C	0
YDL095W	21
YDL096C	0

YDL097C	97
YDL098C	79
YDL099W	2
YDL100C	94
YDL101C	38
YDL102W	92
YDL103C	94
YDL104C	80
YDL105W	41
YDL106C	32
YDL108W	80
YDL110C	3
YDL111C	27
YDL112W	15
YDL113C	17
YDL114W	76
YDL115C	7
YDL116W	56
YDL117W	21
YDL118W	0
YDL119C	57
YDL121C	2
YDL123W	11
YDL124W	15
YDL125C	95
YDL126C	96
YDL127W	12
YDL128W	35
YDL129W	2
YDL130W	14
YDL131W	52
YDL132W	94
YDL133W	9
YDL134C	34
YDL135C	84
YDL137W	83
YDL138W	20
YDL139C	2
YDL140C	91
YDL143W	97
YDL144C	17
YDL145C	94
YDL146W	15
YDL147W	97
YDL148C	81

YDL149W	57
YDL150W	51
YDL153C	66
YDL154W	47
YDL155W	44
YDL156W	52
YDL157C	5
YDL158C	0
YDL159W	53
YDL159W-A	0
YDL160C	93
YDL161W	21
YDL162C	0
YDL164C	93
YDL165W	18
YDL166C	91
YDL167C	14
YDL168W	84
YDL171C	62
YDL172C	0
YDL173W	3
YDL175C	8
YDL176W	2
YDL178W	84
YDL179W	2
YDL180W	2
YDL181W	11
YDL183C	15
YDL185W	17
YDL186W	2
YDL188C	71
YDL189W	24
YDL190C	91
YDL191W	97
YDL192W	43
YDL193W	53
YDL195W	95
YDL196W	0
YDL198C	23
YDL199C	2
YDL200C	19
YDL201W	91
YDL202W	12
YDL203C	7
YDL204W	2

YDL206W	72
YDL207W	52
YDL208W	90
YDL209C	29
YDL210W	39
YDL211C	0
YDL212W	16
YDL213C	21
YDL214C	7
YDL215C	29
YDL216C	16
YDL217C	77
YDL218W	3
YDL219W	85
YDL220C	2
YDL221W	0
YDL223C	0
YDL224C	21
YDL225W	4
YDL226C	81
YDL229W	5
YDL230W	81
YDL231C	11
YDL232W	1
YDL233W	6
YDL235C	10
YDL236W	88
YDL237W	12
YDL238C	75
YDL239C	1
YDL240W	25
YDL241W	0
YDL243C	6
YDL245C	0
YDL246C	21
YDL248W	0
YDR001C	52
YDR002W	76
YDR003W	4
YDR004W	52
YDR005C	48
YDR006C	43
YDR007W	17
YDR009W	8
YDR010C	0

YDR012W95
YDR013W76
YDR016C 10
YDR017C 49
YDR018C 36
YDR020C 41
YDR021W75
YDR022C 1
YDR023W95
YDR024W0
YDR026C 22
YDR027C 40
YDR028C 13
YDR030C 8
YDR031W9
YDR032C 15
YDR034C 6
YDR036C 87
YDR037W99
YDR038C 13
YDR039C 16
YDR040C 4
YDR041W20
YDR042C 0
YDR043C 27
YDR044W83
YDR045C 84
YDR046C 1
YDR049W65
YDR050C 96
YDR051C 13
YDR052C 17
YDR054C 65
YDR055W1
YDR056C 3
YDR060W85
YDR061W19
YDR062W91
YDR063W37
YDR066C 4
YDR067C 10
YDR068W20
YDR069C 23
YDR070C 2
YDR071C 35

YDR072C 6
YDR073W2
YDR074W31
YDR075W32
YDR076W2
YDR077W5
YDR078C 2
YDR079C-A 48
YDR079W13
YDR080W76
YDR081C 20
YDR082W3
YDR084C 82
YDR085C 4
YDR086C 82
YDR087C 50
YDR088C 62
YDR091C 88
YDR092W94
YDR093W15
YDR095C 0
YDR096W23
YDR097C 92
YDR098C 27
YDR099W73
YDR100W13
YDR101C 20
YDR102C 0
YDR103W4
YDR104C 10
YDR105C 86
YDR106W3
YDR107C 11
YDR108W11
YDR110W5
YDR111C 45
YDR113C 2
YDR115W17
YDR116C 30
YDR118W2
YDR119W1
YDR120C 96
YDR121W24
YDR122W28
YDR123C 2

YDR126W20
YDR127W22
YDR128W62
YDR129C 81
YDR130C 2
YDR131C 4
YDR132C 9
YDR133C 0
YDR135C 93
YDR136C 0
YDR137W12
YDR138W2
YDR139C 16
YDR140W88
YDR141C 63
YDR142C 71
YDR143C 25
YDR144C 2
YDR145W22
YDR146C 20
YDR148C 89
YDR150W9
YDR151C 7
YDR152W73
YDR155C 96
YDR156W2
YDR158W33
YDR159W36
YDR160W5
YDR162C 16
YDR164C 91
YDR165W32
YDR166C 47
YDR167W65
YDR168W63
YDR169C 9
YDR170C 85
YDR170W-A 0
YDR171W6
YDR172W96
YDR173C 55
YDR174W20
YDR175C 11
YDR176W19
YDR177W84

YDR178W5
YDR179C 2
YDR180W50
YDR181C 2
YDR183W87
YDR184C 3
YDR186C 2
YDR188W98
YDR189W96
YDR190C 95
YDR192C 9
YDR194C 13
YDR195W2
YDR196C 87
YDR198C 28
YDR200C 20
YDR201W5
YDR202C 5
YDR205W23
YDR206W19
YDR207C 2
YDR208W67
YDR211W92
YDR212W93
YDR213W10
YDR214W84
YDR215C 0
YDR216W5
YDR217C 11
YDR218C 2
YDR219C 2
YDR221W73
YDR223W1
YDR224C 92
YDR225W74
YDR226W97
YDR227W1
YDR228C 45
YDR229W10
YDR231C 9
YDR233C 35
YDR235W27
YDR236C 75
YDR237W27
YDR238C 96

YDR239C 2
YDR240C 2
YDR242W70
YDR243C 83
YDR244W68
YDR245W15
YDR246W77
YDR247W12
YDR249C 2
YDR250C 0
YDR251W0
YDR252W0
YDR253C 21
YDR254W12
YDR255C 75
YDR256C 78
YDR259C 3
YDR260C 1
YDR261W-A 0
YDR261W-B 1
YDR262W7
YDR263C 33
YDR264C 75
YDR265W75
YDR266C 73
YDR267C 93
YDR268W79
YDR269C 0
YDR270W92
YDR271C 0
YDR273W0
YDR274C 0
YDR275W0
YDR276C 31
YDR277C 4
YDR279W3
YDR280W92
YDR281C 0
YDR283C 70
YDR284C 84
YDR285W5
YDR286C 11
YDR287W64
YDR288W8
YDR289C 50

YDR290W0
YDR291W34
YDR292C 94
YDR293C 22
YDR295C 4
YDR296W5
YDR297W30
YDR298C 88
YDR299W76
YDR300C 28
YDR301W83
YDR303C 1
YDR304C 56
YDR305C 35
YDR306C 9
YDR307W2
YDR308C 29
YDR309C 2
YDR310C 1
YDR311W60
YDR312W54
YDR313C 16
YDR314C 3
YDR315C 7
YDR316W10
YDR317W2
YDR318W4
YDR319C 12
YDR320C 27
YDR321W18
YDR322C-A 13
YDR322W19
YDR323C 62
YDR324C 75
YDR326C 19
YDR327W0
YDR328C 94
YDR329C 44
YDR330W57
YDR331W94
YDR332W25
YDR333C 67
YDR334W64
YDR335W55
YDR337W25

YDR339C 93
YDR340W0
YDR341C 79
YDR342C 7
YDR343C 9
YDR344C 0
YDR345C 13
YDR346C 19
YDR347W6
YDR348C 13
YDR349C 5
YDR350C 2
YDR351W1
YDR353W35
YDR356W29
YDR357C 4
YDR359C 16
YDR361C 57
YDR362C 12
YDR363W6
YDR363W-A 6
YDR364C 94
YDR365C 88
YDR366C 0
YDR367W11
YDR368W24
YDR369C 2
YDR370C 2
YDR371W78
YDR372C 70
YDR373W78
YDR374C 37
YDR376W86
YDR377W17
YDR378C 72
YDR379W36
YDR380W4
YDR381W65
YDR382W85
YDR383C 1
YDR384C 2
YDR386W71
YDR387C 19
YDR388W53
YDR389W45

YDR390C 89
YDR392W44
YDR394W92
YDR395W5
YDR397C 80
YDR398W60
YDR399W6
YDR400W69
YDR403W7
YDR404C 91
YDR405W13
YDR406W15
YDR407C 49
YDR408C 29
YDR409W32
YDR410C 88
YDR412W21
YDR413C 0
YDR414C 23
YDR415C 21
YDR416W85
YDR419W76
YDR421W14
YDR422C 3
YDR423C 1
YDR424C 95
YDR425W13
YDR427W93
YDR428C 17
YDR429C 83
YDR430C 81
YDR431W0
YDR432W59
YDR433W0
YDR435C 88
YDR436W16
YDR438W12
YDR439W1
YDR440W23
YDR441C 0
YDR442W0
YDR443C 10
YDR444W5
YDR445C 0
YDR446W3

YDR448W87
YDR449C 29
YDR450W91
YDR452W76
YDR453C 5
YDR454C 93
YDR455C 0
YDR456W94
YDR457W55
YDR459C 11
YDR460W79
YDR461W0
YDR462W9
YDR463W5
YDR464W4
YDR465C 27
YDR466W33
YDR468C 23
YDR469W11
YDR472W62
YDR473C 78
YDR475C 4
YDR477W87
YDR478W3
YDR479C 15
YDR480W0
YDR482C 10
YDR483W4
YDR484W82
YDR485C 56
YDR487C 28
YDR488C 43
YDR489W78
YDR490C 10
YDR492W20
YDR496C 90
YDR497C 30
YDR498C 9
YDR499W4
YDR500C 13
YDR501W9
YDR502C 35
YDR503C 16
YDR504C 0
YDR505C 13

YDR506C	4
YDR507C	33
YDR508C	6
YDR509W	0
YDR510W	95
YDR511W	61
YDR512C	2
YDR513W	66
YDR514C	5
YDR515W	8
YDR516C	1
YDR517W	30
YDR520C	5
YDR522C	1
YDR523C	82
YDR524C	2
YDR526C	0
YDR527W	14
YDR528W	0
YDR529C	71
YDR530C	17
YDR531W	79
YDR532C	2
YDR533C	25
YDR534C	1
YDR536W	16
YDR538W	5
YDR541C	25
YDR542W	0
YDR545W	0
YEL002C	89
YEL003W	91
YEL004W	67
YEL005C	2
YEL006W	14
YEL008W	0
YEL009C	33
YEL011W	86
YEL012W	87
YEL013W	66
YEL015W	20
YEL016C	2
YEL017C-A	0
YEL017W	2
YEL018W	1

YEL019C	12
YEL020C	69
YEL020W-A	79
YEL021W	71
YEL022W	49
YEL023C	14
YEL024W	81
YEL026W	91
YEL027W	85
YEL030W	0
YEL031W	92
YEL032W	91
YEL034W	62
YEL036C	17
YEL037C	94
YEL038W	59
YEL039C	29
YEL041W	39
YEL042W	73
YEL043W	8
YEL046C	74
YEL047C	24
YEL048C	2
YEL049W	0
YEL050C	56
YEL051W	98
YEL053C	63
YEL054C	95
YEL055C	49
YEL056W	94
YEL057C	0
YEL058W	90
YEL059W	0
YEL060C	36
YEL061C	59
YEL062W	62
YEL063C	20
YEL064C	68
YEL065W	9
YEL066W	5
YEL068C	0
YEL069C	3
YEL070W	8
YEL071W	1
YEL072W	2

YEL074W	0
YEL075C	0
YEL077C	0
YER001W	3
YER002W	21
YER003C	94
YER004W	54
YER005W	58
YER006W	84
YER007C-A	89
YER007W	80
YER008C	22
YER009W	91
YER010C	19
YER011W	1
YER012W	92
YER013W	87
YER014W	66
YER015W	89
YER016W	91
YER017C	27
YER018C	1
YER019C-A	36
YER019W	50
YER020W	23
YER021W	93
YER022W	11
YER023W	93
YER025W	89
YER026C	16
YER027C	33
YER028C	31
YER029C	53
YER030W	5
YER031C	70
YER032W	2
YER033C	2
YER035W	0
YER036C	87
YER037W	5
YER038C	2
YER040W	22
YER041W	23
YER043C	96
YER044C	49

YER045C	19
YER047C	49
YER048C	35
YER049W	62
YER051W	31
YER052C	33
YER053C	85
YER054C	22
YER055C	31
YER056C	12
YER057C	70
YER058W	22
YER059W	15
YER060W-A	2
YER061C	86
YER062C	7
YER063W	9
YER064C	1
YER065C	31
YER066C-A	0
YER066W	3
YER067W	2
YER068W	59
YER069W	30
YER070W	75
YER071C	5
YER073W	35
YER075C	34
YER076C	0
YER077C	4
YER078C	78
YER079W	1
YER081W	68
YER082C	94
YER083C	5
YER084W	0
YER086W	46
YER087C-B	24
YER087W	58
YER088C	6
YER089C	62
YER090W	30
YER091C	31
YER092W	2
YER093C	63

YER094C	96
YER095W	86
YER096W	2
YER098W	53
YER099C	16
YER100W	88
YER101C	9
YER103W	15
YER104W	2
YER105C	4
YER106W	1
YER107C	96
YER109C	13
YER110C	70
YER111C	8
YER112W	78
YER113C	4
YER114C	22
YER115C	0
YER116C	14
YER117W	93
YER118C	16
YER119C	35
YER120W	59
YER121W	0
YER122C	81
YER123W	2
YER124C	5
YER125W	79
YER126C	94
YER127W	50
YER128W	14
YER129W	28
YER131W	84
YER132C	3
YER133W	98
YER136W	98
YER137C	3
YER138C	1
YER139C	12
YER140W	66
YER142C	25
YER143W	57
YER144C	22
YER145C	20

YER146W	84
YER147C	2
YER148W	97
YER149C	3
YER150W	3
YER151C	55
YER152C	40
YER153C	2
YER154W	85
YER155C	11
YER156C	93
YER157W	77
YER158C	2
YER159C	23
YER159C-A	0
YER160C	1
YER161C	5
YER162C	74
YER163C	85
YER164W	88
YER165W	96
YER166W	37
YER167W	2
YER168C	71
YER169W	16
YER170W	59
YER171W	93
YER172C	84
YER173W	49
YER174C	67
YER175C	28
YER176W	29
YER177W	33
YER178W	93
YER179W	46
YER180C	1
YER181C	0
YER182W	14
YER183C	71
YER184C	2
YER186C	0
YER188W	0
YER189W	0
YFL001W	81
YFL002C	88

YFL002W-B	0
YFL003C	61
YFL004W	11
YFL005W	74
YFL007W	63
YFL008W	88
YFL009W	51
YFL010C	12
YFL010W-A	0
YFL011W	7
YFL012W	0
YFL013C	12
YFL014W	12
YFL015C	0
YFL016C	86
YFL017C	82
YFL017W-A	82
YFL018C	93
YFL020C	0
YFL021W	12
YFL022C	91
YFL023W	8
YFL024C	55
YFL025C	57
YFL026W	10
YFL027C	32
YFL028C	47
YFL029C	11
YFL030W	82
YFL031W	5
YFL033C	26
YFL034C-B	15
YFL034W	38
YFL036W	78
YFL037W	97
YFL038C	98
YFL039C	98
YFL040W	4
YFL041W	28
YFL042C	49
YFL044C	76
YFL045C	91
YFL047W	31
YFL048C	16
YFL049W	4

YFL050C	4
YFL051C	0
YFL052W	1
YFL053W	54
YFL054C	8
YFL055W	51
YFL056C	2
YFL058W	4
YFL059W	16
YFL060C	5
YFL061W	7
YFL062W	0
YFL063W	0
YFL065C	0
YFL066C	0
YFL067W	1
YFR001W	10
YFR002W	81
YFR003C	36
YFR004W	98
YFR006W	81
YFR007W	28
YFR008W	2
YFR009W	88
YFR010W	93
YFR011C	8
YFR012W	0
YFR013W	4
YFR014C	66
YFR015C	72
YFR016C	4
YFR017C	1
YFR018C	65
YFR019W	74
YFR021W	70
YFR022W	5
YFR024C-A	21
YFR026C	0
YFR027W	44
YFR028C	85
YFR029W	5
YFR030W	29
YFR031C	89
YFR032C	5
YFR033C	17

YFR034C	5
YFR036W	1
YFR037C	38
YFR038W	47
YFR039C	1
YFR040W	7
YFR042W	3
YFR043C	4
YFR044C	80
YFR045W	5
YFR046C	1
YFR047C	49
YFR049W	5
YFR050C	94
YFR051C	93
YFR052W	86
YFR053C	65
YFR054C	0
YFR055W	5
YFR057W	0
YGL001C	92
YGL003C	93
YGL004C	27
YGL005C	4
YGL006W	89
YGL008C	21
YGL009C	26
YGL010W	27
YGL011C	93
YGL012W	24
YGL013C	4
YGL014W	27
YGL015C	0
YGL016W	13
YGL017W	64
YGL018C	66
YGL019W	25
YGL020C	9
YGL021W	10
YGL022W	93
YGL023C	17
YGL024W	0
YGL025C	1
YGL026C	35
YGL027C	80

YGL028C	8
YGL029W	9
YGL030W	91
YGL032C	2
YGL035C	37
YGL036W	2
YGL037C	65
YGL038C	12
YGL039W	5
YGL040C	80
YGL042C	0
YGL043W	89
YGL044C	69
YGL045W	21
YGL047W	74
YGL048C	95
YGL049C	32
YGL050W	42
YGL051W	0
YGL052W	0
YGL053W	0
YGL054C	83
YGL055W	76
YGL057C	0
YGL058W	95
YGL059W	62
YGL060W	4
YGL061C	2
YGL062W	55
YGL063W	23
YGL064C	14
YGL065C	89
YGL066W	29
YGL068W	89
YGL069C	0
YGL070C	85
YGL071W	3
YGL072C	0
YGL073W	45
YGL074C	0
YGL075C	2
YGL077C	15
YGL078C	28
YGL079W	5
YGL081W	0

YGL083W	73
YGL084C	46
YGL085W	19
YGL086W	27
YGL087C	91
YGL090W	1
YGL091C	94
YGL092W	46
YGL093W	15
YGL094C	74
YGL095C	91
YGL096W	11
YGL097W	93
YGL098W	13
YGL099W	90
YGL100W	71
YGL102C	0
YGL104C	4
YGL105W	53
YGL106W	17
YGL110C	10
YGL111W	54
YGL112C	83
YGL113W	3
YGL114W	23
YGL115W	75
YGL116W	22
YGL117W	1
YGL119W	85
YGL120C	89
YGL121C	1
YGL122C	41
YGL124C	78
YGL125W	64
YGL126W	9
YGL127C	24
YGL128C	4
YGL129C	21
YGL130W	61
YGL131C	17
YGL132W	0
YGL133W	46
YGL134W	4
YGL137W	93
YGL138C	3

YGL139W	4
YGL141W	63
YGL142C	80
YGL143C	89
YGL144C	18
YGL145W	8
YGL146C	1
YGL147C	35
YGL148W	36
YGL149W	0
YGL150C	64
YGL151W	3
YGL153W	57
YGL154C	26
YGL155W	23
YGL156W	44
YGL157W	18
YGL158W	0
YGL161C	65
YGL162W	3
YGL163C	91
YGL165C	0
YGL166W	4
YGL167C	97
YGL168W	0
YGL169W	51
YGL170C	0
YGL171W	87
YGL172W	22
YGL173C	70
YGL174W	57
YGL175C	4
YGL177W	0
YGL178W	13
YGL179C	65
YGL180W	66
YGL181W	26
YGL183C	69
YGL184C	19
YGL185C	3
YGL186C	9
YGL187C	49
YGL189C	61
YGL190C	88
YGL192W	25

YGL194C	13
YGL195W	72
YGL197W	7
YGL198W	43
YGL200C	78
YGL201C	96
YGL202W	56
YGL204C	0
YGL205W	85
YGL206C	96
YGL207W	93
YGL208W	57
YGL210W	35
YGL212W	19
YGL213C	13
YGL214W	0
YGL215W	17
YGL216W	73
YGL217C	0
YGL220W	62
YGL221C	62
YGL222C	2
YGL223C	3
YGL225W	80
YGL226C-A	2
YGL226W	2
YGL227W	23
YGL229C	1
YGL230C	0
YGL233W	72
YGL234W	81
YGL235W	0
YGL236C	79
YGL237C	24
YGL238W	96
YGL239C	0
YGL240W	80
YGL241W	69
YGL242C	48
YGL244W	71
YGL245W	50
YGL246C	69
YGL247W	5
YGL249W	2
YGL250W	3

YGL251C	44
YGL252C	14
YGL253W	25
YGL254W	34
YGL256W	74
YGL258W	0
YGL259W	0
YGL263W	0
YGR002C	80
YGR003W	69
YGR004W	4
YGR005C	70
YGR006W	60
YGR009C	14
YGR010W	53
YGR013W	8
YGR014W	7
YGR016W	3
YGR017W	18
YGR018C	0
YGR020C	93
YGR021W	65
YGR023W	1
YGR024C	77
YGR026W	4
YGR028W	83
YGR029W	77
YGR030C	2
YGR031W	82
YGR032W	17
YGR033C	43
YGR034W	88
YGR035C	0
YGR036C	71
YGR037C	87
YGR038W	61
YGR040W	13
YGR041W	0
YGR042W	4
YGR045C	0
YGR046W	78
YGR047C	81
YGR048W	87
YGR049W	1
YGR050C	0

YGR052W6
YGR053C 9
YGR054W87
YGR055W78
YGR056W20
YGR057C 12
YGR058W45
YGR059W3
YGR060W79
YGR061C 74
YGR062C 50
YGR063C 85
YGR064W0
YGR065C 3
YGR066C 29
YGR067C 2
YGR068C 3
YGR069W0
YGR070W2
YGR071C 2
YGR072W9
YGR073C 0
YGR074W89
YGR075C 2
YGR076C 11
YGR077C 2
YGR078C 89
YGR079W2
YGR080W77
YGR081C 4
YGR082W61
YGR083C 84
YGR084C 2
YGR085C 92
YGR086C 14
YGR087C 7
YGR088W9
YGR089W11
YGR090W86
YGR091W86
YGR092W7
YGR094W97
YGR095C 65
YGR097W5
YGR098C 16

YGR099W13
YGR100W43
YGR101W72
YGR102C 3
YGR103W91
YGR104C 7
YGR105W12
YGR107W0
YGR108W17
YGR109C 6
YGR110W20
YGR111W13
YGR113W10
YGR114C 0
YGR115C 0
YGR116W88
YGR117C 4
YGR119C 64
YGR120C 2
YGR121C 14
YGR122W2
YGR123C 84
YGR124W47
YGR125W23
YGR126W2
YGR128C 5
YGR129W4
YGR130C 2
YGR131W1
YGR132C 92
YGR133W18
YGR134W4
YGR135W94
YGR136W14
YGR138C 10
YGR139W0
YGR140W2
YGR141W17
YGR142W2
YGR143W4
YGR144W23
YGR145W93
YGR146C 0
YGR148C 43
YGR149W30

YGR150C	7
YGR151C	0
YGR152C	53
YGR153W	0
YGR154C	0
YGR155W	88
YGR156W	4
YGR157W	20
YGR158C	12
YGR159C	81
YGR160W	0
YGR161C	1
YGR161C-D	0
YGR161W-B	5
YGR162W	45
YGR163W	81
YGR165W	8
YGR166W	4
YGR167W	23
YGR168C	1
YGR169C	6
YGR170W	19
YGR171C	92
YGR172C	94
YGR173W	76
YGR174C	5
YGR175C	54
YGR177C	4
YGR178C	65
YGR179C	2
YGR180C	1
YGR181W	65
YGR182C	0
YGR184C	64
YGR185C	85
YGR186W	20
YGR187C	74
YGR188C	53
YGR189C	17
YGR191W	29
YGR192C	50
YGR193C	16
YGR194C	77
YGR195W	97
YGR196C	3

YGR197C 12
YGR198W6
YGR199W3
YGR200C 84
YGR201C 3
YGR202C 56
YGR203W24
YGR204W54
YGR205W30
YGR206W1
YGR207C 85
YGR208W74
YGR209C 65
YGR210C 23
YGR211W93
YGR213C 8
YGR214W60
YGR215W19
YGR216C 35
YGR217W82
YGR218W94
YGR219W0
YGR220C 89
YGR221C 1
YGR222W3
YGR223C 76
YGR224W5
YGR225W8
YGR227W71
YGR228W0
YGR229C 13
YGR231C 90
YGR232W89
YGR233C 20
YGR234W22
YGR235C 10
YGR236C 1
YGR237C 3
YGR238C 30
YGR239C 2
YGR240C 33
YGR241C 53
YGR242W0
YGR243W72
YGR244C 95

YGR245C 92
YGR246C 89
YGR247W6
YGR249W18
YGR250C 27
YGR251W3
YGR252W52
YGR253C 93
YGR254W86
YGR255C 81
YGR256W6
YGR257C 87
YGR258C 73
YGR260W40
YGR261C 81
YGR262C 80
YGR263C 11
YGR264C 80
YGR266W19
YGR267C 76
YGR268C 7
YGR269W0
YGR270W61
YGR271W82
YGR274C 74
YGR275W2
YGR276C 50
YGR277C 23
YGR278W88
YGR279C 9
YGR280C 55
YGR281W15
YGR282C 18
YGR283C 34
YGR284C 71
YGR285C 70
YGR289C 5
YGR290W0
YGR291C 0
YGR292W8
YGR293C 0
YGR294W0
YGR295C 0
YGR296W0
YHL001W74

YHL002W	72
YHL003C	66
YHL004W	61
YHL005C	0
YHL006C	1
YHL007C	81
YHL008C	6
YHL009C	10
YHL010C	82
YHL011C	27
YHL014C	4
YHL015W	97
YHL016C	64
YHL017W	26
YHL018W	37
YHL019C	4
YHL020C	8
YHL021C	67
YHL022C	53
YHL023C	22
YHL024W	15
YHL025W	2
YHL026C	3
YHL027W	22
YHL029C	5
YHL030W	68
YHL031C	82
YHL032C	95
YHL033C	34
YHL034C	7
YHL035C	5
YHL036W	29
YHL038C	1
YHL039W	39
YHL042W	0
YHL043W	0
YHL044W	0
YHL045W	0
YHL046C	0
YHL047C	2
YHL048W	0
YHL049C	0
YHL050C	0
YHR001W	69
YHR002W	69

YHR003C	0
YHR004C	20
YHR005C	83
YHR005C-A	80
YHR007C	85
YHR008C	95
YHR009C	33
YHR011W	72
YHR012W	92
YHR013C	94
YHR014W	0
YHR015W	5
YHR016C	27
YHR018C	73
YHR019C	79
YHR020W	62
YHR022C	2
YHR023W	69
YHR024C	87
YHR025W	31
YHR026W	92
YHR027C	96
YHR028C	82
YHR030C	40
YHR031C	20
YHR032W	8
YHR033W	6
YHR034C	10
YHR035W	3
YHR036W	16
YHR038W	22
YHR039C	29
YHR039C-A	72
YHR040W	43
YHR041C	6
YHR042W	94
YHR043C	5
YHR044C	3
YHR045W	12
YHR046C	28
YHR047C	55
YHR048W	11
YHR049W	13
YHR050W	28
YHR051W	69

YHR052W	36
YHR053C	0
YHR056C	0
YHR057C	18
YHR058C	63
YHR059W	11
YHR060W	9
YHR061C	1
YHR062C	60
YHR063C	20
YHR064C	17
YHR065C	92
YHR066W	49
YHR067W	15
YHR068W	96
YHR069C	86
YHR070W	87
YHR071W	6
YHR072W	54
YHR072W-A	83
YHR073W	43
YHR074W	84
YHR075C	82
YHR076W	88
YHR077C	74
YHR078W	19
YHR079C	72
YHR079C-A	3
YHR080C	6
YHR081W	6
YHR082C	16
YHR083W	5
YHR084W	9
YHR085W	18
YHR086W	53
YHR087W	14
YHR088W	90
YHR089C	85
YHR090C	57
YHR091C	22
YHR092C	3
YHR094C	1
YHR096C	15
YHR097C	3
YHR098C	26

YHR099W	74
YHR100C	24
YHR102W	71
YHR103W	5
YHR105W	4
YHR106W	9
YHR107C	28
YHR108W	61
YHR109W	4
YHR110W	14
YHR111W	91
YHR112C	15
YHR113W	74
YHR114W	71
YHR115C	9
YHR116W	7
YHR117W	13
YHR118C	4
YHR119W	41
YHR120W	26
YHR121W	61
YHR122W	92
YHR123W	65
YHR124W	7
YHR127W	2
YHR128W	90
YHR129C	1
YHR130C	0
YHR132C	76
YHR133C	0
YHR134W	23
YHR135C	16
YHR136C	0
YHR137W	7
YHR138C	7
YHR140W	8
YHR142W	17
YHR143W	2
YHR143W-A	64
YHR144C	32
YHR145C	0
YHR147C	31
YHR148W	90
YHR149C	3
YHR151C	9

YHR152W	1
YHR154W	21
YHR156C 41	
YHR157W	0
YHR158C 28	
YHR159W	2
YHR160C 0	
YHR161C 33	
YHR163W	57
YHR164C 64	
YHR165C 93	
YHR166C 87	
YHR167W	2
YHR168W	89
YHR169W	81
YHR170W	95
YHR171W	83
YHR172W	62
YHR174W	13
YHR175W	72
YHR177W	7
YHR178W	9
YHR179W	22
YHR180W	0
YHR182W	2
YHR183W	86
YHR184W	2
YHR185C 0	
YHR186C 78	
YHR187W	12
YHR188C 77	
YHR189W	40
YHR190W	54
YHR191C 12	
YHR193C 92	
YHR194W	18
YHR195W	2
YHR196W	3
YHR197W	6
YHR198C 18	
YHR199C 1	
YHR200W	88
YHR201C 63	
YHR203C 95	
YHR204W	79

YHR205W	31
YHR206W	31
YHR207C	64
YHR208W	25
YHR209W	18
YHR211W	3
YHR212W-A	0
YHR214C-B	1
YHR214W-A	0
YHR215W	14
YHR216W	3
YIL001W	45
YIL002C	6
YIL004C	37
YIL005W	25
YIL006W	50
YIL007C	89
YIL008W	78
YIL009C-A	4
YIL009W	5
YIL010W	24
YIL011W	1
YIL013C	4
YIL014W	8
YIL016W	3
YIL017C	15
YIL018W	94
YIL019W	12
YIL020C	30
YIL021W	94
YIL022W	83
YIL023C	78
YIL024C	1
YIL025C	0
YIL026C	51
YIL027C	7
YIL028W	0
YIL029C	0
YIL030C	53
YIL031W	28
YIL032C	0
YIL033C	89
YIL034C	86
YIL035C	76
YIL037C	0

YIL038C	57
YIL039W	20
YIL040W	1
YIL041W	19
YIL042C	54
YIL044C	72
YIL045W	14
YIL046W	58
YIL047C	80
YIL048W	84
YIL049W	71
YIL050W	14
YIL051C	17
YIL052C	81
YIL053W	16
YIL054W	0
YIL055C	1
YIL056W	4
YIL057C	2
YIL059C	0
YIL060W	0
YIL061C	69
YIL062C	68
YIL063C	58
YIL064W	81
YIL065C	74
YIL066C	20
YIL068C	61
YIL070C	37
YIL071C	3
YIL072W	22
YIL074C	32
YIL075C	93
YIL076W	22
YIL077C	3
YIL078W	96
YIL079C	21
YIL082W	0
YIL082W-A	20
YIL084C	9
YIL085C	2
YIL086C	0
YIL088C	39
YIL091C	92
YIL092W	0

YIL093C	19
YIL094C	20
YIL095W	28
YIL096C	26
YIL097W	80
YIL098C	4
YIL101C	3
YIL103W	93
YIL104C	58
YIL105C	15
YIL106W	89
YIL108W	20
YIL109C	91
YIL110W	74
YIL111W	12
YIL112W	45
YIL113W	50
YIL114C	6
YIL115C	11
YIL117C	3
YIL118W	14
YIL119C	0
YIL120W	10
YIL121W	15
YIL122W	1
YIL124W	35
YIL125W	93
YIL126W	43
YIL128W	72
YIL129C	68
YIL130W	17
YIL131C	19
YIL132C	2
YIL134W	40
YIL135C	3
YIL136W	2
YIL137C	5
YIL138C	17
YIL139C	4
YIL140W	14
YIL141W	0
YIL142W	97
YIL143C	93
YIL144W	59
YIL145C	33

YIL147C	25
YIL148W	90
YIL149C	10
YIL150C	33
YIL151C	5
YIL152W	0
YIL153W	45
YIL154C	4
YIL156W	14
YIL157C	13
YIL158W	0
YIL159W	48
YIL160C	58
YIL162W	22
YIL163C	0
YIL166C	16
YIL169C	2
YIL172C	28
YIL173W	16
YIL177C	0
YIR001C	63
YIR002C	55
YIR003W	0
YIR004W	36
YIR005W	85
YIR006C	29
YIR008C	87
YIR009W	18
YIR010W	3
YIR011C	13
YIR012W	50
YIR013C	2
YIR014W	2
YIR016W	0
YIR017C	3
YIR018W	1
YIR021W	0
YIR022W	91
YIR023W	15
YIR024C	2
YIR025W	2
YIR026C	80
YIR027C	79
YIR028W	18
YIR031C	17

YIR032C	13
YIR033W	13
YIR034C	23
YIR035C	36
YIR036C	15
YIR037W	40
YIR038C	16
YIR039C	3
YIR040C	0
YIR042C	21
YJL001W	95
YJL002C	83
YJL004C	62
YJL005W	26
YJL006C	27
YJL008C	96
YJL010C	69
YJL011C	38
YJL012C	30
YJL013C	13
YJL014W	94
YJL015C	0
YJL016W	2
YJL019W	3
YJL020C	17
YJL023C	2
YJL024C	84
YJL025W	8
YJL026W	92
YJL029C	88
YJL030W	87
YJL031C	71
YJL032W	0
YJL033W	87
YJL034W	83
YJL035C	74
YJL036W	67
YJL039C	39
YJL041W	64
YJL042W	7
YJL043W	0
YJL044C	10
YJL045W	13
YJL047C	2
YJL048C	22

YJL050W	91
YJL052W	46
YJL053W	91
YJL054W	19
YJL055W	26
YJL056C	47
YJL057C	14
YJL058C	4
YJL059W	70
YJL060W	89
YJL061W	9
YJL062W	57
YJL063C	54
YJL064W	0
YJL065C	11
YJL066C	4
YJL067W	0
YJL068C	78
YJL069C	87
YJL070C	2
YJL071W	18
YJL072C	75
YJL074C	85
YJL075C	0
YJL076W	4
YJL078C	4
YJL079C	60
YJL080C	72
YJL081C	19
YJL082W	32
YJL083W	9
YJL084C	13
YJL085W	52
YJL086C	0
YJL087C	18
YJL088W	66
YJL089W	3
YJL090C	12
YJL091C	78
YJL092W	38
YJL093C	12
YJL094C	21
YJL095W	54
YJL096W	33
YJL097W	90

YJL098W	44
YJL099W	2
YJL100W	66
YJL102W	63
YJL106W	45
YJL107C	1
YJL108C	7
YJL109C	76
YJL110C	10
YJL111W	95
YJL112W	34
YJL114W	0
YJL115W	96
YJL117W	4
YJL118W	0
YJL120W	0
YJL122W	4
YJL123C	13
YJL124C	74
YJL125C	94
YJL128C	45
YJL129C	20
YJL130C	81
YJL131C	4
YJL133W	66
YJL134W	40
YJL135W	0
YJL137C	41
YJL138C	93
YJL139C	1
YJL140W	31
YJL141C	76
YJL142C	0
YJL143W	87
YJL145W	20
YJL146W	2
YJL149W	2
YJL151C	2
YJL152W	0
YJL153C	82
YJL154C	90
YJL155C	85
YJL156C	6
YJL157C	9
YJL158C	2

YJL159W	4
YJL160C	0
YJL162C	7
YJL163C	30
YJL164C	19
YJL165C	9
YJL166W	19
YJL167W	85
YJL168C	29
YJL170C	1
YJL172W	68
YJL173C	2
YJL174W	6
YJL175W	0
YJL176C	49
YJL178C	5
YJL179W	23
YJL180C	72
YJL181W	0
YJL182C	0
YJL183W	13
YJL184W	4
YJL185C	0
YJL186W	5
YJL187C	33
YJL189W	75
YJL190C	97
YJL191W	94
YJL192C	2
YJL193W	35
YJL194W	82
YJL195C	0
YJL196C	42
YJL197W	94
YJL198W	29
YJL199C	0
YJL200C	18
YJL201W	22
YJL202C	0
YJL203W	24
YJL204C	19
YJL207C	29
YJL208C	78
YJL209W	1
YJL210W	29

YJL211C	0
YJL212C	23
YJL213W	3
YJL214W	5
YJL215C	0
YJL218W	31
YJL219W	0
YJL222W	16
YJL222W-A	0
YJL222W-B	0
YJL225C	0
YJR001W	48
YJR002W	85
YJR003C	4
YJR004C	3
YJR005W	13
YJR006W	89
YJR007W	93
YJR008W	90
YJR009C	9
YJR010C-A	30
YJR010W	80
YJR011C	0
YJR012C	0
YJR014W	81
YJR015W	3
YJR016C	33
YJR017C	88
YJR019C	50
YJR020W	0
YJR021C	1
YJR022W	61
YJR023C	0
YJR024C	70
YJR025C	43
YJR027W	0
YJR028W	0
YJR029W	0
YJR030C	2
YJR031C	29
YJR032W	12
YJR033C	33
YJR034W	39
YJR035W	67
YJR037W	0

YJR038C	0
YJR040W	91
YJR041C	8
YJR042W	7
YJR043C	2
YJR044C	74
YJR045C	93
YJR047C	51
YJR048W	73
YJR049C	41
YJR050W	65
YJR051W	14
YJR052W	58
YJR053W	7
YJR054W	14
YJR055W	19
YJR056C	4
YJR057W	89
YJR058C	83
YJR059W	6
YJR060W	16
YJR061W	1
YJR062C	16
YJR063W	78
YJR064W	97
YJR065C	86
YJR066W	28
YJR067C	12
YJR068W	96
YJR069C	93
YJR070C	93
YJR072C	92
YJR073C	45
YJR074W	50
YJR075W	11
YJR076C	19
YJR077C	18
YJR079W	0
YJR080C	8
YJR082C	7
YJR083C	2
YJR084W	88
YJR086W	11
YJR087W	0
YJR089W	13

YJR090C	18
YJR091C	18
YJR092W	13
YJR093C	49
YJR094C	2
YJR095W	29
YJR097W	42
YJR098C	10
YJR099W	95
YJR100C	62
YJR101W	14
YJR102C	75
YJR103W	25
YJR104C	81
YJR105W	88
YJR106W	23
YJR108W	0
YJR109C	28
YJR110W	89
YJR112W	11
YJR113C	39
YJR115W	1
YJR116W	45
YJR117W	90
YJR119C	40
YJR120W	0
YJR121W	95
YJR122W	61
YJR123W	95
YJR124C	19
YJR125C	68
YJR126C	61
YJR127C	4
YJR130C	20
YJR131W	86
YJR132W	81
YJR133W	16
YJR134C	31
YJR135C	2
YJR135W-A	73
YJR136C	2
YJR138W	65
YJR139C	32
YJR140C	15
YJR141W	22

YJR143C	38
YJR144W	21
YJR146W	0
YJR148W	68
YJR149W	21
YJR152W	33
YJR154W	4
YJR157W	0
YJR158W	0
YJR159W	45
YJR161C	0
YJR162C	0
YKL001C	28
YKL002W	96
YKL003C	27
YKL004W	19
YKL006C-A	16
YKL007W	84
YKL008C	46
YKL009W	95
YKL010C	80
YKL011C	9
YKL012W	81
YKL013C	79
YKL014C	23
YKL015W	6
YKL016C	53
YKL017C	86
YKL018W	84
YKL019W	89
YKL020C	9
YKL021C	77
YKL022C	67
YKL023W	0
YKL024C	95
YKL025C	75
YKL026C	4
YKL028W	84
YKL029C	90
YKL033W	41
YKL034W	24
YKL035W	91
YKL036C	0
YKL038W	5
YKL039W	61

YKL040C	84
YKL042W	2
YKL043W	4
YKL044W	0
YKL045W	88
YKL047W	34
YKL048C	6
YKL049C	14
YKL050C	2
YKL051W	14
YKL052C	9
YKL054C	13
YKL055C	22
YKL056C	90
YKL057C	6
YKL058W	78
YKL059C	46
YKL060C	28
YKL061W	1
YKL062W	27
YKL063C	2
YKL064W	20
YKL065C	6
YKL067W	98
YKL068W	19
YKL069W	23
YKL070W	8
YKL072W	8
YKL073W	22
YKL074C	19
YKL075C	2
YKL076C	0
YKL077W	5
YKL078W	10
YKL079W	7
YKL080W	94
YKL081W	61
YKL082C	57
YKL084W	6
YKL085W	95
YKL086W	48
YKL087C	29
YKL088W	7
YKL089W	23
YKL090W	10

YKL092C	19
YKL093W	0
YKL095W	91
YKL098W	3
YKL099C	91
YKL101W	34
YKL102C	0
YKL103C	12
YKL104C	95
YKL105C	0
YKL106W	4
YKL107W	9
YKL108W	11
YKL109W	1
YKL110C	73
YKL111C	0
YKL112W	1
YKL113C	95
YKL114C	37
YKL116C	12
YKL117W	83
YKL119C	4
YKL120W	72
YKL122C	4
YKL124W	17
YKL125W	77
YKL126W	42
YKL127W	1
YKL129C	7
YKL130C	2
YKL133C	1
YKL134C	88
YKL135C	95
YKL137W	6
YKL138C	16
YKL139W	79
YKL140W	78
YKL141W	39
YKL142W	5
YKL143W	35
YKL144C	90
YKL145W	89
YKL146W	42
YKL147C	0
YKL148C	83

YKL149C	84
YKL150W	30
YKL152C	76
YKL153W	0
YKL154W	72
YKL155C	49
YKL157W	45
YKL159C	2
YKL160W	75
YKL161C	1
YKL163W	0
YKL164C	4
YKL165C	74
YKL166C	16
YKL167C	8
YKL168C	9
YKL170W	29
YKL171W	14
YKL172W	66
YKL173W	13
YKL174C	13
YKL175W	24
YKL176C	4
YKL177W	0
YKL178C	16
YKL179C	46
YKL180W	82
YKL181W	17
YKL182W	20
YKL183W	4
YKL185W	5
YKL186C	5
YKL188C	26
YKL189W	81
YKL190W	91
YKL191W	80
YKL192C	77
YKL193C	94
YKL194C	14
YKL195W	7
YKL196C	95
YKL197C	61
YKL198C	5
YKL201C	4
YKL202W	0

YKL203C	63
YKL204W	2
YKL205W	63
YKL206C	19
YKL208W	1
YKL209C	90
YKL210W	95
YKL211C	24
YKL212W	91
YKL213C	86
YKL214C	2
YKL215C	82
YKL216W	85
YKL217W	26
YKL218C	71
YKL219W	0
YKL220C	14
YKL221W	56
YKL222C	2
YKL224C	0
YKR001C	39
YKR002W	92
YKR005C	0
YKR006C	4
YKR007W	2
YKR008W	5
YKR009C	70
YKR010C	0
YKR011C	2
YKR014C	7
YKR016W	23
YKR017C	91
YKR018C	54
YKR020W	3
YKR021W	6
YKR022C	0
YKR023W	24
YKR024C	79
YKR025W	12
YKR026C	94
YKR027W	1
YKR028W	29
YKR029C	22
YKR030W	78
YKR031C	73

YKR032W	0
YKR034W	8
YKR035C	0
YKR036C	12
YKR037C	3
YKR038C	91
YKR039W	19
YKR040C	0
YKR043C	14
YKR044W	29
YKR046C	2
YKR048C	93
YKR049C	2
YKR050W	6
YKR051W	87
YKR052C	34
YKR053C	30
YKR054C	87
YKR055W	4
YKR058W	41
YKR060W	18
YKR061W	4
YKR062W	67
YKR063C	44
YKR064W	2
YKR065C	21
YKR066C	34
YKR067W	7
YKR068C	94
YKR069W	21
YKR070W	70
YKR071C	33
YKR072C	2
YKR073C	0
YKR074W	35
YKR075C	2
YKR077W	0
YKR078W	0
YKR079C	90
YKR080W	84
YKR081C	92
YKR082W	20
YKR083C	5
YKR084C	17
YKR085C	10

YKR086W	79
YKR087C	56
YKR088C	38
YKR089C	31
YKR090W	33
YKR091W	1
YKR092C	32
YKR093W	83
YKR095W	50
YKR095W-A	14
YKR096W	4
YKR099W	28
YKR100C	4
YKR101W	0
YKR105C	21
YKR106W	1
YLL001W	86
YLL002W	12
YLL003W	5
YLL004W	19
YLL005C	6
YLL006W	19
YLL008W	88
YLL009C	67
YLL010C	48
YLL011W	93
YLL012W	10
YLL013C	62
YLL014W	8
YLL015W	6
YLL018C	97
YLL019C	70
YLL020C	0
YLL021W	17
YLL022C	1
YLL023C	37
YLL024C	10
YLL026W	35
YLL027W	22
YLL028W	25
YLL029W	94
YLL030C	0
YLL031C	79
YLL032C	8
YLL033W	2

YLL034C	81
YLL036C	60
YLL037W	0
YLL038C	9
YLL040C	82
YLL041C	89
YLL042C	2
YLL043W	9
YLL046C	3
YLL047W	0
YLL048C	22
YLL049W	0
YLL050C	91
YLL051C	12
YLL054C	1
YLL056C	18
YLL059C	0
YLL060C	63
YLL061W	6
YLL062C	52
YLL065W	0
YLL067W-A	0
YLR001C	10
YLR002C	86
YLR003C	14
YLR004C	17
YLR005W	94
YLR006C	5
YLR007W	26
YLR008C	21
YLR009W	92
YLR010C	0
YLR011W	16
YLR012C	0
YLR014C	15
YLR015W	21
YLR016C	34
YLR017W	73
YLR018C	2
YLR019W	35
YLR021W	4
YLR024C	30
YLR025W	89
YLR026C	91
YLR027C	95

YLR028C	20
YLR030W	0
YLR031W	0
YLR032W	72
YLR033W	5
YLR034C	38
YLR035C	3
YLR036C	0
YLR037C	0
YLR038C	79
YLR039C	7
YLR040C	2
YLR042C	0
YLR044C	16
YLR045C	35
YLR046C	0
YLR049C	1
YLR050C	31
YLR051C	54
YLR052W	2
YLR053C	0
YLR054C	2
YLR055C	19
YLR056W	59
YLR057W	11
YLR058C	92
YLR059C	79
YLR060W	96
YLR063W	13
YLR064W	5
YLR065C	25
YLR066W	81
YLR067C	10
YLR068W	5
YLR070C	29
YLR071C	13
YLR072W	7
YLR073C	2
YLR074C	73
YLR075W	96
YLR076C	0
YLR077W	17
YLR078C	22
YLR079W	2
YLR080W	15

YLR081W	2
YLR082C	0
YLR083C	88
YLR085C	43
YLR086W	89
YLR087C	19
YLR088W	76
YLR090W	4
YLR092W	58
YLR093C	12
YLR094C	3
YLR095C	4
YLR096W	35
YLR097C	58
YLR098C	9
YLR099C	53
YLR100W	44
YLR102C	1
YLR103C	84
YLR104W	12
YLR105C	47
YLR106C	71
YLR108C	4
YLR109W	68
YLR110C	3
YLR112W	0
YLR113W	71
YLR114C	68
YLR115W	61
YLR116W	88
YLR117C	92
YLR118C	90
YLR119W	4
YLR120C	9
YLR121C	9
YLR123C	0
YLR124W	0
YLR125W	0
YLR126C	28
YLR127C	78
YLR128W	79
YLR129W	93
YLR130C	53
YLR131C	27
YLR132C	5

YLR133W	74
YLR134W	8
YLR135W	4
YLR136C	16
YLR137W	13
YLR138W	19
YLR140W	0
YLR141W	3
YLR142W	46
YLR143W	54
YLR144C	12
YLR145W	8
YLR146C	7
YLR147C	87
YLR148W	89
YLR149C	16
YLR150W	15
YLR151C	43
YLR152C	10
YLR153C	85
YLR154C	0
YLR158C	15
YLR163C	95
YLR164W	43
YLR166C	79
YLR167W	86
YLR168C	78
YLR170C	91
YLR171W	0
YLR172C	96
YLR173W	4
YLR174W	36
YLR175W	93
YLR176C	37
YLR177W	10
YLR178C	52
YLR180W	71
YLR181C	37
YLR182W	13
YLR183C	8
YLR185W	87
YLR186W	90
YLR187W	13
YLR189C	15
YLR190W	1

YLR191W	67
YLR192C	11
YLR193C	57
YLR194C	2
YLR196W	90
YLR197W	89
YLR198C	0
YLR199C	2
YLR200W	87
YLR201C	56
YLR202C	0
YLR203C	20
YLR204W	0
YLR205C	36
YLR206W	62
YLR207W	75
YLR208W	89
YLR209C	68
YLR210W	37
YLR211C	2
YLR212C	93
YLR213C	7
YLR214W	18
YLR215C	67
YLR216C	82
YLR217W	0
YLR218C	3
YLR219W	2
YLR220W	35
YLR221C	7
YLR222C	90
YLR223C	3
YLR224W	2
YLR225C	0
YLR226W	2
YLR227C	2
YLR228C	5
YLR229C	88
YLR231C	55
YLR233C	0
YLR234W	97
YLR237W	3
YLR238W	12
YLR239C	70
YLR240W	92

YLR241W	60
YLR242C	36
YLR243W	94
YLR245C	73
YLR246W	75
YLR247C	31
YLR248W	17
YLR249W	12
YLR250W	14
YLR251W	82
YLR252W	0
YLR253W	88
YLR254C	0
YLR255C	0
YLR256W-A	0
YLR257W	2
YLR258W	2
YLR259C	98
YLR261C	0
YLR262C	92
YLR263W	2
YLR264W	82
YLR265C	1
YLR266C	2
YLR267W	1
YLR268W	94
YLR269C	0
YLR270W	34
YLR271W	60
YLR273C	1
YLR274W	94
YLR275W	91
YLR276C	93
YLR277C	97
YLR278C	6
YLR281C	59
YLR283W	10
YLR284C	69
YLR285W	57
YLR287C	7
YLR288C	4
YLR289W	91
YLR290C	23
YLR291C	87
YLR292C	9

YLR293C	71
YLR294C	0
YLR295C	12
YLR297W	0
YLR298C	18
YLR299W	82
YLR300W	19
YLR301W	2
YLR303W	21
YLR304C	91
YLR305C	68
YLR306W	75
YLR308W	15
YLR309C	19
YLR310C	36
YLR311C	0
YLR312C	0
YLR312W-A	8
YLR313C	3
YLR314C	73
YLR315W	1
YLR316C	67
YLR317W	0
YLR318W	38
YLR319C	17
YLR320W	2
YLR321C	23
YLR322W	0
YLR323C	76
YLR324W	13
YLR325C	86
YLR326W	13
YLR327C	5
YLR328W	32
YLR329W	2
YLR330W	20
YLR331C	0
YLR332W	2
YLR333C	27
YLR334C	0
YLR335W	36
YLR336C	72
YLR337C	29
YLR338W	0
YLR339C	0

YLR340W	95
YLR342W	11
YLR343W	3
YLR344W	78
YLR345W	16
YLR346C	0
YLR347C	93
YLR348C	86
YLR349W	0
YLR350W	12
YLR351C	83
YLR352W	7
YLR353W	2
YLR354C	85
YLR355C	30
YLR356W	5
YLR357W	24
YLR358C	0
YLR359W	89
YLR361C	28
YLR362W	29
YLR363C	3
YLR363W-A	5
YLR365W	0
YLR366W	0
YLR368W	2
YLR369W	2
YLR370C	83
YLR371W	39
YLR372W	58
YLR373C	0
YLR374C	0
YLR376C	2
YLR377C	86
YLR378C	95
YLR379W	0
YLR381W	4
YLR383W	86
YLR384C	79
YLR385C	2
YLR386W	84
YLR387C	13
YLR388W	77
YLR389C	91
YLR390W	1

YLR390W-A	5
YLR392C	4
YLR393W	22
YLR394W	5
YLR395C	5
YLR396C	86
YLR397C	42
YLR398C	82
YLR399C	64
YLR401C	81
YLR403W	21
YLR406C	89
YLR409C	93
YLR410W	85
YLR410W-B	3
YLR411W	13
YLR412W	22
YLR413W	1
YLR415C	0
YLR416C	0
YLR417W	39
YLR418C	63
YLR420W	35
YLR421C	8
YLR422W	39
YLR423C	9
YLR424W	53
YLR425W	5
YLR426W	13
YLR427W	82
YLR428C	0
YLR429W	87
YLR430W	26
YLR432W	38
YLR433C	43
YLR434C	0
YLR435W	58
YLR436C	4
YLR437C	3
YLR438C-A	84
YLR438W	86
YLR439W	8
YLR440C	6
YLR441C	45
YLR442C	9

YLR443W	4
YLR446W	2
YLR447C	96
YLR448W	82
YLR449W	17
YLR450W	27
YLR451W	13
YLR452C	19
YLR453C	0
YLR454W	28
YLR455W	9
YLR456W	4
YLR457C	2
YLR459W	69
YLR460C	2
YLR461W	0
YLR462W	0
YLR465C	0
YLR466W	0
YML001W	94
YML006C	1
YML007W	13
YML008C	40
YML009C	14
YML010W	88
YML011C	3
YML012W	86
YML013W	7
YML014W	64
YML015C	19
YML016C	28
YML018C	62
YML019W	7
YML020W	16
YML021C	79
YML022W	82
YML023C	2
YML025C	37
YML027W	46
YML028W	87
YML029W	2
YML030W	18
YML031W	6
YML032C	29
YML034W	21

YML035C	87
YML036W	58
YML037C	1
YML038C	81
YML041C	8
YML042W	72
YML043C	4
YML046W	19
YML048W	5
YML049C	89
YML051W	12
YML052W	6
YML053C	0
YML054C	86
YML055W	18
YML056C	57
YML057W	48
YML058W	1
YML059C	71
YML060W	72
YML061C	55
YML062C	2
YML064C	30
YML065W	51
YML067C	43
YML068W	60
YML069W	94
YML070W	18
YML071C	13
YML072C	29
YML074C	46
YML075C	44
YML077W	78
YML078W	7
YML079W	13
YML081C-A	15
YML082W	2
YML085C	98
YML088W	6
YML089C	0
YML091C	4
YML092C	92
YML093W	84
YML094C-A	0
YML094W	89

YML095C	34
YML097C	59
YML098W	66
YML099C	10
YML100W	5
YML101C	0
YML102W	89
YML103C	17
YML104C	70
YML105C	15
YML106W	34
YML107C	2
YML108W	2
YML109W	8
YML110C	93
YML111W	0
YML112W	3
YML114C	9
YML115C	7
YML116W	3
YML117W	5
YML119W	2
YML120C	6
YML121W	81
YML123C	67
YML124C	0
YML125C	15
YML126C	86
YML127W	12
YML129C	2
YML130C	86
YML131W	69
YML133C	0
YMR001C	82
YMR002W	69
YMR003W	1
YMR004W	47
YMR005W	10
YMR008C	23
YMR009W	80
YMR010W	54
YMR011W	9
YMR012W	76
YMR013C	43
YMR014W	14

YMR017W	8
YMR018W	6
YMR019W	4
YMR020W	84
YMR021C	8
YMR022W	94
YMR023C	80
YMR024W	48
YMR025W	1
YMR026C	73
YMR028W	81
YMR029C	12
YMR030W	0
YMR031C	6
YMR031W-A	0
YMR032W	23
YMR033W	13
YMR035W	68
YMR036C	41
YMR038C	30
YMR039C	7
YMR041C	53
YMR042W	8
YMR043W	36
YMR044W	6
YMR045C	0
YMR047C	38
YMR048W	31
YMR049C	93
YMR052W	2
YMR053C	9
YMR054W	4
YMR055C	28
YMR056C	0
YMR057C	0
YMR058W	42
YMR059W	8
YMR061W	83
YMR063W	8
YMR064W	2
YMR065W	7
YMR066W	2
YMR067C	33
YMR068W	40
YMR069W	11

YMR071C	15
YMR072W	45
YMR074C	51
YMR075C-A	0
YMR075W	15
YMR076C	77
YMR077C	78
YMR078C	78
YMR079W	76
YMR080C	85
YMR083W	22
YMR086W	3
YMR087W	5
YMR088C	28
YMR089C	76
YMR090W	22
YMR091C	14
YMR092C	85
YMR093W	91
YMR094W	2
YMR095C	28
YMR096W	27
YMR097C	71
YMR100W	16
YMR101C	7
YMR102C	21
YMR103C	0
YMR104C	36
YMR105C	92
YMR106C	28
YMR108W	34
YMR109W	72
YMR110C	86
YMR111C	1
YMR112C	3
YMR114C	60
YMR116C	93
YMR117C	6
YMR118C	35
YMR119W	6
YMR120C	67
YMR123W	18
YMR124W	3
YMR125W	62
YMR127C	25

YMR128W	82
YMR129W	19
YMR131C	96
YMR132C	83
YMR133W	2
YMR134W	2
YMR135C	20
YMR137C	79
YMR138W	7
YMR139W	90
YMR140W	21
YMR141C	0
YMR142C	58
YMR144W	2
YMR145C	41
YMR146C	95
YMR147W	0
YMR149W	12
YMR150C	49
YMR151W	0
YMR152W	55
YMR153W	4
YMR154C	23
YMR155W	27
YMR156C	7
YMR157C	5
YMR158W	18
YMR159C	3
YMR160W	2
YMR162C	14
YMR163C	2
YMR165C	83
YMR167W	89
YMR168C	3
YMR171C	21
YMR172W	4
YMR173W	3
YMR176W	39
YMR177W	12
YMR178W	29
YMR179W	8
YMR180C	3
YMR181C	0
YMR182C	13
YMR183C	70

YMR184W	2
YMR186W	42
YMR187C	2
YMR188C	8
YMR189W	83
YMR190C	47
YMR192W	14
YMR193W	14
YMR195W	0
YMR196W	24
YMR197C	81
YMR198W	2
YMR199W	6
YMR200W	13
YMR201C	57
YMR202W	45
YMR203W	84
YMR204C	2
YMR205C	58
YMR206W	1
YMR207C	2
YMR209C	10
YMR210W	79
YMR211W	42
YMR212C	23
YMR213W	67
YMR214W	16
YMR215W	11
YMR216C	95
YMR218C	41
YMR219W	0
YMR221C	45
YMR222C	13
YMR223W	78
YMR224C	91
YMR225C	12
YMR226C	94
YMR227C	62
YMR228W	14
YMR229C	73
YMR230W	38
YMR231W	82
YMR232W	4
YMR233W	41
YMR234W	50

YMR235C	78
YMR236W	66
YMR237W	10
YMR238W	5
YMR239C	14
YMR240C	62
YMR241W	29
YMR243C	83
YMR244W	8
YMR246W	36
YMR247C	56
YMR250W	30
YMR251W	0
YMR253C	29
YMR255W	1
YMR257C	3
YMR258C	4
YMR260C	95
YMR261C	15
YMR263W	6
YMR264W	5
YMR265C	6
YMR266W	33
YMR267W	5
YMR268C	4
YMR269W	40
YMR270C	3
YMR271C	1
YMR273C	8
YMR274C	57
YMR275C	5
YMR276W	84
YMR277W	80
YMR278W	80
YMR279C	6
YMR280C	9
YMR282C	3
YMR284W	61
YMR285C	37
YMR287C	9
YMR288W	93
YMR289W	13
YMR290C	93
YMR291W	5
YMR292W	83

YMR293C	85
YMR294W	4
YMR295C	10
YMR296C	83
YMR297W	37
YMR298W	2
YMR299C	2
YMR300C	78
YMR302C	21
YMR303C	10
YMR304W	76
YMR305C	10
YMR306W	8
YMR307W	22
YMR308C	92
YMR309C	92
YMR310C	39
YMR311C	11
YMR312W	4
YMR313C	11
YMR314W	95
YMR315W	59
YMR316W	1
YMR317W	0
YMR318C	28
YMR319C	4
YMR322C	0
YMR323W	1
YNL001W	93
YNL002C	4
YNL003C	86
YNL004W	41
YNL005C	11
YNL006W	86
YNL007C	92
YNL008C	6
YNL009W	0
YNL011C	25
YNL012W	18
YNL013C	0
YNL014W	14
YNL015W	7
YNL016W	66
YNL018C	0
YNL019C	0

YNL020C 27
YNL021W 50
YNL023C 75
YNL024C 68
YNL025C 86
YNL027W 51
YNL028W 0
YNL029C 11
YNL030W 96
YNL032W 23
YNL034W 0
YNL035C 60
YNL036W 36
YNL037C 78
YNL038W 4
YNL039W 39
YNL040W 60
YNL041C 57
YNL042W 4
YNL044W 56
YNL045W 81
YNL046W 0
YNL047C 5
YNL048W 87
YNL049C 7
YNL050C 4
YNL051W 12
YNL052W 12
YNL053W 41
YNL054W 12
YNL055C 80
YNL056W 7
YNL058C 5
YNL061W 95
YNL062C 84
YNL063W 40
YNL064C 98
YNL065W 9
YNL066W 5
YNL068C 40
YNL070W 13
YNL071W 89
YNL072W 88
YNL073W 5
YNL074C 0

YNL075W93
YNL076W2
YNL077W2
YNL078W2
YNL079C 5
YNL081C 26
YNL082W91
YNL084C 17
YNL085W23
YNL086W3
YNL087W43
YNL088W96
YNL090W13
YNL091W10
YNL092W84
YNL093W2
YNL094W7
YNL095C 4
YNL096C 31
YNL097C 56
YNL098C 31
YNL099C 15
YNL100W2
YNL101W61
YNL102W91
YNL103W4
YNL104C 18
YNL105W0
YNL106C 62
YNL107W84
YNL109W0
YNL110C 70
YNL112W97
YNL113W73
YNL116W20
YNL118C 40
YNL119W40
YNL121C 64
YNL122C 5
YNL124W55
YNL125C 41
YNL126W53
YNL127W48
YNL128W30
YNL129W53

YNL130C	50
YNL131W	18
YNL132W	94
YNL133C	2
YNL134C	21
YNL135C	75
YNL136W	43
YNL137C	21
YNL138W	79
YNL138W-A	13
YNL139C	81
YNL140C	0
YNL141W	79
YNL142W	72
YNL144C	2
YNL145W	1
YNL146W	0
YNL147W	78
YNL148C	74
YNL151C	10
YNL152W	16
YNL153C	78
YNL154C	57
YNL155W	47
YNL157W	1
YNL158W	2
YNL159C	2
YNL160W	1
YNL161W	89
YNL163C	70
YNL164C	4
YNL165W	2
YNL166C	3
YNL167C	35
YNL169C	87
YNL171C	0
YNL172W	53
YNL173C	4
YNL175C	24
YNL176C	2
YNL178W	97
YNL180C	2
YNL181W	40
YNL182C	53
YNL183C	15

YNL185C 83
YNL186W 21
YNL187W 10
YNL188W 0
YNL189W 97
YNL190W 4
YNL191W 18
YNL192W 20
YNL193W 8
YNL194C 2
YNL196C 0
YNL197C 12
YNL199C 2
YNL201C 74
YNL202W 93
YNL204C 4
YNL205C 0
YNL206C 12
YNL207W 97
YNL208W 2
YNL210W 1
YNL211C 4
YNL212W 30
YNL213C 5
YNL214W 2
YNL215W 13
YNL216W 5
YNL217W 26
YNL218W 58
YNL219C 81
YNL220W 92
YNL221C 62
YNL222W 82
YNL223W 71
YNL224C 27
YNL225C 2
YNL226W 0
YNL227C 89
YNL228W 0
YNL229C 53
YNL230C 11
YNL231C 25
YNL232W 43
YNL233W 22
YNL234W 3

YNL235C	0
YNL236W	7
YNL237W	15
YNL238W	77
YNL239W	56
YNL241C	92
YNL242W	24
YNL243W	66
YNL244C	90
YNL245C	6
YNL246W	20
YNL248C	49
YNL250W	85
YNL251C	38
YNL252C	53
YNL253W	45
YNL255C	80
YNL257C	16
YNL258C	5
YNL259C	59
YNL260C	20
YNL261W	57
YNL262W	91
YNL263C	85
YNL264C	13
YNL265C	74
YNL266W	0
YNL267W	76
YNL271C	74
YNL272C	18
YNL273W	51
YNL275W	74
YNL276C	0
YNL277W	29
YNL277W-A	0
YNL278W	4
YNL279W	18
YNL280C	81
YNL281W	4
YNL282W	4
YNL284C	77
YNL284C-B	1
YNL285W	0
YNL286W	63
YNL287W	95

YNL288W	91
YNL289W	19
YNL290W	90
YNL291C	16
YNL292W	46
YNL293W	49
YNL295W	2
YNL298W	32
YNL300W	1
YNL301C	95
YNL304W	2
YNL306W	10
YNL307C	4
YNL308C	66
YNL309W	2
YNL311C	3
YNL312W	75
YNL313C	83
YNL314W	2
YNL315C	56
YNL316C	28
YNL317W	64
YNL318C	7
YNL319W	0
YNL320W	95
YNL321W	31
YNL323W	7
YNL324W	0
YNL326C	82
YNL327W	2
YNL328C	40
YNL329C	63
YNL330C	97
YNL331C	27
YNL333W	16
YNL334C	4
YNL336W	0
YNL339C	0
YNR001C	86
YNR002C	8
YNR003C	77
YNR004W	0
YNR005C	0
YNR006W	58
YNR007C	94

YNR008W	70
YNR009W	1
YNR010W	2
YNR011C	6
YNR012W	78
YNR013C	47
YNR015W	81
YNR016C	83
YNR017W	65
YNR018W	17
YNR019W	57
YNR022C	2
YNR023W	52
YNR024W	1
YNR025C	0
YNR026C	11
YNR027W	25
YNR028W	0
YNR029C	67
YNR030W	73
YNR031C	40
YNR032W	19
YNR033W	32
YNR034W	20
YNR035C	81
YNR036C	75
YNR037C	26
YNR039C	12
YNR040W	4
YNR041C	86
YNR042W	0
YNR043W	82
YNR045W	2
YNR046W	87
YNR047W	44
YNR048W	11
YNR049C	2
YNR050C	26
YNR051C	38
YNR052C	88
YNR053C	94
YNR054C	84
YNR055C	15
YNR056C	1
YNR058W	14

YNR059W	5
YNR060W	9
YNR061C	1
YNR063W	1
YNR064C	59
YNR065C	18
YNR066C	1
YNR067C	17
YNR068C	0
YNR069C	0
YNR070W	6
YNR071C	30
YNR072W	0
YNR074C	51
YNR075W	0
YOL001W	4
YOL002C	76
YOL003C	45
YOL004W	81
YOL005C	90
YOL006C	92
YOL008W	69
YOL009C	17
YOL010W	95
YOL011W	12
YOL012C	86
YOL013C	61
YOL014W	0
YOL015W	0
YOL016C	41
YOL017W	0
YOL018C	83
YOL019W	12
YOL020W	5
YOL021C	98
YOL022C	84
YOL024W	0
YOL028C	0
YOL030W	15
YOL031C	25
YOL032W	46
YOL033W	83
YOL034W	82
YOL035C	0
YOL036W	2

YOL038W	97
YOL039W	11
YOL041C	78
YOL042W	22
YOL043C	53
YOL044W	2
YOL045W	20
YOL046C	0
YOL050C	0
YOL051W	6
YOL052C	83
YOL054W	44
YOL055C	16
YOL056W	2
YOL058W	71
YOL059W	13
YOL061W	18
YOL062C	18
YOL063C	5
YOL064C	34
YOL065C	41
YOL066C	51
YOL067C	2
YOL068C	34
YOL069W	50
YOL070C	4
YOL072W	9
YOL073C	15
YOL075C	50
YOL077C	97
YOL078W	16
YOL080C	78
YOL081W	28
YOL082W	0
YOL083W	0
YOL085C	0
YOL086C	5
YOL087C	71
YOL088C	6
YOL089C	7
YOL090W	89
YOL091W	0
YOL093W	96
YOL094C	97
YOL095C	14

YOL097C	93
YOL098C	49
YOL099C	0
YOL100W	28
YOL101C	0
YOL102C	63
YOL103W	50
YOL103W-B	2
YOL104C	2
YOL105C	3
YOL106W	0
YOL107W	71
YOL108C	6
YOL109W	0
YOL110W	5
YOL111C	13
YOL112W	61
YOL113W	6
YOL115W	64
YOL116W	3
YOL117W	2
YOL118C	0
YOL122C	41
YOL123W	86
YOL124C	93
YOL126C	4
YOL127W	87
YOL128C	0
YOL129W	29
YOL130W	9
YOL131W	0
YOL132W	6
YOL133W	95
YOL135C	65
YOL136C	3
YOL137W	19
YOL138C	53
YOL139C	98
YOL140W	37
YOL142W	85
YOL143C	31
YOL144W	12
YOL145C	85
YOL146W	60
YOL147C	53

YOL148C	10
YOL149W	6
YOL150C	0
YOL151W	14
YOL152W	20
YOL154W	10
YOL156W	0
YOL159C	0
YOR001W	75
YOR003W	12
YOR004W	80
YOR005C	67
YOR006C	89
YOR007C	77
YOR008C	6
YOR009W	0
YOR010C	0
YOR011W	3
YOR013W	0
YOR014W	91
YOR016C	11
YOR017W	9
YOR018W	11
YOR020C	88
YOR023C	4
YOR025W	23
YOR026W	4
YOR027W	95
YOR028C	0
YOR029W	0
YOR031W	0
YOR032C	13
YOR033C	48
YOR034C	17
YOR035C	32
YOR036W	78
YOR037W	8
YOR038C	22
YOR039W	90
YOR040W	9
YOR042W	11
YOR043W	13
YOR045W	4
YOR046C	83
YOR047C	1

YOR048C 76
YOR049C 16
YOR050C 0
YOR051C 6
YOR053W 0
YOR054C 7
YOR056C 83
YOR057W 82
YOR058C 21
YOR059C 19
YOR060C 2
YOR061W 30
YOR062C 0
YOR063W 94
YOR064C 24
YOR065W 86
YOR066W 2
YOR067C 78
YOR068C 0
YOR069W 79
YOR070C 83
YOR071C 2
YOR072W 0
YOR073W 11
YOR074C 88
YOR075W 22
YOR076C 1
YOR078W 2
YOR079C 57
YOR080W 12
YOR081C 9
YOR082C 0
YOR083W 1
YOR084W 10
YOR085W 74
YOR086C 22
YOR087W 22
YOR089C 91
YOR090C 81
YOR092W 8
YOR093C 67
YOR095C 87
YOR096W 74
YOR097C 1
YOR098C 3

YOR099W	17
YOR100C 73	
YOR101W	66
YOR102W	0
YOR103C 83	
YOR104W	6
YOR106W	11
YOR107W	8
YOR108W	1
YOR109W	16
YOR110W	7
YOR111W	70
YOR112W	89
YOR113W	59
YOR114W	2
YOR115C 28	
YOR116C 90	
YOR117W	94
YOR118W	16
YOR119C 94	
YOR120W	61
YOR121C 0	
YOR122C 78	
YOR123C 73	
YOR124C 15	
YOR125C 71	
YOR127W	17
YOR128C 38	
YOR129C 11	
YOR130C 40	
YOR131C 25	
YOR132W	16
YOR133W	98
YOR134W	23
YOR136W	81
YOR138C 5	
YOR140W	18
YOR141C 64	
YOR142W	93
YOR142W-A	0
YOR143C 71	
YOR144C 10	
YOR145C 94	
YOR146W	0
YOR147W	3

YOR148C	6
YOR150W	84
YOR151C	90
YOR153W	23
YOR154W	43
YOR155C	22
YOR156C	34
YOR157C	96
YOR158W	6
YOR159C	79
YOR160W	77
YOR161C	72
YOR162C	2
YOR164C	64
YOR165W	39
YOR167C	75
YOR171C	31
YOR172W	3
YOR173W	41
YOR174W	7
YOR175C	87
YOR176W	81
YOR177C	1
YOR178C	12
YOR179C	1
YOR180C	22
YOR181W	62
YOR184W	83
YOR185C	36
YOR187W	93
YOR188W	11
YOR189W	0
YOR190W	7
YOR191W	14
YOR192C	1
YOR194C	65
YOR195W	6
YOR196C	86
YOR197W	29
YOR198C	23
YOR201C	47
YOR202W	30
YOR204W	77
YOR205C	16
YOR206W	87

YOR207C	97
YOR208W	24
YOR209C	67
YOR210W	79
YOR211C	22
YOR212W	86
YOR213C	1
YOR214C	0
YOR215C	10
YOR217W	93
YOR219C	9
YOR220W	3
YOR221C	6
YOR222W	59
YOR224C	97
YOR225W	0
YOR226C	45
YOR227W	5
YOR229W	2
YOR230W	1
YOR231W	51
YOR232W	96
YOR233W	18
YOR236W	69
YOR239W	19
YOR241W	81
YOR242C	4
YOR243C	95
YOR244W	96
YOR245C	89
YOR246C	93
YOR247W	0
YOR249C	9
YOR250C	86
YOR251C	66
YOR252W	35
YOR254C	87
YOR257W	86
YOR258W	38
YOR259C	96
YOR260W	46
YOR261C	98
YOR262W	89
YOR263C	0
YOR264W	2

YOR265W	58
YOR266W	2
YOR267C 18	
YOR269W	49
YOR270C 95	
YOR271C 80	
YOR272W	90
YOR273C 9	
YOR274W	83
YOR275C 82	
YOR276W	4
YOR279C 2	
YOR281C 80	
YOR282W	0
YOR283W	29
YOR284W	0
YOR285W	36
YOR286W	48
YOR288C 18	
YOR289W	84
YOR290C 60	
YOR292C 19	
YOR293W	81
YOR294W	63
YOR295W	17
YOR297C 2	
YOR298C-A	91
YOR298W	4
YOR299W	11
YOR300W	0
YOR302W	0
YOR303W	33
YOR304W	81
YOR305W	8
YOR307C 59	
YOR308C 68	
YOR309C 0	
YOR310C 92	
YOR311C 22	
YOR312C 96	
YOR313C 4	
YOR314W	0
YOR315W	1
YOR316C 12	
YOR317W	55

YOR318C	0
YOR319W	58
YOR320C	7
YOR321W	9
YOR322C	11
YOR323C	86
YOR324C	0
YOR325W	0
YOR326W	79
YOR327C	60
YOR329C	4
YOR331C	0
YOR332W	94
YOR333C	0
YOR334W	21
YOR335C	97
YOR336W	73
YOR338W	5
YOR339C	65
YOR340C	28
YOR341W	87
YOR344C	5
YOR345C	0
YOR346W	76
YOR348C	15
YOR349W	50
YOR350C	0
YOR351C	11
YOR352W	10
YOR353C	22
YOR354C	2
YOR355W	4
YOR356W	86
YOR358W	29
YOR359W	18
YOR360C	35
YOR361C	94
YOR362C	96
YOR363C	0
YOR364W	0
YOR366W	0
YOR367W	63
YOR368W	13
YOR370C	35
YOR371C	2

YOR372C	2
YOR373W	9
YOR374W	62
YOR375C	39
YOR377W	0
YOR378W	17
YOR379C	0
YOR380W	4
YOR381W	13
YOR382W	1
YOR383C	0
YOR384W	25
YOR385W	1
YOR386W	68
YOR387C	1
YOR388C	20
YOR389W	7
YOR390W	23
YOR392W	0
YOR393W	1
YPL001W	81
YPL002C	87
YPL003W	41
YPL004C	7
YPL005W	2
YPL007C	3
YPL008W	93
YPL009C	82
YPL010W	88
YPL011C	5
YPL012W	83
YPL013C	76
YPL014W	2
YPL016W	16
YPL018W	1
YPL019C	4
YPL020C	47
YPL022W	88
YPL023C	15
YPL024W	5
YPL025C	0
YPL026C	12
YPL027W	0
YPL028W	92
YPL029W	87

YPL031C	35
YPL032C	13
YPL034W	3
YPL036W	14
YPL037C	89
YPL038W	12
YPL040C	84
YPL042C	76
YPL043W	89
YPL045W	84
YPL046C	80
YPL047W	7
YPL048W	30
YPL049C	1
YPL050C	17
YPL051W	74
YPL052W	1
YPL053C	0
YPL055C	2
YPL056C	0
YPL057C	13
YPL059W	89
YPL060W	41
YPL061W	2
YPL063W	63
YPL064C	30
YPL065W	84
YPL066W	4
YPL067C	12
YPL068C	2
YPL070W	9
YPL071C	2
YPL073C	0
YPL074W	63
YPL075W	2
YPL076W	58
YPL077C	2
YPL078C	52
YPL082C	77
YPL083C	39
YPL084W	29
YPL085W	43
YPL086C	92
YPL087W	59
YPL088W	73

YPL089C	43
YPL090C	96
YPL091W	97
YPL092W	16
YPL093W	95
YPL094C	40
YPL095C	17
YPL096W	35
YPL097W	83
YPL098C	56
YPL100W	30
YPL101W	55
YPL102C	0
YPL104W	76
YPL105C	34
YPL106C	60
YPL107W	8
YPL108W	3
YPL109C	59
YPL110C	16
YPL111W	83
YPL112C	4
YPL113C	9
YPL114W	0
YPL115C	37
YPL116W	20
YPL118W	7
YPL119C	25
YPL120W	76
YPL121C	6
YPL122C	89
YPL124W	0
YPL125W	71
YPL126W	29
YPL127C	56
YPL128C	9
YPL129W	17
YPL130W	1
YPL131W	89
YPL132W	74
YPL133C	16
YPL134C	12
YPL135C-A	0
YPL135W	54
YPL136W	0

YPL137C	7
YPL138C	9
YPL139C	1
YPL140C	19
YPL141C	24
YPL144W	1
YPL145C	20
YPL146C	56
YPL147W	84
YPL149W	58
YPL150W	30
YPL151C	95
YPL152W	63
YPL153C	36
YPL154C	93
YPL155C	34
YPL156C	2
YPL157W	57
YPL158C	2
YPL159C	2
YPL160W	92
YPL161C	2
YPL162C	56
YPL163C	0
YPL164C	39
YPL166W	2
YPL169C	71
YPL170W	76
YPL171C	26
YPL172C	84
YPL174C	46
YPL175W	92
YPL177C	16
YPL178W	89
YPL179W	3
YPL180W	7
YPL181W	22
YPL183W-A	24
YPL187W	3
YPL188W	25
YPL189W	11
YPL190C	29
YPL191C	55
YPL192C	0
YPL193W	2

YPL194W	4
YPL195W	84
YPL196W	57
YPL200W	0
YPL201C	1
YPL202C	1
YPL203W	77
YPL204W	97
YPL205C	0
YPL207W	60
YPL208W	12
YPL209C	94
YPL210C	68
YPL211W	94
YPL212C	76
YPL213W	85
YPL214C	25
YPL215W	16
YPL216W	6
YPL217C	90
YPL218W	96
YPL219W	2
YPL220W	93
YPL221W	5
YPL222W	59
YPL224C	36
YPL226W	21
YPL227C	85
YPL228W	13
YPL229W	2
YPL231W	17
YPL232W	32
YPL233W	7
YPL234C	31
YPL235W	94
YPL236C	69
YPL237W	95
YPL238C	0
YPL240C	62
YPL241C	1
YPL242C	56
YPL243W	58
YPL244C	97
YPL245W	3
YPL246C	28

YPL247C	87
YPL248C	9
YPL249C	71
YPL249C-A	87
YPL250C	0
YPL251W	0
YPL252C	91
YPL253C	1
YPL254W	14
YPL255W	2
YPL256C	5
YPL257W	0
YPL257W-B	0
YPL258C	10
YPL259C	97
YPL260W	18
YPL262W	86
YPL263C	82
YPL264C	40
YPL265W	30
YPL266W	98
YPL267W	1
YPL269W	4
YPL270W	19
YPL271W	19
YPL272C	1
YPL274W	1
YPL277C	2
YPL280W	0
YPR001W	2
YPR003C	44
YPR004C	82
YPR005C	0
YPR006C	15
YPR007C	8
YPR008W	12
YPR010C	94
YPR010C-A	4
YPR011C	39
YPR014C	0
YPR015C	36
YPR016C	91
YPR017C	5
YPR018W	16
YPR019W	92

YPR020W	16
YPR021C	25
YPR023C	81
YPR025C	77
YPR028W	88
YPR029C	93
YPR030W	2
YPR031W	42
YPR032W	30
YPR033C	95
YPR034W	3
YPR035W	86
YPR036W	74
YPR037C	19
YPR040W	81
YPR041W	95
YPR042C	7
YPR045C	56
YPR046W	1
YPR047W	86
YPR048W	84
YPR049C	15
YPR051W	73
YPR052C	47
YPR054W	3
YPR055W	48
YPR056W	77
YPR057W	2
YPR058W	39
YPR062W	31
YPR063C	8
YPR065W	50
YPR066W	87
YPR067W	83
YPR068C	4
YPR069C	82
YPR070W	2
YPR071W	0
YPR072W	31
YPR073C	80
YPR074C	85
YPR075C	5
YPR076W	0
YPR077C	0
YPR078C	0

YPR079W	10
YPR081C	4
YPR082C	92
YPR083W	3
YPR084W	3
YPR085C	9
YPR086W	89
YPR088C	95
YPR091C	21
YPR093C	6
YPR094W	92
YPR095C	16
YPR096C	0
YPR097W	18
YPR099C	0
YPR100W	68
YPR101W	1
YPR102C	94
YPR103W	95
YPR104C	31
YPR105C	75
YPR106W	1
YPR107C	47
YPR108W	97
YPR110C	92
YPR111W	15
YPR112C	92
YPR113W	94
YPR114W	7
YPR115W	1
YPR118W	79
YPR119W	55
YPR120C	10
YPR121W	0
YPR122W	5
YPR123C	0
YPR124W	4
YPR125W	30
YPR126C	0
YPR128C	28
YPR129W	74
YPR130C	0
YPR132W	91
YPR133C	67
YPR134W	1

YPR135W	55
YPR136C	0
YPR137C-A	0
YPR137C-B	0
YPR137W	85
YPR138C	19
YPR141C	89
YPR143W	19
YPR144C	68
YPR145W	35
YPR148C	2
YPR149W	12
YPR152C	7
YPR154W	8
YPR155C	16
YPR156C	11
YPR157W	17
YPR159W	17
YPR160C-A	0
YPR160W	86
YPR161C	24
YPR162C	57
YPR163C	34
YPR164W	1
YPR165W	70
YPR167C	27
YPR168W	66
YPR169W	15
YPR171W	2
YPR172W	9
YPR173C	96
YPR174C	0
YPR175W	87
YPR176C	92
YPR177C	0
YPR178W	90
YPR179C	6
YPR180W	80
YPR181C	94
YPR182W	89
YPR183W	88
YPR184W	71
YPR185W	15
YPR187W	85
YPR188C	3

YPR189W	71
YPR190C	35
YPR191W	20
YPR193C	3
YPR194C	8
YPR198W	9
YPR201W	19
YPR203W	0
