# Supplementary material for: A disease-related essential protein prediction model based on the transfer neural network
Source: Front Genet. 2023 Jan 4;13:1087294. doi: 10.3389/fgene.2022.1087294 (PMC9845409; doi:10.3389/fgene.2022.1087294)
Supplement: Supplementary file 3 [file DataSheet3.PDF]

YKL144C	YPR110C
YKL144C	YOR210W
YKL144C	YPR190C
YKL144C	YOR224C
YKL144C	YDR045C
YKL144C	YNL151C
YKL144C	YNL113W
YKL144C	YKR025W
YKL144C	YOR116C
YKL144C	YPR187W
YKL144C	YBR154C
YKL144C	YDR005C
YKL144C	YJL011C
YKL144C	YNR003C
YKL144C	YDL150W
YKL144C	YOR207C
YPR110C	YBR245C
YPR110C	YOR210W
YPR110C	YPR190C
YPR110C	YOR224C
YPR110C	YNL151C
YPR110C	YBR049C
YPR110C	YPR010C
YPR110C	YKR025W
YPR110C	YOR116C
YPR110C	YGL156W
YPR110C	YPR032W
YPR110C	YPR187W
YPR110C	YBR154C
YPR110C	YJR063W
YPR110C	YOR340C
YPR110C	YDR005C
YPR110C	YNL248C
YPR110C	YNR003C
YPR110C	YOR341W
YPR110C	YDL150W
YPR110C	YOR207C
YML016C	YPR184W
YML016C	YKL193C
YML016C	YKL088W
YML016C	YJL039C
YML016C	YJR077C
YML016C	YBL075C
YPR184W	YJL039C
YPR184W	YJR077C

YPR184W	YBR072W
YDR240C	YGR013W
YDR240C	YLR275W
YDR240C	YER029C
YDR240C	YGR074W
YDR240C	YMR125W
YDR240C	YDL087C
YDR240C	YPL178W
YDR240C	YHR165C
YDR240C	YPR182W
YDR240C	YBR119W
YDR240C	YML046W
YDR240C	YHR086W
YDR240C	YDR235W
YDR240C	YLR147C
YDR240C	YLR298C
YDR240C	YKL012W
YDR240C	YLR345W
YDR240C	YIL061C
YDR240C	YIL129C
YGR013W	YLR275W
YGR013W	YER172C
YGR013W	YER029C
YGR013W	YGR074W
YGR013W	YMR125W
YGR013W	YDL087C
YGR013W	YPL178W
YGR013W	YPR182W
YGR013W	YBR119W
YGR013W	YML046W
YGR013W	YHR086W
YGR013W	YDR235W
YGR013W	YLR147C
YGR013W	YLR298C
YGR013W	YKL012W
YGR013W	YIL061C
YDL083C	YOR234C
YDL083C	YBR086C
YDL083C	YBR181C
YDL083C	YGL120C
YDL083C	YNL224C
YDL083C	YMR230W
YDL083C	YGR034W
YDL083C	YLR221C
YDL083C	YGR281W

YOR234C YBR142W
YOR234C YML026C
YOR234C YMR143W
YOR234C YGR034W
YOR234C YLR221C
YOR234C YGR281W
YOR234C YPR043W
YOR234C YDL195W
YOR234C YDL075W
YOR234C YOR173W
YOR234C YDR382W
YOR234C YDR460W
YOR234C YPR025C
YBR189W YOL139C
YBR189W YDL060W
YBR189W YHR196W
YBR189W YOR310C
YBR189W YNL132W
YBR189W YGR214W
YBR189W YGR081C
YBR189W YBR247C
YBR189W YOR056C
YBR189W YPR189W
YBR189W YPL204W
YOL139C YML056C
YOL139C YDL051W
YOL139C YGL173C
YOL139C YLR367W
YOL139C YER029C
YOL139C YMR125W
YOL139C YDL087C
YOL139C YGR162W
YOL139C YHL034C
YOL139C YOR276W
YOL139C YCL011C
YOL139C YGL049C
YOL139C YER102W
YOL139C YNL262W
YOL139C YKL204W
YML009C YNL284C
YML009C YBL038W
YML009C YBR282W
YML009C YDR116C
YML009C YKR006C
YML009C YKL167C

YML009C YMR098C
YML009C YKR085C
YML009C YNL252C
YML009C YLR189C
YML009C YCR046C
YML009C YLR312W-A
YML009C YNL005C
YML009C YJL063C
YML009C YCL014W
YML009C YDR462W
YML009C YCR071C
YML009C YMR225C
YML009C YOR201C
YML009C YFR051C
YML009C YDR237W
YML009C YDR322W
YML009C YLR371W
YML009C YGR220C
YML009C YMR193W
YML009C YDR296W
YML009C YLR439W
YML009C YOR150W
YML009C YHR147C
YML009C YPL183W-A
YML009C YCR030C
YML009C YBR122C
YML009C YDR405W
YML009C YPL204W
YNL284C YBL038W
YNL284C YBR282W
YNL284C YKR085C
YNL284C YLR189C
YNL284C YDR164C
YNL284C YJL005W
YNL284C YPR100W
YNL284C YNL005C
YNL284C YCL014W
YNL284C YDR462W
YNL284C YCR071C
YNL284C YMR225C
YNL284C YOR201C
YNL284C YDR237W
YNL284C YDR322W
YNL284C YBL004W
YNL284C YLR264W

YNL284C	YMR024W
YNL284C	YLR439W
YNL284C	YOR150W
YNL284C	YHR147C
YNL284C	YBR122C
YNL284C	YBL024W
YHR088W	YMR290C
YHR088W	YOR272W
YHR088W	YLL045C
YHR088W	YHR052W
YHR088W	YOL077C
YHR088W	YER006W
YHR088W	YOR206W
YHR088W	YFL002C
YHR088W	YFR001W
YHR088W	YDR496C
YHR088W	YNL061W
YHR088W	YGL111W
YHR088W	YPR016C
YHR088W	YNL110C
YHR088W	YPL131W
YMR290C	YBR142W
YMR290C	YOR272W
YMR290C	YHR052W
YMR290C	YOL041C
YMR290C	YOL077C
YMR290C	YER126C
YMR290C	YKR081C
YMR290C	YPL043W
YMR290C	YOR206W
YMR290C	YFL002C
YMR290C	YFR001W
YMR290C	YGR103W
YMR290C	YMR229C
YMR290C	YMR049C
YMR290C	YPL211W
YMR290C	YDR060W
YMR290C	YDR496C
YMR290C	YNL061W
YMR290C	YGL111W
YMR290C	YPR016C
YMR290C	YPL093W
YMR290C	YNL110C
YBR142W	YPL249C-A
YBR142W	YOR272W

YBR142W	YOL041C
YBR142W	YML073C
YBR142W	YLR367W
YBR142W	YPL043W
YBR142W	YNL002C
YBR142W	YFR001W
YBR142W	YMR049C
YBR142W	YDR060W
YBR142W	YDR496C
YBR142W	YNL061W
YBR142W	YLR221C
YBR142W	YOL127W
YBR142W	YGL111W
YBR142W	YPL093W
YBR142W	YGR281W
YBR142W	YKL014C
YPL249C-A	YHR052W
YPL249C-A	YFR031C-A
YPL249C-A	YNL301C
YPL249C-A	YDR060W
YPL249C-A	YNL061W
YPL249C-A	YDR101C
YPL249C-A	YGR245C
YER107C	YMR047C
YER107C	YJL041W
YER107C	YJL061W
YER107C	YIL115C
YMR047C	YJL041W
YMR047C	YJL061W
YMR047C	YIL115C
YFR037C	YHR056C
YFR037C	YBR245C
YFR037C	YDR174W
YFR037C	YLR321C
YFR037C	YML127W
YFR037C	YPR034W
YFR037C	YMR072W
YFR037C	YOR116C
YFR037C	YMR091C
YFR037C	YMR033W
YFR037C	YDR303C
YFR037C	YKR008W
YFR037C	YGR056W
YFR037C	YFR013W
YFR037C	YNL030W

YFR037C YLR357W
YFR037C YCR052W
YFR037C YIL126W
YFR037C YLR033W
YFR037C YNR003C
YFR037C YOR207C
YFR037C YOL090W
YFR037C YGR275W
YHR056C YLR321C
YHR056C YML127W
YHR056C YMR091C
YHR056C YDR303C
YHR056C YGR056W
YHR056C YLR033W
YBR245C YKR001C
YBR245C YBL003C
YBR245C YDR224C
YBR245C YPR034W
YBR245C YMR072W
YBR245C YMR091C
YBR245C YBR009C
YBR245C YDR303C
YBR245C YKR008W
YBR245C YFR013W
YBR245C YGL133W
YBR245C YPL082C
YBR245C YLR357W
YBR245C YCR052W
YBR245C YDR190C
YBR245C YLR033W
YBR245C YOL004W
YBL007C YHR114W
YBL007C YLR337C
YBL007C YOR181W
YBL007C YJL201W
YBL007C YFR024C-A
YBL007C YML001W
YBL007C YDL191W
YBL007C YBL027W
YHR114W YOR181W
YHR114W YJL201W
YHR114W YLR150W
YHR114W YFR024C-A
YHR114W YCR030C
YHR114W YDL191W

YHR114W	YBL027W
YDL060W	YNL207W
YDL060W	YDR064W
YDL060W	YBR048W
YDL060W	YGR081C
YDL060W	YOR145C
YDL060W	YPL012W
YDL060W	YDL148C
YDL060W	YJR145C
YDL060W	YJL190C
YDL060W	YPL266W
YDL060W	YOR096W
YDL060W	YBR247C
YDL060W	YKL143W
YDL060W	YDR447C
YDL060W	YOR056C
YDL060W	YGR054W
YDL060W	YPL204W
YDL060W	YIL069C
YDL060W	YML024W
YNL207W	YDR283C
YNL207W	YKR057W
YNL207W	YML026C
YNL207W	YDR064W
YNL207W	YLR367W
YNL207W	YDR025W
YNL207W	YOR145C
YNL207W	YPL012W
YNL207W	YPL266W
YNL207W	YLR264W
YNL207W	YOR290C
YNL207W	YBR247C
YNL207W	YKL143W
YNL207W	YML091C
YNL207W	YOR056C
YNL207W	YGR054W
YNL207W	YPL204W
YNL207W	YER102W
YNL207W	YPL081W
YDR264C	YMR197C
YDR264C	YKL196C
YDR264C	YOR036W
YDR264C	YOR212W
YMR197C	YKL196C
YMR197C	YBL050W

YMR197C	YOR036W
YMR197C	YOR212W
YMR197C	YGL095C
YHR117W	YOR375C
YHR117W	YKL006W
YOR375C	YKL006W
YOR375C	YGL130W
YOR375C	YOR370C
YOR375C	YKR014C
YOR375C	YPL228W
YER094C	YGL011C
YER094C	YGR135W
YER094C	YOR362C
YER094C	YPR103W
YER094C	YGR232W
YER094C	YML092C
YER094C	BLM3
YER094C	YOL038W
YER094C	YFR050C
YER094C	YMR314W
YER094C	YBL041W
YER094C	YER012W
YER094C	YJL001W
YGL011C	YHR200W
YGL011C	YGR135W
YGL011C	YOR362C
YGL011C	YPR103W
YGL011C	YIL075C
YGL011C	YDL147W
YGL011C	YML092C
YGL011C	BLM3
YGL011C	YOL038W
YGL011C	YFR050C
YGL011C	YMR314W
YGL011C	YBL041W
YGL011C	YER012W
YGL011C	YJL001W
YDL097C	YOR259C
YDL097C	YHR027C
YDL097C	YHR200W
YDL097C	YOR261C
YDL097C	YDR427W
YDL097C	YIL075C
YDL097C	YER021W
YDL097C	YHL030W

YDL097C	YLR421C
YDL097C	YFR004W
YDL097C	YFR052W
YDL097C	YPR108W
YDL097C	YDL147W
YDL097C	YFR010W
YDL097C	YOL038W
YDL097C	YKL145W
YDL097C	YDL007W
YDL097C	YGL048C
YDL097C	YDR394W
YDL097C	YOR117W
YOR259C	YHR027C
YOR259C	YHR200W
YOR259C	YML124C
YOR259C	YDR427W
YOR259C	YHL030W
YOR259C	YLR421C
YOR259C	YFR052W
YOR259C	YDL147W
YOR259C	YFR010W
YOR259C	YKL145W
YLR002C	YOR272W
YLR002C	YHR052W
YLR002C	YOL077C
YLR002C	YER006W
YLR002C	YKR081C
YLR002C	YOR206W
YLR002C	YFL002C
YLR002C	YFR001W
YLR002C	YGR103W
YLR002C	YLR449W
YLR002C	YPR016C
YOR272W	YHR052W
YOR272W	YOL077C
YOR272W	YER006W
YOR272W	YKR081C
YOR272W	YPL043W
YOR272W	YNL002C
YOR272W	YOR206W
YOR272W	YFL002C
YOR272W	YFR001W
YOR272W	YGR103W
YOR272W	YMR049C
YOR272W	YLL034C

YOR272W	YPL211W
YOR272W	YDR060W
YOR272W	YDR496C
YOR272W	YNL061W
YOR272W	YKL009W
YOR272W	YDR087C
YOR272W	YGL111W
YOR272W	YPR016C
YOR272W	YNL110C
YOR272W	YLL008W
YOR272W	YDL082W
YOR272W	YDL031W
YOR272W	YKL021C
YGR095C	YGR195W
YGR095C	YGR090W
YGR095C	YGR158C
YGR095C	YCR035C
YGR095C	YHR069C
YGR095C	YNL189W
YGR095C	YOL021C
YGR095C	YOR076C
YGR095C	YHR081W
YGR095C	YNL232W
YGR095C	YDL111C
YGR095C	YDR280W
YGR095C	YOR001W
YGR095C	YOL142W
YGR195W	YMR128W
YGR195W	YGR158C
YGR195W	YML056C
YGR195W	YCR035C
YGR195W	YHR069C
YGR195W	YNL189W
YGR195W	YJL109C
YGR195W	YOL021C
YGR195W	YOR076C
YGR195W	YHR081W
YGR195W	YNL232W
YGR195W	YDL111C
YGR195W	YDR280W
YGR195W	YOR001W
YGR195W	YOL142W
YGR195W	YKL126W
YDL003W	YJL074C
YDL003W	YFL008W

YDL003W	YER147C
YDL003W	YBL027W
YJL074C	YJL008C
YJL074C	YFL008W
YJL074C	YER147C
YJL074C	YDR158W
YGR167W	YNL084C
YGR167W	YOR181W
YGR167W	YGL206C
YGR167W	YFR024C-A
YNL084C	YNL113W
YNL084C	YOR181W
YNL084C	YFR024C-A
YNL084C	YJL011C
YNL084C	YNL243W
YNL084C	YDL150W
YNL084C	YIR006C
YGL099W	YLL045C
YGL099W	YBR084W
YGL099W	YLR074C
YGL099W	YBR267W
YGL099W	YDR101C
YGL099W	YGR245C
YGL099W	YPR016C
YGL099W	YEL054C
YGL099W	YHR170W
YGL099W	YHR010W
YGL099W	YOR063W
YGL099W	YMR194W
YLL045C	YGL246C
YLL045C	YHR170W
YBR143C	YDR172W
YBR143C	YIL074C
YBR143C	YBR136W
YBR143C	YBR079C
YBR143C	YOR096W
YBR143C	YPL083C
YBR143C	YDL058W
YBR143C	YLR386W
YBR143C	YOR069W
YBR143C	YHR203C
YDR172W	YBR136W
YDR172W	YLR371W
YDR172W	YOR150W
YDR172W	YPL083C

YDR172WYDL058W
YDR172WYLR386W
YDR172WYOR069W
YDR172WYIL129C
YBL038W YBR282W
YBL038W YDR116C
YBL038W YKR006C
YBL038W YKL167C
YBL038W YMR098C
YBL038W YKR085C
YBL038W YNL252C
YBL038W YLR189C
YBL038W YDR164C
YBL038W YJL005W
YBL038W YPR100W
YBL038W YCR046C
YBL038W YLR312W-A
YBL038W YNL005C
YBL038W YJL063C
YBL038W YML025C
YBL038W YCL014W
YBL038W YDR462W
YBL038W YCR071C
YBL038W YMR064W
YBL038W YMR225C
YBL038W YOR201C
YBL038W YDR237W
YBL038W YDR322W
YBL038W YGR220C
YBL038W YBL004W
YBL038W YMR193W
YBL038W YMR024W
YBL038W YDR296W
YBL038W YLR439W
YBL038W YOR150W
YBL038W YNL177C
YBL038W YHR147C
YBL038W YPL183W-A
YBL038W YBR122C
YBL038W YDR405W
YBL038W YIL104C
YBR282W YDR116C
YBR282W YKR006C
YBR282W YKL167C
YBR282W YMR098C

YBR282W	YKR085C
YBR282W	YNL252C
YBR282W	YCR046C
YBR282W	YLR312W-A
YBR282W	YNL005C
YBR282W	YJL063C
YBR282W	YML025C
YBR282W	YDR462W
YBR282W	YCR071C
YBR282W	YMR225C
YBR282W	YOR201C
YBR282W	YDR237W
YBR282W	YDR322W
YBR282W	YGR220C
YBR282W	YMR193W
YBR282W	YMR024W
YBR282W	YDR296W
YBR282W	YLR439W
YBR282W	YOR150W
YBR282W	YNL177C
YBR282W	YHR147C
YBR282W	YBR122C
YBR282W	YDR405W
YKL122C	YPL210C
YKL122C	YPL243W
YKL122C	YDL092W
YKL122C	YML105C
YKL122C	YPR088C
YPL210C	YDL051W
YPL210C	YER155C
YPL210C	YPL243W
YPL210C	YPL240C
YPL210C	YDL092W
YPL210C	YML105C
YPL210C	YLR452C
YPL210C	YPR088C
YDR457W	YGR204W
YDR457W	YDL112W
YDR457W	YBR121C
YDR457W	YCL050C
YDR457W	YPL218W
YDR457W	YKL029C
YDR457W	YFL045C
YDR457W	YDR341C
YDR457W	YLR355C

YDR457WYGL238W
YDR457WYHR128W
YDR457WYJL052W
YDR457WYNR016C
YDR457WYKL067W
YDR457WYLL018C
YDR457WYDR335W
YDR457WYBL076C
YDR457WYLL040C
YDR457WYBL039C
YDR457WYHL011C
YGR204WYFR031C-A
YGR204WYDL112W
YGR204WYBR263W
YGR204WYPL009C
YGR204WYBR121C
YGR204WYPL218W
YGR204WYDR341C
YGR204WYLR355C
YGR204WYGL238W
YGR204WYHR128W
YGR204WYJL052W
YGR204WYKL067W
YGR204WYLL018C
YGR204WYOR048C
YGR204WYLL040C

YMR128W	YOR078W
YMR128W	YGR090W
YMR128W	YCL059C
YMR128W	YOR310C
YMR128W	YDR324C
YMR128W	YPR144C
YMR128W	YDL213C
YMR128W	YPL139C
YMR128W	YMR229C
YMR128W	YPL070W
YMR128W	YIL084C
YMR128W	YDL148C
YMR128W	YCR057C
YMR128W	YOL010W
YMR128W	YPR137W
YMR128W	YBR247C
YMR128W	YDR280W
YMR128W	YNL097C
YOR078W	YLR197W

YOR078W YGR090W
YOR078W YPL126W
YOR078W YDR299W
YOR078W YGL171W
YOR078W YCL059C
YOR078W YOR310C
YOR078W YJL109C
YOR078W YKR060W
YOR078W YNL132W
YOR078W YMR143W
YOR078W YDL213C
YOR078W YML130C
YOR078W YBL004W
YOR078W YER082C
YOR078W YHR148W
YOR078W YGR034W
YOR078W YBR084C-A
YOR078W YDR365C
YOR078W YMR093W
YOR078W YPR137W
YOR078W YJR002W
YOR078W YJL033W
YOR078W YGR145W
YOR078W YBR247C
YOR078W YGR128C
YOR078W YPL217C
YOR078W YNR043W
YOR078W YHR169W
YOR078W YJL069C
YOR078W YNL075W
YOR078W YLR222C
YLR180W YMR012W
YLR180W YGL195W
YLR180W YLR106C
YLR180W YIL075C
YLR180W YLR335W
YLR180W YOR151C
YLR180W YFR052W
YLR180W YDR127W
YLR180W YHR019C
YLR180W YLR304C
YLR180W YMR205C
YLR180W YDR502C
YLR180W YGR218W
YLR180W YLL040C

YLR180W	YKL152C
YLR180W	YER090W
YLR180W	YKL211C
YMR012W	YMR116C
YMR012W	YML124C
YMR012W	YKL029C
YMR012W	YHR019C
YMR012W	YBL039C
YMR012W	YBR017C
YIL021W	YOR210W
YIL021W	YOR224C
YIL021W	YGR005C
YIL021W	YOL005C
YIL021W	YJL140W
YIL021W	YOR151C
YIL021W	YPR187W
YIL021W	YGR063C
YIL021W	YML010W
YIL021W	YBR154C
YIL021W	YDR404C
YIL021W	YGR186W
YIL021W	YPR093C
YIL021W	YGL070C
YIL021W	YDL115C
YIL021W	YDL117W
YIL021W	YLR450W
YIL021W	YER139C
YOR210W	YDL213C
YOR210W	YGL156W
YOR210W	YPR032W
YOR210W	YJR063W
YOR210W	YOR340C
YOR210W	YGL070C
YOR210W	YDL115C
YOR210W	YNL248C
YOR210W	YOR341W
YOR210W	YFR028C
YGL005C	YPR105C
YGL005C	YGR120C
YGL005C	YNL051W
YGL005C	YML071C
YGL005C	YNL041C
YGL005C	YER157W
YGL005C	YGL223C
YPR105C	YGR120C

YPR105C YNL051W
YPR105C YML071C
YPR105C YNL041C
YPR105C YER157W
YPR105C YGL223C
YDR116C YKR006C
YDR116C YKL167C
YDR116C YMR098C
YDR116C YKR085C
YDR116C YNL252C
YDR116C YPR100W
YDR116C YCR046C
YDR116C YLR312W-A
YDR116C YNL005C
YDR116C YJL063C
YDR116C YML025C
YDR116C YCL014W
YDR116C YDR462W
YDR116C YCR071C
YDR116C YMR064W
YDR116C YJL041W
YDR116C YMR225C
YDR116C YOR201C
YDR116C YDR237W
YDR116C YDR322W
YDR116C YGR220C
YDR116C YMR193W
YDR116C YMR024W
YDR116C YDR296W
YDR116C YLR439W
YDR116C YOR150W
YDR116C YNL177C
YDR116C YHR147C
YDR116C YPL183W-A
YDR116C YBR122C
YDR116C YDR405W
YDR116C YNL185C
YKR006C YKL167C
YKR006C YMR098C
YKR006C YKR085C
YKR006C YNL252C
YKR006C YLR189C
YKR006C YPR100W
YKR006C YCR046C
YKR006C YLR312W-A

YKR006C YNL005C
YKR006C YJL063C
YKR006C YML025C
YKR006C YCL014W
YKR006C YDR462W
YKR006C YCR071C
YKR006C YMR064W
YKR006C YMR225C
YKR006C YDR237W
YKR006C YDR322W
YKR006C YGR220C
YKR006C YMR193W
YKR006C YMR024W
YKR006C YDR296W
YKR006C YLR439W
YKR006C YOR150W
YKR006C YNL177C
YKR006C YHR147C
YKR006C YPL183W-A
YKR006C YBR122C
YKR006C YDR405W
YKR006C YIL104C
YGR161C YOR267C
YGR161C YMR273C
YGR161C YKR027W
YGR161C YJL098W
YGR161C YGL190C
YGR161C YDL047W
YGR161C YGL197W
YGR161C YLR310C
YGR161C YAL016W
YGR161C YDL134C
YGR161C YGR281W
YGR161C YOR014W
YOR267C YGL115W
YOR267C YJL098W
YOR267C YDL047W
YOR267C YGL197W
YOR267C YLR310C
YOR267C YFR019W
YOR267C YLR249W
YOR267C YFR040W
YOR267C YKR028W
YLR197W YHR196W
YLR197W YCL059C

YLR197W	YOR310C
YLR197W	YJL109C
YLR197W	YPL043W
YLR197W	YCR057C
YLR197W	YER082C
YLR197W	YLR175W
YLR197W	YPR137W
YLR197W	YJL033W
YLR197W	YBR247C
YDR283C	YLR384C
YDR283C	YBL074C
YDR283C	YKL143W
YDR283C	YML091C
YDR283C	YPL204W
YLR384C	YPL086C
YLR384C	YPL101W
YLR384C	YGR200C
YLR384C	YML091C
YLR384C	YPL204W
YLR384C	YHR187W
YLR275W	YGL128C
YLR275W	YDR473C
YLR275W	YKL173W
YLR275W	YNL147W
YLR275W	YDR378C
YLR275W	YER029C
YLR275W	YGR091W
YLR275W	YMR125W
YLR275W	YDL087C
YLR275W	YPL178W
YLR275W	YPL213W
YLR275W	YER112W
YLR275W	YHR086W
YLR275W	YLR298C
YHR085W	YNR053C
YHR085W	YHR052W
YHR085W	YKR081C
YHR085W	YLR106C
YHR085W	YNL002C
YHR085W	YHR197W
YHR085W	YNL182C
YHR085W	YDR101C
YHR085W	YLR406C
YHR085W	YPL093W
YHR085W	YMR242C

YHR085W	YGL076C
YNR053C	YHR052W
YNR053C	YCR072C
YNR053C	YER006W
YNR053C	YER126C
YNR053C	YKR081C
YNR053C	YLR106C
YNR053C	YLR074C
YNR053C	YJR065C
YNR053C	YGR103W
YNR053C	YHR197W
YNR053C	YNL182C
YNR053C	YDR101C
YNR053C	YKL009W
YNR053C	YGR245C
YNR053C	YPR016C
YNR053C	YLR406C
YNR053C	YPL093W
YKL167C	YMR098C
YKL167C	YKR085C
YKL167C	YNL252C
YKL167C	YLR189C
YKL167C	YPR100W
YKL167C	YCR046C
YKL167C	YLR312W-A
YKL167C	YNL005C
YKL167C	YJL063C
YKL167C	YML025C
YKL167C	YCL014W
YKL167C	YDR462W
YKL167C	YCR071C
YKL167C	YMR064W
YKL167C	YMR225C
YKL167C	YDR237W
YKL167C	YDR322W
YKL167C	YGR220C
YKL167C	YMR193W
YKL167C	YMR024W
YKL167C	YDR296W
YKL167C	YLR439W
YKL167C	YOR150W
YKL167C	YNL177C
YKL167C	YHR147C
YKL167C	YPL183W-A
YKL167C	YBR122C

YKL167C	YDR405W
YKL167C	YIL104C
YMR098C	YKR085C
YMR098C	YNL252C
YMR098C	YLR189C
YMR098C	YCR046C
YMR098C	YLR312W-A
YMR098C	YNL005C
YMR098C	YJL063C
YMR098C	YML025C
YMR098C	YCL014W
YMR098C	YDR462W
YMR098C	YCR071C
YMR098C	YMR225C
YMR098C	YDR237W
YMR098C	YDR322W
YMR098C	YLR371W
YMR098C	YGR220C
YMR098C	YMR193W
YMR098C	YDR296W
YMR098C	YLR439W
YMR098C	YOR150W
YMR098C	YNL177C
YMR098C	YHR147C
YMR098C	YPL183W-A
YMR098C	YCR030C
YMR098C	YBR122C
YMR098C	YDR405W
YKR085C	YNL252C
YKR085C	YLR189C
YKR085C	YDR164C
YKR085C	YPR100W
YKR085C	YCR046C
YKR085C	YLR312W-A
YKR085C	YNL005C
YKR085C	YJL063C
YKR085C	YML025C
YKR085C	YCL014W
YKR085C	YDR462W
YKR085C	YCR071C
YKR085C	YMR064W
YKR085C	YMR225C
YKR085C	YDR237W
YKR085C	YDR322W
YKR085C	YGR220C

YKR085C YMR193W
YKR085C YMR024W
YKR085C YDR296W
YKR085C YLR439W
YKR085C YOR150W
YKR085C YNL177C
YKR085C YHR147C
YKR085C YPL183W-A
YKR085C YBR122C
YKR085C YDR405W
YKR085C YIL104C
YDR174WYKR001C
YDR174WYGR261C
YDR174WYNL030W
YDR174WYPL082C
YDR174WYDR507C
YDR174WYNL216W
YDR174WYJL076W
YDR174WYKL112W
YDR174WYCL024W
YDR174WYAL027W
YKR001C YDR224C
YKR001C YMR072W
YKR001C YBR049C
YKR001C YHL025W
YKR001C YDR303C
YKR001C YBR114W
YKR001C YLR052W
YKR001C YFR013W
YKR001C YPL082C
YKR001C YDL002C
YKR001C YBR289W
YKR001C YIL126W
YKR001C YMR075W
YKR001C YER148W
YKR001C YPL016W
YKR001C YDR397C
YKR001C YDR081C
YKR001C YOL072W
YAL038W YFR049W
YFR049W YDR148C
YFR049W YFL018C
YFR049W YMR121C
YFR049W YIL125W
YNL252C YLR189C

YNL252C YPR100W
YNL252C YCR046C
YNL252C YLR312W-A
YNL252C YNL005C
YNL252C YJL063C
YNL252C YML025C
YNL252C YCL014W
YNL252C YDR462W
YNL252C YCR071C
YNL252C YMR064W
YNL252C YMR225C
YNL252C YOR201C
YNL252C YDR237W
YNL252C YDR322W
YNL252C YGR220C
YNL252C YMR193W
YNL252C YMR024W
YNL252C YDR296W
YNL252C YLR439W
YNL252C YOR150W
YNL252C YNL177C
YNL252C YHR147C
YNL252C YPL183W-A
YNL252C YBR122C
YNL252C YDR405W
YNL252C YIL104C
YDR041WYDR337W
YDR041WYNR037C
YDR041WYOR205C
YDR041WYHL004W
YDR041WYKL155C
YDR041WYDR347W
YDR041WYGL129C
YDR041WYDR175C
YDR041WYMR188C
YDR041WYBR146W
YDR041WYGR170W
YDR041WYDR036C
YDR041WYGR165W
YDR041WYNL137C
YDR041WYOR204W
YDR041WYNL306W
YDR041WYBR251W
YDR041WYGR215W
YDR041WYBL090W

YDR041WYGR084C
YDR041WYOR158W
YDR041WYPL013C
YDR041WYKL003C
YDR041WYOR243C
YDR041WYMR158W
YDR041WYJR101W
YDR041WYJR113C
YDR041WYPL118W
YDR041WYNL186W
YDR041WYIL093C
YDR337WYNR037C
YDR337WYOR205C
YDR337WYHL004W
YDR337WYKL155C
YDR337WYDR347W
YDR337WYGL129C
YDR337WYDR175C
YDR337WYMR188C
YDR337WYBR146W
YDR337WYDR036C
YDR337WYNL137C
YDR337WYDR311W
YDR337WYNL306W
YDR337WYBR251W
YDR337WYGR215W
YDR337WYBL090W
YDR337WYGR084C
YDR337WYOR158W
YDR337WYPL013C
YDR337WYKL003C
YDR337WYMR158W
YDR337WYJR101W
YDR337WYJR113C
YDR337WYPL118W
YDR337WYIL093C

YHR052W	YOL041C
YHR052W	YOL077C
YHR052W	YER006W
YHR052W	YKR081C
YHR052W	YLR106C
YHR052W	YPL043W
YHR052W	YOR206W
YHR052W	YFL002C
YHR052W	YFR001W

YHR052W	YGR103W
YHR052W	YMR049C
YHR052W	YLL034C
YHR052W	YPL211W
YHR052W	YPL012W
YHR052W	YDR060W
YHR052W	YDR496C
YHR052W	YHR197W
YHR052W	YNL182C
YHR052W	YNL061W
YHR052W	YDR101C
YHR052W	YKL009W
YHR052W	YDR087C
YHR052W	YOL127W
YHR052W	YGL111W
YHR052W	YPR016C
YHR052W	YGL103W
YHR052W	YKL172W
YHR052W	YLR406C
YHR052W	YPL093W
YHR052W	YNL110C
YHR052W	YLL008W
YHR052W	YDL082W
YHR052W	YEL054C
YHR052W	YHR010W
YHR052W	YDL031W
YHR052W	YPL143W
YBR211C	YPL018W
YBR211C	YDR383C
YBR211C	YIR010W
YBR211C	YJR112W
YBR211C	YBR107C
YBR211C	YDR254W
YBR211C	YPL233W
YBR211C	YLR381W
YBR211C	YJR135C
YBR211C	YAL034W-A
YBR211C	YGR179C
YBR211C	YDR318W
YPL018W	YDR383C
YPL018W	YIR010W
YPL018W	YBR107C
YPL018W	YPL243W
YPL018W	YDR254W
YPL018W	YLR381W

YPL018W	YJR135C
YPL018W	YMR308C
YPL018W	YGR179C
YPL018W	YDR318W
YER172C	YGL128C
YER172C	YLL036C
YER172C	YMR288W
YER172C	YML049C
YER172C	YBR055C
YER172C	YDR473C
YER172C	YKL173W
YER172C	YNL147W
YER172C	YBL026W
YER172C	YGL120C
YER172C	YLR424W
YER172C	YDL098C
YER172C	YDR378C
YER172C	YER029C
YER172C	YGR091W
YER172C	YDR416W
YER172C	YLR117C
YER172C	YGR074W
YER172C	YMR125W
YER172C	YDL087C
YER172C	YCR063W
YER172C	YGL100W
YER172C	YPR101W
YER172C	YHR156C
YER172C	YMR240C
YER172C	YPL213W
YER172C	YDL209C
YER172C	YHR165C
YER172C	YBR065C
YER172C	YJR050W
YER172C	YPR182W
YER172C	YBL104C
YER172C	YMR213W
YER172C	YBR152W
YER172C	YER112W
YER172C	YML046W
YER172C	YLR147C
YER172C	YGR278W
YER172C	YOR308C
YER172C	YER146W
YER172C	YPR178W

YER172C	YPL151C
YER172C	YPR082C
YER172C	YAL032C
YER172C	YJL203W
YER172C	YDL030W
YGL128C	YLL036C
YGL128C	YKL173W
YGL128C	YGL120C
YGL128C	YLR424W
YGL128C	YER029C
YGL128C	YLR117C
YGL128C	YCR063W
YGL128C	YHR156C
YGL128C	YHR165C
YGL128C	YBR065C
YGL128C	YJR050W
YGL128C	YBL104C
YGL128C	YMR213W
YGL128C	YAL032C
YGL128C	YKR022C
YHR027C	YHR200W
YHR027C	YOR261C
YHR027C	YDR427W
YHR027C	YER021W
YHR027C	YHL030W
YHR027C	YLR421C
YHR027C	YFR004W
YHR027C	YFR052W
YHR027C	YPR108W
YHR027C	YDL147W
YHR027C	YFR010W
YHR027C	YGR232W
YHR027C	YKL145W
YHR027C	YDL007W
YHR027C	YGL048C
YHR027C	YDR394W
YHR027C	YJL001W
YHR027C	YOR117W
YHR200W	YGR135W
YHR200W	YOR261C
YHR200W	YPR103W
YHR200W	YDR427W
YHR200W	YIL075C
YHR200W	YER021W
YHR200W	YHL030W

YHR200W	YLR421C
YHR200W	YFR004W
YHR200W	YFR052W
YHR200W	YPR108W
YHR200W	YDL147W
YHR200W	YFR010W
YHR200W	YGR232W
YHR200W	YML092C
YHR200W	YOL038W
YHR200W	YFR050C
YHR200W	YKL145W
YHR200W	YMR314W
YHR200W	YDL007W
YHR200W	YGL048C
YHR200W	YBL041W
YHR200W	YER012W
YHR200W	YDR394W
YHR200W	YBL039C
YBR084W	YHR064C
YBR084W	YMR116C
YBR084W	YFR031C-A
YBR084W	YLR448W
YBR084W	YAL035W
YBR084W	YGR285C
YBR084W	YBL022C
YHR064C	YJL080C
YHR064C	YKR057W
YHR064C	YML124C
YHR064C	YDR064W
YHR064C	YDR418W
YHR064C	YAL036C
YHR064C	YER155C
YHR064C	YGR162W
YHR064C	YBR158W
YHR064C	YGR285C
YHR064C	YHR010W
YHR064C	YPR189W
YHR064C	YBR175W
YDR051C	YER048W-A
YER048W-A	YCL017C
YDR383C	YIR010W
YDR383C	YBR107C
YDR383C	YDR254W
YDR383C	YLR381W
YDR383C	YJR135C

YDR383C YGR179C
YDR383C YDR318W
YIR010W YJR112W
YIR010W YDR254W
YIR010W YPL233W
YIR010W YLR381W
YIR010W YJR135C
YIR010W YAL034W-A
YIR010W YGR179C
YIR010W YDR318W
YDR392WYOL148C
YDR392WYHR099W
YDR392WYLR055C
YDR392WYDR176W
YDR392WYDR448W
YDR392WYGL066W
YDR392WYGL112C
YDR392WYGR252W
YDR392WYBR198C
YDR392WYPL254W
YDR392WYMR223W
YDR392WYCL010C
YDR392WYBR081C
YOL148C YHR099W
YOL148C YLR055C
YOL148C YDR176W
YOL148C YML007W
YOL148C YDR448W
YOL148C YGL066W
YOL148C YGL112C
YOL148C YGR252W
YOL148C YBR198C
YOL148C YDR145W
YOL148C YPL254W
YOL148C YMR223W
YOL148C YDR167W
YOL148C YCL010C
YOL148C YBR081C
YML062C YNL253W
YML062C YIL075C
YML062C YBR025C
YML062C YNL004W
YML062C YDR381W
YML062C YHR167W
YML062C YDR138W

YML062C	YNL139C
YML062C	YDL084W
YNL253W	YNL004W
YNL253W	YHR167W
YNL253W	YDR138W
YNL253W	YNL139C
YNL253W	YDL084W
YJL080C	YMR116C
YJL080C	YKR057W
YJL080C	YHL015W
YJL080C	YJR123W
YJL080C	YAL036C
YJL080C	YJR145C
YJL080C	YLR226W
YJL080C	YGR285C
YJL080C	YPR161C
YJL080C	YPR057W
YMR116C	YNL224C
YMR116C	YOR361C
YMR116C	YLR330W
YMR116C	YGR285C
YMR116C	YDL040C
YMR116C	YHR013C
YMR116C	YJL099W
YMR116C	YPR189W
YMR116C	YGL213C
YMR116C	YBL061C
YNL071W	YOR132W
YNL071W	YER118C
YNL071W	YBR221C
YNL071W	YGR193C
YNL071W	YDR430C
YNL071W	YER178W
YNL071W	YDR338C
YOR132W	YOR069W
YOR132W	YJL154C
YOR132W	YHR012W
YAL002W	YLR396C
YAL002W	YMR231W
YAL002W	YPL045W
YAL002W	YDL077C
YAL002W	YLR148W
YAL002W	YDR080W
YLR396C	YMR231W
YLR396C	YPL045W

YLR396C	YDL077C
YLR396C	YLR148W
YLR396C	YDR080W
YLL036C	YMR288W
YLL036C	YKL173W
YLL036C	YDR364C
YLL036C	YDR416W
YLL036C	YLR117C
YLL036C	YCR063W
YLL036C	YPR101W
YLL036C	YHR156C
YLL036C	YPL213W
YLL036C	YDL209C
YLL036C	YHR165C
YLL036C	YBR065C
YLL036C	YJR050W
YLL036C	YMR213W
YLL036C	YGR278W
YLL036C	YPL151C
YLL036C	YAL032C
YLL036C	YDL030W
YLL036C	YLR132C
YMR288W	YML049C
YMR288W	YNL286W
YMR288W	YER029C
YMR288W	YDL087C
YMR288W	YMR240C
YMR288W	YPL213W
YMR288W	YPR182W
YMR288W	YMR213W
YMR288W	YER112W
YMR288W	YGR278W
YMR288W	YAL032C
YMR288W	YJL203W
YFL034W	YKL135C
YFL034W	YHL019C
YFL034W	YHR023W
YFL034W	YLR170C
YFL034W	YPR029C
YKL135C	YHL019C
YKL135C	YLR170C
YKL135C	YPR029C
YKL135C	YPL259C
YKL135C	YBL027W
YKL135C	YBL032W

YPR190C	YOR224C
YPR190C	YDR045C
YPR190C	YNL151C
YPR190C	YKR025W
YPR190C	YOR116C
YPR190C	YPR187W
YPR190C	YBR154C
YPR190C	YDR005C
YPR190C	YNR003C
YPR190C	YDL150W
YPR190C	YOR207C
YLR189C	YDR164C
YLR189C	YJL005W
YLR189C	YPR100W
YLR189C	YLR312W-A
YLR189C	YJL063C
YLR189C	YML025C
YLR189C	YCL014W
YLR189C	YDR462W
YLR189C	YCR071C
YLR189C	YMR225C
YLR189C	YDR237W
YLR189C	YDR322W
YLR189C	YGR220C
YLR189C	YMR193W
YLR189C	YLR439W
YLR189C	YOR150W
YLR189C	YHR147C
YLR189C	YPL183W-A
YLR189C	YCR030C
YLR189C	YBR122C
YLR189C	YDR405W
YLR189C	YGL043W
YOL041C	YOL077C
YOL041C	YPL043W
YOL041C	YOR206W
YOL041C	YMR229C
YOL041C	YLR196W
YOL041C	YDR060W
YOL041C	YOR312C
YOL041C	YLR029C
YOL041C	YPL198W
YOL077C	YER006W
YOL077C	YKR081C
YOL077C	YPL043W

YOL077C	YNL002C
YOL077C	YOR206W
YOL077C	YFL002C
YOL077C	YGR103W
YOL077C	YMR049C
YOL077C	YLL034C
YOL077C	YPL211W
YOL077C	YLR196W
YOL077C	YDR060W
YOL077C	YDR496C
YOL077C	YHR197W
YOL077C	YNL061W
YOL077C	YKL009W
YOL077C	YDR087C
YOL077C	YGL111W
YOL077C	YKL172W
YOL077C	YPL093W
YOL077C	YNL110C
YOL077C	YLL008W
YOL077C	YDL082W
YOL077C	YDL031W
YOL077C	YER117W
YCR073W-A	YLR258W
YCR073W-A	YDR028C
YCR073W-A	YER177W
YCR073W-A	YKL193C
YCR073W-A	YLR028C
YCR073W-A	YER133W
YCR073W-A	YER070W
YCR073W-A	YAR014C
YLR258W	YDR028C
YLR258W	YKL193C
YLR258W	YLR028C
YLR258W	YER133W
YLR258W	YER070W
YLR258W	YAR014C
YCR072C	YER006W
YCR072C	YER126C
YCR072C	YLR106C
YCR072C	YLR074C
YCR072C	YHR197W
YCR072C	YNL182C
YCR072C	YDR101C
YCR072C	YGR245C
YER006W	YER126C

YER006W YKR081C
YER006W YLR106C
YER006W YLR074C
YER006W YLR325C
YER006W YPL043W
YER006W YOR206W
YER006W YFL002C
YER006W YFR001W
YER006W YGR103W
YER006W YMR049C
YER006W YPL211W
YER006W YLR449W
YER006W YOR349W
YER006W YGR220C
YER006W YDR496C
YER006W YNL175C
YER006W YDR296W
YER006W YNL061W
YER006W YDR101C
YER006W YGR245C
YER006W YOL127W
YER006W YPR016C
YER006W YPL093W
YER006W YNL110C
YER006W YPR043W
YER006W YHR010W
YER006W YPR102C
YER006W YDL031W
YGR135WYOR261C
YGR135WYOR362C
YGR135WYPR103W
YGR135WYML092C
YGR135WYOL038W
YGR135WYFR050C
YGR135WYBL041W
YGR135WYJL001W
YGR135WYKL210W
YDL126C YDR028C
YDL126C YGL246C
YDL126C YBR170C
YDL126C YDR037W
YDL126C YDR049W
YDL126C YNL271C
YDL126C YBL058W
YDL126C YKL039W

YDL126C YML013W
YDL126C YGR048W
YDR028C YNL076W
YDR028C YDR422C
YDR028C YGL115W
YDR028C YBR265W
YDR028C YBL023C
YDR028C YBR126C
YDR028C YPR035W
YDR028C YLR028C
YDR028C YER133W
YDR028C YDR049W
YDR028C YER027C
YDR028C YGL208W
YDR028C YNR034W
YDR028C YAR014C
YDR028C YER129W
YDR028C YDR477W
YJR042W YPL169C
YJR042W YKL057C
YJR042W YLR208W
YJR042W YDL116W
YJR042W YGL100W
YJR042W YLR371W
YJR042W YGL092W
YPL169C YKL057C
YPL169C YDL116W
YPL169C YLR371W
YPL169C YGL092W
YPL169C YBR011C
YGR090WYPL126W
YGR090WYDR299W
YGR090WYGL171W
YGR090WYHR196W
YGR090WYDR449C
YGR090WYOR310C
YGR090WYDR324C
YGR090WYPR144C
YGR090WYJL109C
YGR090WYOL021C
YGR090WYKR060W
YGR090WYNL132W
YGR090WYOR145C
YGR090WYOR039W
YGR090WYMR229C

YGR090WYLR409C
YGR090WYDL148C
YGR090WYPL266W
YGR090WYBL004W
YGR090WYCR057C
YGR090WYER082C
YGR090WYHR148W
YGR090WYLR186W
YGR090WYPR137W
YGR090WYJR002W
YGR090WYLR003C
YGR090WYDL014W
YGR090WYGR145W
YGR090WYLR129W
YGR090WYBR247C
YGR090WYGL019W
YGR090WYGR128C
YGR090WYDR280W
YGR090WYOR061W
YGR090WYPR112C
YGR090WYJL069C
YGR090WYCL031C
YGR090WYLR223C
YPL126W YHR196W
YPL126W YCL059C
YPL126W YDR449C
YPL126W YOR310C
YPL126W YDR324C
YPL126W YPR144C
YPL126W YJL109C
YPL126W YOR145C
YPL126W YMR229C
YPL126W YDL148C
YPL126W YCR057C
YPL126W YER082C
YPL126W YHR148W
YPL126W YMR093W
YPL126W YPR137W
YPL126W YGR128C
YPL126W YNL075W
YGR158C YML056C
YGR158C YCR035C
YGR158C YHR069C
YGR158C YJL109C
YGR158C YOL021C

YGR158C	YOR076C
YGR158C	YHR081W
YGR158C	YNL232W
YGR158C	YDL111C
YGR158C	YDR280W
YGR158C	YPR189W
YGR158C	YOR001W
YGR158C	YLR398C
YGR158C	YOL142W
YGR158C	YKL126W
YML056C	YGL173C
YML056C	YKL130C
YML056C	YDL209C
YML056C	YHR216W
YML056C	YJL052W
YML056C	YLR432W
YML056C	YAL029C
YML056C	YDR280W
YML056C	YJR009C
YML056C	YER102W
YGR086C	YPL004C
YGR086C	YDL019C
YGR086C	YER120W
YGR086C	YDR150W
YGR086C	YGR130C
YGR086C	YHL020C
YGR086C	YKL142W
YGR086C	YMR031C
YGR086C	YDR490C
YPL004C	YKL142W
YER081W	YIL074C
YIL074C	YAR042W
YDR164C	YJL005W
YDR164C	YPR100W
YDR164C	YJR138W
YDR164C	YJL063C
YDR164C	YCL014W
YDR164C	YDR462W
YDR164C	YOR204W
YDR164C	YOR150W
YDR164C	YBR196C
YJL005W	YFL039C
YJL005W	YPR100W
YJL005W	YCL014W
YJL005W	YNL138W

YJL005W	YGR233C
YJL005W	YOL001W
YER126C	YKR081C
YER126C	YLR074C
YER126C	YNL002C
YER126C	YOR206W
YER126C	YMR049C
YER126C	YHR197W
YER126C	YDR101C
YER126C	YKL009W
YER126C	YGR245C
YER126C	YPR016C
YER126C	YPL093W
YER126C	YNL110C
YER126C	YOR063W
YKR081C	YLR074C
YKR081C	YNL002C
YKR081C	YOR206W
YKR081C	YFL002C
YKR081C	YGR103W
YKR081C	YMR049C
YKR081C	YLL034C
YKR081C	YPL211W
YKR081C	YDR060W
YKR081C	YDR496C
YKR081C	YHR197W
YKR081C	YNL061W
YKR081C	YDR101C
YKR081C	YDR087C
YKR081C	YGR245C
YKR081C	YOL127W
YKR081C	YGL111W
YKR081C	YPR016C
YKR081C	YKL172W
YKR081C	YLR406C
YKR081C	YPL093W
YKR081C	YNL110C
YKR081C	YLL008W
YKR081C	YDL031W
YKR081C	YLR276C
YKR081C	YGR085C
YBR136W	YDR097C
YBR136W	YIR002C
YBR136W	YBR114W
YBR136W	YPL083C

YBR136W	YAR007C
YBR136W	YJL173C
YBR136W	YLR386W
YBR136W	YNL312W
YBR136W	YCR092C
YBR136W	YML032C
YBR136W	YDR499W
YBR136W	YDL156W
YBR136W	YHR164C
YBR136W	YOL090W
YDR097C	YIR002C
YDR097C	YBR114W
YDR097C	YAR007C
YDR097C	YNL312W
YDR097C	YCR092C
YDR097C	YML032C
YDR097C	YDR499W
YDR097C	YDL156W
YDR097C	YHR164C
YDR097C	YOL090W
YLL004W	YPR162C
YLL004W	YNL261W
YLL004W	YHR118C
YLL004W	YML065W
YLL004W	YBR060C
YPR162C	YNL261W
YPR162C	YHR118C
YPR162C	YML065W
YPR162C	YBR060C
YFL039C	YMR109W
YFL039C	YMR072W
YFL039C	YOR244W
YFL039C	YNL138W
YFL039C	YNL059C
YFL039C	YHR023W
YFL039C	YFL024C
YFL039C	YDL002C
YFL039C	YAL029C
YFL039C	YDR190C
YFL039C	YNL218W
YFL039C	YGL150C
YFL039C	YFL013C
YFL039C	YDR129C
YFL039C	YLR454W
YMR109W	YDL019C

YMR109W	YKL057C
YMR109W	YKL130C
YMR109W	YLR429W
YMR109W	YBR202W
YMR109W	YBR130C
YMR109W	YPL153C
YMR109W	YGL157W
YMR109W	YBR177C
YMR109W	YIL034C
YMR109W	YBR109C
YMR109W	YKL007W
YBL003C	YDR224C
YBL003C	YHR099W
YBL003C	YDR303C
YBL003C	YFR013W
YBL003C	YNL030W
YBL003C	YPL082C
YBL003C	YDR083W
YBL003C	YOR217W
YBL003C	YOL004W
YBL003C	YGL241W
YBL003C	YKR048C
YBL003C	YOR207C
YBL003C	YPL128C
YDR224C	YOR116C
YDR224C	YBR009C
YDR224C	YDR303C
YDR224C	YDL167C
YDR224C	YGL133W
YDR224C	YPL082C
YDR224C	YLR357W
YDR224C	YGL241W
YDR224C	YPL084W
YDR224C	YOR304W
YDR224C	YPL128C
YLR321C	YML127W
YLR321C	YPR034W
YLR321C	YMR091C
YLR321C	YMR033W
YLR321C	YDR303C
YLR321C	YKR008W
YLR321C	YGR056W
YLR321C	YLR357W
YLR321C	YCR052W
YLR321C	YIL126W

YLR321C	YLR033W
YLR321C	YGR275W
YML127W	YMR091C
YML127W	YDR303C
YML127W	YKR008W
YML127W	YGR056W
YML127W	YLR357W
YML127W	YCR052W
YML127W	YLR033W
YGR020C	YHR039C-A
YGR020C	YLR447C
YGR020C	YPR036W
YGR020C	YEL051W
YGR020C	YOR270C
YGR020C	YKL119C
YGR020C	YDR202C
YGR020C	YDR516C
YGR020C	YOR332W
YGR020C	YDL185W
YGR020C	YKL080W
YGR020C	YJR033C
YHR039C-A	YBR127C
YHR039C-A	YPR036W
YHR039C-A	YEL051W
YHR039C-A	YOR270C
YHR039C-A	YOR332W
YHR039C-A	YKL080W
YHR039C-A	YJR033C
YKR057W	YOR145C
YKR057W	YLR264W
YKR057W	YGR285C
YKR057W	YKL143W
YKR057W	YOR056C
YKR057W	YGR054W
YKR057W	YPL204W
YML049C	YNL286W
YML049C	YDR378C
YML049C	YER029C
YML049C	YLR117C
YML049C	YMR125W
YML049C	YDL087C
YML049C	YPL178W
YML049C	YMR240C
YML049C	YPL213W
YML049C	YDL209C

YML049C YHR165C
YML049C YPR182W
YML049C YOR319W
YML049C YMR213W
YML049C YER112W
YML049C YAL032C
YML049C YJL203W
YDR395W YGL195W
YDR395W YKR060W
YDR395W YBR115C
YDR395W YAL059W
YGL195W YBR025C
YGL195W YFL045C
YGL195W YIL094C
YGL195W YOR136W
YGL195W YJR077C
YGL195W YBL030C
YGL195W YFR009W
YGL195W YER110C
YGL195W YDR214W
YCR002C YNL233W
YCR002C YHR107C
YCR002C YLR314C
YCR002C YDL225W
YCR002C YJR076C
YCR002C YJR092W
YNL233W YHR107C
YNL233W YLR314C
YNL233W YDL225W
YNL233W YJR076C
YGR274C YNL236W
YGR274C YCR042C
YGR274C YDR448W
YGR274C YHR023W
YGR274C YMR005W
YGR274C YGL112C
YGR274C YMR236W
YGR274C YBR198C
YGR274C YML015C
YGR274C YPL011C
YGR274C YMR227C
YGR274C YDR145W
YGR274C YER148W
YGR274C YML114C
YNL236W YPR070W

YNL236WYBR193C
YNL236WYLR071C
YNL236WYER022W
YNL236WYNL025C
YNL236WYML007W
YNL236WYOL135C
YNL236WYGL151W
YNL236WYOL051W
YNL236WYBR253W
YNL236WYHR041C
YNL236WYDR308C
YNL236WYCR081W
YNL236WYDR443C
YNL236WYGR104C
YNL236WYDR145W
YNL236WYPL254W
YPR100WYCR046C
YPR100WYLR312W-A
YPR100WYJR138W
YPR100WYNL005C
YPR100WYJL063C
YPR100WYML025C
YPR100WYCL014W
YPR100WYDR462W
YPR100WYCR071C
YPR100WYMR064W
YPR100WYDR237W
YPR100WYDR322W
YPR100WYGR220C
YPR100WYMR024W
YPR100WYDR296W
YPR100WYLR439W
YPR100WYOR150W
YPR100WYNL177C
YPR100WYPL183W-A
YPR100WYBR122C
YPR100WYIL104C
YDR299WYGL171W
YDR299WYCL059C
YDR299WYPR144C
YDR299WYKR060W
YDR299WYML130C
YDR299WYDL148C
YDR299WYCR057C
YDR299WYER082C

YDR299WYHR148W
YDR299WYLR186W
YDR299WYDR365C
YDR299WYJR002W
YDR299WYLR003C
YDR299WYJL033W
YDR299WYGR145W
YDR299WYBR247C
YDR299WYNR043W
YDR299WYHR169W
YDR299WYDL166C
YDR299WYNL308C
YDR299WYCL031C
YGL171W YDR449C
YGL171W YPR144C
YGL171W YKR060W
YGL171W YML130C
YGL171W YCR057C
YGL171W YER082C
YGL171W YHR148W
YGL171W YLR186W
YGL171W YDR365C
YGL171W YLR003C
YGL171W YJL033W
YGL171W YGR145W
YGL171W YNR043W
YGL171W YHR169W
YGL171W YCL031C
YGL171W YML063W
YGL171W YER074W
YHR196W YDR449C
YHR196W YDR064W
YHR196W YOR310C
YHR196W YDR324C
YHR196W YPR144C
YHR196W YJL109C
YHR196W YNL132W
YHR196W YOR145C
YHR196W YJR123W
YHR196W YPL012W
YHR196W YDL148C
YHR196W YPL266W
YHR196W YBL004W
YHR196W YCR057C
YHR196W YER082C

YHR196W	YHR148W
YHR196W	YLR186W
YHR196W	YOL010W
YHR196W	YMR093W
YHR196W	YPR137W
YHR196W	YJR002W
YHR196W	YLR003C
YHR196W	YDL014W
YHR196W	YLR129W
YHR196W	YGR128C
YHR196W	YPL217C
YHR196W	YLL011W
YHR196W	YNL075W
YHR196W	YBL072C
YIL128W	YNL240C
YIL128W	YDL185W
YIL128W	YER171W
YIL128W	YDR267C
YIL128W	YHR122W
YIL128W	YMR178W
YNL240C	YHR122W
YCR046C	YLR312W-A
YCR046C	YNL005C
YCR046C	YJL063C
YCR046C	YML025C
YCR046C	YCL014W
YCR046C	YDR462W
YCR046C	YCR071C
YCR046C	YMR064W
YCR046C	YMR225C
YCR046C	YOR201C
YCR046C	YDR237W
YCR046C	YDR322W
YCR046C	YGR220C
YCR046C	YMR193W
YCR046C	YMR024W
YCR046C	YDR296W
YCR046C	YLR439W
YCR046C	YOR150W
YCR046C	YNL177C
YCR046C	YHR147C
YCR046C	YPL183W-A
YCR046C	YBR122C
YCR046C	YDR405W
YBR055C	YDR473C

YBR055C	YKL173W
YBR055C	YNL147W
YBR055C	YBL026W
YBR055C	YDR378C
YBR055C	YER029C
YBR055C	YGR091W
YBR055C	YGR074W
YBR055C	YHR165C
YBR055C	YBR152W
YBR055C	YER112W
YBR055C	YER146W
YBR055C	YPR178W
YDR473C	YKL173W
YDR473C	YBL026W
YDR473C	YDL098C
YDR473C	YDR378C
YDR473C	YER029C
YDR473C	YGR091W
YDR473C	YGR074W
YDR473C	YHR156C
YDR473C	YHR165C
YDR473C	YBR152W
YDR473C	YER112W
YDR473C	YLR147C
YDR473C	YOR308C
YDR473C	YPR178W
YDR473C	YPR082C
YDR473C	YGR075C
YLR259C	YOL098C
YLR259C	YER086W
YLR259C	YHR208W
YLR259C	YER178W
YLR259C	YJR148W
YLR259C	YNL085W
YOL098C	YIL075C
YOL098C	YKL145W
YOL098C	YDL055C
YOL098C	YOR133W
YOL098C	YPL208W
YOL098C	YDR361C
YOL098C	YMR243C
YFR014C	YOL097C
YFR014C	YBL030C
YFR014C	YLR153C
YFR014C	YML028W

YOL097C	YMR315W
YOL097C	YBR114W
YOL097C	YBL030C
YOL097C	YLR153C
YOL097C	YPL154C
YOL097C	YOR332W
YOL097C	YML028W
YJR093C	YPR107C
YJR093C	YKL018W
YJR093C	YMR061W
YJR093C	YLR277C
YJR093C	YNL317W
YJR093C	YAL043C
YJR093C	YDR195W
YJR093C	YKR002W
YJR093C	YGR156W
YJR093C	YKL059C
YJR093C	YDR301W
YJR093C	YLR115W
YJR093C	YPR074C
YPR107C	YKL018W
YPR107C	YLR277C
YPR107C	YNL317W
YPR107C	YAL043C
YPR107C	YER133W
YPR107C	YDR195W
YPR107C	YKR002W
YPR107C	YGR156W
YPR107C	YKL059C
YPR107C	YDR301W
YPR107C	YLR115W
YPR107C	YNL222W
YLR312W-A	YNL005C
YLR312W-A	YJL063C
YLR312W-A	YML025C
YLR312W-A	YCL014W
YLR312W-A	YDR462W
YLR312W-A	YCR071C
YLR312W-A	YMR064W
YLR312W-A	YJL041W
YLR312W-A	YMR225C
YLR312W-A	YDR237W
YLR312W-A	YDR322W
YLR312W-A	YGR220C
YLR312W-A	YMR193W

YLR312W-A	YMR024W
YLR312W-A	YDR296W
YLR312W-A	YLR439W
YLR312W-A	YOR150W
YLR312W-A	YNL177C
YLR312W-A	YPL183W-A
YLR312W-A	YBR122C
YLR312W-A	YDR405W
YLR312W-A	YIL104C
YLR106C	YML124C
YLR106C	YLR074C
YLR106C	YPL043W
YLR106C	YGR103W
YLR106C	YKL029C
YLR106C	YDR101C
YLR106C	YGR245C
YLR106C	YPL093W
YLR106C	YNL110C
YLR106C	YJR132W
YML124C	YNL301C
YML124C	YKL029C
YML124C	YDR394W
YML124C	YFR009W
YML124C	YDR188W
YDL051W	YGR162W
YDL051W	YPL243W
YDL051W	YPR016C
YDL051W	YDL092W
YDL051W	YPR088C
YCL059C	YDR449C
YCL059C	YDR324C
YCL059C	YPR144C
YCL059C	YJL109C
YCL059C	YKR060W
YCL059C	YNL132W
YCL059C	YDL213C
YCL059C	YLR409C
YCL059C	YDL148C
YCL059C	YBL004W
YCL059C	YER082C
YCL059C	YHR148W
YCL059C	YLR186W
YCL059C	YDR365C
YCL059C	YLR175W
YCL059C	YMR093W

YCL059C	YPR137W
YCL059C	YJR002W
YCL059C	YDL014W
YCL059C	YGR145W
YCL059C	YBR247C
YCL059C	YPL217C
YCL059C	YNR043W
YCL059C	YLL011W
YCL059C	YDL166C
YCL059C	YBL072C
YCL059C	YNL255C
YDR449C	YOR310C
YDR449C	YDR324C
YDR449C	YPR144C
YDR449C	YJL109C
YDR449C	YNL132W
YDR449C	YLR409C
YDR449C	YBL004W
YDR449C	YCR057C
YDR449C	YER082C
YDR449C	YHR148W
YDR449C	YLR186W
YDR449C	YMR093W
YDR449C	YPR137W
YDR449C	YGR145W
YDR449C	YLR129W
YDR449C	YBR247C
YDR449C	YLL011W
YDR449C	YJL069C
YDR449C	YLR222C
YDR449C	YBL072C
YDL019C	YKL057C
YDL019C	YAR042W
YDL019C	YER120W
YDL019C	YDR150W
YDL019C	YHL020C
YDL019C	YBR177C
YKL057C	YLR208W
YKL057C	YDL116W
YKL057C	YGL100W
YKL057C	YGL092W
YKL057C	YLR357W
YKL057C	YBR177C
YML026C	YOR056C
YML026C	YBR101C

YNL250WYOR261C
YNL250WYFR004W
YNL250WYER092W
YNL250WYBL056W
YNL250WYDR394W
YNL250WYER151C
YNL250WYBR097W
YNL250WYNR051C
YOR261C YDR427W
YOR261C YIL075C
YOR261C YHL030W
YOR261C YLR421C
YOR261C YFR052W
YOR261C YPR108W
YOR261C YDL147W
YOR261C YFR010W
YOR261C YGR232W
YOR261C YOL038W
YOR261C YFR050C
YOR261C YKL145W
YOR261C YMR314W
YOR261C YLR035C
YAR042WYER120W
YAR042WYDR150W
YAR042WYHL020C
YAR042WYLR183C
YAR042WYGR246C
YAR042WYLR397C
YAR042WYHR195W
YGL173C YNL147W
YGL173C YBL026W
YGL173C YOL149W
YGL173C YDR378C
YGL173C YPL190C
YGL173C YCR077C
YGL173C YGR162W
YGL173C YNL251C
YGL173C YER112W
YGL173C YJL124C
YGL173C YNL118C
YGL173C YER146W
YGL173C YLR438C-A
YDR064WYOR310C
YDR064WYPR144C
YDR064WYJL109C

YDR064WYNL132W
YDR064WYBR079C
YDR064WYPR137W
YDR064WYGR285C
YDR064WYOR056C
YDR064WYPR189W
YDR064WYBR017C
YOR310C YDR324C
YOR310C YGL120C
YOR310C YJL109C
YOR310C YKR060W
YOR310C YNL132W
YOR310C YML130C
YOR310C YLR409C
YOR310C YDL148C
YOR310C YBL004W
YOR310C YCR057C
YOR310C YOR096W
YOR310C YLR186W
YOR310C YDR365C
YOR310C YMR093W
YOR310C YJR002W
YOR310C YLR003C
YOR310C YDL014W
YOR310C YJL033W
YOR310C YNR043W
YOR310C YLL011W
YOR310C YHR169W
YOR310C YBL072C
YOR310C YCL037C
YJR138W YNL076W
YJR138W YCL014W
YJR138W YGR170W
YJR138W YMR176W
YJR138W YBR265W
YJR138W YBL023C
YJR138W YOR349W
YJR138W YOL054W
YJR138W YBR126C
YJR138W YPR035W
YJR138W YGL190C
YJR138W YML071C
YJR138W YDL167C
YJR138W YDL070W
YJR138W YOL145C

YJR138W	YIR001C
YJR138W	YFR006W
YNL076W	YGL252C
YNL076W	YER177W
YNL076W	YBR265W
YNL076W	YBL023C
YNL076W	YBR126C
YNL076W	YPR035W
YNL076W	YML100W
YNL076W	YDR074W
YNL076W	YMR261C
YOR224C	YPR010C
YOR224C	YOR116C
YOR224C	YGR005C
YOR224C	YOR151C
YOR224C	YJR063W
YOR224C	YOR340C
YOR224C	YDR404C
YOR224C	YDL115C
YOR224C	YNR003C
YOR224C	YOR341W
YOR224C	YDL150W
YOR224C	YER139C
YAL048C	YOR284W
YOR284W	YML103C
YOR284W	YPL206C
YOR284W	YKL007W
YOR362C	YPR103W
YOR362C	YER021W
YOR362C	BLM3
YOR362C	YOL038W
YOR362C	YFR050C
YOR362C	YMR314W
YOR362C	YBL041W
YOR362C	YER012W
YOR362C	YLR170C
YOR362C	YJL001W
YPR103W	YDL147W
YPR103W	YGR232W
YPR103W	YML092C
YPR103W	BLM3
YPR103W	YOL038W
YPR103W	YFR050C
YPR103W	YMR314W
YPR103W	YBL041W

YPR103W	YER012W
YPR103W	YJL001W
YBR087W	YOL094C
YBR087W	YMR078C
YBR087W	YOR217W
YBR087W	YOR144C
YBR087W	YNL088W
YBR087W	YJR068W
YBR087W	YNL290W
YOL094C	YMR078C
YOL094C	YDR083W
YOL094C	YOR217W
YOL094C	YOR144C
YOL094C	YJR068W
YOL094C	YNL290W
YER025W	YPL237W
YER025W	YLR367W
YER025W	YBR048W
YER025W	YMR309C
YER025W	YLR291C
YER025W	YOR260W
YER025W	YDR211W
YER025W	YGR083C
YER025W	YJR007W
YER025W	YKR026C
YER025W	YLR215C
YPL237W	YLR291C
YPL237W	YOR260W
YPL237W	YDR211W
YPL237W	YGR083C
YPL237W	YJR007W
YPL237W	YKR026C
YPL237W	YOR101W
YJL176C	YPR034W
YJL176C	YML007W
YJL176C	YHL025W
YJL176C	YMR033W
YJL176C	YOR290C
YJL176C	YBR289W
YJL176C	YDR073W
YJL176C	YNR023W
YJL176C	YFL049W
YJL176C	YPL016W
YJL176C	YPL129W
YPR034W	YMR091C

YPR034W	YHL025W
YPR034W	YMR033W
YPR034W	YDR303C
YPR034W	YKR008W
YPR034W	YOR290C
YPR034W	YGR056W
YPR034W	YLR357W
YPR034W	YCR052W
YPR034W	YDR073W
YPR034W	YIL126W
YPR034W	YLR033W
YPR034W	YFL049W
YPR034W	YPL016W
YDR324C	YPR144C
YDR324C	YEL055C
YDR324C	YJL109C
YDR324C	YPL160W
YDR324C	YMR229C
YDR324C	YLR409C
YDR324C	YCR057C
YDR324C	YER082C
YDR324C	YMR093W
YDR324C	YPR137W
YDR324C	YGR128C
YDR324C	YJL069C
YDR324C	YNL075W
YPR144C	YJL109C
YPR144C	YKR060W
YPR144C	YNL132W
YPR144C	YPL012W
YPR144C	YDL148C
YPR144C	YBL004W
YPR144C	YCR057C
YPR144C	YER082C
YPR144C	YOR096W
YPR144C	YHR148W
YPR144C	YLR186W
YPR144C	YMR093W
YPR144C	YPR137W
YPR144C	YLR003C
YPR144C	YDL014W
YPR144C	YGR145W
YPR144C	YLR129W
YPR144C	YBR247C
YPR144C	YGR128C

YPR144C YPL217C
YPR144C YLL011W
YPR144C YNL075W
YPR144C YLR222C
YPR144C YBL072C
YPR144C YPL081W
YNR037C YOR205C
YNR037C YHL004W
YNR037C YKL155C
YNR037C YDR347W
YNR037C YGL129C
YNR037C YDR175C
YNR037C YMR188C
YNR037C YBR146W
YNR037C YGR170W
YNR037C YDR036C
YNR037C YNL137C
YNR037C YNL306W
YNR037C YBR251W
YNR037C YGR215W
YNR037C YBL090W
YNR037C YGR084C
YNR037C YOR158W
YNR037C YPL013C
YNR037C YKL003C
YNR037C YOR243C
YNR037C YMR158W
YNR037C YJR101W
YNR037C YJR113C
YNR037C YPL118W
YNR037C YNR036C
YNR037C YNL186W
YNR037C YOL115W
YNR037C YIL093C
YOR205C YHL004W
YOR205C YKL155C
YOR205C YDR347W
YOR205C YGL129C
YOR205C YDR175C
YOR205C YBR146W
YOR205C YGR170W
YOR205C YDR036C
YOR205C YNL137C
YOR205C YNL306W
YOR205C YBR251W

YOR205C	YGR215W
YOR205C	YBL090W
YOR205C	YGR084C
YOR205C	YPL013C
YOR205C	YKL003C
YOR205C	YOR243C
YOR205C	YDR517W
YOR205C	YJR101W
YOR205C	YJR113C
YOR205C	YPL118W
YOR205C	YNL186W
YOR205C	YOL115W
YOR205C	YIL093C
YCR035C	YHR069C
YCR035C	YOL021C
YCR035C	YOR076C
YCR035C	YHR081W
YCR035C	YNL232W
YCR035C	YDL111C
YCR035C	YDR280W
YCR035C	YOR001W
YCR035C	YOL142W
YCR035C	YKL126W
YHR069C	YNL189W
YHR069C	YOL021C
YHR069C	YOR076C
YHR069C	YHR081W
YHR069C	YNL232W
YHR069C	YDL111C
YHR069C	YDR280W
YHR069C	YOR001W
YHR069C	YOL142W
YHL004W	YKL155C
YHL004W	YDR347W
YHL004W	YGL129C
YHL004W	YDR175C
YHL004W	YMR188C
YHL004W	YBR146W
YHL004W	YGR170W
YHL004W	YDR036C
YHL004W	YNL137C
YHL004W	YOR204W
YHL004W	YNL306W
YHL004W	YBR251W
YHL004W	YGR215W

YHL004W	YBL090W
YHL004W	YGR084C
YHL004W	YOR158W
YHL004W	YPL013C
YHL004W	YKL003C
YHL004W	YOR243C
YHL004W	YMR158W
YHL004W	YJR101W
YHL004W	YJR113C
YHL004W	YPL118W
YHL004W	YNL186W
YHL004W	YIL093C
YKL155C	YDR347W
YKL155C	YGL129C
YKL155C	YDR175C
YKL155C	YGL180W
YKL155C	YMR188C
YKL155C	YBR146W
YKL155C	YGR170W
YKL155C	YDR036C
YKL155C	YGR165W
YKL155C	YNL137C
YKL155C	YNL306W
YKL155C	YBR251W
YKL155C	YGR215W
YKL155C	YBL090W
YKL155C	YGR084C
YKL155C	YOR158W
YKL155C	YPL013C
YKL155C	YKL003C
YKL155C	YMR158W
YKL155C	YJR101W
YKL155C	YGR061C
YKL155C	YJR113C
YKL155C	YPL118W
YKL155C	YNL186W
YKL155C	YIL093C
YCR012W	YGL137W
YCR012W	YDL145C
YCR012W	YOL147C
YCR012W	YEL031W
YCR012W	YDR216W
YCR012W	YOR070C
YCR012W	YNL006W
YCR012W	YGR279C

YCR012W	YMR173W
YCR012W	YNL192W
YGL137W	YKL081W
YGL137W	YDR238C
YGL137W	YFR051C
YGL137W	YGL100W
YGL137W	YPL235W
YGL137W	YDL055C
YGL137W	YIL076W
YGL137W	YPL010W
YGL137W	YDL145C
YGL137W	YER122C
YGL137W	YLR153C
YGL137W	YNL287W
YBR086C	YDL171C
YBR086C	YMR146C
YBR086C	YBR181C
YBR086C	YOL120C
YBR086C	YPR041W
YBR086C	YGR034W
YBR086C	YDR429C
YBR086C	YPR086W
YDL171C	YBR181C
YDL171C	YJR123W
YDL171C	YPL208W
YDL171C	YLR257W
YDL171C	YDR361C
YLR192C	YMR146C
YLR192C	YAL035W
YLR192C	YMR309C
YLR192C	YBR079C
YLR192C	YNL244C
YLR192C	YOR361C
YLR192C	YPR041W
YLR192C	YDR091C
YLR192C	YDL149W
YLR192C	YDR450W
YLR192C	YER102W
YLR192C	YDR429C
YMR146C	YMR309C
YMR146C	YBR079C
YMR146C	YNL244C
YMR146C	YOR361C
YMR146C	YPR041W
YMR146C	YNL096C

YMR146C	YDR091C
YMR146C	YDL149W
YMR146C	YGL022W
YMR146C	YDR429C
YNL189W	YOR098C
YNL189W	YNL224C
YNL189W	YOL135C
YNL189W	YLR335W
YNL189W	YNL232W
YNL189W	YPL178W
YNL189W	YLR347C
YNL189W	YPL020C
YNL189W	YDR280W
YNL189W	YOR001W
YNL189W	YAR002W
YNL189W	YMR308C
YNL189W	YKR029C
YNL189W	YOL142W
YNL189W	YER110C
YOR098C	YNL224C
YOR098C	YLR347C
YOR098C	YPL020C
YOR098C	YAR002W
YDR422C	YGL115W
YDR422C	YER027C
YDR422C	YGL208W
YDR422C	YER129W
YDR422C	YDR477W
YGL115W	YER177W
YGL115W	YDR186C
YGL115W	YER027C
YGL115W	YGL208W
YGL115W	YBR125C
YGL115W	YER129W
YGL115W	YDR477W
YGL115W	YGR152C
YNL005C	YJL063C
YNL005C	YML025C
YNL005C	YCL014W
YNL005C	YDR462W
YNL005C	YCR071C
YNL005C	YMR225C
YNL005C	YDR237W
YNL005C	YDR322W
YNL005C	YGR220C

YNL005C	YMR193W
YNL005C	YMR024W
YNL005C	YDR296W
YNL005C	YLR439W
YNL005C	YOR150W
YNL005C	YNL177C
YNL005C	YHR147C
YNL005C	YGR162W
YNL005C	YPL183W-A
YNL005C	YBR122C
YNL005C	YDR405W
YNL005C	YNL185C
YEL055C	YPL160W
YEL055C	YNL175C
YEL055C	YMR121C
YEL055C	YPL207W
YKL173W	YNL147W
YKL173W	YBL026W
YKL173W	YGL120C
YKL173W	YLR424W
YKL173W	YDL098C
YKL173W	YDR378C
YKL173W	YBL074C
YKL173W	YER029C
YKL173W	YGR091W
YKL173W	YLR117C
YKL173W	YGR074W
YKL173W	YDL087C
YKL173W	YDL209C
YKL173W	YHR165C
YKL173W	YPR182W
YKL173W	YMR213W
YKL173W	YBR152W
YKL173W	YER112W
YKL173W	YHR086W
YKL173W	YGR278W
YKL173W	YER146W
YKL173W	YPR178W
YKL173W	YPR082C
YKL173W	YAL032C
YKL173W	YJL203W
YNL147W	YBL026W
YNL147W	YDL098C
YNL147W	YDR378C
YNL147W	YJR022W

YNL147W	YCR077C
YNL147W	YHR165C
YNL147W	YDL160C
YNL147W	YER112W
YNL147W	YJL124C
YNL147W	YOR308C
YNL147W	YPR178W
YNL147W	YPR082C
YNL147W	YMR268C
YDR045C	YNL151C
YDR045C	YNL113W
YDR045C	YKR025W
YDR045C	YOR116C
YDR045C	YDR005C
YDR045C	YJL011C
YDR045C	YNR003C
YDR045C	YDL150W
YDR045C	YOR207C
YNL151C	YNL113W
YNL151C	YKR025W
YNL151C	YOR116C
YNL151C	YGL156W
YNL151C	YPR032W
YNL151C	YDR005C
YNL151C	YJL011C
YNL151C	YNR003C
YNL151C	YDL150W
YNL151C	YOR207C
YJL063C	YML025C
YJL063C	YCL014W
YJL063C	YDR462W
YJL063C	YCR071C
YJL063C	YMR064W
YJL063C	YJL041W
YJL063C	YMR225C
YJL063C	YDR237W
YJL063C	YDR322W
YJL063C	YGR220C
YJL063C	YMR193W
YJL063C	YMR024W
YJL063C	YDR296W
YJL063C	YLR439W
YJL063C	YOR150W
YJL063C	YNL177C
YJL063C	YHR147C

YJL063C	YPL183W-A
YJL063C	YBR122C
YJL063C	YDR405W
YJR112W	YPL233W
YJR112W	YAL034W-A
YHR099W	YPR070W
YHR099W	YOR244W
YHR099W	YLR071C
YHR099W	YLR055C
YHR099W	YML007W
YHR099W	YDR448W
YHR099W	YGL066W
YHR099W	YMR236W
YHR099W	YFL024C
YHR099W	YJL081C
YHR099W	YPR023C
YHR099W	YDR145W
YHR099W	YPL254W
YPR070W	YBR193C
YPR070W	YLR071C
YPR070W	YER022W
YPR070W	YNL025C
YPR070W	YML007W
YPR070W	YMR112C
YPR070W	YOL135C
YPR070W	YGL151W
YPR070W	YHR058C
YPR070W	YOL051W
YPR070W	YGL025C
YPR070W	YBR253W
YPR070W	YHR041C
YPR070W	YDR448W
YPR070W	YGL112C
YPR070W	YGR252W
YPR070W	YDR308C
YPR070W	YPL042C
YPR070W	YCR081W
YPR070W	YDR443C
YPR070W	YGR104C
YPR070W	YPR168W
YPR070W	YNR010W
YPR070W	YOR174W
YBL026W	YDR378C
YBL026W	YER029C
YBL026W	YGR091W

YBL026W	YHR156C
YBL026W	YCR077C
YBL026W	YHR165C
YBL026W	YDL160C
YBL026W	YER112W
YBL026W	YJL124C
YBL026W	YOR308C
YBL026W	YER146W
YBL026W	YPR178W
YBL026W	YGR075C
YBL026W	YLR438C-A
YLR074C	YLR325C
YLR074C	YNL002C
YLR074C	YGR103W
YLR074C	YHR197W
YLR074C	YNL182C
YLR074C	YDR101C
YLR074C	YGR245C
YLR074C	YOL127W
YLR074C	YPR016C
YLR074C	YGL103W
YLR074C	YPL093W
YLR074C	YGL030W
YLR074C	YGL029W
YLR325C	YBR009C
YLR325C	YOL054W
YLR325C	YGR245C
YLR325C	YDL070W
YBL046W	YDR075W
YBL046W	YDL112W
YBL046W	YML010W
YBL046W	YDR379W
YBL046W	YNL201C
YDR075W	YGR192C
YDR075W	YML010W
YDR075W	YIL153W
YDR075W	YDR379W
YDR075W	YNL201C
YMR072W	YOL006C
YMR072W	YDR303C
YMR072W	YPL082C
YMR072W	YCR052W
YMR072W	YBR198C
YMR072W	YDR190C
YMR072W	YOL004W

YMR072W	YGR017W
YMR072W	YBL103C
YMR072W	YIL056W
YMR072W	YCR084C
YMR072W	YGL013C
YMR072W	YOL067C
YML025C	YCL014W
YML025C	YDR462W
YML025C	YCR071C
YML025C	YJL041W
YML025C	YDR237W
YML025C	YDR322W
YML025C	YGR220C
YML025C	YMR193W
YML025C	YMR024W
YML025C	YDR296W
YML025C	YLR439W
YML025C	YOR150W
YML025C	YNL177C
YML025C	YHR147C
YML025C	YPL183W-A
YML025C	YBR122C
YML025C	YDR405W
YML025C	YNL185C
YAL047C	YLR212C
YAL047C	YHR172W
YAL047C	YNL126W
YLR212C	YHR172W
YLR212C	YNL126W
YGL147C	YHL019C
YGL147C	YFR001W
YGL147C	YBR084C-A
YGL147C	YPR016C
YHL019C	YBR084C-A
YHL019C	YLR170C
YHL019C	YPR029C
YHL019C	YPL259C
YHL019C	YBL027W
YBR049C	YOL006C
YBR049C	YBR009C
YBR049C	YDR303C
YBR049C	YPL082C
YBR049C	YLR357W
YBR049C	YGR047C
YBR049C	YGL234W

YBR049C	YIL056W
YBR049C	YMR284W
YBR049C	YLR176C
YOL006C	YGL234W
YOL006C	YBL002W
YCL014W	YDR462W
YCL014W	YCR071C
YCL014W	YMR225C
YCL014W	YDR237W
YCL014W	YDR322W
YCL014W	YLR371W
YCL014W	YGR220C
YCL014W	YMR193W
YCL014W	YDR296W
YCL014W	YLR439W
YCL014W	YOR150W
YCL014W	YHR147C
YCL014W	YPL183W-A
YCL014W	YCR030C
YCL014W	YBR122C
YCL014W	YDR405W
YDR462W	YCR071C
YDR462W	YMR064W
YDR462W	YMR225C
YDR462W	YOR201C
YDR462W	YDR237W
YDR462W	YDR322W
YDR462W	YGR220C
YDR462W	YNL175C
YDR462W	YMR193W
YDR462W	YMR024W
YDR462W	YDR296W
YDR462W	YLR439W
YDR462W	YOR150W
YDR462W	YNL177C
YDR462W	YHR147C
YDR462W	YPL183W-A
YDR462W	YBR122C
YNL113W	YPR010C
YNL113W	YKR025W
YNL113W	YOR116C
YNL113W	YDR005C
YNL113W	YJL011C
YNL113W	YDL150W
YNL113W	YOR207C

YPR010C	YGL245W
YPR010C	YBR154C
YPR010C	YJR063W
YPR010C	YFR030W
YPR010C	YOR340C
YPR010C	YDR211W
YPR010C	YKL067W
YPR010C	YNL248C
YPR010C	YDR171W
YPR010C	YOR341W
YPR010C	YDR214W
YBR181C	YKL045W
YBR181C	YIR008C
YBR181C	YPR041W
YBR181C	YNL102W
YBR181C	YGR034W
YBR181C	YBR084C-A
YBR181C	YDL058W
YBR181C	YOR217W
YBR181C	YDL191W
YBR181C	YDR429C
YBR181C	YBL035C
YKL045W	YNR052C
YKL045W	YIR008C
YKL045W	YNL102W
YKL045W	YDR292C
YKL045W	YBL035C
YGL120C	YLR424W
YGL120C	YNL224C
YGL120C	YMR230W
YGL120C	YER029C
YGL120C	YLR117C
YGL120C	YJL190C
YGL120C	YHR165C
YGL120C	YMR213W
YGL120C	YDL014W
YGL120C	YCL011C
YGL120C	YPL151C
YGL120C	YAL032C
YGL120C	YCL037C
YGL120C	YGR280C
YLR424W	YER029C
YLR424W	YLR117C
YLR424W	YCR063W
YLR424W	YHR156C

YLR424W	YJR084W
YLR424W	YDL209C
YLR424W	YHR165C
YLR424W	YBL104C
YLR424W	YPL151C
YLR424W	YKR022C
YOR244W	YML007W
YOR244W	YFL024C
YOR244W	YJL081C
YOR244W	YPR023C
YOR244W	YEL018W
YOR244W	YDR359C
YOR244W	YNL107W
YDR427W	YIL075C
YDR427W	YER021W
YDR427W	YHL030W
YDR427W	YLR421C
YDR427W	YFR004W
YDR427W	YFR052W
YDR427W	YDR127W
YDR427W	YPR108W
YDR427W	YDL147W
YDR427W	YFR010W
YDR427W	YGR232W
YDR427W	YOL038W
YDR427W	YKL145W
YDR427W	YMR314W
YDR427W	YDL007W
YDR427W	YGL048C
YDR427W	YDR394W
YDR427W	YOR117W
YIL075C	YKL081W
YIL075C	YHL030W
YIL075C	YLR421C
YIL075C	YFR004W
YIL075C	YFR052W
YIL075C	YPR108W
YIL075C	YDL147W
YIL075C	YFR010W
YIL075C	YGR232W
YIL075C	YOL038W
YIL075C	YKL145W
YIL075C	YJL001W
YIL075C	YNL075W
YBR193C	YLR071C

YBR193C	YER022W
YBR193C	YNL025C
YBR193C	YMR112C
YBR193C	YOL135C
YBR193C	YGL151W
YBR193C	YHR058C
YBR193C	YOL051W
YBR193C	YGL025C
YBR193C	YBR253W
YBR193C	YHR041C
YBR193C	YDR308C
YBR193C	YPL042C
YBR193C	YCR081W
YBR193C	YDR443C
YBR193C	YGR104C
YBR193C	YPR168W
YBR193C	YNR010W
YLR071C	YER022W
YLR071C	YNL025C
YLR071C	YMR112C
YLR071C	YOL135C
YLR071C	YGL151W
YLR071C	YHR058C
YLR071C	YOL051W
YLR071C	YGL025C
YLR071C	YBR253W
YLR071C	YHR041C
YLR071C	YDR308C
YLR071C	YPL042C
YLR071C	YCR081W
YLR071C	YDR443C
YLR071C	YGR104C
YLR071C	YPR168W
YLR071C	YNR010W
YLR071C	YOR174W
YJL109C	YPL043W
YJL109C	YDL213C
YJL109C	YLR409C
YJL109C	YBL004W
YJL109C	YCR057C
YJL109C	YER082C
YJL109C	YHR148W
YJL109C	YLR186W
YJL109C	YDR365C
YJL109C	YOL010W

YJL109C	YMR093W
YJL109C	YPR137W
YJL109C	YJR002W
YJL109C	YDL014W
YJL109C	YGR128C
YJL109C	YDR280W
YJL109C	YPL217C
YJL109C	YLL011W
YJL109C	YJL069C
YJL109C	YNL075W
YJL109C	YLR222C
YLR200W	YML094W
YLR200W	YGR078C
YLR200W	YNL153C
YML094W	YGR078C
YML094W	YIL125W
YML094W	YNL153C
YDR434W	YGR192C
YDR434W	YML032C
YDR434W	YDR331W
YDR434W	YHR188C
YDR434W	YKL039W
YDR434W	YLR088W
YGR192C	YKL039W
YGR192C	YBL013W
YGR192C	YBL113C
YJL006C	YML112W
YJL006C	YKL139W
YJL006C	YBR169C
YML112W	YKL139W
YML112W	YBR169C
YDR347W	YGL129C
YDR347W	YDR175C
YDR347W	YGL180W
YDR347W	YMR188C
YDR347W	YBR146W
YDR347W	YDR036C
YDR347W	YNL137C
YDR347W	YNL306W
YDR347W	YBR251W
YDR347W	YGR215W
YDR347W	YBL090W
YDR347W	YGR084C
YDR347W	YOR158W
YDR347W	YPL013C

YDR347WYKL003C
YDR347WYMR158W
YDR347WYJR101W
YDR347WYJR113C
YDR347WYPL118W
YDR347WYIL093C
YGL129C YDR175C
YGL129C YGL180W
YGL129C YMR188C
YGL129C YBR146W
YGL129C YGR170W
YGL129C YDR036C
YGL129C YGR165W
YGL129C YNL137C
YGL129C YNL306W
YGL129C YBR251W
YGL129C YGR215W
YGL129C YBL090W
YGL129C YGR084C
YGL129C YOR158W
YGL129C YPL013C
YGL129C YKL003C
YGL129C YMR158W
YGL129C YJR101W
YGL129C YGR061C
YGL129C YJR113C
YGL129C YPL118W
YGL129C YNL186W
YGL129C YIL093C
YER022W YNL025C
YER022W YMR112C
YER022W YOL135C
YER022W YGL151W
YER022W YHR058C
YER022W YOL051W
YER022W YGL025C
YER022W YBR253W
YER022W YHR041C
YER022W YDR308C
YER022W YPL042C
YER022W YCR081W
YER022W YDR443C
YER022W YGR104C
YER022W YPR168W
YER022W YNR010W

YER022W	YOR174W
YNL025C	YML007W
YNL025C	YMR112C
YNL025C	YOL135C
YNL025C	YGL151W
YNL025C	YHR058C
YNL025C	YOL051W
YNL025C	YGL025C
YNL025C	YKR095W
YNL025C	YBR253W
YNL025C	YER165W
YNL025C	YHR041C
YNL025C	YGR252W
YNL025C	YDR308C
YNL025C	YPL042C
YNL025C	YCR081W
YNL025C	YDR443C
YNL025C	YGR104C
YNL025C	YPR168W
YNL025C	YNR010W
YNL025C	YOR174W
YEL036C	YGR231C
YEL036C	YDR245W
YEL036C	YGR132C
YEL036C	YJL183W
YEL036C	YPL094C
YEL036C	YJR075W
YEL036C	YKL039W
YEL036C	YPL050C
YEL036C	YCR043C
YEL036C	YLR342W
YGR231C	YEL022W
YGR231C	YDR525W-A
YGR231C	YGR132C
YGR231C	YJL183W
YGR231C	YER017C
YGR231C	YMR089C
YGR231C	YJR075W
YGR231C	YPL050C
YPL086C	YPL101W
YPL086C	YGR200C
YPL086C	YPL204W
YPL086C	YKL164C
YPL086C	YHR187W
YPL101W	YGR200C

YPL101W	YHR187W
YEL015W	YOL149W
YEL015W	YML091C
YEL015W	YNL118C
YEL015W	YBR094W
YOL149W	YML091C
YOL149W	YNL118C
YOL149W	YBR094W
YIR002C	YBR114W
YIR002C	YAR007C
YIR002C	YNL312W
YIR002C	YCR092C
YIR002C	YML032C
YIR002C	YDR499W
YIR002C	YDL156W
YIR002C	YHR164C
YIR002C	YDL225W
YIR002C	YOL090W
YKR025W	YOR116C
YKR025W	YJL011C
YKR025W	YNR003C
YKR025W	YDL150W
YKR025W	YOR207C
YOR116C	YPR187W
YOR116C	YBR154C
YOR116C	YDR005C
YOR116C	YJL011C
YOR116C	YNR003C
YOR116C	YKL213C
YOR116C	YDL150W
YOR116C	YOR207C
YOL021C	YOR076C
YOL021C	YHR081W
YOL021C	YNL232W
YOL021C	YDL111C
YOL021C	YDR280W
YOL021C	YOR001W
YOL021C	YOL142W
YOL021C	YPR055W
YOR076C	YHR081W
YOR076C	YNL232W
YOR076C	YDL111C
YOR076C	YDR280W
YOR076C	YOR001W
YOR076C	YOL142W

YKR060W	YCR057C
YKR060W	YLR186W
YKR060W	YDR365C
YKR060W	YLR003C
YKR060W	YGR145W
YKR060W	YNR043W
YKR060W	YLL011W
YKR060W	YDL166C
YKR060W	YCL031C
YJL207C	YOR299W
YJL207C	YDR495C
YOR299W	YKR027W
YOR299W	YLR330W
YOR299W	YJL099W
YOR299W	YMR237W
YOR299W	YDR495C
YML073C	YFR031C-A
YML073C	YER036C
YDL098C	YER029C
YDL098C	YBL104C
YDL098C	YBR152W
YDL098C	YER112W
YDL098C	YPR178W
YDL098C	YMR268C
YDL098C	YGR075C
YER021W	YHL030W
YER021W	YLR421C
YER021W	YFR004W
YER021W	YFR052W
YER021W	YPR108W
YER021W	YDL147W
YER021W	YFR010W
YER021W	YGR232W
YER021W	YOL038W
YER021W	YKL145W
YER021W	YJL001W
YPL160W	YPL111W
YCR042C	YKL081W
YCR042C	YGL112C
YCR042C	YBR198C
YCR042C	YML015C
YCR042C	YMR227C
YCR042C	YPL129W
YCR042C	YML114C
YKL081W	YBR025C

YKL081W	YAL003W
YKL081W	YGR240C
YKL081W	YPL226W
YKL081W	YBR118W
YDR175C	YGL180W
YDR175C	YMR188C
YDR175C	YBR146W
YDR175C	YGR170W
YDR175C	YDR036C
YDR175C	YGR165W
YDR175C	YNL137C
YDR175C	YNL306W
YDR175C	YBR251W
YDR175C	YGR215W
YDR175C	YBL090W
YDR175C	YGR084C
YDR175C	YOR158W
YDR175C	YPL013C
YDR175C	YKL003C
YDR175C	YMR158W
YDR175C	YJR101W
YDR175C	YGR061C
YDR175C	YJR113C
YDR175C	YPL118W
YDR175C	YNL186W
YDR175C	YIL093C
YGL180W	YGR165W
YGL180W	YNL137C
YGL180W	YBR251W
YGL180W	YGR084C
YGL180W	YOR158W
YGL180W	YGR061C
YFR031C-A	YNL112W
YFR031C-A	YLR367W
YFR031C-A	YAL035W
YFR031C-A	YDR418W
YFR031C-A	YGL031C
YFR031C-A	YNL301C
YFR031C-A	YBR263W
YFR031C-A	YPL009C
YFR031C-A	YIL018W
YFR031C-A	YOL127W
YFR031C-A	YGL103W
YFR031C-A	YDL082W
YFR031C-A	YHR010W

YFR031C-A	YNL069C
YFR031C-A	YPR175W
YFR031C-A	YPL143W
YFR031C-A	YJL191W
YFR031C-A	YGR085C
YFR031C-A	YMR142C
YNL112W	YBR263W
YNL112W	YMR192W
YDR001C	YGL252C
YDR001C	YER177W
YDR001C	YDR099W
YDR001C	YAL017W
YGL252C	YER177W
YGL252C	YLR333C
YGL252C	YGL207W
YGL252C	YDR099W
YLR367W	YNL132W
YLR367W	YAL035W
YLR367W	YLR196W
YLR367W	YNL175C
YLR367W	YDR091C
YLR367W	YOR056C
YNL132W	YPL043W
YNL132W	YMR143W
YNL132W	YLR333C
YNL132W	YDL148C
YNL132W	YER082C
YNL132W	YOR096W
YNL132W	YPR137W
YNL132W	YKL101W
YNL132W	YGR145W
YNL132W	YBR247C
YNL132W	YNL308C
YNL132W	YPL081W
YNL132W	YMR194W
YHL030W	YLR421C
YHL030W	YFR004W
YHL030W	YFR052W
YHL030W	YPR108W
YHL030W	YDL147W
YHL030W	YFR010W
YHL030W	YML092C
YHL030W	YOL038W
YHL030W	YKL145W
YHL030W	YDL007W

YHL030W	YGL048C
YHL030W	YDR394W
YHL030W	YJL001W
YHL030W	YJR077C
YHL030W	YOR117W
YHL030W	YBR017C
YDR025W	YNL224C
YDR025W	YMR309C
YDR025W	YPR041W
YDR025W	YGR285C
YDR025W	YPL204W
YDR025W	YDR228C
YDR025W	YMR110C
YNL224C	YMR230W
YNL224C	YLR335W
YNL224C	YPL020C
YNL224C	YAR002W
YMR188C	YBR146W
YMR188C	YDR036C
YMR188C	YGR165W
YMR188C	YNL137C
YMR188C	YNL306W
YMR188C	YBR251W
YMR188C	YGR215W
YMR188C	YBL090W
YMR188C	YGR084C
YMR188C	YOR158W
YMR188C	YPL013C
YMR188C	YKL003C
YMR188C	YMR158W
YMR188C	YJR101W
YMR188C	YJR113C
YMR188C	YPL118W
YMR188C	YIL093C
YLR055C	YDR176W
YLR055C	YDR448W
YLR055C	YGL066W
YLR055C	YGL112C
YLR055C	YMR236W
YLR055C	YGR252W
YLR055C	YBR198C
YLR055C	YDR145W
YLR055C	YPL254W
YLR055C	YMR223W
YLR055C	YCL010C

YLR055C	YBR081C
YLR055C	YKL078W
YBR146W	YGR170W
YBR146W	YDR036C
YBR146W	YNL137C
YBR146W	YNL306W
YBR146W	YBR251W
YBR146W	YGR215W
YBR146W	YBL090W
YBR146W	YGR084C
YBR146W	YOR158W
YBR146W	YPL013C
YBR146W	YKL003C
YBR146W	YOR243C
YBR146W	YMR158W
YBR146W	YJR101W
YBR146W	YJR113C
YBR146W	YPL118W
YBR146W	YNL186W
YBR146W	YIL093C
YGR170W	YDR036C
YGR170W	YMR176W
YGR170W	YPL001W
YGR170W	YBR009C
YGR170W	YNL306W
YGR170W	YBR251W
YGR170W	YGR215W
YGR170W	YOL054W
YGR170W	YBL090W
YGR170W	YGR084C
YGR170W	YDR300C
YGR170W	YPL013C
YGR170W	YKL003C
YGR170W	YOR243C
YGR170W	YDL070W
YGR170W	YDL058W
YGR170W	YJR101W
YGR170W	YJR113C
YGR170W	YPL118W
YGR170W	YNL186W
YGR170W	YOL115W
YGR170W	YIL093C
YGR170W	YFR006W
YGR170W	YJR132W
YDR176W	YDR448W

YDR176WYGL066W
YDR176WYGL112C
YDR176WYMR236W
YDR176WYDR145W
YDR176WYPL254W
YDR176WYMR223W
YDR176WYCL010C
YDR176WYBR081C
YBR048W YDL148C
YBR048W YBR247C
YBR048W YOR056C
YBR048W YNL308C
YCR071C YMR064W
YCR071C YMR225C
YCR071C YOR201C
YCR071C YDR237W
YCR071C YDR322W
YCR071C YGR220C
YCR071C YMR193W
YCR071C YMR024W
YCR071C YDR296W
YCR071C YLR439W
YCR071C YOR150W
YCR071C YNL177C
YCR071C YHR147C
YCR071C YPL183W-A
YCR071C YBR122C
YCR071C YDR405W
YCR071C YIL104C
YMR064W YGR220C
YMR064W YDR296W
YMR064W YOR150W
YMR064W YNL177C
YMR064W YPL183W-A
YMR064W YBR122C
YMR064W YIL104C
YMR064W YOL123W
YLR421C YFR052W
YLR421C YPR108W
YLR421C YDL147W
YLR421C YFR010W
YLR421C YKL145W
YLR421C YDL007W
YLR421C YGL048C
YLR421C YDR394W

YLR421C	YOR117W
YLR421C	YOL087C
YLR448W	YPL043W
YLR448W	YMR061W
YLR448W	YJL033W
YLR448W	YDR195W
YPL043W	YNL002C
YPL043W	YOR206W
YPL043W	YGR103W
YPL043W	YMR049C
YPL043W	YPL012W
YPL043W	YDR060W
YPL043W	YBL004W
YPL043W	YDR496C
YPL043W	YNL175C
YPL043W	YNL061W
YPL043W	YOL127W
YPL043W	YLR175W
YPL043W	YPL093W
YPL043W	YNL110C
YPL043W	YLL008W
YPL043W	YHR010W
YPL043W	YKL014C
YPL043W	YLR276C
YPL043W	YIL133C
YPL043W	YBR153W
YLR142W	YMR315W
YLR142W	YGL245W
YMR315W	YBR114W
YMR315W	YPL154C
YMR315W	YOR332W
YCR093W	YNR052C
YCR093W	YPL235W
YCR093W	YDL149W
YCR093W	YOR110W
YCR093W	YAL021C
YCR093W	YER068W
YCR093W	YNL288W
YCR093W	YGR134W
YNR052C	YIR008C
YNR052C	YNL102W
YNR052C	YAL021C
YNR052C	YNL288W
YNR052C	YBL035C
YNR052C	YGR134W

YBR057C	YKR031C
YBR057C	YGL036W
YKR031C	YPL050C
YKR031C	YNL290W
YKR031C	YGL036W
YNL002C	YOR206W
YNL002C	YFL002C
YNL002C	YFR001W
YNL002C	YGR103W
YNL002C	YMR049C
YNL002C	YDR060W
YNL002C	YDR496C
YNL002C	YNL175C
YNL002C	YDR101C
YNL002C	YGR245C
YNL002C	YPR016C
YNL002C	YNL110C
YDR036C	YNL137C
YDR036C	YNL306W
YDR036C	YBR251W
YDR036C	YGR215W
YDR036C	YBL090W
YDR036C	YGR084C
YDR036C	YOR158W
YDR036C	YPL013C
YDR036C	YKL003C
YDR036C	YOR243C
YDR036C	YMR158W
YDR036C	YJR101W
YDR036C	YJR113C
YDR036C	YPL118W
YDR036C	YNL186W
YDR036C	YIL093C
YGR005C	YOL005C
YGR005C	YJL140W
YGR005C	YOR151C
YGR005C	YPR187W
YGR005C	YGR063C
YGR005C	YPR180W
YGR005C	YML010W
YGR005C	YBR154C
YGR005C	YDR404C
YGR005C	YGR186W
YGR005C	YDL140C
YGR005C	YPR093C

YGR005C	YGL070C
YGR005C	YDL115C
YGR005C	YPL129W
YOL005C	YJL140W
YOL005C	YOR151C
YOL005C	YGR063C
YOL005C	YPR180W
YOL005C	YDR404C
YOL005C	YGR186W
YOL005C	YPR093C
YOL005C	YGL070C
YOL005C	YDL115C
YER177W	YPR049C
YER177W	YLR028C
YER177W	YER133W
YER177W	YKR091W
YER177W	YDR099W
YER177W	YDL117W
YML007W	YOL135C
YML007W	YGL151W
YML007W	YHR058C
YML007W	YOL051W
YML007W	YGL025C
YML007W	YBR253W
YML007W	YHR041C
YML007W	YDR448W
YML007W	YOR290C
YML007W	YGL112C
YML007W	YFL024C
YML007W	YJL081C
YML007W	YPL042C
YML007W	YBR289W
YML007W	YPR168W
YML007W	YNR023W
YML007W	YPL254W
YML007W	YCL010C
YML007W	YDR359C
YML007W	YBR081C
YBR229C	YML067C
YBR229C	YFR051C
YBR229C	YAL042W
YBR229C	YIL076W
YBR229C	YDR221W
YML067C	YML130C
YML067C	YDR238C

YML067C	YFR051C
YML067C	YAL042W
YML067C	YIL076W
YML067C	YDL145C
YML067C	YLR342W
YFL001W	YKL060C
YKL060C	YOL147C
YKL060C	YMR113W
YMR112C	YOL135C
YMR112C	YGL151W
YMR112C	YHR058C
YMR112C	YOL051W
YMR112C	YGL025C
YMR112C	YBR253W
YMR112C	YHR041C
YMR112C	YDR308C
YMR112C	YPL042C
YMR112C	YDR443C
YMR112C	YGR104C
YMR112C	YPR168W
YMR112C	YNR010W
YMR112C	YOR174W
YOL135C	YGL151W
YOL135C	YHR058C
YOL135C	YOL051W
YOL135C	YGL025C
YOL135C	YKR095W
YOL135C	YBR253W
YOL135C	YHR041C
YOL135C	YDR448W
YOL135C	YGL112C
YOL135C	YDR308C
YOL135C	YPL042C
YOL135C	YCR081W
YOL135C	YDR443C
YOL135C	YGR104C
YOL135C	YPR168W
YOL135C	YNR010W
YOL135C	YOR174W
YOL135C	YFR019W
YAL035W	YDR418W
YAL035W	YMR309C
YAL035W	YOR361C
YAL035W	YJL190C
YAL035W	YDR496C

YAL035W	YPR041W
YAL035W	YDR091C
YAL035W	YLR175W
YAL035W	YDL014W
YAL035W	YDL082W
YAL035W	YHR010W
YAL035W	YDR429C
YAL035W	YHL033C
YDR418W	YGR285C
YIR008C	YNL102W
YIR008C	YDR292C
YIR008C	YBL035C
YDR378C	YGR091W
YDR378C	YCR077C
YDR378C	YHR165C
YDR378C	YDL160C
YDR378C	YBR152W
YDR378C	YER112W
YDR378C	YJL124C
YDR378C	YLR147C
YDR378C	YOR308C
YDR378C	YER146W
YDR378C	YPR178W
YDR378C	YPR082C
YDR378C	YLR438C-A
YGL031C	YGR214W
YOR206W	YFL002C
YOR206W	YFR001W
YOR206W	YGR103W
YOR206W	YMR229C
YOR206W	YMR049C
YOR206W	YLL034C
YOR206W	YLR449W
YOR206W	YDR060W
YOR206W	YNL061W
YOR206W	YKL009W
YOR206W	YGL111W
YOR206W	YPR016C
YOR206W	YBL087C
YOR206W	YPL093W
YOR206W	YDL031W
YOR206W	YIL133C
YFL002C	YFR001W
YFL002C	YMR049C
YFL002C	YPL211W

YFL002C	YLR449W
YFL002C	YDR496C
YFL002C	YHR197W
YFL002C	YNL061W
YFL002C	YDR087C
YFL002C	YOL127W
YFL002C	YKL172W
YFL002C	YNL110C
YFL002C	YHR010W
YFL002C	YPL220W
YFR001W	YGR103W
YFR001W	YMR049C
YFR001W	YPL211W
YFR001W	YNL182C
YFR001W	YNL061W
YFR001W	YKL009W
YFR001W	YBR084C-A
YFR001W	YPR016C
YFR001W	YPL093W
YFR001W	YLL008W
YFR001W	YDL031W
YFR001W	YIL133C
YPL166W	YPR049C
YPL166W	YLR423C
YPR049C	YDR320C
YPR049C	YLR423C
YGL151W	YHR058C
YGL151W	YOL051W
YGL151W	YBR253W
YGL151W	YHR041C
YGL151W	YDR308C
YGL151W	YCR081W
YGL151W	YDR443C
YGL151W	YGR104C
YGL151W	YNR010W
YGL151W	YPL254W
YGL151W	YOR174W
YHR058C	YOL051W
YHR058C	YGL025C
YHR058C	YBR253W
YHR058C	YDR308C
YHR058C	YPL042C
YHR058C	YDR443C
YHR058C	YGR104C
YHR058C	YPR168W

YHR058C YNR010W
YMR143W YML130C
YMR143W YLR333C
YMR143W YGR034W
YMR143W YDR365C
YMR143W YJL033W
YMR143W YKL101W
YMR143W YGR145W
YMR143W YKL067W
YMR143W YNR043W
YMR143W YHR169W
YMR143W YOR173W
YMR143W YDR460W
YMR143W YMR194W
YDL213C YHR089C
YDL213C YML130C
YDL213C YBL004W
YDL213C YOR096W
YDL213C YBR084C-A
YDL213C YDR365C
YDL213C YOL010W
YDL213C YJL033W
YDL213C YOR270C
YDL213C YKL119C
YDL213C YPL217C
YDL213C YDR516C
YDL213C YNR043W
YDL213C YHR169W
YDL213C YER102W
YGR081C YLL021W
YGR081C YPL012W
YGR081C YJR145C
YGR081C YPL020C
YGR081C YBR247C
YGR081C YKL143W
YGR081C YOR056C
YGR081C YMR308C
YGR081C YIL069C
YLL021W YML092C
YLL021W YPL020C
YLL021W YGL197W
YLL021W YLR276C
YLL021W YMR308C
YLL021W YER178W
YLL021W YMR310C

YHL015W	YMR230W
YHL015W	YAL036C
YHL015W	YNL308C
YMR230W	YNL244C
YMR230W	YAL036C
YMR230W	YGR162W
YMR230W	YHL034C
YMR230W	YPR112C
YKL018W	YMR061W
YKL018W	YLR277C
YKL018W	YNL317W
YKL018W	YAL043C
YKL018W	YOR179C
YKL018W	YER133W
YKL018W	YDR195W
YKL018W	YHR119W
YKL018W	YPL138C
YKL018W	YKR002W
YKL018W	YKL059C
YKL018W	YAR003W
YKL018W	YDR301W
YKL018W	YLR115W
YMR061W	YLR277C
YMR061W	YNL317W
YMR061W	YAL043C
YMR061W	YOR250C
YMR061W	YDR195W
YMR061W	YDL082W
YMR061W	YKR002W
YMR061W	YKL059C
YMR061W	YDR301W
YMR061W	YDR228C
YMR061W	YLR115W
YMR061W	YGL044C
YLR277C	YNL317W
YLR277C	YAL043C
YLR277C	YER133W
YLR277C	YDR195W
YLR277C	YKR002W
YLR277C	YKL059C
YLR277C	YDR301W
YLR277C	YDR228C
YLR277C	YLR115W
YNL317W	YAL043C
YNL317W	YER133W

YNL317W	YDR195W
YNL317W	YKR002W
YNL317W	YGR156W
YNL317W	YKL059C
YNL317W	YDR301W
YNL317W	YLR115W
YMR091C	YMR033W
YMR091C	YDR303C
YMR091C	YKR008W
YMR091C	YGR056W
YMR091C	YLR357W
YMR091C	YCR052W
YMR091C	YIL126W
YMR091C	YLR033W
YIL070C	YOR326W
YIL070C	YIL094C
YIL070C	YJL115W
YIL070C	YJR140C
YOR326W	YHR023W
YOR326W	YGL106W
YOR326W	YDR073W
YOR326W	YOR035C
YOR326W	YER083C
YOR326W	YBR109C
YOR326W	YJR132W
YGR165W	YNL137C
YGR165W	YBR251W
YGR165W	YBL090W
YGR165W	YGR084C
YGR165W	YOR158W
YGR165W	YPL013C
YGR165W	YKL003C
YGR165W	YMR158W
YGR165W	YGR061C
YGR165W	YNL271C
YNL137C	YNL306W
YNL137C	YBR251W
YNL137C	YGR215W
YNL137C	YBL090W
YNL137C	YGR084C
YNL137C	YOR158W
YNL137C	YPL013C
YNL137C	YKL003C
YNL137C	YMR158W
YNL137C	YJR101W

YNL137C	YJR113C
YNL137C	YPL118W
YNL137C	YIL093C
YDR364C	YDR416W
YDR364C	YCR063W
YDR364C	YER013W
YDR364C	YPR101W
YDR364C	YDL209C
YDR364C	YBR065C
YDR364C	YJR050W
YDR364C	YMR213W
YDR364C	YGR278W
YDR364C	YPL151C
YDR364C	YKL095W
YDR364C	YAL032C
YJL041W	YDR322W
YJL041W	YMR193W
YJL041W	YMR024W
YJL041W	YJL061W
YJL041W	YGR119C
YJL041W	YIL115C
YJL041W	YFR002W
YJL041W	YGL172W
YBL074C	YER029C
YBL074C	YCR063W
YBL074C	YHR156C
YBL074C	YHR165C
YBL074C	YOR319W
YBL074C	YBL104C
YER029C	YGR091W
YER029C	YDR416W
YER029C	YLR117C
YER029C	YGR074W
YER029C	YMR125W
YER029C	YDL087C
YER029C	YPL178W
YER029C	YGL100W
YER029C	YPR101W
YER029C	YMR240C
YER029C	YPL213W
YER029C	YGR162W
YER029C	YJR084W
YER029C	YHR165C
YER029C	YIR009W
YER029C	YPR182W

YER029C	YDL043C
YER029C	YOR319W
YER029C	YBL104C
YER029C	YBR152W
YER029C	YER112W
YER029C	YBR119W
YER029C	YML046W
YER029C	YHR086W
YER029C	YDR235W
YER029C	YLR298C
YER029C	YPR178W
YER029C	YKL012W
YER029C	YPR082C
YER029C	YIL061C
YER029C	YJL203W
YER029C	YDL030W
YGR250C	YNL138W
YGR250C	YER165W
YGR250C	YIR001C
YNL138W	YGR233C
YNL138W	YIR001C
YNL138W	YOL001W
YOR145C	YPL266W
YOR145C	YCR057C
YOR145C	YLR003C
YOR145C	YBR247C
YOR145C	YKL143W
YOR145C	YGR054W
YOR145C	YCL031C
YJR065C	YBR234C
YJR065C	YIL062C
YJR065C	YLR370C
YJR065C	YNR035C
YJR065C	YKL013C
YJR065C	YDL029W
YMR309C	YOR039W
YMR309C	YBR079C
YMR309C	YOR361C
YMR309C	YJL190C
YMR309C	YPR041W
YMR309C	YDR091C
YMR309C	YGL019W
YMR309C	YER102W
YMR309C	YDR429C
YMR309C	YPL081W

YOR039W	YBR079C
YOR039W	YIL035C
YOR039W	YOR361C
YOR039W	YGL207W
YOR039W	YMR172W
YOR039W	YGL019W
YOR039W	YLR418C
YOR039W	YKL088W
YOR039W	YOR061W
YOR039W	YER103W
YOR039W	YER164W
YOR039W	YOL145C
YOR039W	YDR429C
YOR039W	YML069W
YML109W	YMR273C
YML109W	YOR349W
YML109W	YDL188C
YML109W	YGL190C
YML109W	YAL016W
YML109W	YOR014W
YMR273C	YOR349W
YMR273C	YBR103W
YMR273C	YIL112W
YMR273C	YDL188C
YMR273C	YGL190C
YMR273C	YBL104C
YMR273C	YCR033W
YMR273C	YAL016W
YMR273C	YDL134C
YMR273C	YGR281W
YMR273C	YKR029C
YMR273C	YOR014W
YMR273C	YGL194C
YMR273C	YAL024C
YPL139C	YPL181W
YPL139C	YIL084C
YPL139C	YAL013W
YPL139C	YDR207C
YPL139C	YNL330C
YPL139C	YBR095C
YPL139C	YMR263W
YPL139C	YOL004W
YPL139C	YPR023C
YPL139C	YMR075W
YPL139C	YNL097C

YPL139C	YDL076C
YPL139C	YIL101C
YPL181W	YIL035C
YPL181W	YIL084C
YPL181W	YAL013W
YPL181W	YDR207C
YPL181W	YNL330C
YPL181W	YBR095C
YPL181W	YMR263W
YPL181W	YOR061W
YPL181W	YOL004W
YPL181W	YNL097C
YPL181W	YDL076C
YPL181W	YCR084C
YHR029C	YJL146W
YJL146W	YJL020C
YJL146W	YGR028W
YJL146W	YPL154C
YJL146W	YJL123C
YHR107C	YLR314C
YHR107C	YDL225W
YHR107C	YJR076C
YHR107C	YJR092W
YBR079C	YNL244C
YBR079C	YPL001W
YBR079C	YOR361C
YBR079C	YPR041W
YBR079C	YDR091C
YBR079C	YOR096W
YBR079C	YGL019W
YBR079C	YDR429C
YBR079C	YBL072C
YBR079C	YPL081W
YNL244C	YOR293W
YNL244C	YOR361C
YNL244C	YLR264W
YNL244C	YPR041W
YNL244C	YNL096C
YNL244C	YDR091C
YNL244C	YLR039C
YNL244C	YDR429C
YMR176W	YPL001W
YMR176W	YBR009C
YMR176W	YOL054W
YMR176W	YDR300C

YMR176W	YDL070W
YMR176W	YGL131C
YMR176W	YDL058W
YMR176W	YFR006W
YMR176W	YJR132W
YPL001W	YBR009C
YPL001W	YOL054W
YPL001W	YNL030W
YPL001W	YOR303W
YPL001W	YDL070W
YPL001W	YEL056W
YPL001W	YLL022C
YPL001W	YFR006W
YMR225C	YOR201C
YMR225C	YFR051C
YMR225C	YDR237W
YMR225C	YDR322W
YMR225C	YLR371W
YMR225C	YGR220C
YMR225C	YMR193W
YMR225C	YDR296W
YMR225C	YLR439W
YMR225C	YOR150W
YMR225C	YHR147C
YMR225C	YPL183W-A
YMR225C	YCR030C
YMR225C	YBR122C
YMR225C	YDR405W
YMR225C	YPL204W
YGL127C	YOL051W
YOL051W	YGL025C
YOL051W	YKR095W
YOL051W	YBR253W
YOL051W	YHR041C
YOL051W	YDR308C
YOL051W	YPL042C
YOL051W	YCR081W
YOL051W	YDR443C
YOL051W	YGR104C
YOL051W	YPR168W
YOL051W	YNR010W
YOL051W	YOR174W
YGR200C	YHR187W
YGL025C	YKR095W
YGL025C	YBR253W

YGL025C	YHR041C
YGL025C	YDR308C
YGL025C	YPL042C
YGL025C	YCR081W
YGL025C	YDR443C
YGL025C	YGR104C
YGL025C	YPR168W
YGL025C	YNR010W
YGL025C	YOR174W
YKR095W	YBR253W
YKR095W	YPL042C
YKR095W	YPR168W
YAL034C	YLR335W
YAL034C	YJR123W
YAL034C	YOL120C
YAL034C	YAR002W
YAL034C	YPR174C
YAL034C	YER110C
YAL034C	YML063W
YLR335W	YNL282W
YLR335W	YLR347C
YLR335W	YAR002W
YLR335W	YPR174C
YLR335W	YER110C
YLR335W	YGL191W
YGR103W	YMR049C
YGR103W	YDR060W
YGR103W	YHR197W
YGR103W	YDR101C
YGR103W	YIL018W
YGR103W	YGR245C
YGR103W	YGL111W
YGR103W	YPR016C
YGR103W	YPL093W
YGR103W	YNL110C
YGR103W	YLL008W
YGR103W	YGL030W
YGR103W	YDL031W
YGR103W	YOR063W
YNL301C	YOR361C
YNL301C	YDL149W
YNL301C	YER086W
YNL301C	YPL093W
YNL301C	YLL018C
YNL301C	YDR021W

YNL301C	YMR142C
YGR091W	YGR074W
YGR091W	YHR165C
YGR091W	YBR152W
YGR091W	YER112W
YGR091W	YOR308C
YGR091W	YPR178W
YGR091W	YPR082C
YDR416W	YLR117C
YDR416W	YCR063W
YDR416W	YPR101W
YDR416W	YHR156C
YDR416W	YPL213W
YDR416W	YDL209C
YDR416W	YHR165C
YDR416W	YJR050W
YDR416W	YMR213W
YDR416W	YGR278W
YDR416W	YAL032C
YDR416W	YLR132C
YLR117C	YPR101W
YLR117C	YMR240C
YLR117C	YDL209C
YLR117C	YHR165C
YLR117C	YBR065C
YLR117C	YJR050W
YLR117C	YMR213W
YLR117C	YGR278W
YLR117C	YAL032C
YKR027W	YLR330W
YKR027W	YJL099W
YKR027W	YMR237W
YLR314C	YDL225W
YLR314C	YJR076C
YLR314C	YJR092W
YLR314C	YJL060W
YMR229C	YMR049C
YMR229C	YDR060W
YMR229C	YBR126C
YMR229C	YER082C
YMR229C	YDR365C
YMR229C	YLR029C
YMR229C	YBL072C
YHL021C	YJL140W
YJL140W	YOR151C

YJL140W	YPR187W
YJL140W	YGR063C
YJL140W	YML010W
YJL140W	YDR404C
YJL140W	YGR186W
YJL140W	YGL070C
YJL140W	YDL115C
YDR311W	YKL028W
YDR311W	YNL135C
YDR311W	YLR005W
YDR311W	YPR056W
YKL028W	YGR130C
YKL028W	YLR005W
YKL028W	YER007C-A
YKL028W	YJR014W
YKL028W	YPR056W
YKL028W	YKR062W
YKL028W	YKR018C
YAL043C	YOR179C
YAL043C	YER133W
YAL043C	YDR195W
YAL043C	YKR002W
YAL043C	YGR156W
YAL043C	YKL059C
YAL043C	YDR301W
YAL043C	YLR115W
YAL043C	YNL222W
YOR179C	YER133W
YOR179C	YDR195W
YOR179C	YGR156W
YOR179C	YNL222W
YHL025W	YMR033W
YHL025W	YOR290C
YHL025W	YLR347C
YHL025W	YBR289W
YHL025W	YDR073W
YHL025W	YNR023W
YHL025W	YFL049W
YHL025W	YPL016W
YHL025W	YPL129W
YMR033W	YDR303C
YMR033W	YKR008W
YMR033W	YOR290C
YMR033W	YGR056W
YMR033W	YCR052W

YMR033W	YBR289W
YMR033W	YIL126W
YMR033W	YNR023W
YMR033W	YFL049W
YMR033W	YPL016W
YMR033W	YPL129W
YMR033W	YGR275W
YMR033W	YOL067C
YDR407C	YOR115C
YDR407C	YGR166W
YDR407C	YDR246W
YDR407C	YKR068C
YDR407C	YDR108W
YDR407C	YMR218C
YDR407C	YBR254C
YDR407C	YDR472W
YDR407C	YGR143W
YDR407C	YML077W
YOR115C	YGR166W
YOR115C	YDR246W
YOR115C	YKR068C
YOR115C	YDR108W
YOR115C	YMR218C
YOR115C	YBR254C
YOR115C	YDR472W
YOR115C	YGR143W
YOR115C	YML077W
YMR049C	YLR449W
YMR049C	YDR060W
YMR049C	YOR361C
YMR049C	YDR496C
YMR049C	YNL175C
YMR049C	YNL061W
YMR049C	YGL111W
YMR049C	YPR016C
YMR049C	YPL093W
YMR049C	YNL110C
YMR049C	YOR063W
YLR208W	YPR181C
YLR208W	YML130C
YLR208W	YDL116W
YLR208W	YGL100W
YLR208W	YGL092W
YLR208W	YDL195W
YLR208W	YIL109C

YLR208W	YPL085W
YPR181C	YJL034W
YPR181C	YNL049C
YPR181C	YDR517W
YPR181C	YDL195W
YPR181C	YIL109C
YPR181C	YPL085W
YPR181C	YHR098C
YGR074W	YMR125W
YGR074W	YPL213W
YGR074W	YHR165C
YGR074W	YER112W
YGR074W	YML046W
YGR074W	YLR147C
YGR074W	YLR298C
YGR074W	YKL012W
YGR074W	YPR057W
YGR074W	YJL203W
YGR074W	YDL030W
YBR253W	YHR041C
YBR253W	YDR448W
YBR253W	YGL112C
YBR253W	YGR252W
YBR253W	YDR308C
YBR253W	YPL042C
YBR253W	YCR081W
YBR253W	YDR443C
YBR253W	YGR104C
YBR253W	YPR168W
YBR253W	YNR010W
YBR253W	YOR174W
YOR204W	YBR251W
YOR204W	YPR041W
YOR204W	YPL013C
YJR123W	YOR361C
YJR123W	YPR041W
YJR123W	YMR093W
YJR123W	YPR137W
YJR123W	YGR285C
YJR123W	YGL234W
YJL002C	YML130C
YJL002C	YGL022W
YJL002C	YMR149W
YML130C	YGR034W
YML130C	YBR084C-A

YML130C YDR365C
YML130C YAL042W
YML130C YJL033W
YML130C YGL022W
YML130C YNR043W
YML130C YHR169W
YML130C YNL075W
YML130C YEL002C
YOR201C YFR051C
YOR201C YMR193W
YOR201C YDR296W
YOR201C YOR150W
YOR201C YHR147C
YOR201C YBR122C
YOR201C YPL204W
YLR430W YMR125W
YLR430W YER165W
YLR430W YPL178W
YLR430W YMR239C
YLR430W YPL190C
YLR430W YLR226W
YLR430W YNL251C
YLR430W YOL115W
YLR430W YPR161C
YLR430W YPR057W
YLR430W YDL175C
YLR430W YJL050W

YMR125W	YDL087C
YMR125W	YPL178W
YMR125W	YJL190C
YMR125W	YMR240C
YMR125W	YPL190C
YMR125W	YLR226W
YMR125W	YGR162W
YMR125W	YNL251C
YMR125W	YDL209C
YMR125W	YHR165C
YMR125W	YHL034C
YMR125W	YPR182W
YMR125W	YBR119W
YMR125W	YHR086W
YMR125W	YDR235W
YMR125W	YLR147C
YMR125W	YLR298C
YMR125W	YKL012W

YMR125W	YGL049C
YMR125W	YPR161C
YMR125W	YPR057W
YMR125W	YIL061C
YMR125W	YCL037C
YMR125W	YNL139C
YMR125W	YDL175C
YHR081W	YNL232W
YHR081W	YDL111C
YHR081W	YDR280W
YHR081W	YOR001W
YHR081W	YOL142W
YNL232W	YDL111C
YNL232W	YDR280W
YNL232W	YOR001W
YNL232W	YOL142W
YNL232W	YIR035C
YLR409C	YCR057C
YLR409C	YER082C
YLR409C	YMR093W
YLR409C	YJL069C
YLR409C	YLR222C
YLL034C	YPL211W
YLL034C	YLR449W
YLL034C	YDR496C
YLL034C	YDR087C
YLL034C	YNL110C
YLL034C	YMR242C
YPL211W	YDR060W
YPL211W	YDR496C
YPL211W	YNL175C
YPL211W	YHR197W
YPL211W	YNL061W
YPL211W	YDR101C
YPL211W	YGL111W
YPL211W	YBL087C
YPL211W	YPL093W
YDL112W	YGR026W
YDL112W	YPL218W
YDL112W	YDR341C
YDL112W	YJR045C
YDL112W	YHR128W
YDL112W	YJL052W
YDL112W	YKL182W
YDL112W	YKL067W

YDL112W	YLL018C
YDL112W	YML072C
YGR026W	YBR205W
YGR026W	YML072C
YDR484W	YJL034W
YDR484W	YDR027C
YDR484W	YJL029C
YJL034W	YMR214W
YJL034W	YBR139W
YJL034W	YDR027C
YJL034W	YJL029C
YJL034W	YGR205W
YGL244W	YOR123C
YGL244W	YIL035C
YGL244W	YBR279W
YGL244W	YLR418C
YGL244W	YOL145C
YOR123C	YBR279W
YOR123C	YLR418C
YOR123C	YOL145C
YLR449W	YDR496C
YLR449W	YNL061W
YLR449W	YDR101C
YLR449W	YPL093W
YLR449W	YMR242C
YLR449W	YMR121C
YLR449W	YOR063W
YBR265W	YBL023C
YBR265W	YBR126C
YBR265W	YPR035W
YBR265W	YML100W
YBR265W	YDR074W
YBR265W	YMR261C
YBL023C	YBR126C
YBL023C	YPR035W
YBL023C	YML100W
YBL023C	YDR074W
YBL023C	YMR261C
YBR009C	YDR303C
YBR009C	YOL054W
YBR009C	YGL207W
YBR009C	YFR013W
YBR009C	YDL070W
YBR009C	YPL082C
YBR009C	YLR357W

YBR009C	YEL056W
YBR009C	YER148W
YBR009C	YMR308C
YBR009C	YML069W
YBR009C	YFR006W
YDR303C	YKR008W
YDR303C	YGR056W
YDR303C	YPL082C
YDR303C	YLR357W
YDR303C	YCR052W
YDR303C	YIL126W
YDR303C	YLR033W
YDR303C	YDR225W
YGL156W	YPR032W
YGL156W	YJR063W
YGL156W	YNL248C
YPR032W	YJR063W
YPR032W	YNL248C
YPR032W	YDR314C
YPR032W	YEL037C
YBL037W	YOL062C
YBL037W	YDL191W
YBL037W	YJR005W
YOL062C	YDL191W
YOL062C	YJR005W
YIL035C	YIL084C
YIL035C	YOR361C
YIL035C	YNL330C
YIL035C	YGL207W
YIL035C	YNL251C
YIL035C	YMR172W
YIL035C	YGL019W
YIL035C	YLR418C
YIL035C	YKL088W
YIL035C	YOR061W
YIL035C	YOL004W
YIL035C	YER164W
YIL035C	YOL145C
YIL035C	YKL064W
YIL035C	YML069W
YIL084C	YAL013W
YIL084C	YDR207C
YIL084C	YNL330C
YIL084C	YBR095C
YIL084C	YMR263W

YIL084C	YGL019W
YIL084C	YOL004W
YIL084C	YNL097C
YIL084C	YDL076C
YNR046W	YOL124C
YNR046W	YNR050C
YNR046W	YDR140W
YOL124C	YNR050C
YOL124C	YDR140W
YPL012W	YLR196W
YPL012W	YDR060W
YPL012W	YDL148C
YPL012W	YPL266W
YPL012W	YDR496C
YPL012W	YGL103W
YPL012W	YMR093W
YPL012W	YBR247C
YPL012W	YNL110C
YPL012W	YGL135W
YPL012W	YHR010W
YPL012W	YNL308C
YPL012W	YBL072C
YPL012W	YML024W
YDR238C	YFR051C
YDR238C	YIL076W
YDR238C	YPL010W
YDR238C	YDL145C
YDR238C	YER122C
YDR238C	YNL287W
YFR051C	YIL076W
YFR051C	YPL010W
YFR051C	YDL145C
YFR051C	YNL287W
YFR051C	YKR067W
YOR151C	YPR187W
YOR151C	YKL029C
YOR151C	YBR154C
YOR151C	YDR404C
YOR151C	YGR186W
YOR151C	YDL140C
YOR151C	YPR093C
YOR151C	YGL070C
YOR151C	YDL115C
YOR151C	YDR171W
YOR151C	YFR009W

YOR151C	YPL129W
YOR151C	YBL039C
YOR151C	YER139C
YPR187W	YJR063W
YPR187W	YOR340C
YPR187W	YDR404C
YPR187W	YGR186W
YPR187W	YDL140C
YPR187W	YGL070C
YPR187W	YNL248C
YPR187W	YOR341W
YPR187W	YDL150W
YPR187W	YOR207C
YKR008W	YGR056W
YKR008W	YLR357W
YKR008W	YCR052W
YKR008W	YIL126W
YKR008W	YLR033W
YNL306W	YBR251W
YNL306W	YGR215W
YNL306W	YBL090W
YNL306W	YGR084C
YNL306W	YOR158W
YNL306W	YPL013C
YNL306W	YKL003C
YNL306W	YOR243C
YNL306W	YMR158W
YNL306W	YJR101W
YNL306W	YJR113C
YNL306W	YPL118W
YNL306W	YNL186W
YNL306W	YIL093C
YDL087C	YPL178W
YDL087C	YGR162W
YDL087C	YHR165C
YDL087C	YPR182W
YDL087C	YML046W
YDL087C	YHR086W
YDL087C	YDR235W
YDL087C	YLR147C
YDL087C	YLR298C
YDL087C	YKL012W
YDL087C	YIL061C
YBR263W	YPL009C
YBR263W	YOL120C

YBR263W	YOR048C
YPL009C	YOL120C
YPL009C	YOR048C
YPL009C	YDR471W
YLR196W	YNL175C
YLR196W	YML100W
YHR186C	YJR066W
YJR066W	YBR021W
YJR066W	YLR373C
YJR066W	YJL081C
YJR066W	YER151C
YDR060W	YNL061W
YDR060W	YGL103W
YDR060W	YDL014W
YDR060W	YNL110C
YDR060W	YDL082W
YDR060W	YGL135W
YDR060W	YEL054C
YDR060W	YLR276C
YDR060W	YDL075W
YDR060W	YLR029C
YDR060W	YIL133C
YDR060W	YGR085C
YBR251W	YGR215W
YBR251W	YBL090W
YBR251W	YGR084C
YBR251W	YOR158W
YBR251W	YPL013C
YBR251W	YKL003C
YBR251W	YOR243C
YBR251W	YMR158W
YBR251W	YJR101W
YBR251W	YGR061C
YBR251W	YJR113C
YBR251W	YPL118W
YBR251W	YNR036C
YBR251W	YNL186W
YBR251W	YIL093C
YDR237W	YDR322W
YDR237W	YGR220C
YDR237W	YMR193W
YDR237W	YDR296W
YDR237W	YLR439W
YDR237W	YOR150W
YDR237W	YNL177C

YDR237WYHR147C
YDR237WYPL183W-A
YDR237WYBR122C
YDR237WYDR405W
YDR237WYNL185C
YDR322WYGR220C
YDR322WYMR193W
YDR322WYMR024W
YDR322WYDR296W
YDR322WYLR439W
YDR322WYOR150W
YDR322WYNL177C
YDR322WYHR147C
YDR322WYPL183W-A
YDR322WYBR122C
YDR322WYDR405W
YDR322WYNL185C
YOR349W YBL104C
YOR349W YDL134C
YOR349W YPR043W
YLR333C YMR203W
YLR333C YGR130C
YLR333C YKL101W
YLR333C YGR145W
YLR333C YER053C
YLR333C YJL066C
YMR203W YGR130C
YMR203W YAL010C
YMR203W YJL066C
YMR203W YPR024W
YMR203W YMR241W
YER165W YNL261W
YER165W YGL094C
YER165W YML117W
YER165W YGL049C
YER165W YBL085W
YNL261WYHR118C
YNL261WYML065W
YNL261WYBR060C
YCR063WYER013W
YCR063WYPR101W
YCR063WYHR156C
YCR063WYDL209C
YCR063WYHR165C
YCR063WYBR065C

YCR063W	YJR050W
YCR063W	YBL104C
YCR063W	YMR213W
YCR063W	YGR278W
YCR063W	YAL032C
YAL013W	YNL330C
YAL013W	YBR095C
YAL013W	YMR263W
YAL013W	YMR075W
YAL013W	YNL097C
YAL013W	YDL076C
YGR261C	YJL024C
YGR261C	YLR436C
YGR261C	YMR304W
YGR261C	YJL076W
YGR261C	YPL195W
YGR261C	YBR288C
YPL178W	YPL190C
YPL178W	YNL251C
YPL178W	YHR165C
YPL178W	YLR347C
YPL178W	YBR119W
YPL178W	YHR086W
YPL178W	YDR432W
YPL178W	YLR298C
YPL178W	YGL049C
YPL178W	YPR057W
YPL178W	YIL061C
YBL018C	YBR257W
YBL018C	YAL033W
YBL018C	YNL282W
YBL018C	YBR167C
YBL018C	YGR030C
YBL018C	YHR062C
YBL018C	YNL221C
YBR257W	YAL033W
YBR257W	YNL282W
YBR257W	YBR167C
YBR257W	YGR030C
YBR257W	YHR062C
YBR257W	YDR478W
YBR257W	YNL221C
YGL094C	YKL025C
YGL094C	YKL012W
YDL116W	YGL100W

YDL116W	YGL092W
YGL100W	YGL092W
YGL100W	YBL104C
YGR060W	YOR153W
YGR060W	YGL246C
YGR060W	YGL234W
YGR060W	YML029W
YOR153W	YAL026C
YOR153W	YBR202W
YOR153W	YGL234W
YOR153W	YKL092C
YOR153W	YML029W
YOR153W	YPL153C
YOR153W	YKL039W
YOR153W	YHL003C
YOR153W	YML072C
YOR153W	YAL053W
YER120W	YDR150W
YER120W	YHL020C
YAL036C	YOR293W
YAL036C	YGL246C
YAL036C	YGR162W
YAL036C	YHL034C
YAL036C	YOR243C
YAL036C	YLR406C
YAL036C	YGR285C
YAL036C	YLR310C
YAL036C	YOR048C
YOR293W	YOR361C
YOR293W	YNL030W
YOR293W	YOR303W
YOR293W	YOR056C
YOR293W	YMR205C
YOR293W	YJL168C
YGR215W	YBL090W
YGR215W	YGR084C
YGR215W	YOR158W
YGR215W	YPL013C
YGR215W	YKL003C
YGR215W	YOR243C
YGR215W	YMR158W
YGR215W	YJR101W
YGR215W	YJR113C
YGR215W	YPL118W
YGR215W	YNR036C

YGR215W	YNL186W
YGR215W	YOL115W
YGR215W	YIL093C
YER013W	YPR101W
YER013W	YBR065C
YER013W	YMR213W
YER013W	YGR278W
YER013W	YAL032C
YPR101W	YHR156C
YPR101W	YJR084W
YPR101W	YDL209C
YPR101W	YBR065C
YPR101W	YJR050W
YPR101W	YIR009W
YPR101W	YBL104C
YPR101W	YMR213W
YPR101W	YAL032C
YBR025C	YGL039W
YBR025C	YNL108C
YBR025C	YBR175W
YBR025C	YLR153C
YBR025C	YNL088W
YGL039W	YBR175W
YGL039W	YDL033C
YNL059C	YPL235W
YNL059C	YER092W
YNL059C	YLR052W
YNL059C	YOR141C
YNL059C	YDL002C
YNL059C	YJL081C
YNL059C	YDR190C
YNL059C	YGL150C
YNL059C	YPL129W
YNL059C	YFL013C
YNL059C	YPL053C
YPL235W	YER092W
YPL235W	YFL045C
YPL235W	YLR052W
YPL235W	YOR141C
YPL235W	YOR136W
YPL235W	YHR128W
YPL235W	YDL002C
YPL235W	YDR190C
YPL235W	YBL103C
YPL235W	YGL150C

YPL235W	YFL013C
YPL235W	YMR185W
YDL148C	YJR145C
YDL148C	YBL004W
YDL148C	YCR057C
YDL148C	YOR096W
YDL148C	YLR186W
YDL148C	YOL010W
YDL148C	YPR137W
YDL148C	YJR002W
YDL148C	YLR003C
YDL148C	YDL014W
YDL148C	YBR247C
YDL148C	YGR128C
YDL148C	YPL217C
YDL148C	YLR222C
YDL148C	YIL069C
YDL148C	YNL308C
YOR361C	YJL190C
YOR361C	YLR264W
YOR361C	YPR041W
YOR361C	YNL096C
YOR361C	YGL019W
YOR361C	YLR039C
YOR361C	YDR429C
YOR361C	YBL072C
YOR361C	YLR359W
YDR498C	YLR440C
YDR498C	YLR268W
YDR498C	YGL098W
YDR498C	YGL145W
YDR498C	YNL258C
YDR498C	YOR075W
YLR440C	YKL196C
YLR440C	YLR268W
YLR440C	YGL098W
YLR440C	YGL145W
YLR440C	YNL258C
YLR440C	YOR075W
YER125W	YJL084C
YER125W	YOR124C
YER125W	YOR138C
YER125W	YKR021W
YER125W	YHR131C
YDL203C	YLR371W

YDL203C	YPL066W
YDL203C	YLR425W
YDL203C	YBR011C
YLR371W	YPL083C
YLR371W	YPL183W-A
YLR371W	YGL131C
YLR371W	YLR386W
YLR371W	YNL127W
YLR371W	YPL066W
YLR371W	YLR425W
YLR371W	YBR011C
YJR022W	YMR268C
YHR156C	YDL209C
YHR156C	YHR165C
YHR156C	YBR065C
YHR156C	YJR050W
YHR156C	YBL104C
YHR156C	YMR213W
YHR156C	YGR278W
YHR156C	YOR308C
YHR156C	YDL030W
YML103C	YPL206C
YBR121C	YPL249C
YBR121C	YPL218W
YBR121C	YDR341C
YBR121C	YDR388W
YBR121C	YHR128W
YBR121C	YJL052W
YBR121C	YKL067W
YBR121C	YOR001W
YBR121C	YLL018C
YBR121C	YCR009C
YBR121C	YNR031C
YPL249C	YDR388W
YPL249C	YCR009C
YPL249C	YMR192W
YJL190C	YGR162W
YJL190C	YPR189W
YGR120C	YNL051W
YGR120C	YML071C
YGR120C	YNL041C
YGR120C	YER157W
YGR120C	YGL223C
YNL051W	YML071C
YNL051W	YNL041C

YNL051W	YER157W
YNL051W	YGL223C
YPL266W	YCR057C
YPL266W	YPR137W
YPL266W	YLR003C
YPL266W	YBR247C
YPL266W	YKL143W
YPL266W	YGR054W
YPL266W	YPL204W
YCL050C	YDR341C
YCL050C	YKL067W
YDR150W	YHL020C
YDR150W	YFL016C
YFR004W	YFR052W
YFR004W	YPR108W
YFR004W	YDL147W
YFR004W	YFR010W
YFR004W	YKL145W
YFR004W	YDL007W
YFR004W	YGL048C
YFR004W	YDR394W
YFR052W	YPR108W
YFR052W	YDL147W
YFR052W	YKL145W
YFR052W	YMR314W
YFR052W	YDL007W
YFR052W	YGL048C
YFR052W	YER012W
YFR052W	YDR394W
YFR052W	YOR117W
YGR220C	YMR193W
YGR220C	YMR024W
YGR220C	YDR296W
YGR220C	YLR439W
YGR220C	YOR150W
YGR220C	YNL177C
YGR220C	YHR147C
YGR220C	YPL183W-A
YGR220C	YBR122C
YGR220C	YDR405W
YGR220C	YIL104C
YGR063C	YPR180W
YGR063C	YML010W
YGR063C	YDR404C
YGR063C	YGR186W

YPR180W	YML010W
YPR180W	YDR404C
YPR180W	YGR186W
YPR180W	YDR390C
YLR102C	YLR127C
YLR102C	YHR166C
YLR102C	YGL240W
YLR102C	YDR118W
YLR102C	YOR249C
YLR102C	YBL084C
YLR102C	YKL022C
YLR102C	YNL172W
YLR127C	YHR166C
YLR127C	YGL240W
YLR127C	YDR118W
YLR127C	YOR249C
YLR127C	YBL084C
YLR127C	YKL022C
YLR127C	YNL172W
YDR148C	YFL018C
YDR148C	YGL030W
YDR148C	YMR121C
YDR148C	YIL125W
YFL018C	YBR221C
YFL018C	YGR193C
YFL018C	YIL125W
YFL018C	YDR430C
YFL018C	YMR211W
YMR240C	YPL213W
YMR240C	YJR084W
YMR240C	YHR165C
YMR240C	YIR009W
YMR240C	YDL043C
YMR240C	YOR319W
YMR240C	YBL104C
YMR240C	YLR147C
YMR240C	YPL151C
YMR240C	YJL203W
YMR240C	YDL030W
YPL213W	YHR165C
YPL213W	YIR009W
YPL213W	YLR147C
YPL213W	YAL032C
YPL213W	YJL203W
YPL213W	YDL030W

YHR041C	YDR308C
YHR041C	YPL042C
YHR041C	YGR104C
YHR041C	YPR168W
YHR041C	YNR010W
YBL004W	YER082C
YBL004W	YOL010W
YBL004W	YPR137W
YBL004W	YBR247C
YBL004W	YDL111C
YBL004W	YDR280W
YHR166C	YGL240W
YHR166C	YDR118W
YHR166C	YOR249C
YHR166C	YBL084C
YHR166C	YKL022C
YHR166C	YNL172W
YHR118C	YML065W
YHR118C	YBR060C
YML065W	YBR060C
YDR207C	YNL330C
YDR207C	YMR263W
YDR207C	YDL076C
YBR114W	YCR028C-A
YBR114W	YNL312W
YBR114W	YCR092C
YBR114W	YML032C
YBR114W	YDR499W
YBR114W	YDL156W
YBR114W	YDL225W
YBR114W	YPL154C
YBR114W	YOR332W
YBR114W	YOL090W
YER092W	YPL218W
YER092W	YLR052W
YER092W	YOR141C
YER092W	YDL192W
YER092W	YDL002C
YER092W	YDR190C
YER092W	YGL150C
YER092W	YBL099W
YER092W	YFL013C
YPL218W	YDR341C
YPL218W	YLR355C
YPL218W	YLR052W

YPL218W	YGL238W
YPL218W	YDL192W
YPL218W	YHR128W
YPL218W	YJL052W
YPL218W	YKL067W
YPL218W	YLL018C
YPL218W	YLL040C
YPL218W	YBL099W
YEL022W	YKL029C
YEL022W	YJR077C
YKL029C	YDR127W
YKL029C	YGL245W
YKL029C	YER086W
YKL029C	YNR016C
YKL029C	YFR009W
YKL029C	YDR335W
YKL029C	YBL076C
YKL029C	YDL143W
YKL029C	YDR188W
YBR103W	YIL112W
YBR103W	YDR155C
YBR103W	YLR347C
YBR103W	YCR033W
YBR103W	YKR029C
YBR103W	YGL194C
YBR103W	YOL068C
YIL112W	YDR155C
YIL112W	YGR178C
YIL112W	YCR033W
YIL112W	YKR029C
YIL112W	YGL194C
YBL056W	YDR071C
YBL056W	YPL204W
YBL056W	YBR097W
YBL056W	YER089C
YBR236C	YML010W
YML010W	YBR154C
YML010W	YDL149W
YML010W	YDR404C
YML010W	YDL140C
YML010W	YDR379W
YML010W	YNL201C
YDR496C	YNL175C
YDR496C	YNL061W
YDR496C	YOL127W

YDR496C YGL111W
YDR496C YPR016C
YDR496C YBL087C
YDR496C YLR175W
YDR496C YPL093W
YDR496C YLL008W
YDR496C YMR121C
YDR496C YKL014C
YDR496C YDL031W
YDR496C YLR276C
YNL175C YBR126C
YNL175C YBL087C
YNL175C YPL093W
YNL175C YMR121C
YNL175C YML100W
YNL175C YDL031W
YNL175C YLR276C
YNL175C YIL129C
YBR102C YML097C
YBR102C YJL085W
YBR102C YLR166C
YBR102C YGL233W
YBR102C YER008C
YBR102C YDR166C
YBR102C YPR055W
YBR102C YIL068C
YML097C YLR166C
YML097C YPR055W
YML097C YIL068C
YCR057C YER082C
YCR057C YHR148W
YCR057C YLR186W
YCR057C YMR093W
YCR057C YPR137W
YCR057C YJR002W
YCR057C YGR145W
YCR057C YLR129W
YCR057C YBR247C
YCR057C YGR128C
YCR057C YPL217C
YCR057C YLL011W
YCR057C YJL069C
YCR057C YNL075W
YCR057C YLR222C
YOL054WYDR300C

YOL054W	YDL070W
YOL054W	YDL058W
YOL054W	YEL056W
YOL054W	YLL022C
YOL054W	YFR006W
YOL054W	YBR272C
YOL054W	YJR132W
YOL120C	YPR174C
YKL193C	YLR028C
YKL193C	YER133W
YKL193C	YKL088W
YKL193C	YER070W
YKL193C	YAR014C
YBR267W	YOR312C
YBR267W	YDR101C
YBR267W	YMR242C
YBR267W	YNL227C
YBR267W	YHR170W
YDR127W	YJL008C
YDR127W	YGR240C
YDR127W	YGR234W
YDR127W	YMR186W
YDR127W	YDR214W
YDR127W	YMR299C
YJL008C	YOR281C
YJL008C	YDL029W
YMR193W	YMR024W
YMR193W	YDR296W
YMR193W	YLR439W
YMR193W	YOR150W
YMR193W	YNL177C
YMR193W	YHR147C
YMR193W	YPL183W-A
YMR193W	YBR122C
YMR193W	YDR405W
YBR126C	YPR035W
YBR126C	YMR083W
YBR126C	YML100W
YBR126C	YDR074W
YBR126C	YMR261C
YPR035W	YDR171W
YPR035W	YML100W
YPR035W	YDR074W
YPR035W	YMR261C
YHR197W	YNL182C

YHR197W	YDR101C
YHR197W	YGR245C
YHR197W	YPR016C
YHR197W	YPL093W
YHR197W	YNL110C
YNL182C	YDR101C
YNL182C	YGR245C
YNL182C	YPR016C
YNL182C	YNL110C
YBL090W	YGR084C
YBL090W	YOR158W
YBL090W	YPL013C
YBL090W	YKL003C
YBL090W	YOR243C
YBL090W	YMR158W
YBL090W	YJR101W
YBL090W	YJR113C
YBL090W	YPL118W
YBL090W	YNR036C
YBL090W	YNL186W
YBL090W	YIL093C
YGR084C	YOR158W
YGR084C	YPL013C
YGR084C	YKL003C
YGR084C	YMR158W
YGR084C	YJR101W
YGR084C	YGR061C
YGR084C	YJR113C
YGR084C	YPL118W
YGR084C	YNL186W
YGR084C	YIL093C
YFL045C	YDR341C
YFL045C	YGL167C
YFL045C	YHR128W
YFL045C	YKL067W
YFL045C	YNR031C
YFL045C	YNL169C
YGR166W	YDR246W
YGR166W	YKR068C
YGR166W	YDR108W
YGR166W	YMR218C
YGR166W	YBR254C
YGR166W	YDR472W
YGR166W	YGR143W
YGR166W	YML077W

YLR264W YNL096C
YLR264W YBR247C
YLR264W YOR056C
YLR264W YGR054W
YLR264W YPL204W
YLR264W YLR039C
YLR264W YBL024W
YDR448WYGL066W
YDR448WYGL112C
YDR448WYMR236W
YDR448WYGR252W
YDR448WYBR198C
YDR448WYDR145W
YDR448WYPL254W
YDR448WYDR167W
YDR448WYCL010C
YDR448WYBR081C
YGL066W YGL112C
YGL066W YMR236W
YGL066W YGR252W
YGL066W YBR198C
YGL066W YDR145W
YGL066W YPL254W
YGL066W YMR223W
YGL066W YDR167W
YGL066W YCL010C
YGL066W YBR081C
YDR245WYDR525W-A
YDR245WYJL183W
YDR245WYPL094C
YDR245WYJR075W
YDR245WYPL050C
YDR245WYCR043C
YDR525W-A YGR132C
YMR024W YDR296W
YMR024W YLR439W
YMR024W YNL177C
YMR024W YBR122C
YMR024W YDR405W
YMR024W YNL185C
YDR189WYKL196C
YDR189WYBL050W
YKL196C YBL050W
YKL196C YLR268W
YKL196C YGL098W

YKL196C	YGL145W
YKL196C	YOR075W
YKL196C	YOR036W
YKL196C	YGL095C
YDR296W	YLR439W
YDR296W	YOR150W
YDR296W	YNL177C
YDR296W	YHR147C
YDR296W	YPL183W-A
YDR296W	YBR122C
YDR296W	YDR405W
YDR296W	YNL185C
YDR296W	YIL104C
YPR041W	YNL096C
YPR041W	YDR091C
YPR041W	YOR096W
YPR041W	YDL149W
YPR041W	YLR039C
YPR041W	YDR429C
YPR041W	YIL133C
YMR239C	YPL190C
YMR239C	YNL251C
YMR239C	YOL115W
YMR239C	YDL175C
YMR239C	YJL050W
YPL190C	YNL251C
YPL190C	YML117W
YPL190C	YOL115W
YPL190C	YDL175C
YPL190C	YJL050W
YHR023W	YGL106W
YHR023W	YAL029C
YHR023W	YDR145W
YHR023W	YMR299C
YJL184W	YML036W
YJL184W	YGR262C
YJL184W	YKR038C
YML036W	YGR262C
YML036W	YKR038C
YPR108W	YDL147W
YPR108W	YOL038W
YPR108W	YKL145W
YPR108W	YGL048C
YPR108W	YER012W
YPR108W	YOL087C

YDL147W	YFR010W
YDL147W	YKL145W
YDL147W	YMR314W
YDL147W	YDL007W
YDL147W	YMR025W
YDL147W	YOL117W
YDL147W	YGL048C
YDL147W	YBL041W
YDL147W	YER012W
YDL147W	YDR394W
YDL147W	YDL216C
YDL147W	YJL001W
YDL147W	YOR117W
YDL147W	YDR179C
YDL147W	YIL071C
YDR341C	YLR355C
YDR341C	YGL238W
YDR341C	YHR128W
YDR341C	YJL052W
YDR341C	YKL067W
YDR341C	YLL018C
YDR341C	YDR335W
YDR341C	YBL076C
YDR341C	YLL040C
YLR355C	YHR128W
YLR355C	YJL052W
YLR355C	YKL067W
YLR355C	YLL018C
YLR355C	YDR035W
YLR355C	YJL200C
YNL096C	YDL149W
YNL096C	YGR054W
YNL096C	YLR039C
YNL061W	YKL009W
YNL061W	YDR087C
YNL061W	YPR016C
YNL061W	YPL093W
YNL061W	YLL008W
YNL061W	YPR102C
YNL061W	YKL014C
YNL061W	YDL031W
YNL061W	YGR085C
YLR226W	YPR161C
YLR226W	YPR057W
YLR226W	YDL175C

YDR101C YKL009W
YDR101C YGR245C
YDR101C YPR016C
YDR101C YNL110C
YDR101C YHR170W
YDR101C YDL075W
YDR101C YOR252W
YKL009W YPR016C
YER082C YHR148W
YER082C YLR186W
YER082C YMR093W
YER082C YPR137W
YER082C YJR002W
YER082C YLR003C
YER082C YDL014W
YER082C YGR145W
YER082C YLR129W
YER082C YBR247C
YER082C YGR128C
YER082C YPL217C
YER082C YLL011W
YER082C YPR112C
YER082C YLR222C
YNL330C YBR095C
YNL330C YMR263W
YNL330C YOL004W
YNL330C YPR023C
YNL330C YMR075W
YNL330C YNL097C
YNL330C YDL076C
YNL330C YEL018W
YNL330C YLR103C
YDR091C YOR096W
YDR091C YDL149W
YDR091C YDR429C
YDR091C YML063W
YDR091C YML024W
YOR096W YGR285C
YBR095C YMR263W
YBR095C YOL004W
YBR095C YPR023C
YBR095C YMR075W
YBR095C YNL097C
YBR095C YDL076C
YBR095C YCR084C

YMR263W	YOL004W
YMR263W	YMR075W
YMR263W	YNL097C
YMR263W	YDL076C
YMR263W	YCR084C
YLR291C	YOR260W
YLR291C	YDR211W
YLR291C	YGR083C
YLR291C	YJR007W
YLR291C	YKR026C
YLR291C	YOR101W
YLR291C	YOR335C
YOR260W	YDR211W
YOR260W	YGR083C
YOR260W	YJR007W
YOR260W	YKR026C
YLR330W	YJL099W
YLR330W	YMR237W
YBR069C	YNL064C
YBR069C	YDL043C
YBR069C	YPL233W
YNL064C	YPL233W
YNL064C	YER171W
YNL064C	YGL055W
YLR337C	YOR181W
YLR337C	YML001W
YGL207W	YGL019W
YGL207W	YBR279W
YGL207W	YLR418C
YGL207W	YOR061W
YGL207W	YGR217W
YGL207W	YOL145C
YGL207W	YML069W
YGL207W	YGR163W
YNL102W	YDR292C
YNL102W	YBL035C
YER155C	YJL098W
YER155C	YDL047W
YER155C	YNL201C
YER155C	YPR088C
YJL098W	YDL047W
YJL098W	YPL049C
YJL098W	YGL197W
YJL098W	YLR310C
YJL098W	YKR028W

YJL098W	YBR214W
YDL188C	YPR040W
YDL188C	YGL190C
YDL188C	YAL016W
YDL188C	YJR009C
YDL188C	YPL106C
YDL188C	YOR014W
YBR127C	YLR447C
YBR127C	YDL185W
YBR127C	YKL080W
YBR127C	YJR033C
YLR447C	YAL026C
YLR447C	YOR270C
YLR447C	YKL119C
YLR447C	YMR054W
YLR439W	YOR150W
YLR439W	YNL177C
YLR439W	YHR147C
YLR439W	YPL183W-A
YLR439W	YBR122C
YLR439W	YDR405W
YLR439W	YNL185C
YOR150W	YHR147C
YOR150W	YPL183W-A
YOR150W	YBR122C
YOR150W	YOR069W
YOR150W	YDR405W
YOR150W	YIL104C
YFR010W	YKL145W
YFR010W	YDL007W
YFR010W	YGL048C
YFR010W	YDR394W
YDR300C	YBR208C
YDR300C	YNL030W
YDR300C	YOR303W
YDR300C	YDL070W
YDR300C	YNL265C
YDR300C	YNL001W
YDR300C	YFR006W
YDR300C	YGR194C
YOR290C	YBR289W
YOR290C	YDR073W
YOR290C	YNR023W
YOR290C	YFL049W
YOR290C	YPL016W

YFL048C	YIL053W
YDR155C	YCR033W
YDR155C	YKR029C
YDR155C	YGL194C
YBL064C	YDR069C
YDR069C	YBR208C
YDR069C	YBR221C
YDR069C	YLR436C
YDR069C	YMR304W
YDR069C	YGR193C
YBL050W	YGL095C
YGL245W	YDL055C
YGL245W	YKR071C
YGL245W	YGL105W
YGL245W	YGR264C
YGL246C	YDL047W
YGL246C	YDR447C
YGL246C	YGL234W
YGL246C	YOR048C
YGL246C	YDR049W
YGL246C	YML029W
YGL246C	YBL058W
YGL246C	YGR048W
YKL010C	YOR239W
YKL010C	YIL094C
YKL010C	YJR009C
YKL010C	YDR214W
YNL177C	YBR208C
YNL177C	YPL183W-A
YNL177C	YBR122C
YNL177C	YDR405W
YNL177C	YIL104C
YBR154C	YJR063W
YBR154C	YOR340C
YBR154C	YDR404C
YBR154C	YGL070C
YBR154C	YDL115C
YBR154C	YNL248C
YBR154C	YNR003C
YBR154C	YOR341W
YBR154C	YOR207C
YJR063W	YOR340C
YJR063W	YNL248C
YJR063W	YOR341W
YJR063W	YFR028C

YMR078C	YOR144C
YMR078C	YPR175W
YMR078C	YLL040C
YMR078C	YNL290W
YHR148W	YLR186W
YHR148W	YPR137W
YHR148W	YLR003C
YHR148W	YBR247C
YHR148W	YLL011W
YHR148W	YNL075W
YLR186W	YLR003C
YLR186W	YGR145W
YLR186W	YBR247C
YLR186W	YLL011W
YBR107C	YDR254W
YBR107C	YLR381W
YBR107C	YJR135C
YBR107C	YDR318W
YHR147C	YPL183W-A
YHR147C	YBR122C
YHR147C	YDR405W
YIL018W	YJL033W
YIL018W	YBR011C
YDR246W	YKR068C
YDR246W	YDR108W
YDR246W	YMR218C
YDR246W	YBR254C
YDR246W	YDR472W
YDR246W	YGR143W
YDR246W	YML077W
YKR068C	YDR108W
YKR068C	YMR218C
YKR068C	YBR254C
YKR068C	YDR472W
YKR068C	YGR143W
YKR068C	YML077W
YGR034W	YLR221C
YGR034W	YDR365C
YGR034W	YJL033W
YGR034W	YDR450W
YGR034W	YGR281W
YGR034W	YNR043W
YGR034W	YHR169W
YGR034W	YOR173W
YGR034W	YDR460W

YLR221C	YGR281W
YLR221C	YOL144W
YLR221C	YNL104C
YLR221C	YJR041C
YLR221C	YOL058W
YLR221C	YOR108W
YBR084C-A	YDR365C
YBR084C-A	YJL033W
YBR084C-A	YNR043W
YBR084C-A	YHR169W
YDR365C	YJL033W
YDR365C	YGR145W
YDR365C	YNR043W
YDR365C	YHR169W
YDR365C	YNL075W
YGR232W	YML092C
YGR232W	YKL145W
YGR232W	YGL048C
YGR232W	YBL041W
YGR232W	YER012W
YGR232W	YDR394W
YGL130W	YOR370C
YGL130W	YKR014C
YGL130W	YER103W
YGL130W	YBL075C
YGL130W	YHR121W
YGL130W	YPL228W
YOR370C	YKR014C
YOR370C	YDR328C
YOR370C	YOR347C
YOR370C	YPL228W
YOR370C	YMR258C
YGR245C	YOL127W
YGR245C	YPR016C
YGR245C	YPL093W
YGR245C	YNL110C
YGR245C	YPL259C
YML092C	BLM3
YML092C	YOL038W
YML092C	YFR050C
YML092C	YMR314W
YML092C	YPL020C
YML092C	YJL001W
YGL190C	YDL070W
YGL190C	YAL016W

YGL190C	YDL134C
YLR028C	YLR389C
YLR028C	YER133W
YLR028C	YNR034W
YLR028C	YER070W
YLR028C	YAR014C
YLR389C	YPL184C
YLR389C	YNR034W
YLR389C	YMR037C
YGR056W	YCR052W
YGR056W	YIL126W
YGR056W	YLR033W
YBR208C	YBR221C
YBR208C	YPL259C
YBR208C	YGR193C
YBR208C	YER178W
YPL083C	YDL058W
YPL083C	YLR386W
YPL083C	YOR187W
YPL083C	YIL129C
YOR158W	YPL013C
YOR158W	YKL003C
YOR158W	YMR158W
YOR158W	YJR101W
YOR158W	YGR061C
YOR158W	YJR113C
YOR158W	YPL118W
YOR158W	YNR036C
YOR158W	YIL093C
YCR077C	YGR162W
YCR077C	YDL160C
YCR077C	YER112W
YCR077C	YJL124C
YCR077C	YNL256W
YCR077C	YER146W
YCR077C	YMR268C
YCR077C	YLR438C-A
YGR162W	YHL034C
YGR162W	YLR175W
YGR162W	YOR243C
YGR162W	YGR285C
YGR162W	YCL011C
YGR162W	YNL262W
YGR162W	YIL061C
YGR162W	YIR001C

YGR162W	YCL037C
YGR162W	YJL138C
YNL251C	YML117W
YNL251C	YCL011C
YNL251C	YGL049C
YNL251C	YOL115W
YNL251C	YCL037C
YNL251C	YDL175C
YNL251C	YJL050W
YPL243W	YDL092W
YPL243W	YML105C
YPL243W	YDR214W
YPL243W	YLR452C
YPL243W	YPR088C
YPL243W	YOR374W
YML071C	YNL041C
YML071C	YNL265C
YML071C	YER157W
YML071C	YGL223C
YML071C	YGR194C
YDL167C	YMR080C
YDL167C	YML117W
YDL167C	YJL033W
YDL167C	YDR432W
YDL167C	YPL016W
YDL167C	YIR001C
YDL167C	YNL016W
YMR080C	YJL033W
YMR080C	YGR180C
YMR080C	YDR037W
YMR080C	YJL124C
YMR080C	YMR083W
YMR080C	YER146W
YMR080C	YIR001C
YMR080C	YNL016W
YMR080C	YLR438C-A
YMR080C	YCL010C
BLM3	YOL038W
BLM3	YFR050C
BLM3	YJL001W
BLM3	YKL168C
YOL038W	YIL094C
YOL038W	YFR050C
YOL038W	YKL145W
YOL038W	YMR314W

YOL038W	YBL041W
YOL038W	YER012W
YOL038W	YDR394W
YOL038W	YJL001W
YOL038W	YMR308C
YJR084W	YIR009W
YJR084W	YPR182W
YJR084W	YOR319W
YJR084W	YBL104C
YJR084W	YLR298C
YJR084W	YPR057W
YMR005W	YGL112C
YMR005W	YMR236W
YMR005W	YBR198C
YMR005W	YML015C
YMR005W	YPL011C
YMR005W	YMR227C
YMR005W	YDR145W
YMR005W	YDR167W
YMR005W	YML098W
YAL033W	YNL282W
YAL033W	YBR167C
YAL033W	YGR030C
YAL033W	YHR062C
YAL033W	YNL221C
YNL282W	YBR167C
YNL282W	YGR030C
YNL282W	YHR062C
YNL282W	YNL221C
YGL111W	YPR016C
YGL111W	YPL093W
YGL111W	YNL110C
YGL111W	YPL131W
YGL111W	YGL076C
YPR016C	YPL093W
YPR016C	YNL110C
YPR016C	YLL008W
YPR016C	YDL031W
YPR016C	YLR029C
YPR016C	YIL133C
YFR030W	YIL094C
YFR030W	YJR137C
YIL094C	YGL238W
YIL094C	YOR136W
YIL094C	YPL240C

YIL094C	YGR218W
YIL094C	YER110C
YGL106W	YKL130C
YGL106W	YAL029C
YGL106W	YBR202W
YGL106W	YOR035C
YGL106W	YBR130C
YGL106W	YPL153C
YKL130C	YAL029C
YKL130C	YBR202W
YKL130C	YBR130C
YKL130C	YPL153C
YKL130C	YIL034C
YKL130C	YKL007W
YAR007C	YJL173C
YAR007C	YPR065W
YAR007C	YFR013W
YAR007C	YLR347C
YAR007C	YNL312W
YAR007C	YCR092C
YAR007C	YML032C
YAR007C	YDL156W
YAR007C	YHR164C
YAR007C	YOL090W
YAR007C	YMR234W
YJL173C	YLR347C
YJL173C	YNL312W
YJL173C	YCR092C
YJL173C	YML032C
YJL173C	YDL156W
YJL173C	YMR234W
YML117W	YPL184C
YML117W	YIR001C
YDL047W	YPL049C
YDL047W	YNR016C
YDL047W	YGL197W
YDL047W	YLR310C
YDL047W	YFR019W
YDL047W	YDL219W
YDL047W	YFR040W
YDL047W	YIL142W
YDL047W	YKR028W
YPL049C	YDR432W
YPL049C	YKR028W
YPL049C	YBL017C

YLR429W	YNL108C
YLR429W	YNL225C
YLR429W	YMR262W
YLR429W	YGL157W
YLR429W	YPR125W
YDL149W	YAL021C
YDL149W	YDR429C
YGL103W	YNL110C
YGL103W	YPR189W
YGL103W	YML072C
YOR340C	YNL248C
YOR340C	YOR341W
YOR340C	YFR028C
YGL112C	YMR236W
YGL112C	YGR252W
YGL112C	YBR198C
YGL112C	YML015C
YGL112C	YPL011C
YGL112C	YDR044W
YGL112C	YMR227C
YGL112C	YDR145W
YGL112C	YER148W
YGL112C	YPL254W
YGL112C	YGR264C
YGL112C	YDR167W
YGL112C	YGR094W
YGL112C	YCL010C
YGL112C	YBR081C
YGL112C	YAL017W
YGL112C	YML114C
YMR236W	YGR252W
YMR236W	YBR198C
YMR236W	YML015C
YMR236W	YPL011C
YMR236W	YMR227C
YMR236W	YDR145W
YMR236W	YPL254W
YMR236W	YMR223W
YMR236W	YDR167W
YMR236W	YBR081C
YLR052W	YOR141C
YLR052W	YDL192W
YLR052W	YDL002C
YLR052W	YDR190C
YLR052W	YGL150C

YLR052W	YBL099W
YLR052W	YFL013C
YOR141C	YDL002C
YOR141C	YDR190C
YOR141C	YGL150C
YOR141C	YFL013C
YPR065W	YOL090W
YDL209C	YHR165C
YDL209C	YBR065C
YDL209C	YJR050W
YDL209C	YMR213W
YDL209C	YGR278W
YDL209C	YPL151C
YDL209C	YAL032C
YPL013C	YKL003C
YPL013C	YOR243C
YPL013C	YMR158W
YPL013C	YJR101W
YPL013C	YJR113C
YPL013C	YPL118W
YPL013C	YNR036C
YPL013C	YNL186W
YPL013C	YOL115W
YPL013C	YER091C
YPL013C	YIL093C
YER086W	YDL055C
YER086W	YJR109C
YFL026W	YGL238W
YFL026W	YMR089C
YGL238W	YHR128W
YGL238W	YJL052W
YGL238W	YDR170C
YGL238W	YKL067W
YGL238W	YLL018C
YGL238W	YDR335W
YGL238W	YKL092C
YGL238W	YKL039W
YGL238W	YFR044C
YBR170C	YHR092C
YBR170C	YDR049W
YBR170C	YBL058W
YBR170C	YML013W
YBR170C	YGR048W
YOR136W	YDR170C
YOR136W	YER110C

YOR136W	YPL226W
YOR136W	YPL061W
YJR045C	YKL096W
YJR045C	YLR203C
YJR045C	YKR048C
YJR045C	YGR155W
YJR045C	YNL078W
YJR045C	YKR082W
YBL087C	YPL208W
YBL087C	YDR361C
YHR165C	YBR065C
YHR165C	YPR182W
YHR165C	YMR213W
YHR165C	YBR152W
YHR165C	YER112W
YHR165C	YLR147C
YHR165C	YGR278W
YHR165C	YOR308C
YHR165C	YER146W
YHR165C	YPR178W
YHR165C	YAL032C
YHR165C	YJL203W
YHR165C	YDL030W
YFR013W	YGL133W
YFR013W	YPL082C
YFR013W	YDR190C
YFR013W	YDR083W
YFR013W	YOL004W
YFR013W	YCR084C
YFR013W	YIL031W
YGL133W	YOR304W
YGL133W	YDR121W
YOR181W	YJL201W
YOR181W	YGL206C
YOR181W	YFR024C-A
YOR181W	YML001W
YOR181W	YCR030C
YOR181W	YNL243W
YOR181W	YDL191W
YOR181W	YBL027W
YDL160C	YER112W
YDL160C	YJL124C
YDL160C	YDR170C
YDL160C	YLR438C-A
YBR105C	YOR250C

YBR105C	YNL030W
YBR105C	YDL082W
YBR105C	YDR228C
YBR105C	YGL044C
YBR105C	YGL227W
YOR250C	YHR216W
YOR250C	YDL195W
YOR250C	YBL075C
YOR250C	YDR228C
YOR250C	YGL044C
YBR065C	YJR050W
YBR065C	YPR182W
YBR065C	YBL104C
YBR065C	YMR213W
YBR065C	YPL151C
YBR065C	YAL032C
YJR050W	YBL104C
YJR050W	YMR213W
YJR050W	YAL032C
YOL010W	YLR003C
YOL010W	YPL217C
YOL010W	YPR112C
YOL010W	YNL075W
YOL010W	YER102W
YOL010W	YHR203C
YBR221C	YGR178C
YBR221C	YMR308C
YBR221C	YPL259C
YBR221C	YGR193C
YBR221C	YDR430C
YBR221C	YER178W
YBR221C	YPR152C
YGR178C	YHR121W
YGR178C	YGL221C
YLR347C	YPL020C
YLR347C	YAR002W
YLR347C	YMR308C
YLR347C	YKR029C
YLR347C	YLR005W
YLR347C	YDR502C
YLR347C	YER110C
YLR347C	YDL175C
YLR347C	YDR257C
YKL172W	YLR406C
YKL172W	YLL008W

YKL172W	YER117W
YKL172W	YCL054W
YKL172W	YER139C
YDL192W	YBL099W
YNL030W	YOR303W
YNL030W	YKL067W
YNL030W	YEL056W
YNL030W	YGL049C
YNL030W	YLL022C
YOR303W	YEL056W
YOR303W	YLL022C
YOR303W	YJR109C
YHL034C	YLR175W
YHL034C	YAL003W
YHL034C	YCL011C
YHL034C	YGL049C
YHL034C	YPL048W
YHL034C	YCL037C
YHL034C	YBR118W
YLR175W	YJL033W
YLR175W	YGL049C
YLR175W	YNL262W
YLR175W	YCL037C
YLR175W	YMR310C
YDR108W	YMR218C
YDR108W	YBR254C
YDR108W	YDR472W
YDR108W	YGR143W
YDR108W	YML077W
YGR130C	YER053C
YGR130C	YJL066C
YGR130C	YMR031C
YGR130C	YKR018C
YPL183W-A	YCR030C
YPL183W-A	YBR122C
YPL183W-A	YDR405W
YPL183W-A	YIL104C
YMR218C	YBR254C
YMR218C	YDR472W
YMR218C	YGR143W
YMR218C	YML077W
YBR254C	YDR472W
YBR254C	YGR143W
YBR254C	YML077W
YBR254C	YLR342W

YJL201W	YLR150W
YJL201W	YFR024C-A
YJL201W	YCR030C
YJL201W	YDL191W
YJL201W	YBL027W
YLR150W	YJL099W
YLR150W	YIL056W
YLR150W	YOR252W
YDR211W	YGR083C
YDR211W	YJR007W
YDR211W	YKR026C
YKL003C	YOR243C
YKL003C	YMR158W
YKL003C	YJR101W
YKL003C	YJR113C
YKL003C	YPL118W
YKL003C	YNR036C
YKL003C	YNL186W
YKL003C	YOL115W
YKL003C	YIL093C
YOR243C	YMR158W
YOR243C	YJR101W
YOR243C	YPL118W
YOR243C	YNL186W
YOR243C	YGL232W
YOR243C	YOR356W
YFR050C	YMR314W
YFR050C	YBL041W
YFR050C	YER012W
YFR050C	YJL001W
YBR234C	YIL062C
YBR234C	YLR370C
YBR234C	YNR035C
YBR234C	YKL013C
YBR234C	YDL029W
YBR234C	YJR064W
YIL062C	YLR370C
YIL062C	YNR035C
YIL062C	YKL013C
YIL062C	YDL029W
YDR388W	YCR009C
YDR254W	YLR381W
YDR254W	YJR135C
YDR254W	YGR179C
YDR254W	YDR318W

YDR254W	YPR046W
YGR083C	YJR007W
YGR083C	YKR026C
YDL074C	YPR191W
YDL074C	YDR432W
YDL074C	YBL045C
YPR191W	YDR529C
YPR191W	YBL045C
YPR191W	YLR132C
YPR191W	YDL189W
YDR529C	YBL045C
YGL167C	YNL169C
YMR093W	YPR137W
YMR093W	YBR247C
YMR093W	YGR128C
YMR093W	YJL069C
YPR137W	YJR002W
YPR137W	YPL217C
YPR137W	YLL011W
YPR137W	YJL069C
YPR137W	YNL075W
YPR137W	YLR222C
YKL145W	YDL007W
YKL145W	YGL048C
YKL145W	YDR394W
YKL145W	YJL001W
YKL145W	YOR117W
YKL145W	YJL194W
YKL145W	YGR218W
YGL206C	YBR169C
YGL206C	YER103W
YGL206C	YPL106C
YGL206C	YIR006C
YGL206C	YNL168C
YJL020C	YGR028W
YJL020C	YIR003W
YJL020C	YJL123C
YDR404C	YGR186W
YDR404C	YDL140C
YDR404C	YPR093C
YDR404C	YGL070C
YDR404C	YDL115C
YGR186W	YPR093C
YGR186W	YGL070C
YGR186W	YDL115C

YER036C	YPR036W
YER036C	YLR153C
YER036C	YER070W
YER036C	YHL033C
YPR036W	YEL051W
YPR036W	YDR202C
YPR036W	YOR332W
YPR036W	YKL080W
YPR036W	YJR033C
YIR009W	YPR182W
YIR009W	YOR319W
YIR009W	YBL104C
YIR009W	YBR152W
YIR009W	YDL030W
YPR182W	YML046W
YPR182W	YHR086W
YPR182W	YDR235W
YPR182W	YLR147C
YPR182W	YLR298C
YPR182W	YKL012W
YDL140C	YDR251W
YDL140C	YPR093C
YDL140C	YGL070C
YDL140C	YDL115C
YDL140C	YER139C
YDR251W	YNL271C
YBR158W	YGR285C
YBR158W	YGR163W
YGR285C	YHR010W
YGR285C	YPR189W
YGR285C	YGR054W
YGR285C	YOL121C
YGR132C	YJL183W
YGR132C	YER017C
YGR132C	YMR089C
YGR132C	YPL050C
YJL183W	YJR075W
YJL183W	YPL050C
YJL183W	YCR043C
YJL183W	YLR450W
YDL070W	YDL058W
YDL070W	YFR006W
YDL070W	YJR132W
YPR093C	YDL115C
YNL049C	YIL109C

YNL049C	YPL085W
YNL049C	YGR116W
YHR128W	YJL052W
YHR128W	YKL067W
YHR128W	YLL018C
YHR128W	YBL076C
YHR128W	YLL040C
YBR052C	YCR004C
YBR052C	YNL010W
YBR052C	YDR032C
YCR004C	YNL010W
YCR004C	YDR032C
YGR252W	YDR145W
YGR252W	YPL254W
YGR252W	YMR223W
YGR252W	YDR167W
YGR252W	YBR081C
YDL043C	YOR319W
YDL043C	YHR086W
YDL043C	YJL203W
YOR319W	YBL104C
YOR319W	YJL203W
YEL051W	YOR270C
YEL051W	YDR202C
YEL051W	YOR332W
YEL051W	YDL185W
YEL051W	YKL080W
YEL051W	YJR033C
YPL082C	YIL126W
YPL082C	YER148W
YPL082C	YDR397C
YLR357W	YCR052W
YLR357W	YIL126W
YLR357W	YLR033W
YLR357W	YDR225W
YLR357W	YGR275W
YKL125W	YML126C
YKL125W	YDL191W
YKL125W	YGR027C
YML126C	YGR027C
YJR002W	YLR003C
YJR002W	YBR247C
YJR002W	YNL075W
YLR003C	YGR145W
YLR003C	YLR129W

YLR003C	YGR128C
YLR003C	YPR112C
YLR003C	YCL031C
YCR052W	YIL126W
YCR052W	YLR033W
YHR216W	YDL195W
YHR216W	YDR228C
YFR024C-A	YNL243W
YBL104C	YMR213W
YER133W	YDR195W
YER133W	YKR002W
YER133W	YGR156W
YER133W	YKL059C
YER133W	YDR301W
YER133W	YLR115W
YER133W	YER070W
YER133W	YAR014C
YER133W	YMR311C
YMR213W	YGR278W
YMR213W	YPL151C
YMR213W	YAL032C
YAL026C	YML072C
YAL026C	YAL053W
YBR152W	YOR308C
YBR152W	YPR178W
YBR152W	YGR075C
YDL014W	YJL033W
YDL014W	YBR247C
YDL014W	YCL011C
YJL033W	YDR432W
YJL033W	YNR043W
YJL033W	YHR169W
YJL033W	YNL075W
YJL033W	YNL069C
YJL033W	YIR001C
YJL033W	YNL016W
YGR180C	YJL026W
YGR180C	YJL115W
YOR323C	YPR179C
YOR323C	YDR295C
YOR323C	YOL090W
YOR323C	YNL021W
YPR179C	YDR295C
YPR179C	YOL090W
YPR179C	YNL021W

YDR507C YOL070C
YDR507C YGL241W
YDR507C YKR048C
YDR507C YNL078W
YDR507C YCL024W
YDR507C YAL027W
YOL070C YGL241W
YOL070C YKR048C
YOL070C YNL078W
YOL070C YCL024W
YDR308C YPL042C
YDR308C YDR443C
YDR308C YGR104C
YDR308C YPR168W
YDR308C YNR010W
YAL003W YKL101W
YAL003W YPL048W
YAL003W YDR314C
YAL003W YBR118W
YKL101W YPL048W
YKL101W YDR314C
YKL101W YDR204W
YJL052W YKL067W
YJL052W YLL018C
YJL052W YLL040C
YDR195WYKR002W
YDR195WYGR156W
YDR195WYKL059C
YDR195WYDR301W
YDR195WYLR115W
YDR195WYNL222W
YGL200C YOR016C
YGL200C YAR002C-A
YGL200C YML012W
YGL200C YJR132W
YGL200C YHR110W
YGL200C YDL018C
YGL200C YAL007C
YOR016C YAR002C-A
YGR145WYNR043W
YGR145WYLL011W
YGR145WYDL166C
YGR145WYCL031C
YDR037WYER112W
YDR037WYJL124C

YDR037W	YER146W
YDR037W	YLR438C-A
YDR037W	YPR056W
YER112W	YJL124C
YER112W	YLR147C
YER112W	YOR308C
YER112W	YER146W
YER112W	YPR178W
YER112W	YPR082C
YER112W	YMR268C
YER112W	YPR057W
YER112W	YJL203W
YER112W	YLR438C-A
YER112W	YDL030W
YMR158W	YJR101W
YMR158W	YJR113C
YMR158W	YPL118W
YMR158W	YNR036C
YMR158W	YER091C
YMR158W	YIL093C
YGR240C	YPL193W
YGR240C	YMR205C
YGR240C	YOR286W
YGR240C	YNL045W
YDR472W	YGR143W
YDR472W	YML077W
YMR314W	YBL041W
YMR314W	YER012W
YMR314W	YJL001W
YDR450W	YBR247C
YFL024C	YJL081C
YFL024C	YPR023C
YFL024C	YPL074W
YFL024C	YEL018W
YFL024C	YDR359C
YFL024C	YNL107W
YDR005C	YJL011C
YDR005C	YNR003C
YDR005C	YOR207C
YGL131C	YMR052W
YGL131C	YBR169C
YGL131C	YDR050C
YGL131C	YNL127W
YGL131C	YKL152C
YMR052W	YBR169C

YMR052W	YMR029C
YMR052W	YHL001W
YMR052W	YDR050C
YMR052W	YNL127W
YJL124C	YNL118C
YJL124C	YER146W
YJL124C	YLR438C-A
YCR028C-A	YCR033W
YCR028C-A	YML032C
YCR028C-A	YIL109C
YCR028C-A	YMR234W
YCR033W	YKR029C
YCR033W	YGL194C
YAR002C-A	YML012W
YAR002C-A	YAL007C
YJL011C	YNR003C
YJL011C	YDL150W
YJL011C	YOR207C
YJL011C	YPR055W
YBR021W	YLR373C
YBR021W	YER151C
YLR373C	YER151C
YLR373C	YPL128C
YDL058W	YLR386W
YDL058W	YBL035C
YDL058W	YFR006W
YBR198C	YML015C
YBR198C	YPL011C
YBR198C	YDR145W
YBR198C	YER148W
YBR198C	YPL254W
YBR198C	YDR167W
YBR198C	YCL010C
YBR198C	YBR081C
YBR198C	YML114C
YDL007W	YHR042W
YGR143W	YML077W
YPL233W	YAL034W-A
YLR386W	YIL129C
YCR030C	YLR432W
YCR030C	YDR405W
YCR030C	YDR348C
YCR030C	YBL047C
YCR030C	YOL016C
YLR432W	YLR005W

YPL020C	YGL197W
YPL020C	YAR002W
YPL020C	YMR308C
YPL020C	YMR310C
YBR119W	YML046W
YBR119W	YHR086W
YBR119W	YDR235W
YBR119W	YLR298C
YBR119W	YKL012W
YBR119W	YPR057W
YBR119W	YIL061C
YML046W	YHR086W
YML046W	YDR235W
YML046W	YLR298C
YML046W	YKL012W
YML046W	YIL061C
YBR122C	YDR405W
YBR122C	YIL104C
YDR517W	YJR052W
YDR517W	YOR231W
YDR517W	YJL099W
YDR517W	YGR136W
YDR517W	YIL109C
YDR517W	YIL172C
YDR517W	YOR089C
YDR517W	YNL193W
YGL022W	YMR149W
YGL022W	YEL002C
YMR149W	YEL002C
YLR129W	YBR247C
YLR129W	YJL069C
YMR172W	YGL019W
YMR172W	YKL088W
YMR172W	YER103W
YMR172W	YER164W
YMR172W	YDR429C
YGL070C	YDL115C
YGL070C	YPL129W
YDL002C	YJL081C
YDL002C	YDR190C
YDL002C	YGL150C
YDL002C	YOR304W
YDL002C	YFL013C
YJL081C	YPR023C
YJL081C	YDR359C

YJL081C	YNL107W
YLR203C	YOR232W
YLR203C	YDR430C
YOR232W	YDR430C
YKL139W	YBR169C
YNL312W	YML032C
YNL312W	YDR499W
YNL312W	YDL156W
YNL312W	YHR164C
YDL055C	YNR016C
YDL055C	YML028W
YDL055C	YJR109C
YNR016C	YJR064W
YIL143C	YOR168W
YIL143C	YOR352W
YOR168W	YOR352W
YIL076W	YPL010W
YIL076W	YDL145C
YIL076W	YNL287W
YOR133W	YBR246W
YCR092C	YDR499W
YCR092C	YDL156W
YCR092C	YOL090W
YPL042C	YCR081W
YPL042C	YDR443C
YPL042C	YGR104C
YPL042C	YPR168W
YPL042C	YNR010W
YPL042C	YOR174W
YGL197W	YLR310C
YGL197W	YFR040W
YGL197W	YKR028W
YGL197W	YBR225W
YLR310C	YFR040W
YLR310C	YKR028W
YLR310C	YBR225W
YLR268W	YGL098W
YLR268W	YGL145W
YLR268W	YNL258C
YLR268W	YOR075W
YBR289W	YDR073W
YBR289W	YNR023W
YBR289W	YFL049W
YBR289W	YPL016W
YDR073W	YNR023W

YDR073W	YFL049W
YDR073W	YPL016W
YDR073W	YPL129W
YDR073W	YGR275W
YBR247C	YKL143W
YBR247C	YPL217C
YBR247C	YLL011W
YBR247C	YOR056C
YBR247C	YGR054W
YBR247C	YNL075W
YBR247C	YPL204W
YBR247C	YNL308C
YBR247C	YBL072C
YOR270C	YKL119C
YOR270C	YGR281W
YOR270C	YDR516C
YOR270C	YNR050C
YOR270C	YDL185W
YOR270C	YJL012C
YOR270C	YBL099W
YOR270C	YEL013W
YGL019W	YLR418C
YGL019W	YOR061W
YGL019W	YOL004W
YGL019W	YER164W
YGL019W	YOL145C
YGL019W	YKL064W
YGL019W	YML069W
YGL019W	YLR223C
YAL029C	YBR202W
YAL029C	YBR130C
YAL029C	YMR223W
YAL029C	YBR081C
YBR202W	YBR130C
YBR202W	YPL153C
YBR202W	YIL034C
YBR202W	YKL007W
YBR202W	YGL001C
YDL111C	YDR280W
YDL111C	YPR189W
YDL111C	YOR001W
YDL111C	YOL142W
YKL119C	YDR516C
YKL119C	YNR050C
YKL143W	YML091C

YKL143W	YOR056C
YKL143W	YGR054W
YKL143W	YPL204W
YJR007W	YKR026C
YJR007W	YOR101W
YJR007W	YOR335C
YJR007W	YBR039W
YKR026C	YOR101W
YKR026C	YOR335C
YNL216W	YPL128C
YDR190C	YPL240C
YDR190C	YER164W
YDR190C	YBL103C
YDR190C	YGL150C
YDR190C	YFL013C
YDR190C	YPL053C
YPL240C	YOR027W
YPL240C	YPL161C
YPL240C	YGR123C
YCR081W	YDR443C
YCR081W	YPR168W
YCR081W	YNR010W
YDR443C	YGR104C
YDR443C	YPR168W
YDR443C	YNR010W
YBR169C	YNL127W
YBR169C	YOR007C
YBR169C	YOR164C
YHR086W	YDR432W
YHR086W	YDR235W
YHR086W	YLR147C
YHR086W	YLR298C
YHR086W	YKL012W
YHR086W	YGL049C
YHR086W	YIL061C
YPL093W	YNL110C
YPL093W	YHR010W
YPL093W	YPR102C
YDR432W	YIR001C
YDL102W	YJR043C
YDL102W	YJR006W
YJR043C	YJR006W
YIL126W	YLR033W
YIL126W	YGR275W
YDR202C	YOR332W

YDR202C	YKL080W
YDR202C	YDR328C
YDR202C	YJR033C
YDL040C	YHR013C
YDL040C	YAL009W
YDL040C	YPL198W
YDL040C	YGR016W
YHR013C	YPL198W
YHR013C	YGR254W
YJL024C	YPL195W
YJL024C	YBR288C
YNL041C	YER157W
YNL041C	YGL223C
YMR029C	YHL001W
YNL110C	YLL008W
YNL110C	YDL031W
YNL110C	YDL075W
YNL110C	YCL054W
YBR260C	YGR196C
YGR196C	YDL080C
YGR196C	YIL142W
YAL016W	YDL134C
YAL016W	YOR014W
YDL134C	YOR014W
YMR025W	YOL117W
YMR025W	YDL216C
YMR025W	YDR179C
YMR025W	YIL071C
YOL117W	YDL216C
YOL117W	YDR179C
YOL117W	YIL071C
YDR083W	YOR217W
YDR083W	YGL241W
YDR083W	YNL201C
YOR217W	YOR144C
YOR217W	YBL035C
YOR217W	YJR068W
YOR217W	YNL290W
YDR447C	YGR128C
YDR447C	YOR056C
YGR128C	YIL069C
YDR235W	YLR298C
YDR235W	YKL012W
YDR235W	YIL061C
YOR144C	YJR068W

YOR144C	YNL290W
YBL041W	YER012W
YBL041W	YJL001W
YER012W	YJL001W
YGR233C	YOL001W
YGR233C	YPL031C
YGR233C	YPL219W
YBR279W	YLR418C
YBR279W	YOL145C
YBR279W	YML069W
YLR418C	YOR061W
YLR418C	YOL145C
YLR418C	YML069W
YLR170C	YPR029C
YLR170C	YPL259C
YLR170C	YBL032W
YIL153W	YNL201C
YDR280W	YPR189W
YDR280W	YOR001W
YDR280W	YLR398C
YDR280W	YOL142W
YKL088W	YOR061W
YKL088W	YER103W
YKL088W	YER164W
YKL088W	YBL075C
YKL088W	YDR429C
YOR061W	YOL004W
YOR061W	YER164W
YOR061W	YOL145C
YOR061W	YKL064W
YOR061W	YML069W
YOR061W	YLR223C
YOR061W	YLR407W
YOR069W	YJL154C
YOR069W	YJL053W
YOR069W	YHR012W
YHR208W	YJR148W
YJR101W	YJR113C
YJR101W	YPL118W
YJR101W	YNL186W
YJR101W	YIL093C
YHR019C	YDL142C
YGL030W	YGR281W
YGL030W	YKL067W
YGR281W	YOR014W

YGR281W	YBL099W
YGR281W	YLR075W
YGL098W	YGL145W
YGL098W	YNL258C
YGL098W	YOR075W
YLR147C	YKL012W
YML015C	YPL011C
YML015C	YMR227C
YML015C	YDR145W
YPL011C	YDR145W
YNL256W	YPR178W
YNL256W	YMR268C
YNL218W	YEL058W
YAL001C	YOR110W
YAL001C	YBR123C
YAL001C	YDR362C
YAL001C	YGR047C
YAL001C	YPL007C
YOR110W	YBR123C
YOR110W	YDR362C
YOR110W	YGR047C
YOR110W	YBR089C-A
YOR110W	YPL007C
YDR394W	YJR109C
YML091C	YNL118C
YML091C	YPL204W
YNL118C	YLR438C-A
YNL118C	YPL226W
YER017C	YML075C
YER017C	YMR089C
YML075C	YMR089C
YML075C	YER095W
YML075C	YLR450W
YDR170C	YJR077C
YDR170C	YBR205W
YDR170C	YFR044C
YDR170C	YLR342W
YJR009C	YDR198C
YJR009C	YPL106C
YBR123C	YDR362C
YBR123C	YGR047C
YBR123C	YPL007C
YDR362C	YGR047C
YDR362C	YPL007C
YDR405W	YNL185C

YGR047C	YIL056W
YGR047C	YMR284W
YGR047C	YPL007C
YPL010W	YDL145C
YPL010W	YNL287W
YPL010W	YKR067W
YGR278W	YPL151C
YGR278W	YKL095W
YGR278W	YAL032C
YDR516C	YNR050C
YOL004W	YPR023C
YOL004W	YHL020C
YOL004W	YMR075W
YOL004W	YNL097C
YOL004W	YDL076C
YOL004W	YCR084C
YOL004W	YIL101C
YOL004W	YKL072W
YPR023C	YMR075W
YPR023C	YEL018W
YPR023C	YDR359C
YPR023C	YNL107W
YGR104C	YPR168W
YGR104C	YNR010W
YGR104C	YOR174W
YPR168W	YNR010W
YPR168W	YOR174W
YNR023W	YFL049W
YNR023W	YPL016W
YDL216C	YDR179C
YDL216C	YIL071C
YJL039C	YJR077C
YJL039C	YGR119C
YJL039C	YFR002W
YJL039C	YGL172W
YJR113C	YPL118W
YJR113C	YNL186W
YJR113C	YIL093C
YDR225W	YLR183C
YDR225W	YPR057W
YDR225W	YDR243C
YDR225W	YMR043W
YLR183C	YDR243C
YLR183C	YHR195W
YDL092W	YML105C

YDL092W	YLR452C
YDL092W	YPR088C
YPL118W	YNL186W
YPL118W	YIL093C
YJR077C	YKL182W
YJR077C	YGR218W
YJR077C	YMR186W
YJR077C	YBR017C
YKL182W	YML100W
YKL067W	YLL018C
YKL067W	YDR335W
YKL067W	YBL076C
YKL067W	YLL040C
YLL048C	YML070W
YLL048C	YNL238W
YLL048C	YOL031C
YBL030C	YKR052C
YBL030C	YLR153C
YBL030C	YKL092C
YBL030C	YKL039W
YKR052C	YJL066C
YJL085W	YLR166C
YJL085W	YGL233W
YJL085W	YER008C
YJL085W	YDR166C
YJL085W	YPR055W
YJL085W	YIL068C
YLR166C	YGL233W
YLR166C	YER008C
YLR166C	YDR166C
YLR166C	YPR055W
YLR166C	YIL068C
YEL056W	YLL022C
YEL056W	YBR272C
YPL206C	YMR246W
YNL265C	YGR194C
YHR119W	YPL138C
YHR119W	YLR015W
YHR119W	YAR003W
YHR119W	YDR469W
YPL138C	YBR175W
YPL138C	YLR015W
YPL138C	YAR003W
YPL138C	YDR469W
YNL227C	YHR170W

YNL227C	YFR002W
YOR294W	YPR043W
YPR043W	YDL195W
YNL248C	YOR341W
YNR043W	YHR169W
YNR043W	YNL075W
YBR160W	YKR091W
YBR160W	YBR135W
YBR160W	YPL256C
YBR160W	YDL155W
YBR160W	YMR199W
YBR160W	YLR079W
YKR091W	YBR135W
YKR091W	YPL256C
YKR091W	YDL155W
YKR091W	YMR199W
YKR091W	YLR079W
YLR381W	YJR135C
YLR381W	YGR179C
YLR381W	YDR318W
YLR381W	YPR046W
YDL044C	YMR083W
YDL044C	YLR139C
YDL044C	YCR009C
YDL044C	YBL061C
YMR083W	YLR139C
YMR083W	YCR009C
YLL011W	YNL075W
YKR002W	YGR156W
YKR002W	YKL059C
YKR002W	YDR301W
YKR002W	YDR228C
YKR002W	YLR115W
YKR002W	YNL222W
YGL233W	YER008C
YGL233W	YDR166C
YGL233W	YIL068C
YGL234W	YML029W
YGL234W	YNL178W
YLR240W	YPL120W
YLR240W	YBR097W
YLR240W	YLR360W
YPL120W	YBR097W
YPL120W	YLR360W
YPL120W	YBR128C

YBR135W	YPL256C
YBR135W	YDL155W
YBR135W	YMR199W
YBR135W	YLR079W
YBR135W	YGR155W
YPL256C	YDL155W
YPL256C	YMR199W
YPL256C	YLR079W
YPL256C	YGR155W
YGL241W	YKR048C
YGL241W	YNL078W
YGL241W	YCL024W
YKR048C	YNL078W
YKR048C	YCL024W
YKR048C	YLR133W
YKR048C	YMR139W
YJR135C	YGR179C
YJR135C	YDR318W
YJR135C	YPR046W
YHR169W	YNL075W
YHR169W	YLR029C
YMR117C	YOL069W
YMR117C	YIL144W
YMR117C	YER018C
YOL069W	YIL144W
YOL069W	YER018C
YOR004W	YPR112C
YPR112C	YGR054W
YPR112C	YDL165W
YOR056C	YGR054W
YOR056C	YNL075W
YOR056C	YIL069C
YOR056C	YBL072C
YPL084W	YPR173C
YPL084W	YLR025W
YPR173C	YLR025W
YJL099W	YMR237W
YHR170W	YFR002W
YHR170W	YKL180W
YML032C	YDR499W
YML032C	YDL156W
YML032C	YHR164C
YER157W	YGL223C
YLR139C	YCR009C
YLR304C	YMR246W

YLR304C	YGR234W
YMR246W	YGL082W
YHR010W	YPR189W
YFL008W	YMR121C
YFL008W	YJL026W
YFL008W	YER147C
YFL008W	YPL207W
YMR121C	YPL207W
YMR121C	YIL125W
YMR075W	YNL097C
YMR075W	YDL076C
YMR075W	YCR084C
YMR075W	YEL018W
YPR189W	YLR398C
YPR189W	YGL213C
YBR175W	YLR015W
YBR175W	YAR003W
YLR015W	YAR003W
YLR015W	YDR469W
YNR050C	YDR140W
YNR050C	YOR164C
YOR308C	YPR178W
YOR308C	YGR075C
YDR135C	YDR171W
YDR171W	YAR035W
YDR171W	YJR010W
YER008C	YDR166C
YER008C	YPR055W
YER008C	YIL068C
YBR205W	YFR044C
YBR205W	YML072C
YBR205W	YBR031W
YGR078C	YNL153C
YHL002W	YNR006W
YKR014C	YFL038C
YKR014C	YER136W
YKR014C	YDR328C
YKR014C	YER031C
YKR014C	YPL228W
YKR014C	YMR258C
YPL208W	YER103W
YPL208W	YER117W
YPL208W	YDR361C
YNL243W	YDR348C
YNL243W	YBL047C

YNL243W	YIR006C
YML100W	YDR074W
YML100W	YMR261C
YML100W	YPL106C
YDL195W	YIL109C
YDL195W	YGR155W
YDL195W	YPL085W
YLR298C	YKL012W
YLR298C	YPR057W
YLR298C	YIL061C
YLR044C	YOR245C
YLR044C	YKL039W
YLR044C	YLR134W
YBR167C	YGR030C
YBR167C	YHR062C
YBR167C	YNL221C
YLR436C	YMR304W
YOR001W	YOL142W
YNR010W	YOR174W
YNL004W	YHR167W
YNL004W	YDR138W
YNL004W	YNL139C
YNL004W	YDL084W
YNL004W	YJR121W
YDL145C	YER122C
YDL145C	YNL287W
YER122C	YNL287W
YKL142W	YKR066C
YKL142W	YKL092C
YKL142W	YDL215C
YKR066C	YKL092C
YKR066C	YDL215C
YGR017W	YLR410W
YLR410W	YDL174C
YLL018C	YLL040C
YDR044W	YMR227C
YDR044W	YGR264C
YDR044W	YDR167W
YDR044W	YGR094W
YDR044W	YAL017W
YMR227C	YDR167W
YMR227C	YAL017W
YMR227C	YML098W
YMR227C	YML114C
YCL011C	YHR167W

YCL011C	YDR138W
YCL011C	YNL139C
YCL011C	YHR203C
YBR171W	YLR292C
YBR171W	YOR254C
YBR171W	YPL094C
YLR292C	YOR254C
YLR292C	YPL094C
YPR029C	YPL259C
YPR029C	YBL032W
YDR381W	YHR167W
YDR381W	YDR138W
YDR381W	YOR211C
YDR381W	YML034W
YHR167W	YDR138W
YHR167W	YNL139C
YHR167W	YDL084W
YAR002W	YPR174C
YAR002W	YER110C
YHR172W	YNL126W
YDR186C	YBR125C
YDR099W	YDL117W
YDR099W	YAL017W
YDR099W	YMR296C
YKL014C	YOL144W
YKL014C	YJR041C
YOL144W	YNL104C
YOL144W	YJR041C
YOL144W	YMR297W
YOL144W	YOL058W
YOL144W	YNR038W
YOL144W	YOR108W
YNL135C	YLR005W
YNL135C	YNL023C
YDR138W	YNL139C
YDR138W	YDL084W
YLR398C	YGL213C
YLR398C	YBR039W
YLR398C	YDR168W
YDR145W	YER148W
YDR145W	YOR174W
YDR145W	YNL272C
YDR145W	YDR167W
YDR145W	YGL013C
YDR145W	YCL010C

YDR145W	YBR081C
YER148W	YNL272C
YJL154C	YOR048C
YJL154C	YJL053W
YJL154C	YHR012W
YER146W	YPR178W
YER146W	YLR438C-A
YPR178W	YJL203W
YDL191W	YGR027C
YDL191W	YJR005W
YGR156W	YKL059C
YGR156W	YDR301W
YGR156W	YLR115W
YKL059C	YDR301W
YKL059C	YLR115W
YER103W	YER164W
YER103W	YPL228W
YER103W	YDR361C
YER164W	YDR429C
YDR499W	YDL156W
YDR499W	YHR164C
YDR499W	YDL225W
YDR499W	YOL090W
YPL254W	YMR223W
YPL254W	YCL010C
YPL254W	YBR081C
YFL049W	YPL016W
YNR003C	YDL150W
YNR003C	YOR207C
YDR379W	YNL201C
YDR071C	YGR234W
YDR071C	YBR125C
YDR071C	YER089C
YML105C	YLR452C
YML105C	YPR088C
YOR035C	YKL129C
YOR035C	YBR109C
YGR054W	YPL204W
YGR054W	YOL121C
YGR028W	YJL123C
YKL213C	YOR341W
YKL213C	YBL058W
YJL069C	YNL075W
YJL069C	YLR222C
YNL075W	YLR222C

YKL012W	YPR057W
YKL012W	YIL061C
YNL104C	YJR041C
YNL104C	YOL058W
YNL104C	YOR108W
YDR049W	YBL058W
YDR049W	YGR048W
YER004W	YFR009W
YBR130C	YPL153C
YBR130C	YIL034C
YBR130C	YKL007W
YBR034C	YPR129W
YBR034C	YML017W
YPL016W	YIR001C
YPL016W	YGL013C
YER027C	YGL208W
YER027C	YER129W
YER027C	YDR477W
YGL208W	YER129W
YGL208W	YDR477W
YDR166C	YPR055W
YDR166C	YIL068C
YDL150W	YOR207C
YAR003W	YDR469W
YOR101W	YOR335C
YPL204W	YER102W
YPL204W	YPL081W
YDL156W	YDL225W
YDL156W	YOL090W
YLR153C	YDR502C
YLR153C	YLR249W
YLR153C	YER070W
YLR153C	YKL152C
YLR153C	YML028W
YLR153C	YPL226W
YLR153C	YDR385W
YHR164C	YOL090W
YDL225W	YJR076C
YDL225W	YJR092W
YDR331W	YHR188C
YDR331W	YLR088W
YHR188C	YLR088W
YGL049C	YIR001C
YHR146W	YPL154C
YHR146W	YMR196W

YPL154C	YDR233C
YPL154C	YMR196W
YBL103C	YOL067C
YJR041C	YMR297W
YJR041C	YOL058W
YJR041C	YNR038W
YJR041C	YOR108W
YMR297W	YNR038W
YMR297W	YLR178C
YMR297W	YOL086C
YMR308C	YOL115W
YMR308C	YHL003C
YMR308C	YAL061W
YGR234W	YLL013C
YGR234W	YPL106C
YGR234W	YDR188W
YLL013C	YHR121W
YGL150C	YPL129W
YGL150C	YFL013C
YDL166C	YCR031C
YJL026W	YML055W
YJL026W	YJL115W
YOR332W	YDL185W
YOR332W	YKL080W
YOR332W	YJR033C
YPL259C	YGR193C
YPL259C	YBL032W
YDR335W	YKL104C
YDR335W	YDR141C
YKL104C	YER178W
YER102W	YDR429C
YMR205C	YMR262W
YMR205C	YML006C
YDR292C	YBL035C
YDR292C	YKL154W
YGL145W	YNL258C
YGL145W	YOR075W
YKR029C	YGL194C
YBR115C	YGL154C
YBR115C	YAL059W
YAL021C	YER068W
YAL021C	YNL288W
YAL021C	YGR134W
YNL097C	YDL076C
YNL097C	YCR084C

YDL185W	YER171W
YDL185W	YKL080W
YOR254C	YPL094C
YPL151C	YAL032C
YDR074W	YMR261C
YDR074W	YPL106C
YNL186W	YOL115W
YNL186W	YIL093C
YOL115W	YDL175C
YOL115W	YJL050W
YMR231W	YPL045W
YMR231W	YDL077C
YMR231W	YLR148W
YMR231W	YDR080W
YPL045W	YDL077C
YPL045W	YLR148W
YPL045W	YDR080W
YGL105W	YGR264C
YFR019W	YKL117W
YFR019W	YFR040W
YFR019W	YKR028W
YFR019W	YER072W
YDL155W	YMR199W
YDL155W	YLR079W
YLR370C	YNR035C
YLR370C	YKL013C
YLR370C	YDL029W
YNR035C	YKL013C
YNR035C	YDL029W
YJR076C	YJR092W
YGR175C	YNL272C
YGR175C	YLL001W
YGR175C	YNL183C
YGR175C	YHR020W
YNL272C	YGL013C
YNL272C	YIL129C
YLR005W	YPR056W
YER147C	YPL090C
YER147C	YBL027W
YER147C	YDR180W
YBL075C	YJL155C
YBL075C	YLR345W
YBL075C	YPL228W
YJL155C	YLR345W
YBL076C	YDL100C

YBL076C	YBR058C
YDL100C	YER083C
YDL100C	YJL194W
YDR301W	YLR115W
YDR502C	YER070W
YDR502C	YMR296C
YMR199W	YLR079W
YGR193C	YDR430C
YGR193C	YER178W
YNL258C	YOR075W
YDR228C	YLR115W
YDR228C	YGL044C
YLR115W	YHL033C
YGR179C	YDR318W
YDR295C	YOL090W
YDR295C	YNL021W
YDL042C	YJL076W
YJL076W	YGR208W
YLR039C	YDR137W
YIL069C	YDR429C
YNL069C	YHR183W
YDL143W	YOR281C
YDL143W	YJL111W
YCL032W	YLR362W
YKL013C	YDL029W
YGL240W	YDR118W
YGL240W	YOR249C
YGL240W	YBL084C
YGL240W	YKL022C
YGL240W	YNL172W
YER083C	YJL194W
YJL194W	YDR196C
YJL194W	YFL044C
YAL041W	YNL007C
YAL041W	YBR200W
YAL041W	YER114C
YDL076C	YCR084C
YFL038C	YPR017C
YFL038C	YER136W
YFL038C	YER031C
YNL262W	YPR175W
YNL262W	YDR121W
YPR175W	YDR121W
YPR175W	YBR278W
YGR217W	YNL088W

YER151C	YLR441C
YER151C	YNR051C
YER151C	YDL066W
YDR050C	YJL049W
YDR050C	YNL127W
YDR050C	YKL065C
YJL053W	YHR012W
YOR173W	YDR460W
YOR173W	YDL136W
YOR173W	YPR025C
YOR173W	YLR270W
YOL142W	YKL126W
YFL022C	YLR060W
YAL010C	YJL066C
YJL066C	YMR241W
YMR223W	YDR167W
YMR223W	YCL010C
YMR223W	YBR081C
YMR152W	YPL106C
YPL106C	YML028W
YOR304W	YDR121W
YLR249W	YOR165W
YLR249W	YOR249C
YLR249W	YER150W
YLR249W	YLR043C
YLR249W	YJR142W
YJL061W	YIL115C
YOL058W	YOR108W
YGR218W	YMR235C
YDR158W	YJR109C
YDR179C	YIL071C
YKL092C	YKL039W
YKL092C	YDR141C
YKL092C	YDL215C
YKL092C	YKL008C
YOR281C	YDR188W
YOR281C	YJL111W
YOR281C	YNL212W
YOR281C	YIL142W
YPR161C	YPR057W
YPR161C	YDL175C
YER091C	YPR074C
YER091C	YNR030W
YPR074C	YML028W
YPR074C	YBR117C

YER136W	YLR272C
YER136W	YER031C
YER136W	YLR262C
YGR027C	YPR004C
YLR079W	YGR155W
YLR079W	YBL047C
YJL012C	YEL013W
YNL238W	YOL031C
YDR121W	YBL002W
YDR121W	YBR278W
YDR121W	YJL065C
YKL095W	YAL032C
YIL056W	YMR284W
YIL056W	YPR052C
YDR318W	YPR046W
YLL022C	YBR272C
YOR187W	YJR064W
YJR075W	YPL050C
YJR075W	YBR106W
YDL077C	YLR148W
YDL077C	YDR080W
YDR167W	YBR081C
YDR167W	YML098W
YBR207W	YFL041W
YNL032W	YNR033W
YNL032W	YHL029C
YNL032W	YNL099C
YNL032W	YCR095C
YNL032W	YNL056W
YNR033W	YML028W
YBL111C	YNL088W
YDR188W	YJL111W
YDR188W	YNL212W
YDR188W	YHR104W
YDR188W	YGR187C
YDR188W	YMR028W
YFR040W	YKR028W
YFR040W	YGL056C
YBL061C	YDR471W
YBL061C	YGR185C
YMR284W	YMR106C
YGL155W	YKL019W
YKL019W	YDL090C
YMR268C	YLR438C-A
YML029W	YOL013C

YML029W	YDR406W
YKL080W	YJR033C
YER070W	YAR014C
YDR328C	YJR033C
YDR328C	YMR258C
YBR039W	YDR168W
YBR039W	YBL099W
YBR039W	YDR298C
YGR262C	YKR038C
YIL109C	YGR155W
YIL109C	YPL085W
YJL004C	YNL231C
YJL004C	YCL031C
YNL231C	YCL031C
YBL058W	YGR048W
YPR057W	YDL175C
YPR057W	YFL017W-A
YPR174C	YER110C
YHR121W	YPL228W
YGR030C	YHR062C
YGR030C	YNL221C
YGR094W	YMR234W
YGR094W	YAL017W
YNL288W	YGR134W
YDR430C	YER178W
YPL129W	YML114C
YJL115W	YPL153C
YJL115W	YJR140C
YPL153C	YIL034C
YPL153C	YJR140C
YER007C-A	YJR014W
YJL164C	YPL203W
YJL164C	YIL033C
YPL203W	YIL033C
YMR214W	YBR162C
YOR014W	YAL024C
YPL048W	YDR314C
YPL048W	YBR118W
YJL117W	YMR134W
YKL152C	YDL090C
YKL152C	YDR084C
YMR186W	YJR132W
YKL039W	YDR141C
YKL039W	YKL008C
YIR001C	YNL016W

YER117W	YDR361C
YIL144W	YER018C
YDR216W	YHR174W
YJL111W	YNL212W
YJL111W	YIL142W
YJL111W	YMR028W
YBL099W	YDR298C
YPL050C	YCR043C
YOR124C	YOR138C
YGL013C	YOR038C
YLR148W	YDR080W
YLR148W	YHR047C
YBR196C	YLR088W
YBR196C	YHR036W
YBR196C	YPL109C
YLL001W	YNL183C
YNL212W	YIL142W
YNL212W	YJR064W
YPL128C	YJR122W
YHL003C	YHR047C
YJL203W	YDL030W
YGR075C	YLR438C-A
YEL002C	YKL181W
YKL181W	YHL011C
YAL039C	YAR035W
YDR460W	YDL136W
YDR460W	YPR025C
YEL018W	YDR359C
YEL018W	YNL107W
YHR062C	YDR478W
YHR062C	YNL221C
YDR348C	YBL047C
YGR155W	YPL085W
YGR155W	YBL047C
YBL039C	YHL011C
YBL039C	YBL036C
YBL039C	YPL220W
YHR024C	YLR172C
YHR024C	YLR163C
YDL080C	YIL142W
YDR118W	YOR249C
YDR118W	YBL084C
YDR118W	YKL022C
YDR118W	YNL172W
YOR249C	YBL084C

YOR249C	YKL022C
YOR249C	YNL172W
YGR119C	YFR002W
YGR119C	YGL172W
YJR092W	YKL085W
YIL142W	YKR028W
YNL139C	YDL084W
YIL104C	YPL061W
YOL090W	YNL021W
YER129W	YDR477W
YEL013W	YPR149W
YER110C	YCL009C
YER110C	YJL138C
YJR074W	YOR185C
YOR185C	YPL226W
YCL010C	YBR081C
YDL136W	YOR038C
YOR038C	YBR159W
YPL195W	YBR288C
YER043C	YFL028C
YDR359C	YNL107W
YNL010W	YDR032C
YDR129C	YIL172C
YOL001W	YPL031C
YOL001W	YPL219W
YJR068W	YLR048W
YJR068W	YNL290W
YDR320C	YLR423C
YPL066W	YLR425W
YPL066W	YBR011C
YJL101C	YPL226W
YJL101C	YPL061W
YPL226W	YPL061W
YBR097W	YLR360W
YOL123W	YBR017C
YLR425W	YBR011C
YBR011C	YNL023C
YBR011C	YIL161W
YDR214W	YER090W
YOR036W	YOR212W
YBL084C	YKL022C
YBL084C	YNL172W
YIL034C	YKL007W
YIL034C	YIR003W
YIL034C	YER071C

YOR007C	YOR164C
YKL129C	YBR109C
YDL208W	YMR310C
YOR213C	YOR229W
YER090W	YKL211C
YDL029W	YJR064W
YDR314C	YEL037C
YLR452C	YPR088C
YPR055W	YIL068C
YDR068W	YDR483W
YJL208C	YMR099C
YJL208C	YKR079C
YMR099C	YPL231W
YMR099C	YKR079C
YMR099C	YIL078W
YGR187C	YDR385W
YGR187C	YBR155W
YFR002W	YGL172W
YER095W	YLR450W
YBR053C	YDR032C
YFR006W	YJR132W
YNR051C	YDL066W
YML012W	YGL142C
YML012W	YHR110W
YML012W	YDL018C
YML012W	YAL007C
YHL029C	YNL099C
YHL029C	YCR095C
YHL029C	YNL056W
YNL099C	YCR095C
YNL099C	YNL056W
YDL175C	YJL050W
YCR095C	YNL056W
YNL078W	YCL024W
YNR031C	YJL128C
YNR031C	YLR006C
YDR152W	YGR173W
YBL085W	YBR238C
YBR109C	YLR433C
YBR109C	YML057W
YNR007C	YOR160W
YOR160W	YDR342C
YOR160W	YML120C
YBL047C	YOL016C
YPL031C	YPL219W

YPL031C	YIL050W
YCL024W	YAL027W
YKL007W	YIR003W
YKL007W	YER071C
YFR021W	YNL242W
YDR035W	YOR230W
YGR134W	YJR011C
YJR132W	YPL058C
YIL129C	YML081W
YIL129C	YGL091C
YML072C	YAL053W
YML072C	YPL220W
YDR141C	YNL297C
YDR478W	YNL221C
YBL045C	YLR132C
YBL045C	YDL189W
YDR342C	YHR047C
YKL022C	YNL172W
YJR070C	YLR168C
YHR110W	YDL018C
YBR200W	YER114C
YMR235C	YAL012W
YMR235C	YMR300C
YPL231W	YCL034W
YAL012W	YMR300C
YGL125W	YML082W
YGL125W	YPL023C
YLR372W	YNL055C
YJL138C	YKR059W
YNL023C	YIL161W
YHR047C	Q0080
YDL113C	YJL036W
YDL113C	YDR425W
YJL036W	YDR425W
YDR027C	YJL029C
YER111C	YLR182W
YLR182W	YDL056W
YBL017C	YKL054C
YIR003W	YER071C
YMR192W	YLR274W
YER139C	YJR072C
YLR035C	YMR167W
YMR167W	YNL082W
YIL103W	YKL191W
YMR296C	YDR062W

YNL192W	YDR231C
YGR254W	YMR014W
YGR254W	YBL105C
YAL024C	YHR158C
YML102W	YPR018W
YML102W	YBR195C
YPR018W	YBR195C
YGL162W	YKL183W
YDL165W	YPR072W
YGL201C	YLR274W
YDR310C	YIL147C
YDR346C	YPR118W
YHR179W	YPL171C
YIL106W	YPR111W
YPL003W	YPR066W
YDR122W	YHR158C
YLR146C	YPR069C
YDL201W	YDR165W
YHR109W	YJR139C
