# Supplementary material for: A disease-related essential protein prediction model based on the transfer neural network
Source: Front Genet. 2023 Jan 4;13:1087294. doi: 10.3389/fgene.2022.1087294 (PMC9845409; doi:10.3389/fgene.2022.1087294)
Supplement: Supplementary file 4 [file DataSheet1.PDF]

Q0050	YER142C
Q0080	YBL099W
Q0080	YJR121W
Q0085	YBL099W
Q0085	YDR298C
Q0085	YDR377W
Q0085	YJR121W
Q0085	YKL016C
Q0085	YML081C-A
Q0085	YPL078C
Q0130	YBL099W
Q0130	YJR121W
Q0250	YBR024W
Q0250	YBR037C
Q0250	YER154W
Q0275	YER154W
R0020C	YBL056W
R0020C	YBR217W
R0020C	YDR523C
R0030W	YOR101W
R0030W	YOR351C
YAL001C	YBR123C
YAL002W	YCL018W
YAL002W	YDR171W
YAL002W	YGL206C
YAL002W	YKR026C
YAL002W	YLR148W
YAL002W	YLR396C
YAL002W	YMR231W
YAL002W	YPL045W
YAL003W	YBR118W
YAL003W	YKL081W
YAL003W	YML064C
YAL004W	YGL181W
YAL004W	YML109W
YAL005C	YAR002W
YAL005C	YCL028W
YAL005C	YDR192C
YAL005C	YHR069C
YAL005C	YLR310C
YAL005C	YLR335W
YAL005C	YLR347C
YAL005C	YNL007C
YAL005C	YNL064C
YAL005C	YNL077W

YAL005C	YOR098C
YAL005C	YOR151C
YAL005C	YOR160W
YAL005C	YPL240C
YAL005C	YPR010C
YAL007C	YAR002C-A
YAL007C	YBR036C
YAL007C	YBR183W
YAL007C	YCL025C
YAL007C	YDL054C
YAL007C	YDR087C
YAL007C	YDR101C
YAL007C	YDR127W
YAL007C	YEL002C
YAL007C	YEL063C
YAL007C	YER110C
YAL007C	YGL200C
YAL007C	YGR060W
YAL007C	YHR026W
YAL007C	YHR110W
YAL007C	YHR140W
YAL007C	YJL117W
YAL007C	YLR342W
YAL007C	YML012W
YAL007C	YMR290C
YAL007C	YNL130C
YAL007C	YPL076W
YAL007C	YPL211W
YAL007C	YPL264C
YAL007C	YPR201W
YAL009W	YDL040C
YAL009W	YDR211W
YAL009W	YHR004C
YAL009W	YJL176C
YAL009W	YLR310C
YAL009W	YLR342W
YAL010C	YFL048C
YAL010C	YJL066C
YAL010C	YMR203W
YAL010C	YPL235W
YAL011W	YBL007C
YAL011W	YDL181W
YAL011W	YDR225W
YAL011W	YDR334W
YAL011W	YER081W

YAL011W	YGR002C
YAL011W	YHR158C
YAL011W	YIR018W
YAL011W	YNL107W
YAL011W	YOL012C
YAL011W	YOR127W
YAL011W	YOR269W
YAL012W	YBR055C
YAL012W	YJR091C
YAL012W	YMR101C
YAL012W	YMR300C
YAL012W	YPL149W
YAL013W	YGL061C
YAL013W	YNL330C
YAL014C	YER133W
YAL014C	YIL034C
YAL015C	YBR020W
YAL015C	YBR127C
YAL015C	YCL018W
YAL015C	YCL064C
YAL015C	YDL029W
YAL015C	YDL059C
YAL015C	YDR214W
YAL015C	YEL030W
YAL015C	YEL060C
YAL015C	YFL037W
YAL015C	YFL039C
YAL015C	YGR282C
YAL015C	YJL066C
YAL015C	YJL088W
YAL015C	YJR045C
YAL015C	YJR068W
YAL015C	YJR077C
YAL015C	YML085C
YAL015C	YMR012W
YAL015C	YMR058W
YAL015C	YMR146C
YAL015C	YMR214W
YAL015C	YNL037C
YAL015C	YPR110C
YAL016W	YAL024C
YAL016W	YDL134C
YAL016W	YDL188C
YAL016W	YDR482C
YAL016W	YFR024C-A

YAL016W	YFR040W
YAL016W	YGL190C
YAL016W	YGR161C
YAL016W	YGR281W
YAL016W	YHR082C
YAL016W	YHR135C
YAL016W	YHR158C
YAL016W	YJL020C
YAL016W	YLR191W
YAL016W	YML109W
YAL016W	YMR109W
YAL016W	YMR273C
YAL016W	YNL127W
YAL016W	YNR032W
YAL016W	YOL113W
YAL016W	YOR014W
YAL016W	YPL152W
YAL017W	YCL027W
YAL017W	YDR099W
YAL017W	YDR190C
YAL017W	YER054C
YAL017W	YER133W
YAL017W	YFR053C
YAL017W	YGL112C
YAL017W	YKL035W
YAL017W	YLR258W
YAL017W	YMR205C
YAL017W	YOL045W
YAL017W	YPR160W
YAL018C	YBR036C
YAL018C	YBR290W
YAL018C	YCL025C
YAL018C	YDL017W
YAL018C	YDR331W
YAL018C	YHR026W
YAL018C	YHR114W
YAL018C	YLL028W
YAL018C	YLR034C
YAL018C	YLR264W
YAL018C	YMR001C
YAL019W	YBL088C
YAL019W	YBR084W
YAL019W	YBR160W
YAL019W	YDL117W
YAL019W	YJL124C

YAL019W	YOL045W
YAL021C	YBL099W
YAL021C	YBR020W
YAL021C	YBR072W
YAL021C	YBR127C
YAL021C	YCL018W
YAL021C	YCL028W
YAL021C	YCR093W
YAL021C	YDL165W
YAL021C	YDR171W
YAL021C	YDR188W
YAL021C	YDR190C
YAL021C	YDR443C
YAL021C	YER068W
YAL021C	YFL028C
YAL021C	YFL037W
YAL021C	YFL039C
YAL021C	YGL048C
YAL021C	YGL178W
YAL021C	YGL195W
YAL021C	YGR086C
YAL021C	YGR092W
YAL021C	YGR134W
YAL021C	YGR155W
YAL021C	YGR184C
YAL021C	YJL130C
YAL021C	YJR121W
YAL021C	YKR036C
YAL021C	YLL013C
YAL021C	YLR180W
YAL021C	YLR259C
YAL021C	YML085C
YAL021C	YMR214W
YAL021C	YNL025C
YAL021C	YNL064C
YAL021C	YNL071W
YAL021C	YNL288W
YAL021C	YNR052C
YAL021C	YOR027W
YAL021C	YPL042C
YAL021C	YPR072W
YAL022C	YBR159W
YAL022C	YDR497C
YAL022C	YGL132W
YAL022C	YHR140W

YAL022C	YJR117W
YAL023C	YAR027W
YAL024C	YBR160W
YAL024C	YHR030C
YAL024C	YHR158C
YAL024C	YIR009W
YAL025C	YJR044C
YAL025C	YOR005C
YAL025C	YOR267C
YAL026C	YAL053W
YAL026C	YDL192W
YAL026C	YPL146C
YAL027W	YBR196C
YAL027W	YCL024W
YAL027W	YDR174W
YAL027W	YDR381W
YAL027W	YDR507C
YAL027W	YLR044C
YAL027W	YML095C
YAL027W	YOL086C
YAL027W	YPL022W
YAL028W	YBL050W
YAL028W	YBR160W
YAL028W	YDL239C
YAL028W	YLR233C
YAL029C	YBR081C
YAL029C	YBR109C
YAL029C	YBR130C
YAL029C	YDR101C
YAL029C	YER110C
YAL029C	YFL039C
YAL029C	YGL106W
YAL029C	YGL195W
YAL029C	YHR023W
YAL029C	YKL130C
YAL029C	YLR249W
YAL029C	YMR139W
YAL029C	YMR186W
YAL029C	YMR309C
YAL029C	YOL086C
YAL029C	YOR181W
YAL030W	YDR468C
YAL030W	YGR009C
YAL031C	YBR160W
YAL031C	YCL027W

YAL031C	YER133W
YAL032C	YBR017C
YAL032C	YDL209C
YAL032C	YDR364C
YAL032C	YDR416W
YAL032C	YGL120C
YAL032C	YGL128C
YAL032C	YGR278W
YAL032C	YJL203W
YAL032C	YKL095W
YAL032C	YKL173W
YAL032C	YLL036C
YAL032C	YLR117C
YAL032C	YLR338W
YAL032C	YLR345W
YAL032C	YLR423C
YAL032C	YML049C
YAL032C	YMR213W
YAL032C	YMR288W
YAL032C	YNL189W
YAL032C	YOR036W
YAL032C	YPL151C
YAL032C	YPL213W
YAL033W	YBR167C
YAL033W	YJR115W
YAL034C	YBR198C
YAL034C	YER110C
YAL034C	YGR274C
YAL034C	YOL004W
YAL034C	YOL086C
YAL034W-A	YDL028C
YAL034W-A	YGL172W
YAL034W-A	YGR120C
YAL034W-A	YJR091C
YAL034W-A	YKL103C
YAL035W	YBR084W
YAL035W	YDL014W
YAL035W	YDR496C
YAL035W	YER036C
YAL035W	YER142C
YAL035W	YGL130W
YAL035W	YGR102C
YAL035W	YIL018W
YAL035W	YIR001C
YAL035W	YLR175W

YAL035W	YLR241W
YAL035W	YLR427W
YAL035W	YMR106C
YAL035W	YMR309C
YAL035W	YNL030W
YAL035W	YOR119C
YAL035W	YOR361C
YAL035W	YPR041W
YAL036C	YBR118W
YAL036C	YCL018W
YAL036C	YCR038C
YAL036C	YDL065C
YAL036C	YDL126C
YAL036C	YDR152W
YAL036C	YFL039C
YAL036C	YGR162W
YAL036C	YGR250C
YAL036C	YHR027C
YAL036C	YIL075C
YAL036C	YJL130C
YAL036C	YJL138C
YAL036C	YKL152C
YAL036C	YMR012W
YAL036C	YMR205C
YAL036C	YNR022C
YAL036C	YPL061W
YAL038W	YNL307C
YAL038W	YOR226C
YAL040C	YBR160W
YAL040C	YGL120C
YAL040C	YJL013C
YAL040C	YJL157C
YAL040C	YJR132W
YAL040C	YLL021W
YAL040C	YLR182W
YAL040C	YLR259C
YAL040C	YNL064C
YAL040C	YNL189W
YAL040C	YOR269W
YAL040C	YPL014W
YAL041W	YBL085W
YAL041W	YBR079C
YAL041W	YBR200W
YAL041W	YDR379W
YAL041W	YER114C

YAL041W	YGL052W
YAL041W	YGL127C
YAL041W	YGL233W
YAL041W	YGR152C
YAL041W	YGR221C
YAL041W	YGR268C
YAL041W	YHL007C
YAL041W	YIL079C
YAL041W	YJL008C
YAL041W	YJL157C
YAL041W	YJL187C
YAL041W	YKL130C
YAL041W	YKL172W
YAL041W	YLL008W
YAL041W	YLR206W
YAL041W	YLR229C
YAL041W	YLR357W
YAL041W	YMR298W
YAL041W	YNL007C
YAL041W	YNL118C
YAL041W	YNL175C
YAL041W	YOR212W
YAL041W	YPL161C
YAL041W	YPR129W
YAL042W	YDL239C
YAL042W	YML067C
YAL042W	YML130C
YAL043C	YDR195W
YAL043C	YDR228C
YAL043C	YDR301W
YAL043C	YDR311W
YAL043C	YER133W
YAL043C	YGR156W
YAL043C	YJL033W
YAL043C	YJR093C
YAL043C	YKL018W
YAL043C	YKL059C
YAL043C	YKR002W
YAL043C	YLR115W
YAL043C	YLR277C
YAL043C	YMR061W
YAL043C	YMR182C
YAL043C	YNL222W
YAL043C	YNL317W
YAL043C	YOR179C

YAL043C	YPR107C
YAL045C	YDR328C
YAL045C	YJR063W
YAL047C	YCL032W
YAL047C	YEL061C
YAL047C	YGL212W
YAL047C	YGR218W
YAL047C	YHR158C
YAL047C	YHR172W
YAL047C	YIL061C
YAL047C	YLR045C
YAL047C	YLR212C
YAL047C	YLR429W
YAL047C	YMR001C
YAL047C	YMR047C
YAL047C	YNL126W
YAL047C	YNL188W
YAL047C	YPL093W
YAL048C	YER114C
YAL049C	YDL025C
YAL049C	YNL094W
YAL051W	YOR363C
YAL053W	YER100W
YAL053W	YLR083C
YAL053W	YLR292C
YAL053W	YLR342W
YAL053W	YML072C
YAL053W	YOR153W
YAL054C	YHR041C
YAL054C	YLR049C
YAL054C	YNL189W
YAL055W	YDL065C
YAL055W	YDR265W
YAL055W	YGR133W
YAL055W	YMR026C
YAL055W	YOL044W
YAL055W	YOR098C
YAL056W	YER020W
YAL056W	YGL121C
YAL058W	YIL120W
YAL058W	YOR236W
YAL059W	YBR115C
YAL059W	YDR395W
YAL059W	YER110C
YAL059W	YER165W

YAL059W	YGL195W
YAL059W	YMR186W
YAL059W	YMR308C
YAL059W	YOL086C
YAL060W	YER081W
YAL061W	YMR308C
YAL062W	YLR267W
YAL062W	YNL189W
YAL063C-A	YPR154W
YAL064W	YHR114W
YAL064W	YKR011C
YAL064W	YMR149W
YAL064W-B	YPR126C
YAL066W	YJR091C
YAL067C	YER118C
YAL067C	YGR260W
YAL067C	YHR042W
YAL069W	YMR032W
YAR002C-A	YGL200C
YAR002C-A	YGR132C
YAR002C-A	YJR091C
YAR002C-A	YML012W
YAR002C-A	YNL030W
YAR002W	YBR160W
YAR002W	YCL027W
YAR002W	YDL229W
YAR002W	YER110C
YAR002W	YER165W
YAR002W	YGL097W
YAR002W	YLR293C
YAR002W	YLR335W
YAR002W	YLR347C
YAR002W	YMR047C
YAR002W	YNL189W
YAR002W	YOR133W
YAR003W	YBL026W
YAR003W	YBL099W
YAR003W	YBR175W
YAR003W	YBR258C
YAR003W	YCL040W
YAR003W	YDR069C
YAR003W	YDR140W
YAR003W	YDR469W
YAR003W	YER081W
YAR003W	YFR053C

YAR003WYHR119W
YAR003WYIL148W
YAR003WYJL026W
YAR003WYJL141C
YAR003WYJR113C
YAR003WYKL018W
YAR003WYKL035W
YAR003WYLR015W
YAR003WYPL138C
YAR007C YBL099W
YAR007C YBR020W
YAR007C YBR072W
YAR007C YBR089C-A
YAR007C YBR127C
YAR007C YBR136W
YAR007C YBR196C
YAR007C YCR012W
YAR007C YDL007W
YAR007C YDL029W
YAR007C YDR034C
YAR007C YDR097C
YAR007C YDR190C
YAR007C YER078C
YAR007C YER095W
YAR007C YFL037W
YAR007C YFL039C
YAR007C YGL048C
YAR007C YGL206C
YAR007C YHR164C
YAR007C YIR002C
YAR007C YJL034W
YAR007C YJL088W
YAR007C YJL130C
YAR007C YJL173C
YAR007C YKL152C
YAR007C YLR304C
YAR007C YLR347C
YAR007C YML032C
YAR007C YML085C
YAR007C YML124C
YAR007C YMR116C
YAR007C YMR214W
YAR007C YMR234W
YAR007C YNL312W
YAR007C YOL090W

YAR007C YPL240C
YAR008WYBL051C
YAR008WYJR091C
YAR008WYLR105C
YAR008WYMR059W
YAR008WYPL083C
YAR014C YBR186W
YAR014C YCL028W
YAR014C YCL031C
YAR014C YDL070W
YAR014C YDL240W
YAR014C YDR010C
YAR014C YDR085C
YAR014C YDR100W
YAR014C YDR107C
YAR014C YDR122W
YAR014C YDR195W
YAR014C YDR259C
YAR014C YDR277C
YAR014C YDR480W
YAR014C YER032W
YAR014C YER054C
YAR014C YER115C
YAR014C YER133W
YAR014C YER155C
YAR014C YER158C
YAR014C YER167W
YAR014C YFL059W
YAR014C YFR040W
YAR014C YGL237C
YAR014C YGR037C
YAR014C YGR225W
YAR014C YGR285C
YAR014C YHL048W
YAR014C YHR064C
YAR014C YIL045W
YAR014C YIL144W
YAR014C YJL042W
YAR014C YJL187C
YAR014C YJR115W
YAR014C YKR075C
YAR014C YLR096W
YAR014C YLR098C
YAR014C YLR108C
YAR014C YLR245C

YAR014C YLR273C
YAR014C YLR430W
YAR014C YML052W
YAR014C YMR018W
YAR014C YMR026C
YAR014C YMR071C
YAR014C YMR104C
YAR014C YMR116C
YAR014C YMR258C
YAR014C YNL025C
YAR014C YNL044W
YAR014C YNL206C
YAR014C YNL229C
YAR014C YNL333W
YAR014C YOL033W
YAR014C YOL154W
YAR014C YOR047C
YAR014C YOR062C
YAR014C YOR178C
YAR014C YOR229W
YAR014C YOR284W
YAR014C YOR329C
YAR014C YOR355W
YAR014C YOR358W
YAR014C YPL049C
YAR014C YPL204W
YAR018C YBL106C
YAR018C YDL239C
YAR018C YDR216W
YAR018C YGL181W
YAR018C YGR032W
YAR018C YGR285C
YAR018C YHR064C
YAR018C YIL061C
YAR018C YIL070C
YAR018C YJL098W
YAR018C YLR342W
YAR018C YML006C
YAR018C YML064C
YAR018C YMR116C
YAR018C YNL077W
YAR018C YOL054W
YAR018C YOL083W
YAR018C YOR167C
YAR018C YPL093W

YAR019C YBR118W
YAR019C YDL047W
YAR019C YDL126C
YAR019C YDL185W
YAR019C YDR099W
YAR019C YER110C
YAR019C YGR092W
YAR019C YHL007C
YAR019C YHR030C
YAR019C YHR169W
YAR019C YJR045C
YAR019C YML064C
YAR019C YMR205C
YAR019C YNL223W
YAR023C YDL217C
YAR023C YHL048W
YAR027WYAR028W
YAR027WYAR030C
YAR027WYBR106W
YAR027WYBR135W
YAR027WYBR159W
YAR027WYCL073C
YAR027WYDL198C
YAR027WYDR406W
YAR027WYEL041W
YAR027WYEL063C
YAR027WYGL129C
YAR027WYGR014W
YAR027WYGR060W
YAR027WYGR284C
YAR027WYHR048W
YAR027WYIL004C
YAR027WYIL016W
YAR027WYIL111W
YAR027WYIR033W
YAR027WYJL002C
YAR027WYJL093C
YAR027WYJR040W
YAR027WYKL196C
YAR027WYKR105C
YAR027WYLR018C
YAR027WYLR214W
YAR027WYML038C
YAR027WYMR149W
YAR027WYMR264W

YAR027WYMR279C
YAR027WYNL125C
YAR027WYOL030W
YAR027WYOL156W
YAR027WYOR273C
YAR027WYPL094C
YAR027WYPR128C
YAR028WYBR159W
YAR028WYDR331W
YAR028WYGL104C
YAR028WYGR055W
YAR028WYHR026W
YAR028WYJL219W
YAR028WYML067C
YAR028WYML129C
YAR028WYPL264C
YAR028WYPR128C
YAR030C YJR091C
YAR031WYBR069C
YAR031WYBR217W
YAR031WYCL025C
YAR031WYCR030C
YAR031WYDL054C
YAR031WYHR110W
YAR031WYJR015W
YAR031WYJR091C
YAR031WYLL028W
YAR033WYGL051W
YAR033WYGR260W
YAR033WYHL048W
YAR033WYKL174C
YAR033WYLL028W
YAR033WYPL076W
YAR033WYPR198W
YAR035WYDR328C
YAR035WYGR069W
YAR035WYLR295C
YAR035WYLR381W
YAR035WYLR447C
YAR042WYER120W
YAR042WYGR192C
YAR042WYLR191W
YAR042WYLR397C
YAR042WYMR047C
YAR047C YJR063W

YAR064W	YBR109C
YAR064W	YGL070C
YAR066W	YDR074W
YAR066W	YLR145W
YAR066W	YMR047C
YAR066W	YPR086W
YAR068W	YMR032W
YAR069C	YPL215W
YAR071W	YLR453C
YAR073W	YDL213C
YAR073W	YLR383W
YAR073W	YOL115W
YBL001C	YDR510W
YBL001C	YER067W
YBL001C	YNL189W
YBL001C	YPL068C
YBL002W	YBR114W
YBL002W	YBR245C
YBL002W	YCR040W
YBL002W	YDL213C
YBL002W	YDR121W
YBL002W	YDR496C
YBL002W	YER142C
YBL002W	YGL241W
YBL002W	YGR054W
YBL002W	YGR067C
YBL002W	YHR197W
YBL002W	YIL035C
YBL002W	YIL126W
YBL002W	YJL020C
YBL002W	YJR090C
YBL002W	YKR001C
YBL002W	YKR048C
YBL002W	YLR074C
YBL002W	YMR036C
YBL002W	YMR091C
YBL002W	YNL068C
YBL002W	YNL157W
YBL002W	YNL262W
YBL002W	YOL006C
YBL002W	YOL054W
YBL002W	YOL087C
YBL002W	YOL108C
YBL002W	YOR116C
YBL002W	YOR207C

YBL002W	YOR304W
YBL002W	YPL082C
YBL002W	YPR010C
YBL002W	YPR175W
YBL002W	YPR190C
YBL003C	YBR017C
YBL003C	YCR057C
YBL003C	YDL188C
YBL003C	YOL004W
YBL003C	YOL054W
YBL003C	YPL153C
YBL004W	YBL038W
YBL004W	YBR143C
YBL004W	YBR247C
YBL004W	YBR251W
YBL004W	YCL059C
YBL004W	YCR057C
YBL004W	YDL213C
YBL004W	YDR060W
YBL004W	YDR280W
YBL004W	YER082C
YBL004W	YER100W
YBL004W	YGR090W
YBL004W	YGR220C
YBL004W	YHR089C
YBL004W	YJL095W
YBL004W	YKR086W
YBL004W	YLR074C
YBL004W	YNL030W
YBL004W	YNL110C
YBL004W	YNL284C
YBL004W	YOL139C
YBL004W	YPL043W
YBL004W	YPR137W
YBL005W	YBR200W
YBL005W	YCR009C
YBL005W	YCR088W
YBL005W	YDR176W
YBL005W	YDR388W
YBL005W-B	YMR032W
YBL006C	YDR489W
YBL007C	YBL037W
YBL007C	YBL047C
YBL007C	YBL088C
YBL007C	YBL105C

YBL007C	YBR059C
YBL007C	YBR098W
YBL007C	YBR108W
YBL007C	YBR136W
YBL007C	YBR150C
YBL007C	YBR205W
YBL007C	YBR250W
YBL007C	YCL008C
YBL007C	YCL031C
YBL007C	YCL034W
YBL007C	YCL045C
YBL007C	YCR030C
YBL007C	YCR088W
YBL007C	YCR095C
YBL007C	YCR100C
YBL007C	YDL002C
YBL007C	YDL028C
YBL007C	YDL091C
YBL007C	YDL117W
YBL007C	YDL139C
YBL007C	YDL146W
YBL007C	YDL203C
YBL007C	YDL206W
YBL007C	YDR172W
YBL007C	YDR271C
YBL007C	YDR277C
YBL007C	YDR382W
YBL007C	YDR388W
YBL007C	YDR423C
YBL007C	YDR520C
YBL007C	YEL068C
YBL007C	YER014W
YBL007C	YER025W
YBL007C	YER027C
YBL007C	YER110C
YBL007C	YER118C
YBL007C	YER133W
YBL007C	YER151C
YBL007C	YFL010C
YBL007C	YFR013W
YBL007C	YFR024C-A
YBL007C	YGL035C
YBL007C	YGL104C
YBL007C	YGL181W
YBL007C	YGL201C

YBL007C	YGL215W
YBL007C	YGL237C
YBL007C	YGL238W
YBL007C	YGR166W
YBL007C	YGR227W
YBL007C	YGR240C
YBL007C	YGR268C
YBL007C	YHL002W
YBL007C	YHL016C
YBL007C	YHR016C
YBL007C	YHR114W
YBL007C	YIL076W
YBL007C	YIL095W
YBL007C	YIL144W
YBL007C	YIL156W
YBL007C	YIL173W
YBL007C	YIR003W
YBL007C	YIR006C
YBL007C	YIR033W
YBL007C	YJL004C
YBL007C	YJL020C
YBL007C	YJL145W
YBL007C	YJL151C
YBL007C	YJL201W
YBL007C	YJR113C
YBL007C	YJR115W
YBL007C	YKL054C
YBL007C	YKL074C
YBL007C	YKR022C
YBL007C	YKR030W
YBL007C	YKR095W
YBL007C	YLL005C
YBL007C	YLL018C
YBL007C	YLR064W
YBL007C	YLR078C
YBL007C	YLR088W
YBL007C	YLR095C
YBL007C	YLR106C
YBL007C	YLR144C
YBL007C	YLR150W
YBL007C	YLR191W
YBL007C	YLR206W
YBL007C	YLR315W
YBL007C	YLR337C
YBL007C	YLR363W-A

YBL007C	YLR430W
YBL007C	YML075C
YBL007C	YML097C
YBL007C	YML109W
YBL007C	YML116W
YBL007C	YMR109W
YBL007C	YMR147W
YBL007C	YMR192W
YBL007C	YMR253C
YBL007C	YMR313C
YBL007C	YNL020C
YBL007C	YNL084C
YBL007C	YNL094W
YBL007C	YNL106C
YBL007C	YNL116W
YBL007C	YNL138W
YBL007C	YNL144C
YBL007C	YNL152W
YBL007C	YNL161W
YBL007C	YNL176C
YBL007C	YNL229C
YBL007C	YNL285W
YBL007C	YNR041C
YBL007C	YOL028C
YBL007C	YOR047C
YBL007C	YOR109W
YBL007C	YOR181W
YBL007C	YOR211C
YBL007C	YOR284W
YBL007C	YOR322C
YBL007C	YOR329C
YBL007C	YOR388C
YBL007C	YPL012W
YBL007C	YPL022W
YBL007C	YPL027W
YBL007C	YPL084W
YBL007C	YPL157W
YBL007C	YPL246C
YBL007C	YPL249C
YBL007C	YPR030W
YBL007C	YPR048W
YBL007C	YPR091C
YBL007C	YPR159W
YBL007C	YPR171W
YBL008W	YBR289W

YBL008W YCL018W
YBL008W YER082C
YBL008W YGR180C
YBL008W YJL115W
YBL008W YJL176C
YBL008W YOR038C
YBL008W YOR290C
YBL009W YDL053C
YBL009W YER155C
YBL009W YGL127C
YBL009W YGR090W
YBL009W YHR121W
YBL009W YKL203C
YBL009W YLR196W
YBL009W YOL041C
YBL009W YOR017W
YBL009W YOR233W
YBL010C YKR022C
YBL013W YBR160W
YBL014C YHL004W
YBL014C YJL025W
YBL014C YOR158W
YBL015W YJR035W
YBL015W YJR068W
YBL015W YMR059W
YBL015W YPR110C
YBL016W YBL085W
YBL016W YBR083W
YBL016W YDL117W
YBL016W YDL159W
YBL016W YDR103W
YBL016W YDR216W
YBL016W YDR469W
YBL016W YDR480W
YBL016W YER075C
YBL016W YER114C
YBL016W YER167W
YBL016W YFR028C
YBL016W YGL057C
YBL016W YGL158W
YBL016W YGL178W
YBL016W YGR097W
YBL016W YHR005C
YBL016W YHR084W
YBL016W YHR168W

YBL016W YIL169C
YBL016W YJL076W
YBL016W YLR342W
YBL016W YLR362W
YBL016W YML117W
YBL016W YMR295C
YBL016W YNL053W
YBL016W YOL130W
YBL016W YOR119C
YBL016W YPL049C
YBL016W YPR115W
YBL017C YDR432W
YBL017C YLR310C
YBL017C YMR197C
YBL017C YOR181W
YBL017C YPL049C
YBL018C YBR167C
YBL018C YHR041C
YBL019W YEL009C
YBL020W YPR086W
YBL021C YBL075C
YBL021C YCL018W
YBL021C YDL185W
YBL021C YDR502C
YBL021C YER103W
YBL021C YGL237C
YBL021C YHL006C
YBL021C YJR045C
YBL021C YJR121W
YBL021C YML048W
YBL021C YNL064C
YBL021C YOR358W
YBL022C YGR040W
YBL022C YIL061C
YBL022C YLR180W
YBL022C YML029W
YBL022C YOR136W
YBL023C YBR126C
YBL023C YDL017W
YBL023C YDL171C
YBL023C YDR052C
YBL023C YEL032W
YBL023C YIL150C
YBL023C YJL194W
YBL023C YLR103C

YBL023C YLR274W
YBL023C YOR080W
YBL023C YOR196C
YBL023C YPL001W
YBL023C YPR019W
YBL023C YPR120C
YBL024W YBR084W
YBL024W YDR382W
YBL024W YGR237C
YBL024W YNL284C
YBL025W YBR274W
YBL025W YDL113C
YBL025W YDR247W
YBL025W YER178W
YBL025W YFR028C
YBL025W YHR200W
YBL025W YIL141W
YBL025W YJR087W
YBL025W YKL135C
YBL025W YMR270C
YBL026W YBL066C
YBL026W YBL099W
YBL026W YBR019C
YBL026W YBR118W
YBL026W YCL018W
YBL026W YCL064C
YBL026W YCR020C-A
YBL026W YCR066W
YBL026W YCR077C
YBL026W YDL055C
YBL026W YDL097C
YBL026W YDL160C
YBL026W YDL175C
YBL026W YDR166C
YBL026W YDR378C
YBL026W YDR440W
YBL026W YEL015W
YBL026W YER025W
YBL026W YER112W
YBL026W YER146W
YBL026W YFL037W
YBL026W YFL039C
YBL026W YFR053C
YBL026W YGL173C
YBL026W YGL185C

YBL026W YGL234W
YBL026W YGR077C
YBL026W YGR091W
YBL026W YGR158C
YBL026W YGR282C
YBL026W YHR165C
YBL026W YIL048W
YBL026W YIL066C
YBL026W YIL132C
YBL026W YJL008C
YBL026W YJL066C
YBL026W YJL124C
YBL026W YJL157C
YBL026W YJR022W
YBL026W YJR077C
YBL026W YJR121W
YBL026W YLR039C
YBL026W YLR074C
YBL026W YLR120C
YBL026W YLR126C
YBL026W YLR128W
YBL026W YLR147C
YBL026W YLR264W
YBL026W YLR275W
YBL026W YLR438C-A
YBL026W YML085C
YBL026W YMR066W
YBL026W YMR207C
YBL026W YMR237W
YBL026W YMR268C
YBL026W YNL118C
YBL026W YNL147W
YBL026W YNL264C
YBL026W YNL287W
YBL026W YOL102C
YBL026W YOL149W
YBL026W YOR017W
YBL026W YOR043W
YBL026W YOR191W
YBL026W YPL042C
YBL026W YPL115C
YBL026W YPL119C
YBL026W YPL249C-A
YBL026W YPR032W
YBL026W YPR132W

YBL026W YPR184W
YBL027W YDR388W
YBL027W YGR069W
YBL027W YHR114W
YBL027W YJL020C
YBL027W YMR032W
YBL029W YKR036C
YBL030C YBL050W
YBL030C YBR017C
YBL030C YBR109C
YBL030C YBR217W
YBL030C YDL059C
YBL030C YDL132W
YBL030C YDR388W
YBL030C YEL020W-A
YBL030C YER100W
YBL030C YER161C
YBL030C YER171W
YBL030C YFR028C
YBL030C YGL137W
YBL030C YGL237C
YBL030C YGR040W
YBL030C YGR082W
YBL030C YGR262C
YBL030C YHR005C-A
YBL030C YHR133C
YBL030C YHR166C
YBL030C YHR197W
YBL030C YJL173C
YBL030C YKR026C
YBL030C YLR233C
YBL030C YLR268W
YBL030C YML058W
YBL030C YMR059W
YBL030C YMR106C
YBL030C YOR181W
YBL030C YPL150W
YBL030C YPL194W
YBL031W YGR218W
YBL032W YBR084W
YBL032W YFR012W
YBL032W YGL049C
YBL032W YGR162W
YBL032W YLR150W
YBL032W YLR175W

YBL032W YML105C
YBL032W YML117W
YBL032W YMR125W
YBL032W YMR310C
YBL032W YNL005C
YBL032W YNL030W
YBL032W YNL112W
YBL032W YOL139C
YBL032W YPL237W
YBL033C YNL105W
YBL033C YPR172W
YBL034C YDR330W
YBL034C YER177W
YBL034C YFL037W
YBL034C YNL118C
YBL035C YBR084W
YBL035C YBR087W
YBL035C YDL058W
YBL035C YIR008C
YBL035C YKL045W
YBL035C YNL102W
YBL035C YNL290W
YBL035C YNR052C
YBL035C YOL094C
YBL035C YOR217W
YBL036C YBL099W
YBL036C YBR196C
YBL036C YCL040W
YBL036C YDR023W
YBL036C YDR226W
YBL036C YDR342C
YBL036C YDR343C
YBL036C YFR053C
YBL036C YGR087C
YBL036C YMR319C
YBL036C YOL055C
YBL036C YOL078W
YBL036C YPL061W
YBL037W YBL105C
YBL037W YJR005W
YBL037W YJR058C
YBL037W YNL118C
YBL037W YOL062C
YBL038W YCL014W
YBL038W YDR164C

YBL038W	YDR237W
YBL038W	YDR322W
YBL038W	YDR462W
YBL038W	YER155C
YBL038W	YGR033C
YBL038W	YJL005W
YBL038W	YJL063C
YBL038W	YJL176C
YBL038W	YJR138W
YBL038W	YKR085C
YBL038W	YLR189C
YBL038W	YLR439W
YBL038W	YML025C
YBL038W	YMR024W
YBL038W	YNL005C
YBL038W	YNL284C
YBL038W	YOR150W
YBL038W	YOR290C
YBL038W	YPR100W
YBL039C	YBR017C
YBL039C	YDL029W
YBL039C	YDL047W
YBL039C	YDR260C
YBL039C	YDR388W
YBL039C	YDR394W
YBL039C	YEL056W
YBL039C	YER179W
YBL039C	YFR004W
YBL039C	YGL048C
YBL039C	YGL081W
YBL039C	YGR262C
YBL039C	YHR200W
YBL039C	YIL094C
YBL039C	YJL138C
YBL039C	YJR103W
YBL039C	YJR109C
YBL039C	YKL196C
YBL039C	YKR026C
YBL039C	YLR180W
YBL039C	YLR291C
YBL039C	YMR012W
YBL039C	YMR024W
YBL039C	YMR117C
YBL039C	YNR031C
YBL039C	YOL062C

YBL039C	YOL126C
YBL039C	YOL139C
YBL039C	YOR117W
YBL039C	YOR151C
YBL039C	YOR261C
YBL039C	YOR351C
YBL039C	YOR388C
YBL039C	YPL070W
YBL039C	YPL140C
YBL039C	YPL259C
YBL039C	YPR110C
YBL040C	YDR331W
YBL040C	YDR414C
YBL040C	YEL063C
YBL040C	YGR060W
YBL040C	YJR010C-A
YBL040C	YMR215W
YBL040C	YOL020W
YBL040C	YPR156C
YBL041W	YCR073C
YBL041W	YDL020C
YBL041W	YDL147W
YBL041W	YDL188C
YBL041W	YER012W
YBL041W	YER105C
YBL041W	YHR200W
YBL041W	YIL007C
YBL041W	YPR103W
YBL042C	YER021W
YBL042C	YNL121C
YBL043W	YBL100W-A
YBL043W	YDR099W
YBL044W	YJL159W
YBL044W	YLR074C
YBL044W	YMR304W
YBL045C	YDL179W
YBL045C	YDR339C
YBL045C	YDR388W
YBL045C	YGL137W
YBL045C	YIL061C
YBL045C	YKL095W
YBL045C	YLR090W
YBL045C	YLR442C
YBL045C	YML064C
YBL045C	YMR036C

YBL045C	YMR047C
YBL045C	YMR059W
YBL045C	YNL118C
YBL045C	YOR125C
YBL045C	YPL204W
YBL045C	YPR054W
YBL045C	YPR191W
YBL046W	YDL112W
YBL046W	YDR075W
YBL046W	YML010W
YBL046W	YNL201C
YBL047C	YBL105C
YBL047C	YBR059C
YBL047C	YBR109C
YBL047C	YCR030C
YBL047C	YDR192C
YBL047C	YDR348C
YBL047C	YMR066W
YBL047C	YNL243W
YBL047C	YOL016C
YBL047C	YOL054W
YBL047C	YOL087C
YBL047C	YPL153C
YBL047C	YPL204W
YBL049W	YBR020W
YBL049W	YBR072W
YBL049W	YBR118W
YBL049W	YCL039W
YBL049W	YDL143W
YBL049W	YDR255C
YBL049W	YER066C-A
YBL049W	YGL206C
YBL049W	YGL227W
YBL049W	YGR172C
YBL049W	YHL010C
YBL049W	YHR082C
YBL049W	YIL017C
YBL049W	YIL034C
YBL049W	YIL097W
YBL049W	YIL142W
YBL049W	YJL014W
YBL049W	YJL111W
YBL049W	YJR091C
YBL049W	YLR180W
YBL049W	YMR135C

YBL049W YNL064C
YBL050W YBR272C
YBL050W YCR082W
YBL050W YDL133W
YBL050W YDL173W
YBL050W YDR160W
YBL050W YDR189W
YBL050W YDR468C
YBL050W YER101C
YBL050W YER133W
YBL050W YGL095C
YBL050W YGR009C
YBL050W YIL084C
YBL050W YJR063W
YBL050W YKL090W
YBL050W YKL173W
YBL050W YKL196C
YBL050W YLL009C
YBL050W YLR026C
YBL050W YLR093C
YBL050W YLR117C
YBL050W YLR130C
YBL050W YLR268W
YBL050W YLR273C
YBL050W YMR197C
YBL050W YNL204C
YBL050W YOL018C
YBL050W YOL032W
YBL050W YOL036W
YBL050W YOL135C
YBL050W YOR036W
YBL050W YOR106W
YBL050W YOR158W
YBL050W YOR245C
YBL050W YOR307C
YBL050W YPL192C
YBL051C YGL127C
YBL051C YIL151C
YBL051C YJL138C
YBL051C YKR096W
YBL051C YPL204W
YBL051C YPL235W
YBL052C YBR274W
YBL052C YGL207W
YBL052C YNL258C

YBL052C	YOR064C
YBL052C	YPL129W
YBL053W	YEL069C
YBL053W	YJR091C
YBL053W	YPR165W
YBL054W	YNL118C
YBL056W	YBR039W
YBL056W	YBR097W
YBL056W	YBR247C
YBL056W	YCL018W
YBL056W	YCL064C
YBL056W	YCR009C
YBL056W	YDL059C
YBL056W	YDL060W
YBL056W	YDL145C
YBL056W	YDR071C
YBL056W	YDR076W
YBL056W	YDR148C
YBL056W	YGL173C
YBL056W	YGR205W
YBL056W	YGR240C
YBL056W	YHL030W
YBL056W	YJL088W
YBL056W	YJR077C
YBL056W	YJR121W
YBL056W	YMR246W
YBL056W	YNL064C
YBL056W	YNL250W
YBL056W	YOR020C
YBL056W	YOR086C
YBL056W	YPL204W
YBL058W	YBR114W
YBL058W	YBR223C
YBL058W	YDL126C
YBL058W	YDL190C
YBL058W	YKL213C
YBL058W	YOR098C
YBL059W	YCR099C
YBL059W	YJR091C
YBL061C	YBR023C
YBL061C	YDR247W
YBL061C	YEL015W
YBL061C	YIL007C
YBL061C	YNL233W
YBL063W	YIL061C

YBL064C	YDR069C
YBL064C	YDR488C
YBL064C	YLR310C
YBL064C	YNR031C
YBL065W	YER022W
YBL066C	YDR099W
YBL066C	YDR247W
YBL066C	YEL015W
YBL066C	YGR117C
YBL066C	YPL026C
YBL066C	YPR154W
YBL067C	YHL002W
YBL067C	YJL203W
YBL068W	YHL011C
YBL068W	YKL181W
YBL068W	YOL061W
YBL071C	YCL027W
YBL071C	YNL261W
YBL072C	YBR135W
YBL072C	YDR459C
YBL072C	YFL017C
YBL072C	YLR140W
YBL072C	YMR047C
YBL074C	YDL208W
YBL074C	YDR283C
YBL074C	YHR165C
YBL074C	YKL173W
YBL075C	YBR017C
YBL075C	YBR055C
YBL075C	YCR002C
YBL075C	YDL047W
YBL075C	YDL132W
YBL075C	YDL145C
YBL075C	YDL225W
YBL075C	YDR128W
YBL075C	YDR142C
YBL075C	YDR328C
YBL075C	YGL004C
YBL075C	YGL130W
YBL075C	YGL190C
YBL075C	YGR092W
YBL075C	YHR135C
YBL075C	YHR186C
YBL075C	YHR199C
YBL075C	YIL142W

YBL075C	YJL128C
YBL075C	YKR026C
YBL075C	YKR036C
YBL075C	YLR291C
YBL075C	YLR310C
YBL075C	YLR429W
YBL075C	YNL007C
YBL075C	YNL077W
YBL075C	YNL135C
YBL075C	YOL133W
YBL075C	YOL139C
YBL075C	YOR212W
YBL075C	YOR250C
YBL075C	YPL259C
YBL076C	YBR055C
YBL076C	YBR058C
YBL076C	YBR109C
YBL076C	YDL100C
YBL076C	YLR016C
YBL076C	YNL244C
YBL076C	YOR080W
YBL076C	YPR110C
YBL078C	YHR171W
YBL078C	YIL004C
YBL078C	YLR093C
YBL078C	YNL223W
YBL078C	YNR007C
YBL078C	YOL083W
YBL078C	YOR100C
YBL078C	YOR106W
YBL079W	YBR200W
YBL079W	YGR052W
YBL079W	YHR135C
YBL079W	YJL061W
YBL079W	YKL068W
YBL079W	YOR098C
YBL080C	YCR106W
YBL080C	YMR104C
YBL081W	YDL167C
YBL081W	YOR167C
YBL082C	YKL210W
YBL084C	YDL008W
YBL084C	YDR118W
YBL084C	YFR036W
YBL084C	YGL240W

YBL084C	YHR166C
YBL084C	YKL022C
YBL084C	YLR127C
YBL084C	YNL172W
YBL084C	YOR249C
YBL085W	YBR003W
YBL085W	YBR102C
YBL085W	YBR108W
YBL085W	YBR200W
YBL085W	YBR238C
YBL085W	YCL024W
YBL085W	YCL031C
YBL085W	YDL155W
YBL085W	YDL176W
YBL085W	YDR036C
YBL085W	YDR085C
YBL085W	YDR239C
YBL085W	YDR277C
YBL085W	YDR329C
YBL085W	YDR332W
YBL085W	YDR372C
YBL085W	YDR480W
YBL085W	YER008C
YBL085W	YER028C
YBL085W	YER032W
YBL085W	YER081W
YBL085W	YER124C
YBL085W	YER155C
YBL085W	YER158C
YBL085W	YER164W
YBL085W	YFR028C
YBL085W	YFR034C
YBL085W	YGL035C
YBL085W	YGL190C
YBL085W	YGL201C
YBL085W	YGR052W
YBL085W	YHL007C
YBL085W	YHL014C
YBL085W	YIL144W
YBL085W	YIR018W
YBL085W	YJL155C
YBL085W	YJR056C
YBL085W	YJR061W
YBL085W	YJR138W
YBL085W	YKL125W

YBL085W YKL218C
YBL085W YLL040C
YBL085W YLR096W
YBL085W YLR229C
YBL085W YLR314C
YBL085W YML109W
YBL085W YMR032W
YBL085W YMR124W
YBL085W YMR139W
YBL085W YMR244W
YBL085W YMR273C
YBL085W YNL025C
YBL085W YNL078W
YBL085W YNL206C
YBL085W YNL234W
YBL085W YNR016C
YBL085W YOL019W
YBL085W YOL070C
YBL085W YOL135C
YBL085W YOL156W
YBL085W YOR047C
YBL085W YPL038W
YBL085W YPL049C
YBL085W YPL115C
YBL085W YPL158C
YBL086C YJR040W
YBL086C YLR322W
YBL088C YLR025W
YBL088C YLR191W
YBL088C YNL030W
YBL088C YNL265C
YBL088C YPL110C
YBL089W YBR068C
YBL089W YJR091C
YBL089W YLR325C
YBL089W YOR082C
YBL089W YPL022W
YBL090W YBR251W
YBL090W YHL004W
YBL091C YNL290W
YBL092W YDR026C
YBL092W YDR398W
YBL092W YHR197W
YBL093C YBR193C
YBL093C YCR081W

YBL093C	YDR308C
YBL093C	YDR443C
YBL093C	YER022W
YBL093C	YGL025C
YBL093C	YGL151W
YBL093C	YGR104C
YBL093C	YHR041C
YBL093C	YHR058C
YBL093C	YHR147C
YBL093C	YKL129C
YBL093C	YLR071C
YBL093C	YML007W
YBL093C	YMR109W
YBL093C	YMR112C
YBL093C	YNL236W
YBL093C	YNR010W
YBL093C	YOL051W
YBL093C	YOL135C
YBL093C	YOR140W
YBL093C	YOR174W
YBL093C	YPL042C
YBL093C	YPR070W
YBL093C	YPR168W
YBL094C	YJR091C
YBL094C	YMR047C
YBL095W	YBR162C
YBL095W	YLR453C
YBL097W	YLR373C
YBL098W	YDL226C
YBL099W	YBR017C
YBL099W	YBR039W
YBL099W	YBR088C
YBL099W	YBR114W
YBL099W	YBR217W
YBL099W	YBR223C
YBL099W	YBR264C
YBL099W	YBR288C
YBL099W	YCR001W
YBL099W	YCR014C
YBL099W	YCR079W
YBL099W	YDL004W
YBL099W	YDL013W
YBL099W	YDL017W
YBL099W	YDL029W
YBL099W	YDL132W

YBL099W YDL147W
YBL099W YDL164C
YBL099W YDL181W
YBL099W YDL200C
YBL099W YDR075W
YBL099W YDR131C
YBL099W YDR143C
YBL099W YDR200C
YBL099W YDR227W
YBL099W YDR298C
YBL099W YDR322C-A
YBL099W YDR377W
YBL099W YDR419W
YBL099W YDR523C
YBL099W YER059W
YBL099W YER100W
YBL099W YER133W
YBL099W YER142C
YBL099W YER161C
YBL099W YER171W
YBL099W YER173W
YBL099W YFR028C
YBL099W YGL081W
YBL099W YGL131C
YBL099W YGL137W
YBL099W YGL163C
YBL099W YGR040W
YBL099W YGR092W
YBL099W YGR123C
YBL099W YGR240C
YBL099W YGR262C
YBL099W YHR030C
YBL099W YHR135C
YBL099W YHR166C
YBL099W YIL007C
YBL099W YIL066C
YBL099W YJL044C
YBL099W YJL087C
YBL099W YJL098W
YBL099W YJL106W
YBL099W YJL173C
YBL099W YJL180C
YBL099W YJR121W
YBL099W YKL016C
YBL099W YKL193C

YBL099W YKL215C
YBL099W YKR036C
YBL099W YLL019C
YBL099W YLR148W
YBL099W YLR186W
YBL099W YLR222C
YBL099W YLR238W
YBL099W YLR295C
YBL099W YLR320W
YBL099W YLR340W
YBL099W YLR442C
YBL099W YML064C
YBL099W YML081C-A
YBL099W YMR059W
YBL099W YMR106C
YBL099W YMR284W
YBL099W YNL012W
YBL099W YNL061W
YBL099W YNL090W
YBL099W YNL113W
YBL099W YNL182C
YBL099W YNL290W
YBL099W YNL311C
YBL099W YNL315C
YBL099W YOL094C
YBL099W YOL115W
YBL099W YOL133W
YBL099W YOR181W
YBL099W YOR386W
YBL099W YPL078C
YBL099W YPL140C
YBL099W YPL149W
YBL099W YPL150W
YBL099W YPL153C
YBL099W YPL164C
YBL099W YPL204W
YBL099W YPL271W
YBL099W YPR020W
YBL099W YPR110C
YBL099W YPR111W
YBL100C YJL070C
YBL100W-A YGR161W-B
YBL100W-A YJL162C
YBL100W-A YNL229C
YBL100W-A YNL307C

YBL101C	YER125W
YBL101C	YER177W
YBL101C	YJL203W
YBL101C	YOL122C
YBL102W	YDR414C
YBL102W	YFL058W
YBL102W	YGR068C
YBL102W	YLR083C
YBL102W	YLR338W
YBL102W	YLR453C
YBL102W	YOR036W
YBL103C	YDL126C
YBL103C	YDR212W
YBL103C	YDR448W
YBL103C	YFR021W
YBL103C	YHR099W
YBL103C	YHR186C
YBL103C	YIR023W
YBL103C	YJL176C
YBL103C	YJR066W
YBL103C	YLR423C
YBL103C	YML091C
YBL103C	YMR033W
YBL103C	YNL006W
YBL103C	YNL076W
YBL103C	YNL098C
YBL103C	YNR023W
YBL103C	YOL067C
YBL103C	YOL108C
YBL103C	YPL204W
YBL104C	YDL132W
YBL104C	YDR316W
YBL104C	YGL190C
YBL104C	YLR096W
YBL104C	YNL054W
YBL104C	YNL307C
YBL104C	YOR080W
YBL104C	YPL204W
YBL104C	YPL259C
YBL105C	YBR045C
YBL105C	YBR059C
YBL105C	YBR160W
YBL105C	YCL027W
YBL105C	YCR030C
YBL105C	YEL002C

YBL105C	YER118C
YBL105C	YER165W
YBL105C	YFR024C-A
YBL105C	YGL022W
YBL105C	YGR221C
YBL105C	YGR254W
YBL105C	YJL002C
YBL105C	YJL020C
YBL105C	YML109W
YBL105C	YMR149W
YBL105C	YOL083W
YBL105C	YOR231W
YBL105C	YPR154W
YBL105C	YPR165W
YBL106C	YCL017C
YBL106C	YGR009C
YBL106C	YGR172C
YBL106C	YHR098C
YBL106C	YHR161C
YBL106C	YIL033C
YBL106C	YKR030W
YBL106C	YLR206W
YBL106C	YLR423C
YBL106C	YNL094W
YBL106C	YOR197W
YBL106C	YPL174C
YBL107C	YML116W
YBL108W	YHR135C
YBL112C	YML064C
YBL113C	YLR348C
YBR001C	YDR099W
YBR001C	YHR102W
YBR001C	YJL020C
YBR003W	YER022W
YBR003W	YJR022W
YBR003W	YLR310C
YBR004C	YLR082C
YBR006W	YDR382W
YBR006W	YKL023W
YBR007C	YOL133W
YBR008C	YMR047C
YBR010W	YCR084C
YBR010W	YDL159W
YBR010W	YDL200C
YBR010W	YDR224C

YBR010W	YDR225W
YBR010W	YDR227W
YBR010W	YDR440W
YBR010W	YDR477W
YBR010W	YER142C
YBR010W	YGL194C
YBR010W	YIL035C
YBR010W	YJL081C
YBR010W	YJL115W
YBR010W	YJL168C
YBR010W	YKL103C
YBR010W	YKR029C
YBR010W	YLR442C
YBR010W	YNL030W
YBR010W	YPL209C
YBR011C	YBR055C
YBR011C	YBR109C
YBR011C	YBR217W
YBR011C	YDL203C
YBR011C	YDR129C
YBR011C	YGL100W
YBR011C	YGL120C
YBR011C	YGL237C
YBR011C	YHR030C
YBR011C	YJR062C
YBR011C	YKL108W
YBR011C	YLR371W
YBR011C	YLR425W
YBR011C	YMR267W
YBR011C	YNL135C
YBR011C	YNL180C
YBR011C	YNL244C
YBR011C	YPL066W
YBR011C	YPL111W
YBR011C	YPL153C
YBR011C	YPL204W
YBR012C	YPL221W
YBR012W-B	YLR116W
YBR012W-B	YLR373C
YBR012W-B	YMR032W
YBR013C	YGR292W
YBR014C	YBR101C
YBR014C	YBR288C
YBR014C	YDL100C
YBR014C	YJR091C

YBR014C	YPR086W
YBR015C	YGR260W
YBR017C	YBR019C
YBR017C	YBR072W
YBR017C	YBR120C
YBR017C	YBR127C
YBR017C	YBR196C
YBR017C	YBR205W
YBR017C	YBR247C
YBR017C	YCL018W
YBR017C	YDL063C
YBR017C	YDL204W
YBR017C	YDL229W
YBR017C	YDR002W
YBR017C	YDR062W
YBR017C	YDR071C
YBR017C	YDR127W
YBR017C	YDR190C
YBR017C	YDR192C
YBR017C	YDR216W
YBR017C	YDR394W
YBR017C	YEL050C
YBR017C	YEL051W
YBR017C	YEL060C
YBR017C	YER110C
YBR017C	YER165W
YBR017C	YER177W
YBR017C	YFL037W
YBR017C	YFR030W
YBR017C	YFR053C
YBR017C	YGL008C
YBR017C	YGL105W
YBR017C	YGL122C
YBR017C	YGL195W
YBR017C	YGL256W
YBR017C	YGR005C
YBR017C	YGR094W
YBR017C	YGR119C
YBR017C	YGR254W
YBR017C	YGR282C
YBR017C	YHL007C
YBR017C	YHL030W
YBR017C	YHL034C
YBR017C	YHR020W
YBR017C	YHR089C

YBR017C	YIL061C
YBR017C	YIL094C
YBR017C	YIL124W
YBR017C	YJL052W
YBR017C	YJR009C
YBR017C	YJR045C
YBR017C	YJR077C
YBR017C	YKL009W
YBR017C	YKL060C
YBR017C	YKL068W
YBR017C	YKL104C
YBR017C	YKL120W
YBR017C	YKL212W
YBR017C	YKR046C
YBR017C	YLL018C
YBR017C	YLR044C
YBR017C	YLR259C
YBR017C	YLR293C
YBR017C	YLR310C
YBR017C	YLR355C
YBR017C	YML085C
YBR017C	YML124C
YBR017C	YMR012W
YBR017C	YMR047C
YBR017C	YMR105C
YBR017C	YMR108W
YBR017C	YMR145C
YBR017C	YMR241W
YBR017C	YNL035C
YBR017C	YNL037C
YBR017C	YNL124W
YBR017C	YNL169C
YBR017C	YNR069C
YBR017C	YOL123W
YBR017C	YOR098C
YBR017C	YOR141C
YBR017C	YOR176W
YBR017C	YOR185C
YBR017C	YOR259C
YBR017C	YOR317W
YBR017C	YOR374W
YBR017C	YOR375C
YBR017C	YPL049C
YBR017C	YPL240C
YBR017C	YPL266W

YBR018C YDL047W
YBR018C YDL134C
YBR018C YDR128W
YBR018C YDR142C
YBR018C YDR165W
YBR018C YDR398W
YBR018C YER081W
YBR018C YER123W
YBR018C YFR021W
YBR018C YGL137W
YBR018C YGL180W
YBR018C YHR030C
YBR018C YKL108W
YBR018C YLR229C
YBR018C YML115C
YBR018C YMR106C
YBR018C YNL161W
YBR018C YOL062C
YBR018C YOL139C
YBR018C YPL031C
YBR018C YPL259C
YBR018C YPR054W
YBR019C YBR055C
YBR019C YBR155W
YBR019C YBR234C
YBR019C YCL048W
YBR019C YCR009C
YBR019C YDL047W
YBR019C YDL059C
YBR019C YDL225W
YBR019C YDR128W
YBR019C YDR142C
YBR019C YDR143C
YBR019C YDR225W
YBR019C YDR386W
YBR019C YDR398W
YBR019C YEL056W
YBR019C YER017C
YBR019C YER133W
YBR019C YGL100W
YBR019C YGR083C
YBR019C YHL019C
YBR019C YJL092W
YBR019C YJL173C
YBR019C YJR035W

YBR019C	YKR026C
YBR019C	YLR186W
YBR019C	YLR229C
YBR019C	YLR291C
YBR019C	YLR320W
YBR019C	YML064C
YBR019C	YML102W
YBR019C	YMR049C
YBR019C	YMR117C
YBR019C	YNL250W
YBR019C	YNL290W
YBR019C	YOL062C
YBR019C	YOL133W
YBR019C	YOL139C
YBR019C	YOL150C
YBR019C	YOR212W
YBR019C	YOR351C
YBR019C	YPL149W
YBR019C	YPL259C
YBR019C	YPR110C
YBR020W	YBR055C
YBR020W	YBR082C
YBR020W	YBR198C
YBR020W	YBR217W
YBR020W	YBR223C
YBR020W	YCL018W
YBR020W	YCR002C
YBR020W	YCR009C
YBR020W	YCR066W
YBR020W	YCR088W
YBR020W	YDL017W
YBR020W	YDL043C
YBR020W	YDL047W
YBR020W	YDL059C
YBR020W	YDL156W
YBR020W	YDL164C
YBR020W	YDL200C
YBR020W	YDL225W
YBR020W	YDR030C
YBR020W	YDR075W
YBR020W	YDR076W
YBR020W	YDR128W
YBR020W	YDR131C
YBR020W	YDR142C
YBR020W	YDR200C

YBR020W YDR260C
YBR020W YDR267C
YBR020W YDR306C
YBR020W YDR388W
YBR020W YDR394W
YBR020W YDR398W
YBR020W YDR477W
YBR020W YDR523C
YBR020W YEL056W
YBR020W YER081W
YBR020W YER171W
YBR020W YER173W
YBR020W YER179W
YBR020W YFL038C
YBR020W YFR028C
YBR020W YFR031C
YBR020W YGL004C
YBR020W YGL081W
YBR020W YGL137W
YBR020W YGL163C
YBR020W YGL190C
YBR020W YGL237C
YBR020W YGR040W
YBR020W YHR030C
YBR020W YHR199C
YBR020W YJL106W
YBR020W YJL173C
YBR020W YJR090C
YBR020W YKL130C
YBR020W YKL193C
YBR020W YKL210W
YBR020W YKR017C
YBR020W YKR026C
YBR020W YKR036C
YBR020W YLL019C
YBR020W YLR016C
YBR020W YLR044C
YBR020W YLR222C
YBR020W YLR288C
YBR020W YLR291C
YBR020W YLR427W
YBR020W YML032C
YBR020W YML051W
YBR020W YML057W
YBR020W YML064C

YBR020W	YML115C
YBR020W	YMR059W
YBR020W	YMR094W
YBR020W	YMR106C
YBR020W	YMR117C
YBR020W	YMR284W
YBR020W	YNL032W
YBR020W	YNL061W
YBR020W	YNL090W
YBR020W	YNL161W
YBR020W	YNL250W
YBR020W	YNL323W
YBR020W	YNR031C
YBR020W	YOL100W
YBR020W	YOL115W
YBR020W	YOL133W
YBR020W	YOL139C
YBR020W	YOR181W
YBR020W	YOR212W
YBR020W	YOR276W
YBR020W	YPL149W
YBR020W	YPL150W
YBR020W	YPL164C
YBR020W	YPL240C
YBR020W	YPR093C
YBR020W	YPR110C
YBR021W	YHR042W
YBR021W	YOR128C
YBR023C	YHR123W
YBR023C	YJR117W
YBR023C	YLR242C
YBR024W	YBR037C
YBR024W	YHL042W
YBR024W	YPR119W
YBR025C	YBR055C
YBR025C	YBR198C
YBR025C	YDR398W
YBR025C	YKL081W
YBR025C	YMR059W
YBR025C	YNL088W
YBR025C	YNL207W
YBR025C	YOL090W
YBR027C	YKL130C
YBR027C	YLR353W
YBR027C	YMR047C

YBR027C	YOR046C
YBR028C	YBR084W
YBR028C	YCR016W
YBR028C	YER015W
YBR028C	YHR135C
YBR028C	YLR447C
YBR028C	YOL041C
YBR029C	YCR061W
YBR033W	YNL228W
YBR034C	YDR249C
YBR034C	YER017C
YBR034C	YGR165W
YBR034C	YGR181W
YBR034C	YIL061C
YBR034C	YJR022W
YBR034C	YMR048W
YBR034C	YNL078W
YBR034C	YPR132W
YBR035C	YDL101C
YBR035C	YNL189W
YBR036C	YBR106W
YBR036C	YBR159W
YBR036C	YBR161W
YBR036C	YCL052C
YBR036C	YCR034W
YBR036C	YDL015C
YBR036C	YDL212W
YBR036C	YDR062W
YBR036C	YDR276C
YBR036C	YDR307W
YBR036C	YEL027W
YBR036C	YEL063C
YBR036C	YER026C
YBR036C	YGL051W
YBR036C	YGR284C
YBR036C	YHL003C
YBR036C	YHR026W
YBR036C	YHR110W
YBR036C	YHR140W
YBR036C	YIL016W
YBR036C	YIL114C
YBR036C	YJL196C
YBR036C	YJL214W
YBR036C	YJR117W
YBR036C	YKL065C

YBR036C	YKL154W
YBR036C	YLL048C
YBR036C	YLR018C
YBR036C	YLR372W
YBR036C	YML048W
YBR036C	YMR215W
YBR036C	YMR306W
YBR036C	YNL044W
YBR036C	YOR016C
YBR036C	YPL076W
YBR036C	YPL227C
YBR036C	YPL264C
YBR036C	YPR198W
YBR037C	YPR079W
YBR038W	YBR160W
YBR038W	YHR123W
YBR038W	YNL307C
YBR038W	YPL020C
YBR038W	YPR106W
YBR039W	YBR093C
YBR039W	YDL029W
YBR039W	YDL047W
YBR039W	YDL059C
YBR039W	YDL145C
YBR039W	YDR523C
YBR039W	YER171W
YBR039W	YFR028C
YBR039W	YGL237C
YBR039W	YGR104C
YBR039W	YHR197W
YBR039W	YIL106W
YBR039W	YJR068W
YBR039W	YJR121W
YBR039W	YKR036C
YBR039W	YML058W
YBR039W	YMR059W
YBR039W	YMR106C
YBR039W	YOL087C
YBR039W	YPL256C
YBR039W	YPR106W
YBR040W	YCL040W
YBR040W	YHL002W
YBR041W	YDL153C
YBR042C	YER081W
YBR042C	YHR115C

YBR043C	YGR074W
YBR043C	YGR157W
YBR043C	YJL155C
YBR044C	YBR221C
YBR044C	YDL093W
YBR044C	YHR042W
YBR044C	YJR022W
YBR044C	YKL004W
YBR044C	YKL141W
YBR044C	YKL148C
YBR044C	YLL041C
YBR045C	YDR103W
YBR045C	YER054C
YBR045C	YER133W
YBR045C	YMR001C
YBR045C	YOR178C
YBR047W	YER022W
YBR047W	YJL128C
YBR049C	YDR130C
YBR049C	YDR303C
YBR049C	YGL234W
YBR049C	YGR274C
YBR049C	YKR001C
YBR049C	YLR176C
YBR049C	YLR357W
YBR049C	YMR091C
YBR049C	YNL189W
YBR049C	YOL006C
YBR049C	YPL082C
YBR049C	YPR110C
YBR050C	YBR109C
YBR050C	YDR099W
YBR050C	YER133W
YBR050C	YNR052C
YBR052C	YDR032C
YBR053C	YLR310C
YBR054W	YBR159W
YBR054W	YBR302C
YBR054W	YCL038C
YBR054W	YDR190C
YBR054W	YGL022W
YBR054W	YHR072W
YBR054W	YJR010C-A
YBR054W	YJR091C
YBR054W	YKR039W

YBR054W	YLR372W
YBR054W	YNL048W
YBR054W	YNR017W
YBR054W	YOR016C
YBR054W	YOR040W
YBR054W	YOR254C
YBR055C	YBR121C
YBR055C	YBR126C
YBR055C	YBR127C
YBR055C	YBR133C
YBR055C	YBR149W
YBR055C	YBR152W
YBR055C	YBR169C
YBR055C	YBR196C
YBR055C	YCL018W
YBR055C	YCL040W
YBR055C	YCL043C
YBR055C	YCR002C
YBR055C	YCR053W
YBR055C	YDL064W
YBR055C	YDL078C
YBR055C	YDL124W
YBR055C	YDL126C
YBR055C	YDL229W
YBR055C	YDR037W
YBR055C	YDR099W
YBR055C	YDR129C
YBR055C	YDR158W
YBR055C	YDR283C
YBR055C	YDR353W
YBR055C	YDR473C
YBR055C	YEL034W
YBR055C	YER003C
YBR055C	YER043C
YBR055C	YER091C
YBR055C	YER146W
YBR055C	YER165W
YBR055C	YER172C
YBR055C	YFL017W-A
YBR055C	YFR053C
YBR055C	YGL202W
YBR055C	YGL206C
YBR055C	YGL245W
YBR055C	YGL253W
YBR055C	YGR061C

YBR055C YGR074W
YBR055C YGR091W
YBR055C YGR094W
YBR055C YGR155W
YBR055C YGR204W
YBR055C YGR240C
YBR055C YGR254W
YBR055C YHR019C
YBR055C YHR064C
YBR055C YHR165C
YBR055C YHR183W
YBR055C YHR208W
YBR055C YJL034W
YBR055C YJL052W
YBR055C YJL080C
YBR055C YJL138C
YBR055C YJL167W
YBR055C YJR009C
YBR055C YJR022W
YBR055C YJR104C
YBR055C YJR105W
YBR055C YJR139C
YBR055C YKL035W
YBR055C YKL060C
YBR055C YKL081W
YBR055C YKL085W
YBR055C YKL157W
YBR055C YKL210W
YBR055C YLL018C
YBR055C YLL026W
YBR055C YLR044C
YBR055C YLR147C
YBR055C YLR153C
YBR055C YLR259C
YBR055C YLR304C
YBR055C YLR355C
YBR055C YLR432W
YBR055C YLR446W
YBR055C YMR012W
YBR055C YMR105C
YBR055C YMR120C
YBR055C YMR186W
YBR055C YMR205C
YBR055C YNL147W
YBR055C YNR001C

YBR055C	YOR120W
YBR055C	YOR133W
YBR055C	YOR136W
YBR055C	YOR159C
YBR055C	YOR335C
YBR055C	YOR374W
YBR055C	YOR375C
YBR055C	YPL004C
YBR055C	YPL028W
YBR055C	YPL061W
YBR055C	YPL240C
YBR055C	YPR082C
YBR055C	YPR160W
YBR055C	YPR178W
YBR055C	YPR182W
YBR056W	YFR007W
YBR057C	YCL055W
YBR057C	YDR034C
YBR057C	YDR206W
YBR057C	YGL192W
YBR057C	YIL144W
YBR057C	YKL098W
YBR057C	YKR031C
YBR057C	YLR222C
YBR058C	YGR268C
YBR058C	YMR309C
YBR058C	YOL059W
YBR058C	YOL064C
YBR058C	YOL086C
YBR058C	YOL113W
YBR058C	YOL132W
YBR058C	YOL156W
YBR058C	YOR123C
YBR058C-A	YGR038W
YBR058C-A	YLR350W
YBR058C-A	YMR296C
YBR059C	YBR118W
YBR059C	YCL032W
YBR059C	YER144C
YBR059C	YER177W
YBR059C	YFR014C
YBR059C	YGL213C
YBR059C	YGR199W
YBR059C	YIL095W
YBR059C	YIR006C

YBR059C	YJL039C
YBR059C	YJL098W
YBR059C	YJR045C
YBR059C	YML014W
YBR059C	YMR144W
YBR059C	YNL020C
YBR059C	YNL243W
YBR059C	YOR061W
YBR059C	YPR033C
YBR060C	YBR160W
YBR060C	YHR118C
YBR060C	YJL194W
YBR060C	YLL004W
YBR060C	YLR117C
YBR060C	YML065W
YBR060C	YNL261W
YBR060C	YPR162C
YBR061C	YBR300C
YBR061C	YDR383C
YBR061C	YDR461W
YBR061C	YER028C
YBR061C	YGR182C
YBR061C	YGR195W
YBR061C	YGR201C
YBR061C	YGR219W
YBR061C	YGR235C
YBR061C	YGR277C
YBR061C	YHL011C
YBR061C	YHR008C
YBR061C	YHR022C
YBR061C	YHR026W
YBR061C	YHR058C
YBR061C	YHR059W
YBR061C	YJL016W
YBR061C	YJL097W
YBR061C	YJL140W
YBR061C	YJL166W
YBR063C	YDR369C
YBR064W	YJL159W
YBR064W	YOR317W
YBR065C	YER081W
YBR065C	YLL036C
YBR065C	YLR117C
YBR065C	YMR213W
YBR065C	YPL151C

YBR065C	YPR182W
YBR066C	YDR477W
YBR066C	YJR091C
YBR067C	YJR091C
YBR067C	YNL237W
YBR067C	YPR138C
YBR068C	YDL212W
YBR068C	YDR046C
YBR068C	YFR024C-A
YBR068C	YJR001W
YBR068C	YOR133W
YBR069C	YCR034W
YBR069C	YDL015C
YBR069C	YDL043C
YBR069C	YDL199C
YBR069C	YDR171W
YBR069C	YER110C
YBR069C	YFR024C-A
YBR069C	YGL051W
YBR069C	YGL200C
YBR069C	YGR218W
YBR069C	YGR284C
YBR069C	YHR020W
YBR069C	YHR110W
YBR069C	YHR114W
YBR069C	YJL117W
YBR069C	YJR010C-A
YBR069C	YJR077C
YBR069C	YKL065C
YBR069C	YLR044C
YBR069C	YLR343W
YBR069C	YLR372W
YBR069C	YML048W
YBR069C	YNL064C
YBR069C	YOL109W
YBR069C	YOR016C
YBR069C	YPL233W
YBR069C	YPR198W
YBR070C	YJL019W
YBR072W	YBR094W
YBR072W	YBR103W
YBR072W	YBR109C
YBR072W	YBR114W
YBR072W	YBR160W
YBR072W	YBR198C

YBR072W YBR217W
YBR072W YCR002C
YBR072W YCR005C
YBR072W YCR009C
YBR072W YCR077C
YBR072W YCR084C
YBR072W YDL029W
YBR072W YDL047W
YBR072W YDL059C
YBR072W YDL101C
YBR072W YDL145C
YBR072W YDL179W
YBR072W YDL193W
YBR072W YDL200C
YBR072W YDL225W
YBR072W YDL239C
YBR072W YDR128W
YBR072W YDR142C
YBR072W YDR200C
YBR072W YDR227W
YBR072W YDR267C
YBR072W YDR339C
YBR072W YDR398W
YBR072W YDR523C
YBR072W YEL056W
YBR072W YER012W
YBR072W YER059W
YBR072W YER066C-A
YBR072W YER133W
YBR072W YER171W
YBR072W YFL034C-B
YBR072W YFR028C
YBR072W YFR040W
YBR072W YGL004C
YBR072W YGL137W
YBR072W YGL208W
YBR072W YGR083C
YBR072W YGR092W
YBR072W YGR262C
YBR072W YHR030C
YBR072W YHR107C
YBR072W YHR120W
YBR072W YHR135C
YBR072W YHR186C
YBR072W YHR199C

YBR072W YIL007C
YBR072W YIL035C
YBR072W YIL046W
YBR072W YIL066C
YBR072W YIL128W
YBR072W YIL142W
YBR072W YIL147C
YBR072W YJL005W
YBR072W YJL098W
YBR072W YJL157C
YBR072W YJL173C
YBR072W YJR035W
YBR072W YJR053W
YBR072W YJR062C
YBR072W YJR076C
YBR072W YKL103C
YBR072W YKL139W
YBR072W YKL193C
YBR072W YKR026C
YBR072W YKR036C
YBR072W YLL011W
YBR072W YLR097C
YBR072W YLR148W
YBR072W YLR175W
YBR072W YLR196W
YBR072W YLR208W
YBR072W YLR320W
YBR072W YLR442C
YBR072W YML064C
YBR072W YML112W
YBR072W YML115C
YBR072W YMR049C
YBR072W YMR055C
YBR072W YMR106C
YBR072W YMR117C
YBR072W YMR284W
YBR072W YNL128W
YBR072W YNL189W
YBR072W YNL290W
YBR072W YNL317W
YBR072W YNR019W
YBR072W YNR031C
YBR072W YOL062C
YBR072W YOL133W
YBR072W YOR125C

YBR072W	YOR212W
YBR072W	YOR229W
YBR072W	YOR231W
YBR072W	YOR269W
YBR072W	YOR319W
YBR072W	YPL150W
YBR072W	YPL169C
YBR072W	YPL204W
YBR072W	YPL259C
YBR072W	YPR054W
YBR072W	YPR110C
YBR072W	YPR137W
YBR072W	YPR178W
YBR073W	YBR196C
YBR073W	YER179W
YBR073W	YGL213C
YBR073W	YLR453C
YBR074W	YER081W
YBR074W	YJR091C
YBR074W	YMR083W
YBR076W	YJR091C
YBR077C	YGR201C
YBR077C	YKR007W
YBR077C	YMR004W
YBR077C	YOR045W
YBR078W	YOL133W
YBR079C	YBR143C
YBR079C	YDL005C
YBR079C	YDR091C
YBR079C	YDR429C
YBR079C	YGL130W
YBR079C	YIL071C
YBR079C	YIL106W
YBR079C	YJR007W
YBR079C	YLR192C
YBR079C	YML085C
YBR079C	YMR146C
YBR079C	YMR309C
YBR079C	YNL243W
YBR079C	YNL244C
YBR079C	YOL087C
YBR079C	YOR039W
YBR079C	YOR361C
YBR079C	YPL001W
YBR079C	YPL237W

YBR079C	YPR016C
YBR079C	YPR041W
YBR079C	YPR086W
YBR080C	YBR217W
YBR080C	YGL137W
YBR080C	YGR262C
YBR080C	YOR036W
YBR081C	YBR198C
YBR081C	YBR253W
YBR081C	YCL010C
YBR081C	YDR146C
YBR081C	YDR167W
YBR081C	YDR176W
YBR081C	YDR216W
YBR081C	YDR448W
YBR081C	YGL066W
YBR081C	YGL112C
YBR081C	YGL244W
YBR081C	YGR252W
YBR081C	YHR099W
YBR081C	YLR055C
YBR081C	YML007W
YBR081C	YMR223W
YBR081C	YMR236W
YBR081C	YOL148C
YBR081C	YOR119C
YBR081C	YPL254W
YBR081C	YPR025C
YBR082C	YDR529C
YBR082C	YGL206C
YBR082C	YJL047C
YBR082C	YKL010C
YBR082C	YNL071W
YBR082C	YOL055C
YBR083W	YDR224C
YBR083W	YGR040W
YBR083W	YHR084W
YBR083W	YKL161C
YBR083W	YNL030W
YBR083W	YPL235W
YBR084W	YBR143C
YBR084W	YCR008W
YBR084W	YDR466W
YBR084W	YER123W
YBR084W	YFR040W

YBR084W	YGL019W
YBR084W	YGL099W
YBR084W	YGR285C
YBR084W	YHR013C
YBR084W	YHR089C
YBR084W	YIL018W
YBR084W	YJR059W
YBR084W	YKL135C
YBR084W	YNL030W
YBR084W	YOR014W
YBR085W	YBR217W
YBR085W	YCR061W
YBR085W	YDL059C
YBR085W	YER161C
YBR085W	YER171W
YBR085W	YGL237C
YBR085W	YGR262C
YBR085W	YHR002W
YBR085W	YHR135C
YBR085W	YJL173C
YBR085W	YML058W
YBR085W	YOR181W
YBR086C	YKL212W
YBR086C	YNL194C
YBR086C	YPR086W
YBR087W	YBR088C
YBR087W	YER173W
YBR087W	YHR191C
YBR087W	YJR068W
YBR087W	YLR390W
YBR087W	YMR078C
YBR087W	YMR181C
YBR087W	YNL088W
YBR087W	YNL290W
YBR087W	YOL094C
YBR087W	YOR144C
YBR087W	YOR217W
YBR088C	YBR196C
YBR088C	YCL040W
YBR088C	YCR092C
YBR088C	YDL164C
YBR088C	YDR097C
YBR088C	YER041W
YBR088C	YFR053C
YBR088C	YGR155W

YBR088C	YHR031C
YBR088C	YJL088W
YBR088C	YJR043C
YBR088C	YJR068W
YBR088C	YKL113C
YBR088C	YLR035C
YBR088C	YML021C
YBR088C	YML056C
YBR088C	YMR167W
YBR088C	YNL071W
YBR088C	YNL290W
YBR088C	YOL055C
YBR088C	YOL094C
YBR088C	YOR116C
YBR088C	YOR217W
YBR088C	YPL140C
YBR089C-A	YBR245C
YBR089C-A	YFR013W
YBR089C-A	YFR037C
YBR089C-A	YGL133W
YBR089C-A	YIL126W
YBR089C-A	YJL026W
YBR089C-A	YKR001C
YBR089C-A	YLR033W
YBR089C-A	YLR321C
YBR089C-A	YMR125W
YBR089C-A	YNR003C
YBR089C-A	YOR110W
YBR089C-A	YOR116C
YBR089C-A	YOR304W
YBR089C-A	YPL082C
YBR089C-A	YPL235W
YBR089W	YGL070C
YBR090C	YER158C
YBR091C	YDL217C
YBR091C	YHR005C-A
YBR091C	YJL054W
YBR091C	YKL002W
YBR091C	YLR288C
YBR091C	YOR297C
YBR093C	YCL043C
YBR093C	YGR282C
YBR093C	YJL066C
YBR093C	YJL176C
YBR093C	YKR106W

YBR093C	YML077W
YBR094W	YDL015C
YBR094W	YLR264W
YBR094W	YOL149W
YBR094W	YOR061W
YBR094W	YOR167C
YBR094W	YPR132W
YBR095C	YCR009C
YBR095C	YDR143C
YBR095C	YIL094C
YBR095C	YMR159C
YBR095C	YNL189W
YBR095C	YNL330C
YBR095C	YOL004W
YBR095C	YPL149W
YBR096W	YIL034C
YBR097W	YLR240W
YBR098W	YBR160W
YBR098W	YDR386W
YBR098W	YFR024C-A
YBR098W	YHR016C
YBR098W	YJL088W
YBR098W	YJR045C
YBR098W	YJR121W
YBR099C	YER133W
YBR099C	YJR063W
YBR101C	YDR171W
YBR101C	YHL021C
YBR101C	YJL117W
YBR101C	YJL138C
YBR101C	YKL171W
YBR101C	YLL062C
YBR101C	YLR074C
YBR101C	YLR194C
YBR101C	YMR092C
YBR101C	YMR173W
YBR101C	YOL011W
YBR101C	YPL136W
YBR101C	YPR154W
YBR102C	YBR160W
YBR102C	YDR166C
YBR102C	YER008C
YBR102C	YGL233W
YBR102C	YHR165C
YBR102C	YIL061C

YBR102C YIL068C
YBR102C YLR166C
YBR102C YLR423C
YBR102C YML097C
YBR102C YNR046W
YBR102C YPR055W
YBR103W YCL018W
YBR103W YCR033W
YBR103W YCR077C
YBR103W YDL112W
YBR103W YDL185W
YBR103W YDR148C
YBR103W YDR155C
YBR103W YDR227W
YBR103W YEL064C
YBR103W YGL194C
YBR103W YGR239C
YBR103W YGR254W
YBR103W YGR296W
YBR103W YHL004W
YBR103W YIL112W
YBR103W YJR121W
YBR103W YJR141W
YBR103W YKR029C
YBR103W YLR347C
YBR103W YLR409C
YBR103W YML109W
YBR103W YMR061W
YBR103W YMR155W
YBR103W YMR273C
YBR103W YNL064C
YBR103W YNL189W
YBR103W YOL068C
YBR103W YOR319W
YBR105C YCL039W
YBR105C YMR047C
YBR105C YMR061W
YBR106W YBR110W
YBR106W YBR183W
YBR106W YBR243C
YBR106W YBR283C
YBR106W YBR287W
YBR106W YBR290W
YBR106W YBR293W
YBR106W YBR298C

YBR106W YCL025C
YBR106W YCL038C
YBR106W YCR028C
YBR106W YCR037C
YBR106W YCR061W
YBR106W YCR098C
YBR106W YDL015C
YBR106W YDL054C
YBR106W YDL093W
YBR106W YDL128W
YBR106W YDR046C
YBR106W YDR297W
YBR106W YDR307W
YBR106W YDR331W
YBR106W YDR342C
YBR106W YDR414C
YBR106W YDR456W
YBR106W YDR508C
YBR106W YEL002C
YBR106W YEL017C-A
YBR106W YEL027W
YBR106W YEL063C
YBR106W YER056C
YBR106W YER060W-A
YBR106W YFL025C
YBR106W YGL051W
YBR106W YGL054C
YBR106W YGL055W
YBR106W YGL070C
YBR106W YGL104C
YBR106W YGL167C
YBR106W YGL200C
YBR106W YGR060W
YBR106W YGR224W
YBR106W YGR260W
YBR106W YGR289C
YBR106W YHL042W
YBR106W YHL048W
YBR106W YHR026W
YBR106W YHR094C
YBR106W YHR123W
YBR106W YHR133C
YBR106W YHR140W
YBR106W YHR142W
YBR106W YIL048W

YBR106W YIL120W
YBR106W YJL012C
YBR106W YJL059W
YBR106W YJL108C
YBR106W YJL117W
YBR106W YJL196C
YBR106W YJR010C-A
YBR106W YJR015W
YBR106W YJR124C
YBR106W YKL002W
YBR106W YKL146W
YBR106W YKL165C
YBR106W YKR039W
YBR106W YKR106W
YBR106W YLL006W
YBR106W YLL023C
YBR106W YLL028W
YBR106W YLL061W
YBR106W YLR034C
YBR106W YLR378C
YBR106W YML067C
YBR106W YML075C
YBR106W YML123C
YBR106W YMR058W
YBR106W YMR149W
YBR106W YMR215W
YBR106W YMR274C
YBR106W YMR279C
YBR106W YNL048W
YBR106W YNL101W
YBR106W YNL125C
YBR106W YNL130C
YBR106W YNL275W
YBR106W YNL321W
YBR106W YNR013C
YBR106W YNR019W
YBR106W YNR055C
YBR106W YOL020W
YBR106W YOL030W
YBR106W YOL132W
YBR106W YOL156W
YBR106W YPL076W
YBR106W YPL189W
YBR106W YPL264C
YBR106W YPL274W

YBR106W	YPR124W
YBR106W	YPR156C
YBR106W	YPR194C
YBR106W	YPR198W
YBR106W	YPR201W
YBR107C	YBR211C
YBR107C	YDR254W
YBR107C	YDR318W
YBR107C	YDR383C
YBR107C	YER081W
YBR107C	YGL070C
YBR107C	YJR135C
YBR107C	YLR381W
YBR107C	YPL018W
YBR108W	YCR009C
YBR108W	YCR088W
YBR108W	YDL117W
YBR108W	YDR388W
YBR108W	YFR024C-A
YBR108W	YGR136W
YBR108W	YHL002W
YBR108W	YHR016C
YBR108W	YHR114W
YBR108W	YJL020C
YBR108W	YLR191W
YBR108W	YLR438C-A
YBR108W	YMR032W
YBR108W	YPR154W
YBR109C	YBR118W
YBR109C	YBR127C
YBR109C	YBR130C
YBR109C	YBR196C
YBR109C	YBR213W
YBR109C	YBR229C
YBR109C	YDL028C
YBR109C	YDR032C
YBR109C	YDR099W
YBR109C	YDR155C
YBR109C	YDR195W
YBR109C	YDR231C
YBR109C	YDR292C
YBR109C	YDR356W
YBR109C	YEL034W
YBR109C	YER023W
YBR109C	YER177W

YBR109C YFL003C
YBR109C YFL010W-A
YBR109C YFL039C
YBR109C YFR004W
YBR109C YFR014C
YBR109C YFR053C
YBR109C YFR054C
YBR109C YGL043W
YBR109C YGL063W
YBR109C YGL106W
YBR109C YGL141W
YBR109C YGL206C
YBR109C YGL229C
YBR109C YGR034W
YBR109C YGR050C
YBR109C YGR094W
YBR109C YGR144W
YBR109C YGR253C
YBR109C YHR028C
YBR109C YHR179W
YBR109C YIL021W
YBR109C YIL095W
YBR109C YJL187C
YBR109C YJR073C
YBR109C YJR104C
YBR109C YJR121W
YBR109C YKL067W
YBR109C YKL129C
YBR109C YKL152C
YBR109C YKL175W
YBR109C YKL182W
YBR109C YKL210W
YBR109C YKR025W
YBR109C YLL040C
YBR109C YLL050C
YBR109C YLL051C
YBR109C YLR259C
YBR109C YLR433C
YBR109C YLR443W
YBR109C YML057W
YBR109C YML094C-A
YBR109C YMR105C
YBR109C YMR109W
YBR109C YMR111C
YBR109C YMR200W

YBR109C	YMR250W
YBR109C	YNL053W
YBR109C	YNL066W
YBR109C	YNL079C
YBR109C	YNL202W
YBR109C	YNL281W
YBR109C	YNR035C
YBR109C	YOL016C
YBR109C	YOR029W
YBR109C	YOR035C
YBR109C	YOR326W
YBR109C	YOR358W
YBR109C	YOR374W
YBR109C	YPL242C
YBR109W-A	YHR114W
YBR110W	YBR159W
YBR110W	YBR290W
YBR110W	YCL027W
YBR110W	YCR034W
YBR110W	YDL015C
YBR110W	YDL206W
YBR110W	YDR307W
YBR110W	YDR331W
YBR110W	YEL002C
YBR110W	YEL017C-A
YBR110W	YEL063C
YBR110W	YGL200C
YBR110W	YGR060W
YBR110W	YGR105W
YBR110W	YHL048W
YBR110W	YHR026W
YBR110W	YHR133C
YBR110W	YHR140W
YBR110W	YHR142W
YBR110W	YJR010C-A
YBR110W	YKL065C
YBR110W	YLL028W
YBR110W	YLL061W
YBR110W	YLR241W
YBR110W	YLR372W
YBR110W	YML123C
YBR110W	YMR058W
YBR110W	YMR215W
YBR110W	YPL076W
YBR110W	YPL264C

YBR110W	YPL274W
YBR110W	YPR156C
YBR111W-A	YGL066W
YBR111W-A	YHR099W
YBR111W-A	YMR223W
YBR111W-A	YPL047W
YBR112C	YBR200W
YBR112C	YCR084C
YBR112C	YCR096C
YBR112C	YDR043C
YBR112C	YGL035C
YBR112C	YGL162W
YBR112C	YHL002W
YBR112C	YIL061C
YBR112C	YMR240C
YBR112C	YNL167C
YBR112C	YPL181W
YBR113W	YDL043C
YBR114W	YBR200W
YBR114W	YCL040W
YBR114W	YCR028C-A
YBR114W	YDR097C
YBR114W	YDR453C
YBR114W	YFL018C
YBR114W	YFR053C
YBR114W	YGR192C
YBR114W	YGR193C
YBR114W	YHR183W
YBR114W	YIR009W
YBR114W	YJR052W
YBR114W	YJR077C
YBR114W	YKL112W
YBR114W	YKL152C
YBR114W	YKR001C
YBR114W	YLR044C
YBR114W	YLR147C
YBR114W	YLR259C
YBR114W	YMR190C
YBR114W	YMR201C
YBR114W	YMR226C
YBR114W	YMR315W
YBR114W	YNL030W
YBR114W	YNL071W
YBR114W	YNR001C
YBR114W	YOL012C

YBR114W	YOL086C
YBR114W	YOL097C
YBR114W	YOR332W
YBR114W	YPL061W
YBR114W	YPL154C
YBR115C	YGL154C
YBR116C	YHR005C
YBR116C	YJR063W
YBR117C	YIL061C
YBR117C	YLR447C
YBR117C	YPR074C
YBR118W	YBR217W
YBR118W	YBR264C
YBR118W	YBR288C
YBR118W	YCR005C
YBR118W	YCR094W
YBR118W	YDL017W
YBR118W	YDL043C
YBR118W	YDL126C
YBR118W	YDL164C
YBR118W	YDL179W
YBR118W	YDL192W
YBR118W	YDL220C
YBR118W	YDR075W
YBR118W	YDR131C
YBR118W	YDR192C
YBR118W	YDR228C
YBR118W	YDR306C
YBR118W	YDR386W
YBR118W	YDR388W
YBR118W	YDR394W
YBR118W	YDR477W
YBR118W	YDR480W
YBR118W	YDR522C
YBR118W	YDR523C
YBR118W	YEL056W
YBR118W	YER012W
YBR118W	YER020W
YBR118W	YER095W
YBR118W	YER133W
YBR118W	YER161C
YBR118W	YER179W
YBR118W	YFL014W
YBR118W	YFL038C
YBR118W	YFL039C

YBR118W YGL163C
YBR118W YGL190C
YBR118W YGL208W
YBR118W YGR040W
YBR118W YGR104C
YBR118W YGR173W
YBR118W YGR240C
YBR118W YGR280C
YBR118W YHL034C
YBR118W YHR014W
YBR118W YHR111W
YBR118W YHR135C
YBR118W YHR166C
YBR118W YHR183W
YBR118W YHR186C
YBR118W YHR188C
YBR118W YIL046W
YBR118W YIL066C
YBR118W YIL128W
YBR118W YJL106W
YBR118W YJL173C
YBR118W YJL203W
YBR118W YJR062C
YBR118W YJR076C
YBR118W YKL021C
YBR118W YKL081W
YBR118W YKL166C
YBR118W YLR024C
YBR118W YLR074C
YBR118W YLR216C
YBR118W YLR233C
YBR118W YLR249W
YBR118W YLR262C
YBR118W YLR293C
YBR118W YLR306W
YBR118W YLR340W
YBR118W YML064C
YBR118W YML095C
YBR118W YMR022W
YBR118W YMR059W
YBR118W YMR104C
YBR118W YMR106C
YBR118W YMR205C
YBR118W YMR240C
YBR118W YNL061W

YBR118W	YNL113W
YBR118W	YNL116W
YBR118W	YNL135C
YBR118W	YNL175C
YBR118W	YNL236W
YBR118W	YNL271C
YBR118W	YNL311C
YBR118W	YOL128C
YBR118W	YOL133W
YBR118W	YOR212W
YBR118W	YOR319W
YBR118W	YOR339C
YBR118W	YOR351C
YBR118W	YPL048W
YBR118W	YPL111W
YBR118W	YPL236C
YBR118W	YPR017C
YBR118W	YPR110C
YBR118W	YPR111W
YBR119W	YDR110W
YBR119W	YDR235W
YBR119W	YDR240C
YBR119W	YFL017W-A
YBR119W	YGR013W
YBR119W	YHR086W
YBR119W	YIL034C
YBR119W	YIL061C
YBR119W	YKL012W
YBR119W	YLR223C
YBR119W	YLR298C
YBR119W	YLR449W
YBR119W	YML016C
YBR119W	YML046W
YBR119W	YMR125W
YBR119W	YNL227C
YBR119W	YNL277W
YBR119W	YPL178W
YBR120C	YJR082C
YBR120C	YKR026C
YBR121C	YLR427W
YBR121C	YNL244C
YBR122C	YGR220C
YBR122C	YJL020C
YBR122C	YJR091C
YBR122C	YLR447C

YBR122C	YNL284C
YBR123C	YDR362C
YBR123C	YGL133W
YBR123C	YGR047C
YBR123C	YGR246C
YBR123C	YKL068W
YBR123C	YOR110W
YBR123C	YPL007C
YBR124W	YLR288C
YBR125C	YDR071C
YBR125C	YDR186C
YBR125C	YDR247W
YBR125C	YDR507C
YBR125C	YGL206C
YBR125C	YNL189W
YBR125C	YPL204W
YBR126C	YBR265W
YBR126C	YCR079W
YBR126C	YDL017W
YBR126C	YDR028C
YBR126C	YDR074W
YBR126C	YDR247W
YBR126C	YER112W
YBR126C	YFL033C
YBR126C	YFR028C
YBR126C	YGR092W
YBR126C	YIL177C
YBR126C	YIR032C
YBR126C	YJL138C
YBR126C	YJR138W
YBR126C	YLL019C
YBR126C	YLR411W
YBR126C	YML100W
YBR126C	YMR106C
YBR126C	YMR139W
YBR126C	YMR251W
YBR126C	YMR261C
YBR126C	YNL076W
YBR126C	YNL244C
YBR126C	YOL133W
YBR126C	YOR089C
YBR126C	YOR098C
YBR126C	YPR035W
YBR127C	YBR198C
YBR127C	YBR203W

YBR127C	YBR217W
YBR127C	YBR223C
YBR127C	YCR002C
YBR127C	YCR009C
YBR127C	YCR079W
YBR127C	YDL017W
YBR127C	YDL029W
YBR127C	YDL043C
YBR127C	YDL047W
YBR127C	YDL059C
YBR127C	YDL132W
YBR127C	YDL156W
YBR127C	YDL164C
YBR127C	YDL179W
YBR127C	YDL185W
YBR127C	YDL200C
YBR127C	YDR075W
YBR127C	YDR076W
YBR127C	YDR092W
YBR127C	YDR128W
YBR127C	YDR131C
YBR127C	YDR138W
YBR127C	YDR142C
YBR127C	YDR143C
YBR127C	YDR200C
YBR127C	YDR202C
YBR127C	YDR227W
YBR127C	YDR260C
YBR127C	YDR328C
YBR127C	YDR369C
YBR127C	YDR388W
YBR127C	YDR394W
YBR127C	YDR523C
YBR127C	YER017C
YBR127C	YER125W
YBR127C	YER171W
YBR127C	YER173W
YBR127C	YER179W
YBR127C	YFR031C
YBR127C	YGL004C
YBR127C	YGL137W
YBR127C	YGL163C
YBR127C	YGL190C
YBR127C	YGL208W
YBR127C	YGL237C

YBR127C YGR040W
YBR127C YGR067C
YBR127C YGR083C
YBR127C YGR117C
YBR127C YGR188C
YBR127C YHL007C
YBR127C YHR039C-A
YBR127C YHR107C
YBR127C YHR135C
YBR127C YIL046W
YBR127C YIL061C
YBR127C YJL106W
YBR127C YJR007W
YBR127C YJR033C
YBR127C YKL080W
YBR127C YKL193C
YBR127C YKR026C
YBR127C YKR036C
YBR127C YLL011W
YBR127C YLR222C
YBR127C YLR288C
YBR127C YLR314C
YBR127C YLR427W
YBR127C YLR447C
YBR127C YML057W
YBR127C YML064C
YBR127C YML115C
YBR127C YMR054W
YBR127C YMR059W
YBR127C YMR093W
YBR127C YMR094W
YBR127C YMR106C
YBR127C YMR116C
YBR127C YMR117C
YBR127C YMR284W
YBR127C YNL053W
YBR127C YNL061W
YBR127C YNL090W
YBR127C YNL113W
YBR127C YNL135C
YBR127C YNL161W
YBR127C YNL244C
YBR127C YNL250W
YBR127C YNL290W
YBR127C YNR031C

YBR127C	YOL062C
YBR127C	YOL087C
YBR127C	YOL113W
YBR127C	YOL115W
YBR127C	YOL133W
YBR127C	YOR181W
YBR127C	YOR212W
YBR127C	YOR270C
YBR127C	YOR351C
YBR127C	YPL140C
YBR127C	YPL150W
YBR127C	YPL164C
YBR127C	YPL194W
YBR127C	YPL259C
YBR127C	YPR110C
YBR128C	YDL185W
YBR128C	YDR162C
YBR128C	YER081W
YBR128C	YHR005C
YBR128C	YPL120W
YBR129C	YDL059C
YBR129C	YLR288C
YBR130C	YBR143C
YBR130C	YCL040W
YBR130C	YFR053C
YBR130C	YGL106W
YBR130C	YGL206C
YBR130C	YHR107C
YBR130C	YIL144W
YBR130C	YJL071W
YBR130C	YJL088W
YBR130C	YJR045C
YBR130C	YKL130C
YBR130C	YLR092W
YBR130C	YLR310C
YBR130C	YMR047C
YBR130C	YOL133W
YBR131W	YGL124C
YBR131W	YML001W
YBR131W	YPL045W
YBR132C	YDR388W
YBR132C	YFR024C-A
YBR132C	YHR016C
YBR132C	YHR042W
YBR132C	YLR191W

YBR132C	YMR032W
YBR133C	YCR088W
YBR133C	YDL154W
YBR133C	YER087W
YBR133C	YGL187C
YBR133C	YHL007C
YBR133C	YJL187C
YBR133C	YKL101W
YBR133C	YNL094W
YBR134W	YOR128C
YBR135W	YBR160W
YBR135W	YBR252W
YBR135W	YBR295W
YBR135W	YDL155W
YBR135W	YDR078C
YBR135W	YDR170W-A
YBR135W	YEL034W
YBR135W	YER138C
YBR135W	YGR108W
YBR135W	YGR109C
YBR135W	YJL187C
YBR135W	YKR091W
YBR135W	YLR079W
YBR135W	YLR210W
YBR135W	YLR226W
YBR135W	YMR199W
YBR135W	YOL055C
YBR135W	YOR080W
YBR135W	YPL014W
YBR135W	YPL256C
YBR135W	YPR119W
YBR135W	YPR120C
YBR136W	YBR143C
YBR136W	YCR004C
YBR136W	YDR097C
YBR136W	YDR499W
YBR136W	YGL245W
YBR136W	YIR009W
YBR136W	YJL090C
YBR136W	YJL173C
YBR136W	YJR045C
YBR136W	YKL085W
YBR136W	YLR025W
YBR136W	YLR180W
YBR136W	YLR304C

YBR136W	YMR012W
YBR136W	YNL265C
YBR136W	YNL312W
YBR136W	YOR027W
YBR136W	YPL153C
YBR136W	YPR010C
YBR137W	YKR083C
YBR137W	YML064C
YBR137W	YMR047C
YBR137W	YNL189W
YBR137W	YOR007C
YBR137W	YOR127W
YBR138C	YBR160W
YBR138C	YGL181W
YBR138C	YIL046W
YBR139W	YBR280C
YBR139W	YDR328C
YBR139W	YOR381W
YBR140C	YJL005W
YBR140C	YLR310C
YBR140C	YMR139W
YBR140C	YNL098C
YBR140C	YPL204W
YBR141C	YDR372C
YBR141C	YER059W
YBR141C	YGL091C
YBR142W	YDR060W
YBR142W	YDR170C
YBR142W	YDR466W
YBR142W	YDR496C
YBR142W	YGL111W
YBR142W	YGL195W
YBR142W	YHR052W
YBR142W	YHR066W
YBR142W	YJL005W
YBR142W	YJL095W
YBR142W	YKL014C
YBR142W	YLL019C
YBR142W	YLR398C
YBR142W	YMR012W
YBR142W	YMR049C
YBR142W	YMR163C
YBR142W	YMR290C
YBR142W	YNL002C
YBR142W	YNL061W

YBR142W YOL041C
YBR142W YOR272W
YBR142W YPL043W
YBR142W YPL093W
YBR142W YPL141C
YBR143C YDL058W
YBR143C YDR172W
YBR143C YER081W
YBR143C YER110C
YBR143C YER161C
YBR143C YHL030W
YBR143C YIL129C
YBR143C YJL016W
YBR143C YLR371W
YBR143C YLR386W
YBR143C YLR429W
YBR143C YMR012W
YBR143C YMR080C
YBR143C YPL043W
YBR143C YPL083C
YBR143C YPR010C
YBR144C YDR034C
YBR144C YMR047C
YBR145W YDR034C
YBR145W YDR499W
YBR145W YMR047C
YBR146W YBR251W
YBR146W YDR036C
YBR146W YHL004W
YBR146W YIL070C
YBR149W YDL211C
YBR149W YNL128W
YBR150C YDR388W
YBR150C YPR110C
YBR152W YDL030W
YBR152W YDL043C
YBR152W YDL098C
YBR152W YDR473C
YBR152W YER029C
YBR152W YER172C
YBR152W YGR075C
YBR152W YGR091W
YBR152W YIR009W
YBR152W YJL203W
YBR152W YJR091C

YBR152W	YKL173W
YBR152W	YML049C
YBR152W	YMR240C
YBR152W	YMR288W
YBR152W	YOR308C
YBR152W	YPL213W
YBR152W	YPR178W
YBR154C	YDL140C
YBR154C	YDR404C
YBR154C	YER071C
YBR154C	YER125W
YBR154C	YFL023W
YBR154C	YGL070C
YBR154C	YIL021W
YBR154C	YJL140W
YBR154C	YJR063W
YBR154C	YKL144C
YBR154C	YLR384C
YBR154C	YNR003C
YBR154C	YOR116C
YBR154C	YOR151C
YBR154C	YOR224C
YBR154C	YOR341W
YBR154C	YPL203W
YBR154C	YPR110C
YBR155W	YDR463W
YBR155W	YEL030W
YBR155W	YER081W
YBR155W	YER177W
YBR155W	YGR187C
YBR155W	YGR234W
YBR155W	YJR032W
YBR155W	YJR045C
YBR155W	YJR091C
YBR155W	YJR121W
YBR155W	YLL026W
YBR155W	YLR355C
YBR155W	YMR186W
YBR155W	YPL240C
YBR156C	YGL061C
YBR156C	YGR113W
YBR156C	YPL209C
YBR157C	YGL070C
YBR158W	YDR111C
YBR158W	YGR163W

YBR158W YGR285C
YBR158W YHR064C
YBR159W YBR183W
YBR159W YBR283C
YBR159W YBR290W
YBR159W YBR293W
YBR159W YBR298C
YBR159W YBR302C
YBR159W YCL025C
YBR159W YCR011C
YBR159W YCR024C-A
YBR159W YDL015C
YBR159W YDL054C
YBR159W YDL206W
YBR159W YDL210W
YBR159W YDR046C
YBR159W YDR297W
YBR159W YDR307W
YBR159W YDR331W
YBR159W YDR414C
YBR159W YDR468C
YBR159W YDR508C
YBR159W YEL002C
YBR159W YEL017C-A
YBR159W YEL063C
YBR159W YFL048C
YBR159W YFL062W
YBR159W YGL055W
YBR159W YGL104C
YBR159W YGL200C
YBR159W YGR060W
YBR159W YGR172C
YBR159W YGR191W
YBR159W YGR260W
YBR159W YGR295C
YBR159W YHL042W
YBR159W YHL048W
YBR159W YHR026W
YBR159W YHR110W
YBR159W YHR123W
YBR159W YHR133C
YBR159W YHR140W
YBR159W YHR142W
YBR159W YIL005W
YBR159W YIL120W

YBR159W YIR022W
YBR159W YJL196C
YBR159W YJL214W
YBR159W YJL219W
YBR159W YJL222W
YBR159W YJR010C-A
YBR159W YJR015W
YBR159W YJR161C
YBR159W YKL008C
YBR159W YKL146W
YBR159W YKL165C
YBR159W YKL212W
YBR159W YKR039W
YBR159W YKR106W
YBR159W YLL023C
YBR159W YLL028W
YBR159W YLL043W
YBR159W YLL061W
YBR159W YLR004C
YBR159W YLR056W
YBR159W YLR080W
YBR159W YLR083C
YBR159W YLR241W
YBR159W YLR292C
YBR159W YLR378C
YBR159W YML048W
YBR159W YML067C
YBR159W YML075C
YBR159W YML123C
YBR159W YMR058W
YBR159W YMR149W
YBR159W YMR215W
YBR159W YMR279C
YBR159W YNL048W
YBR159W YNL101W
YBR159W YNL130C
YBR159W YNL279W
YBR159W YNR013C
YBR159W YNR019W
YBR159W YNR070W
YBR159W YOL132W
YBR159W YOL156W
YBR159W YPL076W
YBR159W YPL189W
YBR159W YPL264C

YBR159W YPL274W
YBR159W YPR003C
YBR159W YPR124W
YBR159W YPR156C
YBR159W YPR194C
YBR159W YPR198W
YBR159W YPR201W
YBR160W YBR200W
YBR160W YCL014W
YBR160W YCL027W
YBR160W YCL040W
YBR160W YCL051W
YBR160W YCR065W
YBR160W YDL025C
YBR160W YDL089W
YBR160W YDL106C
YBR160W YDL113C
YBR160W YDL155W
YBR160W YDL189W
YBR160W YDL239C
YBR160W YDR001C
YBR160W YDR027C
YBR160W YDR052C
YBR160W YDR093W
YBR160W YDR097C
YBR160W YDR113C
YBR160W YDR123C
YBR160W YDR130C
YBR160W YDR146C
YBR160W YDR171W
YBR160W YDR212W
YBR160W YDR217C
YBR160W YDR223W
YBR160W YDR227W
YBR160W YDR285W
YBR160W YDR348C
YBR160W YDR356W
YBR160W YDR379W
YBR160W YDR389W
YBR160W YDR439W
YBR160W YDR501W
YBR160W YDR507C
YBR160W YEL032W
YBR160W YEL065W
YBR160W YER008C

YBR160W YER032W
YBR160W YER041W
YBR160W YER059W
YBR160W YER120W
YBR160W YER129W
YBR160W YER158C
YBR160W YER167W
YBR160W YFL029C
YBR160W YFL037W
YBR160W YFR015C
YBR160W YFR027W
YBR160W YFR030W
YBR160W YFR046C
YBR160W YFR053C
YBR160W YGL003C
YBR160W YGL075C
YBR160W YGL097W
YBR160W YGL116W
YBR160W YGL124C
YBR160W YGL178W
YBR160W YGL206C
YBR160W YGL216W
YBR160W YGL235W
YBR160W YGR014W
YBR160W YGR035C
YBR160W YGR092W
YBR160W YGR108W
YBR160W YGR109C
YBR160W YGR186W
YBR160W YGR221C
YBR160W YGR238C
YBR160W YGR270W
YBR160W YGR296W
YBR160W YHL022C
YBR160W YHL035C
YBR160W YHL050C
YBR160W YHR027C
YBR160W YHR098C
YBR160W YHR118C
YBR160W YHR149C
YBR160W YHR158C
YBR160W YHR159W
YBR160W YHR164C
YBR160W YIL031W
YBR160W YIL050W

YBR160W YIL075C
YBR160W YIL101C
YBR160W YIL106W
YBR160W YIL112W
YBR160W YIL122W
YBR160W YIL131C
YBR160W YIL140W
YBR160W YIR023W
YBR160W YIR031C
YBR160W YJL008C
YBR160W YJL060W
YBR160W YJL076W
YBR160W YJL084C
YBR160W YJL092W
YBR160W YJL157C
YBR160W YJL187C
YBR160W YJL194W
YBR160W YJR033C
YBR160W YJR054W
YBR160W YJR059W
YBR160W YJR083C
YBR160W YJR090C
YBR160W YJR091C
YBR160W YJR092W
YBR160W YKL014C
YBR160W YKL043W
YBR160W YKL048C
YBR160W YKL108W
YBR160W YKL116C
YBR160W YKL129C
YBR160W YKL185W
YBR160W YKR077W
YBR160W YKR078W
YBR160W YKR089C
YBR160W YKR090W
YBR160W YKR091W
YBR160W YKR095W
YBR160W YLL003W
YBR160W YLL021W
YBR160W YLR006C
YBR160W YLR035C
YBR160W YLR045C
YBR160W YLR079W
YBR160W YLR086W
YBR160W YLR096W

YBR160W YLR131C
YBR160W YLR180W
YBR160W YLR182W
YBR160W YLR183C
YBR160W YLR187W
YBR160W YLR190W
YBR160W YLR210W
YBR160W YLR219W
YBR160W YLR223C
YBR160W YLR238W
YBR160W YLR259C
YBR160W YLR278C
YBR160W YLR319C
YBR160W YLR394W
YBR160W YLR401C
YBR160W YLR425W
YBR160W YLR430W
YBR160W YLR457C
YBR160W YML027W
YBR160W YML034W
YBR160W YML065W
YBR160W YML085C
YBR160W YML119W
YBR160W YMR001C
YBR160W YMR005W
YBR160W YMR012W
YBR160W YMR036C
YBR160W YMR129W
YBR160W YMR199W
YBR160W YMR205C
YBR160W YNL042W
YBR160W YNL058C
YBR160W YNL064C
YBR160W YNL068C
YBR160W YNL071W
YBR160W YNL102W
YBR160W YNL257C
YBR160W YNL271C
YBR160W YNL278W
YBR160W YNL298W
YBR160W YNL309W
YBR160W YNL321W
YBR160W YNL339C
YBR160W YNR047W
YBR160W YOL036W

YBR160W YOL058W
YBR160W YOL070C
YBR160W YOL100W
YBR160W YOR001W
YBR160W YOR014W
YBR160W YOR037W
YBR160W YOR058C
YBR160W YOR066W
YBR160W YOR075W
YBR160W YOR081C
YBR160W YOR083W
YBR160W YOR098C
YBR160W YOR104W
YBR160W YOR127W
YBR160W YOR151C
YBR160W YOR177C
YBR160W YOR178C
YBR160W YOR188W
YBR160W YOR195W
YBR160W YOR315W
YBR160W YOR372C
YBR160W YPL014W
YBR160W YPL070W
YBR160W YPL115C
YBR160W YPL155C
YBR160W YPL194W
YBR160W YPL209C
YBR160W YPL235W
YBR160W YPL250C
YBR160W YPL255W
YBR160W YPL256C
YBR160W YPL267W
YBR160W YPR018W
YBR160W YPR030W
YBR160W YPR111W
YBR160W YPR119W
YBR160W YPR120C
YBR160W YPR141C
YBR160W YPR171W
YBR160W YPR174C
YBR160W YPR175W
YBR161W YEL042W
YBR161W YJR001W
YBR161W YJR091C
YBR162C YBR192W

YBR162C	YDR238C
YBR162C	YER086W
YBR162C	YGR249W
YBR162C	YMR214W
YBR162C	YNL217W
YBR162C	YNL287W
YBR162C	YOL028C
YBR162C	YPR155C
YBR162W-A	YIL027C
YBR162W-A	YLR090W
YBR162W-A	YML108W
YBR163W	YDR388W
YBR166C	YBR217W
YBR166C	YLR447C
YBR167C	YBR257W
YBR167C	YGL070C
YBR167C	YGR030C
YBR167C	YHR062C
YBR167C	YNL221C
YBR167C	YNL282W
YBR168W	YER081W
YBR169C	YCR002C
YBR169C	YCR009C
YBR169C	YDL005C
YBR169C	YDL225W
YBR169C	YGL190C
YBR169C	YGL206C
YBR169C	YHR199C
YBR169C	YJL098W
YBR169C	YJL128C
YBR169C	YKL130C
YBR169C	YKL139W
YBR169C	YNL007C
YBR169C	YNL127W
YBR170C	YDL126C
YBR170C	YDL190C
YBR170C	YDR259C
YBR170C	YER081W
YBR170C	YGR048W
YBR170C	YLR044C
YBR171W	YCL057W
YBR171W	YER053C
YBR171W	YHL042W
YBR171W	YHR041C
YBR171W	YLR292C

YBR171W	YLR378C
YBR171W	YML064C
YBR171W	YOR254C
YBR171W	YPL076W
YBR171W	YPL094C
YBR171W	YPL227C
YBR172C	YCL027W
YBR172C	YKL074C
YBR172C	YLR116W
YBR173C	YGL011C
YBR173C	YIL034C
YBR173C	YKR086W
YBR173C	YPR103W
YBR174C	YJR091C
YBR175W	YBR258C
YBR175W	YDL126C
YBR175W	YDR138W
YBR175W	YDR171W
YBR175W	YDR469W
YBR175W	YGR249W
YBR175W	YHR027C
YBR175W	YHR064C
YBR175W	YHR119W
YBR175W	YHR177W
YBR175W	YIL160C
YBR175W	YKL018W
YBR175W	YLR015W
YBR175W	YLR259C
YBR175W	YLR403W
YBR175W	YMR190C
YBR175W	YOL045W
YBR175W	YOR047C
YBR175W	YPL029W
YBR175W	YPL138C
YBR175W	YPR086W
YBR176W	YGL070C
YBR176W	YGL127C
YBR176W	YLR291C
YBR176W	YLR347C
YBR176W	YML064C
YBR176W	YNL189W
YBR176W	YPL070W
YBR177C	YHR114W
YBR178W	YHR122W
YBR179C	YDR142C

YBR179C	YHR041C
YBR180W	YDR034C
YBR180W	YHR042W
YBR182C	YKL129C
YBR183W	YBR290W
YBR183W	YCR034W
YBR183W	YDL212W
YBR183W	YDR039C
YBR183W	YDR276C
YBR183W	YDR331W
YBR183W	YDR506C
YBR183W	YER087C-B
YBR183W	YFL041W
YBR183W	YFL048C
YBR183W	YGR060W
YBR183W	YGR284C
YBR183W	YHL048W
YBR183W	YHR140W
YBR183W	YIL016W
YBR183W	YJL117W
YBR183W	YJR117W
YBR183W	YKL065C
YBR183W	YKL154W
YBR183W	YLR018C
YBR183W	YLR372W
YBR183W	YML048W
YBR183W	YML067C
YBR183W	YMR149W
YBR183W	YNL101W
YBR183W	YNL169C
YBR183W	YOL132W
YBR183W	YOR016C
YBR183W	YOR254C
YBR183W	YPL076W
YBR183W	YPL227C
YBR184W	YER171W
YBR184W	YJR091C
YBR184W	YNL225C
YBR185C	YJR063W
YBR186W	YGL127C
YBR186W	YLR373C
YBR187W	YCR061W
YBR187W	YGL137W
YBR187W	YJR063W
YBR187W	YNR032W

YBR187W	YPR171W
YBR188C	YDR416W
YBR188C	YGL070C
YBR188C	YIL034C
YBR188C	YLL036C
YBR188C	YLR117C
YBR190W	YLR117C
YBR192W	YER078C
YBR192W	YHR114W
YBR193C	YBR253W
YBR193C	YDL005C
YBR193C	YEL037C
YBR193C	YER012W
YBR193C	YER022W
YBR193C	YER148W
YBR193C	YGL127C
YBR193C	YGR104C
YBR193C	YHR041C
YBR193C	YLR295C
YBR193C	YMR047C
YBR193C	YOL051W
YBR193C	YOL135C
YBR193C	YOR264W
YBR193C	YPR086W
YBR194W	YPR152C
YBR195C	YHR004C
YBR195C	YKR029C
YBR195C	YLR259C
YBR195C	YLR418C
YBR195C	YML102W
YBR195C	YMR066W
YBR195C	YOR100C
YBR195C	YOR308C
YBR195C	YPL022W
YBR195C	YPR018W
YBR196C	YBR203W
YBR196C	YBR223C
YBR196C	YCL039W
YBR196C	YCR002C
YBR196C	YCR009C
YBR196C	YCR079W
YBR196C	YCR088W
YBR196C	YDL029W
YBR196C	YDL043C
YBR196C	YDL132W

YBR196C	YDL175C
YBR196C	YDL200C
YBR196C	YDL213C
YBR196C	YDR030C
YBR196C	YDR092W
YBR196C	YDR113C
YBR196C	YDR131C
YBR196C	YDR146C
YBR196C	YDR164C
YBR196C	YDR217C
YBR196C	YDR227W
YBR196C	YDR316W
YBR196C	YDR369C
YBR196C	YDR388W
YBR196C	YDR436W
YBR196C	YER017C
YBR196C	YER095W
YBR196C	YER116C
YBR196C	YER125W
YBR196C	YER133W
YBR196C	YER171W
YBR196C	YER173W
YBR196C	YER179W
YBR196C	YFR028C
YBR196C	YGL003C
YBR196C	YGL004C
YBR196C	YGL163C
YBR196C	YGL190C
YBR196C	YGL229C
YBR196C	YGL237C
YBR196C	YGR040W
YBR196C	YGR067C
YBR196C	YGR092W
YBR196C	YGR123C
YBR196C	YGR188C
YBR196C	YHL007C
YBR196C	YHR030C
YBR196C	YHR107C
YBR196C	YHR120W
YBR196C	YHR135C
YBR196C	YHR166C
YBR196C	YIL007C
YBR196C	YIL046W
YBR196C	YIL061C
YBR196C	YIL113W

YBR196C YJL069C
YBR196C YJL128C
YBR196C YJL138C
YBR196C YJL173C
YBR196C YJL187C
YBR196C YJR007W
YBR196C YJR035W
YBR196C YJR062C
YBR196C YJR090C
YBR196C YKL048C
YBR196C YKL130C
YBR196C YKL189W
YBR196C YKR017C
YBR196C YKR026C
YBR196C YKR055W
YBR196C YLL019C
YBR196C YLL050C
YBR196C YLR186W
YBR196C YLR222C
YBR196C YLR262C
YBR196C YLR288C
YBR196C YLR314C
YBR196C YML095C
YBR196C YMR049C
YBR196C YMR059W
YBR196C YMR094W
YBR196C YMR106C
YBR196C YMR291W
YBR196C YNL032W
YBR196C YNL053W
YBR196C YNL088W
YBR196C YNL135C
YBR196C YNL182C
YBR196C YNL183C
YBR196C YNL189W
YBR196C YNL244C
YBR196C YNL250W
YBR196C YNL260C
YBR196C YNL290W
YBR196C YNL323W
YBR196C YNR047W
YBR196C YOL100W
YBR196C YOL113W
YBR196C YOL128C
YBR196C YOR212W

YBR196C	YOR276W
YBR196C	YOR339C
YBR196C	YOR351C
YBR196C	YOR353C
YBR196C	YPL022W
YBR196C	YPL074W
YBR196C	YPL150W
YBR196C	YPL153C
YBR196C	YPL164C
YBR196C	YPL204W
YBR196C	YPL262W
YBR196C	YPR110C
YBR197C	YKL002W
YBR197C	YLR288C
YBR197C	YNR037C
YBR198C	YCL018W
YBR198C	YCR042C
YBR198C	YDL143W
YBR198C	YDR145W
YBR198C	YDR148C
YBR198C	YDR167W
YBR198C	YDR176W
YBR198C	YDR212W
YBR198C	YDR216W
YBR198C	YDR259C
YBR198C	YDR448W
YBR198C	YEL009C
YBR198C	YER148W
YBR198C	YER160C
YBR198C	YFL037W
YBR198C	YGL048C
YBR198C	YGL066W
YBR198C	YGL112C
YBR198C	YGL234W
YBR198C	YGR220C
YBR198C	YGR274C
YBR198C	YHR020W
YBR198C	YHR041C
YBR198C	YHR099W
YBR198C	YHR137W
YBR198C	YIL142W
YBR198C	YJL008C
YBR198C	YJL014W
YBR198C	YJL111W
YBR198C	YJR064W

YBR198C	YJR072C
YBR198C	YKL081W
YBR198C	YLR259C
YBR198C	YML015C
YBR198C	YML061C
YBR198C	YML098W
YBR198C	YMR236W
YBR198C	YOL055C
YBR198C	YOR119C
YBR198C	YOR308C
YBR198C	YPL011C
YBR198C	YPR072W
YBR198C	YPR154W
YBR199W	YDL132W
YBR199W	YDR384C
YBR199W	YOL132W
YBR200W	YBR289W
YBR200W	YCL005W
YBR200W	YCL031C
YBR200W	YDL028C
YBR200W	YDL086W
YBR200W	YDL146W
YBR200W	YDR103W
YBR200W	YDR177W
YBR200W	YDR239C
YBR200W	YDR242W
YBR200W	YDR243C
YBR200W	YDR277C
YBR200W	YDR306C
YBR200W	YDR368W
YBR200W	YEL043W
YBR200W	YER114C
YBR200W	YFL039C
YBR200W	YGL190C
YBR200W	YGL233W
YBR200W	YGR134W
YBR200W	YGR152C
YBR200W	YHL007C
YBR200W	YIL060W
YBR200W	YIL156W
YBR200W	YIR003W
YBR200W	YJL079C
YBR200W	YJL157C
YBR200W	YJL187C
YBR200W	YKL040C

YBR200W YKL082C
YBR200W YKR105C
YBR200W YLL049W
YBR200W YLR229C
YBR200W YLR318W
YBR200W YLR337C
YBR200W YLR361C
YBR200W YLR425W
YBR200W YMR109W
YBR200W YMR195W
YBR200W YMR212C
YBR200W YMR247C
YBR200W YMR287C
YBR200W YNL025C
YBR200W YNL094W
YBR200W YNL298W
YBR200W YNR047W
YBR200W YNR067C
YBR200W YNR071C
YBR200W YOL078W
YBR200W YOL093W
YBR200W YOR047C
YBR200W YOR108W
YBR200W YOR109W
YBR200W YOR129C
YBR200W YOR181W
YBR200W YOR249C
YBR200W YPL038W
YBR200W YPL085W
YBR200W YPL105C
YBR201W YGL070C
YBR201W YIL034C
YBR201W YLR288C
YBR202W YDL029W
YBR202W YEL032W
YBR202W YGL001C
YBR202W YIL150C
YBR202W YLR274W
YBR202W YPR019W
YBR203W YCL037C
YBR203W YDR328C
YBR203W YJL008C
YBR203W YKL006C-A
YBR203W YKR048C
YBR203W YPL061W

YBR204C	YPL096W
YBR205W	YDL101C
YBR205W	YDR170C
YBR205W	YFL038C
YBR205W	YFR024C-A
YBR205W	YGL212W
YBR205W	YHR016C
YBR205W	YHR105W
YBR205W	YHR114W
YBR205W	YML115C
YBR205W	YPR107C
YBR207W	YEL022W
YBR207W	YER021W
YBR207W	YER110C
YBR207W	YFL041W
YBR207W	YGR152C
YBR207W	YLR342W
YBR207W	YOL086C
YBR207W	YOL132W
YBR207W	YOR068C
YBR208C	YBR221C
YBR208C	YCL018W
YBR208C	YDL029W
YBR208C	YDL126C
YBR208C	YHR020W
YBR208C	YJL098W
YBR208C	YKL060C
YBR208C	YOL133W
YBR209W	YPR086W
YBR211C	YDL028C
YBR211C	YDR394W
YBR211C	YGR179C
YBR211C	YKL002W
YBR211C	YLR052W
YBR211C	YOR269W
YBR212W	YDL167C
YBR213W	YMR206W
YBR214W	YDL017W
YBR214W	YJL124C
YBR215W	YLR453C
YBR216C	YKL068W
YBR216C	YML007W
YBR216C	YMR047C
YBR217W	YCL018W
YBR217W	YDL029W

YBR217W YDL055C
YBR217W YDL097C
YBR217W YDL126C
YBR217W YDL147W
YBR217W YDR022C
YBR217W YDR127W
YBR217W YDR148C
YBR217W YDR214W
YBR217W YDR394W
YBR217W YDR465C
YBR217W YDR502C
YBR217W YEL060C
YBR217W YER021W
YBR217W YER025W
YBR217W YER110C
YBR217W YER178W
YBR217W YFL037W
YBR217W YFL039C
YBR217W YFR004W
YBR217W YFR030W
YBR217W YGL016W
YBR217W YGL048C
YBR217W YGL078C
YBR217W YGL105W
YBR217W YGL206C
YBR217W YGL234W
YBR217W YGL245W
YBR217W YGR218W
YBR217W YGR240C
YBR217W YGR282C
YBR217W YHR020W
YBR217W YHR027C
YBR217W YHR033W
YBR217W YHR076W
YBR217W YHR171W
YBR217W YHR200W
YBR217W YHR201C
YBR217W YIL125W
YBR217W YIL128W
YBR217W YJL066C
YBR217W YJL130C
YBR217W YJL138C
YBR217W YJR045C
YBR217W YJR077C
YBR217W YJR109C

YBR217W	YJR121W
YBR217W	YKL035W
YBR217W	YKL104C
YBR217W	YKL145W
YBR217W	YLL042C
YBR217W	YLR031W
YBR217W	YLR059C
YBR217W	YLR180W
YBR217W	YLR216C
YBR217W	YLR423C
YBR217W	YLR424W
YBR217W	YLR438W
YBR217W	YML055W
YBR217W	YML085C
YBR217W	YML124C
YBR217W	YMR047C
YBR217W	YMR056C
YBR217W	YMR058W
YBR217W	YMR159C
YBR217W	YMR319C
YBR217W	YNL071W
YBR217W	YNL208W
YBR217W	YNR001C
YBR217W	YNR007C
YBR217W	YOL055C
YBR217W	YOR086C
YBR217W	YOR151C
YBR217W	YPL061W
YBR217W	YPL149W
YBR217W	YPL235W
YBR217W	YPR049C
YBR217W	YPR108W
YBR218C	YER179W
YBR218C	YHR135C
YBR218C	YKL103C
YBR218C	YKR055W
YBR218C	YLR383W
YBR218C	YPL031C
YBR218C	YPR110C
YBR219C	YJR063W
YBR220C	YJR063W
YBR221C	YCR079W
YBR221C	YDR069C
YBR221C	YDR430C
YBR221C	YER081W

YBR221C	YER178W
YBR221C	YFL018C
YBR221C	YFR004W
YBR221C	YGL130W
YBR221C	YGR193C
YBR221C	YLR218C
YBR221C	YLR345W
YBR221C	YML059C
YBR221C	YMR308C
YBR221C	YNL071W
YBR221C	YPL151C
YBR221C	YPL259C
YBR222C	YFL020C
YBR223C	YCL028W
YBR223C	YDL126C
YBR223C	YDR510W
YBR223C	YFL018C
YBR223C	YJL088W
YBR223C	YJR045C
YBR223C	YLR180W
YBR223C	YNL064C
YBR223C	YNL071W
YBR223C	YOL055C
YBR225W	YGL197W
YBR225W	YLR310C
YBR225W	YNL307C
YBR225W	YPL203W
YBR225W	YPL204W
YBR226C	YDL049C
YBR227C	YKR026C
YBR227C	YML064C
YBR228W	YJR063W
YBR228W	YLR135W
YBR228W	YMR106C
YBR229C	YDL210W
YBR230C	YDR506C
YBR230C	YOR014W
YBR231C	YDR334W
YBR231C	YGL070C
YBR231C	YGR002C
YBR231C	YJR091C
YBR231C	YNR030W
YBR231C	YOL012C
YBR233W	YDR174W
YBR233W	YDR381W

YBR233W	YLR249W
YBR233W	YMR173W
YBR234C	YDL029W
YBR234C	YER022W
YBR234C	YER165W
YBR234C	YGL173C
YBR234C	YGR254W
YBR234C	YHR064C
YBR234C	YIL062C
YBR234C	YJL052W
YBR234C	YJR065C
YBR234C	YKL013C
YBR234C	YKL060C
YBR234C	YKL129C
YBR234C	YLR044C
YBR234C	YLR241W
YBR234C	YLR370C
YBR234C	YLR453C
YBR234C	YML069W
YBR234C	YMR109W
YBR234C	YMR309C
YBR234C	YNL040W
YBR234C	YNR035C
YBR234C	YPL043W
YBR235W	YGR218W
YBR235W	YLR034C
YBR236C	YDL140C
YBR236C	YML010W
YBR236C	YOR151C
YBR237W	YDR073W
YBR238C	YKL104C
YBR238C	YKR043C
YBR239C	YDR388W
YBR239C	YFR024C-A
YBR239C	YGR136W
YBR239C	YHR016C
YBR239C	YMR144W
YBR239C	YOR317W
YBR239C	YPL133C
YBR240C	YDL080C
YBR240C	YER081W
YBR241C	YER081W
YBR242W	YKL002W
YBR242W	YOR272W
YBR243C	YER081W

YBR243C	YML075C
YBR244W	YLR117C
YBR245C	YCR052W
YBR245C	YDL002C
YBR245C	YER164W
YBR245C	YFR013W
YBR245C	YFR037C
YBR245C	YGL133W
YBR245C	YKR001C
YBR245C	YKR008W
YBR245C	YLR033W
YBR245C	YLR163C
YBR245C	YLR176C
YBR245C	YLR357W
YBR245C	YMR091C
YBR245C	YOL004W
YBR245C	YOL017W
YBR245C	YOR304W
YBR245C	YPL082C
YBR245C	YPR110C
YBR246W	YDR520C
YBR247C	YCL059C
YBR247C	YCR057C
YBR247C	YCR073C
YBR247C	YDL014W
YBR247C	YDL060W
YBR247C	YDL132W
YBR247C	YDL148C
YBR247C	YDR449C
YBR247C	YER017C
YBR247C	YER082C
YBR247C	YGR081C
YBR247C	YGR090W
YBR247C	YHR148W
YBR247C	YIL061C
YBR247C	YJR002W
YBR247C	YKL143W
YBR247C	YLL011W
YBR247C	YLR175W
YBR247C	YLR180W
YBR247C	YLR186W
YBR247C	YMR093W
YBR247C	YMR128W
YBR247C	YNL075W
YBR247C	YNL132W

YBR247C	YNL207W
YBR247C	YNL244C
YBR247C	YOL010W
YBR247C	YOR056C
YBR247C	YOR080W
YBR247C	YPL012W
YBR247C	YPL204W
YBR247C	YPR144C
YBR248C	YOL102C
YBR249C	YEL047C
YBR249C	YER081W
YBR249C	YOR050C
YBR249C	YPL150W
YBR249C	YPR110C
YBR250W	YML038C
YBR250W	YNL012W
YBR251W	YDL045W-A
YBR251W	YDL138W
YBR251W	YDR036C
YBR251W	YDR041W
YBR251W	YDR175C
YBR251W	YDR337W
YBR251W	YDR347W
YBR251W	YER155C
YBR251W	YGL129C
YBR251W	YGR084C
YBR251W	YGR090W
YBR251W	YGR091W
YBR251W	YGR150C
YBR251W	YGR162W
YBR251W	YGR170W
YBR251W	YGR215W
YBR251W	YHL004W
YBR251W	YHR059W
YBR251W	YHR197W
YBR251W	YIL093C
YBR251W	YJR101W
YBR251W	YJR113C
YBR251W	YKL003C
YBR251W	YKL155C
YBR251W	YMR128W
YBR251W	YNL137C
YBR251W	YNL186W
YBR251W	YNL306W
YBR251W	YOL115W

YBR251W	YOR205C
YBR251W	YOR243C
YBR251W	YPL013C
YBR251W	YPL118W
YBR252W	YLL037W
YBR252W	YML064C
YBR252W	YNL189W
YBR252W	YPL070W
YBR253W	YCR081W
YBR253W	YDL005C
YBR253W	YDL140C
YBR253W	YDR308C
YBR253W	YDR443C
YBR253W	YDR448W
YBR253W	YER022W
YBR253W	YGL025C
YBR253W	YGL112C
YBR253W	YGL151W
YBR253W	YGR104C
YBR253W	YGR252W
YBR253W	YHR041C
YBR253W	YHR058C
YBR253W	YKR095W
YBR253W	YLR071C
YBR253W	YLR288C
YBR253W	YML007W
YBR253W	YMR112C
YBR253W	YNL025C
YBR253W	YNL236W
YBR253W	YNR010W
YBR253W	YOL051W
YBR253W	YOL135C
YBR253W	YOR174W
YBR253W	YPL042C
YBR253W	YPL248C
YBR253W	YPR070W
YBR253W	YPR168W
YBR254C	YDR108W
YBR254C	YDR246W
YBR254C	YDR407C
YBR254C	YDR472W
YBR254C	YGR143W
YBR254C	YGR166W
YBR254C	YHR041C
YBR254C	YJR116W

YBR254C	YKR068C
YBR254C	YLR342W
YBR254C	YML077W
YBR254C	YMR218C
YBR254C	YOR115C
YBR254C	YPL235W
YBR255W	YIL106W
YBR258C	YDR469W
YBR258C	YHR119W
YBR258C	YLR015W
YBR258C	YPL138C
YBR260C	YGR196C
YBR260C	YHR107C
YBR260C	YIL118W
YBR260C	YJL058C
YBR260C	YKR055W
YBR260C	YMR032W
YBR260C	YOR109W
YBR260C	YOR181W
YBR260C	YOR231W
YBR260C	YPL140C
YBR261C	YDR465C
YBR262C	YKL002W
YBR263W	YIL018W
YBR263W	YML018C
YBR264C	YCL040W
YBR264C	YER136W
YBR264C	YER177W
YBR264C	YFR053C
YBR264C	YGL161C
YBR264C	YGL198W
YBR264C	YIL034C
YBR264C	YKL152C
YBR264C	YNL263C
YBR264C	YOR370C
YBR265W	YCR107W
YBR265W	YLR255C
YBR266C	YPR031W
YBR267W	YDR101C
YBR267W	YGL099W
YBR267W	YHR170W
YBR267W	YJL122W
YBR267W	YJR045C
YBR269C	YLR276C
YBR270C	YCR077C

YBR270C	YDR259C
YBR270C	YER093C
YBR270C	YFL047W
YBR270C	YFR050C
YBR270C	YGR249W
YBR270C	YHR077C
YBR270C	YHR166C
YBR270C	YIL046W
YBR270C	YIL105C
YBR270C	YJL039C
YBR270C	YKR026C
YBR270C	YLR117C
YBR270C	YLR423C
YBR270C	YLR424W
YBR270C	YMR236W
YBR270C	YNL004W
YBR270C	YNL018C
YBR270C	YNL047C
YBR270C	YOR047C
YBR270C	YPL124W
YBR270C	YPL255W
YBR271W	YJR091C
YBR271W	YJR121W
YBR272C	YDL007W
YBR272C	YDL097C
YBR272C	YDR363W-A
YBR272C	YDR394W
YBR272C	YDR427W
YBR272C	YEL034W
YBR272C	YEL056W
YBR272C	YER021W
YBR272C	YFR052W
YBR272C	YGL004C
YBR272C	YGL048C
YBR272C	YGL181W
YBR272C	YGR232W
YBR272C	YKL145W
YBR272C	YLL022C
YBR272C	YLR015W
YBR272C	YNL030W
YBR272C	YOL054W
YBR272C	YOR117W
YBR272C	YOR261C
YBR272C	YPL001W
YBR272C	YPR103W

YBR273C	YMR047C
YBR274W	YCL018W
YBR274W	YDL042C
YBR274W	YDL055C
YBR274W	YDR113C
YBR274W	YDR168W
YBR274W	YDR217C
YBR274W	YDR439W
YBR274W	YER081W
YBR274W	YER171W
YBR274W	YFL039C
YBR274W	YFR028C
YBR274W	YIL084C
YBR274W	YJL036W
YBR274W	YJL076W
YBR274W	YJL124C
YBR274W	YJL130C
YBR274W	YKL104C
YBR274W	YKR010C
YBR274W	YLR152C
YBR274W	YLR180W
YBR274W	YLR258W
YBR274W	YMR102C
YBR274W	YMR205C
YBR274W	YMR255W
YBR274W	YMR270C
YBR274W	YNL064C
YBR274W	YNL234W
YBR274W	YNR013C
YBR274W	YOR151C
YBR274W	YOR341W
YBR274W	YPR010C-A
YBR274W	YPR124W
YBR275C	YDL042C
YBR275C	YDL230W
YBR275C	YER114C
YBR275C	YER133W
YBR275C	YFR028C
YBR275C	YGR136W
YBR275C	YIL035C
YBR275C	YLR019W
YBR275C	YLR453C
YBR275C	YNL216W
YBR275C	YOR208W
YBR276C	YLR359W

YBR277C	YIL034C
YBR278W	YDR121W
YBR278W	YDR448W
YBR278W	YIL006W
YBR278W	YJR091C
YBR278W	YKL011C
YBR278W	YLR181C
YBR278W	YMR290C
YBR278W	YNL262W
YBR278W	YPR175W
YBR279W	YDL140C
YBR279W	YDR138W
YBR279W	YGL207W
YBR279W	YGL244W
YBR279W	YGR104C
YBR279W	YHL002W
YBR279W	YKL145W
YBR279W	YLR418C
YBR279W	YML010W
YBR279W	YOL145C
YBR279W	YOR123C
YBR279W	YPL129W
YBR280C	YDL132W
YBR280C	YDR328C
YBR280C	YEL060C
YBR280C	YJR045C
YBR280C	YNL141W
YBR280C	YOL133W
YBR281C	YDL153C
YBR281C	YML064C
YBR281C	YMR093W
YBR281C	YNL191W
YBR281C	YNL287W
YBR282W	YGR187C
YBR282W	YNL284C
YBR283C	YCR034W
YBR283C	YGL051W
YBR283C	YGR284C
YBR283C	YHR140W
YBR283C	YIL016W
YBR283C	YIL162W
YBR283C	YJL117W
YBR283C	YJR117W
YBR283C	YKL008C
YBR283C	YKL065C

YBR283C	YKL154W
YBR283C	YLR311C
YBR283C	YLR372W
YBR283C	YML048W
YBR283C	YMR215W
YBR283C	YMR264W
YBR283C	YOR254C
YBR283C	YPL076W
YBR283C	YPL234C
YBR283C	YPR028W
YBR284W	YDL238C
YBR284W	YJR090C
YBR284W	YMR138W
YBR285W	YIL049W
YBR285W	YPR156C
YBR286W	YDR311W
YBR286W	YGL100W
YBR286W	YPL200W
YBR287W	YPL235W
YBR288C	YCL018W
YBR288C	YDL055C
YBR288C	YDR394W
YBR288C	YER110C
YBR288C	YFL037W
YBR288C	YFL039C
YBR288C	YGL206C
YBR288C	YGR254W
YBR288C	YGR261C
YBR288C	YJL008C
YBR288C	YJL024C
YBR288C	YJL066C
YBR288C	YJR045C
YBR288C	YJR077C
YBR288C	YJR121W
YBR288C	YKL060C
YBR288C	YLR044C
YBR288C	YLR180W
YBR288C	YLR259C
YBR288C	YML085C
YBR288C	YMR214W
YBR288C	YNL064C
YBR288C	YNL071W
YBR288C	YPL016W
YBR288C	YPL195W
YBR289W	YDR224C

YBR289W	YER112W
YBR289W	YFL049W
YBR289W	YHL025W
YBR289W	YJL176C
YBR289W	YKR001C
YBR289W	YMR033W
YBR289W	YNL207W
YBR289W	YNR023W
YBR289W	YOR038C
YBR289W	YOR119C
YBR289W	YOR290C
YBR289W	YPL016W
YBR289W	YPR034W
YBR290W	YCL052C
YBR290W	YCR011C
YBR290W	YCR024C-A
YBR290W	YCR034W
YBR290W	YDL015C
YBR290W	YDL212W
YBR290W	YDR297W
YBR290W	YEL036C
YBR290W	YGL012W
YBR290W	YGL051W
YBR290W	YGR284C
YBR290W	YHR133C
YBR290W	YIL016W
YBR290W	YIL114C
YBR290W	YJL117W
YBR290W	YJL196C
YBR290W	YJR117W
YBR290W	YKL008C
YBR290W	YKL065C
YBR290W	YLR004C
YBR290W	YLR018C
YBR290W	YLR372W
YBR290W	YML048W
YBR290W	YMR215W
YBR290W	YOR016C
YBR290W	YPL076W
YBR290W	YPL087W
YBR290W	YPL132W
YBR290W	YPL227C
YBR290W	YPR028W
YBR290W	YPR156C
YBR291C	YHR096C

YBR291C	YIL034C
YBR291C	YLL005C
YBR293W	YGR174C
YBR293W	YGR260W
YBR293W	YJR091C
YBR293W	YJR117W
YBR293W	YLR083C
YBR295W	YHR123W
YBR295W	YIL030C
YBR295W	YIL148W
YBR296C	YHR133C
YBR296C	YHR188C
YBR296C	YLL028W
YBR296C	YLR137W
YBR296C	YML089C
YBR298C	YCR034W
YBR298C	YDL212W
YBR298C	YDR276C
YBR298C	YDR284C
YBR298C	YEL027W
YBR298C	YGL051W
YBR298C	YGR041W
YBR298C	YHL042W
YBR298C	YKL065C
YBR298C	YKL154W
YBR298C	YLR372W
YBR298C	YML048W
YBR298C	YMR264W
YBR298C	YMR279C
YBR298C	YNL008C
YBR298C	YNL121C
YBR299W	YGR292W
YBR301W	YMR047C
YBR302C	YDL212W
YBR302C	YDR311W
YBR302C	YHR026W
YBR302C	YLL028W
YBR302C	YLR237W
YBR302C	YML129C
YBR302C	YOR192C
YBR302C	YPL105C
YBR302C	YPR063C
YCL004W	YLR052W
YCL005W	YJR091C
YCL007C	YER071C

YCL008C YFR024C-A
YCL008C YHL002W
YCL008C YHR016C
YCL008C YHR114W
YCL008C YIL148W
YCL008C YJL172W
YCL008C YLR119W
YCL008C YLR191W
YCL008C YMR032W
YCL008C YPL065W
YCL009C YER094C
YCL009C YIL034C
YCL009C YLR288C
YCL009C YMR108W
YCL010C YDR176W
YCL010C YGL112C
YCL010C YGR252W
YCL010C YHR099W
YCL010C YKL023W
YCL010C YOR119C
YCL011C YDL084W
YCL011C YDL213C
YCL011C YDR120C
YCL011C YDR138W
YCL011C YDR381W
YCL011C YFL034C-B
YCL011C YHR167W
YCL011C YKL139W
YCL011C YLL019C
YCL011C YLR432W
YCL011C YML062C
YCL011C YMR216C
YCL011C YNL139C
YCL011C YNL189W
YCL011C YNL253W
YCL011C YNL298W
YCL011C YPR161C
YCL014W YCR002C
YCL014W YER082C
YCL014W YGL158W
YCL014W YGR220C
YCL014W YHR197W
YCL014W YIL104C
YCL014W YJR091C
YCL014W YJR092W

YCL014W	YPR086W
YCL014W	YPR119W
YCL016C	YGR020C
YCL016C	YHL023C
YCL016C	YHR058C
YCL016C	YHR191C
YCL016C	YLR123C
YCL017C	YFL023W
YCL017C	YLR310C
YCL017C	YPL026C
YCL017C	YPL135W
YCL018W	YCL039W
YCL018W	YCR002C
YCL018W	YCR009C
YCL018W	YCR014C
YCL018W	YCR057C
YCL018W	YCR077C
YCL018W	YCR084C
YCL018W	YCR092C
YCL018W	YDL017W
YCL018W	YDL029W
YCL018W	YDL047W
YCL018W	YDL059C
YCL018W	YDL101C
YCL018W	YDL116W
YCL018W	YDL134C
YCL018W	YDL145C
YCL018W	YDL188C
YCL018W	YDL225W
YCL018W	YDR076W
YCL018W	YDR080W
YCL018W	YDR113C
YCL018W	YDR128W
YCL018W	YDR129C
YCL018W	YDR138W
YCL018W	YDR142C
YCL018W	YDR143C
YCL018W	YDR165W
YCL018W	YDR200C
YCL018W	YDR247W
YCL018W	YDR260C
YCL018W	YDR267C
YCL018W	YDR369C
YCL018W	YDR394W
YCL018W	YDR398W

YCL018W YDR488C
YCL018W YDR499W
YCL018W YDR523C
YCL018W YEL056W
YCL018W YER012W
YCL018W YER066C-A
YCL018W YER075C
YCL018W YER133W
YCL018W YER171W
YCL018W YER173W
YCL018W YER179W
YCL018W YFR021W
YCL018W YFR040W
YCL018W YGL003C
YCL018W YGL004C
YCL018W YGL137W
YCL018W YGL158W
YCL018W YGL190C
YCL018W YGL208W
YCL018W YGR040W
YCL018W YGR052W
YCL018W YGR083C
YCL018W YGR262C
YCL018W YHR030C
YCL018W YHR070W
YCL018W YHR135C
YCL018W YHR169W
YCL018W YIL035C
YCL018W YIL066C
YCL018W YIL128W
YCL018W YIL142W
YCL018W YIL147C
YCL018W YJL005W
YCL018W YJL092W
YCL018W YJL098W
YCL018W YJL128C
YCL018W YJL157C
YCL018W YJR022W
YCL018W YKL103C
YCL018W YKL130C
YCL018W YKL166C
YCL018W YKL189W
YCL018W YKR026C
YCL018W YKR036C
YCL018W YLL010C

YCL018W YLL011W
YCL018W YLL019C
YCL018W YLR006C
YCL018W YLR097C
YCL018W YLR175W
YCL018W YLR229C
YCL018W YLR247C
YCL018W YLR248W
YCL018W YLR291C
YCL018W YLR314C
YCL018W YLR403W
YCL018W YML016C
YCL018W YML057W
YCL018W YML064C
YCL018W YML115C
YCL018W YMR022W
YCL018W YMR049C
YCL018W YMR059W
YCL018W YMR106C
YCL018W YMR116C
YCL018W YMR117C
YCL018W YMR205C
YCL018W YMR246W
YCL018W YMR284W
YCL018W YNL094W
YCL018W YNL106C
YCL018W YNL128W
YCL018W YNL135C
YCL018W YNL161W
YCL018W YNL180C
YCL018W YNL183C
YCL018W YNL230C
YCL018W YNR031C
YCL018W YOL062C
YCL018W YOL094C
YCL018W YOL115W
YCL018W YOL126C
YCL018W YOL133W
YCL018W YOL139C
YCL018W YOR061W
YCL018W YOR125C
YCL018W YOR212W
YCL018W YOR272W
YCL018W YOR341W
YCL018W YOR351C

YCL018W	YPL022W
YCL018W	YPL026C
YCL018W	YPL031C
YCL018W	YPL139C
YCL018W	YPL140C
YCL018W	YPL150W
YCL018W	YPL151C
YCL018W	YPL203W
YCL018W	YPL204W
YCL018W	YPL259C
YCL018W	YPR017C
YCL018W	YPR054W
YCL018W	YPR110C
YCL018W	YPR111W
YCL018W	YPR178W
YCL019W	YDR261W-A
YCL019W	YDR261W-B
YCL019W	YHR189W
YCL019W	YOL030W
YCL020W	YGR161W-B
YCL023C	YLR453C
YCL023C	YML008C
YCL024W	YCR002C
YCL024W	YDL225W
YCL024W	YFR028C
YCL024W	YHR107C
YCL024W	YJR076C
YCL024W	YKR048C
YCL024W	YLR314C
YCL024W	YMR139W
YCL024W	YPL093W
YCL025C	YCL027W
YCL025C	YCR034W
YCL025C	YDL212W
YCL025C	YDL232W
YCL025C	YDR276C
YCL025C	YER026C
YCL025C	YFL041W
YCL025C	YGR060W
YCL025C	YGR284C
YCL025C	YHL003C
YCL025C	YHR026W
YCL025C	YHR133C
YCL025C	YIL016W
YCL025C	YIR038C

YCL025C	YJL117W
YCL025C	YJR095W
YCL025C	YJR117W
YCL025C	YKL008C
YCL025C	YKL065C
YCL025C	YLR343W
YCL025C	YLR372W
YCL025C	YML038C
YCL025C	YML048W
YCL025C	YMR215W
YCL025C	YOR016C
YCL025C	YPL076W
YCL025C	YPL227C
YCL025C	YPL234C
YCL025C	YPL265W
YCL025C	YPR020W
YCL025C	YPR198W
YCL026C-A	YCR048W
YCL026C-A	YGL213C
YCL026C-A	YLR177W
YCL026C-A	YNL189W
YCL026C-A	YPR086W
YCL027W	YCL039W
YCL027W	YCL052C
YCL027W	YDL199C
YCL027W	YDR072C
YCL027W	YDR128W
YCL027W	YDR160W
YCL027W	YDR206W
YCL027W	YER032W
YCL027W	YER090W
YCL027W	YER118C
YCL027W	YER149C
YCL027W	YER155C
YCL027W	YFL039C
YCL027W	YGL099W
YCL027W	YGR070W
YCL027W	YGR289C
YCL027W	YHR082C
YCL027W	YIL159W
YCL027W	YIR014W
YCL027W	YJL047C
YCL027W	YJL153C
YCL027W	YJR030C
YCL027W	YKL092C

YCL027W YKL105C
YCL027W YKL202W
YCL027W YKR023W
YCL027W YKR049C
YCL027W YKR101W
YCL027W YLR056W
YCL027W YLR117C
YCL027W YLR291C
YCL027W YML058W
YCL027W YMR047C
YCL027W YMR103C
YCL027W YMR140W
YCL027W YMR156C
YCL027W YMR265C
YCL027W YMR308C
YCL027W YNL042W
YCL027W YNL054W
YCL027W YNL058C
YCL027W YNL075W
YCL027W YNL267W
YCL027W YNL271C
YCL027W YNL291C
YCL027W YNR064C
YCL027W YOL019W
YCL027W YOL046C
YCL027W YOR017W
YCL027W YOR155C
YCL027W YPL038W
YCL027W YPL070W
YCL027W YPL156C
YCL027W YPL224C
YCL027W YPL242C
YCL027W YPR008W
YCL027W YPR023C
YCL027W YPR160C-A
YCL028W YDR128W
YCL028W YDR260C
YCL028W YDR499W
YCL028W YEL021W
YCL028W YER125W
YCL028W YGL215W
YCL028W YHR114W
YCL028W YIL066C
YCL028W YLR403W
YCL028W YML058W

YCL028W YNL007C
YCL028W YNL064C
YCL028W YNL106C
YCL028W YNL189W
YCL028W YNL290W
YCL028W YOL094C
YCL028W YOR212W
YCL028W YPL140C
YCL029C YER016W
YCL029C YKL103C
YCL029C YLR045C
YCL029C YML085C
YCL029C YPL124W
YCL029C YPL155C
YCL030C YDL160C
YCL030C YGR052W
YCL030C YLR044C
YCL030C YLR180W
YCL030C YML124C
YCL031C YDR388W
YCL031C YER118C
YCL031C YFR024C-A
YCL031C YGR090W
YCL031C YHL002W
YCL031C YHR114W
YCL031C YIL035C
YCL031C YJL020C
YCL031C YLR191W
YCL031C YLR310C
YCL031C YMR109W
YCL031C YOL082W
YCL031C YPR154W
YCL032W YCR082W
YCL032W YDL016C
YCL032W YDL239C
YCL032W YDR032C
YCL032W YDR103W
YCL032W YDR308C
YCL032W YDR309C
YCL032W YDR386W
YCL032W YDR416W
YCL032W YEL051W
YCL032W YER040W
YCL032W YER047C
YCL032W YGL233W

YCL032W	YHR061C
YCL032W	YJR093C
YCL032W	YKL061W
YCL032W	YKR020W
YCL032W	YLL049W
YCL032W	YLR223C
YCL032W	YLR362W
YCL032W	YLR423C
YCL032W	YMR117C
YCL032W	YMR270C
YCL032W	YNL086W
YCL032W	YOL043C
YCL032W	YOL123W
YCL032W	YOR036W
YCL032W	YOR270C
YCL032W	YPL120W
YCL032W	YPR051W
YCL032W	YPR075C
YCL032W	YPR076W
YCL032W	YPR182W
YCL032W	YPR193C
YCL033C	YKL130C
YCL034W	YHR016C
YCL034W	YHR114W
YCL034W	YOR181W
YCL035C	YIL034C
YCL035C	YKL198C
YCL035C	YMR059W
YCL036W	YDR388W
YCL037C	YDL043C
YCL037C	YDL175C
YCL037C	YDL208W
YCL037C	YDL213C
YCL037C	YDR477W
YCL037C	YER082C
YCL037C	YER142C
YCL037C	YGL120C
YCL037C	YGR040W
YCL037C	YGR054W
YCL037C	YGR162W
YCL037C	YIL035C
YCL037C	YIR001C
YCL037C	YJL033W
YCL037C	YKL139W
YCL037C	YLR263W

YCL037C	YLR383W
YCL037C	YLR427W
YCL037C	YMR106C
YCL037C	YMR125W
YCL037C	YNL088W
YCL037C	YOL100W
YCL037C	YOL115W
YCL037C	YOR276W
YCL037C	YPL204W
YCL037C	YPR088C
YCL038C	YER081W
YCL038C	YHR178W
YCL038C	YMR009W
YCL039W	YDL185W
YCL039W	YDL225W
YCL039W	YDR255C
YCL039W	YDR342C
YCL039W	YER091C
YCL039W	YER165W
YCL039W	YGL227W
YCL039W	YGR254W
YCL039W	YIL017C
YCL039W	YIL097W
YCL039W	YJL008C
YCL039W	YJR121W
YCL039W	YLR044C
YCL039W	YLR259C
YCL039W	YMR047C
YCL039W	YMR135C
YCL039W	YOR375C
YCL039W	YPL018W
YCL039W	YPL139C
YCL040W	YCL046W
YCL040W	YCR002C
YCL040W	YDL059C
YCL040W	YDL164C
YCL040W	YDR142C
YCL040W	YDR388W
YCL040W	YDR419W
YCL040W	YDR436W
YCL040W	YDR516C
YCL040W	YER017C
YCL040W	YER133W
YCL040W	YER161C
YCL040W	YFL034C-B

YCL040W	YFL039C
YCL040W	YFR028C
YCL040W	YGL081W
YCL040W	YGL158W
YCL040W	YGL237C
YCL040W	YGR123C
YCL040W	YGR278W
YCL040W	YHR030C
YCL040W	YJL044C
YCL040W	YJL092W
YCL040W	YJL173C
YCL040W	YKL056C
YCL040W	YLR320W
YCL040W	YLR340W
YCL040W	YLR442C
YCL040W	YML064C
YCL040W	YML099C
YCL040W	YMR049C
YCL040W	YMR059W
YCL040W	YMR106C
YCL040W	YMR139W
YCL040W	YNL032W
YCL040W	YNL189W
YCL040W	YNL311C
YCL040W	YNR010W
YCL040W	YOL094C
YCL040W	YOR276W
YCL040W	YPL074W
YCL040W	YPL126W
YCL040W	YPL149W
YCL040W	YPL150W
YCL040W	YPL164C
YCL040W	YPL204W
YCL042W	YNL290W
YCL043C	YDR388W
YCL043C	YER189W
YCL043C	YGL190C
YCL043C	YIL035C
YCL043C	YIR001C
YCL043C	YJR076C
YCL043C	YLR233C
YCL043C	YLR354C
YCL043C	YMR102C
YCL043C	YMR291W
YCL043C	YNL135C

YCL043C	YOL062C
YCL043C	YOL126C
YCL044C	YDL116W
YCL045C	YJR091C
YCL046W	YGL115W
YCL046W	YJR093C
YCL046W	YPL208W
YCL048W	YDL017W
YCL048W	YJL034W
YCL049C	YKL002W
YCL050C	YJR022W
YCL050C	YJR091C
YCL051W	YDL116W
YCL051W	YDR099W
YCL051W	YPR086W
YCL052C	YEL060C
YCL052C	YEL063C
YCL052C	YGR260W
YCL052C	YGR281W
YCL052C	YHR026W
YCL052C	YHR140W
YCL052C	YHR142W
YCL052C	YJL108C
YCL052C	YJR010C-A
YCL052C	YJR106W
YCL052C	YLR268W
YCL054W	YDL014W
YCL054W	YER133W
YCL054W	YGR103W
YCL054W	YKR081C
YCL054W	YNL110C
YCL054W	YNL154C
YCL054W	YOR080W
YCL054W	YOR267C
YCL054W	YOR310C
YCL055W	YGL036W
YCL055W	YGL192W
YCL055W	YLR191W
YCL055W	YMR147W
YCL055W	YNL196C
YCL056C	YDL157C
YCL056C	YIL034C
YCL056C	YNL100W
YCL056C	YOR037W
YCL058C	YPL215W

YCL059C	YCR057C
YCL059C	YDL014W
YCL059C	YDL148C
YCL059C	YDL166C
YCL059C	YDL213C
YCL059C	YDR299W
YCL059C	YDR324C
YCL059C	YDR449C
YCL059C	YDR457W
YCL059C	YER082C
YCL059C	YER127W
YCL059C	YGL201C
YCL059C	YGR145W
YCL059C	YHR148W
YCL059C	YJL109C
YCL059C	YJR002W
YCL059C	YKR060W
YCL059C	YLL011W
YCL059C	YLR175W
YCL059C	YLR186W
YCL059C	YLR197W
YCL059C	YLR409C
YCL059C	YMR093W
YCL059C	YMR116C
YCL059C	YMR128W
YCL059C	YNL132W
YCL059C	YNL308C
YCL059C	YPL126W
YCL059C	YPL217C
YCL059C	YPL259C
YCL059C	YPR137W
YCL059C	YPR144C
YCL061C	YDL030W
YCL061C	YLR103C
YCL061C	YNL273W
YCL061C	YOR080W
YCL061C	YPR019W
YCL063W	YER150W
YCL063W	YFR021W
YCL063W	YJL043W
YCL063W	YJL173C
YCL063W	YJR091C
YCL063W	YLR373C
YCL063W	YLR423C
YCL063W	YPL005W

YCL064C	YDL029W
YCL064C	YDL134C
YCL064C	YDR388W
YCL064C	YDR394W
YCL064C	YDR499W
YCL064C	YDR523C
YCL064C	YER171W
YCL064C	YER173W
YCL064C	YGL158W
YCL064C	YHR030C
YCL064C	YJR022W
YCL064C	YKL166C
YCL064C	YLR097C
YCL064C	YML057W
YCL064C	YMR106C
YCL064C	YNL056W
YCL064C	YNL312W
YCL064C	YOL133W
YCL064C	YPL140C
YCL064C	YPR054W
YCL064C	YPR093C
YCL065W	YLR374C
YCL069W	YCR075C
YCL069W	YDL116W
YCL069W	YIL004C
YCL076W	YOR209C
YCR001W	YEL037C
YCR001W	YFL037W
YCR001W	YFR053C
YCR001W	YGL206C
YCR001W	YJL066C
YCR001W	YJR077C
YCR001W	YML085C
YCR001W	YML124C
YCR001W	YOR241W
YCR002C	YDL055C
YCR002C	YDL225W
YCR002C	YDL229W
YCR002C	YDR099W
YCR002C	YDR324C
YCR002C	YDR507C
YCR002C	YER043C
YCR002C	YFL018C
YCR002C	YFR030W
YCR002C	YFR053C

YCR002C	YGL105W
YCR002C	YGL158W
YCR002C	YGL234W
YCR002C	YGL253W
YCR002C	YGR155W
YCR002C	YHR107C
YCR002C	YJL008C
YCR002C	YJL138C
YCR002C	YJR045C
YCR002C	YJR076C
YCR002C	YJR092W
YCR002C	YJR121W
YCR002C	YKL060C
YCR002C	YKL104C
YCR002C	YLR044C
YCR002C	YLR180W
YCR002C	YLR259C
YCR002C	YLR314C
YCR002C	YMR108W
YCR002C	YMR186W
YCR002C	YNL166C
YCR002C	YNL312W
YCR002C	YOR136W
YCR003W	YIR039C
YCR004C	YDL101C
YCR004C	YDR032C
YCR004C	YLR253W
YCR005C	YJL042W
YCR005C	YKL085W
YCR005C	YKL152C
YCR005C	YPL245W
YCR007C	YCR061W
YCR007C	YDR107C
YCR007C	YLR288C
YCR007C	YPL020C
YCR008W	YJL066C
YCR008W	YJL088W
YCR008W	YMR047C
YCR008W	YPL031C
YCR009C	YDL185W
YCR009C	YDR171W
YCR009C	YDR277C
YCR009C	YDR388W
YCR009C	YDR395W
YCR009C	YER043C

YCR009C	YER110C
YCR009C	YGL060W
YCR009C	YGL137W
YCR009C	YGR254W
YCR009C	YJL052W
YCR009C	YJL066C
YCR009C	YJR009C
YCR009C	YJR045C
YCR009C	YJR115W
YCR009C	YKL104C
YCR009C	YLR044C
YCR009C	YLR304C
YCR009C	YLR433C
YCR009C	YMR108W
YCR009C	YMR192W
YCR009C	YMR232W
YCR009C	YNL055C
YCR009C	YNL094W
YCR009C	YOL055C
YCR009C	YOR181W
YCR009C	YOR232W
YCR009C	YOR375C
YCR009C	YPL140C
YCR009C	YPL249C-A
YCR010C	YCR061W
YCR010C	YEL063C
YCR010C	YFL055W
YCR010C	YJR040W
YCR011C	YGL098W
YCR011C	YNL154C
YCR011C	YPR079W
YCR012W	YDL097C
YCR012W	YDL145C
YCR012W	YEL031W
YCR012W	YGL137W
YCR012W	YHR041C
YCR012W	YNL006W
YCR012W	YOL135C
YCR012W	YOR110W
YCR012W	YOR128C
YCR014C	YFL037W
YCR014C	YJL008C
YCR014C	YJL034W
YCR014C	YJL110C
YCR014C	YML124C

YCR014C	YNL064C
YCR014C	YNL180C
YCR014C	YOL085C
YCR014C	YOR005C
YCR014C	YPL240C
YCR015C	YGR163W
YCR016W	YDL231C
YCR016W	YLR288C
YCR020C-A	YEL053C
YCR020C-A	YJR022W
YCR020C-A	YPR051W
YCR021C	YDL153C
YCR021C	YJL068C
YCR021C	YKL220C
YCR021C	YLL043W
YCR021C	YLR453C
YCR021C	YPL189W
YCR021C	YPR124W
YCR022C	YDL017W
YCR022C	YHR114W
YCR023C	YMR075C-A
YCR023C	YMR075W
YCR024C	YER146W
YCR024C-A	YDL212W
YCR024C-A	YDR331W
YCR024C-A	YDR414C
YCR024C-A	YEL063C
YCR024C-A	YGL008C
YCR024C-A	YGR284C
YCR024C-A	YJL004C
YCR024C-A	YJL124C
YCR024C-A	YLL023C
YCR024C-A	YLR241W
YCR024C-A	YLR372W
YCR024C-A	YOL132W
YCR024C-A	YPL227C
YCR027C	YER179W
YCR027C	YKL152C
YCR027C	YOL083W
YCR028C	YGL051W
YCR028C	YJL004C
YCR028C	YLR372W
YCR028C	YNR065C
YCR028C	YOR254C
YCR028C-A	YDR097C

YCR028C-A	YJR144W
YCR028C-A	YMR234W
YCR028C-A	YNL298W
YCR028C-A	YPR161C
YCR030C	YDR158W
YCR030C	YFR024C-A
YCR030C	YGL127C
YCR030C	YGR220C
YCR030C	YHR016C
YCR030C	YJL098W
YCR030C	YJR126C
YCR030C	YMR132C
YCR030C	YNR047W
YCR030C	YOL016C
YCR030C	YOL054W
YCR033W	YDR155C
YCR033W	YGL194C
YCR033W	YGR172C
YCR033W	YIL112W
YCR033W	YKR029C
YCR033W	YMR273C
YCR033W	YOL068C
YCR034W	YDL015C
YCR034W	YDL054C
YCR034W	YDR276C
YCR034W	YDR297W
YCR034W	YDR307W
YCR034W	YDR331W
YCR034W	YDR414C
YCR034W	YDR456W
YCR034W	YEL002C
YCR034W	YEL017C-A
YCR034W	YEL063C
YCR034W	YFL025C
YCR034W	YFL048C
YCR034W	YFL062W
YCR034W	YGL055W
YCR034W	YGL200C
YCR034W	YGR060W
YCR034W	YGR191W
YCR034W	YGR260W
YCR034W	YGR289C
YCR034W	YGR295C
YCR034W	YHL042W
YCR034W	YHL048W

YCR034W YHR007C
YCR034W YHR094C
YCR034W YHR110W
YCR034W YHR123W
YCR034W YHR133C
YCR034W YHR140W
YCR034W YHR142W
YCR034W YIR022W
YCR034W YJL117W
YCR034W YJL196C
YCR034W YJL219W
YCR034W YJL222W
YCR034W YJR010C-A
YCR034W YJR161C
YCR034W YKL212W
YCR034W YLL006W
YCR034W YLL028W
YCR034W YLL061W
YCR034W YLR018C
YCR034W YML067C
YCR034W YMR058W
YCR034W YMR149W
YCR034W YMR215W
YCR034W YNL048W
YCR034W YNL101W
YCR034W YOL020W
YCR034W YOL030W
YCR034W YOL132W
YCR034W YOR049C
YCR034W YPL076W
YCR034W YPL264C
YCR034W YPL274W
YCR034W YPR156C
YCR035C YDR280W
YCR035C YGL127C
YCR035C YGR095C
YCR035C YGR195W
YCR035C YHR069C
YCR035C YHR114W
YCR035C YNL189W
YCR035C YNL232W
YCR035C YOL021C
YCR036W YGL153W
YCR037C YHL042W
YCR038C YDL065C

YCR038C YDR532C
YCR038C YEL061C
YCR038C YHR119W
YCR039C YDL116W
YCR040W YCR096C
YCR040W YGR284C
YCR040W YHR084W
YCR040W YJR091C
YCR040W YLR288C
YCR040W YLR378C
YCR040W YMR043W
YCR041W YCR065W
YCR041W YDR176W
YCR041W YIL118W
YCR041W YJL031C
YCR041W YJR091C
YCR042C YER148W
YCR042C YGL112C
YCR042C YGR274C
YCR042C YKL081W
YCR042C YML015C
YCR042C YML114C
YCR042C YPL129W
YCR044C YDR331W
YCR044C YHR114W
YCR044C YHR140W
YCR045C YOR348C
YCR046C YDL049C
YCR046C YGR091W
YCR046C YGR220C
YCR046C YKL139W
YCR046C YNL284C
YCR047C YLR339C
YCR047C YNL282W
YCR048W YHL002W
YCR048W YLR427W
YCR050C YDL017W
YCR052W YFR037C
YCR052W YIL084C
YCR052W YNL202W
YCR052W YOR232W
YCR053W YJR007W
YCR053W YLR314C
YCR053W YNL244C
YCR054C YDL097C

YCR054C	YMR032W
YCR057C	YDL014W
YCR057C	YDL148C
YCR057C	YDL213C
YCR057C	YDR034C
YCR057C	YDR299W
YCR057C	YDR324C
YCR057C	YDR365C
YCR057C	YDR382W
YCR057C	YDR449C
YCR057C	YDR502C
YCR057C	YEL050C
YCR057C	YER082C
YCR057C	YGL011C
YCR057C	YGL120C
YCR057C	YGL171W
YCR057C	YGR090W
YCR057C	YGR128C
YCR057C	YGR135W
YCR057C	YGR145W
YCR057C	YGR154C
YCR057C	YGR210C
YCR057C	YHR148W
YCR057C	YHR169W
YCR057C	YHR196W
YCR057C	YJL033W
YCR057C	YJL069C
YCR057C	YJL088W
YCR057C	YJL109C
YCR057C	YJR002W
YCR057C	YJR121W
YCR057C	YKL099C
YCR057C	YKR060W
YCR057C	YLL011W
YCR057C	YLR129W
YCR057C	YLR175W
YCR057C	YLR186W
YCR057C	YLR197W
YCR057C	YLR222C
YCR057C	YLR409C
YCR057C	YML085C
YCR057C	YML130C
YCR057C	YMR066W
YCR057C	YMR093W
YCR057C	YMR128W

YCR057C	YMR300C
YCR057C	YNL030W
YCR057C	YNL064C
YCR057C	YNL075W
YCR057C	YNL132W
YCR057C	YNR043W
YCR057C	YNR054C
YCR057C	YOL038W
YCR057C	YOR078W
YCR057C	YOR310C
YCR057C	YPL094C
YCR057C	YPL126W
YCR057C	YPL217C
YCR057C	YPR137W
YCR057C	YPR144C
YCR059C	YDR400W
YCR059C	YJL160C
YCR059C	YJR091C
YCR060W	YHR034C
YCR061W	YDL035C
YCR061W	YEL063C
YCR061W	YER001W
YCR061W	YGR055W
YCR061W	YHL048W
YCR061W	YHR103W
YCR061W	YIL134W
YCR061W	YIL147C
YCR061W	YJR117W
YCR061W	YKL004W
YCR061W	YLL048C
YCR061W	YLR138W
YCR061W	YMR243C
YCR061W	YNL318C
YCR061W	YNR066C
YCR061W	YOR266W
YCR061W	YPR058W
YCR063W	YDR408C
YCR063W	YHR165C
YCR063W	YMR213W
YCR066W	YDR092W
YCR066W	YGL058W
YCR066W	YLR032W
YCR066W	YLR438C-A
YCR066W	YOR128C
YCR067C	YNL171C

YCR067C	YOR128C
YCR068W	YDR331W
YCR068W	YER019W
YCR068W	YFL062W
YCR071C	YGR220C
YCR071C	YNL284C
YCR072C	YER081W
YCR072C	YER126C
YCR072C	YGL181W
YCR072C	YGR245C
YCR072C	YHR197W
YCR072C	YLR074C
YCR072C	YLR106C
YCR072C	YNL182C
YCR073C	YDR170C
YCR073C	YHR027C
YCR073C	YIL051C
YCR073C	YJL128C
YCR073C	YLR006C
YCR073C	YNL250W
YCR073C	YNR031C
YCR075C	YDR456W
YCR075C	YFL062W
YCR076C	YDL138W
YCR076C	YGL137W
YCR076C	YLR291C
YCR076C	YML064C
YCR076C	YNL118C
YCR076C	YNL189W
YCR077C	YDL065C
YCR077C	YDL139C
YCR077C	YDL160C
YCR077C	YDL175C
YCR077C	YDL216C
YCR077C	YDR141C
YCR077C	YDR378C
YCR077C	YDR389W
YCR077C	YER112W
YCR077C	YER115C
YCR077C	YER146W
YCR077C	YGL121C
YCR077C	YGL143C
YCR077C	YGL173C
YCR077C	YGR218W
YCR077C	YHL034C

YCR077C YHR114W
YCR077C YIL154C
YCR077C YJL124C
YCR077C YJR022W
YCR077C YJR045C
YCR077C YKL152C
YCR077C YLR259C
YCR077C YLR438C-A
YCR077C YML109W
YCR077C YMR002W
YCR077C YMR288W
YCR077C YNL088W
YCR077C YNL118C
YCR077C YNL147W
YCR077C YNR027W
YCR077C YNR053C
YCR079W YDR148C
YCR079W YER178W
YCR079W YFL018C
YCR079W YFL037W
YCR079W YFR053C
YCR079W YGL062W
YCR079W YGR193C
YCR079W YIL125W
YCR079W YJL138C
YCR079W YJR077C
YCR079W YKL054C
YCR079W YKL085W
YCR079W YLR192C
YCR079W YLR259C
YCR079W YMR277W
YCR079W YNL014W
YCR079W YNL071W
YCR079W YNL244C
YCR079W YOL055C
YCR079W YOR317W
YCR079W YOR323C
YCR079W YPL022W
YCR079W YPL110C
YCR079W YPL118W
YCR079W YPL160W
YCR081W YDL005C
YCR081W YJL203W
YCR081W YNL025C
YCR081W YOL051W

YCR081WYOL135C
YCR081WYOR119C
YCR082WYER022W
YCR082WYLR423C
YCR082WYOR023C
YCR084C YCR088W
YCR084C YCR096C
YCR084C YDL105W
YCR084C YDL229W
YCR084C YEL030W
YCR084C YGR083C
YCR084C YGR254W
YCR084C YJL156C
YCR084C YJR045C
YCR084C YLR313C
YCR084C YMR012W
YCR084C YNL021W
YCR084C YNL030W
YCR084C YNL167C
YCR084C YOL004W
YCR084C YPL220W
YCR084C YPL258C
YCR086WYDL089W
YCR086WYDL214C
YCR086WYDR061W
YCR086WYDR247W
YCR086WYDR309C
YCR086WYDR439W
YCR086WYER106W
YCR086WYFL008W
YCR086WYFR028C
YCR086WYGL175C
YCR086WYGR155W
YCR086WYHR061C
YCR086WYHR152W
YCR086WYJL095W
YCR086WYKL077W
YCR086WYKR010C
YCR086WYLR291C
YCR086WYML095C
YCR086WYML109W
YCR086WYOL020W
YCR086WYOR061W
YCR086WYOR098C
YCR086WYOR264W

YCR086WYOR281C	
YCR086WYPL093W	
YCR087C-A	YDR510W
YCR087C-A	YJR091C
YCR087C-A	YKR092C
YCR087C-A	YLR265C
YCR087C-A	YML064C
YCR087WYDR146C	
YCR087WYDR365C	
YCR087WYDR388W	
YCR087WYKL130C	
YCR087WYML064C	
YCR088WYCR099C	
YCR088WYDL029W	
YCR088WYDR129C	
YCR088WYDR277C	
YCR088WYDR303C	
YCR088WYDR342C	
YCR088WYDR388W	
YCR088WYDR425W	
YCR088WYDR444W	
YCR088WYDR523C	
YCR088WYFL018C	
YCR088WYFL039C	
YCR088WYFR024C-A	
YCR088WYGL206C	
YCR088WYGR136W	
YCR088WYHR016C	
YCR088WYHR199C	
YCR088WYIL095W	
YCR088WYIR003W	
YCR088WYJL020C	
YCR088WYJR045C	
YCR088WYJR065C	
YCR088WYJR121W	
YCR088WYKL182W	
YCR088WYLL031C	
YCR088WYMR109W	
YCR088WYMR156C	
YCR088WYNL020C	
YCR088WYNL071W	
YCR088WYNL094W	
YCR088WYNL106C	
YCR088WYNL138W	
YCR088WYNL243W	

YCR088WYNL298W
YCR088WYNR064C
YCR088WYOL089C
YCR088WYOR047C
YCR088WYOR116C
YCR088WYOR284W
YCR088WYOR290C
YCR088WYOR367W
YCR088WYPL008W
YCR088WYPL019C
YCR088WYPL115C
YCR088WYPL204W
YCR088WYPR154W
YCR088WYPR171W
YCR091WYMR205C
YCR091WYNR047W
YCR092C YDL126C
YCR092C YDR097C
YCR092C YDR224C
YCR092C YER091C
YCR092C YGR240C
YCR092C YKL060C
YCR092C YLL026W
YCR092C YLR355C
YCR092C YMR186W
YCR092C YNR058W
YCR092C YOL090W
YCR093WYDL145C
YCR093WYDL165W
YCR093WYDR167W
YCR093WYDR376W
YCR093WYDR443C
YCR093WYER068W
YCR093WYGL112C
YCR093WYGL137W
YCR093WYGR134W
YCR093WYGR274C
YCR093WYIL038C
YCR093WYJL141C
YCR093WYKR036C
YCR093WYML015C
YCR093WYMR005W
YCR093WYNL025C
YCR093WYNL288W
YCR093WYNR052C

YCR093W	YOL133W
YCR093W	YOR110W
YCR093W	YPL011C
YCR093W	YPL042C
YCR093W	YPL235W
YCR093W	YPR072W
YCR094W	YFL037W
YCR094W	YGL090W
YCR094W	YJR091C
YCR094W	YNL064C
YCR095C	YDR034C
YCR095C	YHL029C
YCR095C	YNL099C
YCR095C	YPL027W
YCR096C	YCR097W
YCR096C	YLR288C
YCR096C	YMR043W
YCR096C	YOL006C
YCR098C	YHR110W
YCR098C	YJL184W
YCR098C	YLR447C
YCR098C	YNL008C
YCR098C	YPL265W
YCR098C	YPL274W
YCR099C	YGR203W
YCR099C	YGR205W
YCR099C	YGR262C
YCR099C	YHR148W
YCR099C	YIL059C
YCR099C	YLR208W
YCR100C	YDR414C
YCR100C	YIL110W
YCR101C	YGR216C
YCR101C	YHL042W
YCR101C	YIL061C
YCR105W	YOL061W
YCR106W	YJL096W
YCR106W	YNL171C
YCR107W	YJR022W
YCR107W	YOR114W
YDL001W	YDL133W
YDL001W	YDR207C
YDL001W	YIR009W
YDL001W	YLR457C
YDL001W	YMR079W

YDL002C	YDL076C
YDL002C	YER092W
YDL002C	YFL013C
YDL002C	YFL039C
YDL002C	YFR037C
YDL002C	YGL133W
YDL002C	YGL150C
YDL002C	YJL081C
YDL002C	YKR001C
YDL002C	YLR052W
YDL002C	YLR191W
YDL002C	YLR357W
YDL002C	YOR141C
YDL002C	YOR304W
YDL002C	YPL235W
YDL003W	YDR180W
YDL003W	YER147C
YDL003W	YFL008W
YDL003W	YFR027W
YDL003W	YIL026C
YDL003W	YJL074C
YDL003W	YMR001C
YDL004W	YJR121W
YDL005C	YDL153C
YDL005C	YDR443C
YDL005C	YER022W
YDL005C	YER157W
YDL005C	YGL025C
YDL005C	YGL151W
YDL005C	YGR104C
YDL005C	YHR041C
YDL005C	YHR058C
YDL005C	YLR071C
YDL005C	YML007W
YDL005C	YMR112C
YDL005C	YNL189W
YDL005C	YNL236W
YDL005C	YNR010W
YDL005C	YOL051W
YDL005C	YOL135C
YDL005C	YOR174W
YDL005C	YPL042C
YDL005C	YPR070W
YDL005C	YPR168W
YDL006W	YDL132W

YDL006W	YDR162C
YDL006W	YDR167W
YDL006W	YJL095W
YDL006W	YJL128C
YDL006W	YJR121W
YDL006W	YLR148W
YDL006W	YML100W
YDL006W	YNR019W
YDL006W	YOL113W
YDL007W	YDL017W
YDL007W	YDL097C
YDL007W	YDL147W
YDL007W	YDR394W
YDL007W	YEL037C
YDL007W	YER012W
YDL007W	YFR004W
YDL007W	YFR010W
YDL007W	YFR052W
YDL007W	YGL004C
YDL007W	YGR232W
YDL007W	YHR027C
YDL007W	YHR200W
YDL007W	YKL145W
YDL007W	YNL311C
YDL007W	YPR108W
YDL008W	YDR118W
YDL008W	YFR036W
YDL008W	YGL240W
YDL008W	YHR166C
YDL008W	YKL022C
YDL008W	YLR102C
YDL008W	YLR127C
YDL008W	YMR001C
YDL008W	YNL172W
YDL008W	YOR249C
YDL011C	YEL023C
YDL011C	YOR046C
YDL012C	YDR151C
YDL012C	YFR024C-A
YDL012C	YFR047C
YDL012C	YHR032W
YDL012C	YHR140W
YDL012C	YIL172C
YDL012C	YJL065C
YDL012C	YOR355W

YDL013W YDL030W
YDL013W YDR510W
YDL013W YER116C
YDL013W YFL039C
YDL013W YGL173C
YDL013W YGL206C
YDL013W YIL061C
YDL013W YJL124C
YDL013W YJL130C
YDL013W YJR091C
YDL013W YJR121W
YDL013W YKL182W
YDL013W YLR006C
YDL013W YLR438C-A
YDL013W YLR443W
YDL013W YML085C
YDL013W YNR031C
YDL013W YOR208W
YDL013W YPL240C
YDL014W YDL208W
YDL014W YDR060W
YDL014W YER082C
YDL014W YER161C
YDL014W YGL120C
YDL014W YGL130W
YDL014W YGR090W
YDL014W YGR103W
YDL014W YGR159C
YDL014W YGR274C
YDL014W YHR072W-A
YDL014W YHR089C
YDL014W YIL131C
YDL014W YJL033W
YDL014W YJL069C
YDL014W YKL078W
YDL014W YKL130C
YDL014W YLL011W
YDL014W YLR175W
YDL014W YLR197W
YDL014W YNL061W
YDL014W YNL230C
YDL014W YOL010W
YDL014W YOL102C
YDL014W YOR267C
YDL014W YOR310C

YDL014W	YPL043W
YDL014W	YPR016C
YDL015C	YDL206W
YDL015C	YDL232W
YDL015C	YDR307W
YDL015C	YDR331W
YDL015C	YDR506C
YDL015C	YGL200C
YDL015C	YGR060W
YDL015C	YGR284C
YDL015C	YHL048W
YDL015C	YHR026W
YDL015C	YHR114W
YDL015C	YHR133C
YDL015C	YHR140W
YDL015C	YJL002C
YDL015C	YJL117W
YDL015C	YJL196C
YDL015C	YJR117W
YDL015C	YKL008C
YDL015C	YKL065C
YDL015C	YKL154W
YDL015C	YLR343W
YDL015C	YLR372W
YDL015C	YLR411W
YDL015C	YML048W
YDL015C	YMR215W
YDL015C	YNL101W
YDL015C	YOR016C
YDL015C	YPL087W
YDL015C	YPL264C
YDL015C	YPR028W
YDL017W	YDL080C
YDL017W	YDL126C
YDL017W	YDL132W
YDL017W	YDL160C
YDL017W	YDL192W
YDL017W	YDR032C
YDL017W	YDR052C
YDL017W	YDR171W
YDL017W	YDR173C
YDL017W	YDR299W
YDL017W	YDR394W
YDL017W	YDR439W
YDL017W	YDR522C

YDL017W YEL023C
YDL017W YEL030W
YDL017W YFL037W
YDL017W YFL039C
YDL017W YFR057W
YDL017W YGL048C
YDL017W YGL137W
YDL017W YGL151W
YDL017W YGR059W
YDL017W YGR087C
YDL017W YGR099W
YDL017W YHR027C
YDL017W YHR184W
YDL017W YHR185C
YDL017W YIL041W
YDL017W YIL075C
YDL017W YIL148W
YDL017W YJL034W
YDL017W YJL088W
YDL017W YJL138C
YDL017W YJR045C
YDL017W YJR089W
YDL017W YJR121W
YDL017W YKL039W
YDL017W YKL104C
YDL017W YKL145W
YDL017W YKL189W
YDL017W YLR134W
YDL017W YLR231C
YDL017W YLR259C
YDL017W YLR331C
YDL017W YLR386W
YDL017W YLR421C
YDL017W YML057W
YDL017W YML085C
YDL017W YML124C
YDL017W YMR001C
YDL017W YMR205C
YDL017W YMR214W
YDL017W YNL064C
YDL017W YNL071W
YDL017W YNL113W
YDL017W YNR048W
YDL017W YOL055C
YDL017W YOL091W

YDL017W	YOR006C
YDL017W	YOR027W
YDL017W	YOR117W
YDL017W	YOR214C
YDL017W	YOR259C
YDL017W	YOR313C
YDL017W	YPL218W
YDL017W	YPL235W
YDL017W	YPL240C
YDL017W	YPL258C
YDL019C	YER120W
YDL019C	YHR114W
YDL019C	YKL129C
YDL019C	YMR109W
YDL020C	YIL007C
YDL022W	YPL022W
YDL023C	YJL159W
YDL024C	YEL017W
YDL025C	YER155C
YDL025C	YFR028C
YDL025C	YGL105W
YDL025C	YGL245W
YDL025C	YGR016W
YDL025C	YGR090W
YDL025C	YGR103W
YDL025C	YHR009C
YDL025C	YJL076W
YDL025C	YJR045C
YDL025C	YKL152C
YDL025C	YLR002C
YDL025C	YMR049C
YDL025C	YMR290C
YDL025C	YNL175C
YDL025C	YOL041C
YDL025C	YOL139C
YDL025C	YOR061W
YDL025C	YOR272W
YDL025C	YOR294W
YDL025C	YPL093W
YDL026W	YDR318W
YDL027C	YFR024C-A
YDL028C	YDR162C
YDL028C	YDR211W
YDL028C	YDR356W
YDL028C	YDR383C

YDL028C	YER018C
YDL028C	YFR046C
YDL028C	YGL093W
YDL028C	YGL106W
YDL028C	YGR179C
YDL028C	YIL106W
YDL028C	YIL144W
YDL028C	YIL151C
YDL028C	YIR010W
YDL028C	YJR053W
YDL028C	YJR112W
YDL028C	YKL042W
YDL028C	YKL089W
YDL028C	YKR096W
YDL028C	YLL003W
YDL028C	YLR381W
YDL028C	YML091C
YDL028C	YMR109W
YDL028C	YMR117C
YDL028C	YOL069W
YDL028C	YOR257W
YDL028C	YOR373W
YDL028C	YPL124W
YDL028C	YPL204W
YDL028C	YPL233W
YDL029W	YDL047W
YDL029W	YDL055C
YDL029W	YDL126C
YDL029W	YDL225W
YDL029W	YDL229W
YDL029W	YDR032C
YDL029W	YDR127W
YDL029W	YDR143C
YDL029W	YDR171W
YDL029W	YDR190C
YDL029W	YDR216W
YDL029W	YDR260C
YDL029W	YDR388W
YDL029W	YDR394W
YDL029W	YDR523C
YDL029W	YFL016C
YDL029W	YFL037W
YDL029W	YFR030W
YDL029W	YFR053C
YDL029W	YGL008C

YDL029W YGL016W
YDL029W YGL062W
YDL029W YGR240C
YDL029W YGR254W
YDL029W YGR282C
YDL029W YHR020W
YDL029W YHR030C
YDL029W YIL062C
YDL029W YIL066C
YDL029W YIL128W
YDL029W YJL008C
YDL029W YJL034W
YDL029W YJL066C
YDL029W YJL088W
YDL029W YJR029W
YDL029W YJR045C
YDL029W YJR064W
YDL029W YJR065C
YDL029W YJR077C
YDL029W YJR109C
YDL029W YJR121W
YDL029W YKL013C
YDL029W YKL081W
YDL029W YKL104C
YDL029W YKL129C
YDL029W YKL152C
YDL029W YKL211C
YDL029W YLL013C
YDL029W YLL026W
YDL029W YLR044C
YDL029W YLR259C
YDL029W YLR309C
YDL029W YLR370C
YDL029W YLR429W
YDL029W YML085C
YDL029W YML124C
YDL029W YMR058W
YDL029W YMR108W
YDL029W YMR109W
YDL029W YMR205C
YDL029W YNL064C
YDL029W YNL084C
YDL029W YNL161W
YDL029W YNL189W
YDL029W YNL271C

YDL029W YNR035C
YDL029W YOR136W
YDL029W YOR181W
YDL029W YOR212W
YDL029W YOR259C
YDL029W YOR261C
YDL029W YPL140C
YDL029W YPR019W
YDL029W YPR178W
YDL030W YDL043C
YDL030W YDL044C
YDL030W YDL239C
YDL030W YDR026C
YDL030W YDR421W
YDL030W YDR485C
YDL030W YFL017W-A
YDL030W YGR074W
YDL030W YHR165C
YDL030W YJL203W
YDL030W YML049C
YDL030W YML104C
YDL030W YMR005W
YDL030W YMR240C
YDL030W YNR053C
YDL030W YOR017W
YDL030W YOR023C
YDL030W YOR159C
YDL030W YOR191W
YDL030W YPL146C
YDL030W YPR182W
YDL031W YGR103W
YDL031W YHR066W
YDL031W YIL035C
YDL031W YKR081C
YDL031W YNL061W
YDL031W YNL110C
YDL031W YNL207W
YDL031W YOR014W
YDL031W YOR080W
YDL031W YPR016C
YDL035C YER020W
YDL036C YER087W
YDL036C YJL020C
YDL037C YHR114W
YDL040C YHR013C

YDL040C	YML058W
YDL040C	YMR116C
YDL041W	YDR318W
YDL041W	YIR002C
YDL042C	YDR216W
YDL042C	YDR227W
YDL042C	YDR247W
YDL042C	YDR363W
YDL042C	YER129W
YDL042C	YFR028C
YDL042C	YGR052W
YDL042C	YGR192C
YDL042C	YGR282C
YDL042C	YIL035C
YDL042C	YJL076W
YDL042C	YJR057W
YDL042C	YKL023W
YDL042C	YKR010C
YDL042C	YLL001W
YDL042C	YLR249W
YDL042C	YLR453C
YDL042C	YML109W
YDL042C	YMR049C
YDL042C	YMR307W
YDL042C	YNR023W
YDL042C	YOL017W
YDL042C	YOL086C
YDL042C	YOR061W
YDL043C	YDL126C
YDL043C	YDL145C
YDL043C	YDL185W
YDL043C	YDR131C
YDL043C	YDR148C
YDL043C	YDR180W
YDL043C	YDR226W
YDL043C	YDR386W
YDL043C	YDR409W
YDL043C	YER043C
YDL043C	YER091C
YDL043C	YER112W
YDL043C	YER113C
YDL043C	YFL017W-A
YDL043C	YGL049C
YDL043C	YGL137W
YDL043C	YGL173C

YDL043C	YGL206C
YDL043C	YGR162W
YDL043C	YGR250C
YDL043C	YGR254W
YDL043C	YHR086W
YDL043C	YIL075C
YDL043C	YJL146W
YDL043C	YJL203W
YDL043C	YJR009C
YDL043C	YJR121W
YDL043C	YJR139C
YDL043C	YKL060C
YDL043C	YKL074C
YDL043C	YKL152C
YDL043C	YKL155C
YDL043C	YLL014W
YDL043C	YLR044C
YDL043C	YLR058C
YDL043C	YLR059C
YDL043C	YLR222C
YDL043C	YLR259C
YDL043C	YLR354C
YDL043C	YML028W
YDL043C	YMR012W
YDL043C	YMR117C
YDL043C	YMR186W
YDL043C	YMR205C
YDL043C	YMR240C
YDL043C	YMR302C
YDL043C	YMR304W
YDL043C	YNL023C
YDL043C	YNL286W
YDL043C	YNR031C
YDL043C	YNR053C
YDL043C	YOL055C
YDL043C	YOR374W
YDL043C	YPL126W
YDL043C	YPL215W
YDL043C	YPL258C
YDL043C	YPR121W
YDL043C	YPR160W
YDL044C	YDL116W
YDL044C	YDR505C
YDL044C	YER086W
YDL044C	YER144C

YDL044C	YGR056W
YDL044C	YLL051C
YDL044C	YLR386W
YDL045C	YGL127C
YDL045C	YLR295C
YDL046W	YDR318W
YDL046W	YJR091C
YDL047W	YDL126C
YDL047W	YDL143W
YDL047W	YDL171C
YDL047W	YDL185W
YDL047W	YDL219W
YDL047W	YDL229W
YDL047W	YDR171W
YDL047W	YDR188W
YDL047W	YDR190C
YDL047W	YDR212W
YDL047W	YDR394W
YDL047W	YDR465C
YDL047W	YER043C
YDL047W	YER155C
YDL047W	YER177W
YDL047W	YFL037W
YDL047W	YFR019W
YDL047W	YFR021W
YDL047W	YFR040W
YDL047W	YFR053C
YDL047W	YGL062W
YDL047W	YGL197W
YDL047W	YGL246C
YDL047W	YGR161C
YDL047W	YGR184C
YDL047W	YGR240C
YDL047W	YGR254W
YDL047W	YGR282C
YDL047W	YHR096C
YDL047W	YIL061C
YDL047W	YIL094C
YDL047W	YIL142W
YDL047W	YJL008C
YDL047W	YJL014W
YDL047W	YJL026W
YDL047W	YJL034W
YDL047W	YJL098W
YDL047W	YJL111W

YDL047W	YJR045C
YDL047W	YJR072C
YDL047W	YJR105W
YDL047W	YJR109C
YDL047W	YKL029C
YDL047W	YKL060C
YDL047W	YKL080W
YDL047W	YKL139W
YDL047W	YKL195W
YDL047W	YKR028W
YDL047W	YLR044C
YDL047W	YLR180W
YDL047W	YLR259C
YDL047W	YLR310C
YDL047W	YLR355C
YDL047W	YML048W
YDL047W	YML124C
YDL047W	YMR024W
YDL047W	YMR028W
YDL047W	YMR108W
YDL047W	YMR145C
YDL047W	YMR196W
YDL047W	YMR205C
YDL047W	YMR246W
YDL047W	YNL025C
YDL047W	YNL037C
YDL047W	YNL064C
YDL047W	YNL101W
YDL047W	YNL187W
YDL047W	YNR016C
YDL047W	YOR259C
YDL047W	YOR267C
YDL047W	YOR317W
YDL047W	YOR374W
YDL047W	YOR375C
YDL047W	YPL061W
YDL047W	YPL204W
YDL049C	YMR289W
YDL049C	YOR311C
YDL050C	YDR318W
YDL051W	YDR365C
YDL051W	YDR395W
YDL051W	YGR162W
YDL051W	YHR089C
YDL051W	YLR074C

YDL051W	YNL016W
YDL051W	YOL139C
YDL051W	YPR016C
YDL051W	YPR088C
YDL052C	YDL143W
YDL052C	YDR170C
YDL052C	YER110C
YDL052C	YFR009W
YDL052C	YGL195W
YDL052C	YHL030W
YDL052C	YJR091C
YDL052C	YKR026C
YDL052C	YLR071C
YDL052C	YML115C
YDL052C	YMR106C
YDL052C	YNL138W
YDL053C	YDR318W
YDL053C	YGL070C
YDL053C	YIL106W
YDL053C	YJL057C
YDL053C	YLR362W
YDL053C	YPL042C
YDL053C	YPL140C
YDL053C	YPR106W
YDL054C	YDL212W
YDL054C	YDR307W
YDL054C	YEL027W
YDL054C	YER140W
YDL054C	YGL051W
YDL054C	YGR173W
YDL054C	YGR174C
YDL054C	YHR026W
YDL054C	YLR372W
YDL054C	YMR013C
YDL054C	YOR016C
YDL055C	YDR266C
YDL055C	YDR386W
YDL055C	YDR388W
YDL055C	YDR394W
YDL055C	YDR419W
YDL055C	YER012W
YDL055C	YER020W
YDL055C	YER179W
YDL055C	YFR040W
YDL055C	YGL208W

YDL055C	YGL237C
YDL055C	YGR067C
YDL055C	YGR262C
YDL055C	YHR030C
YDL055C	YHR135C
YDL055C	YIL066C
YDL055C	YJL069C
YDL055C	YJL173C
YDL055C	YJR022W
YDL055C	YJR035W
YDL055C	YJR042W
YDL055C	YJR076C
YDL055C	YKL095W
YDL055C	YKL108W
YDL055C	YKR055W
YDL055C	YLR097C
YDL055C	YLR340W
YDL055C	YLR347C
YDL055C	YLR442C
YDL055C	YML057W
YDL055C	YMR106C
YDL055C	YNL056W
YDL055C	YNL113W
YDL055C	YNL161W
YDL055C	YNL189W
YDL055C	YOL108C
YDL055C	YOL133W
YDL055C	YOR080W
YDL055C	YOR125C
YDL055C	YPL140C
YDL055C	YPL150W
YDL055C	YPR054W
YDL055C	YPR110C
YDL056W	YHR206W
YDL056W	YIL131C
YDL056W	YLR182W
YDL058W	YNL030W
YDL059C	YDL126C
YDL059C	YDL130W
YDL059C	YDR148C
YDL059C	YDR214W
YDL059C	YDR394W
YDL059C	YEL030W
YDL059C	YEL051W
YDL059C	YEL060C

YDL059C	YER025W
YDL059C	YER031C
YDL059C	YER052C
YDL059C	YER062C
YDL059C	YER110C
YDL059C	YER138C
YDL059C	YER155C
YDL059C	YER177W
YDL059C	YFL016C
YDL059C	YFL037W
YDL059C	YFL039C
YDL059C	YFL045C
YDL059C	YFL059W
YDL059C	YFR053C
YDL059C	YGL048C
YDL059C	YGL137W
YDL059C	YGR049W
YDL059C	YGR086C
YDL059C	YGR282C
YDL059C	YHR179W
YDL059C	YIL021W
YDL059C	YIL053W
YDL059C	YIL075C
YDL059C	YJL008C
YDL059C	YJL034W
YDL059C	YJL088W
YDL059C	YJL130C
YDL059C	YJR045C
YDL059C	YJR121W
YDL059C	YKL035W
YDL059C	YKL081W
YDL059C	YKL127W
YDL059C	YKL152C
YDL059C	YKL210W
YDL059C	YLR180W
YDL059C	YLR259C
YDL059C	YML032C
YDL059C	YML085C
YDL059C	YMR105C
YDL059C	YMR108W
YDL059C	YMR205C
YDL059C	YMR214W
YDL059C	YNL064C
YDL059C	YNR001C
YDL059C	YOR151C

YDL059C	YPL061W
YDL059C	YPL235W
YDL060W	YDL213C
YDL060W	YDR224C
YDL060W	YER006W
YDL060W	YER165W
YDL060W	YGR081C
YDL060W	YKL143W
YDL060W	YNL098C
YDL060W	YNL207W
YDL060W	YOL041C
YDL060W	YOL123W
YDL060W	YOR056C
YDL060W	YOR145C
YDL060W	YPL012W
YDL060W	YPL042C
YDL060W	YPL204W
YDL060W	YPL266W
YDL061C	YDR318W
YDL062W	YLR277C
YDL063C	YDR381W
YDL063C	YPL131W
YDL064W	YDR409W
YDL064W	YDR510W
YDL064W	YER095W
YDL064W	YJR076C
YDL064W	YMR168C
YDL064W	YPL161C
YDL065C	YDR034C
YDL065C	YDR235W
YDL065C	YDR244W
YDL065C	YDR265W
YDL065C	YDR329C
YDL065C	YER066C-A
YDL065C	YGL153W
YDL065C	YGR119C
YDL065C	YGR218W
YDL065C	YGR242W
YDL065C	YJL210W
YDL065C	YKL189W
YDL065C	YLR063W
YDL065C	YLR071C
YDL065C	YML042W
YDL065C	YMR026C
YDL065C	YMR047C

YDL065C	YMR155W
YDL065C	YMR163C
YDL065C	YMR284W
YDL065C	YNL214W
YDL065C	YOL147C
YDL065C	YPL204W
YDL066W	YJL098W
YDL066W	YMR047C
YDL066W	YOR014W
YDL066W	YPL109C
YDL067C	YDR318W
YDL067C	YGL187C
YDL067C	YHR051W
YDL067C	YNL052W
YDL069C	YJL095W
YDL070W	YER133W
YDL070W	YGL063W
YDL070W	YGL075C
YDL070W	YLR399C
YDL070W	YNL030W
YDL070W	YOL131W
YDL071C	YDR183W
YDL071C	YEL068C
YDL071C	YFL017C
YDL071C	YGR269W
YDL071C	YNL155W
YDL072C	YFR024C-A
YDL072C	YLR310C
YDL072C	YNL079C
YDL072C	YPL126W
YDL073W	YER118C
YDL074C	YDR295C
YDL074C	YEL043W
YDL074C	YFL008W
YDL074C	YFR031C
YDL074C	YHR149C
YDL074C	YJL074C
YDL074C	YNL153C
YDL074C	YOL069W
YDL074C	YOR195W
YDL074C	YPL055C
YDL074C	YPR086W
YDL075W	YJR091C
YDL076C	YDR207C
YDL076C	YER088C

YDL076C	YIL084C
YDL076C	YLR098C
YDL076C	YMR263W
YDL076C	YNL330C
YDL076C	YOL004W
YDL076C	YOR174W
YDL076C	YPL139C
YDL076C	YPL181W
YDL077C	YDR080W
YDL077C	YGL124C
YDL077C	YMR231W
YDL077C	YNL147W
YDL077C	YPL045W
YDL079C	YJL014W
YDL079C	YJL098W
YDL079C	YKL103C
YDL079C	YPL235W
YDL079C	YPR019W
YDL080C	YDR081C
YDL080C	YGR196C
YDL080C	YIL142W
YDL081C	YEL054C
YDL081C	YGR209C
YDL081C	YLR295C
YDL081C	YLR340W
YDL081C	YMR047C
YDL081C	YOR229W
YDL081C	YPL005W
YDL081C	YPR078C
YDL081C	YPR086W
YDL082W	YHR197W
YDL084W	YDR138W
YDL084W	YDR381W
YDL084W	YER063W
YDL084W	YHR102W
YDL084W	YMR314W
YDL085W	YDR034C
YDL085W	YGR139W
YDL085W	YPR054W
YDL086W	YHR169W
YDL086W	YPR086W
YDL087C	YDR080W
YDL087C	YDR235W
YDL087C	YDR240C
YDL087C	YER029C

YDL087C YFL017W-A
YDL087C YGL112C
YDL087C YGR013W
YDL087C YGR162W
YDL087C YHR086W
YDL087C YIL061C
YDL087C YJL074C
YDL087C YKL012W
YDL087C YKL173W
YDL087C YLR147C
YDL087C YLR275W
YDL087C YLR298C
YDL087C YML046W
YDL087C YML049C
YDL087C YMR125W
YDL087C YMR288W
YDL087C YOL139C
YDL087C YPL178W
YDL087C YPR182W
YDL088C YLR347C
YDL088C YML031W
YDL088C YMR153W
YDL089W YDR233C
YDL089W YIR038C
YDL089W YLR324W
YDL089W YML008C
YDL089W YPR028W
YDL090C YKL019W
YDL090C YKL152C
YDL091C YIL022W
YDL092W YKL122C
YDL092W YPR088C
YDL093W YGL055W
YDL094C YDL196W
YDL094C YLR115W
YDL095W YHR096C
YDL096C YDR318W
YDL097C YDL147W
YDL097C YDR142C
YDL097C YDR394W
YDL097C YDR427W
YDL097C YEL009C
YDL097C YEL037C
YDL097C YER012W
YDL097C YER021W

YDL097C	YFR004W
YDL097C	YFR010W
YDL097C	YFR052W
YDL097C	YGL004C
YDL097C	YGL048C
YDL097C	YGR040W
YDL097C	YGR232W
YDL097C	YGR262C
YDL097C	YHL030W
YDL097C	YHR027C
YDL097C	YHR030C
YDL097C	YHR200W
YDL097C	YKL145W
YDL097C	YLR421C
YDL097C	YOR117W
YDL097C	YOR259C
YDL097C	YOR261C
YDL097C	YPR108W
YDL098C	YDR318W
YDL098C	YER172C
YDL098C	YGR075C
YDL098C	YLR147C
YDL098C	YNL147W
YDL098C	YPR178W
YDL099W	YKL050C
YDL099W	YML102W
YDL100C	YDL121C
YDL100C	YDR259C
YDL100C	YDR304C
YDL100C	YDR415C
YDL100C	YDR431W
YDL100C	YDR513W
YDL100C	YER083C
YDL100C	YGL020C
YDL100C	YGR189C
YDL100C	YGR254W
YDL100C	YHL004W
YDL100C	YHR005C
YDL100C	YHR057C
YDL100C	YHR114W
YDL100C	YHR128W
YDL100C	YHR180W
YDL100C	YIL162W
YDL100C	YJL075C
YDL100C	YJL153C

YDL100C	YKL062W
YDL100C	YKR100C
YDL100C	YLR044C
YDL100C	YML048W
YDL100C	YML101C
YDL100C	YNL055C
YDL100C	YNL199C
YDL100C	YOL055C
YDL100C	YOL111C
YDL100C	YOL126C
YDL100C	YOL159C
YDL100C	YOR164C
YDL100C	YPL252C
YDL100C	YPR037C
YDL101C	YDL200C
YDL101C	YDL229W
YDL101C	YDR030C
YDL101C	YDR032C
YDL101C	YDR051C
YDL101C	YDR155C
YDL101C	YDR158W
YDL101C	YDR214W
YDL101C	YDR217C
YDL101C	YER125W
YDL101C	YER173W
YDL101C	YFL030W
YDL101C	YFL037W
YDL101C	YFL039C
YDL101C	YFR028C
YDL101C	YFR053C
YDL101C	YGL253W
YDL101C	YGL256W
YDL101C	YGR086C
YDL101C	YGR130C
YDL101C	YHR082C
YDL101C	YHR135C
YDL101C	YHR179W
YDL101C	YIL094C
YDL101C	YJL076W
YDL101C	YJL092W
YDL101C	YJR045C
YDL101C	YJR104C
YDL101C	YKL060C
YDL101C	YKL095W
YDL101C	YKL104C

YDL101C	YLR044C
YDL101C	YLR234W
YDL101C	YLR259C
YDL101C	YLR355C
YDL101C	YML058W
YDL101C	YMR031C
YDL101C	YMR086W
YDL101C	YMR105C
YDL101C	YMR226C
YDL101C	YNL088W
YDL101C	YNL250W
YDL101C	YOL082W
YDL101C	YOL123W
YDL101C	YOL139C
YDL101C	YOR332W
YDL101C	YPL022W
YDL101C	YPL129W
YDL101C	YPL153C
YDL101C	YPL269W
YDL102W	YJR006W
YDL102W	YJR043C
YDL103C	YDR288W
YDL104C	YGR292W
YDL105W	YDR288W
YDL105W	YER081W
YDL105W	YHR216W
YDL105W	YLR216C
YDL105W	YMR186W
YDL105W	YNR031C
YDL105W	YOR027W
YDL106C	YDR146C
YDL106C	YFR034C
YDL106C	YKR099W
YDL108W	YDL140C
YDL108W	YDR460W
YDL108W	YER171W
YDL108W	YGL134W
YDL108W	YIL021W
YDL108W	YIL094C
YDL108W	YLR005W
YDL108W	YOR151C
YDL108W	YOR299W
YDL108W	YPL235W
YDL108W	YPR025C
YDL110C	YDR318W

YDL110C	YKL015W
YDL110C	YLR125W
YDL110C	YOR078W
YDL111C	YDR280W
YDL111C	YGR095C
YDL111C	YGR158C
YDL111C	YGR195W
YDL111C	YHR069C
YDL111C	YJR022W
YDL111C	YNL232W
YDL111C	YOL021C
YDL111C	YOR326W
YDL112W	YER146W
YDL112W	YNL307C
YDL113C	YDL132W
YDL113C	YFR040W
YDL113C	YJL036W
YDL113C	YLR191W
YDL113C	YLR424W
YDL113C	YOL133W
YDL113C	YPL174C
YDL114W	YDL123W
YDL114W	YDR348C
YDL114W	YLR040C
YDL115C	YIL021W
YDL116W	YDL126C
YDL116W	YDR113C
YDL116W	YDR192C
YDL116W	YDR335W
YDL116W	YFR030W
YDL116W	YGL092W
YDL116W	YGL100W
YDL116W	YGL172W
YDL116W	YGL179C
YDL116W	YGL263W
YDL116W	YGR119C
YDL116W	YHL004W
YDL116W	YHR033W
YDL116W	YJL090C
YDL116W	YJL117W
YDL116W	YJR042W
YDL116W	YKL057C
YDL116W	YKL068W
YDL116W	YKR082W
YDL116W	YLR208W

YDL116W YMR047C
YDL116W YNL118C
YDL116W YOL014W
YDL116W YOL131W
YDL116W YOR180C
YDL116W YOR294W
YDL116W YPL091W
YDL116W YPL124W
YDL117W YDR104C
YDL117W YDR208W
YDL117W YDR211W
YDL117W YDR239C
YDL117W YER032W
YDL117W YER081W
YDL117W YER087W
YDL117W YER118C
YDL117W YER177W
YDL117W YGL060W
YDL117W YGL099W
YDL117W YGL144C
YDL117W YGR037C
YDL117W YGR233C
YDL117W YGR276C
YDL117W YIL156W
YDL117W YJL083W
YDL117W YJL155C
YDL117W YJR138W
YDL117W YKL048C
YDL117W YKR027W
YDL117W YLL028W
YDL117W YLR073C
YDL117W YLR142W
YDL117W YLR187W
YDL117W YLR219W
YDL117W YLR240W
YDL117W YLR410W
YDL117W YLR442C
YDL117W YMR032W
YDL117W YNL078W
YDL117W YNL152W
YDL117W YNL277W-A
YDL117W YNR016C
YDL117W YOL070C
YDL117W YOL095C
YDL117W YOR181W

YDL117W	YPL009C
YDL117W	YPL038W
YDL117W	YPL158C
YDL118W	YDR318W
YDL118W	YLR295C
YDL119C	YDR162C
YDL121C	YMR305C
YDL123W	YHR027C
YDL123W	YPR103W
YDL123W	YPR108W
YDL124W	YNL244C
YDL125C	YER075C
YDL125C	YJR091C
YDL126C	YDL145C
YDL126C	YDL190C
YDL126C	YDL200C
YDL126C	YDR028C
YDL126C	YDR037W
YDL126C	YDR049W
YDL126C	YDR075W
YDL126C	YDR143C
YDL126C	YDR247W
YDL126C	YDR267C
YDL126C	YDR283C
YDL126C	YDR335W
YDL126C	YDR337W
YDL126C	YDR388W
YDL126C	YDR477W
YDL126C	YER007W
YDL126C	YER112W
YDL126C	YER133W
YDL126C	YFR053C
YDL126C	YGL081W
YDL126C	YGL115W
YDL126C	YGL131C
YDL126C	YGL137W
YDL126C	YGL158W
YDL126C	YGL246C
YDL126C	YGR040W
YDL126C	YGR048W
YDL126C	YGR052W
YDL126C	YGR078C
YDL126C	YGR262C
YDL126C	YHR030C
YDL126C	YHR169W

YDL126C YHR188C
YDL126C YHR196W
YDL126C YIL046W
YDL126C YIL148W
YDL126C YJL203W
YDL126C YJR017C
YDL126C YJR045C
YDL126C YJR052W
YDL126C YJR090C
YDL126C YKL078W
YDL126C YKL139W
YDL126C YKL166C
YDL126C YKL213C
YDL126C YLR097C
YDL126C YLR175W
YDL126C YLR191W
YDL126C YLR229C
YDL126C YLR291C
YDL126C YML064C
YDL126C YML068W
YDL126C YMR001C
YDL126C YMR049C
YDL126C YMR055C
YDL126C YMR059W
YDL126C YMR106C
YDL126C YMR117C
YDL126C YMR240C
YDL126C YMR284W
YDL126C YNL012W
YDL126C YNL106C
YDL126C YNL128W
YDL126C YNL135C
YDL126C YNL271C
YDL126C YOL094C
YDL126C YOL126C
YDL126C YOL133W
YDL126C YOR304W
YDL126C YOR341W
YDL126C YOR351C
YDL126C YPL022W
YDL126C YPL031C
YDL126C YPL204W
YDL126C YPR015C
YDL126C YPR054W
YDL126C YPR093C

YDL126C	YPR110C
YDL126C	YPR111W
YDL126C	YPR178W
YDL127W	YDR146C
YDL127W	YDR388W
YDL127W	YKL002W
YDL127W	YLR453C
YDL127W	YPL031C
YDL128W	YER005W
YDL128W	YGR295C
YDL128W	YKL102C
YDL128W	YPR124W
YDL129W	YJL020C
YDL129W	YJR063W
YDL130W	YDR367W
YDL130W	YDR382W
YDL130W	YDR399W
YDL130W	YEL054C
YDL130W	YJL173C
YDL130W	YJL208C
YDL130W	YJL210W
YDL130W	YJR012C
YDL130W	YJR108W
YDL130W	YJR144W
YDL130W	YKL002W
YDL130W	YLR051C
YDL130W	YLR287C
YDL130W	YLR340W
YDL131W	YLR427W
YDL132W	YDR054C
YDL132W	YDR099W
YDL132W	YDR139C
YDL132W	YDR274C
YDL132W	YDR285W
YDL132W	YDR306C
YDL132W	YDR328C
YDL132W	YDR463W
YDL132W	YDR497C
YDL132W	YEL030W
YDL132W	YEL060C
YDL132W	YER091C
YDL132W	YER156C
YDL132W	YER177W
YDL132W	YFL009W
YDL132W	YGL249W

YDL132W	YGR087C
YDL132W	YGR171C
YDL132W	YHR073W
YDL132W	YHR124W
YDL132W	YIL046W
YDL132W	YJL052W
YDL132W	YJL138C
YDL132W	YJL149W
YDL132W	YJL153C
YDL132W	YJR090C
YDL132W	YJR138W
YDL132W	YLR044C
YDL132W	YLR079W
YDL132W	YLR097C
YDL132W	YLR100W
YDL132W	YLR128W
YDL132W	YLR134W
YDL132W	YLR259C
YDL132W	YLR267W
YDL132W	YLR352W
YDL132W	YLR368W
YDL132W	YML046W
YDL132W	YML088W
YDL132W	YMR186W
YDL132W	YMR297W
YDL132W	YNL015W
YDL132W	YNL055C
YDL132W	YNR001C
YDL132W	YOL055C
YDL132W	YOL133W
YDL132W	YOR057W
YDL132W	YOR080W
YDL132W	YOR133W
YDL132W	YPL110C
YDL132W	YPL240C
YDL132W	YPL256C
YDL132W	YPL258C
YDL132W	YPR124W
YDL132W	YPR159W
YDL133W	YMR052W
YDL134C	YDL188C
YDL134C	YDR523C
YDL134C	YER159C-A
YDL134C	YFL039C
YDL134C	YGL190C

YDL134C	YGR142W
YDL134C	YGR161C
YDL134C	YHR075C
YDL134C	YHR114W
YDL134C	YHR135C
YDL134C	YIL021W
YDL134C	YJL008C
YDL134C	YLR259C
YDL134C	YLR373C
YDL134C	YMR028W
YDL134C	YMR205C
YDL134C	YMR214W
YDL134C	YNL064C
YDL134C	YOR014W
YDL134C	YOR176W
YDL134C	YPL152W
YDL134C	YPL235W
YDL134C	YPR040W
YDL134C	YPR110C
YDL135C	YKR055W
YDL135C	YLR229C
YDL135C	YNL176C
YDL135C	YPR165W
YDL137W	YDL192W
YDL137W	YDR007W
YDL137W	YGR052W
YDL137W	YHR188C
YDL137W	YPR110C
YDL138W	YDR034C
YDL138W	YEL026W
YDL138W	YIR039C
YDL138W	YJL130C
YDL138W	YJL186W
YDL138W	YJR121W
YDL138W	YLL015W
YDL138W	YLR180W
YDL138W	YLR313C
YDL138W	YMR035W
YDL138W	YNL064C
YDL138W	YPL145C
YDL138W	YPR023C
YDL138W	YPR077C
YDL138W	YPR079W
YDL139C	YDR388W
YDL139C	YGL153W

YDL139C	YMR109W
YDL139C	YPR154W
YDL140C	YDR138W
YDL140C	YDR167W
YDL140C	YDR228C
YDL140C	YDR301W
YDL140C	YDR404C
YDL140C	YEL037C
YDL140C	YER125W
YDL140C	YGL043W
YDL140C	YGL070C
YDL140C	YGL130W
YDL140C	YGR136W
YDL140C	YJL020C
YDL140C	YJL140W
YDL140C	YJL164C
YDL140C	YJR017C
YDL140C	YJR110W
YDL140C	YKR062W
YDL140C	YLR015W
YDL140C	YLR115W
YDL140C	YLR191W
YDL140C	YLR384C
YDL140C	YLR418C
YDL140C	YMR201C
YDL140C	YNL236W
YDL140C	YNL251C
YDL140C	YNL266W
YDL140C	YOL005C
YDL140C	YOL051W
YDL140C	YOL145C
YDL140C	YOR151C
YDL140C	YOR210W
YDL140C	YOR224C
YDL140C	YPL129W
YDL140C	YPL203W
YDL140C	YPL228W
YDL140C	YPR093C
YDL140C	YPR154W
YDL140C	YPR187W
YDL143W	YDL156W
YDL143W	YDR030C
YDL143W	YDR075W
YDL143W	YDR142C
YDL143W	YER082C

YDL143W	YER171W
YDL143W	YER173W
YDL143W	YGL003C
YDL143W	YGL116W
YDL143W	YGL119W
YDL143W	YGL137W
YDL143W	YGL190C
YDL143W	YGR040W
YDL143W	YKL095W
YDL143W	YKR026C
YDL143W	YKR036C
YDL143W	YLL011W
YDL143W	YLR196W
YDL143W	YML064C
YDL143W	YNL317W
YDL143W	YOR212W
YDL144C	YDR088C
YDL145C	YDR099W
YDL145C	YDR171W
YDL145C	YDR190C
YDL145C	YDR216W
YDL145C	YDR238C
YDL145C	YEL060C
YDL145C	YER103W
YDL145C	YER112W
YDL145C	YER122C
YDL145C	YFL039C
YDL145C	YFR051C
YDL145C	YGL081W
YDL145C	YGL137W
YDL145C	YGR254W
YDL145C	YHR020W
YDL145C	YIL004C
YDL145C	YIL076W
YDL145C	YIL147C
YDL145C	YJL066C
YDL145C	YJL117W
YDL145C	YJL157C
YDL145C	YJL187C
YDL145C	YJR045C
YDL145C	YJR077C
YDL145C	YKL108W
YDL145C	YKL211C
YDL145C	YLR078C
YDL145C	YLR180W

YDL145C	YLR259C
YDL145C	YLR268W
YDL145C	YML124C
YDL145C	YMR106C
YDL145C	YMR108W
YDL145C	YNL258C
YDL145C	YNL284C
YDL145C	YNL287W
YDL145C	YOL087C
YDL145C	YOR133W
YDL145C	YOR375C
YDL145C	YPL010W
YDL145C	YPL222W
YDL145C	YPL240C
YDL146W	YFR024C-A
YDL146W	YHR016C
YDL146W	YHR114W
YDL146W	YKL070W
YDL146W	YKL129C
YDL146W	YMR109W
YDL147W	YDL216C
YDL147W	YDR069C
YDL147W	YDR073W
YDL147W	YDR179C
YDL147W	YDR201W
YDL147W	YDR341C
YDL147W	YDR363W-A
YDL147W	YDR394W
YDL147W	YDR427W
YDL147W	YER012W
YDL147W	YER021W
YDL147W	YFR004W
YDL147W	YFR010W
YDL147W	YFR052W
YDL147W	YFR053C
YDL147W	YGL004C
YDL147W	YGL011C
YDL147W	YGL048C
YDL147W	YGL127C
YDL147W	YGL200C
YDL147W	YGR092W
YDL147W	YGR232W
YDL147W	YGR262C
YDL147W	YHL030W
YDL147W	YHR027C

YDL147W	YHR200W
YDL147W	YIL075C
YDL147W	YIL125W
YDL147W	YJL001W
YDL147W	YJR045C
YDL147W	YJR091C
YDL147W	YKL145W
YDL147W	YLR421C
YDL147W	YMR080C
YDL147W	YMR314W
YDL147W	YOL126C
YDL147W	YOR117W
YDL147W	YOR259C
YDL147W	YOR261C
YDL147W	YPL026C
YDL147W	YPR103W
YDL147W	YPR108W
YDL147W	YPR159W
YDL148C	YER179W
YDL148C	YGR090W
YDL148C	YLR295C
YDL149W	YFR028C
YDL149W	YKL064W
YDL149W	YLR065C
YDL149W	YOR061W
YDL149W	YPR041W
YDL150W	YGR047C
YDL150W	YKL144C
YDL150W	YKR025W
YDL150W	YNR003C
YDL150W	YOR116C
YDL150W	YPR110C
YDL153C	YDL226C
YDL153C	YDR040C
YDL153C	YDR269C
YDL153C	YDR307W
YDL153C	YDR422C
YDL153C	YFL001W
YDL153C	YGL120C
YDL153C	YGL185C
YDL153C	YGL198W
YDL153C	YGL250W
YDL153C	YGR160W
YDL153C	YGR290W
YDL153C	YHR203C

YDL153C	YJL076W
YDL153C	YJL175W
YDL153C	YJR002W
YDL153C	YLR006C
YDL153C	YNL125C
YDL153C	YNL216W
YDL153C	YNL280C
YDL153C	YOL118C
YDL153C	YOR252W
YDL153C	YOR309C
YDL153C	YOR314W
YDL153C	YOR321W
YDL153C	YPL026C
YDL153C	YPL076W
YDL153C	YPL204W
YDL153C	YPR029C
YDL154W	YER173W
YDL154W	YFL003C
YDL154W	YGL025C
YDL154W	YGL170C
YDL154W	YIL144W
YDL154W	YJR082C
YDL154W	YLR394W
YDL154W	YMR224C
YDL155W	YDR034C
YDL155W	YDR216W
YDL155W	YDR507C
YDL155W	YER040W
YDL155W	YER107C
YDL155W	YER118C
YDL155W	YFR028C
YDL155W	YGL003C
YDL155W	YGL197W
YDL155W	YGR092W
YDL155W	YHR033W
YDL155W	YIL115C
YDL155W	YJL076W
YDL155W	YKL068W
YDL155W	YKR010C
YDL155W	YLR079W
YDL155W	YLR131C
YDL155W	YLR310C
YDL155W	YMR036C
YDL155W	YMR047C
YDL155W	YNL076W

YDL155W	YOR081C
YDL155W	YOR207C
YDL155W	YOR210W
YDL155W	YOR341W
YDL155W	YOR372C
YDL155W	YPL124W
YDL155W	YPL267W
YDL155W	YPR010C-A
YDL155W	YPR110C
YDL156W	YDR097C
YDL156W	YFR024C-A
YDL156W	YFR052W
YDL156W	YHR016C
YDL156W	YIL142W
YDL156W	YJL014W
YDL156W	YJL111W
YDL156W	YLR116W
YDL158C	YPR086W
YDL159W	YDR103W
YDL159W	YDR206W
YDL159W	YDR356W
YDL159W	YFR028C
YDL159W	YGR040W
YDL159W	YIL113W
YDL159W	YLL021W
YDL159W	YLR113W
YDL159W	YLR313C
YDL159W	YMR276W
YDL159W	YOL123W
YDL159W	YPL124W
YDL159W-A	YPR154W
YDL160C	YDR170C
YDL160C	YEL015W
YDL160C	YER081W
YDL160C	YER125W
YDL160C	YER140W
YDL160C	YGL137W
YDL160C	YGL178W
YDL160C	YGR178C
YDL160C	YIL106W
YDL160C	YJL124C
YDL160C	YLR373C
YDL160C	YMR106C
YDL160C	YNL147W
YDL160C	YNR052C

YDL160C	YOL149W
YDL160C	YPR110C
YDL161W	YHR016C
YDL161W	YIL095W
YDL161W	YIR006C
YDL161W	YLR116W
YDL161W	YOR111W
YDL161W	YOR181W
YDL162C	YDR318W
YDL162C	YKL002W
YDL164C	YDR148C
YDL164C	YDR502C
YDL164C	YEL030W
YDL164C	YFL018C
YDL164C	YGR282C
YDL164C	YHR178W
YDL164C	YJR045C
YDL164C	YJR121W
YDL164C	YKL035W
YDL164C	YKL182W
YDL164C	YLR180W
YDL164C	YLR276C
YDL164C	YNL064C
YDL164C	YOL055C
YDL164C	YOR378W
YDL165W	YDR443C
YDL165W	YDR448W
YDL165W	YER068W
YDL165W	YER148W
YDL165W	YGL127C
YDL165W	YGR014W
YDL165W	YIL034C
YDL165W	YKL002W
YDL165W	YLR125W
YDL165W	YNL025C
YDL165W	YNL288W
YDL165W	YNL315C
YDL165W	YPL042C
YDL165W	YPR072W
YDL166C	YLR208W
YDL166C	YLR222C
YDL167C	YNL016W
YDL168W	YER081W
YDL171C	YER117W
YDL171C	YMR117C

YDL171C	YPR086W
YDL172C	YDR328C
YDL172C	YHR114W
YDL172C	YLR295C
YDL173W	YNL183C
YDL175C	YDR023W
YDL175C	YDR432W
YDL175C	YEL070W
YDL175C	YGR165W
YDL175C	YJL124C
YDL175C	YKL035W
YDL175C	YMR125W
YDL175C	YMR303C
YDL175C	YNL189W
YDL175C	YOL115W
YDL175C	YOR204W
YDL175C	YPL093W
YDL175C	YPR191W
YDL178W	YFL039C
YDL178W	YGL061C
YDL179W	YDR171W
YDL179W	YDR388W
YDL179W	YKL152C
YDL179W	YKR048C
YDL179W	YLR259C
YDL179W	YML085C
YDL179W	YMR147W
YDL179W	YOR083W
YDL179W	YPL031C
YDL179W	YPL235W
YDL179W	YPR191W
YDL180W	YLR452C
YDL181W	YDR318W
YDL181W	YGR089W
YDL181W	YJR121W
YDL181W	YKR054C
YDL181W	YLL021W
YDL181W	YNL250W
YDL183C	YGL070C
YDL185W	YDR202C
YDL185W	YDR328C
YDL185W	YEL051W
YDL185W	YER171W
YDL185W	YER179W
YDL185W	YGL201C

YDL185W	YGR020C
YDL185W	YGR092W
YDL185W	YIL144W
YDL185W	YJL069C
YDL185W	YJR033C
YDL185W	YJR035W
YDL185W	YJR062C
YDL185W	YKL048C
YDL185W	YKL103C
YDL185W	YKR026C
YDL185W	YLL019C
YDL185W	YLR222C
YDL185W	YLR337C
YDL185W	YLR442C
YDL185W	YML095C
YDL185W	YMR054W
YDL185W	YMR104C
YDL185W	YNL250W
YDL185W	YNL313C
YDL185W	YNR052C
YDL185W	YOR174W
YDL185W	YOR270C
YDL185W	YPR110C
YDL186W	YJL167W
YDL186W	YLR433C
YDL186W	YLR452C
YDL186W	YMR001C
YDL188C	YDR171W
YDL188C	YDR190C
YDL188C	YDR523C
YDL188C	YER012W
YDL188C	YER094C
YDL188C	YER159C-A
YDL188C	YFR050C
YDL188C	YGL011C
YDL188C	YGL121C
YDL188C	YGL190C
YDL188C	YGR135W
YDL188C	YGR161C
YDL188C	YGR253C
YDL188C	YGR282C
YDL188C	YHR075C
YDL188C	YHR135C
YDL188C	YJL008C
YDL188C	YJL066C

YDL188C	YJL130C
YDL188C	YJR009C
YDL188C	YLR259C
YDL188C	YML092C
YDL188C	YML109W
YDL188C	YML126C
YDL188C	YMR028W
YDL188C	YMR205C
YDL188C	YMR214W
YDL188C	YMR273C
YDL188C	YMR314W
YDL188C	YNL064C
YDL188C	YNL085W
YDL188C	YOL005C
YDL188C	YOL038W
YDL188C	YOR014W
YDL188C	YOR151C
YDL188C	YOR157C
YDL188C	YOR362C
YDL188C	YPL003W
YDL188C	YPL235W
YDL188C	YPR010C
YDL188C	YPR040W
YDL188C	YPR103W
YDL189W	YGR218W
YDL190C	YDR049W
YDL190C	YEL037C
YDL190C	YER057C
YDL190C	YIL148W
YDL190C	YKL010C
YDL190C	YML100W
YDL190C	YMR066W
YDL190C	YMR276W
YDL190C	YPL236C
YDL190C	YPL240C
YDL191W	YDR101C
YDL191W	YGL099W
YDL191W	YGL111W
YDL191W	YHR066W
YDL191W	YHR197W
YDL192W	YDL200C
YDL192W	YDR099W
YDL192W	YDR477W
YDL192W	YER165W
YDL192W	YGL206C

YDL192W YGL245W
YDL192W YGR130C
YDL192W YGR254W
YDL192W YHR188C
YDL192W YIL004C
YDL192W YKL060C
YDL192W YLR078C
YDL192W YLR268W
YDL192W YML028W
YDL192W YMR059W
YDL192W YMR094W
YDL192W YNL287W
YDL192W YPR110C
YDL192W YPR176C
YDL193W YER110C
YDL193W YFL022C
YDL193W YFL039C
YDL193W YGL008C
YDL193W YGL195W
YDL193W YGR062C
YDL193W YJR121W
YDL193W YJR132W
YDL193W YKL152C
YDL193W YLR293C
YDL193W YLR447C
YDL193W YOR026W
YDL193W YOR185C
YDL195W YDR337W
YDL195W YDR382W
YDL195W YGL019W
YDL195W YGL200C
YDL195W YIL035C
YDL195W YIL109C
YDL195W YLR150W
YDL195W YLR208W
YDL195W YLR429W
YDL195W YML012W
YDL195W YML028W
YDL195W YNL112W
YDL195W YOR061W
YDL195W YOR250C
YDL195W YPL085W
YDL195W YPR181C
YDL196W YER071C
YDL198C YDR414C

YDL198C	YML123C
YDL199C	YJL004C
YDL199C	YMR243C
YDL199C	YOR037W
YDL200C	YDR155C
YDL200C	YDR214W
YDL200C	YDR224C
YDL200C	YEL024W
YDL200C	YFL037W
YDL200C	YFL039C
YDL200C	YFR053C
YDL200C	YGL048C
YDL200C	YGL121C
YDL200C	YGL137W
YDL200C	YGL206C
YDL200C	YGR184C
YDL200C	YGR282C
YDL200C	YHR033W
YDL200C	YHR082C
YDL200C	YHR183W
YDL200C	YIL075C
YDL200C	YIL148W
YDL200C	YJL008C
YDL200C	YJL066C
YDL200C	YJL088W
YDL200C	YJL130C
YDL200C	YJR045C
YDL200C	YJR077C
YDL200C	YJR121W
YDL200C	YKL010C
YDL200C	YKL056C
YDL200C	YKL073W
YDL200C	YKL152C
YDL200C	YKL182W
YDL200C	YLL011W
YDL200C	YLR109W
YDL200C	YLR180W
YDL200C	YLR259C
YDL200C	YML085C
YDL200C	YMR205C
YDL200C	YNL014W
YDL200C	YNL064C
YDL200C	YOR151C
YDL200C	YOR232W
YDL200C	YPL235W

YDL200C	YPL258C
YDL201W	YDR165W
YDL202W	YER009W
YDL202W	YER022W
YDL203C	YER125W
YDL203C	YGR058W
YDL203C	YJL110C
YDL203C	YLR371W
YDL203C	YOR372C
YDL203C	YPL124W
YDL204W	YER118C
YDL204W	YGL137W
YDL204W	YMR110C
YDL204W	YOL065C
YDL204W	YPR154W
YDL207W	YDR192C
YDL207W	YIL115C
YDL207W	YKL068W
YDL207W	YMR255W
YDL207W	YOR046C
YDL208W	YDR386W
YDL208W	YDR496C
YDL208W	YER029C
YDL208W	YHR089C
YDL208W	YJL109C
YDL208W	YJR091C
YDL208W	YKL014C
YDL208W	YKL173W
YDL208W	YLR175W
YDL208W	YLR409C
YDL208W	YMR049C
YDL208W	YMR290C
YDL208W	YMR310C
YDL208W	YNL124W
YDL208W	YOR206W
YDL208W	YOR308C
YDL208W	YPR178W
YDL209C	YHR165C
YDL209C	YKL095W
YDL209C	YLR426W
YDL209C	YMR213W
YDL209C	YPR182W
YDL210W	YDL212W
YDL210W	YGL200C
YDL210W	YGL230C

YDL210W YHR122W
YDL210W YKL065C
YDL210W YLR292C
YDL210W YLR453C
YDL210W YML048W
YDL210W YNL279W
YDL212W YDR046C
YDL212W YDR276C
YDL212W YDR307W
YDL212W YDR508C
YDL212W YEL002C
YDL212W YEL017C-A
YDL212W YEL063C
YDL212W YGL200C
YDL212W YGR060W
YDL212W YGR191W
YDL212W YGR260W
YDL212W YGR295C
YDL212W YHL042W
YDL212W YHL048W
YDL212W YHR026W
YDL212W YHR094C
YDL212W YHR140W
YDL212W YHR142W
YDL212W YJL117W
YDL212W YJR010C-A
YDL212W YJR015W
YDL212W YKL212W
YDL212W YKR039W
YDL212W YLL028W
YDL212W YLL061W
YDL212W YLR018C
YDL212W YML038C
YDL212W YMR058W
YDL212W YMR149W
YDL212W YMR215W
YDL212W YNL101W
YDL212W YNL238W
YDL212W YOL020W
YDL212W YPL076W
YDL212W YPL274W
YDL212W YPR156C
YDL213C YDR091C
YDL213C YDR174W
YDL213C YDR194C

YDL213C	YDR224C
YDL213C	YDR381W
YDL213C	YDR432W
YDL213C	YDR496C
YDL213C	YEL055C
YDL213C	YER006W
YDL213C	YER165W
YDL213C	YFR032C
YDL213C	YGL068W
YDL213C	YGL120C
YDL213C	YGL173C
YDL213C	YGR103W
YDL213C	YGR145W
YDL213C	YGR150C
YDL213C	YGR162W
YDL213C	YGR198W
YDL213C	YHL034C
YDL213C	YHL038C
YDL213C	YHR052W
YDL213C	YHR089C
YDL213C	YHR099W
YDL213C	YHR114W
YDL213C	YJL109C
YDL213C	YJL138C
YDL213C	YJR041C
YDL213C	YJR144W
YDL213C	YKL014C
YDL213C	YKL182W
YDL213C	YKL193C
YDL213C	YKR024C
YDL213C	YKR081C
YDL213C	YLL008W
YDL213C	YLL027W
YDL213C	YLR175W
YDL213C	YLR196W
YDL213C	YLR347C
YDL213C	YLR432W
YDL213C	YMR049C
YDL213C	YMR229C
YDL213C	YMR290C
YDL213C	YNL061W
YDL213C	YNL132W
YDL213C	YNL189W
YDL213C	YNL308C
YDL213C	YOL041C

YDL213C YOL139C
YDL213C YOR017W
YDL213C YOR206W
YDL213C YOR272W
YDL213C YOR310C
YDL213C YPL012W
YDL213C YPR016C
YDL214C YDR216W
YDL214C YDR439W
YDL214C YEL013W
YDL214C YGL115W
YDL214C YJL066C
YDL214C YML011C
YDL215C YKL171W
YDL215C YLR320W
YDL215C YLR432W
YDL215C YPR048W
YDL216C YDR179C
YDL216C YIL071C
YDL216C YMR025W
YDL216C YOL117W
YDL217C YHR005C-A
YDL217C YHR102W
YDL217C YHR114W
YDL217C YJL054W
YDL217C YOR297C
YDL218W YGL044C
YDL219W YGL070C
YDL220C YDR082W
YDL220C YDR155C
YDL220C YEL030W
YDL220C YER091C
YDL220C YGL256W
YDL220C YGR254W
YDL220C YHR208W
YDL220C YJL034W
YDL220C YJR045C
YDL220C YKL152C
YDL220C YLR010C
YDL220C YLR233C
YDL220C YLR355C
YDL220C YMR186W
YDL220C YNL102W
YDL220C YOR133W
YDL220C YPL153C

YDL220C	YPL240C
YDL221W	YLR447C
YDL223C	YMR001C
YDL224C	YER059W
YDL224C	YIL050W
YDL224C	YML064C
YDL224C	YNL189W
YDL225W	YDL229W
YDL225W	YDR097C
YDL225W	YDR148C
YDL225W	YDR171W
YDL225W	YDR190C
YDL225W	YDR388W
YDL225W	YDR394W
YDL225W	YDR507C
YDL225W	YER066C-A
YDL225W	YFL039C
YDL225W	YGL158W
YDL225W	YGL245W
YDL225W	YGR282C
YDL225W	YHR016C
YDL225W	YHR033W
YDL225W	YHR107C
YDL225W	YJL034W
YDL225W	YJL052W
YDL225W	YJR045C
YDL225W	YJR070C
YDL225W	YJR076C
YDL225W	YJR077C
YDL225W	YKL104C
YDL225W	YLR044C
YDL225W	YLR314C
YDL225W	YML085C
YDL225W	YMR186W
YDL225W	YNR016C
YDL225W	YNR035C
YDL225W	YOR136W
YDL225W	YOR259C
YDL225W	YPL235W
YDL225W	YPR154W
YDL226C	YDR264C
YDL226C	YEL064C
YDL226C	YER118C
YDL226C	YFL039C
YDL226C	YGL161C

YDL226C	YGL198W
YDL226C	YGR172C
YDL226C	YJL151C
YDL226C	YKL126W
YDL226C	YKR088C
YDL226C	YOL129W
YDL226C	YOR327C
YDL226C	YPR113W
YDL226C	YPR183W
YDL229W	YDR142C
YDL229W	YDR267C
YDL229W	YDR324C
YDL229W	YEL056W
YDL229W	YER017C
YDL229W	YER025W
YDL229W	YER133W
YDL229W	YER179W
YDL229W	YFL033C
YDL229W	YGL137W
YDL229W	YGL190C
YDL229W	YGL213C
YDL229W	YHR117W
YDL229W	YHR135C
YDL229W	YHR186C
YDL229W	YHR199C
YDL229W	YIL142W
YDL229W	YJL128C
YDL229W	YJL164C
YDL229W	YJR007W
YDL229W	YKR026C
YDL229W	YLR291C
YDL229W	YLR335W
YDL229W	YLR383W
YDL229W	YMR102C
YDL229W	YMR117C
YDL229W	YNL064C
YDL229W	YNR031C
YDL229W	YOL062C
YDL229W	YOL126C
YDL229W	YOL139C
YDL229W	YOR089C
YDL229W	YOR098C
YDL229W	YOR174W
YDL229W	YOR212W
YDL229W	YOR351C

YDL229W	YPL031C
YDL229W	YPL151C
YDL229W	YPR110C
YDL230W	YER160C
YDL230W	YFR028C
YDL230W	YGR032W
YDL230W	YJR091C
YDL230W	YKL035W
YDL230W	YLR342W
YDL230W	YNL106C
YDL230W	YOL041C
YDL232W	YEL002C
YDL232W	YGL022W
YDL232W	YGL200C
YDL232W	YGL226C-A
YDL232W	YGR172C
YDL232W	YGR260W
YDL232W	YHR026W
YDL232W	YJL002C
YDL232W	YJR015W
YDL232W	YLL028W
YDL232W	YLR372W
YDL232W	YMR149W
YDL232W	YNL279W
YDL232W	YOL132W
YDL232W	YOR085W
YDL232W	YOR103C
YDL233W	YER149C
YDL233W	YGL181W
YDL233W	YLR295C
YDL233W	YLR447C
YDL235C	YIL147C
YDL235C	YLR006C
YDL235C	YOR272W
YDL236W	YNL189W
YDL237W	YLR085C
YDL237W	YPR148C
YDL239C	YDR148C
YDL239C	YDR176W
YDL239C	YDR273W
YDL239C	YER086W
YDL239C	YGR268C
YDL239C	YHR184W
YDL239C	YKL023W
YDL239C	YKL042W

YDL239C YKL103C
YDL239C YKL192C
YDL239C YLR072W
YDL239C YLR098C
YDL239C YLR242C
YDL239C YLR423C
YDL239C YML042W
YDL239C YMR124W
YDL239C YNL201C
YDL239C YNL225C
YDL239C YNR012W
YDL239C YOL083W
YDL239C YOL091W
YDL239C YOR127W
YDL239C YOR284W
YDL239C YOR324C
YDL239C YOR373W
YDL239C YPL049C
YDL239C YPL070W
YDL239C YPL124W
YDL239C YPL255W
YDL240W YJL020C
YDL240W YLR438C-A
YDL240W YPR165W
YDL241W YLR447C
YDL243C YNL331C
YDL245C YHR096C
YDL245C YNL121C
YDL246C YJR037W
YDL246C YJR159W
YDL246C YML064C
YDL246C YMR308C
YDL246C YNL189W
YDL246C YPL031C
YDL248W YFL004W
YDR001C YDR099W
YDR001C YER177W
YDR001C YLR270W
YDR002WYGL097W
YDR002WYGR218W
YDR002WYJL124C
YDR002WYKR048C
YDR002WYLR293C
YDR002WYMR235C
YDR003WYML001W

YDR004WYDR099W
YDR004WYER173W
YDR004WYFL018C
YDR004WYFL037W
YDR004WYFR053C
YDR004WYHR082C
YDR004WYJL023C
YDR004WYJL088W
YDR004WYLR259C
YDR004WYLR268W
YDR004WYLR394W
YDR004WYML085C
YDR004WYML124C
YDR004WYOL055C
YDR004WYOR374W
YDR004WYPL235W
YDR004WYPL258C
YDR005C YDR328C
YDR005C YER081W
YDR005C YGL044C
YDR005C YJR091C
YDR005C YOR116C
YDR006C YER177W
YDR006C YOR178C
YDR007WYLR295C
YDR007WYOR242C
YDR009WYML051W
YDR009WYOL133W
YDR010C YJR091C
YDR010C YKL129C
YDR010C YMR109W
YDR012WYER081W
YDR013WYDR489W
YDR016C YDR201W
YDR016C YGL044C
YDR016C YGL061C
YDR016C YGR113W
YDR016C YGR218W
YDR016C YKL002W
YDR016C YKL052C
YDR016C YKR037C
YDR016C YKR083C
YDR017C YDR099W
YDR017C YER177W
YDR018C YDR479C

YDR020C YNR012W
YDR021WYJR007W
YDR021WYML130C
YDR021WYNL271C
YDR022C YGL070C
YDR022C YGL180W
YDR022C YGL237C
YDR022C YLR288C
YDR022C YLR423C
YDR023WYDR034C
YDR023WYDR388W
YDR023WYLR442C
YDR023WYMR059W
YDR023WYOL087C
YDR023WYOL123W
YDR023WYPL204W
YDR024WYMR109W
YDR026C YDR110W
YDR026C YKR092C
YDR027C YDR468C
YDR027C YDR484W
YDR027C YER114C
YDR027C YFR024C-A
YDR027C YJL029C
YDR027C YKR020W
YDR027C YLR262C
YDR028C YDR477W
YDR028C YER133W
YDR028C YER177W
YDR028C YGL115W
YDR028C YKL193C
YDR028C YOR267C
YDR030C YDR188W
YDR030C YDR212W
YDR030C YIL142W
YDR030C YJL008C
YDR030C YJL111W
YDR030C YLR259C
YDR030C YNL124W
YDR030C YOL055C
YDR030C YPL258C
YDR031WYJR091C
YDR032C YDR369C
YDR032C YFR040W
YDR032C YGL115W

YDR032C YHR135C
YDR032C YML064C
YDR032C YMR106C
YDR034C YDR131C
YDR034C YDR259C
YDR034C YDR277C
YDR034C YDR342C
YDR034C YDR438W
YDR034C YDR449C
YDR034C YDR456W
YDR034C YER038C
YDR034C YER043C
YDR034C YER044C
YDR034C YER064C
YDR034C YFR021W
YDR034C YFR046C
YDR034C YFR055W
YDR034C YGL125W
YDR034C YGL169W
YDR034C YGL181W
YDR034C YGL223C
YDR034C YGR111W
YDR034C YGR113W
YDR034C YGR124W
YDR034C YGR189C
YDR034C YGR295C
YDR034C YHL004W
YDR034C YHR060W
YDR034C YIL045W
YDR034C YIL145C
YDR034C YIR026C
YDR034C YJL002C
YDR034C YJL185C
YDR034C YJR075W
YDR034C YKL068W
YDR034C YKR048C
YDR034C YLR063W
YDR034C YLR098C
YDR034C YLR105C
YDR034C YLR191W
YDR034C YLR328W
YDR034C YML116W
YDR034C YMR064W
YDR034C YMR094W
YDR034C YMR187C

YDR034C YOR009W
YDR034C YPL102C
YDR034C YPL114W
YDR034C YPL138C
YDR034C YPL178W
YDR034C YPR136C
YDR034C YPR187W
YDR036C YDR041W
YDR036C YDR175C
YDR036C YDR337W
YDR036C YDR347W
YDR036C YGL129C
YDR036C YGR084C
YDR036C YHL004W
YDR036C YIL093C
YDR036C YJR113C
YDR036C YKL155C
YDR036C YLR074C
YDR036C YMR188C
YDR036C YNL137C
YDR036C YNL306W
YDR036C YPL118W
YDR037WYDR388W
YDR037WYJL124C
YDR037WYNL135C
YDR037WYPR056W
YDR038C YDR380W
YDR038C YNL217W
YDR038C YOR016C
YDR039C YPR201W
YDR040C YMR047C
YDR041WYHL004W
YDR042C YHR114W
YDR042C YLR413W
YDR043C YDR477W
YDR044WYDR077W
YDR044WYGL112C
YDR044WYNL189W
YDR045C YLR277C
YDR045C YNR003C
YDR045C YOR116C
YDR045C YOR207C
YDR046C YGR060W
YDR046C YIL005W
YDR046C YJR010C-A

YDR046C YLR372W
YDR050C YNL127W
YDR052C YLR423C
YDR052C YMR001C
YDR052C YOR178C
YDR052C YPL153C
YDR054C YDR328C
YDR054C YFL009W
YDR054C YGL070C
YDR054C YIL148W
YDR054C YJL047C
YDR054C YNL236W
YDR054C YOL133W
YDR055WYMR205C
YDR056C YGL070C
YDR056C YHR114W
YDR060WYDR382W
YDR060WYEL026W
YDR060WYGR103W
YDR060WYGR281W
YDR060WYHR052W
YDR060WYHR066W
YDR060WYJL109C
YDR060WYKR081C
YDR060WYLR221C
YDR060WYLR276C
YDR060WYML124C
YDR060WYMR049C
YDR060WYMR290C
YDR060WYNL002C
YDR060WYNL061W
YDR060WYNL110C
YDR060WYNL118C
YDR060WYOL041C
YDR060WYOL077C
YDR060WYOR206W
YDR060WYOR272W
YDR060WYPL012W
YDR060WYPL024W
YDR060WYPL043W
YDR060WYPL141C
YDR060WYPL211W
YDR061WYOR043W
YDR062WYDR099W
YDR062WYDR502C

YDR062WYEL022W
YDR062WYER110C
YDR062WYER177W
YDR062WYGR038W
YDR062WYGR218W
YDR062WYHL030W
YDR062WYHR020W
YDR062WYIL094C
YDR062WYJR077C
YDR062WYKL104C
YDR062WYLR180W
YDR062WYLR342W
YDR062WYLR350W
YDR062WYMR296C
YDR063WYGL239C
YDR063WYKL002W
YDR063WYKL013C
YDR066C YJR082C
YDR067C YGL070C
YDR068WYER103W
YDR068WYLR237W
YDR069C YER022W
YDR069C YHR114W
YDR069C YLR073C
YDR070C YFL017C
YDR070C YHR178W
YDR071C YDR247W
YDR071C YER089C
YDR071C YPL153C
YDR072C YDR107C
YDR073WYML068W
YDR073WYNL189W
YDR073WYOR290C
YDR074WYER019C-A
YDR074WYER178W
YDR074WYHR196W
YDR074WYLR447C
YDR074WYML100W
YDR074WYMR261C
YDR075WYDR212W
YDR075WYER178W
YDR075WYFL037W
YDR075WYGL173C
YDR075WYGL206C
YDR075WYGR192C

YDR075WYHR011W
YDR075WYHR033W
YDR075WYHR082C
YDR075WYHR084W
YDR075WYIL142W
YDR075WYJL014W
YDR075WYJL130C
YDR075WYJR121W
YDR075WYLR259C
YDR075WYML085C
YDR075WYMR205C
YDR075WYNL201C
YDR075WYOL055C
YDR075WYOR151C
YDR075WYPL235W
YDR075WYPL258C
YDR075WYPR040W
YDR076WYER095W
YDR076WYGR282C
YDR076WYHR033W
YDR076WYJL008C
YDR076WYLR259C
YDR078C YHL006C
YDR078C YIL152W
YDR078C YJL092W
YDR078C YLR376C
YDR079C-A YPL122C
YDR079WYGL070C
YDR079WYJL099W
YDR079WYJR063W
YDR080WYGR013W
YDR080WYLR148W
YDR080WYLR248W
YDR080WYLR396C
YDR080WYMR231W
YDR080WYPL045W
YDR080WYPL195W
YDR082WYER112W
YDR082WYLR010C
YDR082WYPL153C
YDR084C YDR441C
YDR084C YDR442W
YDR084C YEL054C
YDR084C YER011W
YDR084C YGL044C

YDR084C YGL127C
YDR084C YGL161C
YDR084C YGL198W
YDR084C YKL192C
YDR085C YER114C
YDR085C YER133W
YDR085C YHR107C
YDR085C YMR059W
YDR085C YPL242C
YDR086C YGR175C
YDR086C YLR378C
YDR086C YPL189W
YDR087C YER133W
YDR087C YGR103W
YDR087C YIL131C
YDR087C YJL095W
YDR087C YKR081C
YDR087C YLR233C
YDR087C YLR447C
YDR087C YNL061W
YDR087C YOR061W
YDR087C YOR267C
YDR087C YPL141C
YDR088C YER013W
YDR088C YER172C
YDR088C YGR006W
YDR088C YGR074W
YDR088C YGR232W
YDR091C YER161C
YDR091C YGL185C
YDR091C YHR195W
YDR091C YLR192C
YDR091C YMR047C
YDR091C YPR041W
YDR092WYGL087C
YDR092WYGL127C
YDR092WYGL206C
YDR092WYHR137W
YDR092WYJL088W
YDR092WYKL210W
YDR092WYLR032W
YDR092WYLR059C
YDR092WYLR180W
YDR092WYLR259C
YDR092WYNL064C

YDR092WYOL055C
YDR092WYPL258C
YDR093WYMR054W
YDR095C YGL127C
YDR095C YIL046W
YDR096WYPR154W
YDR097C YDR499W
YDR097C YER177W
YDR097C YFR037C
YDR097C YIR002C
YDR097C YJL173C
YDR097C YJR144W
YDR097C YKR001C
YDR097C YLR234W
YDR097C YML032C
YDR097C YMR190C
YDR097C YNL312W
YDR097C YOL090W
YDR097C YPL235W
YDR098C YGL044C
YDR098C YGL071W
YDR098C YGL220W
YDR098C YGR262C
YDR099WYDR129C
YDR099WYDR130C
YDR099WYDR227W
YDR099WYDR306C
YDR099WYDR309C
YDR099WYDR316W
YDR099WYDR328C
YDR099WYDR480W
YDR099WYER054C
YDR099WYER075C
YDR099WYER133W
YDR099WYER173W
YDR099WYER177W
YDR099WYFR028C
YDR099WYGL003C
YDR099WYGL115W
YDR099WYGL137W
YDR099WYGL163C
YDR099WYGL252C
YDR099WYGR123C
YDR099WYGR214W
YDR099WYHL007C

YDR099WYIL035C
YDR099WYIL095W
YDR099WYIL147C
YDR099WYJL042W
YDR099WYJL098W
YDR099WYJR035W
YDR099WYKR055W
YDR099WYLR177W
YDR099WYLR267W
YDR099WYLR291C
YDR099WYLR453C
YDR099WYML064C
YDR099WYMR059W
YDR099WYMR137C
YDR099WYMR199W
YDR099WYNL042W
YDR099WYNL088W
YDR099WYNL180C
YDR099WYNL199C
YDR099WYNL244C
YDR099WYNL267W
YDR099WYNL293W
YDR099WYNL307C
YDR099WYNR031C
YDR099WYOL062C
YDR099WYOL133W
YDR099WYOR026W
YDR099WYOR089C
YDR099WYOR298C-A
YDR099WYPL204W
YDR099WYPL259C
YDR099WYPR030W
YDR100WYFR024C-A
YDR100WYGL161C
YDR100WYHR016C
YDR100WYIL004C
YDR100WYNL189W
YDR100WYOR036W
YDR100WYPL020C
YDR100WYPL095C
YDR101C YDR192C
YDR101C YER036C
YDR101C YER126C
YDR101C YGL099W
YDR101C YGR245C

YDR101C YHR170W
YDR101C YHR197W
YDR101C YIL018W
YDR101C YKL068W
YDR101C YKR081C
YDR101C YLR074C
YDR101C YNL110C
YDR101C YPR016C
YDR101C YPR017C
YDR102C YDR365C
YDR102C YGR218W
YDR102C YPR086W
YDR103WYDR264C
YDR103WYER132C
YDR103WYGR040W
YDR103WYGR179C
YDR103WYLR362W
YDR103WYOR212W
YDR104C YMR032W
YDR105C YER081W
YDR105C YNL160W
YDR106WYHR129C
YDR106WYLR222C
YDR107C YGL200C
YDR107C YOL015W
YDR108WYGR234W
YDR108WYIR040C
YDR108WYKR068C
YDR110WYDR235W
YDR110WYER146W
YDR110WYER161C
YDR110WYGL127C
YDR110WYHL004W
YDR110WYIL061C
YDR110WYLR095C
YDR110WYML064C
YDR110WYPL153C
YDR111C YMR304W
YDR113C YFL039C
YDR113C YGL116W
YDR113C YGR060W
YDR113C YGR098C
YDR113C YJR045C
YDR113C YKL035W
YDR113C YNL189W

YDR115WYGL070C
YDR115WYGR220C
YDR115WYKL142W
YDR115WYNL041C
YDR115WYOR264W
YDR116C YDR309C
YDR116C YGR220C
YDR116C YIL035C
YDR116C YJL041W
YDR116C YNL185C
YDR116C YNL284C
YDR118WYFR036W
YDR118WYGL240W
YDR118WYHR166C
YDR118WYLR102C
YDR118WYLR127C
YDR118WYMR001C
YDR118WYNL172W
YDR118WYOR249C
YDR119WYGR041W
YDR119WYHR078W
YDR119WYKL188C
YDR119WYPR058W
YDR120C YEL017W
YDR120C YIR038C
YDR120C YLR035C
YDR121WYLR447C
YDR121WYNL262W
YDR121WYPR175W
YDR122WYER133W
YDR122WYER167W
YDR122WYFL034C-B
YDR122WYGR097W
YDR122WYGR238C
YDR122WYHR158C
YDR122WYJL187C
YDR122WYKL048C
YDR122WYLR310C
YDR122WYML006C
YDR122WYMR124W
YDR122WYNL035C
YDR122WYNL161W
YDR122WYOL054W
YDR122WYOL082W
YDR122WYOL083W

YDR122WYPR115W
YDR122WYPR120C
YDR123C YHL020C
YDR123C YOL108C
YDR126WYEL002C
YDR126WYNR051C
YDR127WYDR260C
YDR127WYDR398W
YDR127WYDR427W
YDR127WYER095W
YDR127WYFR024C-A
YDR127WYGL048C
YDR127WYGR234W
YDR127WYGR240C
YDR127WYHR082C
YDR127WYIL106W
YDR127WYJL008C
YDR127WYJL173C
YDR127WYJR022W
YDR127WYKL104C
YDR127WYKR026C
YDR127WYMR106C
YDR127WYMR117C
YDR127WYMR186W
YDR127WYOR136W
YDR127WYOR351C
YDR127WYPL235W
YDR128WYDR171W
YDR128WYDR188W
YDR128WYDR394W
YDR128WYDR477W
YDR128WYEL062W
YDR128WYER103W
YDR128WYER182W
YDR128WYFL016C
YDR128WYGL100W
YDR128WYGL205W
YDR128WYGR254W
YDR128WYHR033W
YDR128WYJL008C
YDR128WYJL052W
YDR128WYJL088W
YDR128WYJL173C
YDR128WYJR045C
YDR128WYKL104C

YDR128WYLR096W
YDR128WYLR208W
YDR128WYLR216C
YDR128WYLR259C
YDR128WYMR066W
YDR128WYMR106C
YDR128WYMR108W
YDR128WYNL244C
YDR128WYNL307C
YDR128WYOL139C
YDR128WYOR133W
YDR128WYOR374W
YDR128WYPL240C
YDR128WYPL258C
YDR129C YDR353W
YDR129C YFL039C
YDR129C YGR254W
YDR129C YJL052W
YDR129C YKL060C
YDR129C YKL085W
YDR129C YMR246W
YDR129C YNL135C
YDR129C YOR181W
YDR130C YER032W
YDR130C YER133W
YDR130C YER177W
YDR130C YHR134W
YDR130C YPR120C
YDR131C YDR328C
YDR131C YHR197W
YDR131C YIL063C
YDR131C YJR121W
YDR131C YLR180W
YDR131C YLR259C
YDR131C YML124C
YDR131C YNL064C
YDR131C YOL055C
YDR131C YOR374W
YDR131C YPL258C
YDR131C YPR093C
YDR132C YDR395W
YDR132C YHR170W
YDR132C YJL218W
YDR132C YOR098C
YDR133C YJR103W

YDR133C YPR086W
YDR135C YJR022W
YDR136C YGL070C
YDR136C YNL166C
YDR136C YOL091W
YDR137WYLR039C
YDR137WYLR262C
YDR138WYDR214W
YDR138WYEL060C
YDR138WYFL039C
YDR138WYGL048C
YDR138WYLR259C
YDR138WYML062C
YDR138WYML085C
YDR138WYNL139C
YDR138WYNL189W
YDR138WYNR031C
YDR139C YDR328C
YDR139C YGR003W
YDR139C YIL046W
YDR139C YJL047C
YDR139C YLR306W
YDR139C YOL133W
YDR140WYIL151C
YDR140WYNR046W
YDR141C YPL115C
YDR141C YPR110C
YDR141C YPR154W
YDR142C YDR171W
YDR142C YDR188W
YDR142C YDR244W
YDR142C YER081W
YDR142C YFL016C
YDR142C YFL037W
YDR142C YFL039C
YDR142C YGL153W
YDR142C YGR184C
YDR142C YGR239C
YDR142C YHR160C
YDR142C YIL068C
YDR142C YIL142W
YDR142C YIL160C
YDR142C YJL014W
YDR142C YJL052W
YDR142C YJL066C

YDR142C YJL111W
YDR142C YJL130C
YDR142C YJR045C
YDR142C YJR064W
YDR142C YJR077C
YDR142C YJR121W
YDR142C YJR139C
YDR142C YKL060C
YDR142C YKL103C
YDR142C YLR105C
YDR142C YLR191W
YDR142C YLR259C
YDR142C YLR304C
YDR142C YLR355C
YDR142C YML085C
YDR142C YMR066W
YDR142C YMR108W
YDR142C YMR186W
YDR142C YNL064C
YDR142C YNL071W
YDR142C YNL214W
YDR142C YOL055C
YDR142C YOR326W
YDR142C YPL226W
YDR143C YIL148W
YDR143C YJL008C
YDR143C YJR045C
YDR143C YJR121W
YDR143C YLR229C
YDR143C YML085C
YDR143C YNL189W
YDR143C YOL094C
YDR143C YOR151C
YDR143C YPL113C
YDR143C YPR010C
YDR143C YPR019W
YDR144C YLR453C
YDR145WYDR167W
YDR145WYER148W
YDR145WYGL112C
YDR145WYGR274C
YDR145WYHR099W
YDR145WYLR390W
YDR145WYLR390W-A
YDR145WYML098W

YDR145WYMR005W
YDR145WYOR119C
YDR145WYPR072W
YDR146C YDR335W
YDR146C YER015W
YDR146C YFL009W
YDR146C YGR002C
YDR146C YGR089W
YDR146C YHR099W
YDR146C YIL148W
YDR146C YJL081C
YDR146C YMR019W
YDR146C YOL055C
YDR146C YOR116C
YDR146C YPL121C
YDR146C YPL254W
YDR148C YDR388W
YDR148C YDR510W
YDR148C YER110C
YDR148C YFL018C
YDR148C YFR016C
YDR148C YFR049W
YDR148C YGL208W
YDR148C YGR040W
YDR148C YGR092W
YDR148C YGR262C
YDR148C YHR030C
YDR148C YHR114W
YDR148C YIL095W
YDR148C YIL125W
YDR148C YJL092W
YDR148C YJR035W
YDR148C YKL010C
YDR148C YKL103C
YDR148C YLR180W
YDR148C YLR222C
YDR148C YLR410W-B
YDR148C YLR423C
YDR148C YMR012W
YDR148C YMR047C
YDR148C YMR104C
YDR148C YMR308C
YDR148C YNL092W
YDR148C YNL189W
YDR148C YOL133W

YDR148C YPL070W
YDR148C YPR110C
YDR150WYDR201W
YDR150WYDR247W
YDR150WYDR295C
YDR150WYER120W
YDR150WYGL158W
YDR150WYGR089W
YDR150WYJL074C
YDR150WYJR112W
YDR150WYOL069W
YDR150WYOR014W
YDR152WYGR173W
YDR152WYJR091C
YDR155C YDR510W
YDR155C YER081W
YDR155C YFL014W
YDR155C YGL194C
YDR155C YIL112W
YDR155C YIR005W
YDR155C YJL020C
YDR155C YJR090C
YDR155C YKL103C
YDR155C YKR105C
YDR155C YLR074C
YDR155C YML064C
YDR155C YML095C
YDR155C YMR036C
YDR155C YMR059W
YDR155C YNL030W
YDR155C YNL094W
YDR155C YNL157W
YDR155C YNL244C
YDR155C YOL068C
YDR155C YOR290C
YDR155C YOR319W
YDR155C YPL235W
YDR156WYJR063W
YDR156WYJR091C
YDR158WYLR314C
YDR159WYER118C
YDR159WYJL041W
YDR160WYLR310C
YDR162C YDR243C
YDR162C YER114C

YDR162C YGR058W
YDR162C YGR099W
YDR162C YGR162W
YDR162C YHL007C
YDR162C YIL156W
YDR162C YIR003W
YDR162C YJL095W
YDR162C YJL128C
YDR162C YKL192C
YDR162C YKR048C
YDR162C YLR241W
YDR162C YLR425W
YDR162C YMR031W-A
YDR162C YNL093W
YDR162C YNL094W
YDR162C YNL298W
YDR162C YNR064C
YDR162C YOL093W
YDR162C YOL113W
YDR162C YPR008W
YDR164C YGR009C
YDR164C YKR014C
YDR164C YNR049C
YDR164C YOL086C
YDR164C YPL232W
YDR165WYJL052W
YDR166C YER008C
YDR167WYDR207C
YDR167WYDR216W
YDR167WYDR259C
YDR167WYDR448W
YDR167WYER013W
YDR167WYFL033C
YDR167WYGL112C
YDR167WYGR097W
YDR167WYGR252W
YDR167WYGR274C
YDR167WYHR099W
YDR167WYIL129C
YDR167WYJR091C
YDR167WYKL012W
YDR167WYLR228C
YDR167WYLR432W
YDR167WYML015C
YDR167WYML098W

YDR167WYML114C
YDR167WYMR236W
YDR167WYNL216W
YDR167WYOL148C
YDR167WYOR119C
YDR167WYOR151C
YDR167WYOR250C
YDR167WYPL011C
YDR167WYPL089C
YDR167WYPL129W
YDR167WYPR115W
YDR168WYJR032W
YDR168WYKL139W
YDR168WYOR027W
YDR168WYPL240C
YDR169C YML112W
YDR169C YOL004W
YDR170C YDR477W
YDR170C YER100W
YDR170C YER110C
YDR170C YER125W
YDR170C YFR009W
YDR170C YFR044C
YDR170C YGL238W
YDR170C YHR020W
YDR170C YIL109C
YDR170C YJL203W
YDR170C YJR077C
YDR170C YKL104C
YDR170C YKR021W
YDR170C YLR148W
YDR170C YLR180W
YDR170C YLR263W
YDR170C YLR342W
YDR170C YNL287W
YDR170C YOL086C
YDR170C YOR061W
YDR170C YOR136W
YDR170C YPR181C
YDR170W-A YMR139W
YDR171WYDR267C
YDR171WYDR398W
YDR171WYDR430C
YDR171WYDR488C
YDR171WYDR523C

YDR171WYER066C-A
YDR171WYFR016C
YDR171WYFR028C
YDR171WYGL137W
YDR171WYGL158W
YDR171WYGL190C
YDR171WYGR040W
YDR171WYGR262C
YDR171WYHR030C
YDR171WYHR186C
YDR171WYJL020C
YDR171WYJL106W
YDR171WYJR076C
YDR171WYKL078W
YDR171WYKR026C
YDR171WYKR036C
YDR171WYLR016C
YDR171WYLR096W
YDR171WYLR175W
YDR171WYLR229C
YDR171WYLR288C
YDR171WYLR291C
YDR171WYML065W
YDR171WYML115C
YDR171WYMR049C
YDR171WYMR055C
YDR171WYMR106C
YDR171WYMR284W
YDR171WYNL007C
YDR171WYNL216W
YDR171WYNL236W
YDR171WYNR031C
YDR171WYOL062C
YDR171WYOR181W
YDR171WYOR229W
YDR171WYOR351C
YDR171WYPL150W
YDR171WYPL259C
YDR171WYPR054W
YDR171WYPR178W
YDR172WYDR300C
YDR172WYHR015W
YDR172WYLL026W
YDR172WYML117W
YDR172WYMR080C

YDR172WYNL251C
YDR172WYOR069W
YDR172WYOR150W
YDR172WYOR297C
YDR172WYPL190C
YDR172WYPL237W
YDR173C YMR042W
YDR173C YMR043W
YDR174WYDR335W
YDR174WYHL002W
YDR174WYIL013C
YDR174WYKL130C
YDR174WYKR092C
YDR174WYLR074C
YDR174WYML015C
YDR174WYML064C
YDR174WYML098W
YDR174WYNL135C
YDR174WYNL189W
YDR174WYPR104C
YDR175C YJR091C
YDR175C YLR074C
YDR176WYDR308C
YDR176WYDR448W
YDR176WYDR457W
YDR176WYEL009C
YDR176WYER022W
YDR176WYER148W
YDR176WYGL013C
YDR176WYGL112C
YDR176WYGR274C
YDR176WYHR041C
YDR176WYHR099W
YDR176WYIL144W
YDR176WYJR080C
YDR176WYLR423C
YDR176WYMR236W
YDR176WYOL148C
YDR176WYOR119C
YDR176WYPL181W
YDR176WYPL254W
YDR176WYPR086W
YDR177WYDR226W
YDR177WYKL168C
YDR178WYJR063W

YDR178WYMR032W
YDR178WYPL132W
YDR179C YHR005C
YDR179C YJR015W
YDR179C YKR060W
YDR179C YMR025W
YDR180WYER147C
YDR180WYIL026C
YDR181C YJL115W
YDR181C YMR127C
YDR181C YOR213C
YDR181C YPR018W
YDR183WYGL044C
YDR183WYOR212W
YDR184C YGL070C
YDR184C YIR009W
YDR184C YJR022W
YDR184C YLR319C
YDR188WYER082C
YDR188WYGL137W
YDR188WYGL190C
YDR188WYKR036C
YDR188WYLL011W
YDR188WYLR196W
YDR188WYMR028W
YDR188WYMR049C
YDR188WYNL317W
YDR188WYOR212W
YDR188WYOR229W
YDR188WYOR230W
YDR188WYPL151C
YDR189WYIL004C
YDR189WYKL196C
YDR189WYLR026C
YDR189WYLR078C
YDR189WYLR268W
YDR189WYMR316W
YDR189WYNL239W
YDR189WYOR075W
YDR190C YDR334W
YDR190C YDR398W
YDR190C YER082C
YDR190C YER100W
YDR190C YER179W
YDR190C YGL004C

YDR190C YGR002C
YDR190C YGR040W
YDR190C YIL147C
YDR190C YKL193C
YDR190C YKR026C
YDR190C YLR291C
YDR190C YML064C
YDR190C YML112W
YDR190C YMR284W
YDR190C YNL107W
YDR190C YNL311C
YDR190C YOL012C
YDR190C YOR229W
YDR190C YPL140C
YDR190C YPL235W
YDR192C YDR335W
YDR192C YER081W
YDR192C YER107C
YDR192C YER110C
YDR192C YER165W
YDR192C YGL092W
YDR192C YGL122C
YDR192C YGR218W
YDR192C YJR042W
YDR192C YJR069C
YDR192C YKL057C
YDR192C YKR082W
YDR192C YLR335W
YDR192C YLR347C
YDR192C YMR235C
YDR192C YMR255W
YDR192C YNL049C
YDR192C YNL092W
YDR192C YNL189W
YDR192C YOR134W
YDR192C YOR160W
YDR192C YOR204W
YDR192C YPL093W
YDR192C YPR181C
YDR194C YDR365C
YDR194C YER081W
YDR194C YGL035C
YDR194C YHR052W
YDR194C YLR233C
YDR194C YNL175C

YDR194C YNL230C
YDR195WYDR301W
YDR195WYER032W
YDR195WYER133W
YDR195WYGR156W
YDR195WYJL033W
YDR195WYKL018W
YDR195WYKL059C
YDR195WYKR002W
YDR195WYLR115W
YDR195WYLR277C
YDR195WYMR061W
YDR195WYNL317W
YDR195WYOR179C
YDR195WYPR107C
YDR196C YJL194W
YDR196C YJR063W
YDR198C YOR005C
YDR200C YFL016C
YDR200C YFL037W
YDR200C YFR008W
YDR200C YGL206C
YDR200C YGR086C
YDR200C YJL008C
YDR200C YJL130C
YDR200C YJR045C
YDR200C YJR121W
YDR200C YKL129C
YDR200C YKL152C
YDR200C YLR238W
YDR200C YML085C
YDR200C YML123C
YDR200C YMR029C
YDR200C YMR052W
YDR200C YMR109W
YDR200C YMR319C
YDR200C YNL064C
YDR200C YNL127W
YDR200C YOR374W
YDR200C YPL004C
YDR201WYDR507C
YDR201WYEL043W
YDR201WYFL008W
YDR201WYHR083W
YDR201WYIL144W

YDR201WYKL179C
YDR201WYKR037C
YDR201WYKR083C
YDR201WYNL084C
YDR201WYNL189W
YDR201WYOL069W
YDR202C YDR328C
YDR202C YEL051W
YDR202C YJR033C
YDR202C YOR332W
YDR205WYKL221W
YDR205WYNR039C
YDR206WYER027C
YDR206WYHL007C
YDR206WYHR030C
YDR206WYIL106W
YDR206WYJL030W
YDR206WYJL057C
YDR206WYLR362W
YDR206WYOL149W
YDR206WYOR047C
YDR206WYPR106W
YDR207C YJR094C
YDR207C YML099C
YDR207C YMR042W
YDR207C YMR047C
YDR207C YMR139W
YDR207C YNL153C
YDR207C YNL307C
YDR207C YOL004W
YDR207C YOL082W
YDR207C YOR355W
YDR211WYER025W
YDR211WYGR052W
YDR211WYGR083C
YDR211WYJL080C
YDR211WYJR007W
YDR211WYJR090C
YDR211WYKR026C
YDR211WYLR291C
YDR211WYNL265C
YDR211WYOL128C
YDR211WYOR260W
YDR211WYPL140C
YDR211WYPL237W

YDR211WYPR161C
YDR212WYER173W
YDR212WYGL116W
YDR212WYGL137W
YDR212WYGL190C
YDR212WYGL208W
YDR212WYIL142W
YDR212WYJL106W
YDR212WYJL164C
YDR212WYKL095W
YDR212WYKR036C
YDR212WYLL011W
YDR212WYLL019C
YDR212WYLR030W
YDR212WYLR196W
YDR212WYNL207W
YDR212WYNL317W
YDR212WYNL323W
YDR212WYOR212W
YDR212WYOR230W
YDR212WYOR324C
YDR212WYPL180W
YDR213WYHR165C
YDR214WYDR523C
YDR214WYER173W
YDR214WYHR030C
YDR214WYIL038C
YDR214WYJL030W
YDR214WYOL052C
YDR214WYOL094C
YDR214WYOL139C
YDR214WYOR212W
YDR214WYPR110C
YDR215C YKR024C
YDR215C YPL175W
YDR216WYDR448W
YDR216WYER095W
YDR216WYER118C
YDR216WYER148W
YDR216WYER177W
YDR216WYGL112C
YDR216WYGR052W
YDR216WYGR123C
YDR216WYGR252W
YDR216WYGR274C

YDR216WYHR099W
YDR216WYJL076W
YDR216WYLR055C
YDR216WYMR106C
YDR216WYNL025C
YDR216WYOL001W
YDR216WYOL133W
YDR216WYOL148C
YDR216WYOR208W
YDR216WYPR086W
YDR216WYPR110C
YDR217C YFL039C
YDR217C YGL158W
YDR217C YHR031C
YDR217C YKL152C
YDR217C YMR001C
YDR217C YOL055C
YDR217C YOR374W
YDR217C YPL153C
YDR218C YJR076C
YDR218C YPL161C
YDR219C YDR328C
YDR219C YFL039C
YDR219C YHR122W
YDR219C YJR045C
YDR219C YLR259C
YDR221WYMR151W
YDR224C YDR225W
YDR224C YDR334W
YDR224C YDR365C
YDR224C YDR510W
YDR224C YER082C
YDR224C YGL058W
YDR224C YIL066C
YDR224C YIL131C
YDR224C YJL081C
YDR224C YKL103C
YDR224C YLR074C
YDR224C YLR442C
YDR224C YNL030W
YDR224C YNL312W
YDR224C YOL054W
YDR224C YOL108C
YDR224C YPR041W
YDR225WYDR243C

YDR225WYDR485C
YDR225WYER022W
YDR225WYGL127C
YDR225WYGL173C
YDR225WYGL207W
YDR225WYGL241W
YDR225WYGR002C
YDR225WYGR103W
YDR225WYHL001W
YDR225WYHL034C
YDR225WYIL035C
YDR225WYJL034W
YDR225WYJL081C
YDR225WYKR048C
YDR225WYLR074C
YDR225WYLR222C
YDR225WYLR247C
YDR225WYLR385C
YDR225WYLR399C
YDR225WYLR442C
YDR225WYMR061W
YDR225WYMR125W
YDR225WYNL030W
YDR225WYNL308C
YDR225WYOL054W
YDR225WYOR005C
YDR225WYOR038C
YDR225WYOR116C
YDR225WYOR207C
YDR225WYPL043W
YDR225WYPR104C
YDR225WYPR190C
YDR226WYFR028C
YDR226WYJR091C
YDR226WYML064C
YDR226WYOR176W
YDR226WYPL031C
YDR227WYER151C
YDR227WYER177W
YDR227WYFL007W
YDR227WYFL037W
YDR227WYFL045C
YDR227WYFR053C
YDR227WYJL088W
YDR227WYJR045C

YDR227WYKL152C
YDR227WYKR101W
YDR227WYLR259C
YDR227WYLR442C
YDR227WYML085C
YDR227WYML109W
YDR227WYML124C
YDR227WYMR205C
YDR227WYMR219W
YDR227WYMR284W
YDR227WYNL030W
YDR227WYNL071W
YDR227WYNL189W
YDR227WYNL216W
YDR227WYOL055C
YDR227WYOR191W
YDR227WYPL258C
YDR228C YDR301W
YDR228C YDR448W
YDR228C YER118C
YDR228C YER165W
YDR228C YER177W
YDR228C YGL044C
YDR228C YIL035C
YDR228C YJR022W
YDR228C YJR045C
YDR228C YKL059C
YDR228C YKR002W
YDR228C YLR115W
YDR228C YLR259C
YDR228C YLR277C
YDR228C YLR423C
YDR228C YLR424W
YDR228C YMR061W
YDR228C YOR151C
YDR228C YOR250C
YDR228C YPL031C
YDR228C YPR049C
YDR229WYDR388W
YDR229WYGL066W
YDR229WYGR218W
YDR229WYHR102W
YDR229WYJL016W
YDR229WYMR001C
YDR229WYMR047C

YDR229WYNL041C
YDR229WYNL053W
YDR229WYPR119W
YDR233C YEL056W
YDR233C YOR165W
YDR233C YOR285W
YDR235WYGR013W
YDR235WYHR086W
YDR235WYIL061C
YDR235WYJR131W
YDR235WYLR298C
YDR235WYMR125W
YDR235WYNL094W
YDR235WYPR182W
YDR236C YDR398W
YDR237WYGR220C
YDR237WYNL185C
YDR237WYNL284C
YDR238C YER112W
YDR238C YER122C
YDR238C YFR051C
YDR238C YGL137W
YDR238C YHR169W
YDR238C YIL004C
YDR238C YIL106W
YDR238C YLR078C
YDR238C YLR268W
YDR238C YNL284C
YDR239C YDR388W
YDR239C YER114C
YDR239C YFR028C
YDR239C YGR040W
YDR239C YGR136W
YDR239C YLR096W
YDR240C YFL017W-A
YDR240C YGR013W
YDR240C YGR074W
YDR240C YHR086W
YDR240C YIL061C
YDR240C YLR298C
YDR240C YNL189W
YDR240C YPL178W
YDR243C YDR260C
YDR243C YLR183C
YDR243C YOR125C

YDR243C YPL235W
YDR244WYGL153W
YDR244WYGL205W
YDR244WYGR077C
YDR244WYLR191W
YDR244WYML042W
YDR244WYNL214W
YDR245WYDR414C
YDR245WYEL036C
YDR245WYJL183W
YDR245WYJR075W
YDR245WYPL094C
YDR246WYKR068C
YDR247WYDR416W
YDR247WYDR439W
YDR247WYER133W
YDR247WYER183C
YDR247WYFR028C
YDR247WYGL207W
YDR247WYGR097W
YDR247WYGR200C
YDR247WYGR237C
YDR247WYIL070C
YDR247WYIL154C
YDR247WYJL005W
YDR247WYJL076W
YDR247WYKR010C
YDR247WYLR384C
YDR247WYMR270C
YDR247WYNL210W
YDR247WYNL295W
YDR247WYOL010W
YDR247WYOL116W
YDR247WYOR028C
YDR247WYPL086C
YDR247WYPL217C
YDR247WYPR010C-A
YDR247WYPR115W
YDR250C YJL070C
YDR251WYDR466W
YDR251WYGL206C
YDR251WYJL095W
YDR251WYPL057C
YDR252WYGR134W
YDR252WYHR193C

YDR252WYJR055W
YDR253C YNL103W
YDR254WYEL077C
YDR254WYIL111W
YDR255C YKL144C
YDR256C YGL153W
YDR256C YLR347C
YDR256C YMR314W
YDR256C YNL189W
YDR259C YDR311W
YDR259C YDR484W
YDR259C YER022W
YDR259C YER032W
YDR259C YER114C
YDR259C YFR015C
YDR259C YGL127C
YDR259C YGL181W
YDR259C YGR066C
YDR259C YGR239C
YDR259C YHL002W
YDR259C YKR048C
YDR259C YLR423C
YDR259C YLR446W
YDR259C YLR447C
YDR259C YNL020C
YDR259C YNL092W
YDR259C YNR051C
YDR259C YNR052C
YDR260C YEL060C
YDR260C YGL070C
YDR260C YGL206C
YDR260C YHR166C
YDR260C YJL008C
YDR260C YJR045C
YDR260C YKL152C
YDR260C YLR116W
YDR260C YLR180W
YDR260C YML072C
YDR260C YMR205C
YDR260C YMR214W
YDR260C YNL064C
YDR260C YOR303W
YDR261W-A YDR261W-B
YDR261W-A YFL002W-B
YDR262WYDR454C

YDR263C YOR214C
YDR264C YHR135C
YDR264C YJR086W
YDR264C YKL178C
YDR264C YNL154C
YDR264C YOR043W
YDR264C YOR101W
YDR264C YOR212W
YDR264C YPL242C
YDR265WYGR133W
YDR265WYLR191W
YDR265WYMR026C
YDR265WYOL044W
YDR266C YER081W
YDR266C YGL173C
YDR266C YHL034C
YDR266C YJL066C
YDR266C YJR045C
YDR266C YLR180W
YDR266C YMR012W
YDR266C YOR232W
YDR267C YER098W
YDR267C YFL016C
YDR267C YHR044C
YDR267C YHR122W
YDR267C YHR152W
YDR267C YIL128W
YDR267C YJL052W
YDR267C YJL153C
YDR267C YKL152C
YDR267C YLR044C
YDR267C YMR066W
YDR267C YMR108W
YDR267C YOL111C
YDR267C YOR261C
YDR267C YOR374W
YDR268WYJR091C
YDR268WYLR453C
YDR268WYOR308C
YDR269C YJR091C
YDR270WYHR016C
YDR270WYHR114W
YDR270WYNL259C
YDR271C YDR509W
YDR271C YFR024C-A

YDR271C YHR114W
YDR271C YJR091C
YDR271C YMR047C
YDR271C YOR128C
YDR271C YOR244W
YDR271C YPR086W
YDR273WYFR052W
YDR273WYMR001C
YDR275WYDR363W
YDR276C YDR307W
YDR276C YDR331W
YDR276C YEL002C
YDR276C YJL117W
YDR276C YJR010C-A
YDR276C YKL212W
YDR276C YLL061W
YDR276C YLR241W
YDR276C YMR149W
YDR276C YOR211C
YDR277C YDR388W
YDR277C YGL237C
YDR277C YHL002W
YDR277C YHR114W
YDR277C YJL020C
YDR277C YJR022W
YDR277C YKL129C
YDR277C YLR191W
YDR277C YLR373C
YDR277C YLR447C
YDR277C YMR109W
YDR277C YNL201C
YDR277C YOR060C
YDR277C YOR358W
YDR279WYFL029C
YDR279WYGR262C
YDR279WYIL131C
YDR279WYLR154C
YDR279WYNL106C
YDR280WYGR090W
YDR280WYGR095C
YDR280WYGR158C
YDR280WYGR195W
YDR280WYHR069C
YDR280WYJL109C
YDR280WYLR398C

YDR280WYMR128W
YDR280WYNL189W
YDR280WYNL232W
YDR280WYOL021C
YDR280WYOL142W
YDR280WYOR001W
YDR280WYOR076C
YDR280WYPR189W
YDR281C YLR267W
YDR283C YGR091W
YDR283C YJR110W
YDR283C YKL173W
YDR283C YLR096W
YDR283C YLR409C
YDR283C YMR186W
YDR283C YMR205C
YDR283C YNL025C
YDR283C YNL183C
YDR283C YNL213C
YDR283C YOR308C
YDR283C YPL204W
YDR283C YPL240C
YDR283C YPR115W
YDR283C YPR178W
YDR284C YHL017W
YDR284C YKR088C
YDR284C YLR083C
YDR284C YPL147W
YDR284C YPR079W
YDR285WYLR394W
YDR285WYNL201C
YDR285WYOL069W
YDR286C YGL070C
YDR287WYHR163W
YDR289C YER112W
YDR289C YNL189W
YDR290WYKL002W
YDR291WYJL020C
YDR292C YKL154W
YDR292C YMR163C
YDR293C YER082C
YDR293C YML064C
YDR293C YNL161W
YDR295C YGL086W
YDR295C YJR112W

YDR295C YLR131C
YDR295C YMR117C
YDR295C YNL021W
YDR295C YNL250W
YDR295C YOL069W
YDR295C YOL090W
YDR296WYGR220C
YDR296WYNL185C
YDR296WYNL284C
YDR296WYPL248C
YDR297WYDR331W
YDR297WYEL002C
YDR297WYER026C
YDR297WYGL200C
YDR297WYGR125W
YDR297WYGR260W
YDR297WYHR140W
YDR297WYHR142W
YDR297WYIL016W
YDR297WYJL117W
YDR297WYJL196C
YDR297WYJR010C-A
YDR297WYKL065C
YDR297WYKL154W
YDR297WYLL028W
YDR297WYLR372W
YDR297WYML048W
YDR297WYMR149W
YDR297WYOL003C
YDR297WYOR016C
YDR297WYPL076W
YDR297WYPL264C
YDR297WYPL274W
YDR298C YFR028C
YDR298C YJR121W
YDR298C YKL016C
YDR299WYDR466W
YDR299WYER127W
YDR299WYGR090W
YDR299WYGR145W
YDR299WYNL207W
YDR299WYOR014W
YDR299WYPL042C
YDR299WYPR161C
YDR300C YER107C

YDR300C YML095C
YDR300C YNL030W
YDR300C YNL265C
YDR300C YPR137W
YDR301WYER133W
YDR301WYGR156W
YDR301WYGR240C
YDR301WYJR093C
YDR301WYKL018W
YDR301WYKL059C
YDR301WYKR002W
YDR301WYLR115W
YDR301WYLR277C
YDR301WYMR061W
YDR301WYNL317W
YDR301WYOR250C
YDR301WYPR107C
YDR303C YHR056C
YDR303C YLR113W
YDR303C YLR176C
YDR303C YNL030W
YDR305C YLR447C
YDR306C YDR328C
YDR306C YER043C
YDR306C YER081W
YDR306C YIR017C
YDR306C YJR045C
YDR306C YKL085W
YDR306C YLR259C
YDR306C YMR105C
YDR306C YNL138W
YDR306C YOR027W
YDR306C YPL240C
YDR307WYER026C
YDR307WYER081W
YDR307WYER145C
YDR307WYFL062W
YDR307WYHR140W
YDR307WYJL091C
YDR307WYJL196C
YDR307WYJR117W
YDR307WYKL065C
YDR307WYKL154W
YDR307WYLR372W
YDR307WYML048W

YDR307WYMR153W
YDR307WYOR016C
YDR308C YDR448W
YDR308C YEL009C
YDR308C YGL127C
YDR308C YGR104C
YDR308C YOL051W
YDR308C YOL135C
YDR308C YOR119C
YDR308C YOR174W
YDR309C YGR207C
YDR309C YHR061C
YDR309C YHR107C
YDR309C YKL007W
YDR309C YKL082C
YDR309C YLR229C
YDR309C YML109W
YDR309C YMR055C
YDR309C YMR238W
YDR309C YMR273C
YDR309C YNL298W
YDR309C YOR127W
YDR309C YPL161C
YDR310C YFR024C-A
YDR310C YHR152W
YDR310C YOL068C
YDR310C YOR279C
YDR311WYDR337W
YDR311WYEL037C
YDR311WYER086W
YDR311WYGL198W
YDR311WYGR033C
YDR311WYGR120C
YDR311WYGR258C
YDR311WYHR113W
YDR311WYIL021W
YDR311WYIL071C
YDR311WYIL144W
YDR311WYKL028W
YDR311WYKL103C
YDR311WYKL104C
YDR311WYKR050W
YDR311WYLR005W
YDR311WYLR288C
YDR311WYLR423C

YDR311WYMR201C
YDR311WYMR294W
YDR311WYNL135C
YDR311WYOL082W
YDR311WYOR089C
YDR311WYOR197W
YDR311WYPL024W
YDR311WYPL170W
YDR311WYPR056W
YDR311WYPR183W
YDR312WYER114C
YDR312WYOR080W
YDR313C YIL148W
YDR313C YJL088W
YDR313C YJR091C
YDR313C YNL044W
YDR313C YPL133C
YDR314C YEL037C
YDR314C YML011C
YDR315C YJR117W
YDR315C YLR264W
YDR315C YLR323C
YDR315C YOR078W
YDR315C YPR132W
YDR316WYER110C
YDR316WYFL053W
YDR316WYGR041W
YDR316WYHR082C
YDR316WYLR447C
YDR316WYOL055C
YDR316WYPL258C
YDR316WYPR121W
YDR317WYLR373C
YDR318WYDR328C
YDR318WYDR455C
YDR318WYDR478W
YDR318WYDR542W
YDR318WYEL068C
YDR318WYER029C
YDR318WYER035W
YDR318WYER084W
YDR318WYFL015C
YDR318WYFR054C
YDR318WYGL239C
YDR318WYGR018C

YDR318WYGR036C
YDR318WYGR146C
YDR318WYGR179C
YDR318WYHL042W
YDR318WYHR142W
YDR318WYHR152W
YDR318WYHR189W
YDR318WYIL141W
YDR318WYJR060W
YDR318WYKL089W
YDR318WYLL033W
YDR318WYLL037W
YDR318WYNL189W
YDR318WYPL018W
YDR319C YHR114W
YDR319C YLR295C
YDR319C YLR452C
YDR320C YGL206C
YDR320C YGR167W
YDR320C YPL022W
YDR321WYGL095C
YDR321WYLR347C
YDR321WYML064C
YDR321WYPL070W
YDR322C-A YJR121W
YDR322WYGR091W
YDR322WYGR220C
YDR322WYJL041W
YDR322WYNL185C
YDR322WYNL284C
YDR323C YKR014C
YDR324C YER082C
YDR324C YGR090W
YDR324C YJL069C
YDR324C YOL108C
YDR324C YPL022W
YDR326C YER007C-A
YDR326C YIL105C
YDR326C YJR035W
YDR327WYOR378W
YDR328C YDR463W
YDR328C YEL030W
YDR328C YEL051W
YDR328C YEL060C
YDR328C YFL009W

YDR328C YFR030W
YDR328C YGL149W
YDR328C YGL186C
YDR328C YGR020C
YDR328C YGR032W
YDR328C YGR140W
YDR328C YHR073W
YDR328C YHR102W
YDR328C YHR115C
YDR328C YHR205W
YDR328C YIL046W
YDR328C YJL149W
YDR328C YJL204C
YDR328C YJR033C
YDR328C YJR089W
YDR328C YJR090C
YDR328C YLR079W
YDR328C YLR097C
YDR328C YLR224W
YDR328C YLR267W
YDR328C YLR352W
YDR328C YLR368W
YDR328C YLR399C
YDR328C YLR421C
YDR328C YLR429W
YDR328C YML088W
YDR328C YMR094W
YDR328C YMR155W
YDR328C YMR168C
YDR328C YMR199W
YDR328C YMR258C
YDR328C YMR291W
YDR328C YNL015W
YDR328C YNL116W
YDR328C YNL311C
YDR328C YNR019W
YDR328C YOL055C
YDR328C YOL098C
YDR328C YOL117W
YDR328C YOL133W
YDR328C YOR057W
YDR328C YOR080W
YDR328C YOR133W
YDR328C YOR332W
YDR328C YPL240C

YDR328C YPL256C
YDR328C YPR019W
YDR328C YPR036W
YDR329C YLR002C
YDR329C YLR191W
YDR329C YMR203W
YDR329C YOL044W
YDR331WYDR343C
YDR331WYDR456W
YDR331WYEL027W
YDR331WYER026C
YDR331WYER081W
YDR331WYGL051W
YDR331WYGL053W
YDR331WYGL104C
YDR331WYGR174C
YDR331WYGR284C
YDR331WYGR289C
YDR331WYHL003C
YDR331WYHR007C
YDR331WYHR026W
YDR331WYHR133C
YDR331WYHR140W
YDR331WYHR188C
YDR331WYIL016W
YDR331WYJL091C
YDR331WYJL117W
YDR331WYJL196C
YDR331WYJR117W
YDR331WYJR158W
YDR331WYKL006C-A
YDR331WYKL008C
YDR331WYKL065C
YDR331WYKL154W
YDR331WYKL212W
YDR331WYKR039W
YDR331WYLR018C
YDR331WYLR088W
YDR331WYLR372W
YDR331WYML048W
YDR331WYMR058W
YDR331WYMR215W
YDR331WYMR296C
YDR331WYNL044W
YDR331WYOL003C

YDR331WYOL156W
YDR331WYOR307C
YDR331WYPL076W
YDR331WYPL227C
YDR331WYPL274W
YDR331WYPR028W
YDR332WYMR074C
YDR333C YPL118W
YDR334WYDR485C
YDR334WYER114C
YDR334WYFL039C
YDR334WYGR002C
YDR334WYJL081C
YDR334WYLR385C
YDR334WYLR399C
YDR334WYML041C
YDR334WYNL107W
YDR334WYOL012C
YDR334WYPL022W
YDR334WYPL235W
YDR335WYFR002W
YDR335WYFR034C
YDR335WYGL035C
YDR335WYGL092W
YDR335WYGL172W
YDR335WYGR009C
YDR335WYGR119C
YDR335WYJL061W
YDR335WYKL068W
YDR335WYKR082W
YDR335WYLR293C
YDR335WYLR335W
YDR335WYML103C
YDR335WYMR047C
YDR335WYOL051W
YDR335WYOR098C
YDR335WYOR359W
YDR335WYPL240C
YDR335WYPR008W
YDR337WYHL004W
YDR337WYPR084W
YDR339C YGL006W
YDR339C YJR045C
YDR339C YLR180W
YDR339C YNL064C

YDR340WYGL070C
YDR341C YIR005W
YDR341C YNL135C
YDR342C YER048C
YDR342C YGL042C
YDR342C YJL173C
YDR342C YKR026C
YDR342C YLR019W
YDR342C YLR340W
YDR342C YMR049C
YDR342C YNL323W
YDR342C YOL100W
YDR342C YOR045W
YDR342C YOR181W
YDR342C YPL204W
YDR343C YER081W
YDR343C YER125W
YDR343C YER171W
YDR343C YGR040W
YDR343C YGR060W
YDR343C YJR017C
YDR343C YKL193C
YDR343C YLR019W
YDR343C YNL032W
YDR343C YOL100W
YDR343C YOR181W
YDR344C YOR196C
YDR345C YLL006W
YDR345C YOR047C
YDR346C YLR077W
YDR347WYHL004W
YDR348C YER125W
YDR348C YMR295C
YDR349C YLR099C
YDR349C YOR007C
YDR350C YNL126W
YDR350C YOL123W
YDR351WYJR005W
YDR351WYLR191W
YDR351WYNL070W
YDR351WYOR284W
YDR353WYKR083C
YDR353WYLR216C
YDR353WYLR347C
YDR353WYLR373C

YDR353WYNL180C
YDR353WYNL189W
YDR353WYNL244C
YDR353WYOL128C
YDR356WYER129W
YDR356WYGR161C
YDR356WYHR172W
YDR356WYJL187C
YDR356WYKL042W
YDR356WYLR006C
YDR356WYLR212C
YDR356WYNL053W
YDR356WYNL126W
YDR356WYOR257W
YDR356WYPL124W
YDR356WYPL180W
YDR357C YGL079W
YDR357C YLR423C
YDR357C YPR182W
YDR359C YFL024C
YDR359C YGR002C
YDR359C YHR099W
YDR359C YJL098W
YDR359C YLR113W
YDR359C YNL107W
YDR359C YOR119C
YDR359C YOR244W
YDR361C YER117W
YDR361C YJR091C
YDR362C YDR388W
YDR362C YMR226C
YDR363W-A YER021W
YDR363W-A YGR232W
YDR363W-A YHR027C
YDR363W-A YPR108W
YDR364C YKL095W
YDR364C YMR213W
YDR365C YDR381W
YDR365C YER165W
YDR365C YFR053C
YDR365C YGL008C
YDR365C YGL019W
YDR365C YGL173C
YDR365C YHL034C
YDR365C YIL035C

YDR365C YJL207C
YDR365C YJR045C
YDR365C YKR081C
YDR365C YLR432W
YDR365C YMR296C
YDR365C YNR054C
YDR365C YOL041C
YDR365C YOL139C
YDR365C YOR061W
YDR366C YER071C
YDR366C YGR054W
YDR367WYHR065C
YDR368WYNL166C
YDR369C YFL039C
YDR369C YGL090W
YDR369C YHR082C
YDR369C YJL167W
YDR369C YJR045C
YDR369C YKL022C
YDR369C YLR109W
YDR369C YMR224C
YDR369C YNL250W
YDR369C YOL055C
YDR370C YFL049W
YDR371WYIR038C
YDR372C YDR523C
YDR372C YJL053W
YDR372C YLR352W
YDR372C YMR047C
YDR373WYGL153W
YDR373WYGR082W
YDR373WYNL267W
YDR373WYOR136W
YDR374C YJL070C
YDR376WYIR024C
YDR377WYJR121W
YDR377WYKL016C
YDR377WYML081C-A
YDR377WYPL078C
YDR378C YER112W
YDR378C YER146W
YDR378C YGL251C
YDR378C YGR091W
YDR378C YHR165C
YDR378C YJL124C

YDR378C YJL139C
YDR378C YJR022W
YDR378C YLR053C
YDR378C YLR147C
YDR378C YLR275W
YDR378C YLR295C
YDR378C YLR438C-A
YDR378C YMR221C
YDR378C YMR268C
YDR378C YNL070W
YDR378C YNL147W
YDR378C YOL149W
YDR378C YOR320C
YDR379WYNL201C
YDR380WYOR382W
YDR381WYDR386W
YDR381WYGL190C
YDR381WYGL237C
YDR381WYIL035C
YDR381WYIL038C
YDR381WYIL095W
YDR381WYJL098W
YDR381WYKL214C
YDR381WYKR048C
YDR381WYMR308C
YDR381WYNL088W
YDR381WYNL230C
YDR381WYOL113W
YDR381WYOR267C
YDR381WYPL169C
YDR382WYEL054C
YDR382WYFL039C
YDR382WYLR340W
YDR383C YLR083C
YDR383C YLR315W
YDR383C YLR423C
YDR383C YNL189W
YDR383C YOL104C
YDR383C YOR159C
YDR383C YPL002C
YDR384C YMR009W
YDR384C YNR060W
YDR384C YPR156C
YDR386WYDR388W
YDR386WYDR502C

YDR386WYER006W
YDR386WYER078C
YDR386WYER110C
YDR386WYER112W
YDR386WYER165W
YDR386WYFL039C
YDR386WYFR001W
YDR386WYGL173C
YDR386WYGL206C
YDR386WYGR090W
YDR386WYGR234W
YDR386WYGR264C
YDR386WYHL034C
YDR386WYHR143W-A
YDR386WYIL148W
YDR386WYJL008C
YDR386WYJL020C
YDR386WYJL066C
YDR386WYJL088W
YDR386WYJR077C
YDR386WYJR144W
YDR386WYKL022C
YDR386WYKL152C
YDR386WYKR081C
YDR386WYLR180W
YDR386WYLR196W
YDR386WYML074C
YDR386WYMR001C
YDR386WYMR049C
YDR386WYMR226C
YDR386WYMR303C
YDR386WYNL024C
YDR386WYNL030W
YDR386WYNL061W
YDR386WYNL064C
YDR386WYNL085W
YDR386WYNL132W
YDR386WYNL308C
YDR386WYOL041C
YDR386WYOL139C
YDR386WYPL061W
YDR386WYPL108W
YDR386WYPL127C
YDR386WYPL129W
YDR386WYPL153C

YDR386WYPR119W
YDR386WYPR181C
YDR387C YLR292C
YDR387C YOR292C
YDR388WYDR515W
YDR388WYER003C
YDR388WYER032W
YDR388WYER043C
YDR388WYER091C
YDR388WYER110C
YDR388WYER125W
YDR388WYER127W
YDR388WYFL016C
YDR388WYFL018C
YDR388WYFL039C
YDR388WYFR013W
YDR388WYFR053C
YDR388WYGL016W
YDR388WYGL026C
YDR388WYGL060W
YDR388WYGL144C
YDR388WYGL181W
YDR388WYGL190C
YDR388WYGL201C
YDR388WYGL202W
YDR388WYGL226W
YDR388WYGL253W
YDR388WYGR058W
YDR388WYGR098C
YDR388WYGR099W
YDR388WYGR155W
YDR388WYGR159C
YDR388WYGR240C
YDR388WYGR268C
YDR388WYHL030W
YDR388WYHR016C
YDR388WYHR065C
YDR388WYHR137W
YDR388WYHR183W
YDR388WYIL094C
YDR388WYIL105C
YDR388WYIL106W
YDR388WYIL128W
YDR388WYIL147C
YDR388WYIL148W

YDR388WYIL156W
YDR388WYJL034W
YDR388WYJL085W
YDR388WYJL130C
YDR388WYJL133W
YDR388WYJL138C
YDR388WYJL153C
YDR388WYJL194W
YDR388WYJL195C
YDR388WYJR035W
YDR388WYJR045C
YDR388WYJR077C
YDR388WYJR083C
YDR388WYJR090C
YDR388WYJR115W
YDR388WYJR121W
YDR388WYJR139C
YDR388WYKL035W
YDR388WYKL129C
YDR388WYKL214C
YDR388WYKR027W
YDR388WYLL023C
YDR388WYLL028W
YDR388WYLR044C
YDR388WYLR144C
YDR388WYLR180W
YDR388WYLR243W
YDR388WYLR337C
YDR388WYLR355C
YDR388WYLR425W
YDR388WYLR433C
YDR388WYLR436C
YDR388WYML085C
YDR388WYML109W
YDR388WYML123C
YDR388WYMR003W
YDR388WYMR068W
YDR388WYMR109W
YDR388WYMR162C
YDR388WYMR192W
YDR388WYMR287C
YDR388WYMR308C
YDR388WYNL037C
YDR388WYNL047C
YDR388WYNL064C

YDR388WYNL086W
YDR388WYNL094W
YDR388WYNL138W
YDR388WYNL152W
YDR388WYNL176C
YDR388WYNL206C
YDR388WYNL243W
YDR388WYNL287W
YDR388WYNR001C
YDR388WYOL058W
YDR388WYOL139C
YDR388WYOR004W
YDR388WYOR042W
YDR388WYOR047C
YDR388WYOR181W
YDR388WYOR204W
YDR388WYOR284W
YDR388WYOR329C
YDR388WYOR362C
YDR388WYOR375C
YDR388WYPL061W
YDR388WYPL135C-A
YDR388WYPL140C
YDR388WYPL181W
YDR388WYPL235W
YDR388WYPL249C
YDR388WYPL249C-A
YDR388WYPR055W
YDR388WYPR074C
YDR388WYPR081C
YDR388WYPR088C
YDR388WYPR091C
YDR388WYPR095C
YDR388WYPR110C
YDR388WYPR171W
YDR389WYGR074W
YDR389WYJR091C
YDR389WYPR165W
YDR390C YDR510W
YDR390C YGR048W
YDR390C YKR002W
YDR390C YPR180W
YDR392WYDR448W
YDR392WYER148W
YDR392WYHR099W

YDR392WYOL148C
YDR392WYOR119C
YDR392WYPL181W
YDR394WYDR427W
YDR394WYDR488C
YDR394WYEL034W
YDR394WYEL037C
YDR394WYER012W
YDR394WYER021W
YDR394WYER081W
YDR394WYER095W
YDR394WYER171W
YDR394WYER173W
YDR394WYER179W
YDR394WYFL016C
YDR394WYFR004W
YDR394WYFR010W
YDR394WYFR052W
YDR394WYGL004C
YDR394WYGL048C
YDR394WYGL074C
YDR394WYGL137W
YDR394WYGR092W
YDR394WYGR232W
YDR394WYGR262C
YDR394WYHR027C
YDR394WYHR030C
YDR394WYHR200W
YDR394WYIL075C
YDR394WYJL008C
YDR394WYJL130C
YDR394WYJL157C
YDR394WYJR045C
YDR394WYKL073W
YDR394WYKL145W
YDR394WYKR026C
YDR394WYLR106C
YDR394WYLR180W
YDR394WYLR191W
YDR394WYLR196W
YDR394WYLR216C
YDR394WYLR259C
YDR394WYLR306W
YDR394WYML115C
YDR394WYMR001C

YDR394WYMR049C
YDR394WYMR205C
YDR394WYMR216C
YDR394WYMR284W
YDR394WYMR308C
YDR394WYNL055C
YDR394WYNL064C
YDR394WYNL085W
YDR394WYNL250W
YDR394WYNL290W
YDR394WYOL055C
YDR394WYOL094C
YDR394WYOR027W
YDR394WYOR089C
YDR394WYOR117W
YDR394WYOR136W
YDR394WYOR151C
YDR394WYOR259C
YDR394WYOR261C
YDR394WYPL070W
YDR394WYPL235W
YDR394WYPL240C
YDR394WYPR108W
YDR395WYGR119C
YDR395WYIL115C
YDR395WYJL041W
YDR395WYKL068W
YDR395WYMR047C
YDR395WYOL070C
YDR395WYOR098C
YDR395WYPR015C
YDR397C YER159C
YDR398WYER138C
YDR398WYFL045C
YDR398WYGL206C
YDR398WYGR254W
YDR398WYJL026W
YDR398WYJR070C
YDR398WYKL081W
YDR398WYLR044C
YDR398WYLR216C
YDR398WYLR336C
YDR398WYMR093W
YDR398WYMR246W
YDR398WYNR016C

YDR398WYOR133W
YDR398WYOR136W
YDR398WYOR259C
YDR398WYOR375C
YDR398WYPL061W
YDR398WYPL216W
YDR399WYNL189W
YDR403WYKR053C
YDR404C YER125W
YDR404C YGL070C
YDR404C YGR005C
YDR404C YGR063C
YDR404C YGR186W
YDR404C YIL021W
YDR404C YJL070C
YDR404C YJL140W
YDR404C YJL164C
YDR404C YML010W
YDR404C YOL005C
YDR404C YOR151C
YDR404C YOR224C
YDR404C YPL203W
YDR404C YPR180W
YDR405WYER154W
YDR405WYGR220C
YDR405WYNL185C
YDR407C YJL044C
YDR407C YKR068C
YDR408C YGL127C
YDR408C YKL002W
YDR408C YOR174W
YDR409WYJL020C
YDR409WYJR076C
YDR409WYLR314C
YDR409WYMR032W
YDR410C YJL117W
YDR410C YLR399C
YDR410C YNL101W
YDR412WYER133W
YDR412WYMR049C
YDR412WYPR119W
YDR413C YGR151C
YDR413C YGR289C
YDR414C YDR503C
YDR414C YEL004W

YDR414C YEL017C-A
YDR414C YER005W
YDR414C YER118C
YDR414C YFL040W
YDR414C YFL041W
YDR414C YFR024C-A
YDR414C YGL104C
YDR414C YGL259W
YDR414C YGR199W
YDR414C YGR284C
YDR414C YHL042W
YDR414C YIL173W
YDR414C YJL004C
YDR414C YJL133W
YDR414C YJL193W
YDR414C YKL120W
YDR414C YLL023C
YDR414C YLL056C
YDR414C YLR026C
YDR414C YLR242C
YDR414C YLR372W
YDR414C YLR411W
YDR414C YML038C
YDR414C YMR155W
YDR414C YMR292W
YDR414C YNL044W
YDR414C YNL326C
YDR414C YNR026C
YDR414C YOL075C
YDR414C YOR254C
YDR414C YPL264C
YDR415C YJR089W
YDR415C YOR305W
YDR416WYGR119C
YDR416WYGR129W
YDR416WYHR114W
YDR416WYHR165C
YDR416WYJR050W
YDR416WYKL095W
YDR416WYLL036C
YDR416WYLR117C
YDR416WYLR291C
YDR416WYMR213W
YDR416WYPL077C
YDR416WYPR101W

YDR419WYFL018C
YDR419WYGL173C
YDR419WYJL088W
YDR419WYJL130C
YDR419WYJR121W
YDR419WYNL064C
YDR419WYOL055C
YDR419WYPR160W
YDR422C YDR477W
YDR422C YDR516C
YDR422C YER029C
YDR422C YGL115W
YDR422C YHR114W
YDR422C YJL066C
YDR422C YJL124C
YDR422C YLR306W
YDR422C YLR453C
YDR422C YMR106C
YDR422C YOR027W
YDR422C YPR078C
YDR423C YMR047C
YDR424C YDR488C
YDR424C YIL095W
YDR424C YPR119W
YDR425WYGL161C
YDR425WYGL198W
YDR425WYGR172C
YDR425WYJL036W
YDR425WYJR110W
YDR425WYKR014C
YDR425WYPL280W
YDR427WYEL037C
YDR427WYER012W
YDR427WYER148W
YDR427WYFR004W
YDR427WYFR010W
YDR427WYFR052W
YDR427WYGL004C
YDR427WYGL201C
YDR427WYGR067C
YDR427WYGR232W
YDR427WYHR027C
YDR427WYHR200W
YDR427WYIL066C
YDR427WYKL145W

YDR427WYLR386W
YDR427WYMR276W
YDR427WYNL244C
YDR427WYNR059W
YDR427WYOR261C
YDR427WYPR108W
YDR428C YNL189W
YDR428C YOR157C
YDR429C YFL017C
YDR429C YGL137W
YDR429C YJL070C
YDR429C YLR190W
YDR429C YLR192C
YDR429C YMR146C
YDR429C YMR309C
YDR429C YNL244C
YDR429C YOL087C
YDR429C YOR039W
YDR429C YOR361C
YDR429C YPL105C
YDR429C YPR041W
YDR429C YPR086W
YDR430C YER178W
YDR430C YFL018C
YDR430C YGR193C
YDR430C YNL071W
YDR432WYGR054W
YDR432WYHR086W
YDR432WYIL079C
YDR432WYIR001C
YDR432WYKL139W
YDR432WYLR427W
YDR432WYMR001C
YDR432WYNL016W
YDR432WYOR267C
YDR432WYPL178W
YDR433WYLR453C
YDR433WYNL116W
YDR435C YFL040W
YDR436WYFR003C
YDR436WYKL088W
YDR436WYKL193C
YDR436WYKR072C
YDR436WYML016C
YDR436WYMR311C

YDR436WYOR054C
YDR436WYPR005C
YDR438WYJR091C
YDR439WYFR028C
YDR439WYJR091C
YDR439WYMR001C
YDR441C YML022W
YDR443C YFL028C
YDR443C YKR036C
YDR443C YNL288W
YDR443C YNR052C
YDR443C YOL135C
YDR443C YOR119C
YDR443C YOR140W
YDR443C YPL042C
YDR444WYLR291C
YDR445C YER071C
YDR445C YLR295C
YDR446WYDR510W
YDR448WYEL009C
YDR448WYER022W
YDR448WYER148W
YDR448WYFL031W
YDR448WYGL066W
YDR448WYGL112C
YDR448WYGR252W
YDR448WYGR274C
YDR448WYHR041C
YDR448WYHR099W
YDR448WYHR166C
YDR448WYKL012W
YDR448WYLR291C
YDR448WYMR223W
YDR448WYMR236W
YDR448WYOL135C
YDR448WYOL148C
YDR448WYOR023C
YDR448WYOR119C
YDR448WYPL181W
YDR448WYPL248C
YDR448WYPL254W
YDR449C YER082C
YDR449C YGR090W
YDR449C YJL069C
YDR449C YPR041W

YDR450WYOR037W
YDR452WYER112W
YDR452WYNL217W
YDR452WYPR086W
YDR453C YFR028C
YDR453C YIL039W
YDR453C YJL141C
YDR453C YMR059W
YDR453C YMR106C
YDR453C YNL189W
YDR453C YOR031W
YDR453C YPR110C
YDR455C YFL020C
YDR455C YLL046C
YDR455C YLR173W
YDR455C YLR295C
YDR455C YOR108W
YDR456WYER145C
YDR456WYGL008C
YDR456WYGR216C
YDR456WYHL042W
YDR456WYIL030C
YDR456WYIR022W
YDR456WYKR050W
YDR456WYLR372W
YDR456WYNR056C
YDR456WYOR123C
YDR456WYOR125C
YDR456WYPL092W
YDR456WYPR079W
YDR457WYGR052W
YDR457WYJR017C
YDR459C YGR216C
YDR459C YJL012C
YDR459C YMR149W
YDR459C YNL048W
YDR460WYER171W
YDR460WYLR259C
YDR460WYLR288C
YDR460WYPR025C
YDR461WYLR384C
YDR462WYGR091W
YDR462WYGR220C
YDR462WYNL284C
YDR463WYOL133W

YDR464WYHR077C
YDR464WYNL135C
YDR465C YJR021C
YDR465C YMR047C
YDR466WYER160C
YDR466WYHR052W
YDR466WYHR088W
YDR466WYJL095W
YDR466WYLR196W
YDR466WYMR049C
YDR466WYMR066W
YDR466WYMR109W
YDR466WYOL041C
YDR466WYOR201C
YDR466WYPL093W
YDR468C YGL095C
YDR468C YGR172C
YDR468C YIL140W
YDR468C YKR020W
YDR468C YLL043W
YDR468C YMR018W
YDR468C YMR197C
YDR468C YNL044W
YDR468C YOL018C
YDR469WYEL005C
YDR469WYHR060W
YDR469WYHR119W
YDR469WYLR015W
YDR469WYLR432W
YDR469WYPL138C
YDR472WYKR068C
YDR472WYML077W
YDR473C YER172C
YDR473C YFL017W-A
YDR473C YGR091W
YDR473C YHR165C
YDR473C YJR022W
YDR473C YLR147C
YDR473C YLR423C
YDR473C YLR438C-A
YDR473C YPL031C
YDR473C YPR178W
YDR475C YER133W
YDR475C YML016C
YDR477WYEL060C

YDR477WYER027C
YDR477WYER040W
YDR477WYER129W
YDR477WYER177W
YDR477WYFL037W
YDR477WYFR028C
YDR477WYFR053C
YDR477WYGL035C
YDR477WYGL115W
YDR477WYGL158W
YDR477WYGL208W
YDR477WYIL148W
YDR477WYJL076W
YDR477WYJL089W
YDR477WYJL130C
YDR477WYKL182W
YDR477WYLR055C
YDR477WYLR180W
YDR477WYLR191W
YDR477WYML006C
YDR477WYML085C
YDR477WYMR001C
YDR477WYMR086W
YDR477WYMR104C
YDR477WYNL025C
YDR477WYNL183C
YDR477WYNL218W
YDR477WYNL236W
YDR477WYNL257C
YDR477WYOL062C
YDR477WYOL139C
YDR477WYOR047C
YDR477WYOR267C
YDR477WYOR351C
YDR477WYPL042C
YDR477WYPL259C
YDR479C YER081W
YDR479C YGL127C
YDR479C YLR324W
YDR480WYER114C
YDR480WYER177W
YDR480WYGR040W
YDR480WYHR016C
YDR480WYHR039C
YDR480WYHR084W

YDR480WYHR114W
YDR480WYKL161C
YDR480WYLR191W
YDR480WYLR259C
YDR480WYLR304C
YDR480WYLR310C
YDR480WYMR199W
YDR480WYNL189W
YDR480WYPL020C
YDR480WYPL049C
YDR480WYPL256C
YDR482C YGL028C
YDR482C YJR091C
YDR482C YLR259C
YDR482C YOR276W
YDR483WYER081W
YDR483WYHR096C
YDR484WYGR245C
YDR484WYJL029C
YDR484WYKR020W
YDR484WYLR262C
YDR484WYLR291C
YDR484WYLR447C
YDR484WYPL218W
YDR485C YER112W
YDR485C YGR002C
YDR485C YNL107W
YDR485C YOL012C
YDR487C YNL189W
YDR488C YGR180C
YDR488C YGR254W
YDR488C YIL135C
YDR488C YJL034W
YDR488C YJR045C
YDR488C YKL152C
YDR488C YMR066W
YDR488C YNR058W
YDR488C YOR172W
YDR488C YPL174C
YDR488C YPL258C
YDR489WYFR043C
YDR489WYJL072C
YDR489WYLR453C
YDR489WYML034W
YDR489WYPL077C

YDR490C YGR086C
YDR490C YHL048W
YDR490C YHR207C
YDR490C YJR059W
YDR490C YKL166C
YDR490C YLR466W
YDR490C YML074C
YDR490C YMR047C
YDR490C YOR061W
YDR490C YPL004C
YDR492WYJR067C
YDR492WYOR315W
YDR496C YER161C
YDR496C YGR274C
YDR496C YHR066W
YDR496C YHR089C
YDR496C YIL035C
YDR496C YKR081C
YDR496C YLR074C
YDR496C YLR175W
YDR496C YLR233C
YDR496C YNL061W
YDR496C YNL175C
YDR496C YNL230C
YDR496C YPL043W
YDR496C YPR016C
YDR497C YER125W
YDR497C YHR076W
YDR497C YLL028W
YDR497C YNL217W
YDR497C YNL307C
YDR497C YOR254C
YDR498C YGL145W
YDR498C YLR268W
YDR498C YOR075W
YDR499WYGR218W
YDR499WYHR164C
YDR499WYJL090C
YDR499WYJR045C
YDR499WYJR121W
YDR499WYLR180W
YDR499WYLR355C
YDR499WYMR303C
YDR499WYNL030W
YDR499WYOR176W

YDR500C YFL018C
YDR500C YJR091C
YDR500C YNL071W
YDR502C YER066C-A
YDR502C YER171W
YDR502C YER173W
YDR502C YGL116W
YDR502C YGR040W
YDR502C YHR030C
YDR502C YJL173C
YDR502C YJR017C
YDR502C YJR068W
YDR502C YJR090C
YDR502C YKL139W
YDR502C YLR096W
YDR502C YLR320W
YDR502C YLR442C
YDR502C YML064C
YDR502C YML095C
YDR502C YMR059W
YDR502C YNL094W
YDR502C YNL230C
YDR502C YOL133W
YDR502C YOR319W
YDR502C YPL070W
YDR502C YPL125W
YDR502C YPL150W
YDR502C YPR110C
YDR503C YER049W
YDR503C YKL130C
YDR503C YNR074C
YDR504C YJL070C
YDR505C YGL122C
YDR505C YPR154W
YDR506C YJR010C-A
YDR506C YPR201W
YDR507C YGL158W
YDR507C YHR107C
YDR507C YJL095W
YDR507C YJR076C
YDR507C YKL101W
YDR507C YKR048C
YDR507C YLR314C
YDR507C YMR139W
YDR507C YNL166C

YDR507C YOL069W
YDR507C YOR231W
YDR508C YIL114C
YDR508C YKL220C
YDR508C YLR373C
YDR508C YMR279C
YDR508C YOR123C
YDR508C YPR025C
YDR510WYGL127C
YDR510WYJL092W
YDR510WYJR091C
YDR510WYKL043W
YDR510WYLR263W
YDR510WYLR295C
YDR510WYMR047C
YDR510WYMR159C
YDR510WYMR211W
YDR510WYNL088W
YDR510WYNL189W
YDR510WYNR012W
YDR510WYOL034W
YDR510WYOR156C
YDR510WYOR191W
YDR510WYPR104C
YDR510WYPR180W
YDR511WYGL181W
YDR512C YER081W
YDR514C YNL173C
YDR515WYGR172C
YDR515WYHR114W
YDR515WYPR161C
YDR516C YGR169C
YDR516C YJR091C
YDR516C YNL032W
YDR517WYGL237C
YDR520C YKR034W
YDR523C YEL051W
YDR523C YEL060C
YDR523C YER110C
YDR523C YER178W
YDR523C YFL016C
YDR523C YFL037W
YDR523C YFL039C
YDR523C YGL048C
YDR523C YGL202W

YDR523C YGR282C
YDR523C YHL002W
YDR523C YHR033W
YDR523C YJL066C
YDR523C YJR077C
YDR523C YJR121W
YDR523C YJR132W
YDR523C YKL152C
YDR523C YKR046C
YDR523C YLL041C
YDR523C YLR180W
YDR523C YLR216C
YDR523C YML085C
YDR523C YML123C
YDR523C YML124C
YDR523C YMR214W
YDR523C YNL037C
YDR523C YNL064C
YDR523C YOR261C
YDR523C YPL235W
YDR524C YER081W
YDR524C YOR066W
YDR526C YJR091C
YDR527WYJR091C
YDR527WYOL131W
YDR527WYOR210W
YDR528WYER081W
YDR528WYNL189W
YDR528WYNL206C
YDR529C YGL061C
YDR529C YHR114W
YDR529C YLR292C
YDR530C YLR295C
YDR531WYER081W
YDR532C YER022W
YDR532C YHR079C
YDR532C YIL134W
YDR532C YKL194C
YDR533C YJR091C
YDR533C YMR010W
YDR533C YNL189W
YDR534C YGL127C
YDR534C YPR191W
YDR536WYIL135C
YDR538WYGL044C

YDR541C	YLR025W
YDR542W	YOR187W
YDR545W	YFR024C-A
YEL002C	YER026C
YEL002C	YGL022W
YEL002C	YGL226C-A
YEL002C	YGR284C
YEL002C	YHL003C
YEL002C	YIL016W
YEL002C	YJL002C
YEL002C	YJL117W
YEL002C	YJR117W
YEL002C	YKL008C
YEL002C	YKL065C
YEL002C	YLL028W
YEL002C	YLR372W
YEL002C	YML048W
YEL002C	YML075C
YEL002C	YML115C
YEL002C	YMR149W
YEL002C	YMR264W
YEL002C	YOL003C
YEL002C	YOR016C
YEL002C	YOR085W
YEL002C	YOR103C
YEL002C	YOR254C
YEL002C	YPL076W
YEL002C	YPL227C
YEL002C	YPL234C
YEL002C	YPR011C
YEL003W	YLR200W
YEL003W	YML094W
YEL004W	YPL264C
YEL005C	YEL013W
YEL005C	YGL079W
YEL005C	YGR107W
YEL005C	YJL061W
YEL005C	YNL086W
YEL006W	YLR373C
YEL008W	YJR091C
YEL009C	YER022W
YEL009C	YER148W
YEL009C	YFL009W
YEL009C	YGL112C
YEL009C	YGR274C

YEL009C	YHR041C
YEL009C	YHR145C
YEL009C	YMR236W
YEL009C	YPL038W
YEL009C	YPL042C
YEL011W	YGR136W
YEL011W	YPR086W
YEL012W	YER022W
YEL013W	YFL039C
YEL013W	YHR195W
YEL013W	YJR068W
YEL013W	YJR091C
YEL013W	YKL061W
YEL013W	YLR254C
YEL013W	YLR291C
YEL013W	YPR185W
YEL015W	YER032W
YEL015W	YER105C
YEL015W	YER124C
YEL015W	YGL173C
YEL015W	YGR116W
YEL015W	YGR218W
YEL015W	YIL046W
YEL015W	YJR022W
YEL015W	YJR140C
YEL015W	YLR082C
YEL015W	YLR264W
YEL015W	YML064C
YEL015W	YMR109W
YEL015W	YNL118C
YEL015W	YNL189W
YEL015W	YOL149W
YEL015W	YOR167C
YEL015W	YOR285W
YEL015W	YPL204W
YEL016C	YJL157C
YEL016C	YOL150C
YEL017C-A	YER005W
YEL017C-A	YGL200C
YEL017C-A	YGR060W
YEL017C-A	YGR284C
YEL017C-A	YHL042W
YEL017C-A	YJL108C
YEL017C-A	YJL117W
YEL017C-A	YJR117W

YEL017C-A	YKL154W
YEL017C-A	YLL023C
YEL017C-A	YLR083C
YEL017C-A	YLR292C
YEL017C-A	YLR372W
YEL017C-A	YML048W
YEL017C-A	YMR299C
YEL017C-A	YNL194C
YEL017C-A	YNL308C
YEL017C-A	YOL018C
YEL017C-A	YPL227C
YEL017C-A	YPR079W
YEL017W	YEL042W
YEL017W	YGL098W
YEL017W	YGR121C
YEL017W	YHR114W
YEL017W	YHR123W
YEL017W	YIR038C
YEL017W	YKL063C
YEL017W	YOL020W
YEL017W	YPL244C
YEL017W	YPR174C
YEL018W	YFL024C
YEL018W	YHR099W
YEL018W	YOR128C
YEL019C	YOR266W
YEL020C	YIL118W
YEL020W-A	YHR005C-A
YEL020W-A	YNR017W
YEL021W	YFR053C
YEL021W	YKL152C
YEL023C	YJR022W
YEL023C	YML109W
YEL023C	YMR104C
YEL026W	YER172C
YEL026W	YLR147C
YEL026W	YMR059W
YEL027W	YHR026W
YEL027W	YKL065C
YEL027W	YLL028W
YEL027W	YLR034C
YEL027W	YOR016C
YEL027W	YOR270C
YEL027W	YPR201W
YEL030W	YIL095W

YEL030W YJR035W
YEL030W YJR062C
YEL030W YML058W
YEL030W YMR106C
YEL030W YNL161W
YEL030W YNL230C
YEL030W YOL133W
YEL030W YPL256C
YEL031W YHR174W
YEL031W YLR207W
YEL031W YMR215W
YEL032W YGL201C
YEL032W YIL150C
YEL032W YLR103C
YEL032W YLR274W
YEL032W YOR080W
YEL032W YPR019W
YEL034W YHR135C
YEL034W YHR166C
YEL034W YLR074C
YEL034W YLR340W
YEL034W YMR284W
YEL034W YOL069W
YEL034W YOL133W
YEL034W YOR043W
YEL034W YPL111W
YEL034W YPL204W
YEL036C YHR080C
YEL036C YJL183W
YEL036C YJR075W
YEL036C YOL038W
YEL036C YPL050C
YEL036C YPL094C
YEL037C YER012W
YEL037C YER021W
YEL037C YER143W
YEL037C YER148W
YEL037C YER162C
YEL037C YFR004W
YEL037C YFR052W
YEL037C YGL048C
YEL037C YGL207W
YEL037C YHL025W
YEL037C YHL030W
YEL037C YHR027C

YEL037C	YHR200W
YEL037C	YIL075C
YEL037C	YIL143C
YEL037C	YIL148W
YEL037C	YKL145W
YEL037C	YLR441C
YEL037C	YMR201C
YEL037C	YMR276W
YEL037C	YOR117W
YEL037C	YOR157C
YEL037C	YOR261C
YEL037C	YPL096W
YEL037C	YPL122C
YEL037C	YPR032W
YEL038W	YGL127C
YEL039C	YHR195W
YEL041W	YHR115C
YEL041W	YJR049C
YEL041W	YKL009W
YEL041W	YOR315W
YEL042W	YER073W
YEL042W	YHL002W
YEL042W	YJL152W
YEL042W	YLR250W
YEL042W	YPR193C
YEL043W	YER114C
YEL043W	YGR089W
YEL043W	YGR119C
YEL043W	YGR218W
YEL043W	YJL074C
YEL043W	YOL069W
YEL043W	YOR164C
YEL046C	YNL030W
YEL046C	YNL189W
YEL046C	YNL220W
YEL048C	YGL127C
YEL048C	YGR040W
YEL048C	YHR030C
YEL048C	YIL136W
YEL048C	YNL227C
YEL049W	YKL002W
YEL049W	YOR159C
YEL051W	YGL061C
YEL051W	YGR020C
YEL051W	YJR033C

YEL051W	YKL080W
YEL051W	YKR026C
YEL051W	YLR291C
YEL051W	YMR054W
YEL051W	YMR106C
YEL051W	YNL250W
YEL051W	YOR270C
YEL051W	YOR332W
YEL051W	YPR036W
YEL053C	YPR051W
YEL054C	YLR347C
YEL054C	YOL039W
YEL055C	YER082C
YEL055C	YER100W
YEL055C	YGL237C
YEL055C	YJL095W
YEL055C	YLL019C
YEL055C	YLR196W
YEL055C	YPL141C
YEL056W	YHR183W
YEL056W	YJL052W
YEL056W	YJR077C
YEL056W	YJR121W
YEL056W	YLL022C
YEL056W	YMR108W
YEL056W	YNL030W
YEL056W	YOR136W
YEL056W	YOR374W
YEL056W	YPL001W
YEL056W	YPL061W
YEL057C	YKL002W
YEL058W	YNL218W
YEL059W	YJR130C
YEL060C	YGL137W
YEL060C	YIL128W
YEL060C	YJL124C
YEL060C	YMR104C
YEL060C	YNL161W
YEL060C	YOL133W
YEL060C	YPL140C
YEL061C	YER082C
YEL061C	YLR453C
YEL061C	YLR454W
YEL061C	YMR124W
YEL061C	YOL069W

YEL062W	YKL048C
YEL062W	YLR096W
YEL062W	YNL307C
YEL062W	YOR138C
YEL062W	YPL255W
YEL063C	YFL041W
YEL063C	YGL051W
YEL063C	YGR014W
YEL063C	YGR057C
YEL063C	YGR284C
YEL063C	YHL042W
YEL063C	YHR133C
YEL063C	YHR140W
YEL063C	YHR142W
YEL063C	YIL016W
YEL063C	YIL114C
YEL063C	YIL140W
YEL063C	YJL002C
YEL063C	YJL091C
YEL063C	YJL117W
YEL063C	YJL196C
YEL063C	YJR010C-A
YEL063C	YJR117W
YEL063C	YKL065C
YEL063C	YKL154W
YEL063C	YLR343W
YEL063C	YLR372W
YEL063C	YML048W
YEL063C	YMR264W
YEL063C	YMR292W
YEL063C	YNL125C
YEL063C	YOL132W
YEL063C	YOR016C
YEL063C	YOR161C
YEL063C	YOR254C
YEL063C	YOR345C
YEL063C	YPL076W
YEL063C	YPL139C
YEL063C	YPL227C
YEL063C	YPL234C
YEL064C	YIR034C
YEL064C	YNR058W
YEL064C	YOR242C
YEL066W	YHR041C
YEL066W	YLR347C

YEL066W	YML064C
YEL066W	YNL189W
YEL066W	YPL070W
YEL066W	YPL112C
YEL068C	YJR091C
YEL068C	YKL002W
YEL068C	YLR225C
YEL069C	YGL249W
YEL069C	YPR110C
YEL070W	YJR091C
YEL070W	YNR058W
YEL071W	YKL103C
YEL071W	YLR383W
YEL072W	YOR273C
YEL074W	YKL002W
YEL075C	YJR091C
YEL077C	YFR053C
YER002W	YGL237C
YER002W	YMR049C
YER002W	YPR016C
YER004W	YER009W
YER004W	YGR218W
YER005W	YGL212W
YER005W	YGR284C
YER005W	YJL004C
YER005W	YLR214W
YER005W	YNL044W
YER005W	YPL264C
YER006W	YER126C
YER006W	YER133W
YER006W	YGL081W
YER006W	YGR103W
YER006W	YGR220C
YER006W	YGR245C
YER006W	YHR197W
YER006W	YIL018W
YER006W	YIL035C
YER006W	YJR110W
YER006W	YKR081C
YER006W	YLR074C
YER006W	YMR049C
YER006W	YNL061W
YER006W	YNL110C
YER006W	YNL284C
YER006W	YOR267C

YER006W	YPL043W
YER006W	YPL204W
YER006W	YPR161C
YER007C-A	YJR014W
YER007C-A	YKL028W
YER007W	YHR027C
YER007W	YJL120W
YER007W	YML085C
YER007W	YOL082W
YER007W	YOR349W
YER008C	YER114C
YER008C	YLR191W
YER008C	YLR229C
YER008C	YMR304W
YER008C	YPR165W
YER009W	YGL092W
YER009W	YIL063C
YER009W	YJL041W
YER009W	YLR293C
YER009W	YLR335W
YER009W	YLR347C
YER009W	YMR059W
YER009W	YNL189W
YER009W	YOR098C
YER009W	YOR185C
YER010C	YNL189W
YER010C	YOR274W
YER011W	YKL002W
YER011W	YLR347C
YER011W	YPL178W
YER012W	YER021W
YER012W	YER094C
YER012W	YFR004W
YER012W	YFR050C
YER012W	YFR052W
YER012W	YGL011C
YER012W	YGL048C
YER012W	YGR135W
YER012W	YGR253C
YER012W	YHR027C
YER012W	YHR033W
YER012W	YHR200W
YER012W	YIL075C
YER012W	YJL001W
YER012W	YJL008C

YER012W	YKL145W
YER012W	YKL152C
YER012W	YKL206C
YER012W	YLR180W
YER012W	YLR199C
YER012W	YLR259C
YER012W	YLR421C
YER012W	YML092C
YER012W	YMR106C
YER012W	YMR314W
YER012W	YOL038W
YER012W	YOR117W
YER012W	YOR261C
YER012W	YOR362C
YER012W	YPR103W
YER012W	YPR108W
YER013W	YLR116W
YER013W	YLR117C
YER013W	YMR213W
YER015W	YJL095W
YER016W	YFR050C
YER016W	YGL061C
YER016W	YML085C
YER017C	YER091C
YER017C	YFL018C
YER017C	YGL253W
YER017C	YGR155W
YER017C	YJL034W
YER017C	YJR045C
YER017C	YKL035W
YER017C	YKR066C
YER017C	YLR044C
YER017C	YLR259C
YER017C	YMR089C
YER017C	YMR186W
YER018C	YGL086W
YER018C	YKR054C
YER018C	YMR117C
YER019C-A	YJR091C
YER019W	YER022W
YER019W	YER104W
YER019W	YPR065W
YER020W	YFR053C
YER020W	YGL121C
YER020W	YGL245W

YER020W YHR005C
YER020W YMR029C
YER020W YNL037C
YER020W YNL071W
YER020W YNL124W
YER020W YOL055C
YER020W YOR371C
YER020W YPL036W
YER020W YPL258C
YER021W YFR004W
YER021W YFR010W
YER021W YFR052W
YER021W YGL004C
YER021W YGR232W
YER021W YHR027C
YER021W YHR200W
YER021W YKL145W
YER021W YPR108W
YER021W YPR110C
YER022W YFR037C
YER022W YFR038W
YER022W YGL027C
YER022W YGL127C
YER022W YGL129C
YER022W YGR090W
YER022W YGR104C
YER022W YGR218W
YER022W YGR243W
YER022W YHL005C
YER022W YHL044W
YER022W YHL045W
YER022W YHR041C
YER022W YHR058C
YER022W YHR116W
YER022W YHR152W
YER022W YHR200W
YER022W YIL022W
YER022W YIL078W
YER022W YIL141W
YER022W YIR038C
YER022W YJL142C
YER022W YJR094C
YER022W YJR126C
YER022W YJR148W
YER022W YKL051W

YER022W YKL063C
YER022W YKL141W
YER022W YKL143W
YER022W YKR093W
YER022W YLL020C
YER022W YLL065W
YER022W YLR175W
YER022W YLR288C
YER022W YLR331C
YER022W YLR453C
YER022W YML007W
YER022W YML022W
YER022W YML055W
YER022W YML082W
YER022W YMR039C
YER022W YMR042W
YER022W YMR043W
YER022W YMR058W
YER022W YMR087W
YER022W YMR112C
YER022W YMR114C
YER022W YMR118C
YER022W YMR139W
YER022W YMR181C
YER022W YMR244W
YER022W YMR293C
YER022W YNL006W
YER022W YNL073W
YER022W YNL084C
YER022W YNL159C
YER022W YNL210W
YER022W YNL211C
YER022W YNL235C
YER022W YNL281W
YER022W YNL282W
YER022W YNL288W
YER022W YNL301C
YER022W YNR001C
YER022W YNR012W
YER022W YNR024W
YER022W YNR052C
YER022W YNR071C
YER022W YOL022C
YER022W YOL042W
YER022W YOL051W

YER022W YOL106W
YER022W YOL135C
YER022W YOR028C
YER022W YOR039W
YER022W YOR072W
YER022W YOR119C
YER022W YOR136W
YER022W YOR143C
YER022W YOR187W
YER022W YOR197W
YER022W YOR303W
YER022W YOR334W
YER022W YOR336W
YER022W YOR388C
YER022W YPL011C
YER022W YPL042C
YER022W YPL053C
YER022W YPL098C
YER022W YPL248C
YER023W YGL137W
YER023W YLR291C
YER023W YLR347C
YER023W YML064C
YER023W YNL189W
YER023W YPL070W
YER023W YPL169C
YER025W YER043C
YER025W YER091C
YER025W YER133W
YER025W YER146W
YER025W YGR052W
YER025W YGR254W
YER025W YJR007W
YER025W YKL095W
YER025W YKR026C
YER025W YLR044C
YER025W YLR215C
YER025W YLR291C
YER025W YML064C
YER025W YMR117C
YER025W YNL312W
YER025W YOL142W
YER025W YOR119C
YER025W YOR212W
YER025W YOR260W

YER025W	YPR110C
YER026C	YGR060W
YER026C	YHL042W
YER026C	YHR140W
YER026C	YJR010C-A
YER026C	YLR214W
YER026C	YLR292C
YER026C	YMR058W
YER026C	YMR153W
YER026C	YMR215W
YER026C	YNL101W
YER026C	YPL076W
YER026C	YPL274W
YER027C	YGL115W
YER027C	YGL208W
YER027C	YJL089W
YER027C	YNL183C
YER027C	YOR276W
YER028C	YIL106W
YER028C	YJL124C
YER029C	YER112W
YER029C	YER172C
YER029C	YFL017W-A
YER029C	YGL070C
YER029C	YGR074W
YER029C	YGR091W
YER029C	YHR086W
YER029C	YHR165C
YER029C	YJR022W
YER029C	YJR091C
YER029C	YLR147C
YER029C	YLR220W
YER029C	YLR298C
YER029C	YLR438C-A
YER029C	YMR240C
YER029C	YNL187W
YER029C	YNR053C
YER029C	YPR182W
YER030W	YLR186W
YER031C	YER136W
YER031C	YGL161C
YER031C	YGL198W
YER031C	YGR172C
YER031C	YJL099W
YER031C	YKL002W

YER031C	YKR030W
YER031C	YNL146W
YER031C	YNL263C
YER031C	YOR036W
YER031C	YOR070C
YER031C	YPL192C
YER031C	YPR017C
YER032W	YER114C
YER032W	YER118C
YER032W	YHR114W
YER032W	YHR152W
YER032W	YJL020C
YER032W	YJR110W
YER032W	YLR456W
YER032W	YMR032W
YER032W	YMR093W
YER032W	YNL161W
YER032W	YPR120C
YER033C	YKL129C
YER033C	YLR096W
YER033C	YLR191W
YER033C	YMR032W
YER033C	YMR109W
YER033C	YNL161W
YER036C	YGR206W
YER036C	YJR059W
YER036C	YJR091C
YER036C	YNL020C
YER036C	YNL142W
YER036C	YPR036W
YER037W	YER052C
YER038C	YIR002C
YER038C	YML023C
YER040W	YER118C
YER040W	YGL181W
YER040W	YJR066W
YER040W	YKL203C
YER040W	YMR139W
YER040W	YNL189W
YER040W	YNL229C
YER041W	YER165W
YER041W	YHL034C
YER041W	YJL130C
YER041W	YJR121W
YER041W	YKL152C

YER041W	YKR081C
YER041W	YLR449W
YER041W	YPR120C
YER043C	YER161C
YER043C	YFL033C
YER043C	YGL100W
YER043C	YGL237C
YER043C	YGR083C
YER043C	YHR135C
YER043C	YIR005W
YER043C	YJL098W
YER043C	YJL128C
YER043C	YJL164C
YER043C	YJR090C
YER043C	YKL103C
YER043C	YKL161C
YER043C	YLR175W
YER043C	YLR291C
YER043C	YLR309C
YER043C	YLR442C
YER043C	YML095C
YER043C	YML115C
YER043C	YMR049C
YER043C	YMR059W
YER043C	YNL135C
YER043C	YNL157W
YER043C	YNL189W
YER043C	YNL244C
YER043C	YOR061W
YER043C	YOR089C
YER043C	YOR212W
YER043C	YOR351C
YER043C	YPL203W
YER043C	YPR110C
YER044C	YKL002W
YER044C	YPL051W
YER045C	YGL181W
YER045C	YGR123C
YER045C	YGR247W
YER045C	YMR308C
YER045C	YOR059C
YER047C	YER095W
YER047C	YER161C
YER047C	YGL181W
YER047C	YKL129C

YER047C	YKR034W
YER047C	YLR191W
YER047C	YMR032W
YER047C	YMR109W
YER047C	YPL126W
YER047C	YPL161C
YER048C	YPL205C
YER048C	YPR020W
YER049W	YFR004W
YER049W	YGL061C
YER049W	YGL137W
YER049W	YGR271W
YER049W	YPL141C
YER051W	YER081W
YER051W	YLR121C
YER051W	YMR310C
YER052C	YGL190C
YER052C	YIR014W
YER052C	YIR018W
YER052C	YKR079C
YER052C	YLR293C
YER052C	YLR310C
YER052C	YMR139W
YER052C	YMR263W
YER052C	YNL135C
YER053C	YGL070C
YER053C	YLR326W
YER054C	YER118C
YER054C	YER133W
YER054C	YER177W
YER054C	YGL134W
YER054C	YGR052W
YER054C	YJL164C
YER054C	YKL085W
YER054C	YKL166C
YER054C	YLR258W
YER054C	YML016C
YER054C	YPL203W
YER054C	YPR160W
YER054C	YPR184W
YER055C	YPR154W
YER056C	YER120W
YER056C	YOL132W
YER057C	YNL189W
YER058W	YJR091C

YER059W	YER156C
YER059W	YFL037W
YER059W	YFL039C
YER059W	YGR109C
YER059W	YJL020C
YER059W	YJL034W
YER059W	YJL084C
YER059W	YKL103C
YER059W	YKL152C
YER059W	YLR079W
YER059W	YLR190W
YER059W	YMR165C
YER059W	YOR392W
YER059W	YPL031C
YER060W-A	YOL132W
YER061C	YPL073C
YER062C	YER171W
YER062C	YGR218W
YER062C	YHL032C
YER062C	YJL173C
YER062C	YML058W
YER062C	YOL133W
YER062C	YPL201C
YER063W	YGL181W
YER063W	YJR091C
YER063W	YKL056C
YER063W	YMR048W
YER063W	YOR333C
YER063W	YPL172C
YER064C	YLR447C
YER064C	YNL157W
YER064C	YPL212C
YER064C	YPR125W
YER065C	YNL030W
YER065C	YNL189W
YER066C-A	YGR254W
YER066C-A	YJR121W
YER066C-A	YMR066W
YER066C-A	YNL064C
YER066C-A	YOR027W
YER066W	YHR041C
YER066W	YHR147C
YER066W	YMR154C
YER066W	YPL022W
YER067W	YLR340W

YER067W	YPL180W
YER068W	YGR134W
YER068W	YIL038C
YER068W	YIL128W
YER068W	YNL288W
YER068W	YNR052C
YER068W	YPR072W
YER069W	YJL071W
YER069W	YLR214W
YER070W	YHR169W
YER070W	YIL066C
YER070W	YML058W
YER070W	YML064C
YER071C	YKL007W
YER071C	YLR200W
YER071C	YMR048W
YER071C	YOR171C
YER071C	YOR176W
YER071C	YPL032C
YER073W	YGL181W
YER073W	YML032C
YER073W	YPL257W
YER073W	YPR086W
YER075C	YER177W
YER075C	YFL039C
YER075C	YGL206C
YER075C	YHR030C
YER075C	YJR045C
YER075C	YLR113W
YER075C	YMR229C
YER075C	YMR319C
YER075C	YNL030W
YER076C	YPR086W
YER077C	YFR015C
YER077C	YIL110W
YER077C	YLR233C
YER077C	YMR106C
YER077C	YNL230C
YER078C	YER081W
YER078C	YFL027C
YER078C	YGR091W
YER078C	YML036W
YER079W	YHR114W
YER079W	YHR135C
YER079W	YKL002W

YER079W YNL154C
YER081W YER145C
YER081W YFL016C
YER081W YFL037W
YER081W YFR017C
YER081W YFR053C
YER081W YFR055W
YER081W YGL039W
YER081W YGL096W
YER081W YGL110C
YER081W YGL181W
YER081W YGR020C
YER081W YGR084C
YER081W YGR180C
YER081W YGR210C
YER081W YGR293C
YER081W YHR215W
YER081W YIL044C
YER081W YIL074C
YER081W YJR108W
YER081W YKR073C
YER081W YLR151C
YER081W YNR042W
YER081W YOR150W
YER081W YOR167C
YER081W YOR189W
YER081W YOR222W
YER081W YOR246C
YER081W YOR258W
YER081W YOR263C
YER081W YOR300W
YER081W YOR318C
YER081W YOR362C
YER081W YOR387C
YER081W YOR388C
YER081W YOR392W
YER081W YPL205C
YER081W YPL213W
YER081W YPL220W
YER081W YPL229W
YER081W YPL233W
YER081W YPL234C
YER081W YPR016C
YER081W YPR046W
YER081W YPR126C

YER081W	YPR134W
YER081W	YPR136C
YER081W	YPR137C-B
YER081W	YPR149W
YER082C	YER118C
YER082C	YER161C
YER082C	YER165W
YER082C	YGL068W
YER082C	YGL173C
YER082C	YGR090W
YER082C	YGR128C
YER082C	YGR162W
YER082C	YHL034C
YER082C	YIL142W
YER082C	YJL014W
YER082C	YJL109C
YER082C	YJL111W
YER082C	YJL135W
YER082C	YJR002W
YER082C	YKL056C
YER082C	YLR129W
YER082C	YLR180W
YER082C	YLR197W
YER082C	YLR222C
YER082C	YLR259C
YER082C	YLR409C
YER082C	YMR012W
YER082C	YMR093W
YER082C	YMR205C
YER082C	YNL132W
YER082C	YOL077C
YER082C	YPL012W
YER082C	YPL126W
YER082C	YPL212C
YER082C	YPL217C
YER082C	YPR112C
YER083C	YER126C
YER083C	YMR038C
YER083C	YPR175W
YER084W	YIL035C
YER084W	YKL002W
YER086W	YER095W
YER086W	YHL004W
YER086W	YJL020C
YER086W	YKL057C

YER086W	YKL081W
YER086W	YLR191W
YER086W	YMR109W
YER086W	YOR383C
YER086W	YPL070W
YER087C-B	YJL091C
YER088C	YLR103C
YER089C	YIL033C
YER089C	YPL153C
YER090W	YKL211C
YER090W	YLR180W
YER090W	YOL139C
YER090W	YOR155C
YER091C	YER133W
YER091C	YFL033C
YER091C	YGL100W
YER091C	YGL190C
YER091C	YGR092W
YER091C	YHL007C
YER091C	YHR107C
YER091C	YHR135C
YER091C	YKL103C
YER091C	YKR055W
YER091C	YLR175W
YER091C	YLR291C
YER091C	YLR314C
YER091C	YLR383W
YER091C	YML057W
YER091C	YML064C
YER091C	YMR049C
YER091C	YMR059W
YER091C	YMR246W
YER091C	YNL135C
YER091C	YNL189W
YER091C	YNL244C
YER091C	YOR089C
YER091C	YOR212W
YER091C	YPL031C
YER091C	YPL149W
YER091C	YPR110C
YER091C	YPR178W
YER092W	YKL002W
YER092W	YLR052W
YER092W	YLR423C
YER093C	YGR040W

YER093C	YJL058C
YER093C	YKL126W
YER093C	YKL203C
YER093C	YLR210W
YER093C	YNL006W
YER093C	YNL030W
YER094C	YGL011C
YER094C	YHR200W
YER094C	YML092C
YER094C	YMR106C
YER094C	YOR157C
YER094C	YPR103W
YER095W	YER110C
YER095W	YFR009W
YER095W	YGL163C
YER095W	YGL245W
YER095W	YGR234W
YER095W	YGR240C
YER095W	YJR109C
YER095W	YKL104C
YER095W	YLR337C
YER095W	YLR394W
YER095W	YML032C
YER095W	YMR012W
YER095W	YMR167W
YER095W	YMR186W
YER095W	YMR233W
YER095W	YNL085W
YER095W	YNL189W
YER095W	YOR151C
YER095W	YPL204W
YER095W	YPL238C
YER095W	YPR010C
YER095W	YPR011C
YER096W	YHR114W
YER096W	YPR165W
YER099C	YKL181W
YER099C	YMR139W
YER099C	YOL061W
YER099C	YOL069W
YER100W	YER110C
YER100W	YFL037W
YER100W	YFL038C
YER100W	YFR053C
YER100W	YGL195W

YER100W	YHR082C
YER100W	YHR129C
YER100W	YJL130C
YER100W	YJR077C
YER100W	YKL056C
YER100W	YKL205W
YER100W	YKL210W
YER100W	YML085C
YER100W	YPL078C
YER100W	YPL229W
YER100W	YPL235W
YER103W	YER117W
YER103W	YFR021W
YER103W	YGL065C
YER103W	YGL130W
YER103W	YGL137W
YER103W	YGL190C
YER103W	YHR199C
YER103W	YIL142W
YER103W	YLR175W
YER103W	YNL007C
YER103W	YNL077W
YER103W	YNL135C
YER103W	YOL062C
YER103W	YOR039W
YER103W	YPL259C
YER103W	YPR001W
YER103W	YPR110C
YER104W	YKL002W
YER104W	YNL106C
YER105C	YFR050C
YER105C	YGL092W
YER105C	YIL061C
YER105C	YJL030W
YER105C	YJL061W
YER105C	YMR153W
YER105C	YNR047W
YER105C	YPR120C
YER106W	YPL204W
YER107C	YGL143C
YER107C	YHL008C
YER107C	YMR047C
YER107C	YNL189W
YER107C	YPR119W
YER109C	YGL070C

YER110C	YER117W
YER110C	YFR028C
YER110C	YGL172W
YER110C	YGL237C
YER110C	YGR067C
YER110C	YGR119C
YER110C	YHR102W
YER110C	YHR169W
YER110C	YHR199C
YER110C	YIL026C
YER110C	YJR017C
YER110C	YJR068W
YER110C	YKL068W
YER110C	YKL081W
YER110C	YLR148W
YER110C	YLR293C
YER110C	YML007W
YER110C	YMR001C
YER110C	YMR036C
YER110C	YMR047C
YER110C	YMR106C
YER110C	YNL030W
YER110C	YNL189W
YER110C	YNL313C
YER110C	YNL323W
YER110C	YOL127W
YER110C	YOL133W
YER110C	YPL203W
YER110C	YPL259C
YER110C	YPR017C
YER111C	YER167W
YER111C	YHR030C
YER111C	YLR182W
YER111C	YPL153C
YER111C	YPL256C
YER111C	YPR119W
YER112W	YER124C
YER112W	YER146W
YER112W	YER172C
YER112W	YFL017W-A
YER112W	YGL173C
YER112W	YIL029C
YER112W	YJL110C
YER112W	YJL124C
YER112W	YJL155C

YER112W	YJR022W
YER112W	YJR138W
YER112W	YKL209C
YER112W	YLL032C
YER112W	YLR147C
YER112W	YLR264W
YER112W	YLR269C
YER112W	YLR275W
YER112W	YLR386W
YER112W	YLR438C-A
YER112W	YMR304W
YER112W	YNL091W
YER112W	YNL118C
YER112W	YNL147W
YER112W	YNL199C
YER112W	YOL004W
YER112W	YOL149W
YER112W	YOR039W
YER112W	YOR195W
YER112W	YOR219C
YER112W	YPR132W
YER113C	YER118C
YER113C	YGR123C
YER113C	YIL120W
YER114C	YER118C
YER114C	YER124C
YER114C	YER155C
YER114C	YER158C
YER114C	YER177W
YER114C	YGL190C
YER114C	YGR033C
YER114C	YHL007C
YER114C	YHL014C
YER114C	YHR066W
YER114C	YIL144W
YER114C	YIR006C
YER114C	YIR039C
YER114C	YJR091C
YER114C	YJR138W
YER114C	YLR056W
YER114C	YLR086W
YER114C	YLR229C
YER114C	YML109W
YER114C	YMR080C
YER114C	YMR124W

YER114C	YMR244W
YER114C	YNL078W
YER114C	YNL298W
YER114C	YOR188W
YER114C	YOR370C
YER114C	YPL038W
YER114C	YPL049C
YER114C	YPL158C
YER114C	YPL272C
YER115C	YGL070C
YER115C	YJL020C
YER115C	YLR310C
YER115C	YML109W
YER115C	YOL003C
YER116C	YLR295C
YER117W	YFL037W
YER117W	YHR114W
YER117W	YKL104C
YER117W	YLR259C
YER117W	YML056C
YER117W	YML085C
YER117W	YMR319C
YER117W	YOL098C
YER117W	YOR206W
YER117W	YPL208W
YER117W	YPL258C
YER118C	YFL062W
YER118C	YGL085W
YER118C	YGL162W
YER118C	YGL173C
YER118C	YGL201C
YER118C	YGR178C
YER118C	YGR257C
YER118C	YGR295C
YER118C	YHL002W
YER118C	YHL007C
YER118C	YHL048W
YER118C	YHR140W
YER118C	YHR143W
YER118C	YIL045W
YER118C	YIL095W
YER118C	YIL144W
YER118C	YIL156W
YER118C	YIR003W
YER118C	YJL062W

YER118C	YJL128C
YER118C	YJR115W
YER118C	YKL221W
YER118C	YKR027W
YER118C	YLL048C
YER118C	YLR096W
YER118C	YLR138W
YER118C	YLR292C
YER118C	YLR314C
YER118C	YLR372W
YER118C	YLR425W
YER118C	YLR452C
YER118C	YMR032W
YER118C	YMR065W
YER118C	YMR068W
YER118C	YMR109W
YER118C	YMR153W
YER118C	YNL049C
YER118C	YNL101W
YER118C	YNL106C
YER118C	YNL112W
YER118C	YNL139C
YER118C	YNL152W
YER118C	YNL331C
YER118C	YNR033W
YER118C	YNR064C
YER118C	YOL070C
YER118C	YOR127W
YER118C	YOR181W
YER118C	YOR254C
YER118C	YOR311C
YER118C	YPL115C
YER118C	YPR171W
YER119C	YGL127C
YER119C	YKL055C
YER119C	YMR047C
YER120W	YGR086C
YER120W	YHL020C
YER120W	YHR196W
YER120W	YHR200W
YER120W	YLR305C
YER120W	YLR342W
YER120W	YMR049C
YER120W	YMR216C
YER120W	YNL027W

YER121W	YKL002W
YER122C	YER176W
YER122C	YGL137W
YER122C	YHR148W
YER122C	YJL030W
YER122C	YJR132W
YER122C	YNL287W
YER123W	YHR135C
YER123W	YNL049C
YER123W	YOL083W
YER124C	YHR003C
YER124C	YML109W
YER125W	YFL039C
YER125W	YFR022W
YER125W	YFR028C
YER125W	YGL087C
YER125W	YGR005C
YER125W	YGR052W
YER125W	YGR068C
YER125W	YGR136W
YER125W	YGR186W
YER125W	YGR268C
YER125W	YHR097C
YER125W	YHR114W
YER125W	YHR143W-A
YER125W	YIL021W
YER125W	YJL034W
YER125W	YJL084C
YER125W	YJL140W
YER125W	YJL151C
YER125W	YJR091C
YER125W	YKL020C
YER125W	YKR018C
YER125W	YKR021W
YER125W	YLR342W
YER125W	YLR392C
YER125W	YML010W
YER125W	YML111W
YER125W	YML123C
YER125W	YMR171C
YER125W	YMR275C
YER125W	YMR316W
YER125W	YNL071W
YER125W	YNL183C
YER125W	YNL243W

YER125W	YOL005C
YER125W	YOL055C
YER125W	YOR018W
YER125W	YOR151C
YER125W	YOR181W
YER125W	YOR210W
YER125W	YOR224C
YER125W	YOR322C
YER125W	YOR340C
YER125W	YOR385W
YER125W	YPL258C
YER125W	YPR030W
YER125W	YPR084W
YER125W	YPR187W
YER126C	YGL111W
YER126C	YGL237C
YER126C	YGR245C
YER126C	YHR066W
YER126C	YHR197W
YER126C	YHR204W
YER126C	YKL009W
YER126C	YKR081C
YER126C	YLR074C
YER126C	YMR049C
YER126C	YMR290C
YER126C	YNL002C
YER126C	YNL061W
YER126C	YNL110C
YER126C	YNR053C
YER126C	YOR206W
YER126C	YPL093W
YER126C	YPR016C
YER127W	YGL061C
YER127W	YJL069C
YER127W	YLR423C
YER127W	YOL010W
YER128W	YPR173C
YER129W	YER155C
YER129W	YFR028C
YER129W	YGL019W
YER129W	YGL115W
YER129W	YIL035C
YER129W	YIL144W
YER129W	YKL042W
YER129W	YLR420W

YER129W	YNL225C
YER129W	YOR061W
YER129W	YOR373W
YER129W	YPL204W
YER131W	YER146W
YER131W	YJL020C
YER131W	YLL027W
YER131W	YLR435W
YER132C	YER177W
YER132C	YGR052W
YER132C	YLR310C
YER132C	YMR139W
YER132C	YNL025C
YER132C	YNL307C
YER132C	YPL203W
YER132C	YPL204W
YER133W	YER167W
YER133W	YER177W
YER133W	YFL018C
YER133W	YFL023W
YER133W	YFR001W
YER133W	YFR003C
YER133W	YFR015C
YER133W	YFR028C
YER133W	YFR053C
YER133W	YGL111W
YER133W	YGL245W
YER133W	YGL253W
YER133W	YGL256W
YER133W	YGR052W
YER133W	YGR097W
YER133W	YGR103W
YER133W	YGR156W
YER133W	YGR238C
YER133W	YHR052W
YER133W	YHR100C
YER133W	YHR158C
YER133W	YIL033C
YER133W	YIL045W
YER133W	YIL070C
YER133W	YIL154C
YER133W	YIR006C
YER133W	YJL033W
YER133W	YJL034W
YER133W	YJL042W

YER133W YJL052W
YER133W YJL153C
YER133W YJL187C
YER133W YJR007W
YER133W YJR009C
YER133W YJR093C
YER133W YKL018W
YER133W YKL035W
YER133W YKL059C
YER133W YKL060C
YER133W YKL085W
YER133W YKL193C
YER133W YKR002W
YER133W YKR081C
YER133W YLR002C
YER133W YLR044C
YER133W YLR096W
YER133W YLR115W
YER133W YLR134W
YER133W YLR215C
YER133W YLR258W
YER133W YLR259C
YER133W YLR263W
YER133W YLR277C
YER133W YLR355C
YER133W YLR430W
YER133W YLR449W
YER133W YML074C
YER133W YMR001C
YER133W YMR012W
YER133W YMR049C
YER133W YMR186W
YER133W YMR290C
YER133W YMR311C
YER133W YNL002C
YER133W YNL061W
YER133W YNL064C
YER133W YNL233W
YER133W YOL055C
YER133W YOL091W
YER133W YOR078W
YER133W YOR178C
YER133W YOR179C
YER133W YOR227W
YER133W YOR272W

YER133W	YOR294W
YER133W	YOR315W
YER133W	YOR329C
YER133W	YOR351C
YER133W	YPL061W
YER133W	YPL093W
YER133W	YPL137C
YER133W	YPL204W
YER133W	YPL211W
YER133W	YPL237W
YER133W	YPR016C
YER133W	YPR107C
YER133W	YPR160W
YER136W	YFL005W
YER136W	YFL038C
YER136W	YGL210W
YER136W	YIR009W
YER136W	YJR035W
YER136W	YKR014C
YER136W	YLR262C
YER136W	YLR362W
YER136W	YML001W
YER136W	YMR047C
YER136W	YNL135C
YER136W	YOL055C
YER136W	YOR089C
YER136W	YPR180W
YER137C	YMR025W
YER138C	YMR139W
YER138C	YNL307C
YER138C	YPL204W
YER138C	YPR110C
YER139C	YKR081C
YER139C	YPL203W
YER140W	YGR136W
YER140W	YKL056C
YER140W	YPR154W
YER142C	YER165W
YER142C	YGL049C
YER142C	YGL173C
YER142C	YGR090W
YER142C	YGR162W
YER142C	YHR216W
YER142C	YIR002C
YER142C	YJL088W

YER142C	YLR432W
YER142C	YML056C
YER142C	YOL041C
YER142C	YOL090W
YER142C	YOR207C
YER142C	YPL061W
YER142C	YPL127C
YER142C	YPR190C
YER143W	YGL044C
YER143W	YIL148W
YER143W	YJR141W
YER143W	YMR055C
YER143W	YNR071C
YER144C	YMR032W
YER144C	YOR034C
YER144C	YOR157C
YER145C	YIL056W
YER145C	YJL001W
YER146W	YFL066C
YER146W	YGR210C
YER146W	YHL008C
YER146W	YIL038C
YER146W	YIL048W
YER146W	YJL084C
YER146W	YJL124C
YER146W	YJR022W
YER146W	YJR138W
YER146W	YKL173W
YER146W	YKL209C
YER146W	YKR026C
YER146W	YLR058C
YER146W	YLR147C
YER146W	YLR275W
YER146W	YLR438C-A
YER146W	YMR268C
YER146W	YNL147W
YER146W	YOL149W
YER146W	YOR201C
YER146W	YOR320C
YER146W	YPL090C
YER146W	YPL152W
YER146W	YPR010C
YER146W	YPR178W
YER146W	YPR184W
YER147C	YHR168W

YER148W	YFR034C
YER148W	YGL048C
YER148W	YGL112C
YER148W	YGL241W
YER148W	YGR104C
YER148W	YGR246C
YER148W	YGR274C
YER148W	YHR041C
YER148W	YIL021W
YER148W	YIR005W
YER148W	YKL058W
YER148W	YKR001C
YER148W	YMR205C
YER148W	YMR270C
YER148W	YMR308C
YER148W	YNL039W
YER148W	YOL148C
YER148W	YOR047C
YER148W	YOR117W
YER148W	YOR194C
YER148W	YPL082C
YER148W	YPL122C
YER148W	YPL248C
YER148W	YPR072W
YER148W	YPR086W
YER149C	YGL127C
YER149C	YIL040W
YER149C	YJL074C
YER149C	YKL034W
YER149C	YLL021W
YER149C	YMR124W
YER149C	YOR315W
YER151C	YGL011C
YER151C	YML092C
YER151C	YML109W
YER151C	YNR051C
YER152C	YJL107C
YER153C	YGR222W
YER153C	YJL170C
YER154W	YNR003C
YER154W	YOR121C
YER154W	YPR086W
YER155C	YGL019W
YER155C	YGL130W
YER155C	YGR080W

YER155C	YIL113W
YER155C	YJL098W
YER155C	YNL061W
YER155C	YNL201C
YER155C	YNL207W
YER155C	YOL087C
YER155C	YOR014W
YER155C	YOR061W
YER155C	YPL042C
YER155C	YPR088C
YER156C	YFL038C
YER156C	YOR035C
YER157W	YFL038C
YER157W	YGL223C
YER157W	YGR120C
YER157W	YHL031C
YER157W	YKL196C
YER157W	YLR026C
YER157W	YLR268W
YER157W	YLR295C
YER157W	YNL041C
YER157W	YNL287W
YER157W	YPR105C
YER158C	YKL120W
YER158C	YPL161C
YER159C	YHR114W
YER159C-A	YJL098W
YER159C-A	YPL253C
YER160C	YMR032W
YER160C	YMR139W
YER160C	YNL307C
YER161C	YER165W
YER161C	YER177W
YER161C	YFL018C
YER161C	YFL039C
YER161C	YGL019W
YER161C	YGL181W
YER161C	YGL201C
YER161C	YGR090W
YER161C	YGR155W
YER161C	YGR162W
YER161C	YHL034C
YER161C	YHR066W
YER161C	YHR166C
YER161C	YHR183W

YER161C	YHR216W
YER161C	YIL035C
YER161C	YIR001C
YER161C	YJR045C
YER161C	YJR121W
YER161C	YKR081C
YER161C	YLR259C
YER161C	YLR293C
YER161C	YLR389C
YER161C	YMR205C
YER161C	YNL183C
YER161C	YNL189W
YER161C	YOL041C
YER161C	YOR061W
YER161C	YPL125W
YER162C	YFR021W
YER162C	YHL025W
YER162C	YIL148W
YER162C	YJR052W
YER162C	YLR167W
YER162C	YMR201C
YER163C	YOL061W
YER164W	YGL019W
YER164W	YIL035C
YER164W	YKR001C
YER164W	YNL030W
YER164W	YOR039W
YER164W	YOR061W
YER164W	YOR207C
YER164W	YOR304W
YER164W	YOR319W
YER165W	YGL035C
YER165W	YGL044C
YER165W	YGL049C
YER165W	YGL172W
YER165W	YGR103W
YER165W	YGR119C
YER165W	YGR162W
YER165W	YGR178C
YER165W	YHR052W
YER165W	YHR073W
YER165W	YIL035C
YER165W	YIL079C
YER165W	YIL095W
YER165W	YIL113W

YER165W YIR001C
YER165W YIR005W
YER165W YJR076C
YER165W YKL068W
YER165W YKL139W
YER165W YLL013C
YER165W YLR074C
YER165W YLR233C
YER165W YLR335W
YER165W YLR362W
YER165W YLR427W
YER165W YMR047C
YER165W YMR061W
YER165W YMR093W
YER165W YMR137C
YER165W YNL088W
YER165W YNL093W
YER165W YNL161W
YER165W YNL175C
YER165W YNL230C
YER165W YNL255C
YER165W YOL006C
YER165W YOL054W
YER165W YOL102C
YER165W YOL108C
YER165W YOL115W
YER165W YOL139C
YER165W YOR080W
YER165W YOR098C
YER165W YPL169C
YER165W YPL204W
YER165W YPL259C
YER165W YPR017C
YER165W YPR159W
YER166W YGL200C
YER166W YIR006C
YER166W YNR047W
YER167W YER177W
YER167W YGR040W
YER167W YGR229C
YER167W YGR238C
YER167W YHR158C
YER167W YLR096W
YER167W YOL108C
YER167W YPL204W

YER168C	YHR174W
YER168C	YPL022W
YER169W	YPL071C
YER170W	YKL002W
YER171W	YER177W
YER171W	YFL016C
YER171W	YFL037W
YER171W	YFR009W
YER171W	YFR053C
YER171W	YGL048C
YER171W	YGL195W
YER171W	YGR282C
YER171W	YHL030W
YER171W	YHR027C
YER171W	YHR200W
YER171W	YIL053W
YER171W	YIL128W
YER171W	YIL143C
YER171W	YJL008C
YER171W	YJL034W
YER171W	YJL066C
YER171W	YJL130C
YER171W	YJR035W
YER171W	YJR045C
YER171W	YJR121W
YER171W	YKL085W
YER171W	YKL152C
YER171W	YLR005W
YER171W	YLR259C
YER171W	YLR304C
YER171W	YML085C
YER171W	YML124C
YER171W	YMR012W
YER171W	YMR056C
YER171W	YMR116C
YER171W	YMR201C
YER171W	YNL064C
YER171W	YOL055C
YER171W	YOL090W
YER171W	YOR136W
YER171W	YOR142W
YER171W	YOR261C
YER171W	YOR374W
YER171W	YPL235W
YER171W	YPL240C

YER171W YPR025C
YER171W YPR121W
YER172C YGR074W
YER172C YGR075C
YER172C YGR091W
YER172C YHR156C
YER172C YHR165C
YER172C YIL061C
YER172C YJR022W
YER172C YJR068W
YER172C YKL173W
YER172C YKR086W
YER172C YLR147C
YER172C YLR310C
YER172C YLR438C-A
YER172C YMR240C
YER172C YNR053C
YER172C YOR308C
YER172C YPL105C
YER172C YPR082C
YER172C YPR178W
YER173W YER177W
YER173W YFL016C
YER173W YFL037W
YER173W YFL039C
YER173W YGR282C
YER173W YHR079C-A
YER173W YJL008C
YER173W YJL014W
YER173W YJL034W
YER173W YJL111W
YER173W YJR045C
YER173W YJR068W
YER173W YJR072C
YER173W YJR121W
YER173W YLR180W
YER173W YLR259C
YER173W YLR413W
YER173W YML085C
YER173W YNL064C
YER173W YNL290W
YER173W YOL055C
YER173W YOR151C
YER173W YPL061W
YER173W YPL178W

YER173W	YPL194W
YER173W	YPL235W
YER173W	YPL240C
YER174C	YGL220W
YER174C	YGR262C
YER174C	YML036W
YER175C	YLR158C
YER177W	YFL033C
YER177W	YFR016C
YER177W	YFR017C
YER177W	YFR028C
YER177W	YFR040W
YER177W	YGL003C
YER177W	YGL100W
YER177W	YGL115W
YER177W	YGL163C
YER177W	YGL237C
YER177W	YGR083C
YER177W	YGR097W
YER177W	YHL007C
YER177W	YHR082C
YER177W	YHR135C
YER177W	YHR169W
YER177W	YHR196W
YER177W	YIL028W
YER177W	YIL035C
YER177W	YIL147C
YER177W	YIL159W
YER177W	YIR003W
YER177W	YJL042W
YER177W	YJL076W
YER177W	YJL138C
YER177W	YJL173C
YER177W	YJR068W
YER177W	YKL018W
YER177W	YKL103C
YER177W	YKL130C
YER177W	YKL140W
YER177W	YKL168C
YER177W	YKL171W
YER177W	YKL193C
YER177W	YKR026C
YER177W	YLL013C
YER177W	YLR175W
YER177W	YLR233C

YER177W YLR258W
YER177W YLR310C
YER177W YLR383W
YER177W YML058W
YER177W YML064C
YER177W YMR055C
YER177W YMR059W
YER177W YMR106C
YER177W YMR137C
YER177W YNL012W
YER177W YNL094W
YER177W YNL189W
YER177W YNL307C
YER177W YNR031C
YER177W YOL062C
YER177W YOL128C
YER177W YOR089C
YER177W YOR351C
YER177W YPL022W
YER177W YPL031C
YER177W YPL032C
YER177W YPL150W
YER177W YPL204W
YER177W YPR008W
YER177W YPR030W
YER177W YPR110C
YER177W YPR111W
YER177W YPR115W
YER178W YGL137W
YER178W YGL237C
YER178W YGL253W
YER178W YGR229C
YER178W YHR030C
YER178W YHR107C
YER178W YHR169W
YER178W YJL005W
YER178W YJL128C
YER178W YJR091C
YER178W YKL095W
YER178W YKL113C
YER178W YKL124W
YER178W YLL019C
YER178W YML064C
YER178W YML095C
YER178W YMR308C

YER178W	YNL157W
YER178W	YOL108C
YER178W	YPL111W
YER179W	YFL016C
YER179W	YFL037W
YER179W	YFR030W
YER179W	YGL206C
YER179W	YGR085C
YER179W	YGR254W
YER179W	YHR020W
YER179W	YIL105C
YER179W	YJL008C
YER179W	YJL125C
YER179W	YJR045C
YER179W	YJR121W
YER179W	YKL060C
YER179W	YKL081W
YER179W	YKL104C
YER179W	YKL211C
YER179W	YLR127C
YER179W	YLR259C
YER179W	YML028W
YER179W	YML064C
YER179W	YML085C
YER179W	YML124C
YER179W	YMR108W
YER179W	YNL013C
YER179W	YNL037C
YER179W	YNL055C
YER179W	YNL064C
YER179W	YNL189W
YER179W	YNR016C
YER179W	YOR136W
YER179W	YOR191W
YER179W	YOR285W
YER179W	YOR375C
YER179W	YPL235W
YER179W	YPR183W
YER180C	YGL026C
YER180C	YGL070C
YER181C	YGL070C
YER184C	YGL127C
YER186C	YKR026C
YER188W	YLR452C
YFL002C	YJR082C

YFL002C	YKR081C
YFL002C	YMR049C
YFL002C	YNL061W
YFL003C	YMR125W
YFL004W	YGR136W
YFL004W	YPR154W
YFL005W	YFL038C
YFL005W	YGL161C
YFL005W	YGL198W
YFL005W	YNL263C
YFL005W	YNL272C
YFL005W	YPR017C
YFL007W	YGL011C
YFL007W	YLR074C
YFL007W	YPR103W
YFL008W	YFR031C
YFL008W	YGL086W
YFL008W	YGR089W
YFL008W	YGR130C
YFL008W	YIL026C
YFL008W	YIL144W
YFL008W	YIR017C
YFL008W	YJL074C
YFL008W	YJR112W
YFL008W	YKL042W
YFL008W	YKR054C
YFL008W	YLR196W
YFL008W	YLR259C
YFL008W	YMR001C
YFL008W	YMR117C
YFL008W	YNL084C
YFL008W	YNL250W
YFL008W	YOL069W
YFL008W	YOL115W
YFL008W	YPR141C
YFL009W	YGL170C
YFL009W	YJL194W
YFL009W	YKL159C
YFL009W	YLR079W
YFL009W	YOL133W
YFL009W	YOR057W
YFL010C	YFR024C-A
YFL010C	YHR016C
YFL010C	YML064C
YFL010C	YNL189W

YFL010C	YOL106W
YFL010C	YPL059W
YFL010C	YPR054W
YFL011W	YJL222W
YFL011W	YMR004W
YFL012W	YHR114W
YFL012W	YPL111W
YFL013C	YLR453C
YFL013C	YOR264W
YFL013C	YPL235W
YFL013C	YPR086W
YFL014W	YFR053C
YFL014W	YMR066W
YFL016C	YGL137W
YFL016C	YGL208W
YFL016C	YHL045W
YFL016C	YHR030C
YFL016C	YHR082C
YFL016C	YIL061C
YFL016C	YJL106W
YFL016C	YJR045C
YFL016C	YKL189W
YFL016C	YKL193C
YFL016C	YKR036C
YFL016C	YMR106C
YFL016C	YMR205C
YFL016C	YNL182C
YFL016C	YNL312W
YFL016C	YOL133W
YFL016C	YOR232W
YFL016C	YPR054W
YFL016C	YPR178W
YFL017C	YGL070C
YFL017C	YNL189W
YFL017C	YOL059W
YFL017C	YOR362C
YFL017W-A	YGR013W
YFL017W-A	YGR074W
YFL017W-A	YGR091W
YFL017W-A	YIL061C
YFL017W-A	YJL203W
YFL017W-A	YJR084W
YFL017W-A	YKL012W
YFL017W-A	YKL173W
YFL017W-A	YLL036C

YFL017W-A	YLR117C
YFL017W-A	YLR147C
YFL017W-A	YLR275W
YFL017W-A	YLR298C
YFL017W-A	YLR424W
YFL017W-A	YML049C
YFL017W-A	YMR125W
YFL017W-A	YMR240C
YFL017W-A	YMR288W
YFL017W-A	YOR308C
YFL017W-A	YPL151C
YFL017W-A	YPL213W
YFL017W-A	YPR057W
YFL017W-A	YPR082C
YFL017W-A	YPR178W
YFL018C	YGL063W
YFL018C	YGL081W
YFL018C	YGL163C
YFL018C	YGL220W
YFL018C	YGR040W
YFL018C	YGR092W
YFL018C	YGR123C
YFL018C	YGR262C
YFL018C	YHR107C
YFL018C	YHR114W
YFL018C	YHR135C
YFL018C	YIL066C
YFL018C	YIL125W
YFL018C	YJL069C
YFL018C	YJL092W
YFL018C	YJL098W
YFL018C	YJL128C
YFL018C	YJR035W
YFL018C	YJR051W
YFL018C	YLL019C
YFL018C	YML064C
YFL018C	YMR059W
YFL018C	YMR106C
YFL018C	YNL113W
YFL018C	YNL175C
YFL018C	YNL182C
YFL018C	YOL094C
YFL018C	YOL123W
YFL018C	YOL133W
YFL018C	YOR005C

YFL018C	YOR180C
YFL018C	YOR386W
YFL018C	YPL150W
YFL020C	YGR072W
YFL020C	YML038C
YFL021W	YGL011C
YFL021W	YJR066W
YFL021W	YJR082C
YFL021W	YKL161C
YFL021W	YLR068W
YFL021W	YNL258C
YFL022C	YGR169C
YFL022C	YKR026C
YFL022C	YLR060W
YFL022C	YMR059W
YFL022C	YMR284W
YFL023W	YLR200W
YFL023W	YLR243W
YFL024C	YFL039C
YFL024C	YGR002C
YFL024C	YHR099W
YFL024C	YJL081C
YFL024C	YJL098W
YFL024C	YNL030W
YFL024C	YNL107W
YFL024C	YOL012C
YFL024C	YOR119C
YFL024C	YOR244W
YFL024C	YPR023C
YFL025C	YGL053W
YFL025C	YLR083C
YFL026W	YGR141W
YFL026W	YHR016C
YFL026W	YNL217W
YFL026W	YNL307C
YFL026W	YOR242C
YFL026W	YPL189W
YFL027C	YHR115C
YFL027C	YKL195W
YFL029C	YGR080W
YFL029C	YPR054W
YFL029C	YPR161C
YFL030W	YIL077C
YFL030W	YKR007W
YFL030W	YPL031C

YFL033C	YGL062W
YFL033C	YGR254W
YFL033C	YJL164C
YFL033C	YLR096W
YFL033C	YLR259C
YFL033C	YMR186W
YFL033C	YPL031C
YFL033C	YPL204W
YFL034C-B	YFR053C
YFL034C-B	YGL070C
YFL034C-B	YIR016W
YFL034C-B	YKL002W
YFL034C-B	YKL035W
YFL034C-B	YKL152C
YFL034C-B	YLR096W
YFL034C-B	YNL161W
YFL034C-B	YOL036W
YFL034C-B	YOR259C
YFL034C-B	YPL240C
YFL034W	YKL103C
YFL034W	YPL110C
YFL036W	YMR228W
YFL036W	YMR238W
YFL037W	YGL116W
YFL037W	YGL131C
YFL037W	YGL163C
YFL037W	YGL213C
YFL037W	YGL237C
YFL037W	YGR040W
YFL037W	YGR092W
YFL037W	YGR262C
YFL037W	YIL046W
YFL037W	YIL066C
YFL037W	YJL106W
YFL037W	YJL173C
YFL037W	YKR026C
YFL037W	YLL019C
YFL037W	YLR148W
YFL037W	YLR186W
YFL037W	YLR238W
YFL037W	YLR291C
YFL037W	YML064C
YFL037W	YML085C
YFL037W	YML112W
YFL037W	YML115C

YFL037W	YMR106C
YFL037W	YMR284W
YFL037W	YNL012W
YFL037W	YNL061W
YFL037W	YNL090W
YFL037W	YNL113W
YFL037W	YNL161W
YFL037W	YNL182C
YFL037W	YNL223W
YFL037W	YNL290W
YFL037W	YNL311C
YFL037W	YOL087C
YFL037W	YOL115W
YFL037W	YOL133W
YFL037W	YOR005C
YFL037W	YOR181W
YFL037W	YOR265W
YFL037W	YOR276W
YFL037W	YOR319W
YFL037W	YOR349W
YFL037W	YPL140C
YFL037W	YPL149W
YFL037W	YPL150W
YFL037W	YPL164C
YFL037W	YPL194W
YFL037W	YPL204W
YFL037W	YPR110C
YFL037W	YPR111W
YFL038C	YGL070C
YFL038C	YGL161C
YFL038C	YGL198W
YFL038C	YGR120C
YFL038C	YGR172C
YFL038C	YJR045C
YFL038C	YKL166C
YFL038C	YLR078C
YFL038C	YLR268W
YFL038C	YNL263C
YFL038C	YOL133W
YFL038C	YOR036W
YFL038C	YOR370C
YFL038C	YPL061W
YFL038C	YPL246C
YFL038C	YPR017C
YFL038C	YPR086W

YFL039C	YFR016C
YFL039C	YFR028C
YFL039C	YGL137W
YFL039C	YGL240W
YFL039C	YGR002C
YFL039C	YGR040W
YFL039C	YGR067C
YFL039C	YGR080W
YFL039C	YGR083C
YFL039C	YGR262C
YFL039C	YHL007C
YFL039C	YHR023W
YFL039C	YHR030C
YFL039C	YHR070W
YFL039C	YHR082C
YFL039C	YHR135C
YFL039C	YHR169W
YFL039C	YHR179W
YFL039C	YIL063C
YFL039C	YIL066C
YFL039C	YIL095W
YFL039C	YIL128W
YFL039C	YIL131C
YFL039C	YIL138C
YFL039C	YJL005W
YFL039C	YJL081C
YFL039C	YJL092W
YFL039C	YJL098W
YFL039C	YJL128C
YFL039C	YKL078W
YFL039C	YKR026C
YFL039C	YKR036C
YFL039C	YLL011W
YFL039C	YLL050C
YFL039C	YLR006C
YFL039C	YLR074C
YFL039C	YLR096W
YFL039C	YLR186W
YFL039C	YLR196W
YFL039C	YLR247C
YFL039C	YLR319C
YFL039C	YLR337C
YFL039C	YLR383W
YFL039C	YLR427W
YFL039C	YLR429W

YFL039C	YML068W
YFL039C	YML126C
YFL039C	YMR022W
YFL039C	YMR059W
YFL039C	YMR061W
YFL039C	YMR092C
YFL039C	YMR106C
YFL039C	YMR284W
YFL039C	YNL032W
YFL039C	YNL079C
YFL039C	YNL094W
YFL039C	YNL106C
YFL039C	YNL107W
YFL039C	YNL138W
YFL039C	YNL161W
YFL039C	YNL243W
YFL039C	YNL271C
YFL039C	YOL012C
YFL039C	YOL094C
YFL039C	YOL115W
YFL039C	YOL126C
YFL039C	YOL133W
YFL039C	YOR122C
YFL039C	YOR181W
YFL039C	YOR212W
YFL039C	YOR244W
YFL039C	YOR272W
YFL039C	YOR367W
YFL039C	YPL022W
YFL039C	YPL140C
YFL039C	YPL164C
YFL039C	YPL204W
YFL039C	YPL235W
YFL039C	YPL242C
YFL039C	YPR110C
YFL040W	YHL042W
YFL040W	YLR214W
YFL040W	YLR349W
YFL040W	YMR159C
YFL040W	YMR266W
YFL041W	YGR260W
YFL041W	YHL042W
YFL041W	YHR133C
YFL041W	YJL117W
YFL041W	YKL175W

YFL041W YLL028W
YFL041W YLR083C
YFL041W YML123C
YFL041W YOL044W
YFL041W YPL076W
YFL041W YPL264C
YFL041W YPR156C
YFL042C YMR303C
YFL042C YPR086W
YFL042C YPR110C
YFL044C YLR339C
YFL045C YGL208W
YFL045C YHR107C
YFL045C YJL092W
YFL045C YJR035W
YFL045C YLR343W
YFL045C YML064C
YFL045C YMR049C
YFL045C YMR059W
YFL045C YNL189W
YFL045C YOL062C
YFL045C YOR212W
YFL047W YJL095W
YFL047W YJR091C
YFL047W YOR231W
YFL048C YGL212W
YFL048C YKL065C
YFL048C YKL154W
YFL048C YLR080W
YFL048C YLR372W
YFL048C YPR003C
YFL049W YOL152W
YFL049W YOR119C
YFL049W YOR290C
YFL049W YPL016W
YFL050C YLL061W
YFL050C YLR453C
YFL051C YKL026C
YFL051C YNL316C
YFL052W YJL125C
YFL052W YOR366W
YFL054C YML064C
YFL054C YPL076W
YFL055W YOR076C
YFL056C YNL201C

YFL056C	YOL018C
YFL059W	YFL060C
YFL059W	YJL047C
YFL059W	YMR095C
YFL059W	YMR096W
YFL059W	YMR322C
YFL059W	YNL189W
YFL059W	YNL333W
YFL059W	YNL334C
YFL060C	YLR295C
YFL060C	YMR096W
YFL061W	YMR055C
YFL061W	YNL189W
YFL062W	YGL042C
YFL062W	YGR191W
YFL062W	YIL016W
YFL062W	YJL059W
YFL062W	YJL108C
YFL062W	YKL004W
YFL062W	YLR034C
YFL062W	YLR237W
YFL062W	YLR372W
YFL062W	YML048W
YFL062W	YMR221C
YFL062W	YMR306W
YFL062W	YOR071C
YFL062W	YOR079C
YFL062W	YOR092W
YFL063W	YMR032W
YFL065C	YLR415C
YFL066C	YNL147W
YFL067W	YHR056C
YFR001W	YGL237C
YFR001W	YNL061W
YFR001W	YNL230C
YFR001W	YPL204W
YFR001W	YPR016C
YFR002W	YGL172W
YFR002W	YGR119C
YFR002W	YGR120C
YFR002W	YHR135C
YFR002W	YIL149C
YFR002W	YJL039C
YFR002W	YJL041W
YFR002W	YLR178C

YFR002W YML103C
YFR002W YMR129W
YFR002W YMR153W
YFR002W YMR308C
YFR003C YIL064W
YFR003C YML016C
YFR003C YOR147W
YFR003C YOR232W
YFR003C YPL179W
YFR004W YFR010W
YFR004W YFR052W
YFR004W YGL004C
YFR004W YGL048C
YFR004W YGR232W
YFR004W YHL030W
YFR004W YHR027C
YFR004W YHR102W
YFR004W YHR200W
YFR004W YIL075C
YFR004W YIR011C
YFR004W YKL145W
YFR004W YNL290W
YFR004W YOR261C
YFR004W YPR033C
YFR004W YPR108W
YFR006W YNL030W
YFR008W YJL061W
YFR008W YLR291C
YFR008W YLR395C
YFR008W YLR423C
YFR008W YMR052W
YFR008W YOL135C
YFR008W YPR046W
YFR009W YGL195W
YFR009W YLR058C
YFR010W YGL004C
YFR010W YGL048C
YFR010W YGR232W
YFR010W YHL030W
YFR010W YHR200W
YFR010W YKL145W
YFR010W YMR314W
YFR010W YOR259C
YFR010W YOR261C
YFR011C YJL020C

YFR011C	YNL113W
YFR011C	YOL006C
YFR013W	YLR095C
YFR013W	YNR047W
YFR013W	YOL004W
YFR014C	YKL119C
YFR015C	YGL081W
YFR015C	YGL134W
YFR015C	YGR052W
YFR015C	YHR008C
YFR015C	YIL045W
YFR015C	YJL095W
YFR015C	YJL137C
YFR015C	YLR258W
YFR015C	YOR047C
YFR015C	YPL031C
YFR015C	YPL204W
YFR015C	YPL219W
YFR016C	YGL206C
YFR016C	YIL034C
YFR016C	YIL125W
YFR016C	YJR045C
YFR016C	YKL007W
YFR016C	YLL050C
YFR016C	YOR010C
YFR017C	YJL212C
YFR017C	YLR295C
YFR017C	YPR184W
YFR018C	YGR105W
YFR019W	YLR386W
YFR019W	YOL135C
YFR021W	YFR024C-A
YFR021W	YFR040W
YFR021W	YGL190C
YFR021W	YHR103W
YFR021W	YHR199C
YFR021W	YJL052W
YFR021W	YJL153C
YFR021W	YJR140C
YFR021W	YLR094C
YFR021W	YLR191W
YFR021W	YLR222C
YFR021W	YML059C
YFR021W	YNL242W
YFR021W	YNR058W

YFR021W YOL086C	
YFR021W YPL258C	
YFR022W YOR264W	
YFR024C-A	YFR040W
YFR024C-A	YGL060W
YFR024C-A	YGL104C
YFR024C-A	YGL144C
YFR024C-A	YGL181W
YFR024C-A	YGL195W
YFR024C-A	YGL201C
YFR024C-A	YGL238W
YFR024C-A	YGR218W
YFR024C-A	YGR227W
YFR024C-A	YGR240C
YFR024C-A	YGR241C
YFR024C-A	YGR268C
YFR024C-A	YHL004W
YFR024C-A	YHL027W
YFR024C-A	YHR178W
YFR024C-A	YHR182W
YFR024C-A	YIL156W
YFR024C-A	YIR003W
YFR024C-A	YJL004C
YFR024C-A	YJL045W
YFR024C-A	YJL151C
YFR024C-A	YJL194W
YFR024C-A	YJL201W
YFR024C-A	YJR083C
YFR024C-A	YKR030W
YFR024C-A	YLR078C
YFR024C-A	YLR144C
YFR024C-A	YLR206W
YFR024C-A	YLR422W
YFR024C-A	YMR109W
YFR024C-A	YMR162C
YFR024C-A	YMR192W
YFR024C-A	YMR216C
YFR024C-A	YMR232W
YFR024C-A	YMR253C
YFR024C-A	YMR302C
YFR024C-A	YMR304W
YFR024C-A	YNL020C
YFR024C-A	YNL047C
YFR024C-A	YNL065W
YFR024C-A	YNL094W

YFR024C-A	YNL176C
YFR024C-A	YNR031C
YFR024C-A	YOL107W
YFR024C-A	YOR039W
YFR024C-A	YOR042W
YFR024C-A	YOR109W
YFR024C-A	YOR181W
YFR024C-A	YOR247W
YFR024C-A	YOR284W
YFR024C-A	YPL038W
YFR024C-A	YPL246C
YFR024C-A	YPL249C
YFR024C-A	YPR055W
YFR024C-A	YPR081C
YFR024C-A	YPR097W
YFR024C-A	YPR171W
YFR026C	YGL127C
YFR026C	YOR362C
YFR027W	YHR114W
YFR027W	YIL026C
YFR027W	YMR076C
YFR028C	YGL003C
YFR028C	YGL115W
YFR028C	YGL206C
YFR028C	YGR040W
YFR028C	YGR052W
YFR028C	YGR092W
YFR028C	YGR094W
YFR028C	YGR097W
YFR028C	YGR203W
YFR028C	YHR030C
YFR028C	YHR064C
YFR028C	YHR076W
YFR028C	YHR128W
YFR028C	YIL035C
YFR028C	YIL095W
YFR028C	YIR026C
YFR028C	YJL076W
YFR028C	YJL095W
YFR028C	YJL128C
YFR028C	YJL187C
YFR028C	YJR045C
YFR028C	YJR053W
YFR028C	YJR057W
YFR028C	YJR063W

YFR028C	YJR066W
YFR028C	YJR077C
YFR028C	YJR091C
YFR028C	YKL001C
YFR028C	YKL016C
YFR028C	YKL150W
YFR028C	YKL152C
YFR028C	YKL203C
YFR028C	YKR010C
YFR028C	YKR021W
YFR028C	YLR019W
YFR028C	YLR079W
YFR028C	YLR096W
YFR028C	YLR131C
YFR028C	YLR259C
YFR028C	YML064C
YFR028C	YMR049C
YFR028C	YMR055C
YFR028C	YMR270C
YFR028C	YNL001W
YFR028C	YNL014W
YFR028C	YNL071W
YFR028C	YNL161W
YFR028C	YNL307C
YFR028C	YOL055C
YFR028C	YOL078W
YFR028C	YOR032C
YFR028C	YOR061W
YFR028C	YOR093C
YFR028C	YOR119C
YFR028C	YOR265W
YFR028C	YPL061W
YFR028C	YPL258C
YFR028C	YPR042C
YFR028C	YPR069C
YFR028C	YPR183W
YFR029W	YFR040W
YFR030W	YGL004C
YFR030W	YGL137W
YFR030W	YKR026C
YFR030W	YLR291C
YFR030W	YMR117C
YFR030W	YOL126C
YFR031C	YIL144W
YFR031C	YJL074C

YFR031C	YKL068W
YFR031C	YMR065W
YFR031C	YOL055C
YFR031C	YOL115W
YFR031C	YOR195W
YFR031C	YPL124W
YFR033C	YNL236W
YFR034C	YGL048C
YFR034C	YHL002W
YFR034C	YKL145W
YFR034C	YLR293C
YFR034C	YOL001W
YFR034C	YOL108C
YFR034C	YOR259C
YFR034C	YPL122C
YFR034C	YPR086W
YFR036W	YGL240W
YFR036W	YHR166C
YFR036W	YJR091C
YFR036W	YKL022C
YFR036W	YLL051C
YFR036W	YLR127C
YFR036W	YMR001C
YFR036W	YNL172W
YFR036W	YOR249C
YFR037C	YIL126W
YFR037C	YJR031C
YFR037C	YLR176C
YFR037C	YLR321C
YFR037C	YML127W
YFR037C	YMR091C
YFR037C	YOL004W
YFR037C	YOL090W
YFR037C	YOR116C
YFR037C	YOR290C
YFR039C	YIL061C
YFR039C	YNL006W
YFR040W	YGL195W
YFR040W	YGR130C
YFR040W	YGR254W
YFR040W	YHR016C
YFR040W	YHR020W
YFR040W	YHR114W
YFR040W	YJL052W
YFR040W	YJR009C

YFR040W	YKL060C
YFR040W	YLR044C
YFR040W	YLR191W
YFR040W	YLR222C
YFR040W	YLR259C
YFR040W	YMR012W
YFR040W	YOL069W
YFR040W	YPR040W
YFR042W	YJL020C
YFR042W	YLR310C
YFR042W	YNR025C
YFR042W	YPR159W
YFR043C	YLR295C
YFR043C	YPR029C
YFR044C	YLR383W
YFR045W	YHL019C
YFR047C	YLL046C
YFR047C	YLR347C
YFR047C	YNL189W
YFR049W	YIL125W
YFR049W	YMR147W
YFR049W	YOL062C
YFR049W	YOL082W
YFR049W	YOL083W
YFR049W	YOR047C
YFR049W	YOR049C
YFR049W	YPL049C
YFR049W	YPL255W
YFR050C	YGL011C
YFR050C	YGR067C
YFR050C	YHR200W
YFR050C	YJL047C
YFR050C	YJL141C
YFR050C	YML092C
YFR050C	YNL253W
YFR050C	YOR261C
YFR050C	YPR103W
YFR051C	YGL137W
YFR051C	YIL004C
YFR051C	YIL076W
YFR051C	YKR067W
YFR051C	YLR078C
YFR051C	YLR180W
YFR051C	YLR268W
YFR051C	YNL258C

YFR051C	YNL284C
YFR051C	YNL287W
YFR051C	YOR133W
YFR051C	YPL010W
YFR052W	YGL004C
YFR052W	YGL048C
YFR052W	YGR232W
YFR052W	YHL030W
YFR052W	YHR027C
YFR052W	YHR200W
YFR052W	YIL066C
YFR052W	YJR022W
YFR052W	YJR133W
YFR052W	YKL145W
YFR052W	YMR106C
YFR052W	YMR314W
YFR052W	YOR117W
YFR052W	YOR181W
YFR052W	YOR259C
YFR052W	YOR261C
YFR052W	YPR108W
YFR053C	YGL100W
YFR053C	YGL131C
YFR053C	YGL163C
YFR053C	YGL179C
YFR053C	YGL190C
YFR053C	YGL237C
YFR053C	YGR040W
YFR053C	YGR092W
YFR053C	YGR123C
YFR053C	YGR278W
YFR053C	YHR014W
YFR053C	YHR030C
YFR053C	YHR135C
YFR053C	YHR166C
YFR053C	YHR188C
YFR053C	YHR197W
YFR053C	YIL007C
YFR053C	YIL046W
YFR053C	YIL079C
YFR053C	YJL044C
YFR053C	YJL128C
YFR053C	YJL173C
YFR053C	YJR061W
YFR053C	YJR062C

YFR053C	YKL056C
YFR053C	YKL166C
YFR053C	YLL019C
YFR053C	YLR216C
YFR053C	YLR248W
YFR053C	YLR291C
YFR053C	YLR293C
YFR053C	YLR340W
YFR053C	YLR352W
YFR053C	YLR383W
YFR053C	YML064C
YFR053C	YML115C
YFR053C	YMR059W
YFR053C	YMR139W
YFR053C	YMR284W
YFR053C	YMR291W
YFR053C	YNL032W
YFR053C	YNL113W
YFR053C	YNL135C
YFR053C	YNL154C
YFR053C	YNL311C
YFR053C	YNL317W
YFR053C	YOL115W
YFR053C	YOL128C
YFR053C	YOR181W
YFR053C	YOR230W
YFR053C	YOR319W
YFR053C	YOR351C
YFR053C	YPL074W
YFR053C	YPL149W
YFR053C	YPL164C
YFR053C	YPL204W
YFR053C	YPR110C
YFR053C	YPR111W
YFR053C	YPR137W
YGL001C	YGR060W
YGL003C	YHR166C
YGL003C	YIL142W
YGL003C	YJL008C
YGL003C	YJL014W
YGL003C	YJL106W
YGL003C	YJL111W
YGL003C	YJR045C
YGL003C	YKL022C
YGL003C	YKL101W

YGL003C	YKR048C
YGL003C	YLR259C
YGL003C	YMR001C
YGL003C	YMR304W
YGL003C	YOL055C
YGL003C	YPR119W
YGL004C	YGL048C
YGL004C	YGR232W
YGL004C	YHR027C
YGL004C	YHR200W
YGL004C	YIL007C
YGL004C	YIL075C
YGL004C	YIL094C
YGL004C	YJL052W
YGL004C	YJL066C
YGL004C	YJR045C
YGL004C	YJR077C
YGL004C	YKL060C
YGL004C	YKL145W
YGL004C	YLR044C
YGL004C	YLR421C
YGL004C	YNL037C
YGL004C	YOR117W
YGL004C	YOR136W
YGL004C	YOR259C
YGL004C	YOR261C
YGL004C	YOR374W
YGL004C	YPL061W
YGL004C	YPL235W
YGL004C	YPR108W
YGL005C	YGR119C
YGL005C	YLR453C
YGL006W	YLR093C
YGL006W	YNL328C
YGL008C	YGR040W
YGL008C	YHR030C
YGL008C	YIL147C
YGL008C	YKL193C
YGL008C	YKR026C
YGL008C	YLR175W
YGL008C	YLR291C
YGL008C	YOL126C
YGL008C	YPL170W
YGL009C	YMR291W
YGL009C	YOL086C

YGL010W	YLR295C
YGL010W	YPL257W
YGL011C	YGR135W
YGL011C	YHR200W
YGL011C	YJL001W
YGL011C	YLR071C
YGL011C	YML092C
YGL011C	YMR308C
YGL011C	YMR314W
YGL011C	YOL038W
YGL011C	YOR362C
YGL011C	YPL144W
YGL011C	YPR103W
YGL012W	YMR215W
YGL014W	YNL118C
YGL014W	YNL231C
YGL014W	YNR052C
YGL015C	YLR319C
YGL016W	YKL058W
YGL016W	YLR335W
YGL016W	YMR047C
YGL016W	YOR098C
YGL016W	YOR185C
YGL016W	YOR194C
YGL016W	YPR110C
YGL017W	YJL098W
YGL018C	YHR114W
YGL019W	YGL115W
YGL019W	YGL127C
YGL019W	YGL158W
YGL019W	YGL207W
YGL019W	YGR068C
YGL019W	YGR090W
YGL019W	YGR116W
YGL019W	YIL035C
YGL019W	YIL118W
YGL019W	YIL131C
YGL019W	YJL069C
YGL019W	YJL187C
YGL019W	YKL112W
YGL019W	YLR019W
YGL019W	YMR172W
YGL019W	YNL030W
YGL019W	YNL061W
YGL019W	YNL087W

YGL019W	YNL088W
YGL019W	YNL207W
YGL019W	YNL234W
YGL019W	YOL004W
YGL019W	YOL145C
YGL019W	YOR014W
YGL019W	YOR039W
YGL019W	YOR061W
YGL019W	YOR119C
YGL019W	YOR267C
YGL019W	YPL195W
YGL021W	YLL015W
YGL021W	YLR133W
YGL021W	YNR070W
YGL022W	YGL226C-A
YGL022W	YJL002C
YGL022W	YOR085W
YGL022W	YOR103C
YGL023C	YMR049C
YGL024W	YOL130W
YGL025C	YGR104C
YGL025C	YOL051W
YGL025C	YOL130W
YGL026C	YKR026C
YGL026C	YNL135C
YGL026C	YPR110C
YGL027C	YIL022W
YGL027C	YNR002C
YGL028C	YJR022W
YGL029W	YNL243W
YGL030W	YHR114W
YGL030W	YHR204W
YGL032C	YGR257C
YGL035C	YGL173C
YGL035C	YJL020C
YGL035C	YJR045C
YGL035C	YKL074C
YGL035C	YLR259C
YGL035C	YMR109W
YGL035C	YMR240C
YGL035C	YOL041C
YGL035C	YPL025C
YGL036W	YGL061C
YGL037C	YHR114W
YGL037C	YLR347C

YGL037C	YNL189W
YGL037C	YOR098C
YGL038C	YGL055W
YGL040C	YLR347C
YGL040C	YML064C
YGL040C	YNL189W
YGL042C	YGL212W
YGL042C	YHL042W
YGL042C	YJR030C
YGL042C	YLR207W
YGL042C	YMR058W
YGL042C	YOL003C
YGL042C	YPR079W
YGL043W	YJR132W
YGL044C	YGR004W
YGL044C	YHR174W
YGL044C	YJR113C
YGL044C	YLR335W
YGL044C	YMR061W
YGL044C	YMR153W
YGL044C	YMR270C
YGL044C	YNL013C
YGL044C	YNL044W
YGL044C	YNL157W
YGL044C	YOL123W
YGL044C	YOR250C
YGL045W	YJR091C
YGL047W	YLR295C
YGL048C	YGL137W
YGL048C	YGR184C
YGL048C	YGR232W
YGL048C	YGR262C
YGL048C	YHR027C
YGL048C	YHR030C
YGL048C	YHR200W
YGL048C	YIL066C
YGL048C	YJL157C
YGL048C	YJR017C
YGL048C	YJR076C
YGL048C	YKL145W
YGL048C	YKL166C
YGL048C	YLL034C
YGL048C	YLR295C
YGL048C	YLR421C
YGL048C	YML064C

YGL048C	YMR001C
YGL048C	YMR106C
YGL048C	YMR205C
YGL048C	YNL161W
YGL048C	YNL244C
YGL048C	YOL094C
YGL048C	YOR181W
YGL048C	YOR212W
YGL048C	YOR259C
YGL048C	YPL248C
YGL048C	YPR108W
YGL049C	YHR086W
YGL049C	YHR089C
YGL049C	YIR001C
YGL049C	YJL138C
YGL049C	YJR007W
YGL049C	YJR076C
YGL049C	YKL059C
YGL049C	YLR175W
YGL049C	YLR427W
YGL049C	YMR125W
YGL049C	YMR139W
YGL049C	YNL016W
YGL049C	YNL118C
YGL049C	YNL161W
YGL049C	YNL244C
YGL049C	YOL139C
YGL049C	YPL178W
YGL049C	YPR041W
YGL050W	YPL022W
YGL051W	YGL129C
YGL051W	YGL200C
YGL051W	YGR060W
YGL051W	YGR260W
YGL051W	YGR295C
YGL051W	YHL048W
YGL051W	YHR140W
YGL051W	YIL120W
YGL051W	YKL146W
YGL051W	YKL174C
YGL051W	YKR039W
YGL051W	YLL028W
YGL051W	YLR004C
YGL051W	YMR011W
YGL051W	YMR058W

YGL051W	YMR149W
YGL051W	YNL101W
YGL051W	YPL076W
YGL051W	YPL264C
YGL051W	YPR156C
YGL051W	YPR198W
YGL053W	YHR140W
YGL053W	YJL134W
YGL053W	YJL196C
YGL053W	YLR065C
YGL053W	YOL107W
YGL053W	YOR059C
YGL053W	YOR245C
YGL053W	YOR307C
YGL054C	YLL043W
YGL054C	YLR372W
YGL054C	YNL201C
YGL054C	YPL132W
YGL055W	YIL016W
YGL057C	YJL135W
YGL058W	YGL200C
YGL058W	YGR184C
YGL058W	YJL047C
YGL058W	YKL153W
YGL058W	YLR015W
YGL058W	YOL080C
YGL058W	YOR194C
YGL059W	YIL042C
YGL060W	YGL092W
YGL060W	YGR136W
YGL060W	YHL002W
YGL060W	YHR016C
YGL060W	YJL020C
YGL060W	YLR191W
YGL060W	YMR032W
YGL060W	YOL133W
YGL060W	YPR154W
YGL061C	YGR113W
YGL061C	YGR120C
YGL061C	YIR004W
YGL061C	YJL179W
YGL061C	YKL145W
YGL061C	YKR037C
YGL061C	YMR012W
YGL061C	YOR157C

YGL061C	YPL209C
YGL062W	YGR040W
YGL062W	YHR107C
YGL062W	YKL103C
YGL062W	YML058W
YGL062W	YNL135C
YGL062W	YOR089C
YGL062W	YPL149W
YGL062W	YPR110C
YGL064C	YGR220C
YGL066W	YHR099W
YGL066W	YMR223W
YGL066W	YOR119C
YGL066W	YPL047W
YGL068W	YLR074C
YGL068W	YLR340W
YGL069C	YJR091C
YGL070C	YGR005C
YGL070C	YGR186W
YGL070C	YIL021W
YGL070C	YJL140W
YGL070C	YJL170C
YGL070C	YJL182C
YGL070C	YJL190C
YGL070C	YJR162C
YGL070C	YKL036C
YGL070C	YKL086W
YGL070C	YKL141W
YGL070C	YKL150W
YGL070C	YKL190W
YGL070C	YOR116C
YGL070C	YOR151C
YGL070C	YOR224C
YGL070C	YPL129W
YGL070C	YPL203W
YGL070C	YPR093C
YGL070C	YPR187W
YGL071W	YIR018W
YGL071W	YML007W
YGL071W	YMR109W
YGL072C	YPR154W
YGL073W	YHR099W
YGL073W	YHR206W
YGL073W	YOR178C
YGL075C	YJR112W

YGL075C YJR117W
YGL075C YMR117C
YGL075C YPL255W
YGL077C YPL076W
YGL079W YGR113W
YGL081W YGR155W
YGL081W YGR184C
YGL081W YHR082C
YGL081W YJL008C
YGL081W YJL074C
YGL081W YJL130C
YGL081W YJR121W
YGL081W YKL104C
YGL081W YKL182W
YGL081W YLR180W
YGL081W YMR309C
YGL081W YNL064C
YGL081W YOL094C
YGL081W YPL061W
YGL083W YMR264W
YGL084C YHL042W
YGL084C YJL012C
YGL084C YJR091C
YGL084C YLR308W
YGL084C YMR058W
YGL084C YNL058C
YGL085W YGR136W
YGL085W YPR154W
YGL086W YGL116W
YGL086W YJL030W
YGL087C YJL088W
YGL087C YOL081W
YGL087C YOR220W
YGL090W YKL152C
YGL090W YLR109W
YGL090W YLR265C
YGL090W YLR288C
YGL090W YLR424W
YGL090W YOR005C
YGL090W YOR061W
YGL090W YPR120C
YGL091C YIL129C
YGL092W YGL100W
YGL092W YGL172W
YGL092W YGR119C

YGL092W	YGR178C
YGL092W	YGR218W
YGL092W	YHR036W
YGL092W	YJR042W
YGL092W	YKL057C
YGL092W	YKL068W
YGL092W	YLR208W
YGL092W	YLR347C
YGL092W	YMR153W
YGL092W	YMR308C
YGL092W	YPR111W
YGL093W	YJR112W
YGL094C	YIR006C
YGL094C	YKL025C
YGL095C	YKL196C
YGL095C	YMR197C
YGL095C	YOL018C
YGL095C	YOR299W
YGL096W	YJR022W
YGL096W	YLR447C
YGL096W	YOR319W
YGL097W	YGR218W
YGL097W	YIL063C
YGL097W	YLR293C
YGL097W	YLR335W
YGL097W	YOR098C
YGL097W	YOR364W
YGL098W	YLR440C
YGL099W	YGR245C
YGL099W	YHR170W
YGL099W	YIL018W
YGL099W	YIR026C
YGL099W	YLR310C
YGL099W	YMR032W
YGL099W	YPR016C
YGL099W	YPR041W
YGL100W	YGL137W
YGL100W	YGL202W
YGL100W	YGR254W
YGL100W	YHR183W
YGL100W	YIL053W
YGL100W	YJL002C
YGL100W	YJR042W
YGL100W	YKL057C
YGL100W	YLR058C

YGL100W	YLR096W
YGL100W	YLR208W
YGL100W	YLR304C
YGL100W	YLR359W
YGL100W	YML124C
YGL100W	YMR108W
YGL100W	YMR186W
YGL100W	YNL307C
YGL100W	YOR014W
YGL100W	YOR080W
YGL100W	YOR375C
YGL102C	YHR194W
YGL104C	YGL212W
YGL104C	YGL225W
YGL104C	YHR114W
YGL104C	YIL016W
YGL104C	YIL035C
YGL104C	YJL133W
YGL104C	YJR117W
YGL104C	YKL065C
YGL104C	YLR372W
YGL104C	YML048W
YGL104C	YML075C
YGL104C	YMR153W
YGL104C	YMR264W
YGL104C	YNL054W
YGL104C	YOR016C
YGL104C	YPL076W
YGL104C	YPL175W
YGL104C	YPL270W
YGL104C	YPR028W
YGL105W	YGL245W
YGL105W	YGR264C
YGL105W	YKL205W
YGL105W	YML095C
YGL105W	YMR059W
YGL106W	YHR019C
YGL106W	YHR023W
YGL106W	YJL187C
YGL106W	YOR326W
YGL111W	YGR097W
YGL111W	YHR066W
YGL111W	YHR088W
YGL111W	YKR081C
YGL111W	YLR002C

YGL111W	YMR049C
YGL111W	YNL061W
YGL111W	YNL110C
YGL111W	YOR005C
YGL111W	YOR267C
YGL111W	YOR272W
YGL111W	YPL093W
YGL111W	YPL204W
YGL111W	YPR016C
YGL112C	YGR094W
YGL112C	YGR252W
YGL112C	YGR264C
YGL112C	YGR274C
YGL112C	YHR099W
YGL112C	YIL106W
YGL112C	YJL222W-B
YGL112C	YLR055C
YGL112C	YML098W
YGL112C	YMR061W
YGL112C	YMR154C
YGL112C	YMR227C
YGL112C	YMR236W
YGL112C	YMR255W
YGL112C	YNL016W
YGL112C	YOL135C
YGL112C	YOR119C
YGL112C	YOR220W
YGL112C	YPL248C
YGL113W	YPR120C
YGL113W	YPR194C
YGL114W	YPR128C
YGL115W	YGL158W
YGL115W	YGL179C
YGL115W	YGL208W
YGL115W	YIL035C
YGL115W	YJL114W
YGL115W	YJR083C
YGL115W	YKL193C
YGL115W	YML110C
YGL115W	YMR001C
YGL115W	YMR291W
YGL115W	YNL183C
YGL115W	YOL139C
YGL115W	YOR018W
YGL115W	YOR267C

YGL115W	YPR160W
YGL116W	YIL142W
YGL116W	YJL008C
YGL116W	YJL013C
YGL116W	YJL014W
YGL116W	YJL030W
YGL116W	YJL111W
YGL116W	YJR064W
YGL116W	YKL085W
YGL116W	YKL101W
YGL116W	YLR259C
YGL116W	YNL064C
YGL116W	YPL140C
YGL116W	YPL235W
YGL116W	YPR119W
YGL117W	YJR022W
YGL119W	YJR091C
YGL120C	YGL128C
YGL120C	YGL130W
YGL120C	YGR220C
YGL120C	YHR165C
YGL120C	YIL002C
YGL120C	YKL095W
YGL120C	YKL173W
YGL120C	YLL019C
YGL120C	YLR074C
YGL120C	YLR117C
YGL120C	YLR191W
YGL120C	YLR424W
YGL120C	YMR049C
YGL120C	YMR116C
YGL120C	YMR213W
YGL120C	YNL224C
YGL120C	YOR061W
YGL120C	YOR119C
YGL120C	YPL151C
YGL120C	YPR182W
YGL121C	YJR053W
YGL121C	YOR371C
YGL122C	YHR089C
YGL122C	YIL092W
YGL122C	YJL092W
YGL122C	YKR026C
YGL122C	YMR255W
YGL122C	YNL016W

YGL122C	YOL123W
YGL124C	YKL025C
YGL124C	YLR148W
YGL124C	YMR231W
YGL126W	YPL161C
YGL127C	YGL153W
YGL127C	YGL157W
YGL127C	YGL250W
YGL127C	YGR104C
YGL127C	YGR139W
YGL127C	YHR058C
YGL127C	YHR130C
YGL127C	YIL025C
YGL127C	YIL031W
YGL127C	YIR017C
YGL127C	YJL032W
YGL127C	YJR069C
YGL127C	YKL137W
YGL127C	YKR101W
YGL127C	YLL065W
YGL127C	YLR019W
YGL127C	YLR108C
YGL127C	YLR222C
YGL127C	YLR311C
YGL127C	YLR321C
YGL127C	YLR423C
YGL127C	YML133C
YGL127C	YMR054W
YGL127C	YMR072W
YGL127C	YMR202W
YGL127C	YNR010W
YGL127C	YNR028W
YGL127C	YOL016C
YGL127C	YOL018C
YGL127C	YOL135C
YGL127C	YOR047C
YGL127C	YOR119C
YGL127C	YOR128C
YGL127C	YOR162C
YGL127C	YOR174W
YGL127C	YOR263C
YGL127C	YOR289W
YGL127C	YOR377W
YGL127C	YPL064C
YGL127C	YPL155C

YGL127C	YPR168W
YGL128C	YHR165C
YGL128C	YMR213W
YGL129C	YHL004W
YGL129C	YHL048W
YGL129C	YHR114W
YGL130W	YGL207W
YGL130W	YHR121W
YGL130W	YIL131C
YGL130W	YKR001C
YGL130W	YMR012W
YGL130W	YMR309C
YGL130W	YNL030W
YGL130W	YNL088W
YGL130W	YOL078W
YGL130W	YOL145C
YGL130W	YPL228W
YGL131C	YLR413W
YGL131C	YML124C
YGL131C	YNL127W
YGL132W	YOR054C
YGL133W	YGR136W
YGL133W	YLR176C
YGL133W	YOR304W
YGL134W	YJL137C
YGL134W	YKR058W
YGL134W	YKR096W
YGL134W	YLR258W
YGL134W	YML091C
YGL134W	YOR178C
YGL134W	YPL031C
YGL134W	YPL204W
YGL134W	YPR115W
YGL137W	YGR086C
YGL137W	YGR092W
YGL137W	YGR142W
YGL137W	YGR202C
YGL137W	YGR240C
YGL137W	YGR254W
YGL137W	YHR018C
YGL137W	YHR020W
YGL137W	YIL004C
YGL137W	YIL076W
YGL137W	YIL094C
YGL137W	YIL124W

YGL137W YIL136W
YGL137W YIL142W
YGL137W YJL008C
YGL137W YJL026W
YGL137W YJL052W
YGL137W YJL066C
YGL137W YJL111W
YGL137W YJL130C
YGL137W YJR009C
YGL137W YJR045C
YGL137W YJR070C
YGL137W YJR077C
YGL137W YKL029C
YGL137W YKL078W
YGL137W YKL081W
YGL137W YKL104C
YGL137W YKL152C
YGL137W YKL166C
YGL137W YKL182W
YGL137W YKL204W
YGL137W YKR007W
YGL137W YKR046C
YGL137W YKR067W
YGL137W YLR044C
YGL137W YLR078C
YGL137W YLR100W
YGL137W YLR180W
YGL137W YLR216C
YGL137W YLR259C
YGL137W YLR268W
YGL137W YLR355C
YGL137W YML085C
YGL137W YML124C
YGL137W YMR108W
YGL137W YMR146C
YGL137W YMR214W
YGL137W YNL037C
YGL137W YNL064C
YGL137W YNL181W
YGL137W YNL250W
YGL137W YNL284C
YGL137W YNL287W
YGL137W YNR001C
YGL137W YOL133W
YGL137W YOR027W

YGL137W	YOR136W
YGL137W	YOR151C
YGL137W	YOR362C
YGL137W	YOR374W
YGL137W	YPL010W
YGL137W	YPL061W
YGL137W	YPL235W
YGL137W	YPL240C
YGL137W	YPR108W
YGL137W	YPR110C
YGL137W	YPR124W
YGL138C	YGR123C
YGL139W	YOR010C
YGL141W	YKL021C
YGL142C	YML012W
YGL144C	YHR016C
YGL144C	YPR154W
YGL145W	YLR373C
YGL145W	YNL258C
YGL145W	YOR075W
YGL145W	YPR086W
YGL145W	YPR105C
YGL146C	YOR005C
YGL146C	YPR137W
YGL147C	YLR078C
YGL148W	YOL107W
YGL149W	YGR218W
YGL149W	YML092C
YGL150C	YNL068C
YGL150C	YOR355W
YGL150C	YPL235W
YGL151W	YGR104C
YGL151W	YNL025C
YGL151W	YOL135C
YGL151W	YOR119C
YGL153W	YIR034C
YGL153W	YLR191W
YGL153W	YLR284C
YGL153W	YNL214W
YGL153W	YOL044W
YGL153W	YOL105C
YGL153W	YOR180C
YGL153W	YOR264W
YGL153W	YPR105C
YGL154C	YGL254W

YGL154C	YOR028C
YGL154C	YOR128C
YGL155W	YKL019W
YGL156W	YGL215W
YGL156W	YKL103C
YGL156W	YOL001W
YGL156W	YOL082W
YGL156W	YOL083W
YGL156W	YPL204W
YGL156W	YPR110C
YGL157W	YJR091C
YGL157W	YNR064C
YGL158W	YGR071C
YGL158W	YGR130C
YGL158W	YGR254W
YGL158W	YGR270W
YGL158W	YHR107C
YGL158W	YJL052W
YGL158W	YJR009C
YGL158W	YJR045C
YGL158W	YJR076C
YGL158W	YJR092W
YGL158W	YKL152C
YGL158W	YLR113W
YGL158W	YLR314C
YGL158W	YLR373C
YGL158W	YMR205C
YGL158W	YNL166C
YGL158W	YOL082W
YGL158W	YOR018W
YGL158W	YOR136W
YGL158W	YOR261C
YGL158W	YPL128C
YGL161C	YGL198W
YGL161C	YGL210W
YGL161C	YGL212W
YGL161C	YGR172C
YGL161C	YHR105W
YGL161C	YKR014C
YGL161C	YML001W
YGL161C	YNL093W
YGL161C	YNL304W
YGL161C	YOR036W
YGL161C	YOR089C
YGL161C	YPL095C

YGL163C	YHR016C
YGL163C	YJL034W
YGL163C	YJR045C
YGL163C	YKL035W
YGL163C	YKL056C
YGL163C	YKL085W
YGL163C	YLR259C
YGL163C	YML085C
YGL163C	YOL055C
YGL163C	YOR232W
YGL163C	YOR374W
YGL163C	YPL061W
YGL163C	YPL240C
YGL163C	YPL258C
YGL165C	YOR362C
YGL166W	YJL154C
YGL166W	YKR011C
YGL166W	YLL028W
YGL166W	YML006C
YGL166W	YML092C
YGL166W	YNL189W
YGL166W	YOR210W
YGL166W	YOR220W
YGL168W	YLR288C
YGL169W	YLL004W
YGL170C	YGR119C
YGL170C	YGR218W
YGL170C	YMR047C
YGL170C	YMR153W
YGL171W	YGR090W
YGL171W	YJR091C
YGL171W	YMR093W
YGL172W	YGR119C
YGL172W	YGR218W
YGL172W	YHR216W
YGL172W	YJL041W
YGL172W	YJR042W
YGL172W	YKL057C
YGL172W	YKR082W
YGL172W	YLL024C
YGL172W	YLR178C
YGL172W	YLR335W
YGL172W	YLR347C
YGL172W	YML092C
YGL172W	YMR294W

YGL172W	YMR308C
YGL172W	YNL041C
YGL172W	YNL086W
YGL172W	YNL189W
YGL172W	YPL125W
YGL173C	YGL174W
YGL173C	YGR054W
YGL173C	YGR103W
YGL173C	YHR052W
YGL173C	YIL035C
YGL173C	YIL131C
YGL173C	YJL095W
YGL173C	YJR022W
YGL173C	YJR076C
YGL173C	YKL021C
YGL173C	YKL078W
YGL173C	YKL139W
YGL173C	YKL189W
YGL173C	YKL198C
YGL173C	YLL019C
YGL173C	YLR233C
YGL173C	YLR263W
YGL173C	YLR427W
YGL173C	YLR438C-A
YGL173C	YML064C
YGL173C	YML111W
YGL173C	YMR093W
YGL173C	YMR137C
YGL173C	YNL118C
YGL173C	YNL175C
YGL173C	YNL230C
YGL173C	YNL311C
YGL173C	YOL100W
YGL173C	YOL115W
YGL173C	YOR080W
YGL173C	YPL109C
YGL173C	YPR106W
YGL173C	YPR110C
YGL174W	YGL216W
YGL174W	YIR005W
YGL174W	YLR016C
YGL174W	YMR012W
YGL175C	YLR347C
YGL175C	YNL189W
YGL177W	YJL054W

YGL178W	YGR040W
YGL178W	YHR114W
YGL178W	YLR452C
YGL178W	YNR052C
YGL178W	YOL149W
YGL179C	YKR096W
YGL179C	YLL050C
YGL179C	YOL055C
YGL180W	YLR423C
YGL180W	YML112W
YGL180W	YPR049C
YGL180W	YPR185W
YGL181W	YGR241C
YGL181W	YHR016C
YGL181W	YHR086W
YGL181W	YHR177W
YGL181W	YIL010W
YGL181W	YIL052C
YGL181W	YIL057C
YGL181W	YIL122W
YGL181W	YIR006C
YGL181W	YJL141C
YGL181W	YKR028W
YGL181W	YLL019C
YGL181W	YLR191W
YGL181W	YLR291C
YGL181W	YNR068C
YGL181W	YOR140W
YGL181W	YOR284W
YGL181W	YOR331C
YGL181W	YOR333C
YGL181W	YOR384W
YGL181W	YPL013C
YGL181W	YPL053C
YGL181W	YPL156C
YGL181W	YPL188W
YGL181W	YPR171W
YGL183C	YPL051W
YGL184C	YML127W
YGL185C	YOL113W
YGL187C	YGL213C
YGL187C	YML042W
YGL189C	YHR114W
YGL189C	YJL020C
YGL189C	YLL027W

YGL189C	YLR288C
YGL189C	YLR435W
YGL190C	YGR161C
YGL190C	YGR240C
YGL190C	YGR254W
YGL190C	YHR016C
YGL190C	YHR033W
YGL190C	YHR082C
YGL190C	YHR114W
YGL190C	YIL142W
YGL190C	YJL008C
YGL190C	YJL014W
YGL190C	YJL020C
YGL190C	YJL095W
YGL190C	YJL111W
YGL190C	YJL153C
YGL190C	YJR045C
YGL190C	YJR064W
YGL190C	YJR066W
YGL190C	YJR138W
YGL190C	YKL042W
YGL190C	YKL081W
YGL190C	YKL129C
YGL190C	YLR044C
YGL190C	YLR180W
YGL190C	YLR191W
YGL190C	YLR249W
YGL190C	YLR259C
YGL190C	YLR373C
YGL190C	YML109W
YGL190C	YMR109W
YGL190C	YMR273C
YGL190C	YNL030W
YGL190C	YNL064C
YGL190C	YNL154C
YGL190C	YNL222W
YGL190C	YNR032W
YGL190C	YOR133W
YGL190C	YOR349W
YGL190C	YOR374W
YGL190C	YPR154W
YGL192W	YJL020C
YGL192W	YOR125C
YGL194C	YIL112W
YGL194C	YKR029C

YGL194C	YMR273C
YGL194C	YOL068C
YGL195W	YHR108W
YGL195W	YIL106W
YGL195W	YJL050W
YGL195W	YJL074C
YGL195W	YLL050C
YGL195W	YNL132W
YGL195W	YNL323W
YGL195W	YOL133W
YGL195W	YOR326W
YGL195W	YPL082C
YGL195W	YPL140C
YGL195W	YPR161C
YGL197W	YHL002W
YGL197W	YKL129C
YGL197W	YLR191W
YGL197W	YLR310C
YGL197W	YMR109W
YGL197W	YMR308C
YGL197W	YNL025C
YGL197W	YNL307C
YGL197W	YPL203W
YGL197W	YPL204W
YGL198W	YGL210W
YGL198W	YGR172C
YGL198W	YJR091C
YGL198W	YKR014C
YGL198W	YLR262C
YGL198W	YML001W
YGL198W	YMR215W
YGL198W	YNL093W
YGL198W	YNL263C
YGL198W	YNL304W
YGL198W	YPL095C
YGL200C	YGR060W
YGL200C	YGR138C
YGL200C	YGR284C
YGL200C	YHR110W
YGL200C	YHR123W
YGL200C	YHR140W
YGL200C	YIL016W
YGL200C	YIL109C
YGL200C	YJL108C
YGL200C	YJL117W

YGL200C	YJR117W
YGL200C	YKL065C
YGL200C	YKL154W
YGL200C	YLR208W
YGL200C	YLR372W
YGL200C	YLR378C
YGL200C	YML012W
YGL200C	YML048W
YGL200C	YMR215W
YGL200C	YNL130C
YGL200C	YOR016C
YGL200C	YPL218W
YGL200C	YPL227C
YGL200C	YPR181C
YGL200C	YPR194C
YGL200C	YPR201W
YGL201C	YHR114W
YGL201C	YIL150C
YGL201C	YLR233C
YGL201C	YLR274W
YGL201C	YMR032W
YGL201C	YMR109W
YGL201C	YMR216C
YGL201C	YPL093W
YGL201C	YPR019W
YGL201C	YPR154W
YGL202W	YGR074W
YGL202W	YLR383W
YGL202W	YPL149W
YGL204C	YJR091C
YGL204C	YMR209C
YGL205W	YJL095W
YGL206C	YGR092W
YGL206C	YGR167W
YGL206C	YHR030C
YGL206C	YHR082C
YGL206C	YHR108W
YGL206C	YHR161C
YGL206C	YHR186C
YGL206C	YHR196W
YGL206C	YIL066C
YGL206C	YIL094C
YGL206C	YIL095W
YGL206C	YKL103C
YGL206C	YKL135C

YGL206C	YKR026C
YGL206C	YLR233C
YGL206C	YML064C
YGL206C	YMR026C
YGL206C	YMR106C
YGL206C	YMR117C
YGL206C	YNL056W
YGL206C	YNL244C
YGL206C	YNL311C
YGL206C	YNR031C
YGL206C	YOL126C
YGL206C	YOR080W
YGL206C	YOR109W
YGL206C	YOR181W
YGL206C	YOR319W
YGL206C	YOR351C
YGL206C	YOR386W
YGL206C	YPL106C
YGL206C	YPL140C
YGL206C	YPR110C
YGL206C	YPR178W
YGL207W	YGR163W
YGL207W	YIL035C
YGL207W	YJL124C
YGL207W	YLR418C
YGL207W	YML069W
YGL207W	YNL102W
YGL207W	YOL006C
YGL207W	YOL054W
YGL207W	YOL128C
YGL207W	YOL145C
YGL207W	YOR039W
YGL207W	YOR061W
YGL208W	YGR231C
YGL208W	YJL008C
YGL208W	YJL034W
YGL208W	YJR045C
YGL208W	YJR077C
YGL208W	YJR121W
YGL208W	YKL152C
YGL208W	YLR259C
YGL208W	YML085C
YGL208W	YML124C
YGL208W	YMR214W
YGL208W	YNL064C

YGL208W	YNR035C
YGL208W	YOR136W
YGL210W	YNL263C
YGL210W	YOR070C
YGL212W	YJL151C
YGL212W	YJR040W
YGL212W	YKL196C
YGL212W	YLR093C
YGL212W	YLR166C
YGL212W	YLR423C
YGL212W	YML001W
YGL212W	YMR053C
YGL212W	YMR071C
YGL212W	YMR197C
YGL212W	YNL263C
YGL212W	YOL129W
YGL212W	YOR106W
YGL212W	YOR292C
YGL212W	YPL246C
YGL213C	YHL022C
YGL213C	YHR199C
YGL213C	YLR398C
YGL213C	YOR076C
YGL213C	YPR189W
YGL214W	YLR435W
YGL215W	YKL103C
YGL215W	YMR047C
YGL215W	YPL031C
YGL216W	YHR183W
YGL216W	YOL001W
YGL217C	YJR091C
YGL220W	YLL029W
YGL220W	YLR052W
YGL220W	YNL071W
YGL220W	YOL055C
YGL220W	YOR264W
YGL221C	YML064C
YGL221C	YNL189W
YGL222C	YHR114W
YGL222C	YJR091C
YGL223C	YGR120C
YGL223C	YLR026C
YGL223C	YNL041C
YGL225W	YHR114W
YGL225W	YIR038C

YGL225W	YJR091C
YGL226C-A	YJL002C
YGL226C-A	YMR149W
YGL226C-A	YOR085W
YGL226C-A	YOR103C
YGL226W	YLR295C
YGL227W	YJL106W
YGL229C	YHR186C
YGL229C	YJL013C
YGL229C	YJL030W
YGL229C	YJL178C
YGL229C	YJL211C
YGL229C	YJR066W
YGL229C	YJR091C
YGL229C	YMR047C
YGL229C	YMR181C
YGL229C	YNL006W
YGL229C	YNL076W
YGL229C	YOR062C
YGL229C	YPL180W
YGL229C	YPR040W
YGL230C	YKL110C
YGL230C	YOR161C
YGL233W	YGR152C
YGL233W	YLR166C
YGL234W	YJL098W
YGL234W	YJR068W
YGL234W	YKL166C
YGL234W	YLR175W
YGL234W	YLR291C
YGL234W	YOL094C
YGL234W	YOL126C
YGL234W	YOR090C
YGL234W	YOR298W
YGL234W	YPL259C
YGL234W	YPR154W
YGL236C	YHR114W
YGL236C	YIL057C
YGL236C	YMR023C
YGL236C	YMR047C
YGL236C	YPL179W
YGL237C	YGR146C
YGL237C	YGR155W
YGL237C	YGR267C
YGL237C	YHR052W

YGL237C	YIL053W
YGL237C	YIL070C
YGL237C	YJL081C
YGL237C	YJL138C
YGL237C	YJR045C
YGL237C	YJR121W
YGL237C	YJR132W
YGL237C	YKL152C
YGL237C	YKL214C
YGL237C	YKR028W
YGL237C	YKR048C
YGL237C	YLR191W
YGL237C	YLR423C
YGL237C	YMR153W
YGL237C	YMR308C
YGL237C	YNL063W
YGL237C	YNL064C
YGL237C	YNR031C
YGL237C	YOL055C
YGL237C	YOL139C
YGL237C	YOR047C
YGL237C	YOR272W
YGL237C	YOR358W
YGL237C	YPL166W
YGL237C	YPR016C
YGL237C	YPR069C
YGL237C	YPR085C
YGL238W	YHR114W
YGL238W	YLR293C
YGL238W	YNL189W
YGL238W	YNL236W
YGL240W	YHR166C
YGL240W	YKL022C
YGL240W	YLR127C
YGL240W	YNL172W
YGL240W	YOR249C
YGL241W	YKR048C
YGL241W	YLL050C
YGL241W	YOL012C
YGL242C	YKR099W
YGL244W	YHR009C
YGL244W	YIL035C
YGL244W	YLR418C
YGL244W	YML010W
YGL244W	YMR066W

YGL244W	YOL145C
YGL244W	YOR123C
YGL244W	YOR319W
YGL245W	YGR040W
YGL245W	YGR092W
YGL245W	YHL020C
YGL245W	YHR107C
YGL245W	YHR135C
YGL245W	YKL081W
YGL245W	YKL098W
YGL245W	YKR055W
YGL245W	YLR291C
YGL245W	YML064C
YGL245W	YMR049C
YGL245W	YMR059W
YGL245W	YNL135C
YGL245W	YNL244C
YGL245W	YOL094C
YGL245W	YOR014W
YGL245W	YOR212W
YGL245W	YPR110C
YGL246C	YOR048C
YGL247W	YOR103C
YGL249W	YLR394W
YGL250W	YJL020C
YGL250W	YLR295C
YGL250W	YMR068W
YGL250W	YNL039W
YGL251C	YNL127W
YGL252C	YNL076W
YGL252C	YNL132W
YGL253W	YLR291C
YGL253W	YLR383W
YGL253W	YNL135C
YGL253W	YNL244C
YGL253W	YPL149W
YGL254W	YGR047C
YGL254W	YHR215W
YGL254W	YNL189W
YGL254W	YOR039W
YGL256W	YJR035W
YGL256W	YKL108W
YGL256W	YLR146C
YGL256W	YNL244C
YGL258W	YKL002W

YGL259W	YHL042W
YGL259W	YJL070C
YGL259W	YKL002W
YGL263W	YJR020W
YGL263W	YJR095W
YGR002C	YHR090C
YGR002C	YHR099W
YGR002C	YJL081C
YGR002C	YLR385C
YGR002C	YLR399C
YGR002C	YML041C
YGR002C	YNL107W
YGR002C	YOL012C
YGR002C	YOR244W
YGR002C	YPL235W
YGR002C	YPR023C
YGR002C	YPR154W
YGR003W	YLR297W
YGR003W	YOL133W
YGR004W	YHR114W
YGR004W	YLR324W
YGR004W	YPR028W
YGR005C	YHR191C
YGR005C	YIL021W
YGR005C	YNL092W
YGR005C	YPL129W
YGR005C	YPL203W
YGR005C	YPR086W
YGR009C	YLR268W
YGR009C	YLR295C
YGR009C	YMR047C
YGR009C	YMR183C
YGR009C	YOR327C
YGR009C	YPL232W
YGR009C	YPR032W
YGR010W	YLR328W
YGR010W	YLR438W
YGR010W	YML031W
YGR010W	YNL189W
YGR010W	YNL218W
YGR010W	YPL070W
YGR013W	YGR074W
YGR013W	YHR086W
YGR013W	YIL009W
YGR013W	YIL061C

YGR013WYKL012W
YGR013WYLR147C
YGR013WYLR275W
YGR013WYLR298C
YGR013WYML046W
YGR013WYMR125W
YGR013WYPL178W
YGR013WYPR182W
YGR014WYIL144W
YGR014WYJL013C
YGR014WYJL030W
YGR014WYNL233W
YGR014WYNL298W
YGR014WYPL211W
YGR016WYLR135W
YGR016WYMR104C
YGR017WYIL131C
YGR017WYLR072W
YGR017WYLR403W
YGR018C YJR091C
YGR018C YLR452C
YGR020C YHR039C-A
YGR020C YHR060W
YGR020C YJL141C
YGR020C YKL002W
YGR020C YLR447C
YGR020C YMR054W
YGR020C YOR270C
YGR020C YOR332W
YGR020C YPR036W
YGR021WYHR165C
YGR023WYKL119C
YGR023WYLR460C
YGR023WYNL237W
YGR024C YIL082W
YGR024C YKL090W
YGR024C YLR291C
YGR024C YNL189W
YGR024C YPL110C
YGR026WYLR324W
YGR028WYLR067C
YGR029WYKL084W
YGR029WYKL195W
YGR031WYHR114W
YGR032WYNL298W

YGR032WYPR165W
YGR033C YLR135W
YGR033C YOL100W
YGR034WYHR197W
YGR035C YKL002W
YGR035C YLR295C
YGR035C YOR031W
YGR035C YOR118W
YGR037C YGR136W
YGR037C YHL002W
YGR037C YHR016C
YGR037C YLR191W
YGR037C YLR310C
YGR037C YMR109W
YGR038WYKL212W
YGR038WYLR350W
YGR038WYMR296C
YGR040WYGR097W
YGR040WYGR155W
YGR040WYHR033W
YGR040WYHR084W
YGR040WYHR200W
YGR040WYIL075C
YGR040WYIL142W
YGR040WYIL148W
YGR040WYJL111W
YGR040WYJL130C
YGR040WYJR045C
YGR040WYJR072C
YGR040WYJR077C
YGR040WYJR121W
YGR040WYKL035W
YGR040WYKL104C
YGR040WYKL161C
YGR040WYKR048C
YGR040WYLR154C
YGR040WYLR180W
YGR040WYLR259C
YGR040WYLR362W
YGR040WYLR430W
YGR040WYLR452C
YGR040WYML085C
YGR040WYML099C
YGR040WYML123C
YGR040WYML124C

YGR040WYMR205C
YGR040WYMR290C
YGR040WYMR319C
YGR040WYNL064C
YGR040WYNL071W
YGR040WYNL085W
YGR040WYOL033W
YGR040WYOL055C
YGR040WYOL078W
YGR040WYOR374W
YGR040WYPL049C
YGR040WYPL115C
YGR040WYPL235W
YGR040WYPL240C
YGR040WYPR010C
YGR040WYPR034W
YGR040WYPR115W
YGR041WYPL251W
YGR042WYJR091C
YGR045C YOR271C
YGR046WYNL236W
YGR047C YGR246C
YGR047C YLR096W
YGR047C YLR257W
YGR047C YNL039W
YGR047C YOR110W
YGR048WYHR039C-A
YGR048WYJL020C
YGR048WYKR002W
YGR048WYPL222W
YGR048WYPR154W
YGR049WYGR278W
YGR049WYJR091C
YGR052WYGR097W
YGR052WYHR082C
YGR052WYHR186C
YGR052WYIL045W
YGR052WYJL076W
YGR052WYJL086C
YGR052WYJR045C
YGR052WYJR066W
YGR052WYJR138W
YGR052WYKL010C
YGR052WYLL013C
YGR052WYLR072W

YGR052WYLR127C
YGR052WYLR258W
YGR052WYLR259C
YGR052WYNL006W
YGR052WYNL076W
YGR052WYOR341W
YGR052WYPL060W
YGR052WYPL180W
YGR052WYPR010C-A
YGR052WYPR030W
YGR053C YPR086W
YGR054WYHL034C
YGR054WYNL132W
YGR054WYNL207W
YGR055WYOR322C
YGR056WYMR047C
YGR056WYOR319W
YGR057C YIL109C
YGR057C YKL015W
YGR058WYGR136W
YGR058WYLR113W
YGR058WYML064C
YGR058WYNL047C
YGR058WYOR097C
YGR058WYOR264W
YGR059WYHR023W
YGR059WYMR065W
YGR060WYGR105W
YGR060WYGR175C
YGR060WYGR284C
YGR060WYHL003C
YGR060WYHR123W
YGR060WYHR140W
YGR060WYIL016W
YGR060WYJL117W
YGR060WYJL129C
YGR060WYJL202C
YGR060WYJR010C-A
YGR060WYJR117W
YGR060WYKL008C
YGR060WYKL065C
YGR060WYKL154W
YGR060WYKR088C
YGR060WYLL005C
YGR060WYLL028W

YGR060WYLR100W
YGR060WYLR343W
YGR060WYLR372W
YGR060WYML048W
YGR060WYML075C
YGR060WYMR215W
YGR060WYMR264W
YGR060WYNL101W
YGR060WYOL003C
YGR060WYOR016C
YGR060WYPL076W
YGR060WYPL227C
YGR060WYPL264C
YGR060WYPR028W
YGR061C YLR386W
YGR063C YML010W
YGR063C YML095C
YGR064WYJR091C
YGR065C YJL199C
YGR066C YKL130C
YGR066C YLR386W
YGR066C YML064C
YGR067C YJL080C
YGR067C YMR012W
YGR067C YNL085W
YGR068C YGR080W
YGR068C YOR119C
YGR070WYHR030C
YGR071C YHR109W
YGR071C YJL058C
YGR071C YPL128C
YGR072WYHR077C
YGR072WYIL008W
YGR072WYKL149C
YGR072WYMR080C
YGR073C YLR453C
YGR073C YML119W
YGR074WYGR091W
YGR074WYJL203W
YGR074WYJR033C
YGR074WYKL173W
YGR074WYKL183W
YGR074WYKL188C
YGR074WYKL205W
YGR074WYLR147C

YGR074WYLR298C
YGR074WYLR440C
YGR074WYML046W
YGR074WYMR125W
YGR074WYPL213W
YGR075C YLR147C
YGR076C YGR220C
YGR078C YLR200W
YGR078C YLR212C
YGR078C YML094W
YGR078C YNL153C
YGR079WYLR002C
YGR079WYNL081C
YGR079WYNR012W
YGR080WYMR047C
YGR080WYNL110C
YGR080WYNL138W-A
YGR081C YLR295C
YGR081C YMR308C
YGR082WYJR091C
YGR082WYMR203W
YGR082WYMR299C
YGR082WYNL003C
YGR082WYNL018C
YGR082WYNL131W
YGR083C YGR254W
YGR083C YIL053W
YGR083C YIL131C
YGR083C YJL052W
YGR083C YJR007W
YGR083C YJR009C
YGR083C YKL081W
YGR083C YKR026C
YGR083C YLR044C
YGR083C YLR291C
YGR083C YLR355C
YGR083C YMR108W
YGR083C YNL265C
YGR083C YOR260W
YGR084C YGR251W
YGR084C YHL004W
YGR085C YLR288C
YGR086C YHR030C
YGR086C YKL142W
YGR086C YLR447C

YGR086C YMR059W
YGR086C YNL230C
YGR086C YOL100W
YGR086C YPL022W
YGR086C YPL074W
YGR086C YPL150W
YGR086C YPL204W
YGR087C YHR030C
YGR087C YJR090C
YGR087C YLR427W
YGR087C YPL022W
YGR087C YPR030W
YGR088WYNL189W
YGR089WYHL010C
YGR089WYLR086W
YGR089WYNL153C
YGR089WYOR269W
YGR089WYPR141C
YGR090WYGR095C
YGR090WYGR128C
YGR090WYGR145W
YGR090WYHR013C
YGR090WYHR148W
YGR090WYIL035C
YGR090WYJL069C
YGR090WYJL095W
YGR090WYJL109C
YGR090WYJL168C
YGR090WYJR002W
YGR090WYJR017C
YGR090WYKL139W
YGR090WYKR060W
YGR090WYLL019C
YGR090WYLR003C
YGR090WYLR129W
YGR090WYLR186W
YGR090WYLR409C
YGR090WYLR427W
YGR090WYML111W
YGR090WYMR116C
YGR090WYMR128W
YGR090WYNL061W
YGR090WYNL132W
YGR090WYNL207W
YGR090WYNL230C

YGR090WYOL021C
YGR090WYOL077C
YGR090WYOR014W
YGR090WYOR039W
YGR090WYOR061W
YGR090WYOR145C
YGR090WYOR206W
YGR090WYPL126W
YGR090WYPL153C
YGR090WYPL212C
YGR090WYPL266W
YGR090WYPR144C
YGR091WYHR165C
YGR091WYJL063C
YGR091WYJR022W
YGR091WYKL170W
YGR091WYKL173W
YGR091WYLR147C
YGR091WYLR275W
YGR091WYLR438C-A
YGR091WYLR439W
YGR091WYML025C
YGR091WYMR024W
YGR091WYNL005C
YGR091WYOR308C
YGR091WYPR082C
YGR091WYPR178W
YGR091WYPR182W
YGR092WYHR030C
YGR092WYIL075C
YGR092WYIL106W
YGR092WYJL005W
YGR092WYJL076W
YGR092WYJL088W
YGR092WYJL098W
YGR092WYJL130C
YGR092WYJR045C
YGR092WYJR072C
YGR092WYJR121W
YGR092WYKL061W
YGR092WYKL126W
YGR092WYKL152C
YGR092WYKL182W
YGR092WYKR036C
YGR092WYLR180W

YGR092WYLR424W
YGR092WYML085C
YGR092WYMR186W
YGR092WYMR205C
YGR092WYNL071W
YGR092WYNL085W
YGR092WYNL241C
YGR092WYNL288W
YGR092WYNR052C
YGR092WYOL055C
YGR092WYOR117W
YGR092WYOR151C
YGR092WYOR317W
YGR092WYOR374W
YGR092WYPL042C
YGR092WYPL235W
YGR092WYPL240C
YGR092WYPR160W
YGR094WYLR175W
YGR094WYLR383W
YGR094WYMR234W
YGR094WYNL135C
YGR094WYNL244C
YGR094WYOR089C
YGR094WYPR110C
YGR094WYPR154W
YGR095C YGR158C
YGR095C YGR195W
YGR095C YGR262C
YGR095C YHR069C
YGR095C YHR081W
YGR095C YNL189W
YGR095C YNL232W
YGR095C YNL265C
YGR095C YOL021C
YGR095C YOL142W
YGR095C YOR001W
YGR095C YOR076C
YGR095C YOR326W
YGR097WYJL106W
YGR097WYKL116C
YGR097WYLR096W
YGR097WYLR113W
YGR097WYMR049C
YGR097WYMR159C

YGR097WYNL207W
YGR097WYNL307C
YGR097WYOR014W
YGR097WYOR061W
YGR097WYPL093W
YGR097WYPR115W
YGR098C YGR163W
YGR099WYPR086W
YGR100WYLR262C
YGR100WYLR453C
YGR101WYHL021C
YGR101WYJL213W
YGR101WYKR066C
YGR102C YLR191W
YGR103WYGR245C
YGR103WYHL034C
YGR103WYHR066W
YGR103WYHR197W
YGR103WYIL018W
YGR103WYIL035C
YGR103WYIL070C
YGR103WYIR026C
YGR103WYJL095W
YGR103WYKL021C
YGR103WYKR081C
YGR103WYLR074C
YGR103WYMR049C
YGR103WYMR229C
YGR103WYMR290C
YGR103WYNL061W
YGR103WYNL071W
YGR103WYNL110C
YGR103WYNL175C
YGR103WYNL189W
YGR103WYNL230C
YGR103WYOR005C
YGR103WYOR039W
YGR103WYOR061W
YGR103WYOR272W
YGR103WYPL043W
YGR103WYPL141C
YGR103WYPR016C
YGR103WYPR017C
YGR103WYPR041W
YGR103WYPR115W

YGR103WYPR143W
YGR104C YHR041C
YGR104C YHR058C
YGR104C YJL005W
YGR104C YLR071C
YGR104C YLR340W
YGR104C YMR112C
YGR104C YNL025C
YGR104C YNL236W
YGR104C YNR010W
YGR104C YOL051W
YGR104C YOL135C
YGR104C YOR174W
YGR104C YPL042C
YGR104C YPL248C
YGR104C YPR070W
YGR104C YPR168W
YGR108WYLR079W
YGR108WYOR171C
YGR108WYPL124W
YGR109C YJL157C
YGR109C YLR079W
YGR109C YOL054W
YGR109C YOR368W
YGR109C YPL256C
YGR110WYHR114W
YGR111WYHR135C
YGR111WYOL151W
YGR113WYIL144W
YGR113WYJL064W
YGR113WYKR037C
YGR113WYLR329W
YGR113WYLR423C
YGR113WYLR424W
YGR113WYMR308C
YGR113WYPL209C
YGR113WYPR045C
YGR113WYPR046W
YGR113WYPR049C
YGR114C YHR114W
YGR115C YJR086W
YGR116WYIL035C
YGR116WYNL118C
YGR116WYOR061W
YGR116WYPL203W

YGR116WYPR133C
YGR117C YNL236W
YGR119C YGR120C
YGR119C YGR218W
YGR119C YJL041W
YGR119C YJR042W
YGR119C YKL057C
YGR119C YKL061W
YGR119C YKL103C
YGR119C YKR082W
YGR119C YLL024C
YGR119C YLL026W
YGR119C YLR178C
YGR119C YLR335W
YGR119C YLR347C
YGR119C YLR423C
YGR119C YMR139W
YGR119C YMR236W
YGR119C YMR294W
YGR119C YMR308C
YGR119C YNL189W
YGR119C YOL069W
YGR119C YOR035C
YGR119C YOR160W
YGR119C YPL125W
YGR119C YPR046W
YGR119C YPR083W
YGR120C YLR026C
YGR120C YLR423C
YGR120C YMR025W
YGR120C YNL041C
YGR120C YNL086W
YGR120C YNL287W
YGR120C YNR025C
YGR120C YOR158W
YGR120C YOR353C
YGR120C YPR105C
YGR120C YPR185W
YGR121C YHR140W
YGR121C YIR038C
YGR121C YOR321W
YGR122WYLR025W
YGR122WYLR295C
YGR123C YMR008C
YGR123C YMR021C

YGR123C YMR186W
YGR123C YNL011C
YGR123C YNR032W
YGR123C YOR176W
YGR123C YPL240C
YGR124WYKL137W
YGR126WYLR063W
YGR129WYJR050W
YGR129WYLL036C
YGR129WYLR117C
YGR129WYMR213W
YGR130C YKL193C
YGR130C YMR117C
YGR131WYLR310C
YGR133WYMR211W
YGR134WYIL038C
YGR134WYNL288W
YGR134WYNR052C
YGR134WYPR072W
YGR135WYHR200W
YGR135WYML092C
YGR135WYNL244C
YGR135WYOR043W
YGR135WYOR261C
YGR135WYPL144W
YGR135WYPR103W
YGR136WYGR255C
YGR136WYIL095W
YGR136WYIL108W
YGR136WYIL156W
YGR136WYIR003W
YGR136WYJL012C
YGR136WYJR083C
YGR136WYKL182W
YGR136WYKR050W
YGR136WYLL021W
YGR136WYLR144C
YGR136WYLR149C
YGR136WYLR191W
YGR136WYML069W
YGR136WYMR192W
YGR136WYMR200W
YGR136WYNL082W
YGR136WYNL094W
YGR136WYNL176C

YGR136WYNL189W
YGR136WYNL293W
YGR136WYOL024W
YGR136WYOR042W
YGR136WYOR058C
YGR136WYOR113W
YGR136WYOR181W
YGR136WYOR197W
YGR136WYOR329C
YGR136WYPL038W
YGR136WYPL111W
YGR136WYPL249C
YGR136WYPR081C
YGR136WYPR171W
YGR139WYMR047C
YGR140WYGR179C
YGR140WYJR060W
YGR140WYJR089W
YGR140WYMR094W
YGR140WYNL307C
YGR140WYPL018W
YGR140WYPL209C
YGR141WYLR191W
YGR141WYMR265C
YGR142WYNL141W
YGR142WYOR232W
YGR144WYJR091C
YGR144WYLR347C
YGR144WYML064C
YGR144WYMR021C
YGR144WYNL116W
YGR144WYNL189W
YGR145WYIL035C
YGR145WYIL121W
YGR145WYMR116C
YGR145WYMR304W
YGR145WYNL132W
YGR146C YOR259C
YGR148C YLR295C
YGR149WYIR038C
YGR149WYNL219C
YGR151C YMR047C
YGR152C YOR374W
YGR152C YPL161C
YGR153WYLR295C

YGR153WYMR055C
YGR154C YHR135C
YGR155WYHR135C
YGR155WYHR170W
YGR155WYIL066C
YGR155WYJL173C
YGR155WYJR045C
YGR155WYKR026C
YGR155WYLR383W
YGR155WYLR442C
YGR155WYML064C
YGR155WYMR059W
YGR155WYNL189W
YGR155WYPL149W
YGR155WYPL204W
YGR156WYJL033W
YGR156WYJR093C
YGR156WYKL059C
YGR156WYKL129C
YGR156WYKR002W
YGR156WYLR115W
YGR156WYMR260C
YGR156WYNL317W
YGR156WYOR179C
YGR156WYPR107C
YGR158C YGR195W
YGR158C YJL124C
YGR158C YJR022W
YGR158C YLR288C
YGR158C YLR345W
YGR158C YNL232W
YGR158C YOL021C
YGR159C YKL193C
YGR159C YLR113W
YGR159C YNL053W
YGR160WYLR233C
YGR160WYPL051W
YGR161C YJL098W
YGR161C YKR028W
YGR161C-D YMR032W
YGR162WYGR285C
YGR162WYHL034C
YGR162WYIL035C
YGR162WYIL061C
YGR162WYIR001C

YGR162WYJL090C
YGR162WYJL138C
YGR162WYJR007W
YGR162WYJR059W
YGR162WYJR076C
YGR162WYLR175W
YGR162WYLR362W
YGR162WYLR427W
YGR162WYMR116C
YGR162WYMR125W
YGR162WYNL005C
YGR162WYNL016W
YGR162WYNL020C
YGR162WYNL030W
YGR162WYNL161W
YGR162WYNL175C
YGR162WYNL244C
YGR162WYNL262W
YGR162WYOL139C
YGR162WYOR243C
YGR162WYOR276W
YGR162WYPL178W
YGR162WYPR041W
YGR163WYGR203W
YGR163WYKR007W
YGR163WYML121W
YGR163WYPL235W
YGR165WYLR035C
YGR165WYPL265W
YGR166WYJR091C
YGR166WYKR068C
YGR166WYOR197W
YGR167WYHR161C
YGR167WYOR028C
YGR168C YIR018W
YGR170WYJL020C
YGR170WYNL030W
YGR172C YGR177C
YGR172C YJR091C
YGR172C YLR324W
YGR172C YLR452C
YGR172C YNL044W
YGR172C YNL263C
YGR172C YOL103W
YGR172C YPL095C

YGR173WYIL162W
YGR173WYJR045C
YGR173WYKL085W
YGR173WYKR086W
YGR173WYLR180W
YGR173WYLR259C
YGR173WYMR319C
YGR173WYNL064C
YGR173WYOL055C
YGR173WYPL061W
YGR174C YLL028W
YGR174C YOL115W
YGR174C YPR201W
YGR175C YGR289C
YGR175C YNL311C
YGR178C YGR218W
YGR178C YHR121W
YGR178C YJL035C
YGR178C YLR295C
YGR179C YMR168C
YGR179C YPL018W
YGR180C YJL026W
YGR180C YJL069C
YGR180C YJL115W
YGR180C YLR196W
YGR180C YML102W
YGR180C YNL244C
YGR180C YOR229W
YGR181WYJR135W-A
YGR181WYNR017W
YGR182C YJR091C
YGR182C YPL066W
YGR184C YHR135C
YGR184C YIL075C
YGR184C YJL141C
YGR184C YKL145W
YGR184C YNL307C
YGR184C YPL177C
YGR185C YGR229C
YGR185C YPL013C
YGR185C YPR106W
YGR186WYIL021W
YGR186WYJL164C
YGR186WYJR017C
YGR186WYMR277W

YGR186WYPL129W
YGR186WYPL203W
YGR187C YJR091C
YGR187C YLR295C
YGR187C YNL317W
YGR188C YGR254W
YGR188C YHR137W
YGR188C YJL034W
YGR188C YKL129C
YGR188C YLR044C
YGR188C YNL265C
YGR188C YOR026W
YGR188C YOR047C
YGR189C YNL092W
YGR191WYHL042W
YGR191WYIR038C
YGR191WYJL012C
YGR191WYJR117W
YGR191WYLR283W
YGR191WYLR372W
YGR191WYOR016C
YGR191WYPL218W
YGR192C YIR001C
YGR192C YKL129C
YGR192C YMR109W
YGR192C YNL189W
YGR192C YOR014W
YGR193C YMR106C
YGR193C YNL121C
YGR194C YNL265C
YGR195WYHR069C
YGR195WYJL109C
YGR195WYMR128W
YGR195WYNL189W
YGR195WYNL232W
YGR195WYOL021C
YGR195WYOL142W
YGR195WYOR001W
YGR195WYOR076C
YGR195WYOR326W
YGR196C YGR253C
YGR196C YIL131C
YGR196C YLR200W
YGR196C YNL094W
YGR196C YOR231W

YGR197C YOR327C
YGR198WYLR305C
YGR199WYHL042W
YGR200C YHR187W
YGR200C YJR127C
YGR200C YKL110C
YGR200C YLR384C
YGR200C YMR312W
YGR200C YNL078W
YGR200C YPL086C
YGR200C YPL101W
YGR202C YJL020C
YGR202C YNL189W
YGR203WYIL061C
YGR203WYJL076W
YGR203WYOR341W
YGR204WYHL002W
YGR204WYIL018W
YGR204WYKL198C
YGR204WYNL135C
YGR204WYPR086W
YGR205WYLL026W
YGR205WYLR210W
YGR205WYPR086W
YGR207C YPR086W
YGR208WYKL177W
YGR208WYPL011C
YGR209C YHR199C
YGR209C YPL020C
YGR210C YMR109W
YGR211WYMR199W
YGR211WYNL281W
YGR211WYOL133W
YGR211WYOR028C
YGR213C YHR134W
YGR213C YOR059C
YGR214WYIL009W
YGR215WYHL004W
YGR215WYHR114W
YGR217WYJL020C
YGR218WYGR262C
YGR218WYHR016C
YGR218WYHR082C
YGR218WYHR108W
YGR218WYIL063C

YGR218WYIL115C
YGR218WYJL210W
YGR218WYJR061W
YGR218WYJR083C
YGR218WYJR134C
YGR218WYKL045W
YGR218WYKL068W
YGR218WYKL143W
YGR218WYLL060C
YGR218WYLL067W-A
YGR218WYLR131C
YGR218WYLR151C
YGR218WYLR293C
YGR218WYLR436C
YGR218WYML007W
YGR218WYML117W
YGR218WYML120C
YGR218WYMR047C
YGR218WYMR124W
YGR218WYMR180C
YGR218WYNL118C
YGR218WYNL164C
YGR218WYNL196C
YGR218WYOL133W
YGR218WYOL149W
YGR218WYOR098C
YGR218WYOR147W
YGR218WYOR177C
YGR218WYOR184W
YGR218WYOR329C
YGR218WYOR371C
YGR218WYPL056C
YGR218WYPL120W
YGR220C YJL063C
YGR220C YKL167C
YGR220C YKR006C
YGR220C YKR085C
YGR220C YLR189C
YGR220C YLR371W
YGR220C YLR439W
YGR220C YML009C
YGR220C YML025C
YGR220C YMR193W
YGR220C YMR225C
YGR220C YNL005C

YGR220C YNL236W
YGR220C YPL183W-A
YGR222WYMR047C
YGR222WYNR045W
YGR223C YMR109W
YGR223C YOR089C
YGR223C YPL028W
YGR224WYKL020C
YGR224WYLR080W
YGR225WYKL022C
YGR227WYHR016C
YGR227WYHR114W
YGR227WYLR310C
YGR228WYOR245C
YGR229C YKR099W
YGR229C YPR110C
YGR232WYHR027C
YGR232WYHR200W
YGR232WYIL007C
YGR232WYIL075C
YGR232WYKL145W
YGR232WYLR421C
YGR232WYNL244C
YGR232WYOR117W
YGR232WYOR259C
YGR232WYOR261C
YGR232WYPR086W
YGR232WYPR108W
YGR233C YGR262C
YGR233C YIL050W
YGR233C YJL005W
YGR233C YLL050C
YGR233C YLR186W
YGR233C YOL001W
YGR233C YPL031C
YGR234WYJL074C
YGR234WYLL013C
YGR234WYML064C
YGR234WYNL106C
YGR234WYOL133W
YGR234WYPL031C
YGR234WYPL106C
YGR236C YOR299W
YGR236C YOR358W
YGR237C YLR128W

YGR238C YHR158C
YGR238C YJL187C
YGR238C YLR096W
YGR238C YOL069W
YGR239C YGR249W
YGR239C YIL160C
YGR239C YLR191W
YGR239C YLR234W
YGR239C YMR192W
YGR239C YMR267W
YGR239C YOR389W
YGR240C YHR102W
YGR240C YHR169W
YGR240C YHR196W
YGR240C YIL106W
YGR240C YJR045C
YGR240C YJR090C
YGR240C YKL166C
YGR240C YLR175W
YGR240C YLR212C
YGR240C YLR291C
YGR240C YLR370C
YGR240C YML058W
YGR240C YML064C
YGR240C YML085C
YGR240C YMR032W
YGR240C YMR059W
YGR240C YMR205C
YGR240C YMR223W
YGR240C YNL030W
YGR240C YNL045W
YGR240C YNL135C
YGR240C YNL244C
YGR240C YOL126C
YGR240C YOL133W
YGR240C YOR100C
YGR240C YPL140C
YGR240C YPL151C
YGR240C YPR110C
YGR241C YHR016C
YGR241C YIL095W
YGR241C YJR091C
YGR241C YNL020C
YGR241C YNL092W
YGR241C YNL183C

YGR241C YPR178W
YGR242WYPL051W
YGR243WYHR016C
YGR244C YPL118W
YGR245C YHR197W
YGR245C YKR048C
YGR245C YKR081C
YGR245C YLR074C
YGR245C YLR106C
YGR245C YNL002C
YGR245C YNL110C
YGR245C YNL182C
YGR245C YNR053C
YGR245C YPL093W
YGR245C YPR016C
YGR246C YNL039W
YGR246C YNR003C
YGR247WYOR327C
YGR249WYHL004W
YGR249WYKL057C
YGR249WYLR446W
YGR249WYNL092W
YGR249WYNL189W
YGR249WYOL063C
YGR250C YIR001C
YGR250C YJL057C
YGR250C YMR032W
YGR250C YNL025C
YGR252WYHR099W
YGR252WYJL008C
YGR252WYJR077C
YGR252WYMR223W
YGR252WYMR319C
YGR252WYNL030W
YGR252WYOR119C
YGR252WYOR225W
YGR252WYPL061W
YGR252WYPL181W
YGR252WYPL254W
YGR253C YJR017C
YGR253C YOR362C
YGR253C YPL144W
YGR253C YPR185W
YGR254WYHL007C
YGR254WYHR013C

YGR254WYIL147C
YGR254WYJL098W
YGR254WYJL128C
YGR254WYJR045C
YGR254WYKL018W
YGR254WYKL103C
YGR254WYKR026C
YGR254WYKR055W
YGR254WYLR019W
YGR254WYLR175W
YGR254WYLR196W
YGR254WYLR309C
YGR254WYLR314C
YGR254WYML016C
YGR254WYML115C
YGR254WYMR049C
YGR254WYMR117C
YGR254WYNL135C
YGR254WYNL244C
YGR254WYNR031C
YGR254WYNR036C
YGR254WYOL062C
YGR254WYOR061W
YGR254WYOR089C
YGR254WYOR174W
YGR254WYOR212W
YGR254WYOR269W
YGR254WYOR351C
YGR254WYPL031C
YGR254WYPL139C
YGR254WYPL203W
YGR254WYPL262W
YGR254WYPR110C
YGR254WYPR178W
YGR255C YLR310C
YGR255C YPR154W
YGR256WYMR059W
YGR256WYPL126W
YGR258C YIL143C
YGR258C YJL034W
YGR258C YJL088W
YGR258C YJL130C
YGR258C YJR045C
YGR258C YLR259C
YGR258C YOL044W

YGR260WYGR284C
YGR260WYHR026W
YGR260WYHR048W
YGR260WYHR110W
YGR260WYHR142W
YGR260WYIL016W
YGR260WYIL047C
YGR260WYJL117W
YGR260WYJL196C
YGR260WYJR117W
YGR260WYKL008C
YGR260WYKL065C
YGR260WYKL154W
YGR260WYKR052C
YGR260WYLR372W
YGR260WYML048W
YGR260WYML075C
YGR260WYMR150C
YGR260WYMR257C
YGR260WYMR299C
YGR260WYNL044W
YGR260WYOR016C
YGR260WYPL019C
YGR260WYPL076W
YGR260WYPR028W
YGR261C YHR174W
YGR261C YOR106W
YGR261C YPL115C
YGR261C YPL195W
YGR262C YHL034C
YGR262C YHR011W
YGR262C YHR027C
YGR262C YHR033W
YGR262C YHR205W
YGR262C YHR216W
YGR262C YIL075C
YGR262C YJL008C
YGR262C YJR045C
YGR262C YJR072C
YGR262C YJR077C
YGR262C YJR091C
YGR262C YJR121W
YGR262C YKL145W
YGR262C YKL152C
YGR262C YKR038C

YGR262C YKR048C
YGR262C YLR174W
YGR262C YLR180W
YGR262C YLR216C
YGR262C YLR259C
YGR262C YLR438W
YGR262C YML036W
YGR262C YML056C
YGR262C YML085C
YGR262C YML124C
YGR262C YMR205C
YGR262C YMR226C
YGR262C YNL014W
YGR262C YNL055C
YGR262C YNL064C
YGR262C YNL071W
YGR262C YOR073W
YGR262C YOR151C
YGR262C YPR181C
YGR263C YJR091C
YGR263C YLR433C
YGR263C YNL066W
YGR263C YOR180C
YGR264C YNL189W
YGR266WYMR106C
YGR267C YHR030C
YGR267C YHR135C
YGR267C YIR034C
YGR267C YJR090C
YGR267C YKL210W
YGR267C YLR347C
YGR267C YML064C
YGR267C YMR106C
YGR267C YNL189W
YGR267C YOL087C
YGR267C YOR014W
YGR267C YPL003W
YGR268C YHL002W
YGR268C YHR016C
YGR268C YJL020C
YGR268C YML064C
YGR268C YOR124C
YGR268C YOR138C
YGR268C YOR302W
YGR268C YPR154W

YGR269WYJR091C
YGR269WYOR159C
YGR270WYOR039W
YGR270WYPL115C
YGR270WYPL153C
YGR271WYJL095W
YGR271WYMR036C
YGR274C YHR023W
YGR274C YIL038C
YGR274C YKL201C
YGR274C YKR001C
YGR274C YML015C
YGR274C YML098W
YGR274C YMR005W
YGR274C YMR012W
YGR274C YMR227C
YGR274C YMR236W
YGR274C YNL236W
YGR274C YPL011C
YGR274C YPL128C
YGR274C YPL235W
YGR274C YPR072W
YGR275WYML115C
YGR275WYPR086W
YGR276C YNR015W
YGR277C YLL061W
YGR278WYHR165C
YGR278WYKL095W
YGR278WYKL182W
YGR278WYMR213W
YGR278WYNL071W
YGR279C YLR191W
YGR279C YLR291C
YGR280C YKR092C
YGR280C YMR102C
YGR280C YMR302C
YGR280C YNL189W
YGR280C YOR014W
YGR280C YOR061W
YGR280C YPL110C
YGR281WYHR114W
YGR281WYPR021C
YGR282C YIL066C
YGR282C YJR022W
YGR282C YJR068W

YGR282C YKR026C
YGR282C YML064C
YGR282C YMR106C
YGR282C YNL012W
YGR282C YPL140C
YGR282C YPL256C
YGR282C YPR111W
YGR283C YNL281W
YGR284C YGR289C
YGR284C YHL048W
YGR284C YHR026W
YGR284C YHR110W
YGR284C YHR140W
YGR284C YHR142W
YGR284C YJR010C-A
YGR284C YLL023C
YGR284C YLL061W
YGR284C YLR018C
YGR284C YML067C
YGR284C YMR058W
YGR284C YMR215W
YGR284C YNL101W
YGR284C YOL132W
YGR284C YPL076W
YGR284C YPL264C
YGR284C YPL274W
YGR284C YPR156C
YGR285C YHR064C
YGR285C YJL080C
YGR285C YJL172W
YGR285C YLR295C
YGR285C YMR116C
YGR285C YPR189W
YGR289C YHL048W
YGR289C YIL016W
YGR289C YKL154W
YGR289C YLR372W
YGR289C YOL003C
YGR289C YPR028W
YGR290WYLR147C
YGR291C YPR086W
YGR292WYLR054C
YGR294WYHL018W
YGR294WYJR091C
YGR294WYMR294W

YGR294WYOR299W
YGR294WYPR086W
YGR295C YHR007C
YGR295C YKL004W
YGR295C YLR237W
YGR295C YLR343W
YGR295C YLR372W
YGR295C YML048W
YGR295C YMR221C
YGR295C YNL130C
YGR295C YOR079C
YGR296WYJL020C
YGR296WYKL011C
YHL002WYHL034C
YHL002WYIL095W
YHL002WYIL144W
YHL002WYIL148W
YHL002WYIL156W
YHL002WYIR003W
YHL002WYJL056C
YHL002WYJR054W
YHL002WYKL213C
YHL002WYKR009C
YHL002WYLL015W
YHL002WYLR144C
YHL002WYML099C
YHL002WYMR004W
YHL002WYMR089C
YHL002WYMR109W
YHL002WYMR189W
YHL002WYMR275C
YHL002WYNL015W
YHL002WYNL094W
YHL002WYNL239W
YHL002WYNL271C
YHL002WYNR005C
YHL002WYNR006W
YHL002WYNR016C
YHL002WYNR052C
YHL002WYOR067C
YHL002WYOR076C
YHL002WYOR181W
YHL002WYOR227W
YHL002WYOR355W
YHL002WYPL038W

YHL002W	YPL144W
YHL002W	YPR115W
YHL002W	YPR171W
YHL003C	YHL048W
YHL003C	YHR110W
YHL003C	YHR142W
YHL003C	YJR010C-A
YHL003C	YKL008C
YHL003C	YMR047C
YHL003C	YMR149W
YHL003C	YMR298W
YHL003C	YNL101W
YHL003C	YOR092W
YHL004W	YIL093C
YHL004W	YJR101W
YHL004W	YJR113C
YHL004W	YKL155C
YHL004W	YLR423C
YHL004W	YMR153W
YHL004W	YMR158W
YHL004W	YMR186W
YHL004W	YMR188C
YHL004W	YNL137C
YHL004W	YNL306W
YHL004W	YNR037C
YHL004W	YOR158W
YHL004W	YOR212W
YHL004W	YOR243C
YHL004W	YPL118W
YHL004W	YPL255W
YHL006C	YLR046C
YHL006C	YLR134W
YHL006C	YLR376C
YHL006C	YNL021W
YHL006C	YPL255W
YHL007C	YHR114W
YHL007C	YJL020C
YHL007C	YJL052W
YHL007C	YJL203W
YHL007C	YJR009C
YHL007C	YJR045C
YHL007C	YKL129C
YHL007C	YLR191W
YHL007C	YLR229C
YHL007C	YLR353W

YHL007C	YMR109W
YHL007C	YMR186W
YHL007C	YNL161W
YHL007C	YOR299W
YHL007C	YPL022W
YHL007C	YPL256C
YHL009C	YMR047C
YHL009C	YNL189W
YHL010C	YKR017C
YHL010C	YLR138W
YHL011C	YKL181W
YHL011C	YMR139W
YHL011C	YNL189W
YHL011C	YOR090C
YHL015W	YLL031C
YHL015W	YOR350C
YHL017W	YHR188C
YHL017W	YIL111W
YHL017W	YJL004C
YHL017W	YLR453C
YHL018W	YJL098W
YHL018W	YLR291C
YHL018W	YNL113W
YHL018W	YNL189W
YHL018W	YNL207W
YHL018W	YPL070W
YHL019C	YKL135C
YHL019C	YLR170C
YHL019C	YPR029C
YHL020C	YOL004W
YHL021C	YJL140W
YHL021C	YOR107W
YHL021C	YOR210W
YHL022C	YLR329W
YHL023C	YLR096W
YHL023C	YMR032W
YHL023C	YNL307C
YHL023C	YOL133W
YHL024W	YJL063C
YHL025W	YJL176C
YHL025W	YLR438W
YHL025W	YML092C
YHL025W	YNR023W
YHL025W	YOR119C
YHL026C	YHR005C

YHL026C	YJR091C
YHL026C	YOL064C
YHL027W	YHR016C
YHL027W	YIL131C
YHL027W	YJL056C
YHL027W	YLR191W
YHL027W	YMR032W
YHL027W	YOR275C
YHL030W	YHR200W
YHL030W	YKL145W
YHL030W	YML092C
YHL030W	YNL132W
YHL030W	YOL133W
YHL030W	YOR261C
YHL030W	YPR103W
YHL030W	YPR110C
YHL031C	YLR026C
YHL032C	YIL053W
YHL032C	YKR026C
YHL033C	YJR091C
YHL034C	YHR135C
YHL034C	YHR169W
YHL034C	YIL035C
YHL034C	YIL131C
YHL034C	YIR001C
YHL034C	YJL092W
YHL034C	YLR074C
YHL034C	YLR233C
YHL034C	YLR247C
YHL034C	YLR263W
YHL034C	YLR427W
YHL034C	YMR137C
YHL034C	YNL088W
YHL034C	YNL230C
YHL034C	YOL102C
YHL034C	YOL115W
YHL034C	YOL139C
YHL034C	YPL204W
YHL035C	YHR114W
YHL035C	YIL035C
YHL035C	YLR115W
YHL035C	YOR327C
YHL036W	YPR163C
YHL039W	YLR208W
YHL042W	YHR032W

YHL042W	YIL006W
YHL042W	YIL016W
YHL042W	YJL117W
YHL042W	YJR010C-A
YHL042W	YJR117W
YHL042W	YKL065C
YHL042W	YLR018C
YHL042W	YLR372W
YHL042W	YML048W
YHL042W	YMR264W
YHL042W	YNL003C
YHL042W	YNL329C
YHL042W	YOR016C
YHL042W	YOR127W
YHL042W	YOR254C
YHL042W	YPL076W
YHL042W	YPL264C
YHL042W	YPL265W
YHL042W	YPR028W
YHL043W	YMR047C
YHL043W	YMR232W
YHL044W	YHR114W
YHL044W	YKR035C
YHL044W	YPR086W
YHL045W	YKR006C
YHL045W	YPR086W
YHL046C	YOR355W
YHL047C	YHR133C
YHL048W	YHR026W
YHL048W	YHR140W
YHL048W	YHR142W
YHL048W	YIL016W
YHL048W	YJL002C
YHL048W	YJL091C
YHL048W	YJL108C
YHL048W	YJL117W
YHL048W	YJL196C
YHL048W	YJR117W
YHL048W	YKL004W
YHL048W	YKL008C
YHL048W	YKL065C
YHL048W	YKL079W
YHL048W	YKL154W
YHL048W	YKR088C
YHL048W	YLR372W

YHL048W	YML048W
YHL048W	YMR221C
YHL048W	YMR264W
YHL048W	YNL130C
YHL048W	YOL003C
YHL048W	YOR016C
YHL048W	YOR254C
YHL048W	YOR378W
YHL048W	YPL076W
YHL048W	YPL092W
YHL048W	YPR028W
YHL048W	YPR156C
YHL049C	YMR047C
YHL049C	YOL099C
YHR001W	YKR036C
YHR003C	YJR091C
YHR003C	YMR203W
YHR005C	YIL148W
YHR005C	YJL080C
YHR005C	YLL024C
YHR005C	YLR362W
YHR005C	YLR452C
YHR005C	YMR069W
YHR005C	YOR178C
YHR005C	YOR212W
YHR005C-A	YJL054W
YHR005C-A	YKL195W
YHR005C-A	YNR017W
YHR005C-A	YOL034W
YHR007C	YJR117W
YHR007C	YKL008C
YHR007C	YKL201C
YHR007C	YKR050W
YHR007C	YLL006W
YHR007C	YLL048C
YHR007C	YLR242C
YHR007C	YNL070W
YHR007C	YNL101W
YHR007C	YPL076W
YHR007C	YPL274W
YHR008C	YML095C
YHR008C	YMR106C
YHR009C	YLR447C
YHR009C	YOR359W
YHR011W	YIL063C

YHR011W	YJL069C
YHR011W	YPR004C
YHR012W	YJL053W
YHR012W	YJL154C
YHR012W	YJL187C
YHR012W	YOL145C
YHR012W	YOR069W
YHR013C	YHR023W
YHR013C	YMR116C
YHR013C	YOR014W
YHR014W	YHR185C
YHR014W	YJL138C
YHR014W	YKL152C
YHR014W	YLR259C
YHR014W	YMR001C
YHR014W	YOR136W
YHR015W	YMR001C
YHR015W	YPL169C
YHR016C	YIL095W
YHR016C	YIL156W
YHR016C	YJL004C
YHR016C	YJL151C
YHR016C	YJL194W
YHR016C	YJL201W
YHR016C	YJR083C
YHR016C	YKL109W
YHR016C	YKL129C
YHR016C	YKR030W
YHR016C	YLL005C
YHR016C	YLL023C
YHR016C	YLL028W
YHR016C	YLR064W
YHR016C	YLR144C
YHR016C	YLR206W
YHR016C	YLR243W
YHR016C	YLR426W
YHR016C	YMR109W
YHR016C	YMR192W
YHR016C	YMR255W
YHR016C	YMR313C
YHR016C	YNL084C
YHR016C	YNL094W
YHR016C	YNL243W
YHR016C	YNR007C
YHR016C	YOL070C

YHR016C YOR042W
YHR016C YOR109W
YHR016C YOR181W
YHR016C YOR247W
YHR016C YOR284W
YHR016C YPL038W
YHR016C YPL246C
YHR016C YPL249C
YHR016C YPR052C
YHR016C YPR055W
YHR016C YPR081C
YHR016C YPR171W
YHR018C YNL189W
YHR018C YNL244C
YHR019C YHR204W
YHR019C YIR005W
YHR019C YKL095W
YHR019C YKL198C
YHR019C YNL244C
YHR019C YOR080W
YHR020W YJL098W
YHR020W YKR026C
YHR020W YLR175W
YHR020W YLR222C
YHR020W YLR291C
YHR020W YMR022W
YHR020W YNL244C
YHR020W YOL094C
YHR020W YOL126C
YHR020W YOR212W
YHR020W YOR351C
YHR020W YPL203W
YHR020W YPR110C
YHR022C YIL028W
YHR022C YKL152C
YHR022C YLR259C
YHR022C YNR005C
YHR023W YIL061C
YHR023W YLR423C
YHR023W YOR326W
YHR023W YPR032W
YHR023W YPR188C
YHR024C YHR120W
YHR024C YIL066C
YHR024C YLR163C

YHR025W	YML064C
YHR025W	YNL189W
YHR026W	YIL088C
YHR026W	YJR091C
YHR026W	YKL065C
YHR026W	YKL221W
YHR026W	YLL028W
YHR026W	YLR034C
YHR026W	YLR372W
YHR026W	YMR149W
YHR026W	YOR016C
YHR026W	YOR092W
YHR026W	YOR374W
YHR026W	YPR156C
YHR026W	YPR198W
YHR027C	YHR169W
YHR027C	YIL075C
YHR027C	YJR017C
YHR027C	YJR076C
YHR027C	YKL145W
YHR027C	YKL171W
YHR027C	YMR001C
YHR027C	YMR276W
YHR027C	YNL244C
YHR027C	YOL133W
YHR027C	YOR117W
YHR027C	YOR181W
YHR027C	YOR259C
YHR027C	YOR261C
YHR027C	YOR351C
YHR027C	YPL140C
YHR027C	YPL259C
YHR027C	YPR108W
YHR030C	YHR084W
YHR030C	YHR102W
YHR030C	YHR179W
YHR030C	YHR183W
YHR030C	YHR193C
YHR030C	YIL094C
YHR030C	YIL106W
YHR030C	YIL113W
YHR030C	YJL066C
YHR030C	YJL095W
YHR030C	YJL130C
YHR030C	YJL138C

YHR030C YJR077C
YHR030C YJR121W
YHR030C YKL073W
YHR030C YKL152C
YHR030C YLL021W
YHR030C YLR096W
YHR030C YLR147C
YHR030C YLR175W
YHR030C YLR180W
YHR030C YLR182W
YHR030C YLR187W
YHR030C YLR216C
YHR030C YLR262C
YHR030C YLR268W
YHR030C YLR346C
YHR030C YLR350W
YHR030C YLR355C
YHR030C YLR371W
YHR030C YLR446W
YHR030C YML064C
YHR030C YMR116C
YHR030C YMR205C
YHR030C YMR214W
YHR030C YMR276W
YHR030C YNL007C
YHR030C YNL037C
YHR030C YNL053W
YHR030C YNL064C
YHR030C YNL085W
YHR030C YNR001C
YHR030C YNR031C
YHR030C YOL055C
YHR030C YOL123W
YHR030C YOR080W
YHR030C YOR208W
YHR030C YOR220W
YHR030C YOR231W
YHR030C YOR353C
YHR030C YPL049C
YHR030C YPL089C
YHR030C YPL140C
YHR030C YPL240C
YHR030C YPR047W
YHR030C YPR054W
YHR030C YPR088C

YHR030C	YPR111W
YHR030C	YPR191W
YHR033W	YJR017C
YHR033W	YJR076C
YHR033W	YKL166C
YHR033W	YML064C
YHR033W	YMR106C
YHR033W	YOR212W
YHR033W	YPL022W
YHR034C	YJR022W
YHR034C	YLR241W
YHR035W	YJR022W
YHR035W	YLL013C
YHR035W	YLR259C
YHR035W	YPR119W
YHR038W	YML064C
YHR038W	YMR047C
YHR038W	YPR086W
YHR039C	YOR264W
YHR039C-A	YLR216C
YHR039C-A	YMR054W
YHR039C-A	YOR270C
YHR039C-A	YOR332W
YHR040W	YNL206C
YHR040W	YPR086W
YHR041C	YJL015C
YHR041C	YJL086C
YHR041C	YLR164W
YHR041C	YLR309C
YHR041C	YLR344W
YHR041C	YNL334C
YHR041C	YOL051W
YHR041C	YOL135C
YHR041C	YOR073W
YHR041C	YOR159C
YHR041C	YPL248C
YHR041C	YPR203W
YHR042W	YHR140W
YHR042W	YPL092W
YHR043C	YHR107C
YHR043C	YLR423C
YHR044C	YHR107C
YHR044C	YIL061C
YHR044C	YLR096W
YHR045W	YOR128C

YHR046C	YPR015C
YHR047C	YLR148W
YHR048W	YNL192W
YHR049W	YJR091C
YHR050W	YJR015W
YHR050W	YKR089C
YHR050W	YMR149W
YHR050W	YOR057W
YHR051W	YNL061W
YHR052W	YHR066W
YHR052W	YHR197W
YHR052W	YIL035C
YHR052W	YIR026C
YHR052W	YJL095W
YHR052W	YKL172W
YHR052W	YKR081C
YHR052W	YLR074C
YHR052W	YMR049C
YHR052W	YMR093W
YHR052W	YMR229C
YHR052W	YNL061W
YHR052W	YNL110C
YHR052W	YNL132W
YHR052W	YNL175C
YHR052W	YNL230C
YHR052W	YOR005C
YHR052W	YOR061W
YHR052W	YOR080W
YHR052W	YOR206W
YHR052W	YOR267C
YHR052W	YOR272W
YHR052W	YOR310C
YHR052W	YPL043W
YHR052W	YPL131W
YHR052W	YPL141C
YHR052W	YPL259C
YHR053C	YKL023W
YHR056C	YLL018C
YHR056C	YNL258C
YHR057C	YJR091C
YHR057C	YKL023W
YHR057C	YLR373C
YHR058C	YKL023W
YHR058C	YOL051W
YHR058C	YOL135C

YHR058C YOR119C
YHR058C YPR086W
YHR059W YLR074C
YHR060W YHR107C
YHR060W YJR091C
YHR060W YKL119C
YHR060W YKR034W
YHR060W YLR447C
YHR060W YOR270C
YHR060W YPL019C
YHR060W YPR105C
YHR061C YHR107C
YHR061C YKL082C
YHR061C YLR229C
YHR061C YML109W
YHR061C YMR032W
YHR061C YMR055C
YHR061C YMR238W
YHR061C YMR273C
YHR061C YNL298W
YHR061C YPL161C
YHR062C YOR176W
YHR063C YPR180W
YHR064C YIR005W
YHR064C YNL135C
YHR064C YNL244C
YHR064C YOR080W
YHR065C YMR106C
YHR066W YHR088W
YHR066W YIL035C
YHR066W YKL014C
YHR066W YKL172W
YHR066W YKR081C
YHR066W YLL008W
YHR066W YLR249W
YHR066W YLR276C
YHR066W YMR049C
YHR066W YMR290C
YHR066W YNL002C
YHR066W YNL061W
YHR066W YOR080W
YHR066W YOR206W
YHR066W YOR272W
YHR066W YPL043W
YHR066W YPL093W

YHR066W YPL211W
YHR066W YPR016C
YHR067W YOR264W
YHR068W YKL135C
YHR068W YNL189W
YHR068W YOR098C
YHR069C YHR081W
YHR069C YLR163C
YHR069C YNL189W
YHR069C YNL232W
YHR069C YOL021C
YHR069C YOR076C
YHR070W YJL066C
YHR070W YJR045C
YHR070W YNL138W
YHR071W YPL031C
YHR072W YLR191W
YHR072W-A YHR089C
YHR072W-A YLR175W
YHR072W-A YNL124W
YHR073W YHR152W
YHR073W YPL043W
YHR074W YIL092W
YHR074W YLR216C
YHR075C YLR368W
YHR076W YJL076W
YHR076W YJR035W
YHR076W YKL108W
YHR076W YMR079W
YHR076W YMR137C
YHR077C YKL023W
YHR077C YLR263W
YHR077C YMR080C
YHR077C YNR023W
YHR077C YOR076C
YHR078W YMR150C
YHR079C YIL061C
YHR079C YML075C
YHR079C-A YJR091C
YHR079C-A YPR086W
YHR081W YNL092W
YHR081W YOL021C
YHR081W YOR001W
YHR082C YHR099W
YHR082C YHR119W

YHR082C	YHR186C
YHR082C	YJL020C
YHR082C	YJL087C
YHR082C	YJL090C
YHR082C	YJL095W
YHR082C	YJL098W
YHR082C	YJL106W
YHR082C	YJL173C
YHR082C	YJR007W
YHR082C	YJR066W
YHR082C	YJR076C
YHR082C	YKL045W
YHR082C	YKL161C
YHR082C	YKR024C
YHR082C	YLR096W
YHR082C	YLR233C
YHR082C	YLR259C
YHR082C	YML025C
YHR082C	YML057W
YHR082C	YMR137C
YHR082C	YNL006W
YHR082C	YNL094W
YHR082C	YNL161W
YHR082C	YNL192W
YHR082C	YNL201C
YHR082C	YNL307C
YHR082C	YNL311C
YHR082C	YOR005C
YHR082C	YOR043W
YHR082C	YOR080W
YHR082C	YPL150W
YHR082C	YPL153C
YHR082C	YPL180W
YHR082C	YPL203W
YHR082C	YPR093C
YHR082C	YPR104C
YHR083W	YNL092W
YHR084W	YKL161C
YHR084W	YLR310C
YHR084W	YMR043W
YHR084W	YPL049C
YHR085W	YHR197W
YHR085W	YLR074C
YHR085W	YLR356W
YHR086W	YIL061C

YHR086W	YKL012W
YHR086W	YKL173W
YHR086W	YLR147C
YHR086W	YLR249W
YHR086W	YLR275W
YHR086W	YLR298C
YHR086W	YML046W
YHR086W	YMR125W
YHR086W	YMR257C
YHR086W	YNL189W
YHR086W	YPL178W
YHR087W	YIL118W
YHR087W	YLR340W
YHR087W	YMR216C
YHR088W	YMR290C
YHR088W	YNL061W
YHR088W	YNL110C
YHR088W	YOR267C
YHR088W	YOR272W
YHR088W	YPR016C
YHR089C	YJR022W
YHR089C	YKL014C
YHR089C	YLR175W
YHR089C	YMR047C
YHR089C	YMR239C
YHR089C	YNL005C
YHR089C	YNL061W
YHR089C	YNL124W
YHR089C	YNL262W
YHR089C	YPL012W
YHR089C	YPL043W
YHR090C	YHR099W
YHR090C	YKR020W
YHR090C	YNL107W
YHR090C	YOR244W
YHR091C	YOL088C
YHR092C	YLR292C
YHR092C	YPR194C
YHR094C	YHR122W
YHR094C	YJL012C
YHR094C	YKL065C
YHR094C	YLL006W
YHR094C	YLR372W
YHR094C	YML048W
YHR096C	YLL020C

YHR096C YML048W
YHR097C YMR001C
YHR098C YJL020C
YHR098C YJL199C
YHR098C YJR048W
YHR098C YLR026C
YHR098C YPL204W
YHR098C YPR181C

YHR099W	YIR009W
YHR099W	YJL081C
YHR099W	YJL098W
YHR099W	YJR082C
YHR099W	YKL062W
YHR099W	YLR055C
YHR099W	YMR223W
YHR099W	YMR236W
YHR099W	YNL107W
YHR099W	YNL136W
YHR099W	YNL189W
YHR099W	YOL012C
YHR099W	YOL083W
YHR099W	YOL148C
YHR099W	YOR119C
YHR099W	YOR244W
YHR099W	YPL047W
YHR099W	YPL254W
YHR099W	YPR023C
YHR102W	YJL008C
YHR102W	YKL189W
YHR102W	YKR086W
YHR102W	YLR180W
YHR102W	YLR315W
YHR102W	YLR365W
YHR102W	YLR368W
YHR102W	YMR032W
YHR102W	YOL086C
YHR102W	YOR257W
YHR102W	YOR353C
YHR102W	YPL018W
YHR102W	YPR160W
YHR103W	YLR191W
YHR103W	YMR183C
YHR103W	YNR039C
YHR105W	YLL040C
YHR105W	YNL263C

YHR105W	YOR034C
YHR105W	YPL246C
YHR106W	YLR368W
YHR107C	YIL144W
YHR107C	YJR045C
YHR107C	YJR076C
YHR107C	YJR121W
YHR107C	YKL056C
YHR107C	YKL060C
YHR107C	YKL152C
YHR107C	YLR058C
YHR107C	YLR147C
YHR107C	YLR314C
YHR107C	YML056C
YHR107C	YMR117C
YHR107C	YMR198W
YHR107C	YNL073W
YHR107C	YNL166C
YHR107C	YNL298W
YHR107C	YNR001C
YHR107C	YOL055C
YHR107C	YOR374W
YHR107C	YPL161C
YHR107C	YPL235W
YHR107C	YPL240C
YHR107C	YPL241C
YHR108W	YIL094C
YHR109W	YJL010C
YHR110W	YJL196C
YHR110W	YKL065C
YHR110W	YLR372W
YHR110W	YML048W
YHR110W	YNL130C
YHR110W	YOR016C
YHR111W	YIL008W
YHR111W	YIL132C
YHR111W	YML064C
YHR112C	YHR135C
YHR112C	YML064C
YHR112C	YNL189W
YHR112C	YPL022W
YHR112C	YPR110C
YHR113W	YML064C
YHR113W	YOL082W
YHR113W	YPR145W

YHR114W	YHR133C
YHR114W	YHR165C
YHR114W	YHR172W
YHR114W	YIL008W
YHR114W	YIL013C
YHR114W	YIL045W
YHR114W	YIL132C
YHR114W	YIL144W
YHR114W	YIL156W
YHR114W	YIL159W
YHR114W	YIR003W
YHR114W	YIR021W
YHR114W	YIR023W
YHR114W	YJL010C
YHR114W	YJL012C
YHR114W	YJL020C
YHR114W	YJL047C
YHR114W	YJL065C
YHR114W	YJL086C
YHR114W	YJL117W
YHR114W	YJL134W
YHR114W	YJL151C
YHR114W	YJL152W
YHR114W	YJL159W
YHR114W	YJL174W
YHR114W	YJL180C
YHR114W	YJL203W
YHR114W	YJR090C
YHR114W	YJR108W
YHR114W	YKL007W
YHR114W	YKL129C
YHR114W	YKL157W
YHR114W	YKL175W
YHR114W	YKL177W
YHR114W	YKL198C
YHR114W	YKL204W
YHR114W	YKR030W
YHR114W	YKR032W
YHR114W	YLL005C
YHR114W	YLL054C
YHR114W	YLR024C
YHR114W	YLR064W
YHR114W	YLR078C
YHR114W	YLR081W
YHR114W	YLR105C

YHR114W	YLR112W
YHR114W	YLR127C
YHR114W	YLR136C
YHR114W	YLR144C
YHR114W	YLR309C
YHR114W	YLR324W
YHR114W	YLR337C
YHR114W	YLR425W
YHR114W	YLR426W
YHR114W	YLR461W
YHR114W	YML008C
YHR114W	YML092C
YHR114W	YML121W
YHR114W	YML131W
YHR114W	YMR109W
YHR114W	YMR162C
YHR114W	YMR232W
YHR114W	YMR253C
YHR114W	YMR313C
YHR114W	YNL078W
YHR114W	YNL084C
YHR114W	YNL092W
YHR114W	YNL094W
YHR114W	YNL138W
YHR114W	YNL271C
YHR114W	YNL280C
YHR114W	YNR007C
YHR114W	YNR016C
YHR114W	YNR035C
YHR114W	YOL002C
YHR114W	YOL018C
YHR114W	YOL082W
YHR114W	YOR059C
YHR114W	YOR109W
YHR114W	YOR175C
YHR114W	YOR181W
YHR114W	YOR224C
YHR114W	YOR231W
YHR114W	YOR281C
YHR114W	YOR329C
YHR114W	YOR355W
YHR114W	YOR389W
YHR114W	YPL014W
YHR114W	YPL032C
YHR114W	YPL049C

YHR114W	YPL057C
YHR114W	YPL063W
YHR114W	YPL111W
YHR114W	YPL124W
YHR114W	YPL152W
YHR114W	YPL174C
YHR114W	YPL179W
YHR114W	YPL246C
YHR114W	YPL277C
YHR114W	YPR052C
YHR114W	YPR094W
YHR114W	YPR171W
YHR115C	YLR215C
YHR115C	YNL116W
YHR115C	YNL311C
YHR115C	YOR215C
YHR117W	YOR110W
YHR118C	YJL194W
YHR118C	YLL004W
YHR118C	YML065W
YHR118C	YNL261W
YHR118C	YPR162C
YHR119W	YJL130C
YHR119W	YJR045C
YHR119W	YKL018W
YHR119W	YLR015W
YHR119W	YLR288C
YHR119W	YLR447C
YHR119W	YOL055C
YHR119W	YOR374W
YHR119W	YPL138C
YHR120W	YJL066C
YHR120W	YJR045C
YHR120W	YKL152C
YHR120W	YLR163C
YHR120W	YLR180W
YHR120W	YLR259C
YHR120W	YNL064C
YHR121W	YHR178W
YHR121W	YLL013C
YHR121W	YMR001C
YHR121W	YNL207W
YHR121W	YOL128C
YHR122W	YHR164C
YHR123W	YIR038C

YHR123W	YKL065C
YHR123W	YLR372W
YHR123W	YNL130C
YHR123W	YPL227C
YHR124W	YOL133W
YHR127W	YLR295C
YHR128W	YHR152W
YHR128W	YLR056W
YHR128W	YLR295C
YHR128W	YNR012W
YHR128W	YPR185W

YHR129C YJR008W
YHR129C YJR091C
YHR129C YMR294W
YHR129C YOR098C
YHR129C YPL174C
YHR130C YJR091C
YHR130C YNL005C
YHR132C YLR447C
YHR132C YNL291C
YHR133C YHR140W
YHR133C YIL016W
YHR133C YJL117W
YHR133C YLR343W
YHR133C YLR372W
YHR133C YML048W
YHR133C YML075C
YHR133C YMR149W
YHR133C YMR215W
YHR133C YNL131W
YHR133C YNL279W
YHR133C YNL321W
YHR133C YOR016C
YHR133C YOR181W
YHR133C YPL227C
YHR133C YPL274W
YHR133C YPR028W
YHR135C YHR179W
YHR135C YHR183W
YHR135C YIL033C
YHR135C YIL115C
YHR135C YIR034C
YHR135C YJL039C
YHR135C YJL041W
YHR135C YJL061W

YHR135C YJL138C
YHR135C YJL207C
YHR135C YJR045C
YHR135C YJR104C
YHR135C YJR121W
YHR135C YKL152C
YHR135C YKL166C
YHR135C YKL210W
YHR135C YLR044C
YHR135C YLR109W
YHR135C YLR134W
YHR135C YLR180W
YHR135C YLR259C
YHR135C YLR355C
YHR135C YLR438W
YHR135C YML103C
YHR135C YMR186W
YHR135C YMR226C
YHR135C YMR303C
YHR135C YNL025C
YHR135C YNL055C
YHR135C YNL093W
YHR135C YNL116W
YHR135C YNL139C
YHR135C YNL154C
YHR135C YNL172W
YHR135C YNL334C
YHR135C YNR001C
YHR135C YOL055C
YHR135C YOR089C
YHR135C YOR177C
YHR135C YPL061W
YHR135C YPL169C
YHR135C YPL171C
YHR135C YPL203W
YHR135C YPL258C
YHR135C YPR115W
YHR135C YPR121W
YHR136C YMR246W
YHR136C YOR285W
YHR137W YJL128C
YHR137W YMR152W
YHR137W YNL183C
YHR137W YOR089C
YHR137W YOR351C

YHR138C YLR295C	
YHR140W	YIL016W
YHR140W	YIL023C
YHR140W	YIL114C
YHR140W	YJL117W
YHR140W	YJL196C
YHR140W	YJL214W
YHR140W	YJR117W
YHR140W	YKL006C-A
YHR140W	YKL065C
YHR140W	YKL154W
YHR140W	YLL028W
YHR140W	YLR018C
YHR140W	YLR295C
YHR140W	YLR343W
YHR140W	YLR372W
YHR140W	YML048W
YHR140W	YMR058W
YHR140W	YMR215W
YHR140W	YMR279C
YHR140W	YNL044W
YHR140W	YNL048W
YHR140W	YNL101W
YHR140W	YOL003C
YHR140W	YOL132W
YHR140W	YOR016C
YHR140W	YOR254C
YHR140W	YPL076W
YHR140W	YPL227C
YHR140W	YPL264C
YHR140W	YPR028W
YHR140W	YPR156C
YHR142W	YIL016W
YHR142W	YJL117W
YHR142W	YJL131C
YHR142W	YJL196C
YHR142W	YJR015W
YHR142W	YJR117W
YHR142W	YKL127W
YHR142W	YKL154W
YHR142W	YLR372W
YHR142W	YML048W
YHR142W	YNR013C
YHR142W	YOR316C
YHR143W-A	YIL021W

YHR143W-A YIL025C
YHR143W-A YPL203W
YHR143W-A YPR010C
YHR144C YJL090C
YHR144C YLR191W
YHR144C YPL070W
YHR145C YNL012W
YHR147C YNL284C
YHR148W YJR002W
YHR149C YLR448W
YHR149C YML109W
YHR149C YMR273C
YHR151C YNL193W
YHR152W YJL168C
YHR152W YJR045C
YHR152W YJR148W
YHR152W YLR016C
YHR152W YLR132C
YHR152W YLR288C
YHR152W YMR308C
YHR152W YNL138W
YHR152W YOR173W
YHR152W YPR152C
YHR154W YLR320W
YHR156C YHR165C
YHR156C YJR010W
YHR156C YJR091C
YHR156C YMR235C
YHR156C YOR181W
YHR157W YLR329W
YHR158C YJL187C
YHR158C YJR122W
YHR158C YKR054C
YHR158C YLR096W
YHR158C YLR452C
YHR158C YLR453C
YHR158C YMR036C
YHR158C YMR181C
YHR158C YNL084C
YHR158C YOR047C
YHR158C YOR269W
YHR159W YKR044W
YHR160C YIL160C
YHR160C YLR191W
YHR161C YIL095W

YHR161C YIR006C
YHR161C YMR183C
YHR161C YNL020C
YHR161C YPL077C
YHR161C YPL232W
YHR164C YKL113C
YHR165C YIL061C
YHR165C YIL148W
YHR165C YIR004W
YHR165C YJR022W
YHR165C YKL129C
YHR165C YKL152C
YHR165C YKL173W
YHR165C YLL036C
YHR165C YLR116W
YHR165C YLR117C
YHR165C YLR147C
YHR165C YLR424W
YHR165C YLR438C-A
YHR165C YML046W
YHR165C YML049C
YHR165C YMR109W
YHR165C YMR213W
YHR165C YNR053C
YHR165C YOR308C
YHR165C YPR178W
YHR166C YJR121W
YHR166C YKL022C
YHR166C YLR102C
YHR166C YLR127C
YHR166C YLR451W
YHR166C YNL172W
YHR166C YOR249C
YHR166C YPL124W
YHR166C YPR119W
YHR167W YNL139C
YHR167W YOL015W
YHR168W YNL260C
YHR169W YHR179W
YHR169W YIL125W
YHR169W YJL026W
YHR169W YJL138C
YHR169W YJR045C
YHR169W YJR109C
YHR169W YKL075C

YHR169W	YLR259C
YHR169W	YLR438W
YHR169W	YMR105C
YHR169W	YMR205C
YHR169W	YNL014W
YHR169W	YOL055C
YHR169W	YPL061W
YHR169W	YPL258C
YHR169W	YPR121W
YHR170W	YHR197W
YHR170W	YIR026C
YHR170W	YJL090C
YHR170W	YKL095W
YHR170W	YLR074C
YHR170W	YLR340W
YHR170W	YML064C
YHR170W	YMR080C
YHR170W	YNL061W
YHR170W	YOR080W
YHR170W	YOR351C
YHR171W	YKL134C
YHR171W	YMR183C
YHR171W	YNL212W
YHR171W	YNL255C
YHR171W	YNR007C
YHR171W	YOR191W
YHR172W	YJR091C
YHR172W	YLR045C
YHR172W	YLR212C
YHR172W	YNL126W
YHR172W	YPR086W
YHR174W	YIL091C
YHR174W	YLR196W
YHR174W	YLR265C
YHR174W	YNL006W
YHR174W	YNL127W
YHR174W	YOL135C
YHR175W	YLR288C
YHR177W	YLR447C
YHR178W	YJR007W
YHR178W	YLR147C
YHR178W	YMR020W
YHR178W	YNL252C
YHR178W	YOL004W
YHR178W	YOR171C

YHR178W	YOR351C
YHR178W	YOR377W
YHR178W	YPL076W
YHR179W	YJL090C
YHR179W	YKL103C
YHR179W	YLR074C
YHR179W	YLR186W
YHR179W	YLR314C
YHR179W	YLR383W
YHR179W	YLR427W
YHR179W	YMR059W
YHR179W	YMR106C
YHR179W	YNL094W
YHR179W	YNL244C
YHR179W	YOL128C
YHR179W	YPL150W
YHR179W	YPL204W
YHR179W	YPR110C
YHR180W	YLR288C
YHR180W	YNL175C
YHR182W	YJL020C
YHR182W	YLR433C
YHR183W	YHR196W
YHR183W	YIL148W
YHR183W	YKL085W
YHR183W	YLR175W
YHR183W	YLR309C
YHR183W	YMR049C
YHR183W	YMR059W
YHR183W	YNL014W
YHR183W	YNL244C
YHR183W	YOR212W
YHR183W	YPL126W
YHR183W	YPR110C
YHR184W	YLR185W
YHR184W	YML064C
YHR184W	YMR001C
YHR185C	YIL007C
YHR185C	YPL204W
YHR186C	YJL008C
YHR186C	YJL095W
YHR186C	YJL098W
YHR186C	YJL106W
YHR186C	YJR066W
YHR186C	YKL119C

YHR186C YKL171W
YHR186C YKL193C
YHR186C YLR096W
YHR186C YLR259C
YHR186C YNL006W
YHR186C YNL076W
YHR186C YNL118C
YHR186C YNL183C
YHR186C YPL180W
YHR187W YKL110C
YHR187W YLR384C
YHR187W YMR047C
YHR187W YMR312W
YHR187W YPL086C
YHR187W YPL101W
YHR188C YIL075C
YHR188C YIL088C
YHR188C YJL034W
YHR188C YJR015W
YHR188C YJR045C
YHR188C YLL028W
YHR188C YLR088W
YHR188C YLR372W
YHR188C YML085C
YHR188C YMR066W
YHR188C YNL092W
YHR188C YPL076W
YHR190W YIR038C
YHR190W YLR453C
YHR190W YMR153W
YHR190W YOR075W
YHR191C YJR068W
YHR191C YMR078C
YHR191C YNL290W
YHR191C YOL094C
YHR193C YJR091C
YHR193C YLR295C
YHR193C YMR049C
YHR193C YPL204W
YHR193C YPR111W
YHR194W YKR086W
YHR195W YIL048W
YHR195W YJL108C
YHR195W YKL120W
YHR195W YOL115W

YHR195W	YOR065W
YHR196W	YIL125W
YHR196W	YJL109C
YHR196W	YJR121W
YHR196W	YKL182W
YHR196W	YLL026W
YHR196W	YLR259C
YHR196W	YMR205C
YHR196W	YOL055C
YHR196W	YPL126W
YHR196W	YPR160W
YHR197W	YIR009W
YHR197W	YJL122W
YHR197W	YKL009W
YHR197W	YKR081C
YHR197W	YLR009W
YHR197W	YLR074C
YHR197W	YLR075W
YHR197W	YLR106C
YHR197W	YLR259C
YHR197W	YLR344W
YHR197W	YLR423C
YHR197W	YML085C
YHR197W	YMR142C
YHR197W	YNL002C
YHR197W	YNL110C
YHR197W	YNL113W
YHR197W	YNL182C
YHR197W	YNR053C
YHR197W	YOL127W
YHR197W	YOR063W
YHR197W	YPL061W
YHR197W	YPL093W
YHR197W	YPL131W
YHR197W	YPR016C

YHR198C YKR029C
YHR198C YML064C
YHR199C YJL034W
YHR199C YJL066C
YHR199C YJL138C
YHR199C YJR045C
YHR199C YLR427W
YHR199C YMR092C
YHR199C YMR308C
YHR199C YNL095C

YHR199C	YNL323W
YHR199C	YPR137W
YHR200W	YKL104C
YHR200W	YKL145W
YHR200W	YLR421C
YHR200W	YML092C
YHR200W	YMR314W
YHR200W	YOL038W
YHR200W	YOR259C
YHR200W	YOR261C
YHR200W	YPR103W
YHR200W	YPR108W
YHR201C	YPR056W
YHR203C	YNL034W
YHR204W	YJL053W
YHR204W	YLR321C
YHR204W	YMR304W
YHR205W	YOL133W
YHR206W	YLR433C
YHR206W	YNL027W
YHR206W	YPR165W
YHR207C	YNL027W
YHR207C	YPL258C
YHR208W	YJR148W
YHR209W	YMR047C
YHR209W	YOR319W
YHR211W	YJR091C
YHR211W	YOR360C
YHR212W-A	YJR086W
YHR212W-A	YMR182C
YHR214C-B	YKL129C
YHR214C-B	YMR032W
YHR214W-A	YKL166C
YHR215W	YLR088W
YHR215W	YLR453C
YHR216W	YLR347C
YHR216W	YLR427W
YHR216W	YNL175C
YHR216W	YNL189W
YIL001W	YOL133W
YIL002C	YLR399C
YIL002C	YML074C
YIL004C	YIL076W
YIL004C	YIL109C
YIL004C	YLR026C

YIL004C	YLR078C
YIL004C	YLR295C
YIL004C	YNL287W
YIL004C	YPL010W
YIL004C	YPL218W
YIL004C	YPR181C
YIL005W	YIL166C
YIL005W	YLR295C
YIL005W	YPR086W
YIL007C	YKL152C
YIL007C	YKR055W
YIL007C	YLR304C
YIL007C	YMR200W
YIL007C	YOR117W
YIL007C	YOR259C
YIL007C	YPR165W
YIL008W	YLR197W
YIL008W	YLR288C
YIL008W	YNL189W
YIL009C-A	YLR318W
YIL009C-A	YML092C
YIL009W	YLR310C
YIL009W	YOL130W
YIL009W	YPL141C
YIL010W	YLR288C
YIL011W	YMR201C
YIL013C	YLR083C
YIL014W	YOR231W
YIL016W	YJL196C
YIL016W	YJR010C-A
YIL016W	YKL008C
YIL016W	YKL212W
YIL016W	YLL023C
YIL016W	YLL028W
YIL016W	YLL061W
YIL016W	YLR018C
YIL016W	YML067C
YIL016W	YML075C
YIL016W	YML123C
YIL016W	YMR058W
YIL016W	YMR215W
YIL016W	YMR222C
YIL016W	YNL101W
YIL016W	YPL076W
YIL016W	YPL264C

YIL016W	YPL274W
YIL016W	YPR198W
YIL018W	YNL112W
YIL018W	YOR048C
YIL018W	YPL009C
YIL018W	YPL093W
YIL019W	YMR258C
YIL020C	YLR295C
YIL021W	YJL140W
YIL021W	YJL164C
YIL021W	YJR017C
YIL021W	YKR062W
YIL021W	YLR384C
YIL021W	YNL113W
YIL021W	YOL005C
YIL021W	YOR151C
YIL021W	YOR224C
YIL021W	YPL203W
YIL021W	YPR086W
YIL021W	YPR093C
YIL022W	YJR045C
YIL022W	YNR017W
YIL022W	YOR232W
YIL023C	YIR038C
YIL023C	YLL023C
YIL023C	YMR104C
YIL023C	YNR028W
YIL024C	YNL092W
YIL025C	YOR006C
YIL025C	YOR352W
YIL026C	YJL074C
YIL026C	YMR001C
YIL026C	YMR313C
YIL030C	YKL209C
YIL032C	YLR295C
YIL033C	YIL035C
YIL033C	YIL061C
YIL033C	YIL128W
YIL033C	YJL164C
YIL033C	YJR017C
YIL033C	YKL166C
YIL033C	YLR347C
YIL033C	YMR022W
YIL033C	YNL093W
YIL033C	YNL189W

YIL033C	YPL203W
YIL033C	YPR086W
YIL034C	YKL007W
YIL034C	YOR181W
YIL035C	YIL118W
YIL035C	YIL131C
YIL035C	YJL008C
YIL035C	YJL069C
YIL035C	YJL076W
YIL035C	YJL080C
YIL035C	YJL087C
YIL035C	YJR144W
YIL035C	YKL064W
YIL035C	YKL082C
YIL035C	YKL108W
YIL035C	YKL112W
YIL035C	YKR010C
YIL035C	YLR002C
YIL035C	YLR019W
YIL035C	YLR113W
YIL035C	YLR196W
YIL035C	YLR197W
YIL035C	YLR223C
YIL035C	YLR403W
YIL035C	YML069W
YIL035C	YML074C
YIL035C	YMR049C
YIL035C	YMR172W
YIL035C	YMR229C
YIL035C	YMR270C
YIL035C	YMR290C
YIL035C	YNL030W
YIL035C	YNL061W
YIL035C	YNL112W
YIL035C	YNL132W
YIL035C	YNL207W
YIL035C	YNL262W
YIL035C	YNL308C
YIL035C	YNL330C
YIL035C	YOL004W
YIL035C	YOL041C
YIL035C	YOL145C
YIL035C	YOR014W
YIL035C	YOR039W
YIL035C	YOR061W

YIL035C	YOR119C
YIL035C	YOR229W
YIL035C	YOR295W
YIL035C	YOR341W
YIL035C	YPL037C
YIL035C	YPL042C
YIL035C	YPL043W
YIL035C	YPL093W
YIL035C	YPL110C
YIL035C	YPL258C
YIL035C	YPR010C-A
YIL035C	YPR016C
YIL035C	YPR110C
YIL035C	YPR143W
YIL037C	YMR059W
YIL038C	YLR044C
YIL038C	YLR180W
YIL038C	YNL288W
YIL038C	YNR052C
YIL038C	YPL231W
YIL038C	YPR072W
YIL039W	YLR453C
YIL040W	YLL024C
YIL042C	YJL098W
YIL042C	YJL124C
YIL045W	YIL070C
YIL045W	YLR258W
YIL045W	YLR347C
YIL045W	YNL189W
YIL045W	YOR178C
YIL045W	YPR160W
YIL046W	YIL148W
YIL046W	YJL058C
YIL046W	YJL066C
YIL046W	YJL187C
YIL046W	YJR045C
YIL046W	YJR131W
YIL046W	YKL081W
YIL046W	YKL152C
YIL046W	YLR082C
YIL046W	YLR180W
YIL046W	YNL007C
YIL046W	YNL064C
YIL046W	YNL103W
YIL046W	YNL182C

YIL046W	YNL245C
YIL046W	YOL133W
YIL046W	YOR087W
YIL046W	YOR207C
YIL046W	YPL038W
YIL046W	YPL240C
YIL047C	YOL132W
YIL047C	YOR212W
YIL048W	YJL124C
YIL048W	YJL196C
YIL048W	YLR241W
YIL049W	YLR373C
YIL050W	YLR190W
YIL050W	YLR295C
YIL050W	YLR449W
YIL050W	YNL218W
YIL050W	YPL031C
YIL051C	YJL165C
YIL051C	YLR295C
YIL053W	YJL173C
YIL053W	YJR035W
YIL053W	YLR295C
YIL053W	YNR031C
YIL053W	YOR089C
YIL053W	YOR208W
YIL053W	YPL201C
YIL054W	YLR386W
YIL055C	YMR047C
YIL055C	YPL115C
YIL059C	YLR295C
YIL060W	YJR091C
YIL060W	YOR264W
YIL061C	YIL070C
YIL061C	YIL075C
YIL061C	YJL098W
YIL061C	YJR045C
YIL061C	YKL012W
YIL061C	YKR028W
YIL061C	YLR017W
YIL061C	YLR153C
YIL061C	YLR213C
YIL061C	YLR298C
YIL061C	YLR386W
YIL061C	YLR439W
YIL061C	YML016C

YIL061C	YML046W
YIL061C	YML074C
YIL061C	YMR009W
YIL061C	YMR044W
YIL061C	YMR080C
YIL061C	YMR125W
YIL061C	YMR290C
YIL061C	YMR319C
YIL061C	YNL064C
YIL061C	YNL189W
YIL061C	YNL199C
YIL061C	YNR016C
YIL061C	YOL004W
YIL061C	YOR275C
YIL061C	YOR346W
YIL061C	YPL016W
YIL061C	YPL178W
YIL061C	YPL215W
YIL061C	YPR191W
YIL062C	YJR065C
YIL062C	YKL013C
YIL062C	YLR370C
YIL062C	YNR035C
YIL063C	YJR091C
YIL063C	YLR293C
YIL063C	YOL055C
YIL063C	YOL061W
YIL065C	YJL112W
YIL065C	YJR091C
YIL065C	YKR036C
YIL065C	YLR292C
YIL065C	YLR321C
YIL066C	YJL066C
YIL066C	YKL011C
YIL066C	YKL035W
YIL066C	YLR163C
YIL066C	YLR259C
YIL066C	YML058W
YIL066C	YML085C
YIL066C	YMR214W
YIL066C	YNL064C
YIL066C	YNL085W
YIL066C	YNL134C
YIL066C	YPL235W
YIL066C	YPR086W

YIL068C	YJL085W
YIL068C	YLR166C
YIL068C	YPL022W
YIL068C	YPL179W
YIL068C	YPR055W
YIL070C	YJL115W
YIL070C	YJL128C
YIL070C	YJR035W
YIL070C	YKL108W
YIL070C	YLR074C
YIL070C	YMR036C
YIL070C	YNL250W
YIL070C	YOL054W
YIL070C	YOL108C
YIL070C	YOR090C
YIL070C	YOR326W
YIL070C	YPL141C
YIL070C	YPR015C
YIL071C	YOL117W
YIL071C	YOR361C
YIL072W	YLR263W
YIL074C	YLR053C
YIL074C	YLR056W
YIL074C	YMR047C
YIL074C	YNL311C
YIL075C	YJR017C
YIL075C	YKL078W
YIL075C	YKL166C
YIL075C	YLR421C
YIL075C	YMR001C
YIL075C	YMR276W
YIL075C	YNL290W
YIL075C	YOL094C
YIL075C	YOL133W
YIL075C	YOR181W
YIL075C	YPR108W
YIL075C	YPR111W
YIL076W	YJR091C
YIL076W	YLR078C
YIL076W	YLR268W
YIL076W	YLR295C
YIL076W	YNL161W
YIL077C	YOL135C
YIL078W	YKL198C
YIL078W	YMR059W

YIL078W	YNL135C
YIL079C	YLR259C
YIL079C	YML056C
YIL079C	YMR216C
YIL079C	YNL004W
YIL079C	YOL115W
YIL079C	YOR204W
YIL082W-A	YLR310C
YIL084C	YMR128W
YIL084C	YNL330C
YIL084C	YOL004W
YIL085C	YLR325C
YIL086C	YKL007W
YIL092W	YMR109W
YIL092W	YNR028W
YIL092W	YNR040W
YIL093C	YLR074C
YIL094C	YKR036C
YIL094C	YKR048C
YIL094C	YLR103C
YIL094C	YML126C
YIL094C	YMR106C
YIL094C	YMR308C
YIL094C	YNL313C
YIL094C	YOL139C
YIL094C	YOL145C
YIL094C	YOR073W
YIL094C	YPL031C
YIL094C	YPL140C
YIL095W	YIR006C
YIL095W	YJL088W
YIL095W	YJR045C
YIL095W	YLR191W
YIL095W	YLR196W
YIL095W	YLR259C
YIL095W	YLR337C
YIL095W	YLR342W
YIL095W	YNL084C
YIL095W	YNL243W
YIL095W	YOL055C
YIL095W	YPL258C
YIL096C	YJR091C
YIL097W	YOR117W
YIL097W	YPL227C
YIL098C	YPR180W

YIL103W	YKL191W
YIL104C	YLR175W
YIL104C	YMR296C
YIL104C	YNL124W
YIL104C	YNL326C
YIL105C	YJL095W
YIL105C	YKL130C
YIL105C	YMR068W
YIL105C	YNL047C
YIL105C	YNL161W
YIL105C	YNL207W
YIL105C	YNL307C
YIL105C	YNL308C
YIL105C	YOR014W
YIL105C	YPL059W
YIL106W	YIL169C
YIL106W	YJL066C
YIL106W	YKL080W
YIL106W	YKL126W
YIL106W	YLR180W
YIL106W	YMR276W
YIL106W	YNL161W
YIL106W	YOL123W
YIL106W	YOR046C
YIL106W	YPL085W
YIL106W	YPR111W
YIL106W	YPR191W
YIL108W	YOL126C
YIL108W	YOR032C
YIL108W	YPR154W
YIL109C	YKL171W
YIL109C	YLR026C
YIL109C	YLR078C
YIL109C	YML012W
YIL109C	YPL085W
YIL109C	YPL187W
YIL109C	YPR181C
YIL110W	YLR295C
YIL111W	YLR295C
YIL112W	YKR029C
YIL112W	YMR273C
YIL112W	YNL147W
YIL112W	YOL068C
YIL113W	YLL019C
YIL113W	YNL138W

YIL113W	YPL258C
YIL114C	YMR215W
YIL114C	YNL130C
YIL114C	YPL274W
YIL115C	YJL041W
YIL115C	YJL061W
YIL115C	YJR132W
YIL115C	YMR047C
YIL115C	YOR046C
YIL115C	YOR160W
YIL115C	YPL204W
YIL115C	YPR119W
YIL117C	YKL092C
YIL117C	YLR295C
YIL117C	YNL307C
YIL117C	YOL116W
YIL118W	YLR114C
YIL118W	YOR061W
YIL119C	YPR174C
YIL120W	YLL023C
YIL122W	YKL161C
YIL124W	YLR295C
YIL124W	YLR447C
YIL125W	YKL198C
YIL125W	YML064C
YIL125W	YMR235C
YIL125W	YOR005C
YIL125W	YPL213W
YIL126W	YLR113W
YIL126W	YLR176C
YIL126W	YLR321C
YIL126W	YNL116W
YIL128W	YJL008C
YIL128W	YLR291C
YIL128W	YMR112C
YIL128W	YMR117C
YIL128W	YMR214W
YIL128W	YNL173C
YIL128W	YOL126C
YIL128W	YPL061W
YIL128W	YPL064C
YIL128W	YPL191C
YIL128W	YPR110C
YIL129C	YNL161W
YIL130W	YLR116W

YIL130W	YLR337C
YIL131C	YIR002C
YIL131C	YJL076W
YIL131C	YJL197W
YIL131C	YJR007W
YIL131C	YJR144W
YIL131C	YKR026C
YIL131C	YLR291C
YIL131C	YMR144W
YIL131C	YNL030W
YIL131C	YNL042W
YIL131C	YNL071W
YIL131C	YNL229C
YIL131C	YNL272C
YIL131C	YOL004W
YIL131C	YOR039W
YIL131C	YOR061W
YIL131C	YPL235W
YIL131C	YPL237W
YIL132C	YLR321C
YIL132C	YLR322W
YIL132C	YLR376C
YIL132C	YML092C
YIL132C	YNL189W
YIL137C	YJR091C
YIL137C	YOR272W
YIL139C	YLR147C
YIL140W	YJL129C
YIL142W	YJL052W
YIL142W	YJL106W
YIL142W	YJR045C
YIL142W	YJR070C
YIL142W	YKL060C
YIL142W	YKL198C
YIL142W	YKR036C
YIL142W	YLL011W
YIL142W	YLR105C
YIL142W	YLR196W
YIL142W	YLR355C
YIL142W	YMR054W
YIL142W	YNL037C
YIL142W	YNL317W
YIL142W	YNR032W
YIL142W	YOR136W
YIL142W	YOR212W

YIL142W	YOR230W
YIL143C	YIR009W
YIL143C	YLR191W
YIL143C	YLR259C
YIL143C	YLR295C
YIL143C	YNL085W
YIL143C	YOL090W
YIL143C	YOR027W
YIL143C	YPL122C
YIL143C	YPR056W
YIL144W	YJL020C
YIL144W	YJR112W
YIL144W	YLL021W
YIL144W	YLR191W
YIL144W	YLR456W
YIL144W	YMR117C
YIL144W	YNL041C
YIL144W	YNR035C
YIL144W	YOL069W
YIL144W	YPL174C
YIL144W	YPL260W
YIL144W	YPR105C
YIL144W	YPR163C
YIL145C	YLR447C
YIL145C	YOR142W-A
YIL147C	YJR077C
YIL147C	YMR108W
YIL148W	YJL172W
YIL148W	YJR052W
YIL148W	YJR090C
YIL148W	YKL210W
YIL148W	YLR079W
YIL148W	YLR097C
YIL148W	YMR022W
YIL148W	YMR199W
YIL148W	YMR276W
YIL148W	YMR297W
YIL148W	YNL103W
YIL148W	YNL311C
YIL148W	YNL327W
YIL148W	YNR006W
YIL148W	YOL087C
YIL148W	YOL133W
YIL148W	YPL022W
YIL148W	YPL065W

YIL148W	YPR119W
YIL148W	YPR154W
YIL149C	YLR347C
YIL149C	YNL128W
YIL149C	YNL135C
YIL150C	YLR288C
YIL150C	YPL161C
YIL150C	YPR019W
YIL151C	YKL171W
YIL151C	YLR121C
YIL152W	YOR057W
YIL153W	YOL044W
YIL154C	YML016C
YIL156W	YKL129C
YIL156W	YLR191W
YIL156W	YMR032W
YIL156W	YMR109W
YIL156W	YPR154W
YIL157C	YLR295C
YIL158W	YIR002C
YIL159W	YJL020C
YIL159W	YKL079W
YIL159W	YKL129C
YIL159W	YKR055W
YIL159W	YLR191W
YIL159W	YLR310C
YIL159W	YLR319C
YIL159W	YLR452C
YIL159W	YMR032W
YIL159W	YMR109W
YIL159W	YOR122C
YIL162W	YLR078C
YIL162W	YLR378C
YIL163C	YPL161C
YIL166C	YKL217W
YIL169C	YNR074C
YIL173W	YJR022W
YIL173W	YLR310C
YIR001C	YMR012W
YIR001C	YNL016W
YIR001C	YNL138W
YIR002C	YLR383W
YIR002C	YOL034W
YIR003W	YJL020C
YIR003W	YJR091C

YIR003W	YKL007W
YIR003W	YKL129C
YIR003W	YLR074C
YIR003W	YLR191W
YIR003W	YMR109W
YIR005W	YJL034W
YIR005W	YJL130C
YIR005W	YJR045C
YIR005W	YLR259C
YIR005W	YLR438W
YIR005W	YMR057C
YIR005W	YMR105C
YIR005W	YNR001C
YIR006C	YNL020C
YIR006C	YNL084C
YIR008C	YLR453C
YIR008C	YNL102W
YIR008C	YNL262W
YIR008C	YNR052C
YIR009W	YJL092W
YIR009W	YKL173W
YIR009W	YKR099W
YIR009W	YLR067C
YIR009W	YLR288C
YIR009W	YLR433C
YIR009W	YLR456W
YIR009W	YNL036W
YIR009W	YNL091W
YIR009W	YNL165W
YIR009W	YOR011W
YIR009W	YOR017W
YIR009W	YOR264W
YIR009W	YPL016W
YIR009W	YPL213W
YIR009W	YPR182W
YIR010W	YLR202C
YIR011C	YNL189W
YIR012W	YJR004C
YIR012W	YLR075W
YIR012W	YOR181W
YIR013C	YJL102W
YIR014W	YNL118C
YIR016W	YNL161W
YIR017C	YJR060W
YIR017C	YJR112W

YIR017C	YLR423C
YIR017C	YLR437C
YIR017C	YNL016W
YIR017C	YNL103W
YIR022W	YJR010C-A
YIR022W	YLR343W
YIR022W	YLR372W
YIR022W	YOL132W
YIR022W	YPR163C
YIR023W	YJR066W
YIR023W	YNL314W
YIR024C	YNL118C
YIR025W	YJR091C
YIR025W	YMR308C
YIR026C	YJL020C
YIR026C	YJL083W
YIR026C	YKL023W
YIR026C	YMR049C
YIR026C	YMR229C
YIR026C	YOL063C
YIR026C	YPR029C
YIR026C	YPR115W
YIR026C	YPR164W
YIR027C	YNL252C
YIR028W	YJL183W
YIR033W	YKL081W
YIR034C	YKL152C
YIR034C	YLR354C
YIR034C	YNL055C
YIR035C	YLR447C
YIR035C	YNL232W
YIR036C	YLR295C
YIR037W	YLR216C
YIR037W	YMR047C
YIR038C	YJL097W
YIR038C	YLR026C
YIR038C	YLR088W
YIR038C	YML048W
YIR038C	YMR119W
YIR038C	YNL234W
YIR038C	YOL065C
YIR038C	YPL020C
YIR040C	YLR295C
YIR040C	YNL007C
YIR042C	YPL240C

YJL001W	YLR295C
YJL001W	YLR386W
YJL001W	YOR157C
YJL001W	YPR054W
YJL001W	YPR103W
YJL001W	YPR180W
YJL002C	YJR015W
YJL002C	YLR208W
YJL002C	YML019W
YJL002C	YML130C
YJL002C	YMR146C
YJL002C	YMR149W
YJL002C	YOR085W
YJL002C	YOR103C
YJL002C	YPL076W
YJL004C	YJR022W
YJL004C	YKR088C
YJL004C	YLR083C
YJL004C	YLR241W
YJL004C	YML038C
YJL004C	YNL044W
YJL004C	YNL121C
YJL004C	YPL264C
YJL005W	YJL026W
YJL005W	YJL187C
YJL005W	YLL050C
YJL005W	YLR310C
YJL005W	YMR059W
YJL005W	YNL098C
YJL005W	YNL138W
YJL005W	YOL139C
YJL006C	YKL139W
YJL006C	YML112W
YJL008C	YJL074C
YJL008C	YJL106W
YJL008C	YJL157C
YJL008C	YJR053W
YJL008C	YJR068W
YJL008C	YKL095W
YJL008C	YKR036C
YJL008C	YLL011W
YJL008C	YLR097C
YJL008C	YLR196W
YJL008C	YLR222C
YJL008C	YLR340W

YJL008C	YML064C
YJL008C	YMR049C
YJL008C	YMR106C
YJL008C	YMR205C
YJL008C	YNL106C
YJL008C	YNL290W
YJL008C	YNL312W
YJL008C	YNL317W
YJL008C	YNL323W
YJL008C	YOL045W
YJL008C	YOL094C
YJL008C	YOL115W
YJL008C	YOL133W
YJL008C	YOR080W
YJL008C	YOR117W
YJL008C	YOR212W
YJL008C	YOR230W
YJL008C	YPL151C
YJL008C	YPR018W
YJL008C	YPR054W
YJL008C	YPR093C
YJL008C	YPR110C
YJL008C	YPR111W
YJL010C	YLL019C
YJL011C	YKL144C
YJL011C	YNR003C
YJL011C	YOR116C
YJL011C	YOR210W
YJL011C	YOR224C
YJL011C	YPR110C
YJL012C	YJR143C
YJL012C	YJR152W
YJL012C	YML048W
YJL012C	YPR154W
YJL013C	YNL236W
YJL013C	YOR026W
YJL014W	YKL198C
YJL014W	YKR036C
YJL014W	YLL011W
YJL014W	YLR196W
YJL014W	YMR106C
YJL014W	YOR212W
YJL014W	YOR230W
YJL015C	YLR453C
YJL015C	YOR136W

YJL019W	YLR233C
YJL019W	YMR001C
YJL019W	YOL012C
YJL019W	YOL104C
YJL020C	YJL088W
YJL020C	YJL106W
YJL020C	YJL118W
YJL020C	YJL128C
YJL020C	YJL194W
YJL020C	YJL195C
YJL020C	YJR022W
YJL020C	YJR023C
YJL020C	YJR047C
YJL020C	YJR115W
YJL020C	YJR149W
YJL020C	YKL109W
YJL020C	YKL129C
YJL020C	YLR144C
YJL020C	YLR191W
YJL020C	YLR206W
YJL020C	YLR258W
YJL020C	YLR337C
YJL020C	YML061C
YJL020C	YMR032W
YJL020C	YMR109W
YJL020C	YMR162C
YJL020C	YMR184W
YJL020C	YMR221C
YJL020C	YNL023C
YJL020C	YNL025C
YJL020C	YNL053W
YJL020C	YNL094W
YJL020C	YNL199C
YJL020C	YNL206C
YJL020C	YNR016C
YJL020C	YNR064C
YJL020C	YOL055C
YJL020C	YOL133W
YJL020C	YOR047C
YJL020C	YOR081C
YJL020C	YOR156C
YJL020C	YOR181W
YJL020C	YOR290C
YJL020C	YPL202C
YJL020C	YPL245W

YJL020C	YPL269W
YJL020C	YPR072W
YJL020C	YPR091C
YJL020C	YPR104C
YJL020C	YPR171W
YJL020C	YPR184W
YJL023C	YLR449W
YJL024C	YPL195W
YJL025W	YMR270C
YJL026W	YJL115W
YJL026W	YLR442C
YJL026W	YML064C
YJL026W	YML095C
YJL026W	YMR059W
YJL026W	YOR229W
YJL026W	YPL227C
YJL026W	YPR054W
YJL029C	YKR002W
YJL029C	YKR020W
YJL029C	YLR262C
YJL030W	YNL218W
YJL030W	YNL236W
YJL031C	YOR370C
YJL031C	YPR176C
YJL033W	YJR093C
YJL033W	YKR002W
YJL033W	YLR115W
YJL033W	YLR175W
YJL033W	YLR197W
YJL033W	YLR277C
YJL033W	YMR061W
YJL033W	YMR290C
YJL033W	YNL016W
YJL033W	YNL317W
YJL033W	YOL041C
YJL033W	YOR310C
YJL034W	YJL098W
YJL034W	YJL128C
YJL034W	YJL173C
YJL034W	YJR035W
YJL034W	YJR091C
YJL034W	YLL011W
YJL034W	YLR229C
YJL034W	YLR288C
YJL034W	YLR291C

YJL034W	YLR383W
YJL034W	YLR403W
YJL034W	YML016C
YJL034W	YML058W
YJL034W	YML064C
YJL034W	YML115C
YJL034W	YMR049C
YJL034W	YMR117C
YJL034W	YMR201C
YJL034W	YMR214W
YJL034W	YMR297W
YJL034W	YNL012W
YJL034W	YNL244C
YJL034W	YNL250W
YJL034W	YNR031C
YJL034W	YOL054W
YJL034W	YOL062C
YJL034W	YOL094C
YJL034W	YOL115W
YJL034W	YOL126C
YJL034W	YOL139C
YJL034W	YOR254C
YJL034W	YOR351C
YJL034W	YPL149W
YJL034W	YPL151C
YJL034W	YPL153C
YJL034W	YPL187W
YJL034W	YPL194W
YJL034W	YPL259C
YJL034W	YPR110C
YJL035C	YLR316C
YJL036W	YLR423C
YJL039C	YLR447C
YJL039C	YMR032W
YJL039C	YMR153W
YJL041W	YJL061W
YJL041W	YJL063C
YJL041W	YKL205W
YJL041W	YLR293C
YJL041W	YLR312W-A
YJL041W	YLR423C
YJL041W	YML025C
YJL041W	YMR024W
YJL041W	YMR047C
YJL041W	YMR193W

YJL041W	YMR308C
YJL041W	YOL132W
YJL041W	YPL204W
YJL041W	YPR119W
YJL044C	YLR295C
YJL044C	YMR218C
YJL044C	YNL071W
YJL044C	YPL207W
YJL045W	YNR028W
YJL047C	YJR073C
YJL047C	YJR091C
YJL047C	YLR320W
YJL047C	YOL063C
YJL047C	YOL133W
YJL047C	YPR164W
YJL048C	YKL130C
YJL048C	YLR368W
YJL048C	YOR047C
YJL048C	YPR120C
YJL050W	YMR304W
YJL050W	YOL115W
YJL050W	YPL190C
YJL052W	YJL098W
YJL052W	YJR007W
YJL052W	YKR026C
YJL052W	YKR055W
YJL052W	YMR049C
YJL052W	YMR117C
YJL052W	YNL180C
YJL052W	YNL189W
YJL052W	YNL244C
YJL052W	YNR031C
YJL052W	YOL062C
YJL052W	YOL139C
YJL052W	YOR089C
YJL052W	YOR212W
YJL052W	YOR230W
YJL052W	YOR269W
YJL052W	YPL031C
YJL052W	YPL140C
YJL052W	YPL149W
YJL052W	YPL259C
YJL052W	YPL262W
YJL052W	YPR110C
YJL052W	YPR137W

YJL053W	YJL154C
YJL053W	YJL187C
YJL053W	YLR453C
YJL053W	YOR069W
YJL053W	YOR132W
YJL054W	YOR297C
YJL055W	YOR264W
YJL056C	YPR154W
YJL057C	YLR452C
YJL057C	YML117W
YJL057C	YMR129W
YJL057C	YMR153W
YJL057C	YMR276W
YJL057C	YNL007C
YJL057C	YNL112W
YJL057C	YOL123W
YJL057C	YOR028C
YJL058C	YKL203C
YJL058C	YLR295C
YJL058C	YLR373C
YJL058C	YLR447C
YJL058C	YML064C
YJL058C	YML098W
YJL058C	YNL006W
YJL058C	YNL092W
YJL058C	YNL189W
YJL058C	YNL201C
YJL059W	YLR288C
YJL060W	YLR314C
YJL061W	YKL061W
YJL061W	YLR178C
YJL061W	YLR423C
YJL061W	YMR047C
YJL061W	YOR098C
YJL061W	YPL204W
YJL061W	YPR119W
YJL062W	YKL146W
YJL062W	YPL076W
YJL064W	YJL143W
YJL064W	YNR029C
YJL065C	YNR029C
YJL066C	YJL157C
YJL066C	YJR068W
YJL066C	YKL193C
YJL066C	YKR026C

YJL066C	YKR036C
YJL066C	YLR097C
YJL066C	YMR106C
YJL066C	YNL006W
YJL066C	YNL128W
YJL066C	YNL250W
YJL066C	YOR125C
YJL066C	YPL140C
YJL066C	YPL256C
YJL066C	YPR086W
YJL066C	YPR110C
YJL066C	YPR111W
YJL066C	YPR154W
YJL067W	YLR295C
YJL067W	YPL020C
YJL068C	YKL152C
YJL068C	YLR354C
YJL068C	YOR125C
YJL069C	YJL109C
YJL069C	YJR045C
YJL069C	YKL012W
YJL069C	YKL099C
YJL069C	YKR060W
YJL069C	YKR096W
YJL069C	YLR129W
YJL069C	YLR197W
YJL069C	YLR222C
YJL069C	YLR409C
YJL069C	YML093W
YJL069C	YMR093W
YJL069C	YMR229C
YJL069C	YNL132W
YJL069C	YOL055C
YJL069C	YOR039W
YJL069C	YOR059C
YJL069C	YOR061W
YJL069C	YOR090C
YJL069C	YOR145C
YJL069C	YPL126W
YJL072C	YPR086W
YJL074C	YLR086W
YJL074C	YMR001C
YJL074C	YMR065W
YJL074C	YMR124W
YJL074C	YMR192W

YJL074C	YNL030W
YJL074C	YOL041C
YJL074C	YOL069W
YJL074C	YPL153C
YJL074C	YPL235W
YJL075C	YNL157W
YJL076W	YJL128C
YJL076W	YJR057W
YJL076W	YKL001C
YJL076W	YKL193C
YJL076W	YKR010C
YJL076W	YLR019W
YJL076W	YLR096W
YJL076W	YML102W
YJL076W	YNL307C
YJL076W	YOR061W
YJL076W	YOR119C
YJL076W	YOR341W
YJL076W	YPL126W
YJL076W	YPR010C
YJL076W	YPR010C-A
YJL078C	YKL157W
YJL078C	YLR295C
YJL078C	YPL266W
YJL079C	YPR086W
YJL080C	YMR059W
YJL080C	YMR116C
YJL080C	YMR125W
YJL080C	YPL042C
YJL081C	YJL098W
YJL081C	YNL030W
YJL081C	YNL107W
YJL081C	YOL012C
YJL081C	YOR119C
YJL081C	YOR244W
YJL081C	YPL235W
YJL082W	YLR014C
YJL083W	YJR066W
YJL083W	YPL109C
YJL084C	YLR288C
YJL084C	YMR032W
YJL084C	YOR348C
YJL085W	YLR247C
YJL087C	YKL108W
YJL087C	YLR447C

YJL088W	YJL092W
YJL088W	YJL128C
YJL088W	YJR052W
YJL088W	YJR059W
YJL088W	YJR068W
YJL088W	YKL103C
YJL088W	YKL108W
YJL088W	YLR196W
YJL088W	YLR208W
YJL088W	YLR238W
YJL088W	YLR247C
YJL088W	YLR288C
YJL088W	YLR323C
YJL088W	YLR383W
YJL088W	YML058W
YJL088W	YML068W
YJL088W	YMR106C
YJL088W	YMR137C
YJL088W	YMR167W
YJL088W	YMR201C
YJL088W	YNL023C
YJL088W	YNL088W
YJL088W	YNL094W
YJL088W	YNL250W
YJL088W	YNL311C
YJL088W	YOL054W
YJL088W	YOR026W
YJL088W	YOR231W
YJL088W	YPL022W
YJL088W	YPL194W
YJL088W	YPR104C
YJL088W	YPR110C
YJL089W	YMR280C
YJL089W	YPL042C
YJL090C	YKL108W
YJL090C	YLR259C
YJL090C	YNL064C
YJL090C	YNL189W
YJL091C	YMR047C
YJL091C	YMR215W
YJL091C	YPL076W
YJL092W	YKL152C
YJL092W	YLR259C
YJL092W	YLR265C
YJL092W	YOR355W

YJL092W	YPR181C
YJL093C	YNL121C
YJL094C	YLR193C
YJL095W	YJL209W
YJL095W	YKL129C
YJL095W	YKL161C
YJL095W	YKL172W
YJL095W	YKL203C
YJL095W	YKR024C
YJL095W	YKR048C
YJL095W	YKR081C
YJL095W	YLL008W
YJL095W	YLR067C
YJL095W	YLR196W
YJL095W	YLR305C
YJL095W	YML074C
YJL095W	YMR064W
YJL095W	YMR109W
YJL095W	YMR229C
YJL095W	YMR290C
YJL095W	YMR304W
YJL095W	YNL061W
YJL095W	YNL175C
YJL095W	YNL271C
YJL095W	YOL041C
YJL095W	YOR017W
YJL095W	YOR061W
YJL095W	YOR201C
YJL095W	YOR231W
YJL095W	YOR294W
YJL095W	YPL012W
YJL095W	YPL043W
YJL095W	YPL084W
YJL095W	YPL093W
YJL095W	YPL140C
YJL096W	YKL191W
YJL096W	YOL075C
YJL098W	YJR009C
YJL098W	YJR045C
YJL098W	YJR066W
YJL098W	YJR077C
YJL098W	YJR121W
YJL098W	YKL035W
YJL098W	YML085C
YJL098W	YMR005W

YJL098W	YMR209C
YJL098W	YNL006W
YJL098W	YNL025C
YJL098W	YNL055C
YJL098W	YNL076W
YJL098W	YNL107W
YJL098W	YOL139C
YJL098W	YOR061W
YJL098W	YOR187W
YJL098W	YOR244W
YJL098W	YPL129W
YJL098W	YPL180W
YJL098W	YPL204W
YJL098W	YPR040W
YJL098W	YPR054W
YJL098W	YPR111W
YJL099W	YLR424W
YJL099W	YPL242C
YJL100W	YLR295C
YJL100W	YOR181W
YJL100W	YPL040C
YJL102W	YOL133W
YJL106W	YJR045C
YJL106W	YJR091C
YJL106W	YJR094C
YJL106W	YJR121W
YJL106W	YKL140W
YJL106W	YLR180W
YJL106W	YLR259C
YJL106W	YLR310C
YJL106W	YLR447C
YJL106W	YML085C
YJL106W	YML124C
YJL106W	YMR001C
YJL106W	YMR100W
YJL106W	YNL006W
YJL106W	YNL007C
YJL106W	YNL064C
YJL106W	YOL055C
YJL106W	YPL235W
YJL106W	YPL267W
YJL106W	YPR115W
YJL107C	YJL138C
YJL107C	YJR091C
YJL108C	YPL076W

YJL109C	YLR233C
YJL109C	YMR093W
YJL109C	YOR080W
YJL109C	YPL043W
YJL110C	YJR066W
YJL110C	YJR091C
YJL110C	YKR034W
YJL110C	YNL021W
YJL111W	YKR036C
YJL111W	YLL011W
YJL111W	YLR196W
YJL111W	YML064C
YJL111W	YMR028W
YJL111W	YNL006W
YJL111W	YNL061W
YJL111W	YNL317W
YJL111W	YOR212W
YJL111W	YOR230W
YJL112W	YLL001W
YJL115W	YJR140C
YJL115W	YMR127C
YJL115W	YMR200W
YJL115W	YOR213C
YJL115W	YPL153C
YJL117W	YJR010C-A
YJL117W	YKL008C
YJL117W	YKL065C
YJL117W	YLR018C
YJL117W	YLR372W
YJL117W	YLR423C
YJL117W	YML123C
YJL117W	YMR058W
YJL117W	YMR106C
YJL117W	YMR153W
YJL117W	YMR215W
YJL117W	YNL101W
YJL117W	YOL065C
YJL117W	YOR099W
YJL117W	YPL076W
YJL117W	YPL264C
YJL122W	YLR074C
YJL122W	YPR017C
YJL123C	YOL128C
YJL124C	YJR022W
YJL124C	YJR143C

YJL124C	YKL173W
YJL124C	YKR026C
YJL124C	YLR003C
YJL124C	YLR362W
YJL124C	YLR438C-A
YJL124C	YML088W
YJL124C	YMR056C
YJL124C	YMR080C
YJL124C	YMR250W
YJL124C	YNL032W
YJL124C	YNL118C
YJL124C	YNL147W
YJL124C	YNL163C
YJL124C	YNL276C
YJL124C	YOL149W
YJL124C	YOR109W
YJL124C	YOR147W
YJL124C	YOR320C
YJL124C	YOR375C
YJL124C	YPL016W
YJL124C	YPL084W
YJL124C	YPL152W
YJL128C	YJR045C
YJL128C	YJR121W
YJL128C	YLR006C
YJL128C	YLR044C
YJL128C	YLR113W
YJL128C	YLR191W
YJL128C	YLR259C
YJL128C	YLR310C
YJL128C	YLR362W
YJL128C	YMR032W
YJL128C	YMR066W
YJL128C	YMR109W
YJL128C	YMR319C
YJL128C	YNL053W
YJL128C	YNL110C
YJL128C	YNR031C
YJL128C	YNR058W
YJL128C	YOR201C
YJL128C	YOR375C
YJL130C	YJL141C
YJL130C	YKL166C
YJL130C	YLR148W
YJL130C	YLR216C

YJL130C	YLR229C
YJL130C	YLR263W
YJL130C	YLR340W
YJL130C	YML032C
YJL130C	YML057W
YJL130C	YML064C
YJL130C	YMR022W
YJL130C	YMR106C
YJL130C	YMR137C
YJL130C	YMR205C
YJL130C	YNL161W
YJL130C	YNL290W
YJL130C	YOL087C
YJL130C	YOL115W
YJL130C	YOL133W
YJL130C	YOR181W
YJL130C	YOR351C
YJL130C	YPR110C
YJL137C	YLR258W
YJL137C	YMR212C
YJL138C	YJR007W
YJL138C	YJR035W
YJL138C	YKL060C
YJL138C	YKL095W
YJL138C	YLR044C
YJL138C	YLR259C
YJL138C	YLR309C
YJL138C	YLR453C
YJL138C	YML064C
YJL138C	YML095C
YJL138C	YMR059W
YJL138C	YMR205C
YJL138C	YNL106C
YJL138C	YNL132W
YJL138C	YNL244C
YJL138C	YOL090W
YJL138C	YOL135C
YJL138C	YOL139C
YJL138C	YOR212W
YJL138C	YOR351C
YJL140W	YJL164C
YJL140W	YJR091C
YJL140W	YLR295C
YJL140W	YLR384C
YJL140W	YOR151C

YJL140W	YPL203W
YJL141C	YJR045C
YJL141C	YKL182W
YJL141C	YKR001C
YJL141C	YLL001W
YJL141C	YLR109W
YJL141C	YLR180W
YJL141C	YLR241W
YJL141C	YLR256W-A
YJL141C	YLR259C
YJL141C	YLR270W
YJL141C	YLR342W
YJL141C	YMR295C
YJL141C	YMR304W
YJL141C	YNL064C
YJL141C	YNL230C
YJL141C	YNL250W
YJL141C	YNR052C
YJL141C	YOL055C
YJL141C	YOL133W
YJL141C	YOR080W
YJL141C	YOR173W
YJL141C	YOR197W
YJL141C	YPL203W
YJL141C	YPL247C
YJL141C	YPL258C
YJL141C	YPR184W
YJL143W	YJR045C
YJL143W	YMR203W
YJL143W	YNL131W
YJL143W	YNR017W
YJL151C	YOR181W
YJL152W	YOR281C
YJL153C	YJL190C
YJL153C	YLR310C
YJL153C	YOR212W
YJL153C	YOR351C
YJL154C	YJL187C
YJL154C	YJR110W
YJL154C	YKL171W
YJL154C	YOR069W
YJL154C	YOR132W
YJL154C	YOR272W
YJL157C	YJR045C
YJL157C	YJR121W

YJL157C	YLR229C
YJL157C	YLR259C
YJL157C	YMR012W
YJL157C	YMR199W
YJL157C	YMR214W
YJL157C	YMR304W
YJL157C	YNL189W
YJL157C	YNR031C
YJL157C	YOR151C
YJL157C	YOR212W
YJL157C	YPL022W
YJL157C	YPL256C
YJL157C	YPR120C
YJL158C	YJR030C
YJL158C	YLR226W
YJL159W	YNL321W
YJL162C	YPL059W
YJL163C	YLL061W
YJL164C	YJR045C
YJL164C	YKL166C
YJL164C	YLL040C
YJL164C	YLR259C
YJL164C	YML010W
YJL164C	YMR196W
YJL164C	YMR319C
YJL164C	YNL093W
YJL164C	YOL005C
YJL164C	YOR151C
YJL164C	YOR210W
YJL164C	YOR375C
YJL164C	YPL203W
YJL165C	YOL103W
YJL166W	YLR295C
YJL167W	YJR035W
YJL167W	YNL244C
YJL167W	YPL149W
YJL168C	YPL073C
YJL172W	YNL217W
YJL173C	YJR121W
YJL173C	YJR141W
YJL173C	YKL035W
YJL173C	YKL152C
YJL173C	YLR180W
YJL173C	YLR259C
YJL173C	YML032C

YJL173C	YMR105C
YJL173C	YMR234W
YJL173C	YNL014W
YJL173C	YNR001C
YJL173C	YOL055C
YJL173C	YPL061W
YJL173C	YPL240C
YJL173C	YPL258C
YJL174W	YOR194C
YJL176C	YLR453C
YJL176C	YNL207W
YJL176C	YOR038C
YJL176C	YOR119C
YJL176C	YOR290C
YJL176C	YPL016W
YJL179W	YLR447C
YJL179W	YOR380W
YJL180C	YLR295C
YJL181W	YPL031C
YJL183W	YJR075W
YJL184W	YKR038C
YJL184W	YML036W
YJL184W	YMR052W
YJL184W	YOR117W
YJL184W	YPL174C
YJL185C	YMR204C
YJL185C	YOR127W
YJL187C	YKL042W
YJL187C	YLR067C
YJL187C	YLR096W
YJL187C	YMR001C
YJL187C	YMR304W
YJL187C	YNL094W
YJL187C	YOR023C
YJL187C	YOR061W
YJL187C	YOR257W
YJL187C	YOR326W
YJL187C	YPL124W
YJL187C	YPR119W
YJL189W	YML109W
YJL191W	YNL041C
YJL192C	YJR091C
YJL194W	YKL145W
YJL194W	YLL004W
YJL194W	YLR191W

YJL194W	YML065W
YJL194W	YNL261W
YJL194W	YPR162C
YJL196C	YJR010C-A
YJL196C	YJR117W
YJL196C	YKL004W
YJL196C	YKL065C
YJL196C	YKL154W
YJL196C	YLR372W
YJL196C	YMR058W
YJL196C	YMR215W
YJL196C	YNL044W
YJL196C	YNL101W
YJL196C	YPL076W
YJL196C	YPL227C
YJL196C	YPR028W
YJL198W	YPR154W
YJL199C	YJR091C
YJL199C	YLR198C
YJL199C	YLR288C
YJL199C	YLR291C
YJL199C	YLR447C
YJL199C	YML064C
YJL199C	YML092C
YJL199C	YNL189W
YJL199C	YPL070W
YJL200C	YOL133W
YJL203W	YKR090W
YJL203W	YLR067C
YJL203W	YMR240C
YJL203W	YMR285C
YJL203W	YNR053C
YJL203W	YOL091W
YJL203W	YOL136C
YJL208C	YKL222C
YJL208C	YKR079C
YJL210W	YLR083C
YJL210W	YLR288C
YJL214W	YLR093C
YJL214W	YLR372W
YJL214W	YML048W
YJL214W	YMR243C
YJL214W	YPR198W
YJL215C	YKR026C
YJL215C	YOR264W

YJL218W	YML064C
YJL218W	YNL189W
YJL218W	YPL070W
YJL219W	YJR161C
YJL219W	YLR447C
YJL219W	YML048W
YJL222W	YKL065C
YJL222W	YLR372W
YJL222W	YML048W
YJL222W	YNL030W
YJL222W	YOR085W
YJL222W-A	YJR091C
YJL222W-A	YLR453C
YJL225C	YLR295C
YJR001W	YJR082C
YJR001W	YNR075W
YJR001W	YOR098C
YJR002W	YNL075W
YJR003C	YOL030W
YJR005W	YJR058C
YJR005W	YLR014C
YJR005W	YLR116W
YJR005W	YOL062C
YJR005W	YPL248C
YJR006W	YJR043C
YJR006W	YLR447C
YJR007W	YJR009C
YJR007W	YKR026C
YJR007W	YLR044C
YJR007W	YOL139C
YJR007W	YOR260W
YJR007W	YPL237W
YJR009C	YKR026C
YJR009C	YKR036C
YJR009C	YKR055W
YJR009C	YLR295C
YJR009C	YML053C
YJR009C	YMR117C
YJR009C	YNL180C
YJR009C	YNL189W
YJR009C	YNL244C
YJR009C	YNR031C
YJR009C	YOL062C
YJR009C	YOL139C
YJR009C	YOR089C

YJR009C	YPL031C
YJR009C	YPL140C
YJR009C	YPL149W
YJR009C	YPL259C
YJR009C	YPR110C
YJR010C-A	YJR091C
YJR010C-A	YJR117W
YJR010C-A	YKL008C
YJR010C-A	YKL065C
YJR010C-A	YKL154W
YJR010C-A	YLR004C
YJR010C-A	YLR372W
YJR010C-A	YLR447C
YJR010C-A	YML008C
YJR010C-A	YML048W
YJR010C-A	YML067C
YJR010C-A	YMR264W
YJR010C-A	YOL065C
YJR010C-A	YOR016C
YJR010C-A	YOR254C
YJR010C-A	YPL076W
YJR010C-A	YPL227C
YJR010W	YNL189W
YJR011C	YNR069C
YJR011C	YOR047C
YJR014W	YMR116C
YJR015W	YLR372W
YJR015W	YMR149W
YJR015W	YOR016C
YJR015W	YOR036W
YJR016C	YLR383W
YJR017C	YLL026W
YJR017C	YLR106C
YJR017C	YLR216C
YJR017C	YLR259C
YJR017C	YLR438W
YJR017C	YML010W
YJR017C	YMR066W
YJR017C	YNL014W
YJR017C	YOR151C
YJR017C	YPR086W
YJR019C	YKR026C
YJR019C	YMR047C
YJR019C	YPR106W
YJR020W	YNL092W

YJR021C	YNL189W
YJR022W	YJR077C
YJR022W	YKL173W
YJR022W	YKR021W
YJR022W	YLL015W
YJR022W	YLR133W
YJR022W	YLR143W
YJR022W	YLR147C
YJR022W	YLR264W
YJR022W	YLR269C
YJR022W	YLR430W
YJR022W	YLR438C-A
YJR022W	YMR205C
YJR022W	YMR268C
YJR022W	YNL050C
YJR022W	YNL118C
YJR022W	YNL147W
YJR022W	YNL227C
YJR022W	YNL242W
YJR022W	YNL329C
YJR022W	YNR050C
YJR022W	YNR053C
YJR022W	YOL031C
YJR022W	YOL140W
YJR022W	YOL149W
YJR022W	YOR076C
YJR022W	YOR261C
YJR022W	YOR308C
YJR022W	YOR319W
YJR022W	YPR016C
YJR022W	YPR017C
YJR022W	YPR132W
YJR022W	YPR178W
YJR022W	YPR191W
YJR023C	YNL118C
YJR024C	YLR423C
YJR025C	YLR288C
YJR025C	YML108W
YJR027W	YMR139W
YJR028W	YMR139W
YJR029W	YMR139W
YJR030C	YLR310C
YJR031C	YNL287W
YJR032W	YLL026W
YJR032W	YNL330C

YJR032W	YPL240C
YJR033C	YOR332W
YJR034W	YNL236W
YJR035W	YJR045C
YJR035W	YJR121W
YJR035W	YKL035W
YJR035W	YKL081W
YJR035W	YKL085W
YJR035W	YKL152C
YJR035W	YLR304C
YJR035W	YMR226C
YJR035W	YMR318C
YJR035W	YNL084C
YJR035W	YNR001C
YJR035W	YNR058W
YJR035W	YOL055C
YJR035W	YOL139C
YJR035W	YOR374W
YJR035W	YPL061W
YJR035W	YPL258C
YJR035W	YPR191W
YJR037W	YLR295C
YJR037W	YNL189W
YJR038C	YJR091C
YJR038C	YMR203W
YJR040W	YOR092W
YJR042W	YKL057C
YJR042W	YKL068W
YJR042W	YKR082W
YJR042W	YLR208W
YJR042W	YMR047C
YJR042W	YMR209C
YJR042W	YOL055C
YJR042W	YOR176W
YJR042W	YPL215W
YJR043C	YNL102W
YJR045C	YJR052W
YJR045C	YJR068W
YJR045C	YKL095W
YJR045C	YKL108W
YJR045C	YKL130C
YJR045C	YKL161C
YJR045C	YKR026C
YJR045C	YKR048C
YJR045C	YKR055W

YJR045C	YLL011W
YJR045C	YLL019C
YJR045C	YLR175W
YJR045C	YLR186W
YJR045C	YLR238W
YJR045C	YLR291C
YJR045C	YLR429W
YJR045C	YML016C
YJR045C	YML058W
YJR045C	YML064C
YJR045C	YML095C
YJR045C	YMR001C
YJR045C	YMR022W
YJR045C	YMR049C
YJR045C	YMR102C
YJR045C	YMR106C
YJR045C	YMR139W
YJR045C	YMR167W
YJR045C	YMR201C
YJR045C	YMR205C
YJR045C	YNL053W
YJR045C	YNL061W
YJR045C	YNL094W
YJR045C	YNL106C
YJR045C	YNL113W
YJR045C	YNL135C
YJR045C	YNL157W
YJR045C	YNL161W
YJR045C	YNL175C
YJR045C	YNL189W
YJR045C	YNL230C
YJR045C	YNL311C
YJR045C	YNR017W
YJR045C	YNR031C
YJR045C	YOL045W
YJR045C	YOL054W
YJR045C	YOL062C
YJR045C	YOL100W
YJR045C	YOL115W
YJR045C	YOL126C
YJR045C	YOL139C
YJR045C	YOR080W
YJR045C	YOR125C
YJR045C	YOR174W
YJR045C	YOR181W

YJR045C	YOR212W
YJR045C	YOR232W
YJR045C	YOR304W
YJR045C	YOR351C
YJR045C	YOR386W
YJR045C	YPL022W
YJR045C	YPL140C
YJR045C	YPL149W
YJR045C	YPL153C
YJR045C	YPL240C
YJR045C	YPL259C
YJR045C	YPL262W
YJR045C	YPR015C
YJR045C	YPR017C
YJR045C	YPR018W
YJR045C	YPR110C
YJR045C	YPR111W
YJR048W	YKR066C
YJR048W	YLR288C
YJR048W	YNL334C
YJR049C	YMR047C
YJR049C	YPL026C
YJR050W	YJR091C
YJR050W	YLL036C
YJR050W	YLR117C
YJR050W	YLR297W
YJR050W	YMR213W
YJR050W	YPL213W
YJR052W	YJR121W
YJR052W	YLR180W
YJR052W	YLR442C
YJR052W	YMR201C
YJR052W	YPL046C
YJR053W	YJR077C
YJR053W	YKL152C
YJR053W	YLR078C
YJR053W	YLR389C
YJR053W	YML064C
YJR053W	YMR001C
YJR053W	YMR055C
YJR053W	YMR319C
YJR053W	YNL238W
YJR053W	YPL061W
YJR055W	YOL030W
YJR055W	YPL193W

YJR056C	YML064C
YJR056C	YNL189W
YJR056C	YNR012W
YJR057W	YMR270C
YJR058C	YJR091C
YJR058C	YMR119W
YJR058C	YOL062C
YJR059W	YLR259C
YJR059W	YML074C
YJR059W	YOL041C
YJR059W	YPL012W
YJR060W	YKL089W
YJR060W	YMR094W
YJR060W	YMR168C
YJR060W	YNL103W
YJR060W	YPL153C
YJR061W	YKL168C
YJR062C	YJR121W
YJR062C	YKL085W
YJR062C	YKL152C
YJR062C	YLL026W
YJR062C	YMR205C
YJR062C	YNL064C
YJR062C	YOR232W
YJR062C	YOR332W
YJR062C	YPL061W
YJR063W	YJR113C
YJR063W	YLR288C
YJR063W	YNL113W
YJR063W	YOR210W
YJR063W	YOR224C
YJR063W	YOR340C
YJR063W	YOR341W
YJR063W	YPR010C
YJR063W	YPR110C
YJR063W	YPR187W
YJR064W	YKL095W
YJR064W	YKR036C
YJR064W	YLL011W
YJR064W	YLR196W
YJR064W	YMR106C
YJR064W	YNL148C
YJR064W	YNL317W
YJR064W	YNR016C
YJR064W	YOR212W

YJR064W	YPL151C
YJR065C	YKL013C
YJR065C	YLR370C
YJR065C	YMR109W
YJR065C	YNR035C
YJR065C	YNR053C
YJR065C	YOR181W
YJR065C	YPR086W
YJR066W	YKL161C
YJR066W	YKL171W
YJR066W	YKR034W
YJR066W	YLR108C
YJR066W	YLR116W
YJR066W	YMR216C
YJR066W	YNL006W
YJR066W	YNL076W
YJR066W	YNL135C
YJR066W	YNL183C
YJR066W	YNL229C
YJR066W	YNL314W
YJR066W	YOL100W
YJR066W	YOR119C
YJR066W	YPL180W
YJR067C	YLR406C
YJR068W	YJR109C
YJR068W	YJR121W
YJR068W	YLR180W
YJR068W	YLR371W
YJR068W	YMR078C
YJR068W	YMR105C
YJR068W	YNL014W
YJR068W	YNL064C
YJR068W	YNL189W
YJR068W	YNL290W
YJR068W	YOL094C
YJR068W	YOL126C
YJR068W	YOR144C
YJR068W	YPL061W
YJR070C	YKR086W
YJR070C	YNL092W
YJR072C	YKR026C
YJR072C	YLR150W
YJR072C	YLR243W
YJR072C	YMR106C
YJR072C	YOR262W

YJR072C	YPL140C
YJR072C	YPL203W
YJR074W	YLR293C
YJR074W	YOR185C
YJR075W	YLR026C
YJR076C	YKL182W
YJR076C	YLR259C
YJR076C	YLR314C
YJR076C	YML109W
YJR076C	YMR012W
YJR076C	YMR304W
YJR076C	YNL088W
YJR076C	YNL166C
YJR076C	YOR084W
YJR076C	YOR151C
YJR076C	YOR156C
YJR076C	YOR284W
YJR076C	YPL018W
YJR076C	YPL061W
YJR076C	YPL161C
YJR077C	YKR026C
YJR077C	YKR036C
YJR077C	YLR288C
YJR077C	YML064C
YJR077C	YMR059W
YJR077C	YMR106C
YJR077C	YNL006W
YJR077C	YNL012W
YJR077C	YNL056W
YJR077C	YNR031C
YJR077C	YOR125C
YJR077C	YOR181W
YJR077C	YOR386W
YJR077C	YPL140C
YJR079W	YMR047C
YJR080C	YLL024C
YJR082C	YLR217W
YJR082C	YNL004W
YJR082C	YNL051W
YJR082C	YNL094W
YJR082C	YNR028W
YJR082C	YOL105C
YJR082C	YOL129W
YJR082C	YOR354C
YJR082C	YPR096C

YJR082C	YPR173C
YJR083C	YLR191W
YJR083C	YLR288C
YJR083C	YLR295C
YJR083C	YPL211W
YJR083C	YPR154W
YJR084W	YPR182W
YJR086W	YOR212W
YJR087W	YJR091C
YJR089W	YPL209C
YJR090C	YLL050C
YJR090C	YLR134W
YJR090C	YMR032W
YJR090C	YMR199W
YJR090C	YMR205C
YJR090C	YNL055C
YJR090C	YNL071W
YJR090C	YNR001C
YJR090C	YOL055C
YJR090C	YOL133W
YJR090C	YOR374W
YJR090C	YPL256C
YJR091C	YJR119C
YJR091C	YJR123W
YJR091C	YJR133W
YJR091C	YKL002W
YJR091C	YKL076C
YJR091C	YKL111C
YJR091C	YKL113C
YJR091C	YKL117W
YJR091C	YKL138C
YJR091C	YKL144C
YJR091C	YKL146W
YJR091C	YKL160W
YJR091C	YKL224C
YJR091C	YKR037C
YJR091C	YKR040C
YJR091C	YKR087C
YJR091C	YKR105C
YJR091C	YLL009C
YJR091C	YLL030C
YJR091C	YLL042C
YJR091C	YLL065W
YJR091C	YLR009W
YJR091C	YLR035C

YJR091C	YLR037C
YJR091C	YLR051C
YJR091C	YLR059C
YJR091C	YLR066W
YJR091C	YLR068W
YJR091C	YLR092W
YJR091C	YLR104W
YJR091C	YLR118C
YJR091C	YLR153C
YJR091C	YLR172C
YJR091C	YLR191W
YJR091C	YLR205C
YJR091C	YLR248W
YJR091C	YLR269C
YJR091C	YLR283W
YJR091C	YLR292C
YJR091C	YLR293C
YJR091C	YLR322W
YJR091C	YLR324W
YJR091C	YLR333C
YJR091C	YLR370C
YJR091C	YLR379W
YJR091C	YLR386W
YJR091C	YLR392C
YJR091C	YLR397C
YJR091C	YLR413W
YJR091C	YLR420W
YJR091C	YML013W
YJR091C	YML015C
YJR091C	YML018C
YJR091C	YML041C
YJR091C	YML042W
YJR091C	YML123C
YJR091C	YML126C
YJR091C	YMR049C
YJR091C	YMR052W
YJR091C	YMR067C
YJR091C	YMR104C
YJR091C	YMR160W
YJR091C	YMR187C
YJR091C	YMR197C
YJR091C	YMR199W
YJR091C	YMR202W
YJR091C	YMR253C
YJR091C	YMR263W

YJR091C	YMR278W
YJR091C	YMR287C
YJR091C	YMR293C
YJR091C	YNL008C
YJR091C	YNL024C
YJR091C	YNL032W
YJR091C	YNL092W
YJR091C	YNL105W
YJR091C	YNL109W
YJR091C	YNL144C
YJR091C	YNL146W
YJR091C	YNL171C
YJR091C	YNL176C
YJR091C	YNL214W
YJR091C	YNL215W
YJR091C	YNL230C
YJR091C	YNL241C
YJR091C	YNL266W
YJR091C	YNL285W
YJR091C	YNR037C
YJR091C	YNR048W
YJR091C	YNR071C
YJR091C	YOL072W
YJR091C	YOL101C
YJR091C	YOL145C
YJR091C	YOR008C
YJR091C	YOR014W
YJR091C	YOR033C
YJR091C	YOR097C
YJR091C	YOR114W
YJR091C	YOR116C
YJR091C	YOR122C
YJR091C	YOR167C
YJR091C	YOR189W
YJR091C	YOR197W
YJR091C	YOR209C
YJR091C	YOR265W
YJR091C	YOR303W
YJR091C	YOR314W
YJR091C	YOR317W
YJR091C	YOR379C
YJR091C	YPL004C
YJR091C	YPL032C
YJR091C	YPL052W
YJR091C	YPL088W

YJR091C	YPL092W
YJR091C	YPL140C
YJR091C	YPL158C
YJR091C	YPL159C
YJR091C	YPL219W
YJR091C	YPL220W
YJR091C	YPL270W
YJR091C	YPL271W
YJR091C	YPR003C
YJR091C	YPR047W
YJR091C	YPR096C
YJR091C	YPR114W
YJR091C	YPR120C
YJR091C	YPR126C
YJR091C	YPR130C
YJR091C	YPR148C
YJR091C	YPR178W
YJR091C	YPR188C
YJR092W	YMR032W
YJR093C	YKL059C
YJR093C	YKR002W
YJR093C	YLR115W
YJR093C	YLR221C
YJR093C	YLR277C
YJR093C	YML030W
YJR093C	YMR061W
YJR093C	YNL092W
YJR093C	YNL189W
YJR093C	YNL317W
YJR093C	YPR107C
YJR094C	YMR139W
YJR097W	YNL092W
YJR098C	YMR304W
YJR099W	YKL181W
YJR099W	YOR047C
YJR100C	YLR295C
YJR102C	YLR295C
YJR102C	YLR417W
YJR102C	YML101C
YJR102C	YMR077C
YJR102C	YPL002C
YJR103W	YLR147C
YJR104C	YKL095W
YJR104C	YMR038C
YJR104C	YMR059W

YJR104C	YNL135C
YJR104C	YPL031C
YJR105W	YLR301W
YJR105W	YNL106C
YJR109C	YLR314C
YJR109C	YMR106C
YJR109C	YNL244C
YJR109C	YOR302W
YJR109C	YOR303W
YJR109C	YOR351C
YJR109C	YPL031C
YJR109C	YPR110C
YJR110W	YLR002C
YJR110W	YLR071C
YJR110W	YLR259C
YJR110W	YMR049C
YJR110W	YOL054W
YJR110W	YOR069W
YJR110W	YOR151C
YJR110W	YOR206W
YJR112W	YLR086W
YJR112W	YLR200W
YJR112W	YLR288C
YJR112W	YMR198W
YJR112W	YNL250W
YJR112W	YOL069W
YJR112W	YOR269W
YJR112W	YOR326W
YJR112W	YPR141C
YJR113C	YKL048C
YJR116W	YOR299W
YJR117W	YLR018C
YJR117W	YML123C
YJR117W	YMR215W
YJR117W	YNL048W
YJR117W	YNL101W
YJR117W	YNR019W
YJR117W	YPL076W
YJR117W	YPL274W
YJR119C	YLR014C
YJR120W	YLR295C
YJR121W	YKL016C
YJR121W	YKL048C
YJR121W	YKL193C
YJR121W	YKR036C

YJR121W YLL019C
YJR121W YLR016C
YJR121W YLR196W
YJR121W YLR222C
YJR121W YLR262C
YJR121W YLR295C
YJR121W YLR306W
YJR121W YLR340W
YJR121W YLR442C
YJR121W YLR453C
YJR121W YML057W
YJR121W YML081C-A
YJR121W YML095C
YJR121W YML112W
YJR121W YMR049C
YJR121W YMR055C
YJR121W YMR104C
YJR121W YMR106C
YJR121W YMR138W
YJR121W YMR205C
YJR121W YMR284W
YJR121W YNL061W
YJR121W YNL090W
YJR121W YNL094W
YJR121W YNL113W
YJR121W YNL135C
YJR121W YNL250W
YJR121W YNL290W
YJR121W YNL312W
YJR121W YNL313C
YJR121W YNL315C
YJR121W YOL087C
YJR121W YOL094C
YJR121W YOL100W
YJR121W YOL115W
YJR121W YOL133W
YJR121W YOR181W
YJR121W YOR229W
YJR121W YOR230W
YJR121W YOR231W
YJR121W YOR351C
YJR121W YOR386W
YJR121W YPL078C
YJR121W YPL140C
YJR121W YPL150W

YJR121W	YPL151C
YJR121W	YPL153C
YJR121W	YPL204W
YJR121W	YPL271W
YJR121W	YPR020W
YJR121W	YPR110C
YJR121W	YPR178W
YJR125C	YOR111W
YJR127C	YKR086W
YJR131W	YLR083C
YJR131W	YML064C
YJR132W	YLR291C
YJR132W	YLR310C
YJR132W	YMR080C
YJR132W	YNL027W
YJR132W	YNL030W
YJR132W	YNL323W
YJR132W	YOR098C
YJR132W	YOR177C
YJR132W	YOR326W
YJR133W	YLR295C
YJR133W	YNL189W
YJR134C	YLR262C
YJR134C	YMR047C
YJR134C	YPL204W
YJR135C	YPR046W
YJR135W-A	YNR017W
YJR136C	YKL033W
YJR136C	YMR047C
YJR138W	YLR096W
YJR138W	YLR438C-A
YJR138W	YMR304W
YJR138W	YNL016W
YJR138W	YNL030W
YJR138W	YNL307C
YJR138W	YOL145C
YJR139C	YOR061W
YJR140C	YML058W
YJR140C	YNL312W
YJR141W	YLR295C
YJR141W	YLR447C
YJR141W	YMR047C
YJR144W	YLR074C
YJR144W	YLR427W
YJR144W	YMR137C

YJR144W	YNL312W
YJR144W	YPR161C
YJR146W	YLR246W
YJR146W	YLR288C
YJR152W	YPR003C
YJR154W	YLR295C
YJR157W	YLR288C
YJR159W	YLR447C
YJR159W	YML064C
YJR159W	YMR308C
YJR159W	YNL189W
YJR159W	YOR098C
YJR159W	YPL031C
YJR162C	YMR032W
YKL001C	YLR288C
YKL001C	YNL311C
YKL002W	YLR108C
YKL002W	YLR295C
YKL002W	YLR423C
YKL002W	YMR117C
YKL002W	YOR047C
YKL002W	YOR284W
YKL004W	YNR072W
YKL006C-A	YLR026C
YKL006C-A	YMR215W
YKL006C-A	YNL092W
YKL007W	YPR171W
YKL008C	YKL065C
YKL008C	YKL146W
YKL008C	YKL174C
YKL008C	YLR372W
YKL008C	YML064C
YKL008C	YMR058W
YKL008C	YMR215W
YKL008C	YMR298W
YKL008C	YNL107W
YKL008C	YPL076W
YKL008C	YPL264C
YKL008C	YPR048W
YKL009W	YLR074C
YKL009W	YMR049C
YKL009W	YNL061W
YKL009W	YOR267C
YKL009W	YPR016C
YKL010C	YKL189W

YKL010C	YML064C
YKL010C	YPL259C
YKL011C	YKR026C
YKL011C	YLR259C
YKL011C	YMR201C
YKL011C	YMR205C
YKL011C	YPR122W
YKL012W	YLR116W
YKL012W	YLR298C
YKL012W	YMR125W
YKL012W	YNL236W
YKL012W	YPR182W
YKL013C	YKL129C
YKL013C	YLR370C
YKL013C	YMR109W
YKL013C	YNR035C
YKL014C	YLR233C
YKL014C	YNL061W
YKL014C	YOR014W
YKL014C	YOR080W
YKL014C	YPL043W
YKL016C	YML081C-A
YKL017C	YOL108C
YKL017C	YOR167C
YKL018W	YKL059C
YKL018W	YKL060C
YKL018W	YLR015W
YKL018W	YLR115W
YKL018W	YLR277C
YKL018W	YNL317W
YKL018W	YOR179C
YKL018W	YPL138C
YKL019W	YML097C
YKL020C	YLR233C
YKL020C	YOR098C
YKL021C	YMR049C
YKL021C	YNL061W
YKL021C	YPR016C
YKL022C	YLR102C
YKL022C	YLR127C
YKL022C	YNL172W
YKL022C	YOR249C
YKL023W	YKR092C
YKL023W	YNL189W
YKL024C	YLR288C

YKL025C	YKL129C
YKL025C	YLR447C
YKL025C	YNL092W
YKL025C	YPL169C
YKL028W	YKR062W
YKL028W	YNL277W
YKL028W	YOL051W
YKL029C	YLR447C
YKL029C	YMR090W
YKL029C	YPR054W
YKL035W	YLR442C
YKL035W	YML064C
YKL035W	YMR059W
YKL035W	YNL032W
YKL035W	YOL045W
YKL035W	YOR181W
YKL035W	YOR386W
YKL035W	YPL149W
YKL035W	YPL150W
YKL035W	YPL204W
YKL035W	YPR110C
YKL036C	YKL126W
YKL036C	YKL160W
YKL038W	YOR047C
YKL042W	YNL053W
YKL042W	YNL225C
YKL042W	YOL069W
YKL042W	YPL124W
YKL044W	YLR288C
YKL045W	YNL262W
YKL045W	YNR052C
YKL047W	YPL130W
YKL048C	YLR096W
YKL049C	YPL018W
YKL050C	YLR373C
YKL050C	YLR423C
YKL052C	YKR083C
YKL052C	YLR288C
YKL052C	YMR077C
YKL054C	YMR106C
YKL056C	YLR285W
YKL056C	YMR059W
YKL056C	YPL204W
YKL057C	YKL068W
YKL057C	YKR082W

YKL057C YLR208W
YKL057C YLR216C
YKL057C YNR012W
YKL057C YOR142W-A
YKL058W YLR288C
YKL058W YOR194C
YKL058W YPR086W
YKL059C YKR002W
YKL059C YLR115W
YKL059C YLR277C
YKL059C YMR061W
YKL059C YNL317W
YKL059C YOR344C
YKL059C YPR107C
YKL060C YKL103C
YKL060C YLR175W
YKL060C YLR295C
YKL060C YLR314C
YKL060C YML064C
YKL060C YML115C
YKL060C YMR049C
YKL060C YNL127W
YKL060C YNL180C
YKL060C YNL189W
YKL060C YNL244C
YKL060C YOL062C
YKL060C YOL135C
YKL060C YOR061W
YKL060C YOR116C
YKL060C YOR174W
YKL060C YPL031C
YKL060C YPL149W
YKL060C YPL203W
YKL060C YPR110C
YKL061W YMR047C
YKL061W YNL086W
YKL061W YNL122C
YKL061W YOR098C
YKL062W YPL203W
YKL062W YPL204W
YKL064W YLR138W
YKL064W YNL307C
YKL065C YKL212W
YKL065C YLL023C
YKL065C YLL028W

YKL065C	YLL061W
YKL065C	YLR018C
YKL065C	YLR034C
YKL065C	YML123C
YKL065C	YMR058W
YKL065C	YMR149W
YKL065C	YMR215W
YKL065C	YNL048W
YKL065C	YNL101W
YKL065C	YPL076W
YKL065C	YPL264C
YKL065C	YPR124W
YKL065C	YPR156C
YKL065C	YPR198W
YKL067W	YKL095W
YKL067W	YLR347C
YKL067W	YML064C
YKL067W	YMR059W
YKL067W	YNL030W
YKL067W	YNL189W
YKL067W	YPR010C
YKL068W	YKR082W
YKL068W	YLL024C
YKL068W	YLR335W
YKL068W	YLR347C
YKL068W	YML103C
YKL068W	YMR047C
YKL068W	YMR308C
YKL068W	YNL189W
YKL068W	YNL243W
YKL068W	YNL273W
YKL068W	YNL298W
YKL068W	YOL123W
YKL068W	YPL093W
YKL068W	YPL125W
YKL068W	YPL169C
YKL068W	YPR119W
YKL068W	YPR120C
YKL069W	YNL189W
YKL072W	YLR014C
YKL072W	YOL004W
YKL073W	YOL087C
YKL074C	YLR116W
YKL074C	YLR357W
YKL074C	YML046W

YKL074C	YMR216C
YKL074C	YPL016W
YKL074C	YPL105C
YKL075C	YKR086W
YKL075C	YNL091W
YKL075C	YOR148C
YKL078W	YLR180W
YKL078W	YLR259C
YKL078W	YMR205C
YKL078W	YNL064C
YKL079W	YMR047C
YKL080W	YLR295C
YKL080W	YML064C
YKL080W	YMR054W
YKL080W	YMR059W
YKL080W	YOR270C
YKL081W	YKL104C
YKL081W	YLR074C
YKL081W	YLR096W
YKL081W	YLR113W
YKL081W	YLR442C
YKL081W	YML057W
YKL081W	YML064C
YKL081W	YMR059W
YKL081W	YNL023C
YKL081W	YPL226W
YKL082C	YLR353W
YKL082C	YML109W
YKL082C	YOR233W
YKL082C	YOR299W
YKL082C	YPL141C
YKL085W	YKL108W
YKL085W	YLR074C
YKL085W	YNL094W
YKL085W	YNR001C
YKL085W	YOL128C
YKL085W	YPL111W
YKL085W	YPL153C
YKL085W	YPL204W
YKL085W	YPR110C
YKL086W	YNL141W
YKL087C	YLR285W
YKL088W	YLR295C
YKL088W	YML016C
YKL088W	YMR047C

YKL088W	YOR039W
YKL090W	YPL128C
YKL092C	YPL095C
YKL092C	YPL256C
YKL093W	YOR239W
YKL095W	YKL173W
YKL095W	YKR048C
YKL095W	YLL036C
YKL095W	YLR117C
YKL095W	YLR259C
YKL095W	YLR424W
YKL095W	YMR125W
YKL095W	YNL229C
YKL095W	YNR001C
YKL095W	YOL055C
YKL095W	YPL037C
YKL095W	YPL061W
YKL095W	YPL151C
YKL095W	YPL213W
YKL095W	YPL258C
YKL095W	YPR101W
YKL095W	YPR191W
YKL098W	YPR016C
YKL098W	YPR041W
YKL101W	YKR048C
YKL101W	YLR314C
YKL101W	YMR032W
YKL101W	YNL132W
YKL103C	YKL152C
YKL103C	YLL013C
YKL103C	YLR044C
YKL103C	YLR295C
YKL103C	YLR355C
YKL103C	YML064C
YKL103C	YMR104C
YKL103C	YMR186W
YKL103C	YMR290C
YKL103C	YNL189W
YKL103C	YOL001W
YKL103C	YOL082W
YKL103C	YOR302W
YKL103C	YOR332W
YKL103C	YPL204W
YKL104C	YKL116C
YKL104C	YKL161C

YKL104C	YKR026C
YKL104C	YLR058C
YKL104C	YLR291C
YKL104C	YMR117C
YKL104C	YMR308C
YKL104C	YNL313C
YKL104C	YOL062C
YKL104C	YOL126C
YKL104C	YOR212W
YKL104C	YOR351C
YKL104C	YPL111W
YKL104C	YPL259C
YKL104C	YPR106W
YKL104C	YPR110C
YKL104C	YPR178W
YKL106W	YLR447C
YKL107W	YLR288C
YKL108W	YLR191W
YKL108W	YLR259C
YKL108W	YLR354C
YKL108W	YMR287C
YKL108W	YMR303C
YKL108W	YNL189W
YKL108W	YNR001C
YKL108W	YOR232W
YKL109W	YOR358W
YKL110C	YPL086C
YKL112W	YOR039W
YKL112W	YOR061W
YKL113C	YLR447C
YKL113C	YLR453C
YKL114C	YOR264W
YKL116C	YLR389C
YKL116C	YPL031C
YKL117W	YLR216C
YKL117W	YMR186W
YKL117W	YOR027W
YKL117W	YPL240C
YKL119C	YLR292C
YKL119C	YOR270C
YKL120W	YLR207W
YKL120W	YNR055C
YKL122C	YPR088C
YKL125W	YML126C
YKL126W	YKL203C

YKL126W YML006C
YKL126W YNR047W
YKL126W YOL078W
YKL126W YPR041W
YKL126W YPR111W
YKL127W YLR295C
YKL127W YOR269W
YKL129C YKL152C
YKL129C YKL159C
YKL129C YKR069W
YKL129C YLR337C
YKL129C YMR109W
YKL129C YNL025C
YKL129C YNL084C
YKL129C YNL094W
YKL129C YNL138W
YKL129C YNL271C
YKL129C YNL298W
YKL129C YOR047C
YKL129C YOR181W
YKL129C YOR184W
YKL129C YOR247W
YKL129C YOR389W
YKL129C YPL038W
YKL129C YPL277C
YKL130C YKR022C
YKL130C YLR037C
YKL130C YLR044C
YKL130C YML088W
YKL130C YML106W
YKL130C YMR314W
YKL130C YNL023C
YKL130C YNL189W
YKL130C YOL073C
YKL130C YPR020W
YKL133C YNL118C
YKL134C YPL109C
YKL134C YPR086W
YKL135C YLR170C
YKL135C YMR308C
YKL135C YNL189W
YKL135C YOL108C
YKL135C YPL259C
YKL135C YPR010C
YKL135C YPR029C

YKL137W YML043C
YKL139W YKL152C
YKL139W YLR180W
YKL139W YLR403W
YKL139W YML112W
YKL139W YNL004W
YKL139W YNL030W
YKL139W YNL132W
YKL140W YNL001W
YKL140W YPL204W
YKL142W YMR165C
YKL142W YMR210W
YKL142W YMR243C
YKL142W YNL161W
YKL142W YNL210W
YKL143W YLR295C
YKL143W YMR047C
YKL143W YMR257C
YKL143W YNL178W
YKL143W YNL207W
YKL143W YPL204W
YKL144C YKL205W
YKL144C YKR025W
YKL144C YNR003C
YKL144C YOR116C
YKL144C YOR207C
YKL144C YOR210W
YKL144C YOR224C
YKL144C YPR110C
YKL144C YPR187W
YKL144C YPR190C
YKL145W YLR421C
YKL145W YML099C
YKL145W YMR001C
YKL145W YOL145C
YKL145W YOR117W
YKL145W YOR123C
YKL145W YOR259C
YKL145W YOR261C
YKL145W YPR108W
YKL146W YLR246W
YKL146W YOR264W
YKL147C YMR032W
YKL150W YPR154W
YKL152C YKL210W

YKL152C	YKL215C
YKL152C	YLL011W
YKL152C	YLL050C
YKL152C	YLR148W
YKL152C	YLR288C
YKL152C	YLR306W
YKL152C	YLR320W
YKL152C	YLR427W
YKL152C	YML064C
YKL152C	YMR049C
YKL152C	YMR055C
YKL152C	YMR059W
YKL152C	YMR106C
YKL152C	YMR109W
YKL152C	YNL006W
YKL152C	YNL127W
YKL152C	YNL128W
YKL152C	YNL290W
YKL152C	YNR010W
YKL152C	YNR019W
YKL152C	YOL113W
YKL152C	YOR276W
YKL152C	YOR319W
YKL152C	YOR341W
YKL152C	YPL150W
YKL152C	YPL164C
YKL152C	YPL204W
YKL152C	YPR054W
YKL152C	YPR110C
YKL152C	YPR111W
YKL153W	YNL092W
YKL154W	YKL212W
YKL154W	YLL023C
YKL154W	YLR288C
YKL154W	YLR378C
YKL154W	YML067C
YKL154W	YML075C
YKL154W	YMR058W
YKL154W	YMR149W
YKL154W	YMR215W
YKL154W	YNL101W
YKL154W	YPL076W
YKL154W	YPL264C
YKL154W	YPL274W
YKL154W	YPR198W

YKL157W	YMR307W
YKL157W	YPR168W
YKL159C	YLR433C
YKL161C	YLR096W
YKL161C	YLR180W
YKL161C	YNL053W
YKL161C	YNL064C
YKL161C	YOR151C
YKL161C	YPL049C
YKL161C	YPL089C
YKL161C	YPL140C
YKL163W	YLR288C
YKL164C	YLR447C
YKL165C	YLR372W
YKL165C	YPR028W
YKL166C	YLR216C
YKL166C	YML001W
YKL166C	YMR205C
YKL166C	YNL093W
YKL166C	YNL227C
YKL166C	YOR151C
YKL166C	YPL203W
YKL166C	YPR160W
YKL168C	YLR012C
YKL171W	YKL203C
YKL171W	YNL006W
YKL171W	YNL229C
YKL171W	YNL307C
YKL171W	YPL180W
YKL171W	YPL204W
YKL171W	YPR181C
YKL172W	YKR081C
YKL172W	YMR049C
YKL172W	YNL175C
YKL172W	YNL230C
YKL172W	YOR267C
YKL172W	YOR294W
YKL173W	YLR117C
YKL173W	YLR147C
YKL173W	YLR438C-A
YKL173W	YMR213W
YKL173W	YNL147W
YKL173W	YOR159C
YKL173W	YPR178W
YKL173W	YPR182W

YKL174C	YLL006W
YKL174C	YLR345W
YKL174C	YPL036W
YKL175W	YPR198W
YKL176C	YLR264W
YKL176C	YOR080W
YKL178C	YOL044W
YKL179C	YPL022W
YKL180W	YPR132W
YKL181W	YOL061W
YKL182W	YLL011W
YKL182W	YLR288C
YKL182W	YMR026C
YKL182W	YMR106C
YKL182W	YNL250W
YKL182W	YOL087C
YKL182W	YOL133W
YKL182W	YPL231W
YKL182W	YPR110C
YKL183W	YLR288C
YKL183W	YNL104C
YKL186C	YPL138C
YKL189W	YLR259C
YKL189W	YMR058W
YKL189W	YMR303C
YKL189W	YNL064C
YKL190W	YLR433C
YKL190W	YML057W
YKL190W	YMR211W
YKL190W	YPR159W
YKL192C	YPL163C
YKL193C	YLR259C
YKL193C	YML016C
YKL193C	YML049C
YKL193C	YPL036W
YKL193C	YPL179W
YKL193C	YPL235W
YKL194C	YPL203W
YKL196C	YLR026C
YKL196C	YLR093C
YKL196C	YMR197C
YKL196C	YOR106W
YKL197C	YNL329C
YKL198C	YMR229C
YKL198C	YNR016C

YKL198C	YOR198C
YKL198C	YOR298C-A
YKL201C	YOR003W
YKL203C	YMR036C
YKL203C	YMR068W
YKL203C	YNL006W
YKL203C	YNL135C
YKL203C	YNL183C
YKL203C	YNL229C
YKL203C	YOL078W
YKL203C	YOR014W
YKL204W	YNL154C
YKL204W	YNL161W
YKL204W	YOL139C
YKL205W	YLR291C
YKL205W	YLR293C
YKL205W	YNR034W
YKL205W	YOR374W
YKL206C	YPL051W
YKL208W	YPR086W
YKL209C	YLR292C
YKL210W	YLR096W
YKL210W	YML112W
YKL210W	YMR059W
YKL210W	YOR027W
YKL210W	YOR184W
YKL211C	YKR026C
YKL212W	YLR350W
YKL212W	YLR372W
YKL212W	YMR296C
YKL212W	YPR028W
YKL213C	YOR116C
YKL213C	YOR341W
YKL213C	YPR010C
YKL214C	YMR125W
YKL214C	YMR308C
YKL214C	YPL169C
YKL214C	YPR154W
YKL215C	YLL026W
YKL216W	YKR055W
YKL216W	YNL135C
YKL218C	YPR110C
YKL219W	YLL061W
YKL221W	YMR047C
YKL221W	YOR221C

YKL224C	YLR030W
YKR001C	YLR176C
YKR001C	YNL330C
YKR001C	YPL082C
YKR002W	YLR115W
YKR002W	YLR277C
YKR002W	YMR061W
YKR002W	YNL189W
YKR002W	YNL222W
YKR002W	YNL317W
YKR002W	YPR107C
YKR005C	YLR447C
YKR007W	YML121W
YKR010C	YOL006C
YKR010C	YOR061W
YKR010C	YPR172W
YKR014C	YML064C
YKR014C	YNL093W
YKR014C	YNL263C
YKR014C	YOR089C
YKR016W	YLR388W
YKR017C	YOL055C
YKR017C	YOR294W
YKR020W	YLR262C
YKR021W	YOL014W
YKR021W	YOR244W
YKR022C	YLR424W
YKR022C	YNL258C
YKR024C	YLR233C
YKR024C	YNL175C
YKR025W	YNR003C
YKR025W	YOR116C
YKR025W	YPR110C
YKR026C	YKR046C
YKR026C	YLR044C
YKR026C	YLR081W
YKR026C	YLR259C
YKR026C	YLR289W
YKR026C	YLR291C
YKR026C	YLR432W
YKR026C	YML085C
YKR026C	YML124C
YKR026C	YMR021C
YKR026C	YMR145C
YKR026C	YMR241W

YKR026C	YMR267W
YKR026C	YMR269W
YKR026C	YNL085W
YKR026C	YNL265C
YKR026C	YOR047C
YKR026C	YOR128C
YKR026C	YOR133W
YKR026C	YOR190W
YKR026C	YOR260W
YKR026C	YOR317W
YKR026C	YOR374W
YKR026C	YOR375C
YKR026C	YPL204W
YKR026C	YPL237W
YKR026C	YPR104C
YKR027W	YLR191W
YKR028W	YNL106C
YKR028W	YPL204W
YKR028W	YPR040W
YKR029C	YMR273C
YKR029C	YNL030W
YKR029C	YOL068C
YKR030W	YOR198C
YKR030W	YPL051W
YKR031C	YNL118C
YKR034W	YLR376C
YKR034W	YNL021W
YKR036C	YLR206W
YKR036C	YPL042C
YKR036C	YPR088C
YKR037C	YLR423C
YKR038C	YML036W
YKR048C	YLL002W
YKR048C	YLR133W
YKR048C	YLR347C
YKR048C	YLR457C
YKR048C	YMR139W
YKR048C	YMR172W
YKR048C	YNL078W
YKR048C	YOL012C
YKR048C	YOL070C
YKR048C	YOL115W
YKR048C	YOR090C
YKR048C	YOR276W
YKR048C	YPL150W

YKR048C	YPL180W
YKR048C	YPR119W
YKR050W	YKR088C
YKR051W	YMR106C
YKR052C	YLR288C
YKR052C	YNL275W
YKR054C	YMR117C
YKR054C	YNL030W
YKR054C	YNL118C
YKR055W	YLR259C
YKR055W	YMR186W
YKR055W	YNL127W
YKR055W	YPL161C
YKR061W	YLR191W
YKR062W	YOL051W
YKR063C	YLR403W
YKR064W	YOR098C
YKR065C	YLR036C
YKR065C	YLR241W
YKR066C	YMR089C
YKR068C	YLR342W
YKR068C	YML077W
YKR068C	YMR218C
YKR068C	YOR115C
YKR069W	YMR109W
YKR069W	YOL038W
YKR070W	YOR299W
YKR071C	YMR304W
YKR072C	YML016C
YKR074W	YLR446W
YKR077W	YPR120C
YKR080W	YLR147C
YKR081C	YLL008W
YKR081C	YLL034C
YKR081C	YLR002C
YKR081C	YLR074C
YKR081C	YLR233C
YKR081C	YLR276C
YKR081C	YLR427W
YKR081C	YLR449W
YKR081C	YMR049C
YKR081C	YMR290C
YKR081C	YNL002C
YKR081C	YNL061W
YKR081C	YNL110C

YKR081C	YNL230C
YKR081C	YNR053C
YKR081C	YOL077C
YKR081C	YOL102C
YKR081C	YOR005C
YKR081C	YOR206W
YKR081C	YOR233W
YKR081C	YOR267C
YKR081C	YOR272W
YKR081C	YPL093W
YKR081C	YPL141C
YKR081C	YPL211W
YKR081C	YPR016C
YKR082W	YLR208W
YKR082W	YMR047C
YKR082W	YMR109W
YKR083C	YLR423C
YKR084C	YLR456W
YKR086W	YLR118C
YKR086W	YLR462W
YKR086W	YOL021C
YKR086W	YOL063C
YKR086W	YPL023C
YKR086W	YPL067C
YKR086W	YPL242C
YKR086W	YPR025C
YKR086W	YPR082C
YKR086W	YPR177C
YKR088C	YLR080W
YKR088C	YPL053C
YKR088C	YPR079W
YKR090W	YOR264W
YKR091W	YLR288C
YKR091W	YNL309W
YKR092C	YOR060C
YKR092C	YOR267C
YKR093W	YPR079W
YKR095W	YLR096W
YKR095W	YPL140C
YKR095W-A	YML036W
YKR096W	YPL204W
YKR099W	YLR058C
YKR099W	YLR438C-A
YKR100C	YLR447C
YKR101W	YLR442C

YKR101W	YML065W
YKR101W	YML109W
YKR101W	YPR018W
YLL001W	YNL118C
YLL001W	YOR098C
YLL001W	YPL204W
YLL002W	YNL246W
YLL003W	YOR257W
YLL004W	YML065W
YLL004W	YNL261W
YLL004W	YPR120C
YLL004W	YPR162C
YLL005C	YML075C
YLL006W	YLR372W
YLL006W	YOL009C
YLL006W	YOR112W
YLL008W	YNL001W
YLL008W	YNL061W
YLL008W	YNL110C
YLL008W	YNL175C
YLL008W	YOR233W
YLL008W	YOR267C
YLL008W	YPL043W
YLL008W	YPR016C
YLL010C	YOR043W
YLL011W	YLR259C
YLL011W	YMR229C
YLL011W	YNL132W
YLL012W	YNR075W
YLL012W	YOR095C
YLL013C	YLR067C
YLL013C	YLR423C
YLL013C	YML091C
YLL013C	YML124C
YLL013C	YMR056C
YLL013C	YMR106C
YLL013C	YOL128C
YLL013C	YPL204W
YLL015W	YLL023C
YLL018C	YLR310C
YLL018C	YOR282W
YLL019C	YLR196W
YLL019C	YLR259C
YLL019C	YLR438W
YLL019C	YMR229C

YLL019C	YNL004W
YLL019C	YNL189W
YLL019C	YOL041C
YLL019C	YOL054W
YLL019C	YOL055C
YLL019C	YOR017W
YLL019C	YOR201C
YLL019C	YOR374W
YLL019C	YPL012W
YLL019C	YPL043W
YLL019C	YPL093W
YLL020C	YPL190C
YLL021W	YLR147C
YLL021W	YLR319C
YLL021W	YLR362W
YLL021W	YMR308C
YLL021W	YNL271C
YLL021W	YNL293W
YLL021W	YOL112W
YLL021W	YOR231W
YLL021W	YPL140C
YLL022C	YPL001W
YLL022C	YPR086W
YLL023C	YLL056C
YLL023C	YLR288C
YLL023C	YLR372W
YLL023C	YML048W
YLL023C	YMR215W
YLL023C	YMR292W
YLL023C	YNL044W
YLL023C	YNR026C
YLL023C	YOL030W
YLL023C	YPR028W
YLL024C	YLR310C
YLL024C	YLR459W
YLL024C	YMR047C
YLL024C	YNL007C
YLL024C	YNL077W
YLL024C	YOR060C
YLL024C	YPL240C
YLL026W	YLR096W
YLL026W	YLR175W
YLL026W	YMR047C
YLL026W	YNL064C
YLL026W	YNL135C

YLL026W	YOL094C
YLL026W	YOR027W
YLL026W	YPL204W
YLL027W	YOL133W
YLL028W	YLR018C
YLL028W	YLR056W
YLL028W	YLR372W
YLL028W	YML048W
YLL028W	YMR149W
YLL028W	YMR215W
YLL028W	YMR264W
YLL028W	YNL237W
YLL028W	YNL238W
YLL028W	YOR016C
YLL028W	YOR254C
YLL028W	YPL234C
YLL030C	YPR054W
YLL032C	YLR191W
YLL032C	YML119W
YLL033W	YLR447C
YLL033W	YNL180C
YLL034C	YLR180W
YLL034C	YNL110C
YLL034C	YOL133W
YLL034C	YOR341W
YLL034C	YPR010C
YLL036C	YLR117C
YLL036C	YMR213W
YLL036C	YPL151C
YLL036C	YPR101W
YLL036C	YPR182W
YLL038C	YPL161C
YLL038C	YPL242C
YLL040C	YMR078C
YLL040C	YOL133W
YLL043W	YMR104C
YLL043W	YPL192C
YLL047W	YMR069W
YLL048C	YMR106C
YLL048C	YPL147W
YLL049W	YLR423C
YLL049W	YMR294W
YLL049W	YNR069C
YLL050C	YLR059C
YLL050C	YLR074C

YLL050C	YLR429W
YLL050C	YLR453C
YLL050C	YML064C
YLL050C	YMR059W
YLL050C	YMR092C
YLL050C	YNL071W
YLL050C	YNL094W
YLL050C	YNL138W
YLL050C	YOL055C
YLL050C	YOR080W
YLL050C	YOR181W
YLL050C	YPR033C
YLL059C	YML011C
YLL061W	YLR372W
YLL061W	YML048W
YLL062C	YOL143C
YLL067W-A	YMR159C
YLR001C	YNL019C
YLR002C	YMR049C
YLR002C	YOR267C
YLR002C	YPR016C
YLR003C	YLR288C
YLR004C	YLR372W
YLR004C	YML067C
YLR004C	YPL076W
YLR005W	YLR347C
YLR005W	YNL135C
YLR006C	YLR233C
YLR006C	YMR022W
YLR006C	YNR031C
YLR007W	YLR383W
YLR007W	YOL034W
YLR008C	YLR125W
YLR009W	YLR074C
YLR009W	YLR288C
YLR009W	YNL061W
YLR011W	YLR288C
YLR011W	YLR447C
YLR014C	YML042W
YLR015W	YPL138C
YLR016C	YLR086W
YLR016C	YLR259C
YLR016C	YLR453C
YLR016C	YNL189W
YLR018C	YLR372W

YLR018C	YML048W
YLR018C	YMR264W
YLR018C	YNL279W
YLR018C	YPR028W
YLR019W	YLR310C
YLR019W	YMR012W
YLR019W	YMR205C
YLR019W	YMR246W
YLR019W	YOR043W
YLR019W	YOR061W
YLR019W	YOR341W
YLR019W	YPR010C-A
YLR021W	YLR288C
YLR021W	YPL144W
YLR024C	YMR049C
YLR025W	YLR191W
YLR025W	YLR447C
YLR025W	YMR154C
YLR025W	YOR275C
YLR025W	YPR173C
YLR026C	YLR078C
YLR026C	YLR268W
YLR026C	YMR197C
YLR026C	YNL041C
YLR026C	YNL049C
YLR026C	YPR105C
YLR026C	YPR181C
YLR027C	YLR314C
YLR027C	YMR059W
YLR027C	YNL244C
YLR028C	YLR373C
YLR030W	YNL326C
YLR031W	YMR124W
YLR034C	YLR056W
YLR034C	YMR215W
YLR034C	YOR069W
YLR034C	YPL076W
YLR035C	YMR167W
YLR035C	YMR206W
YLR035C	YOR261C
YLR037C	YLR183C
YLR038C	YML125C
YLR039C	YLR262C
YLR039C	YOR361C
YLR042C	YLR434C

YLR044C	YLR175W
YLR044C	YLR314C
YLR044C	YML016C
YLR044C	YML064C
YLR044C	YML115C
YLR044C	YMR117C
YLR044C	YMR246W
YLR044C	YNL127W
YLR044C	YNL135C
YLR044C	YNL183C
YLR044C	YNL189W
YLR044C	YNL244C
YLR044C	YNR031C
YLR044C	YOL139C
YLR044C	YOR089C
YLR044C	YOR212W
YLR044C	YOR260W
YLR044C	YOR269W
YLR044C	YOR351C
YLR044C	YPL031C
YLR044C	YPL082C
YLR044C	YPL203W
YLR044C	YPR110C
YLR044C	YPR178W
YLR045C	YPR086W
YLR049C	YLR191W
YLR049C	YPL161C
YLR050C	YLR288C
YLR051C	YLR119W
YLR051C	YLR288C
YLR052W	YLR424W
YLR052W	YPL235W
YLR053C	YLR285W
YLR054C	YLR257W
YLR054C	YML038C
YLR055C	YOL148C
YLR055C	YOR119C
YLR056W	YLR292C
YLR056W	YOL099C
YLR056W	YOR167C
YLR057W	YLR373C
YLR058C	YLR175W
YLR058C	YNL189W
YLR058C	YNL244C
YLR058C	YNL290W

YLR058C	YOR212W
YLR058C	YPR110C
YLR059C	YNL250W
YLR064W	YLR263W
YLR066W	YOL020W
YLR067C	YLR438C-A
YLR070C	YML064C
YLR071C	YNL236W
YLR071C	YOL051W
YLR071C	YOL135C
YLR071C	YOR119C
YLR072W	YNL307C
YLR073C	YPL084W
YLR074C	YLR106C
YLR074C	YLR196W
YLR074C	YLR397C
YLR074C	YMR303C
YLR074C	YNL002C
YLR074C	YNL030W
YLR074C	YNL038W
YLR074C	YNL110C
YLR074C	YNL175C
YLR074C	YNL182C
YLR074C	YNR001C
YLR074C	YNR053C
YLR074C	YOL041C
YLR074C	YPL013C
YLR074C	YPL093W
YLR074C	YPR016C
YLR076C	YOR362C
YLR077W	YMR047C
YLR078C	YLR268W
YLR078C	YML055W
YLR078C	YMR047C
YLR078C	YMR257C
YLR078C	YMR297W
YLR078C	YNL287W
YLR078C	YPL010W
YLR078C	YPL218W
YLR078C	YPR181C
YLR079W	YLR210W
YLR079W	YOR209C
YLR079W	YPR120C
YLR080W	YMR083W
YLR080W	YOL131W

YLR081W	YLR270W
YLR081W	YOR181W
YLR082C	YML064C
YLR082C	YMR043W
YLR082C	YMR061W
YLR082C	YNL189W
YLR083C	YLR295C
YLR083C	YML048W
YLR083C	YMR177W
YLR083C	YOR016C
YLR083C	YOR036W
YLR083C	YOR071C
YLR083C	YPL162C
YLR083C	YPL264C
YLR086W	YLR163C
YLR086W	YOL069W
YLR086W	YPR110C
YLR087C	YPR154W
YLR090W	YMR047C
YLR090W	YMR240C
YLR093C	YMR197C
YLR093C	YOR106W
YLR094C	YLR295C
YLR094C	YLR453C
YLR094C	YMR047C
YLR096W	YLR182W
YLR096W	YLR191W
YLR096W	YLR342W
YLR096W	YML006C
YLR096W	YML057W
YLR096W	YMR036C
YLR096W	YMR066W
YLR096W	YMR124W
YLR096W	YMR205C
YLR096W	YNL049C
YLR096W	YNL161W
YLR096W	YNR052C
YLR096W	YOL054W
YLR096W	YOL083W
YLR096W	YOL138C
YLR096W	YOR014W
YLR096W	YOR061W
YLR096W	YOR093C
YLR096W	YPR115W
YLR096W	YPR159W

YLR096W	YPR181C
YLR097C	YLR259C
YLR097C	YLR289W
YLR097C	YMR303C
YLR097C	YNL037C
YLR097C	YOL133W
YLR097C	YOR151C
YLR097C	YPL240C
YLR098C	YPL130W
YLR102C	YLR127C
YLR102C	YML092C
YLR102C	YMR001C
YLR102C	YMR092C
YLR102C	YNL176C
YLR102C	YOR249C
YLR103C	YLR259C
YLR103C	YNL330C
YLR103C	YOL004W
YLR104W	YLR288C
YLR105C	YMR059W
YLR105C	YNR058W
YLR105C	YPL083C
YLR106C	YML029W
YLR106C	YNL030W
YLR106C	YNL110C
YLR106C	YOL133W
YLR106C	YPL043W
YLR108C	YLR423C
YLR108C	YOR299W
YLR109W	YLR340W
YLR109W	YMR022W
YLR109W	YMR059W
YLR109W	YNL312W
YLR110C	YLR453C
YLR113W	YLR248W
YLR113W	YLR362W
YLR113W	YML127W
YLR113W	YMR172W
YLR113W	YMR276W
YLR113W	YNL167C
YLR113W	YOL123W
YLR113W	YOR039W
YLR113W	YOR061W
YLR113W	YOR198C
YLR113W	YOR208W

YLR113W YPL240C
YLR113W YPR115W
YLR113W YPR137C-A
YLR115W YLR277C
YLR115W YMR061W
YLR115W YNL222W
YLR115W YNL317W
YLR115W YPR107C
YLR116W YML046W
YLR116W YMR065W
YLR116W YMR216C
YLR116W YOR142W-A
YLR116W YPL016W
YLR116W YPL105C
YLR116W YPR137C-B
YLR117C YML049C
YLR117C YMR213W
YLR117C YMR240C
YLR117C YPR101W
YLR119W YLR288C
YLR119W YMR235C
YLR121C YLR453C
YLR124W YLR288C
YLR124W YML012W
YLR124W YML041C
YLR124W YNL092W
YLR125W YLR288C
YLR125W YMR030W
YLR127C YMR001C
YLR127C YNL172W
YLR127C YOL133W
YLR127C YOR249C
YLR128W YOR198C
YLR129W YLR222C
YLR131C YMR304W
YLR131C YNL157W
YLR131C YNL161W
YLR131C YPR086W
YLR133W YMR139W
YLR134W YLR175W
YLR134W YLR261C
YLR134W YLR447C
YLR134W YNL244C
YLR134W YOR390W
YLR135W YOR167C

YLR135W	YPL022W
YLR136C	YNL230C
YLR141W	YLR295C
YLR141W	YPL224C
YLR143W	YML023C
YLR144C	YLR191W
YLR144C	YPR154W
YLR146C	YPR069C
YLR147C	YLR275W
YLR147C	YLR430W
YLR147C	YLR433C
YLR147C	YMR088C
YLR147C	YMR125W
YLR147C	YMR240C
YLR147C	YNL016W
YLR147C	YNR072W
YLR147C	YOR159C
YLR147C	YOR308C
YLR147C	YPR014C
YLR147C	YPR082C
YLR147C	YPR178W
YLR147C	YPR182W
YLR148W	YLR396C
YLR148W	YML085C
YLR148W	YMR197C
YLR148W	YMR231W
YLR148W	YOR106W
YLR148W	YPL045W
YLR148W	YPL240C
YLR150W	YLR288C
YLR151C	YPR066W
YLR153C	YLR383W
YLR153C	YNL244C
YLR154C	YNL088W
YLR158C	YLR295C
YLR158C	YNL070W
YLR158C	YPR163C
YLR163C	YOL021C
YLR166C	YMR002W
YLR166C	YPR055W
YLR168C	YLR295C
YLR170C	YPL259C
YLR170C	YPR029C
YLR171W	YPR201W
YLR172C	YOL042W

YLR172C YOR159C
YLR174W YNR065C
YLR175W YLR197W
YLR175W YLR233C
YLR175W YLR259C
YLR175W YLR304C
YLR175W YMR047C
YLR175W YMR108W
YLR175W YMR186W
YLR175W YMR205C
YLR175W YMR290C
YLR175W YMR309C
YLR175W YNL124W
YLR175W YNL189W
YLR175W YNL307C
YLR175W YNR001C
YLR175W YOR133W
YLR175W YOR267C
YLR175W YPL012W
YLR175W YPL043W
YLR176C YLR357W
YLR176C YNL030W
YLR176C YOR304W
YLR176C YPL082C
YLR177W YPL203W
YLR180W YLR186W
YLR180W YLR288C
YLR180W YLR383W
YLR180W YLR442C
YLR180W YML057W
YLR180W YML064C
YLR180W YML126C
YLR180W YMR055C
YLR180W YMR059W
YLR180W YMR093W
YLR180W YMR106C
YLR180W YMR137C
YLR180W YMR198W
YLR180W YMR205C
YLR180W YMR284W
YLR180W YNL030W
YLR180W YNL128W
YLR180W YNL182C
YLR180W YNL230C
YLR180W YNL236W

YLR180W YOL045W
YLR180W YOL126C
YLR180W YOL128C
YLR180W YOL133W
YLR180W YOR212W
YLR180W YOR319W
YLR180W YOR326W
YLR180W YOR351C
YLR180W YPL140C
YLR180W YPL164C
YLR180W YPL236C
YLR180W YPL259C
YLR180W YPR110C
YLR180W YPR111W
YLR181C YPR173C
YLR182W YOL131W
YLR182W YOR158W
YLR182W YOR159C
YLR182W YPL204W
YLR182W YPL256C
YLR186W YLR438W
YLR186W YMR205C
YLR186W YNR001C
YLR186W YOL055C
YLR186W YPL004C
YLR190W YMR117C
YLR190W YPL031C
YLR191W YLR330W
YLR191W YML034W
YLR191W YML035C
YLR191W YML052W
YLR191W YMR026C
YLR191W YMR066W
YLR191W YMR192W
YLR191W YNL025C
YLR191W YNL047C
YLR191W YNL094W
YLR191W YNL131W
YLR191W YNL138W
YLR191W YNL152W
YLR191W YNL206C
YLR191W YNL214W
YLR191W YNR016C
YLR191W YNR064C
YLR191W YOL044W

YLR191W YOR057W
YLR191W YOR181W
YLR191W YOR229W
YLR191W YOR259C
YLR191W YOR355W
YLR191W YPL027W
YLR191W YPL049C
YLR191W YPL084W
YLR191W YPL202C
YLR191W YPR091C
YLR191W YPR157W
YLR191W YPR171W
YLR192C YML064C
YLR192C YNL244C
YLR192C YOR361C
YLR192C YPR041W
YLR196W YLR233C
YLR196W YLR449W
YLR196W YMR047C
YLR196W YNL037C
YLR196W YNL125C
YLR196W YNL230C
YLR196W YOL077C
YLR196W YOL151W
YLR196W YOR176W
YLR196W YOR267C
YLR196W YPL126W
YLR196W YPL141C
YLR196W YPL207W
YLR196W YPL259C
YLR196W YPR115W
YLR196W YPR191W
YLR197W YMR116C
YLR197W YMR236W
YLR197W YNL061W
YLR197W YNL088W
YLR197W YOL115W
YLR197W YOR303W
YLR197W YPL043W
YLR198C YPL094C
YLR199C YMR263W
YLR200W YLR212C
YLR200W YLR288C
YLR200W YML094W
YLR200W YMR052W

YLR201C	YPR086W
YLR202C	YLR288C
YLR203C	YLR447C
YLR203C	YPL019C
YLR204W	YMR299C
YLR207W	YLR450W
YLR207W	YML075C
YLR207W	YNL095C
YLR207W	YOL013C
YLR207W	YPL022W
YLR208W	YML012W
YLR208W	YML130C
YLR208W	YNL307C
YLR208W	YPL085W
YLR208W	YPR107C
YLR209C	YNL189W
YLR211C	YLR446W
YLR211C	YNL189W
YLR212C	YML094W
YLR212C	YNL126W
YLR212C	YNL153C
YLR214W	YMR026C
YLR214W	YMR054W
YLR215C	YLR295C
YLR215C	YLR386W
YLR215C	YLR447C
YLR215C	YNL116W
YLR216C	YMR186W
YLR216C	YMR303C
YLR216C	YNL278W
YLR216C	YNL330C
YLR216C	YNR032W
YLR216C	YOR154W
YLR216C	YOR220W
YLR216C	YPL240C
YLR219W	YLR427W
YLR220W	YOR085W
YLR221C	YMR187C
YLR221C	YPR115W
YLR222C	YLR259C
YLR222C	YMR105C
YLR222C	YMR319C
YLR222C	YNL055C
YLR222C	YNL138W
YLR222C	YNL239W

YLR222C	YNR041C
YLR222C	YPL265W
YLR223C	YLR362W
YLR223C	YOR247W
YLR224W	YOL133W
YLR224W	YOR057W
YLR225C	YNL141W
YLR226W	YMR125W
YLR226W	YPR161C
YLR227C	YML133C
YLR227C	YOR089C
YLR229C	YLR259C
YLR229C	YMR303C
YLR229C	YNL271C
YLR229C	YNL298W
YLR229C	YOL069W
YLR229C	YOL113W
YLR229C	YOR127W
YLR229C	YOR151C
YLR229C	YPL161C
YLR229C	YPL242C
YLR233C	YLR318W
YLR233C	YML038C
YLR233C	YNL064C
YLR233C	YNL132W
YLR233C	YNL229C
YLR233C	YOR201C
YLR233C	YOR319W
YLR233C	YPL012W
YLR234W	YMR190C
YLR238W	YML085C
YLR238W	YML123C
YLR238W	YMR029C
YLR238W	YMR319C
YLR238W	YPR110C
YLR239C	YLR288C
YLR239C	YNL030W
YLR239C	YPL073C
YLR240W	YPL120W
YLR241W	YLR372W
YLR241W	YMR243C
YLR241W	YNR060W
YLR241W	YOR307C
YLR245C	YLR291C
YLR245C	YML064C

YLR245C	YNL189W
YLR245C	YPL070W
YLR246W	YOL110W
YLR247C	YMR066W
YLR247C	YNL030W
YLR247C	YOR028C
YLR248W	YMR319C
YLR248W	YPL078C
YLR249W	YOR264W
YLR249W	YPL082C
YLR249W	YPR180W
YLR251W	YLR453C
YLR252W	YLR288C
YLR254C	YLR288C
YLR254C	YLR314C
YLR254C	YNL103W
YLR254C	YOR269W
YLR258W	YLR273C
YLR258W	YOR026W
YLR258W	YOR178C
YLR258W	YPL031C
YLR258W	YPL204W
YLR258W	YPL219W
YLR259C	YLR291C
YLR259C	YLR320W
YLR259C	YLR352W
YLR259C	YLR383W
YLR259C	YLR403W
YLR259C	YML016C
YLR259C	YML057W
YLR259C	YML064C
YLR259C	YML112W
YLR259C	YMR001C
YLR259C	YMR022W
YLR259C	YMR036C
YLR259C	YMR049C
YLR259C	YMR055C
YLR259C	YMR106C
YLR259C	YMR138W
YLR259C	YMR205C
YLR259C	YNL006W
YLR259C	YNL061W
YLR259C	YNL094W
YLR259C	YNL106C
YLR259C	YNL128W

YLR259C	YNL135C
YLR259C	YNL230C
YLR259C	YNL244C
YLR259C	YNL317W
YLR259C	YNR047W
YLR259C	YOL062C
YLR259C	YOL094C
YLR259C	YOL108C
YLR259C	YOL113W
YLR259C	YOL126C
YLR259C	YOL128C
YLR259C	YOR005C
YLR259C	YOR020C
YLR259C	YOR125C
YLR259C	YOR174W
YLR259C	YOR181W
YLR259C	YOR212W
YLR259C	YOR233W
YLR259C	YOR269W
YLR259C	YOR351C
YLR259C	YOR386W
YLR259C	YPL026C
YLR259C	YPL135W
YLR259C	YPL149W
YLR259C	YPL151C
YLR259C	YPL164C
YLR259C	YPL203W
YLR259C	YPL204W
YLR259C	YPL262W
YLR259C	YPR015C
YLR259C	YPR054W
YLR259C	YPR111W
YLR259C	YPR178W
YLR261C	YNL025C
YLR262C	YLR304C
YLR262C	YMR235C
YLR262C	YNL263C
YLR262C	YOL055C
YLR264W	YOL149W
YLR265C	YNL044W
YLR266C	YNL113W
YLR267W	YOL133W
YLR268W	YNL237W
YLR268W	YNL287W
YLR268W	YOR037W

YLR268W	YOR075W
YLR268W	YOR311C
YLR268W	YPL010W
YLR268W	YPL218W
YLR271W	YMR106C
YLR273C	YLR447C
YLR273C	YNL025C
YLR274W	YOR080W
YLR274W	YPR019W
YLR275W	YLR298C
YLR275W	YMR125W
YLR275W	YMR213W
YLR275W	YNL147W
YLR275W	YOR159C
YLR275W	YPL178W
YLR275W	YPR182W
YLR276C	YMR308C
YLR276C	YPL043W
YLR277C	YLR437C
YLR277C	YMR061W
YLR277C	YNL317W
YLR277C	YOR250C
YLR277C	YPR107C
YLR281C	YLR438C-A
YLR284C	YML064C
YLR284C	YNL189W
YLR284C	YOR180C
YLR284C	YPL070W
YLR287C	YLR340W
YLR287C	YNL092W
YLR288C	YLR290C
YLR288C	YMR159C
YLR288C	YMR295C
YLR288C	YNL013C
YLR288C	YNL064C
YLR288C	YNL110C
YLR288C	YNL131W
YLR288C	YNL158W
YLR288C	YNL190W
YLR288C	YNL301C
YLR288C	YOL012C
YLR288C	YOL046C
YLR288C	YOL055C
YLR288C	YOL093W
YLR288C	YOL131W

YLR288C	YOL139C
YLR288C	YOR087W
YLR288C	YOR104W
YLR288C	YOR368W
YLR288C	YPL194W
YLR288C	YPL258C
YLR288C	YPR018W
YLR289W	YOL133W
YLR289W	YPL022W
YLR289W	YPL170W
YLR291C	YLR386W
YLR291C	YLR423C
YLR291C	YML085C
YLR291C	YML124C
YLR291C	YMR052W
YLR291C	YMR058W
YLR291C	YMR108W
YLR291C	YMR186W
YLR291C	YMR246W
YLR291C	YNL064C
YLR291C	YNL265C
YLR291C	YOL130W
YLR291C	YOR260W
YLR291C	YOR284W
YLR291C	YOR380W
YLR291C	YPL070W
YLR291C	YPL124W
YLR291C	YPL237W
YLR291C	YPL240C
YLR292C	YML008C
YLR292C	YML123C
YLR292C	YNL003C
YLR292C	YOR254C
YLR292C	YPL094C
YLR292C	YPR048W
YLR293C	YLR335W
YLR293C	YLR347C
YLR293C	YMR235C
YLR293C	YMR308C
YLR293C	YNL071W
YLR293C	YNL189W
YLR293C	YOL021C
YLR293C	YOR160W
YLR293C	YOR185C
YLR294C	YMR047C

YLR294C	YOR190W
YLR294C	YOR348C
YLR294C	YPR086W
YLR295C	YLR455W
YLR295C	YLR460C
YLR295C	YML007W
YLR295C	YML034W
YLR295C	YML058W
YLR295C	YML079W
YLR295C	YML121W
YLR295C	YMR003W
YLR295C	YMR039C
YLR295C	YMR090W
YLR295C	YMR138W
YLR295C	YMR141C
YLR295C	YMR144W
YLR295C	YMR152W
YLR295C	YMR153W
YLR295C	YMR177W
YLR295C	YMR181C
YLR295C	YMR238W
YLR295C	YNL009W
YLR295C	YNL066W
YLR295C	YNL194C
YLR295C	YNL205C
YLR295C	YNL263C
YLR295C	YNL324W
YLR295C	YNL328C
YLR295C	YOL008W
YLR295C	YOL050C
YLR295C	YOR145C
YLR295C	YOR184W
YLR295C	YOR324C
YLR295C	YPL124W
YLR295C	YPR086W
YLR295C	YPR118W
YLR295C	YPR163C
YLR295C	YPR182W
YLR297W	YPR156C
YLR298C	YML046W
YLR298C	YMR125W
YLR299W	YOL137W
YLR300W	YMR049C
YLR300W	YOL126C
YLR301W	YLR453C

YLR301W	YNL178W
YLR303W	YNL189W
YLR304C	YML064C
YLR304C	YMR049C
YLR304C	YMR059W
YLR304C	YNL135C
YLR304C	YNL189W
YLR304C	YNL192W
YLR304C	YOL006C
YLR304C	YOL094C
YLR304C	YOR005C
YLR304C	YOR089C
YLR304C	YPL204W
YLR304C	YPR110C
YLR305C	YMR212C
YLR305C	YOR047C
YLR305C	YOR355W
YLR306W	YPL003W
YLR310C	YMR139W
YLR310C	YMR156C
YLR310C	YMR189W
YLR310C	YMR275C
YLR310C	YNL025C
YLR310C	YNL098C
YLR310C	YNL116W
YLR310C	YNL229C
YLR310C	YNL231C
YLR310C	YNL307C
YLR310C	YNR037C
YLR310C	YNR064C
YLR310C	YOR013W
YLR310C	YOR101W
YLR310C	YOR156C
YLR310C	YPL038W
YLR310C	YPL049C
YLR310C	YPL082C
YLR310C	YPL100W
YLR310C	YPL203W
YLR310C	YPL204W
YLR310C	YPL240C
YLR310C	YPR023C
YLR310C	YPR108W
YLR310C	YPR171W
YLR310C	YPR175W
YLR312C	YMR047C

YLR312C	YOL088C
YLR313C	YLR362W
YLR313C	YOR231W
YLR313C	YPL140C
YLR314C	YLR355C
YLR314C	YMR055C
YLR314C	YMR186W
YLR314C	YNL166C
YLR315W	YLR453C
YLR315W	YPR105C
YLR317W	YNL140C
YLR319C	YLR362W
YLR319C	YMR186W
YLR319C	YNL271C
YLR320W	YNR001C
YLR321C	YLR345W
YLR321C	YML064C
YLR321C	YPL022W
YLR321C	YPL070W
YLR322W	YNL025C
YLR324W	YMR153W
YLR324W	YNL189W
YLR324W	YPR028W
YLR325C	YOR327C
YLR326W	YNL323W
YLR326W	YPL151C
YLR327C	YMR312W
YLR327C	YPL042C
YLR328W	YLR332W
YLR328W	YLR347C
YLR328W	YLR438W
YLR328W	YML064C
YLR328W	YNL189W
YLR328W	YPL259C
YLR329W	YPL259C
YLR332W	YLR371W
YLR332W	YOL109W
YLR334C	YOR264W
YLR335W	YLR347C
YLR335W	YMR047C
YLR335W	YNL189W
YLR335W	YOR020C
YLR335W	YOR133W
YLR337C	YML001W
YLR337C	YMR032W

YLR337C	YMR109W
YLR337C	YNL020C
YLR337C	YOR181W
YLR339C	YLR461W
YLR340W	YNL030W
YLR340W	YNL064C
YLR340W	YPR016C
YLR342W	YML130C
YLR342W	YNL020C
YLR342W	YNL025C
YLR342W	YNL298W
YLR342W	YNL307C
YLR342W	YPL094C
YLR342W	YPR165W
YLR343W	YML019W
YLR343W	YMR215W
YLR343W	YPL076W
YLR345W	YML092C
YLR345W	YNL189W
YLR347C	YLR377C
YLR347C	YML007W
YLR347C	YMR001C
YLR347C	YMR047C
YLR347C	YMR125W
YLR347C	YMR159C
YLR347C	YMR273C
YLR347C	YMR308C
YLR347C	YNL044W
YLR347C	YNL189W
YLR347C	YNL323W
YLR347C	YNL331C
YLR347C	YOL143C
YLR347C	YOR098C
YLR347C	YOR185C
YLR347C	YOR304W
YLR347C	YPL089C
YLR347C	YPL111W
YLR347C	YPL124W
YLR347C	YPL153C
YLR347C	YPL178W
YLR347C	YPR018W
YLR347C	YPR025C
YLR350W	YMR296C
YLR351C	YOR327C
YLR352W	YLR447C

YLR352W	YOL133W
YLR353W	YMR047C
YLR354C	YNL053W
YLR354C	YNL189W
YLR354C	YOR061W
YLR354C	YPR110C
YLR355C	YLR442C
YLR355C	YNL180C
YLR355C	YNL244C
YLR355C	YOL062C
YLR355C	YOR061W
YLR355C	YOR174W
YLR355C	YPL031C
YLR355C	YPL149W
YLR355C	YPL203W
YLR355C	YPR110C
YLR358C	YMR228W
YLR358C	YNL025C
YLR358C	YNL145W
YLR359W	YOR098C
YLR359W	YOR361C
YLR362W	YMR186W
YLR362W	YOR089C
YLR362W	YPL240C
YLR363C	YMR080C
YLR366W	YOR312C
YLR368W	YMR035W
YLR368W	YMR048W
YLR368W	YPL022W
YLR368W	YPR078C
YLR368W	YPR093C
YLR369W	YMR049C
YLR370C	YNR035C
YLR370C	YOR361C
YLR371W	YNL127W
YLR371W	YOL139C
YLR371W	YOR008C
YLR371W	YPR165W
YLR372W	YLR378C
YLR372W	YML048W
YLR372W	YML075C
YLR372W	YML123C
YLR372W	YMR058W
YLR372W	YMR149W
YLR372W	YMR215W

YLR372W	YNL048W
YLR372W	YNL101W
YLR372W	YNL238W
YLR372W	YOL132W
YLR372W	YOL156W
YLR372W	YPL076W
YLR372W	YPL264C
YLR372W	YPR156C
YLR372W	YPR194C
YLR372W	YPR198W
YLR373C	YMR048W
YLR373C	YMR140W
YLR373C	YMR154C
YLR373C	YNR041C
YLR373C	YOR028C
YLR373C	YOR317W
YLR373C	YOR368W
YLR373C	YPL060W
YLR373C	YPL107W
YLR373C	YPR025C
YLR373C	YPR065W
YLR377C	YML064C
YLR377C	YML121W
YLR377C	YNL189W
YLR377C	YPL169C
YLR378C	YMR297W
YLR378C	YPL253C
YLR383W	YLR438W
YLR383W	YML028W
YLR383W	YML056C
YLR383W	YMR186W
YLR383W	YNL030W
YLR383W	YNL064C
YLR383W	YNL189W
YLR383W	YOL055C
YLR383W	YOR230W
YLR383W	YOR375C
YLR383W	YPL028W
YLR383W	YPL061W
YLR383W	YPL262W
YLR383W	YPR165W
YLR384C	YMR312W
YLR384C	YOR151C
YLR384C	YPL086C
YLR384C	YPL101W

YLR384C	YPR187W
YLR385C	YLR424W
YLR385C	YNL107W
YLR385C	YOL012C
YLR385C	YPL235W
YLR386W	YML092C
YLR386W	YNL041C
YLR386W	YNR016C
YLR386W	YPL151C
YLR386W	YPL256C
YLR387C	YOL056W
YLR390W-A	YOL132W
YLR390W-A	YPL149W
YLR392C	YPL255W
YLR393W	YMR279C
YLR394W	YMR224C
YLR396C	YMR231W
YLR396C	YMR291W
YLR396C	YOR106W
YLR396C	YPL045W
YLR397C	YMR047C
YLR397C	YPR017C
YLR398C	YOR076C
YLR399C	YNL107W
YLR399C	YNL244C
YLR399C	YOL012C
YLR399C	YOR338W
YLR401C	YMR047C
YLR403W	YOR025W
YLR403W	YOR370C
YLR411W	YNL221C
YLR412W	YOL031C
YLR416C	YPR135W
YLR417W	YLR452C
YLR417W	YMR077C
YLR417W	YPL002C
YLR418C	YML010W
YLR418C	YOL145C
YLR418C	YOR123C
YLR418C	YOR151C
YLR418C	YPL129W
YLR418C	YPR086W
YLR421C	YOL133W
YLR422W	YOL108C
YLR423C	YML041C

YLR423C	YML064C
YLR423C	YMR017W
YLR423C	YMR025W
YLR423C	YMR032W
YLR423C	YMR068W
YLR423C	YMR124W
YLR423C	YMR204C
YLR423C	YMR213W
YLR423C	YNL078W
YLR423C	YNL090W
YLR423C	YNL182C
YLR423C	YNL230C
YLR423C	YOR158W
YLR423C	YOR164C
YLR423C	YOR232W
YLR423C	YPL077C
YLR423C	YPL124W
YLR423C	YPL159C
YLR423C	YPL174C
YLR423C	YPR105C
YLR423C	YPR165W
YLR423C	YPR179C
YLR423C	YPR185W
YLR424W	YOR023C
YLR424W	YOR158W
YLR424W	YPR182W
YLR425W	YOR053W
YLR426W	YLR447C
YLR426W	YLR453C
YLR427W	YLR432W
YLR427W	YML056C
YLR427W	YMR290C
YLR427W	YMR319C
YLR427W	YNL132W
YLR427W	YNL308C
YLR427W	YOL041C
YLR427W	YOL055C
YLR427W	YOL139C
YLR427W	YOR206W
YLR427W	YPL012W
YLR427W	YPL043W
YLR428C	YPL242C
YLR429W	YNL094W
YLR429W	YNR035C
YLR429W	YPL032C

YLR430W	YMR125W
YLR430W	YPL022W
YLR432W	YNL175C
YLR432W	YNL230C
YLR432W	YOL115W
YLR433C	YLR453C
YLR433C	YMR205C
YLR433C	YMR246W
YLR433C	YNL027W
YLR433C	YNL047C
YLR433C	YNL064C
YLR433C	YNL085W
YLR433C	YOR324C
YLR434C	YMR047C
YLR435W	YML107C
YLR435W	YNL221C
YLR436C	YMR304W
YLR438C-A	YMR142C
YLR438C-A	YMR268C
YLR438C-A	YNL147W
YLR438C-A	YOL149W
YLR438C-A	YOR096W
YLR438C-A	YPL084W
YLR438C-A	YPR178W
YLR438W	YMR059W
YLR438W	YNL244C
YLR438W	YPL022W
YLR438W	YPL150W
YLR438W	YPL204W
YLR439W	YNL185C
YLR439W	YNL284C
YLR440C	YNL066W
YLR440C	YNL258C
YLR441C	YOR119C
YLR442C	YML109W
YLR442C	YMR223W
YLR442C	YMR226C
YLR442C	YMR307W
YLR442C	YMR318C
YLR442C	YNL030W
YLR442C	YNL064C
YLR442C	YNL216W
YLR442C	YNR001C
YLR446W	YOR028C
YLR446W	YOR047C

YLR447C	YLR465C
YLR447C	YML022W
YLR447C	YML041C
YLR447C	YMR054W
YLR447C	YMR071C
YLR447C	YMR127C
YLR447C	YMR154C
YLR447C	YNL028W
YLR447C	YNL134C
YLR447C	YNL161W
YLR447C	YNL169C
YLR447C	YNL175C
YLR447C	YNL182C
YLR447C	YNL196C
YLR447C	YNL323W
YLR447C	YNR001C
YLR447C	YOL011W
YLR447C	YOL036W
YLR447C	YOR028C
YLR447C	YOR036W
YLR447C	YOR047C
YLR447C	YOR053W
YLR447C	YOR181W
YLR447C	YOR190W
YLR447C	YOR231W
YLR447C	YOR239W
YLR447C	YOR260W
YLR447C	YOR270C
YLR447C	YOR315W
YLR447C	YOR332W
YLR447C	YOR374W
YLR447C	YOR379C
YLR447C	YPL003W
YLR447C	YPL050C
YLR447C	YPL152W
YLR447C	YPR006C
YLR447C	YPR078C
YLR449W	YLR453C
YLR449W	YMR049C
YLR449W	YNL061W
YLR449W	YOR267C
YLR450W	YMR022W
YLR450W	YOL013C
YLR450W	YOL113W
YLR450W	YPR154W

YLR451W	YNL118C
YLR452C	YLR457C
YLR452C	YML038C
YLR452C	YMR004W
YLR452C	YOL018C
YLR452C	YOR036W
YLR453C	YML027W
YLR453C	YML038C
YLR453C	YMR052W
YLR453C	YMR147W
YLR453C	YNL013C
YLR453C	YNL072W
YLR453C	YNL133C
YLR453C	YNL216W
YLR453C	YNL237W
YLR453C	YNL260C
YLR453C	YNR009W
YLR453C	YNR013C
YLR453C	YNR050C
YLR453C	YNR066C
YLR453C	YNR071C
YLR453C	YOL006C
YLR453C	YOR001W
YLR453C	YOR195W
YLR453C	YOR198C
YLR453C	YOR206W
YLR453C	YOR220W
YLR453C	YOR230W
YLR453C	YOR314W
YLR453C	YPL034W
YLR453C	YPL057C
YLR453C	YPL098C
YLR453C	YPL154C
YLR453C	YPR110C
YLR453C	YPR144C
YLR454W	YMR065W
YLR455W	YPR086W
YLR456W	YNR053C
YLR456W	YPR172W
YLR457C	YML006C
YLR457C	YNL258C
YLR457C	YPR086W
YLR459W	YNL301C
YLR465C	YML035C
YLR465C	YPR071W

YML001W YNL263C
YML006C YNL025C
YML006C YNL183C
YML006C YOR267C
YML006C YPL204W
YML007W YMR047C
YML007W YMR308C
YML007W YOR244W
YML008C YMR059W
YML008C YOR097C
YML008C YPL020C
YML008C YPR113W
YML009C YNL284C
YML010W YNL201C
YML010W YOL145C
YML010W YOR123C
YML010W YPL203W
YML010W YPR041W
YML011C YOR047C
YML011C YOR128C
YML012W YMR054W
YML012W YPR181C
YML014W YML099C
YML014W YNR046W
YML015C YML098W
YML015C YMR077C
YML015C YMR236W
YML016C YMR108W
YML016C YMR311C
YML016C YOR054C
YML016C YOR308C
YML016C YOR329C
YML016C YPL074W
YML016C YPR005C
YML019W YNL318C
YML019W YPR086W
YML020W YMR106C
YML022W YMR106C
YML023C YOL128C
YML025C YNL185C
YML028W YML102W
YML028W YMR022W
YML028W YNL189W
YML028W YNL244C
YML028W YOR031W

YML028W	YOR089C
YML028W	YOR174W
YML028W	YPL106C
YML028W	YPL208W
YML029W	YMR066W
YML029W	YNL329C
YML031W	YMR153W
YML031W	YOR284W
YML031W	YPR021C
YML032C	YNL312W
YML032C	YOL055C
YML032C	YPL153C
YML032C	YPL258C
YML036W	YMR319C
YML037C	YNL092W
YML038C	YNR066C
YML041C	YOL012C
YML041C	YOR299W
YML041C	YPL235W
YML042W	YMR038C
YML042W	YNL046W
YML042W	YNL189W
YML042W	YOL075C
YML046W	YPR182W
YML048W	YML067C
YML048W	YML123C
YML048W	YMR011W
YML048W	YMR058W
YML048W	YMR149W
YML048W	YMR215W
YML048W	YNL101W
YML048W	YNL300W
YML048W	YOL132W
YML048W	YOL156W
YML048W	YPL076W
YML048W	YPL264C
YML048W	YPL274W
YML048W	YPR156C
YML048W	YPR198W
YML049C	YMR125W
YML049C	YMR213W
YML049C	YMR240C
YML049C	YMR288W
YML049C	YOR159C
YML049C	YOR319W

YML049C	YPL178W
YML049C	YPR182W
YML051W	YPL248C
YML054C	YNR017W
YML054C	YPL063W
YML055W	YOL016C
YML056C	YNL230C
YML056C	YOL115W
YML057W	YMR066W
YML057W	YNL037C
YML057W	YNL047C
YML057W	YNL290W
YML057W	YPL235W
YML057W	YPR108W
YML057W	YPR115W
YML058W	YMR083W
YML058W	YMR205C
YML058W	YNL116W
YML058W	YPR160W
YML059C	YMR308C
YML060W	YOL010W
YML061C	YMR063W
YML062C	YNL097C
YML062C	YNL139C
YML062C	YNL189W
YML064C	YML078W
YML064C	YML085C
YML064C	YML124C
YML064C	YMR012W
YML064C	YMR055C
YML064C	YMR106C
YML064C	YMR153W
YML064C	YMR159C
YML064C	YMR186W
YML064C	YMR205C
YML064C	YMR214W
YML064C	YMR226C
YML064C	YMR246W
YML064C	YMR290C
YML064C	YNL044W
YML064C	YNL064C
YML064C	YNL071W
YML064C	YNL218W
YML064C	YNL239W
YML064C	YNL331C

YML064C YNL333W
YML064C YNR001C
YML064C YNR012W
YML064C YOL055C
YML064C YOL139C
YML064C YOR020C
YML064C YOR128C
YML064C YOR133W
YML064C YOR229W
YML064C YOR232W
YML064C YOR272W
YML064C YOR284W
YML064C YOR374W
YML064C YOR380W
YML064C YPL049C
YML064C YPL070W
YML064C YPL111W
YML064C YPL124W
YML064C YPL192C
YML064C YPL218W
YML064C YPL235W
YML064C YPL242C
YML064C YPL255W
YML064C YPL258C
YML064C YPR182W
YML064C YPR191W
YML064C YPR193C
YML065W YML109W
YML065W YNL261W
YML065W YPL022W
YML065W YPR162C
YML067C YML130C
YML067C YPL234C
YML069W YNL102W
YML069W YOL054W
YML069W YOR292C
YML070W YOR266W
YML071C YNL265C
YML074C YOR080W
YML074C YOR267C
YML074C YPL204W
YML074C YPR115W
YML075C YNR009W
YML075C YOL013C
YML075C YOR102W

YML075C YPL264C
YML081C-A YPR020W
YML085C YML112W
YML085C YML115C
YML085C YMR001C
YML085C YMR106C
YML085C YMR284W
YML085C YNL090W
YML085C YNL113W
YML085C YNL148C
YML085C YNL161W
YML085C YNL182C
YML085C YNL223W
YML085C YNL290W
YML085C YNL311C
YML085C YOL086C
YML085C YOL087C
YML085C YOL115W
YML085C YOL126C
YML085C YOL133W
YML085C YOR181W
YML085C YOR229W
YML085C YOR319W
YML085C YPL140C
YML085C YPL150W
YML085C YPR110C
YML085C YPR111W
YML091C YML111W
YML091C YMR036C
YML091C YNL118C
YML091C YOR119C
YML091C YPL204W
YML092C YMR047C
YML092C YMR308C
YML092C YMR314W
YML092C YOL038W
YML092C YOL082W
YML092C YOR020C
YML092C YOR157C
YML092C YPL144W
YML092C YPL255W
YML092C YPR103W
YML092C YPR126C
YML095C YMR201C
YML095C YNR001C

YML095C YOL055C
YML095C YOL090W
YML095C YOR260W
YML095C YPL022W
YML095C YPL262W
YML098W YMR005W
YML098W YMR227C
YML098W YMR236W
YML098W YOR174W
YML098W YPL011C
YML099C YMR042W
YML099C YMR043W
YML099C YNL118C
YML100W YMR139W
YML102W YPR018W
YML103C YMR129W
YML104C YPR099C
YML104C YPR154W
YML105C YPR086W
YML105C YPR088C
YML108W YPR086W
YML109W YMR109W
YML109W YMR273C
YML109W YNL271C
YML109W YNL298W
YML109W YOR362C
YML109W YPL115C
YML109W YPR165W
YML111W YMR229C
YML111W YOR201C
YML111W YPL043W
YML111W YPL204W
YML112W YNL118C
YML112W YPL235W
YML115C YPL050C
YML117W YNL016W
YML117W YPL178W
YML117W YPR106W
YML121W YNL113W
YML121W YOL144W
YML123C YNL061W
YML123C YNL116W
YML123C YNR041C
YML123C YNR075W
YML123C YOR016C

YML123C	YOR181W
YML123C	YOR254C
YML123C	YOR307C
YML123C	YPR156C
YML124C	YMR106C
YML124C	YMR137C
YML124C	YNL148C
YML124C	YNL311C
YML124C	YOL115W
YML124C	YOL133W
YML124C	YOR181W
YML124C	YPR110C
YML124C	YPR111W
YML125C	YMR153W
YML126C	YMR059W
YML126C	YNL119W
YML129C	YOR110W
YMR001C	YMR012W
YMR001C	YMR076C
YMR001C	YNL092W
YMR001C	YNL172W
YMR001C	YNL175C
YMR001C	YNL189W
YMR001C	YNL225C
YMR001C	YOR117W
YMR001C	YOR259C
YMR001C	YPL153C
YMR001C	YPL154C
YMR001C	YPR007C
YMR002W	YOR008C
YMR003W	YPR141C
YMR009W	YPL222W
YMR012W	YMR059W
YMR012W	YMR106C
YMR012W	YMR198W
YMR012W	YOL006C
YMR012W	YOR109W
YMR012W	YPR015C
YMR014W	YOL133W
YMR014W	YOR264W
YMR017W	YOR036W
YMR017W	YOR327C
YMR017W	YPL232W
YMR019W	YOL004W
YMR020W	YOR075W

YMR020W	YPL244C
YMR021C	YNL030W
YMR022W	YNR031C
YMR024W	YNL185C
YMR024W	YNL284C
YMR025W	YNR052C
YMR026C	YOL044W
YMR032W	YMR045C
YMR032W	YMR109W
YMR032W	YMR162C
YMR032W	YNL078W
YMR032W	YNL094W
YMR032W	YNL152W
YMR032W	YNL199C
YMR032W	YNL271C
YMR032W	YNL284C-B
YMR032W	YOL036W
YMR032W	YOL070C
YMR032W	YOL093W
YMR032W	YOR098C
YMR032W	YOR181W
YMR032W	YOR191W
YMR032W	YOR264W
YMR032W	YOR324C
YMR032W	YPL059W
YMR032W	YPL084W
YMR032W	YPL115C
YMR032W	YPL140C
YMR032W	YPL158C
YMR032W	YPR137C-B
YMR033W	YOR290C
YMR033W	YPL016W
YMR036C	YOR061W
YMR036C	YPL204W
YMR036C	YPR120C
YMR036C	YPR191W
YMR041C	YPL131W
YMR042W	YMR043W
YMR043W	YNL068C
YMR043W	YOR087W
YMR047C	YMR152W
YMR047C	YMR157C
YMR047C	YMR206W
YMR047C	YMR294W
YMR047C	YMR307W

YMR047C	YMR308C
YMR047C	YNL078W
YMR047C	YNL189W
YMR047C	YNL194C
YMR047C	YNL204C
YMR047C	YNL300W
YMR047C	YNL319W
YMR047C	YNR007C
YMR047C	YNR040W
YMR047C	YNR061C
YMR047C	YNR074C
YMR047C	YOL111C
YMR047C	YOL123W
YMR047C	YOL144W
YMR047C	YOL146W
YMR047C	YOL149W
YMR047C	YOL154W
YMR047C	YOR020C
YMR047C	YOR060C
YMR047C	YOR062C
YMR047C	YOR064C
YMR047C	YOR102W
YMR047C	YOR104W
YMR047C	YOR112W
YMR047C	YOR121C
YMR047C	YOR157C
YMR047C	YOR160W
YMR047C	YOR213C
YMR047C	YOR251C
YMR047C	YOR286W
YMR047C	YOR289W
YMR047C	YOR302W
YMR047C	YOR314W
YMR047C	YOR329C
YMR047C	YOR344C
YMR047C	YOR362C
YMR047C	YPL013C
YMR047C	YPL083C
YMR047C	YPL125W
YMR047C	YPR119W
YMR047C	YPR172W
YMR047C	YPR181C
YMR048W	YMR159C
YMR048W	YMR308C
YMR048W	YNL273W

YMR048W	YOL131W
YMR049C	YMR093W
YMR049C	YMR100W
YMR049C	YMR290C
YMR049C	YNL002C
YMR049C	YNL061W
YMR049C	YNL110C
YMR049C	YNL230C
YMR049C	YOL077C
YMR049C	YOR061W
YMR049C	YOR206W
YMR049C	YOR233W
YMR049C	YOR267C
YMR049C	YOR272W
YMR049C	YOR361C
YMR049C	YPL042C
YMR049C	YPL043W
YMR049C	YPL093W
YMR049C	YPL141C
YMR049C	YPL214C
YMR049C	YPL258C
YMR049C	YPR016C
YMR049C	YPR115W
YMR052W	YNL127W
YMR053C	YOL004W
YMR054W	YMR243C
YMR054W	YOR332W
YMR054W	YPR036W
YMR055C	YNL064C
YMR055C	YPL040C
YMR056C	YNR056C
YMR058W	YOL003C
YMR058W	YOR016C
YMR058W	YOR161C
YMR058W	YOR307C
YMR058W	YPL094C
YMR058W	YPL149W
YMR058W	YPL227C
YMR058W	YPL234C
YMR058W	YPL264C
YMR058W	YPL274W
YMR059W	YMR266W
YMR059W	YNL014W
YMR059W	YNL030W
YMR059W	YNL135C

YMR059W	YNR001C
YMR059W	YNR008W
YMR059W	YOL055C
YMR059W	YOL139C
YMR059W	YOR230W
YMR059W	YOR332W
YMR059W	YOR374W
YMR059W	YPL004C
YMR059W	YPL037C
YMR059W	YPL061W
YMR059W	YPL083C
YMR061W	YNL030W
YMR061W	YNL317W
YMR061W	YOL123W
YMR061W	YOR250C
YMR064W	YOL123W
YMR065W	YNL084C
YMR066W	YMR106C
YMR066W	YMR139W
YMR066W	YNL183C
YMR066W	YOR272W
YMR066W	YOR351C
YMR068W	YNL006W
YMR068W	YNL047C
YMR068W	YOR014W
YMR072W	YOL004W
YMR075W	YNL330C
YMR075W	YOL004W
YMR075W	YOR096W
YMR077C	YPR173C
YMR078C	YNL262W
YMR078C	YNL290W
YMR078C	YOL094C
YMR078C	YPR175W
YMR080C	YNL016W
YMR080C	YNL112W
YMR080C	YNL118C
YMR080C	YOL123W
YMR080C	YOR076C
YMR080C	YPL204W
YMR087W	YNL135C
YMR088C	YPL050C
YMR092C	YNL138W
YMR092C	YPL263C
YMR094W	YMR168C

YMR094W	YOR359W
YMR094W	YPL240C
YMR095C	YMR096W
YMR095C	YNL333W
YMR096W	YMR322C
YMR096W	YNL333W
YMR096W	YOR098C
YMR097C	YPL154C
YMR102C	YNL202W
YMR102C	YNL218W
YMR102C	YOR174W
YMR104C	YOL082W
YMR104C	YOL139C
YMR104C	YOR154W
YMR105C	YMR199W
YMR105C	YNL094W
YMR105C	YOR212W
YMR105C	YPR015C
YMR106C	YMR108W
YMR106C	YMR205C
YMR106C	YMR214W
YMR106C	YMR226C
YMR106C	YMR284W
YMR106C	YMR287C
YMR106C	YNL006W
YMR106C	YNL007C
YMR106C	YNL037C
YMR106C	YNL055C
YMR106C	YNL064C
YMR106C	YNR016C
YMR106C	YNR053C
YMR106C	YOL055C
YMR106C	YOL078W
YMR106C	YOR027W
YMR106C	YOR110W
YMR106C	YOR142W
YMR106C	YOR151C
YMR106C	YOR302W
YMR106C	YOR374W
YMR106C	YPL031C
YMR106C	YPL061W
YMR106C	YPL140C
YMR106C	YPL235W
YMR106C	YPL240C
YMR106C	YPR003C

YMR106C	YPR088C
YMR106C	YPR160W
YMR106C	YPR167C
YMR106C	YPR175W
YMR108W	YMR116C
YMR108W	YMR117C
YMR108W	YNL128W
YMR108W	YNR031C
YMR108W	YOL062C
YMR108W	YOL126C
YMR108W	YOR174W
YMR108W	YOR351C
YMR108W	YPL026C
YMR108W	YPL140C
YMR109W	YMR280C
YMR109W	YMR299C
YMR109W	YNL025C
YMR109W	YNL094W
YMR109W	YNL138W
YMR109W	YNL206C
YMR109W	YNL271C
YMR109W	YNL298W
YMR109W	YNL307C
YMR109W	YNR007C
YMR109W	YNR016C
YMR109W	YNR059W
YMR109W	YOL028C
YMR109W	YOL070C
YMR109W	YOL113W
YMR109W	YOR047C
YMR109W	YOR181W
YMR109W	YOR227W
YMR109W	YOR247W
YMR109W	YOR389W
YMR109W	YPL038W
YMR109W	YPL277C
YMR109W	YPR159W
YMR112C	YOL135C
YMR116C	YNL308C
YMR116C	YOR056C
YMR116C	YOR116C
YMR116C	YOR133W
YMR116C	YOR361C
YMR116C	YPL106C
YMR117C	YNL250W

YMR117C	YOL034W
YMR118C	YPL134C
YMR120C	YOR212W
YMR123W	YPL169C
YMR124W	YPR119W
YMR125W	YMR240C
YMR125W	YNL016W
YMR125W	YNL139C
YMR125W	YNL189W
YMR125W	YNL251C
YMR125W	YNL330C
YMR125W	YOL139C
YMR125W	YPL178W
YMR125W	YPL190C
YMR125W	YPR057W
YMR125W	YPR161C
YMR125W	YPR182W
YMR127C	YOR213C
YMR127C	YPR018W
YMR128W	YNL097C
YMR128W	YPL139C
YMR129W	YMR153W
YMR131C	YOR063W
YMR131C	YPL042C
YMR133W	YPL114W
YMR134W	YOR303W
YMR135C	YPL153C
YMR137C	YNL064C
YMR138W	YOR070C
YMR138W	YPL241C
YMR139W	YNL071W
YMR139W	YNL078W
YMR139W	YNL199C
YMR139W	YOL061W
YMR139W	YOL081W
YMR139W	YOR326W
YMR139W	YPL158C
YMR146C	YMR309C
YMR146C	YNL244C
YMR146C	YOL087C
YMR146C	YOR361C
YMR146C	YPL105C
YMR146C	YPR041W
YMR146C	YPR086W
YMR147W	YOR232W

YMR147W	YPR047W
YMR149W	YMR279C
YMR149W	YNL125C
YMR149W	YNR055C
YMR149W	YOR016C
YMR149W	YOR085W
YMR149W	YOR103C
YMR149W	YOR279C
YMR151W	YMR235C
YMR153W	YMR298W
YMR153W	YMR308C
YMR153W	YNL234W
YMR153W	YOL018C
YMR153W	YOL065C
YMR153W	YOL129W
YMR154C	YOR362C
YMR159C	YOR098C
YMR159C	YOR212W
YMR159C	YPL070W
YMR159C	YPL149W
YMR162C	YPR154W
YMR167W	YNL071W
YMR167W	YNL082W
YMR167W	YOL043C
YMR167W	YOL055C
YMR167W	YOL090W
YMR167W	YOR033C
YMR167W	YOR155C
YMR167W	YOR232W
YMR167W	YPL164C
YMR168C	YPL018W
YMR172W	YOR039W
YMR172W	YOR061W
YMR173W	YPR096C
YMR176W	YNL030W
YMR178W	YOR018W
YMR179W	YNL003C
YMR179W	YOR213C
YMR179W	YPL138C
YMR179W	YPL153C
YMR180C	YNL154C
YMR181C	YPR105C
YMR184W	YPR086W
YMR186W	YMR246W
YMR186W	YNL127W

YMR186W	YNL135C
YMR186W	YNL183C
YMR186W	YNL189W
YMR186W	YNL244C
YMR186W	YNR032W
YMR186W	YOL090W
YMR186W	YOL126C
YMR186W	YOR027W
YMR186W	YOR057W
YMR186W	YOR089C
YMR186W	YOR212W
YMR186W	YOR351C
YMR186W	YPL031C
YMR186W	YPL240C
YMR186W	YPL259C
YMR190C	YNL088W
YMR190C	YPL164C
YMR192W	YOR264W
YMR192W	YPR154W
YMR193W	YNL284C
YMR193W	YPR086W
YMR195W	YOR059C
YMR197C	YOL018C
YMR197C	YOR036W
YMR197C	YOR106W
YMR198W	YNL064C
YMR198W	YNR001C
YMR198W	YPR141C
YMR199W	YPL031C
YMR199W	YPL049C
YMR199W	YPL061W
YMR199W	YPL255W
YMR201C	YNL312W
YMR201C	YOL090W
YMR201C	YPL022W
YMR201C	YPR135W
YMR203W	YNL131W
YMR205C	YMR214W
YMR205C	YNL064C
YMR205C	YNL135C
YMR205C	YNL290W
YMR205C	YNL317W
YMR205C	YOL094C
YMR205C	YOL133W
YMR205C	YOR351C

YMR205C	YPL204W
YMR205C	YPR110C
YMR205C	YPR111W
YMR213W	YMR288W
YMR213W	YNR011C
YMR213W	YPL151C
YMR213W	YPR101W
YMR214W	YMR284W
YMR214W	YNL161W
YMR214W	YOL094C
YMR214W	YOL126C
YMR214W	YOR212W
YMR214W	YOR272W
YMR214W	YPL140C
YMR214W	YPR178W
YMR215W	YNL044W
YMR215W	YOR016C
YMR215W	YPL076W
YMR215W	YPL227C
YMR215W	YPL234C
YMR215W	YPR028W
YMR215W	YPR156C
YMR216C	YNL004W
YMR216C	YOR177C
YMR216C	YOR259C
YMR223W	YOR119C
YMR223W	YPL047W
YMR224C	YNL023C
YMR224C	YNL250W
YMR225C	YNL284C
YMR226C	YNL189W
YMR226C	YNL290W
YMR228W	YOR348C
YMR229C	YNL090W
YMR229C	YNL175C
YMR230W	YPR154W
YMR231W	YPL045W
YMR232W	YPR120C
YMR233W	YMR257C
YMR233W	YOL006C
YMR235C	YMR300C
YMR235C	YOR185C
YMR235C	YPL111W
YMR236W	YNL099C
YMR236W	YNL308C

YMR236W	YOR047C
YMR236W	YOR128C
YMR238W	YOL131W
YMR239C	YNL189W
YMR240C	YMR288W
YMR240C	YOR117W
YMR240C	YOR123C
YMR240C	YOR319W
YMR240C	YPL016W
YMR240C	YPL151C
YMR240C	YPL213W
YMR240C	YPR182W
YMR241W	YOL132W
YMR241W	YOR181W
YMR243C	YNL096C
YMR243C	YNR072W
YMR246W	YNL088W
YMR246W	YOL126C
YMR246W	YOL133W
YMR246W	YOL139C
YMR246W	YPR017C
YMR255W	YOR046C
YMR257C	YNL121C
YMR257C	YOR197W
YMR263W	YNL330C
YMR263W	YOL004W
YMR263W	YPR086W
YMR264W	YOR045W
YMR264W	YPL076W
YMR267W	YNL154C
YMR268C	YNL147W
YMR268C	YPR046W
YMR271C	YOR098C
YMR271C	YOR350C
YMR273C	YNL189W
YMR273C	YPL115C
YMR274C	YOL132W
YMR275C	YNL183C
YMR275C	YPL140C
YMR276W	YNL281W
YMR276W	YOR117W
YMR280C	YOR047C
YMR282C	YOL123W
YMR284W	YNL064C
YMR284W	YOR374W

YMR284W	YPL078C
YMR284W	YPL240C
YMR285C	YPR110C
YMR288W	YOR159C
YMR288W	YPR182W
YMR290C	YNL061W
YMR290C	YNL110C
YMR290C	YNL189W
YMR290C	YOR267C
YMR290C	YPL043W
YMR290C	YPR016C
YMR291W	YOL055C
YMR291W	YPL262W
YMR293C	YOR299W
YMR294W	YNL281W
YMR294W	YOL069W
YMR294W	YPL174C
YMR294W	YPR083W
YMR294W	YPR126C
YMR295C	YNL307C
YMR303C	YNL068C
YMR303C	YOL094C
YMR303C	YPR110C
YMR304W	YNL161W
YMR304W	YNL313C
YMR304W	YOR326W
YMR304W	YPR115W
YMR305C	YPR086W
YMR306W	YPR128C
YMR307W	YPL204W
YMR308C	YMR310C
YMR308C	YMR314W
YMR308C	YNL030W
YMR308C	YNL189W
YMR308C	YOL038W
YMR308C	YOL108C
YMR308C	YOL115W
YMR308C	YOL127W
YMR308C	YOR020C
YMR308C	YOR098C
YMR308C	YOR117W
YMR308C	YOR229W
YMR308C	YOR362C
YMR308C	YOR373W
YMR308C	YPL012W

YMR308C	YPL020C
YMR308C	YPL133C
YMR308C	YPL209C
YMR309C	YNL047C
YMR309C	YNL244C
YMR309C	YOL087C
YMR309C	YOR039W
YMR309C	YOR284W
YMR309C	YOR361C
YMR309C	YPL001W
YMR309C	YPR016C
YMR309C	YPR041W
YMR309C	YPR086W
YMR311C	YPL031C
YMR312W	YPL086C
YMR312W	YPL101W
YMR314W	YOR261C
YMR314W	YOR362C
YMR314W	YPL144W
YMR314W	YPR103W
YMR315W	YNL135C
YMR315W	YPR086W
YMR317W	YOL108C
YMR319C	YNL061W
YMR322C	YNL260C
YMR322C	YNL333W
YMR323W	YOR351C
YNL001W	YOL041C
YNL001W	YOR061W
YNL002C	YNL110C
YNL002C	YOR267C
YNL002C	YPL043W
YNL002C	YPL141C
YNL002C	YPR016C
YNL002C	YPR115W
YNL004W	YNL298W
YNL004W	YPR161C
YNL005C	YNL185C
YNL005C	YNL284C
YNL006W	YNL183C
YNL006W	YOL078W
YNL006W	YPL180W
YNL007C	YNL161W
YNL007C	YPL106C
YNL008C	YNL260C

YNL012WYNL064C
YNL015WYOL133W
YNL015WYPR086W
YNL016WYNL251C
YNL016WYNL262W
YNL016WYOR290C
YNL016WYPL016W
YNL016WYPL190C
YNL018C YOL075C
YNL020C YNL084C
YNL020C YNL243W
YNL020C YOR326W
YNL021WYOL090W
YNL021WYPR016C
YNL021WYPR179C
YNL023C YNL135C
YNL025C YNL094W
YNL025C YNL236W
YNL025C YOR140W
YNL025C YPL031C
YNL025C YPL042C
YNL027WYNR018W
YNL027WYPL204W
YNL028WYNL045W
YNL029C YPR041W
YNL029C YPR124W
YNL030WYNL113W
YNL030WYNL132W
YNL030WYNL182C
YNL030WYOL054W
YNL030WYOL108C
YNL030WYOR188W
YNL030WYPL001W
YNL030WYPL204W
YNL030WYPR010C
YNL030WYPR077C
YNL030WYPR104C
YNL030WYPR110C
YNL032WYNL056W
YNL032WYNL093W
YNL032WYNL099C
YNL036WYOL061W
YNL036WYOR303W
YNL037C YOL082W
YNL037C YOL133W

YNL037C YOR136W
YNL037C YPL140C
YNL037C YPL259C
YNL039W YOL115W
YNL039W YPL061W
YNL042W YOR014W
YNL042W YPL026C
YNL044W YNL189W
YNL044W YNL263C
YNL044W YPL070W
YNL047C YNL189W
YNL047C YOR014W
YNL047C YPL059W
YNL048W YPL076W
YNL048W YPL078C
YNL048W YPR028W
YNL048W YPR128C
YNL049C YPL085W
YNL049C YPR181C
YNL053W YOL055C
YNL053W YOR257W
YNL055C YNL090W
YNL055C YNL093W
YNL055C YNL106C
YNL055C YNL260C
YNL055C YNR010W
YNL055C YOL087C
YNL055C YOL132W
YNL056W YNL099C
YNL058C YNL307C
YNL061W YOL077C
YNL061W YOR005C
YNL061W YOR191W
YNL061W YOR206W
YNL061W YOR227W
YNL061W YOR267C
YNL061W YOR272W
YNL061W YPL009C
YNL061W YPL043W
YNL061W YPL093W
YNL061W YPL211W
YNL061W YPL217C
YNL061W YPL235W
YNL061W YPR016C
YNL062C YOR361C

YNL062C YPR086W
YNL064C YNL128W
YNL064C YNL182C
YNL064C YNL236W
YNL064C YNL250W
YNL064C YNL317W
YNL064C YNR031C
YNL064C YOL087C
YNL064C YOL108C
YNL064C YOL115W
YNL064C YOL133W
YNL064C YOL145C
YNL064C YOR080W
YNL064C YOR125C
YNL064C YOR181W
YNL064C YOR212W
YNL064C YOR319W
YNL064C YPL126W
YNL064C YPL140C
YNL064C YPL150W
YNL064C YPL259C
YNL064C YPR054W
YNL064C YPR119W
YNL068C YOL004W
YNL071W YNL113W
YNL071W YNL116W
YNL071W YNL135C
YNL071W YNL182C
YNL071W YOR181W
YNL071W YOR276W
YNL071W YPL194W
YNL071W YPL204W
YNL074C YPL256C
YNL076W YOR061W
YNL076W YOR326W
YNL078W YNR012W
YNL078W YOL070C
YNL082W YOL090W
YNL082W YPR154W
YNL084C YNL103W
YNL084C YNL250W
YNL084C YOR181W
YNL084C YPL174C
YNL085W YNL090W
YNL085W YNL250W

YNL085WYOL133W
YNL085WYPR111W
YNL086WYOL069W
YNL086WYOL091W
YNL086WYOL130W
YNL086WYOR174W
YNL086WYPR185W
YNL088WYNL161W
YNL088WYOR010C
YNL088WYOR061W
YNL088WYPL074W
YNL090WYNL210W
YNL090WYOL055C
YNL090WYOR089C
YNL090WYPL161C
YNL090WYPL174C
YNL090WYPL258C
YNL091WYNL164C
YNL091WYNL288W
YNL091WYOR355W
YNL091WYPL229W
YNL092WYOR004W
YNL092WYOR028C
YNL092WYOR040W
YNL092WYOR329C
YNL093WYNL227C
YNL093WYNL263C
YNL093WYOL139C
YNL093WYOR089C
YNL093WYOR370C
YNL093WYPL258C
YNL094WYNL243W
YNL094WYOR368W
YNL094WYPL004C
YNL094WYPR154W
YNL095C YPR198W
YNL096C YPR086W
YNL097C YNL330C
YNL097C YOL004W
YNL097C YOL020W
YNL098C YNL204C
YNL098C YOR101W
YNL099C YNL180C
YNL099C YOL006C
YNL101WYOR254C

YNL101WYPL076W
YNL101WYPR028W
YNL102WYNL262W
YNL102WYNR052C
YNL102WYPR135W
YNL103WYNL272C
YNL103WYPL038W
YNL106C YPL032C
YNL106C YPR173C
YNL106C YPR188C
YNL107WYOL012C
YNL107WYOR119C
YNL107WYOR244W
YNL107WYPL235W
YNL107WYPR023C
YNL110C YNL182C
YNL110C YNL307C
YNL110C YOL077C
YNL110C YOR005C
YNL110C YOR272W
YNL110C YPL012W
YNL110C YPL043W
YNL110C YPL093W
YNL110C YPR016C
YNL113WYOR116C
YNL113WYOR207C
YNL113WYOR341W
YNL113WYPL204W
YNL113WYPR010C
YNL113WYPR110C
YNL116WYNL311C
YNL116WYPL022W
YNL116WYPL110C
YNL118C YOL149W
YNL118C YOL151W
YNL118C YOR023C
YNL118C YOR093C
YNL118C YOR124C
YNL118C YPL204W
YNL118C YPR137C-B
YNL118C YPR160W
YNL121C YNL131W
YNL121C YOR008C
YNL121C YPR128C
YNL125C YNL130C

YNL125C YNR059W
YNL125C YPL076W
YNL127WYOL086C
YNL128WYPL164C
YNL129WYOR353C
YNL130C YNL336W
YNL130C YOR016C
YNL130C YOR067C
YNL132WYNL207W
YNL132WYNL230C
YNL132WYNL287W
YNL132WYOL102C
YNL132WYOR014W
YNL132WYOR080W
YNL132WYPL042C
YNL132WYPL043W
YNL132WYPL259C
YNL132WYPR106W
YNL135C YOR133W
YNL135C YOR319W
YNL135C YOR335C
YNL135C YOR374W
YNL135C YPL240C
YNL135C YPR074C
YNL135C YPR093C
YNL135C YPR119W
YNL138WYOL087C
YNL138WYOR122C
YNL139C YNL189W
YNL147WYOL139C
YNL147WYOL149W
YNL147WYOR308C
YNL147WYPR082C
YNL147WYPR178W
YNL151C YNR003C
YNL151C YOR116C
YNL151C YPR110C
YNL152WYPR154W
YNL154C YOR355W
YNL155WYOR364W
YNL159C YOR128C
YNL160WYOL133W
YNL161WYOL036W
YNL161WYOL139C
YNL161WYOR007C

YNL161WYOR151C
YNL172WYOR249C
YNL173C YOR020C
YNL175C YNL308C
YNL175C YOR206W
YNL175C YPL043W
YNL182C YNL189W
YNL182C YPL235W
YNL182C YPR016C
YNL183C YPL180W
YNL183C YPL204W
YNL187WYNR028W
YNL188WYOR257W
YNL188WYPL255W
YNL189WYNL232W
YNL189WYNL331C
YNL189WYNL333W
YNL189WYNR069C
YNL189WYOL004W
YNL189WYOL021C
YNL189WYOL058W
YNL189WYOL082W
YNL189WYOL135C
YNL189WYOR001W
YNL189WYOR020C
YNL189WYOR098C
YNL189WYOR133W
YNL189WYOR136W
YNL189WYOR155C
YNL189WYOR284W
YNL189WYPL049C
YNL189WYPL061W
YNL189WYPL088W
YNL189WYPL089C
YNL189WYPL111W
YNL189WYPL139C
YNL189WYPL153C
YNL189WYPL178W
YNL189WYPL214C
YNL189WYPL235W
YNL189WYPR018W
YNL189WYPR023C
YNL189WYPR025C
YNL189WYPR062W
YNL189WYPR182W

YNL189W	YPR191W
YNL189W	YPR193C
YNL191W	YPL249C
YNL194C	YPR079W
YNL197C	YPL204W
YNL199C	YPL075W
YNL199C	YPR048W
YNL201C	YOR047C
YNL201C	YOR355W
YNL201C	YPL031C
YNL201C	YPL153C
YNL201C	YPR101W
YNL201C	YPR115W
YNL207W	YOR039W
YNL207W	YOR056C
YNL207W	YOR061W
YNL207W	YOR145C
YNL207W	YOR290C
YNL207W	YPL012W
YNL207W	YPL042C
YNL207W	YPL204W
YNL207W	YPL266W
YNL208W	YOR285W
YNL214W	YOL044W
YNL215W	YPL235W
YNL216W	YPL075W
YNL217W	YOR099W
YNL222W	YPR107C
YNL225C	YOR373W
YNL226W	YPL211W
YNL229C	YNL307C
YNL229C	YPL204W
YNL229C	YPR110C
YNL230C	YOL041C
YNL230C	YOL133W
YNL230C	YOR017W
YNL230C	YOR272W
YNL230C	YPL001W
YNL230C	YPL004C
YNL230C	YPL012W
YNL230C	YPL046C
YNL230C	YPL093W
YNL232W	YOL021C
YNL232W	YOR001W
YNL232W	YOR076C

YNL236WYOL051W
YNL236WYOL135C
YNL236WYOR119C
YNL236WYOR140W
YNL236WYOR355W
YNL236WYPL240C
YNL238WYPL076W
YNL243WYNL298W
YNL243WYOR181W
YNL243WYOR284W
YNL244C YOR177C
YNL244C YOR335C
YNL244C YOR361C
YNL244C YOR362C
YNL244C YOR375C
YNL244C YPL028W
YNL244C YPL160W
YNL244C YPR041W
YNL244C YPR103W
YNL246WYOR299W
YNL246WYPR058W
YNL248C YOR341W
YNL248C YPR110C
YNL250WYOL069W
YNL250WYPL240C
YNL250WYPR160W
YNL251C YNL330C
YNL251C YPL178W
YNL251C YPL190C
YNL252C YNL284C
YNL252C YOR344C
YNL253WYPL150W
YNL261WYPR162C
YNL262WYOL139C
YNL262WYPR175W
YNL263C YNL304W
YNL263C YOR089C
YNL263C YPR028W
YNL265C YOL142W
YNL265C YOR260W
YNL265C YPL237W
YNL266WYOL131W
YNL271C YOR122C
YNL271C YOR188W
YNL271C YPR165W

YNL272C YPL204W
YNL273W YOL006C
YNL277W YPL169C
YNL279W YOL108C
YNL284C YOR150W
YNL284C YOR201C
YNL284C YPL204W
YNL285W YPL245W
YNL288W YNR052C
YNL288W YPR072W
YNL288W YPR086W
YNL289W YPL031C
YNL290W YOL055C
YNL290W YOL094C
YNL290W YOR144C
YNL290W YOR374W
YNL290W YPL235W
YNL290W YPL240C
YNL292W YNR050C
YNL292W YPR154W
YNL298W YOR127W
YNL298W YPL115C
YNL300W YPL076W
YNL304W YOR326W
YNL307C YOL054W
YNL307C YOL083W
YNL307C YOL130W
YNL307C YOL138C
YNL307C YOR266W
YNL307C YPR115W
YNL307C YPR159W
YNL308C YOR080W
YNL308C YOR299W
YNL308C YPL259C
YNL308C YPR110C
YNL309W YOL004W
YNL309W YPR120C
YNL311C YOR027W
YNL311C YOR151C
YNL311C YOR259C
YNL311C YPL061W
YNL311C YPL235W
YNL311C YPL240C
YNL312W YPL061W
YNL313C YOR117W

YNL314W	YOR047C
YNL317W	YPR107C
YNL320W	YOR078W
YNL323W	YOL055C
YNL330C	YOL004W
YNL330C	YOL006C
YNL330C	YOR039W
YNL330C	YOR083W
YNL330C	YPL139C
YNL330C	YPL181W
YNL330C	YPR023C
YNL331C	YPL070W
YNL333W	YNL334C
YNL334C	YPR175W
YNR001C	YOL133W
YNR001C	YOR212W
YNR002C	YOR130C
YNR003C	YOR116C
YNR003C	YOR207C
YNR003C	YOR224C
YNR003C	YPR110C
YNR003C	YPR190C
YNR004W	YPL157W
YNR007C	YOR160W
YNR010W	YOL135C
YNR010W	YOL139C
YNR010W	YOR174W
YNR011C	YPL241C
YNR015W	YOR264W
YNR016C	YPL026C
YNR016C	YPR110C
YNR017W	YPL063W
YNR019W	YOR381W
YNR022C	YPR119W
YNR023W	YOR119C
YNR023W	YOR290C
YNR023W	YPL016W
YNR031C	YOR374W
YNR031C	YOR375C
YNR032W	YPR040W
YNR033W	YPR154W
YNR039C	YOR174W
YNR040W	YOL131W
YNR042W	YPL253C
YNR046W	YNR050C

YNR046W	YOL124C
YNR047W	YOR353C
YNR050C	YOR164C
YNR052C	YOR047C
YNR053C	YOR308C
YNR053C	YPR016C
YNR063W	YOR368W
YNR064C	YOL108C
YNR064C	YPR154W
YNR065C	YOR085W
YNR065C	YOR181W
YNR068C	YNR069C
YNR068C	YOR264W
YNR069C	YOR299W
YOL001W	YPL031C
YOL001W	YPR115W
YOL003C	YPL076W
YOL003C	YPL264C
YOL004W	YOR061W
YOL004W	YOR160W
YOL004W	YPL139C
YOL004W	YPL181W
YOL004W	YPR023C
YOL005C	YOR224C
YOL005C	YPL203W
YOL005C	YPR093C
YOL006C	YPR190C
YOL009C	YOR023C
YOL010W	YPL259C
YOL011W	YOL105C
YOL012C	YPL235W
YOL014W	YOL039W
YOL015W	YPR171W
YOL016C	YOR007C
YOL017W	YOL051W
YOL018C	YOL132W
YOL018C	YOR327C
YOL021C	YOL142W
YOL021C	YOR001W
YOL021C	YOR076C
YOL034W	YOR324C
YOL035C	YOR315W
YOL038W	YPL144W
YOL038W	YPR103W
YOL038W	YPR154W

YOL041C YOL100W
YOL041C YOR080W
YOL041C YOR208W
YOL041C YOR233W
YOL041C YPL043W
YOL041C YPL141C
YOL043C YOR264W
YOL044W YOR076C
YOL051W YOL135C
YOL051W YOR119C
YOL051W YOR174W
YOL051W YPL248C
YOL051W YPR070W
YOL054W YOL055C
YOL054W YOR056C
YOL054W YOR178C
YOL054W YPL258C
YOL054W YPR120C
YOL055C YOL100W
YOL055C YOL115W
YOL055C YOR080W
YOL055C YOR231W
YOL055C YOR276W
YOL055C YOR339C
YOL055C YOR386W
YOL055C YPL022W
YOL055C YPL150W
YOL055C YPL153C
YOL055C YPL194W
YOL055C YPR110C
YOL059W YPL022W
YOL061W YPL196W
YOL063C YOL133W
YOL064C YOR299W
YOL066C YPR086W
YOL067C YOL108C
YOL068C YOR279C
YOL069W YPL124W
YOL069W YPR141C
YOL070C YOR098C
YOL070C YPR086W
YOL073C YOR065W
YOL077C YPL043W
YOL077C YPL259C
YOL078W YOR014W

YOL078WYPR137W
YOL081WYPL204W
YOL082WYOR115C
YOL082WYOR302W
YOL082WYOR353C
YOL082WYPL070W
YOL082WYPL091W
YOL082WYPL204W
YOL083WYPL204W
YOL086C YOL135C
YOL086C YOR056C
YOL086C YOR244W
YOL086C YOR304W
YOL086C YPL042C
YOL086C YPL082C
YOL086C YPR180W
YOL090WYOR033C
YOL090WYOR323C
YOL090WYPL022W
YOL090WYPR179C
YOL094C YOR144C
YOL094C YOR217W
YOL094C YPL061W
YOL094C YPL194W
YOL094C YPL240C
YOL095C YPL270W
YOL100WYPL004C
YOL103W-B YOR142W-A
YOL106WYPR182W
YOL108C YOR344C
YOL108C YPL211W
YOL111C YOR007C
YOL111C YPR086W
YOL113WYPL169C
YOL115WYPL061W
YOL115WYPL146C
YOL115WYPL235W
YOL115WYPL258C
YOL117WYOL133W
YOL123WYPL140C
YOL123WYPR106W
YOL128C YOR185C
YOL131WYOR047C
YOL131WYOR123C
YOL131WYPL249C

YOL132W	YOR016C
YOL132W	YOR099W
YOL132W	YPL274W
YOL133W	YOR261C
YOL133W	YOR319W
YOL133W	YOR326W
YOL133W	YOR341W
YOL133W	YPL036W
YOL133W	YPL061W
YOL133W	YPL240C
YOL133W	YPR137C-B
YOL133W	YPR164W
YOL135C	YOR174W
YOL135C	YPR070W
YOL139C	YOR276W
YOL139C	YPL178W
YOL145C	YOR039W
YOL145C	YOR061W
YOL145C	YOR123C
YOL145C	YOR326W
YOL145C	YPR103W
YOL147C	YPR086W
YOL148C	YOR119C
YOL148C	YPL254W
YOL148C	YPR086W
YOL149W	YOR167C
YOL149W	YPL204W
YOL149W	YPR132W
YOL150C	YPL008W
YOL150C	YPR086W
YOL152W	YPR086W
YOL156W	YOR299W
YOR001W	YOR326W
YOR005C	YOR272W
YOR005C	YPL110C
YOR005C	YPL235W
YOR005C	YPL240C
YOR005C	YPR016C
YOR007C	YOR164C
YOR014W	YOR039W
YOR014W	YOR061W
YOR014W	YOR119C
YOR014W	YPR115W
YOR014W	YPR143W
YOR014W	YPR164W

YOR016C	YOR211C
YOR016C	YPL076W
YOR016C	YPL264C
YOR016C	YPL274W
YOR016C	YPR156C
YOR016C	YPR198W
YOR016C	YPR201W
YOR027W	YPL240C
YOR027W	YPR054W
YOR027W	YPR178W
YOR029W	YPR154W
YOR034C	YOR171C
YOR035C	YOR326W
YOR035C	YPL200W
YOR036W	YPL151C
YOR036W	YPL240C
YOR037W	YOR299W
YOR037W	YPR086W
YOR038C	YOR290C
YOR039W	YOR061W
YOR039W	YOR119C
YOR039W	YOR302W
YOR039W	YOR303W
YOR039W	YOR361C
YOR039W	YPL093W
YOR039W	YPL174C
YOR039W	YPL235W
YOR042W	YPR154W
YOR043W	YPR030W
YOR045W	YPR086W
YOR046C	YPL140C
YOR047C	YOR167C
YOR047C	YOR299W
YOR047C	YOR302W
YOR047C	YOR358W
YOR047C	YPR103W
YOR049C	YOR299W
YOR049C	YOR302W
YOR049C	YPR086W
YOR051C	YPL022W
YOR054C	YOR061W
YOR057W	YPL240C
YOR061W	YOR119C
YOR061W	YOR267C
YOR061W	YOR294W

YOR061W	YOR341W
YOR061W	YPL204W
YOR061W	YPR181C
YOR063W	YPR154W
YOR064C	YPR086W
YOR066W	YPR120C
YOR069W	YOR132W
YOR069W	YOR181W
YOR074C	YPL093W
YOR074C	YPR086W
YOR075W	YPR040W
YOR079C	YPR086W
YOR079C	YPR198W
YOR080W	YPL012W
YOR080W	YPL061W
YOR080W	YPL211W
YOR080W	YPL217C
YOR080W	YPL258C
YOR080W	YPR016C
YOR080W	YPR135W
YOR082C	YPR086W
YOR083W	YPL116W
YOR083W	YPR068C
YOR084W	YPL209C
YOR085W	YOR103C
YOR089C	YOR370C
YOR089C	YOR375C
YOR089C	YPR174C
YOR090C	YPR016C
YOR097C	YOR245C
YOR098C	YOR133W
YOR098C	YOR160W
YOR098C	YOR229W
YOR098C	YOR326W
YOR098C	YOR370C
YOR098C	YPL174C
YOR098C	YPR172W
YOR098C	YPR187W
YOR101W	YOR351C
YOR101W	YPR169W
YOR106W	YPL195W
YOR110W	YPR086W
YOR114W	YPR086W
YOR116C	YOR207C
YOR116C	YOR224C

YOR116C YPR110C
YOR116C YPR187W
YOR116C YPR190C
YOR117W YOR177C
YOR117W YOR259C
YOR117W YPR108W
YOR119C YOR174W
YOR119C YOR290C
YOR119C YPL016W
YOR119C YPL031C
YOR119C YPL042C
YOR119C YPL204W
YOR119C YPL254W
YOR119C YPR010C-A
YOR119C YPR070W
YOR119C YPR110C
YOR119C YPR168W
YOR122C YOR363C
YOR127W YPL256C
YOR127W YPR165W
YOR128C YOR393W
YOR128C YPL169C
YOR128C YPR045C
YOR128C YPR054W
YOR128C YPR084W
YOR128C YPR085C
YOR128C YPR088C
YOR128C YPR111W
YOR131C YPL034W
YOR133W YPL031C
YOR136W YPL031C
YOR136W YPR110C
YOR136W YPR111W
YOR146W YPL194W
YOR151C YOR210W
YOR151C YPL137C
YOR151C YPL203W
YOR151C YPR093C
YOR151C YPR110C
YOR151C YPR111W
YOR151C YPR187W
YOR154W YPR086W
YOR155C YPL070W
YOR156C YPL161C
YOR156C YPL174C

YOR157C	YPL144W
YOR157C	YPR103W
YOR159C	YPL213W
YOR159C	YPR178W
YOR159C	YPR182W
YOR161C	YPR154W
YOR164C	YPR105C
YOR165W	YPR028W
YOR171C	YOR185C
YOR171C	YPL022W
YOR172W	YOR239W
YOR173W	YPR025C
YOR177C	YOR265W
YOR178C	YPL204W
YOR181W	YPL235W
YOR181W	YPL246C
YOR181W	YPR154W
YOR181W	YPR159W
YOR187W	YPL259C
YOR190W	YPR033C
YOR191W	YPR016C
YOR191W	YPR154W
YOR194C	YPR086W
YOR197W	YPR154W
YOR198C	YPL042C
YOR198C	YPR106W
YOR201C	YPL141C
YOR202W	YPR016C
YOR206W	YOR267C
YOR206W	YPL043W
YOR207C	YPR015C
YOR207C	YPR110C
YOR210W	YOR341W
YOR210W	YPL203W
YOR210W	YPR010C
YOR210W	YPR110C
YOR210W	YPR111W
YOR212W	YOR281C
YOR212W	YOR374W
YOR212W	YPL240C
YOR213C	YPL208W
YOR213C	YPR018W
YOR215C	YPL204W
YOR224C	YOR341W
YOR224C	YPL203W

YOR224C	YPR110C
YOR226C	YPL088W
YOR229W	YOR230W
YOR229W	YOR283W
YOR229W	YPL235W
YOR230W	YPL061W
YOR232W	YOR239W
YOR232W	YPL204W
YOR232W	YPL222W
YOR233W	YOR294W
YOR233W	YPL093W
YOR243C	YOR356W
YOR244W	YPR023C
YOR252W	YPR152C
YOR254C	YPL076W
YOR254C	YPL094C
YOR254C	YPL264C
YOR257W	YPL180W
YOR259C	YPL248C
YOR259C	YPR108W
YOR260W	YPL070W
YOR260W	YPL237W
YOR261C	YPR108W
YOR264W	YOR324C
YOR264W	YPR119W
YOR264W	YPR120C
YOR267C	YOR294W
YOR267C	YPL093W
YOR267C	YPL260W
YOR267C	YPR016C
YOR267C	YPR143W
YOR270C	YOR332W
YOR270C	YPR036W
YOR271C	YPR086W
YOR272W	YPL043W
YOR272W	YPR016C
YOR272W	YPR088C
YOR275C	YPR173C
YOR284W	YPL070W
YOR285W	YOR312C
YOR285W	YPR183W
YOR288C	YPR085C
YOR290C	YPL016W
YOR290C	YPL129W
YOR290C	YPR034W

YOR293W	YPR154W
YOR294W	YPR102C
YOR294W	YPR115W
YOR299W	YPR054W
YOR308C	YPR178W
YOR310C	YPR137W
YOR311C	YPR124W
YOR312C	YPR119W
YOR319W	YPR188C
YOR324C	YPL093W
YOR325W	YPR141C
YOR327C	YPL232W
YOR327C	YPL270W
YOR329C	YPR154W
YOR331C	YPR105C
YOR332W	YPL204W
YOR332W	YPR110C
YOR335C	YPR111W
YOR339C	YPL258C
YOR340C	YOR341W
YOR341W	YPR010C
YOR341W	YPR110C
YOR341W	YPR187W
YOR355W	YPR048W
YOR358W	YPL248C
YOR361C	YPL001W
YOR361C	YPR016C
YOR361C	YPR041W
YOR361C	YPR086W
YOR362C	YPL144W
YOR362C	YPR103W
YOR368W	YPL194W
YOR374W	YPL150W
YOR374W	YPR110C
YOR380W	YPR040W
YOR386W	YPL104W
YPL002C	YPR086W
YPL003W	YPR066W
YPL004C	YPL022W
YPL004C	YPL074W
YPL004C	YPL150W
YPL004C	YPL157W
YPL004C	YPL204W
YPL011C	YPL129W
YPL012W	YPL043W

YPL012W	YPL141C
YPL016W	YPR034W
YPL019C	YPR154W
YPL022W	YPL061W
YPL026C	YPL028W
YPL029W	YPL194W
YPL031C	YPL219W
YPL031C	YPL240C
YPL031C	YPR025C
YPL034W	YPR123C
YPL036W	YPL136W
YPL038W	YPR154W
YPL042C	YPL248C
YPL042C	YPR181C
YPL043W	YPL093W
YPL043W	YPL259C
YPL043W	YPR072W
YPL049C	YPL256C
YPL050C	YPR154W
YPL051W	YPR086W
YPL061W	YPR110C
YPL066W	YPR103W
YPL070W	YPL124W
YPL070W	YPR119W
YPL070W	YPR193C
YPL076W	YPL264C
YPL076W	YPR028W
YPL078C	YPL204W
YPL082C	YPR073C
YPL084W	YPR154W
YPL084W	YPR173C
YPL085W	YPR181C
YPL086C	YPL101W
YPL093W	YPL259C
YPL093W	YPR016C
YPL093W	YPR017C
YPL093W	YPR041W
YPL093W	YPR115W
YPL094C	YPR086W
YPL097W	YPR086W
YPL111W	YPL160W
YPL112C	YPR154W
YPL115C	YPL161C
YPL124W	YPL155C
YPL124W	YPL255W

YPL127C	YPL153C
YPL128C	YPL153C
YPL129W	YPL203W
YPL131W	YPR016C
YPL139C	YPL181W
YPL140C	YPL235W
YPL140C	YPR108W
YPL147W	YPL274W
YPL150W	YPL235W
YPL150W	YPL258C
YPL151C	YPR182W
YPL160W	YPR110C
YPL161C	YPR165W
YPL162C	YPR079W
YPL164C	YPR191W
YPL174C	YPR165W
YPL178W	YPL190C
YPL187W	YPL218W
YPL187W	YPR181C
YPL203W	YPL247C
YPL203W	YPR133C
YPL203W	YPR187W
YPL204W	YPR008W
YPL204W	YPR049C
YPL204W	YPR160W
YPL204W	YPR181C
YPL210C	YPR088C
YPL213W	YPR182W
YPL227C	YPL264C
YPL227C	YPL274W
YPL227C	YPR198W
YPL234C	YPL264C
YPL235W	YPR110C
YPL235W	YPR111W
YPL237W	YPR041W
YPL240C	YPR054W
YPL240C	YPR178W
YPL243W	YPR088C
YPL248C	YPR086W
YPL249C	YPR154W
YPL249C-A	YPR154W
YPL253C	YPR141C
YPL257W-B	YPR137C-B
YPL258C	YPR110C
YPL258C	YPR154W

YPL259C	YPR016C
YPL259C	YPR029C
YPL259C	YPR159W
YPL264C	YPR028W
YPL269W	YPR120C
YPR010C	YPR016C
YPR010C	YPR110C
YPR016C	YPR025C
YPR019W	YPR135W
YPR020W	YPR086W
YPR020W	YPR194C
YPR032W	YPR086W
YPR032W	YPR110C
YPR037C	YPR156C
YPR041W	YPR086W
YPR048W	YPR070W
YPR054W	YPR191W
YPR066W	YPR086W
YPR067W	YPR110C
YPR071W	YPR154W
YPR079W	YPR201W
YPR081C	YPR154W
YPR082C	YPR182W
YPR086W	YPR155C
YPR086W	YPR157W
YPR094W	YPR133C
YPR101W	YPR182W
YPR105C	YPR191W
YPR106W	YPR115W
YPR106W	YPR163C
YPR110C	YPR187W
YPR110C	YPR190C
YPR111W	YPR122W
YPR111W	YPR160W
YPR112C	YPR154W
YPR120C	YPR162C
YPR154W	YPR171W
YPR178W	YPR182W
