# Supplementary material for: A disease-related essential protein prediction model based on the transfer neural network
Source: Front Genet. 2023 Jan 4;13:1087294. doi: 10.3389/fgene.2022.1087294 (PMC9845409; doi:10.3389/fgene.2022.1087294)
Supplement: Supplementary file 5 [file DataSheet5.PDF]

YAL007C Endoplasmic
YAL009W Endoplasmic
YAL010C Endoplasmic
YAL023C Endoplasmic
YAL028W Endoplasmic
YAL042W Endoplasmic
YAL048C Endoplasmic
YAL053W Endoplasmic
YAL054C Endoplasmic
YAL058W Endoplasmic
YAR002C-A Endoplasmic
YAR031W Endoplasmic
YAR033W Endoplasmic
YAR042W Endoplasmic
YBL011W Endoplasmic
YBL020W Endoplasmic
YBL040C Endoplasmic
YBL041W Endoplasmic
YBL082C Endoplasmic
YBR002C Endoplasmic
YBR004C Endoplasmic
YBR005W Endoplasmic
YBR014C Endoplasmic
YBR029C Endoplasmic
YBR036C Endoplasmic
YBR041W Endoplasmic
YBR058C-A Endoplasmic
YBR070C Endoplasmic
YBR086C Endoplasmic
YBR096W Endoplasmic
YBR110W Endoplasmic
YBR130C Endoplasmic
YBR132C Endoplasmic
YBR159W Endoplasmic
YBR160W Endoplasmic
YBR162W-A Endoplasmic
YBR170C Endoplasmic
YBR171W Endoplasmic
YBR172C Endoplasmic
YBR183W Endoplasmic
YBR201W Endoplasmic
YBR229C Endoplasmic
YBR243C Endoplasmic
YBR254C Endoplasmic
YBR265W Endoplasmic

YBR273C Endoplasmic
YBR283C Endoplasmic
YBR287W Endoplasmic
YBR290W Endoplasmic
YCL043C Endoplasmic
YCL045C Endoplasmic
YCL052C Endoplasmic
YCR011C Endoplasmic
YCR017C Endoplasmic
YCR034W Endoplasmic
YCR044C Endoplasmic
YCR048W Endoplasmic
YCR067C Endoplasmic
YCR068W Endoplasmic
YDL010W Endoplasmic
YDL015C Endoplasmic
YDL018C Endoplasmic
YDL019C Endoplasmic
YDL058W Endoplasmic
YDL065C Endoplasmic
YDL072C Endoplasmic
YDL093W Endoplasmic
YDL095W Endoplasmic
YDL100C Endoplasmic
YDL121C Endoplasmic
YDL122W Endoplasmic
YDL126C Endoplasmic
YDL149W Endoplasmic
YDL193W Endoplasmic
YDL195W Endoplasmic
YDL204W Endoplasmic
YDL212W Endoplasmic
YDL226C Endoplasmic
YDL232W Endoplasmic
YDR049W Endoplasmic
YDR056C Endoplasmic
YDR057W Endoplasmic
YDR062W Endoplasmic
YDR086C Endoplasmic
YDR126W Endoplasmic
YDR150W Endoplasmic
YDR182W Endoplasmic
YDR189W Endoplasmic
YDR196C Endoplasmic
YDR200C Endoplasmic

YDR205W Endoplasmic
YDR221W Endoplasmic
YDR233C Endoplasmic
YDR236C Endoplasmic
YDR245W Endoplasmic
YDR246W Endoplasmic
YDR292C Endoplasmic
YDR294C Endoplasmic
YDR297W Endoplasmic
YDR302W Endoplasmic
YDR304C Endoplasmic
YDR307W Endoplasmic
YDR320C Endoplasmic
YDR329C Endoplasmic
YDR331W Endoplasmic
YDR349C Endoplasmic
YDR410C Endoplasmic
YDR411C Endoplasmic
YDR414C Endoplasmic
YDR434W Endoplasmic
YDR437W Endoplasmic
YDR472W Endoplasmic
YDR476C Endoplasmic
YDR492W Endoplasmic
YDR498C Endoplasmic
YDR518W Endoplasmic
YDR519W Endoplasmic
YEL001C Endoplasmic
YEL002C Endoplasmic
YEL004W Endoplasmic
YEL031W Endoplasmic
YEL036C Endoplasmic
YEL043W Endoplasmic
YEL064C Endoplasmic
YER004W Endoplasmic
YER012W Endoplasmic
YER019C-A Endoplasmic
YER019W Endoplasmic
YER039C Endoplasmic
YER044C Endoplasmic
YER053C-A Endoplasmic
YER072W Endoplasmic
YER074W-A Endoplasmic
YER083C Endoplasmic
YER087C-B Endoplasmic

YER094C Endoplasmic
YER100W Endoplasmic
YER120W Endoplasmic
YER122C Endoplasmic
YER140W Endoplasmic
YFL004W Endoplasmic
YFL025C Endoplasmic
YFL038C Endoplasmic
YFL048C Endoplasmic
YFR008W Endoplasmic
YFR041C Endoplasmic
YFR042W Endoplasmic
YFR050C Endoplasmic
YGL001C Endoplasmic
YGL002W Endoplasmic
YGL010W Endoplasmic
YGL012W Endoplasmic
YGL020C Endoplasmic
YGL022W Endoplasmic
YGL027C Endoplasmic
YGL038C Endoplasmic
YGL047W Endoplasmic
YGL051W Endoplasmic
YGL053W Endoplasmic
YGL054C Endoplasmic
YGL055W Endoplasmic
YGL065C Endoplasmic
YGL084C Endoplasmic
YGL098W Endoplasmic
YGL126W Endoplasmic
YGL139W Endoplasmic
YGL142C Endoplasmic
YGL145W Endoplasmic
YGL160W Endoplasmic
YGL200C Endoplasmic
YGL219C Endoplasmic
YGL225W Endoplasmic
YGL226C-A Endoplasmic
YGL231C Endoplasmic
YGR036C Endoplasmic
YGR038W Endoplasmic
YGR048W Endoplasmic
YGR060W Endoplasmic
YGR089W Endoplasmic
YGR105W Endoplasmic

YGR106C Endoplasmic
YGR157W Endoplasmic
YGR172C Endoplasmic
YGR175C Endoplasmic
YGR177C Endoplasmic
YGR199W Endoplasmic
YGR212W Endoplasmic
YGR216C Endoplasmic
YGR227W Endoplasmic
YGR263C Endoplasmic
YGR270W Endoplasmic
YGR284C Endoplasmic
YHL003C Endoplasmic
YHL020C Endoplasmic
YHL028W Endoplasmic
YHR001W Endoplasmic
YHR004C Endoplasmic
YHR007C Endoplasmic
YHR027C Endoplasmic
YHR036W Endoplasmic
YHR039C Endoplasmic
YHR042W Endoplasmic
YHR045W Endoplasmic
YHR060W Endoplasmic
YHR072W Endoplasmic
YHR073W Endoplasmic
YHR079C Endoplasmic
YHR098C Endoplasmic
YHR101C Endoplasmic
YHR110W Endoplasmic
YHR133C Endoplasmic
YHR135C Endoplasmic
YHR140W Endoplasmic
YHR142W Endoplasmic
YHR176W Endoplasmic
YHR181W Endoplasmic
YHR188C Endoplasmic
YHR190W Endoplasmic
YHR192W Endoplasmic
YHR204W Endoplasmic
YIL004C Endoplasmic
YIL005W Endoplasmic
YIL016W Endoplasmic
YIL023C Endoplasmic
YIL027C Endoplasmic

YIL030C Endoplasmic
YIL039W Endoplasmic
YIL040W Endoplasmic
YIL043C Endoplasmic
YIL049W Endoplasmic
YIL089W Endoplasmic
YIL090W Endoplasmic
YIL102C-A Endoplasmic
YIL109C Endoplasmic
YIL124W Endoplasmic
YIR022W Endoplasmic
YIR033W Endoplasmic
YIR038C Endoplasmic
YJL001W Endoplasmic
YJL002C Endoplasmic
YJL005W Endoplasmic
YJL012C Endoplasmic
YJL034W Endoplasmic
YJL062W Endoplasmic
YJL073W Endoplasmic
YJL079C Endoplasmic
YJL080C Endoplasmic
YJL091C Endoplasmic
YJL097W Endoplasmic
YJL117W Endoplasmic
YJL134W Endoplasmic
YJL145W Endoplasmic
YJL158C Endoplasmic
YJL167W Endoplasmic
YJL192C Endoplasmic
YJL196C Endoplasmic
YJR010C-A Endoplasmic
YJR013W Endoplasmic
YJR015W Endoplasmic
YJR040W Endoplasmic
YJR073C Endoplasmic
YJR088C Endoplasmic
YJR117W Endoplasmic
YJR118C Endoplasmic
YJR131W Endoplasmic
YJR143C Endoplasmic
YKL008C Endoplasmic
YKL020C Endoplasmic
YKL044W Endoplasmic
YKL046C Endoplasmic

YKL065C Endoplasmic
YKL073W Endoplasmic
YKL094W Endoplasmic
YKL100C Endoplasmic
YKL119C Endoplasmic
YKL154W Endoplasmic
YKL165C Endoplasmic
YKL207W Endoplasmic
YKL212W Endoplasmic
YKR003W Endoplasmic
YKR014C Endoplasmic
YKR022C Endoplasmic
YKR030W Endoplasmic
YKR053C Endoplasmic
YKR067W Endoplasmic
YKR068C Endoplasmic
YLL006W Endoplasmic
YLL014W Endoplasmic
YLL023C Endoplasmic
YLL031C Endoplasmic
YLL052C Endoplasmic
YLL053C Endoplasmic
YLL055W Endoplasmic
YLL061W Endoplasmic
YLR004C Endoplasmic
YLR023C Endoplasmic
YLR034C Endoplasmic
YLR050C Endoplasmic
YLR056W Endoplasmic
YLR057W Endoplasmic
YLR064W Endoplasmic
YLR066W Endoplasmic
YLR074C Endoplasmic
YLR078C Endoplasmic
YLR080W Endoplasmic
YLR088W Endoplasmic
YLR100W Endoplasmic
YLR155C Endoplasmic
YLR160C Endoplasmic
YLR205C Endoplasmic
YLR207W Endoplasmic
YLR208W Endoplasmic
YLR238W Endoplasmic
YLR242C Endoplasmic
YLR246W Endoplasmic

YLR268W Endoplasmic
YLR286C Endoplasmic
YLR292C Endoplasmic
YLR301W Endoplasmic
YLR310C Endoplasmic
YLR350W Endoplasmic
YLR372W Endoplasmic
YLR378C Endoplasmic
YLR404W Endoplasmic
YLR440C Endoplasmic
YLR450W Endoplasmic
YLR459W Endoplasmic
YML008C Endoplasmic
YML012W Endoplasmic
YML013W Endoplasmic
YML019W Endoplasmic
YML029W Endoplasmic
YML048W Endoplasmic
YML055W Endoplasmic
YML059C Endoplasmic
YML066C Endoplasmic
YML067C Endoplasmic
YML072C Endoplasmic
YML075C Endoplasmic
YML077W Endoplasmic
YML101C Endoplasmic
YML115C Endoplasmic
YML125C Endoplasmic
YML128C Endoplasmic
YML130C Endoplasmic
YMR008C Endoplasmic
YMR013C Endoplasmic
YMR015C Endoplasmic
YMR022W Endoplasmic
YMR029C Endoplasmic
YMR040W Endoplasmic
YMR052W Endoplasmic
YMR065W Endoplasmic
YMR122W-A Endoplasmic
YMR123W Endoplasmic
YMR134W Endoplasmic
YMR149W Endoplasmic
YMR161W Endoplasmic
YMR165C Endoplasmic
YMR183C Endoplasmic

YMR200W Endoplasmic
YMR202W Endoplasmic
YMR214W Endoplasmic
YMR238W Endoplasmic
YMR251W-A Endoplasmic
YMR264W Endoplasmic
YMR272C Endoplasmic
YMR274C Endoplasmic
YMR281W Endoplasmic
YMR292W Endoplasmic
YMR296C Endoplasmic
YMR297W Endoplasmic
YMR298W Endoplasmic
YMR305C Endoplasmic
YNL012W Endoplasmic
YNL024C-A Endoplasmic
YNL038W Endoplasmic
YNL044W Endoplasmic
YNL046W Endoplasmic
YNL048W Endoplasmic
YNL049C Endoplasmic
YNL080C Endoplasmic
YNL087W Endoplasmic
YNL098C Endoplasmic
YNL111C Endoplasmic
YNL125C Endoplasmic
YNL127W Endoplasmic
YNL130C Endoplasmic
YNL146W Endoplasmic
YNL149C Endoplasmic
YNL156C Endoplasmic
YNL158W Endoplasmic
YNL181W Endoplasmic
YNL194C Endoplasmic
YNL219C Endoplasmic
YNL231C Endoplasmic
YNL258C Endoplasmic
YNL263C Endoplasmic
YNL280C Endoplasmic
YNL291C Endoplasmic
YNL304W Endoplasmic
YNL305C Endoplasmic
YNL321W Endoplasmic
YNL323W Endoplasmic
YNR008W Endoplasmic

YNR016C Endoplasmic
YNR019W Endoplasmic
YNR021W Endoplasmic
YNR026C Endoplasmic
YNR030W Endoplasmic
YNR039C Endoplasmic
YNR075W Endoplasmic
YOL003C Endoplasmic
YOL009C Endoplasmic
YOL013C Endoplasmic
YOL031C Endoplasmic
YOL044W Endoplasmic
YOL065C Endoplasmic
YOL081W Endoplasmic
YOL088C Endoplasmic
YOL101C Endoplasmic
YOL110W Endoplasmic
YOL147C Endoplasmic
YOR002W Endoplasmic
YOR016C Endoplasmic
YOR044W Endoplasmic
YOR049C Endoplasmic
YOR056C Endoplasmic
YOR060C Endoplasmic
YOR067C Endoplasmic
YOR075W Endoplasmic
YOR085W Endoplasmic
YOR086C Endoplasmic
YOR092W Endoplasmic
YOR103C Endoplasmic
YOR115C Endoplasmic
YOR149C Endoplasmic
YOR154W Endoplasmic
YOR157C Endoplasmic
YOR165W Endoplasmic
YOR171C Endoplasmic
YOR175C Endoplasmic
YOR198C Endoplasmic
YOR223W Endoplasmic
YOR245C Endoplasmic
YOR254C Endoplasmic
YOR285W Endoplasmic
YOR288C Endoplasmic
YOR307C Endoplasmic
YOR311C Endoplasmic

YOR317W Endoplasmic
YOR321W Endoplasmic
YOR324C Endoplasmic
YOR336W Endoplasmic
YPL019C Endoplasmic
YPL050C Endoplasmic
YPL076W Endoplasmic
YPL085W Endoplasmic
YPL087W Endoplasmic
YPL094C Endoplasmic
YPL096C-A Endoplasmic
YPL137C Endoplasmic
YPL175W Endoplasmic
YPL186C Endoplasmic
YPL200W Endoplasmic
YPL207W Endoplasmic
YPL210C Endoplasmic
YPL218W Endoplasmic
YPL221W Endoplasmic
YPL227C Endoplasmic
YPL244C Endoplasmic
YPL274W Endoplasmic
YPR003C Endoplasmic
YPR028W Endoplasmic
YPR037C Endoplasmic
YPR063C Endoplasmic
YPR071W Endoplasmic
YPR091C Endoplasmic
YPR103W Endoplasmic
YPR105C Endoplasmic
YPR113W Endoplasmic
YPR114W Endoplasmic
YPR139C Endoplasmic
YPR149W Endoplasmic
YPR159W Endoplasmic
YPR173C Endoplasmic
YPR181C Endoplasmic
YPR183W Endoplasmic
YPR192W Endoplasmic
YAL016W Cytoskeleton
YAL029C Cytoskeleton
YAL034W-A Cytoskeleton
YAL047C Cytoskeleton
YAR019C Cytoskeleton
YBL007C Cytoskeleton

YBL031W Cytoskeleton
YBL034C Cytoskeleton
YBL047C Cytoskeleton
YBL063W Cytoskeleton
YBL105C Cytoskeleton
YBR108W Cytoskeleton
YBR109C Cytoskeleton
YBR118W Cytoskeleton
YBR133C Cytoskeleton
YBR148W Cytoskeleton
YBR156C Cytoskeleton
YBR200W Cytoskeleton
YBR211C Cytoskeleton
YBR233W-A Cytoskeleton
YBR234C Cytoskeleton
YBR260C Cytoskeleton
YCL014W Cytoskeleton
YCL024W Cytoskeleton
YCL029C Cytoskeleton
YCL034W Cytoskeleton
YCR002C Cytoskeleton
YCR009C Cytoskeleton
YCR088W Cytoskeleton
YDL028C Cytoskeleton
YDL029W Cytoskeleton
YDL058W Cytoskeleton
YDL117W Cytoskeleton
YDL161W Cytoskeleton
YDL225W Cytoskeleton
YDL226C Cytoskeleton
YDL239C Cytoskeleton
YDR016C Cytoskeleton
YDR022C Cytoskeleton
YDR063W Cytoskeleton
YDR106W Cytoskeleton
YDR113C Cytoskeleton
YDR126W Cytoskeleton
YDR129C Cytoskeleton
YDR130C Cytoskeleton
YDR171W Cytoskeleton
YDR201W Cytoskeleton
YDR218C Cytoskeleton
YDR309C Cytoskeleton
YDR320C-A Cytoskeleton
YDR356W Cytoskeleton

YDR379W Cytoskeleton
YDR388W Cytoskeleton
YDR389W Cytoskeleton
YDR409W Cytoskeleton
YDR424C Cytoskeleton
YDR484W Cytoskeleton
YDR488C Cytoskeleton
YDR510W Cytoskeleton
YDR532C Cytoskeleton
YEL040W Cytoskeleton
YEL061C Cytoskeleton
YER007W Cytoskeleton
YER016W Cytoskeleton
YER114C Cytoskeleton
YER125W Cytoskeleton
YER133W Cytoskeleton
YFL005W Cytoskeleton
YFL037W Cytoskeleton
YFL039C Cytoskeleton
YFR028C Cytoskeleton
YGL061C Cytoskeleton
YGL075C Cytoskeleton
YGL093W Cytoskeleton
YGL106W Cytoskeleton
YGL170C Cytoskeleton
YGL173C Cytoskeleton
YGL181W Cytoskeleton
YGL216W Cytoskeleton
YGR032W Cytoskeleton
YGR059W Cytoskeleton
YGR080W Cytoskeleton
YGR092W Cytoskeleton
YGR098C Cytoskeleton
YGR113W Cytoskeleton
YGR136W Cytoskeleton
YGR140W Cytoskeleton
YGR198W Cytoskeleton
YGR218W Cytoskeleton
YGR241C Cytoskeleton
YHR014W Cytoskeleton
YHR016C Cytoskeleton
YHR023W Cytoskeleton
YHR061C Cytoskeleton
YHR107C Cytoskeleton
YHR114W Cytoskeleton

YHR129C Cytoskeleton
YHR161C Cytoskeleton
YHR172W Cytoskeleton
YHR185C Cytoskeleton
YHR199C-A Cytoskeleton
YIL002C Cytoskeleton
YIL034C Cytoskeleton
YIL062C Cytoskeleton
YIL095W Cytoskeleton
YIL106W Cytoskeleton
YIL138C Cytoskeleton
YIL140W Cytoskeleton
YIL149C Cytoskeleton
YIR003W Cytoskeleton
YIR006C Cytoskeleton
YIR010W Cytoskeleton
YJL019W Cytoskeleton
YJL020C Cytoskeleton
YJL042W Cytoskeleton
YJR053W Cytoskeleton
YJR065C Cytoskeleton
YJR076C Cytoskeleton
YJR089W Cytoskeleton
YJR090C Cytoskeleton
YJR092W Cytoskeleton
YJR125C Cytoskeleton
YKL007W Cytoskeleton
YKL013C Cytoskeleton
YKL042W Cytoskeleton
YKL048C Cytoskeleton
YKL052C Cytoskeleton
YKL056C Cytoskeleton
YKL079W Cytoskeleton
YKL101W Cytoskeleton
YKL129C Cytoskeleton
YKL138C-A Cytoskeleton
YKL222C Cytoskeleton
YKR001C Cytoskeleton
YKR037C Cytoskeleton
YKR041W Cytoskeleton
YKR054C Cytoskeleton
YKR083C Cytoskeleton
YLL003W Cytoskeleton
YLL038C Cytoskeleton
YLL049W Cytoskeleton

YLL050C Cytoskeleton
YLR045C Cytoskeleton
YLR175W Cytoskeleton
YLR186W Cytoskeleton
YLR206W Cytoskeleton
YLR212C Cytoskeleton
YLR227C Cytoskeleton
YLR229C Cytoskeleton
YLR254C Cytoskeleton
YLR313C Cytoskeleton
YLR314C Cytoskeleton
YLR319C Cytoskeleton
YLR337C Cytoskeleton
YLR342W Cytoskeleton
YLR370C Cytoskeleton
YLR410W Cytoskeleton
YLR412W Cytoskeleton
YLR429W Cytoskeleton
YLR457C Cytoskeleton
YML031W Cytoskeleton
YML064C Cytoskeleton
YML085C Cytoskeleton
YML104C Cytoskeleton
YML124C Cytoskeleton
YMR001C Cytoskeleton
YMR032W Cytoskeleton
YMR055C Cytoskeleton
YMR092C Cytoskeleton
YMR109W Cytoskeleton
YMR198W Cytoskeleton
YMR294W Cytoskeleton
YMR299C Cytoskeleton
YNL020C Cytoskeleton
YNL079C Cytoskeleton
YNL084C Cytoskeleton
YNL094W Cytoskeleton
YNL106C Cytoskeleton
YNL116W Cytoskeleton
YNL126W Cytoskeleton
YNL138W Cytoskeleton
YNL148C Cytoskeleton
YNL152W Cytoskeleton
YNL164C Cytoskeleton
YNL166C Cytoskeleton
YNL172W Cytoskeleton

YNL188W Cytoskeleton
YNL225C Cytoskeleton
YNL233W Cytoskeleton
YNL243W Cytoskeleton
YNL271C Cytoskeleton
YNR035C Cytoskeleton
YOL069W Cytoskeleton
YOL091W Cytoskeleton
YOR014W Cytoskeleton
YOR058C Cytoskeleton
YOR060C Cytoskeleton
YOR073W Cytoskeleton
YOR109W Cytoskeleton
YOR122C Cytoskeleton
YOR127W Cytoskeleton
YOR141C Cytoskeleton
YOR177C Cytoskeleton
YOR181W Cytoskeleton
YOR195W Cytoskeleton
YOR233W Cytoskeleton
YOR239W Cytoskeleton
YOR257W Cytoskeleton
YOR265W Cytoskeleton
YOR269W Cytoskeleton
YOR326W Cytoskeleton
YOR329C Cytoskeleton
YOR367W Cytoskeleton
YOR373W Cytoskeleton
YPL017C Cytoskeleton
YPL124W Cytoskeleton
YPL155C Cytoskeleton
YPL174C Cytoskeleton
YPL192C Cytoskeleton
YPL204W Cytoskeleton
YPL209C Cytoskeleton
YPL233W Cytoskeleton
YPL241C Cytoskeleton
YPL242C Cytoskeleton
YPL253C Cytoskeleton
YPL255W Cytoskeleton
YPL269W Cytoskeleton
YPR080W Cytoskeleton
YPR111W Cytoskeleton
YPR119W Cytoskeleton
YPR141C Cytoskeleton

YPR154W Cytoskeleton
YPR171W Cytoskeleton
YPR174C Cytoskeleton
YPR188C Cytoskeleton
YAL002W Golgi
YAL026C Golgi
YAL042W Golgi
YAR033W Golgi
YAR042W Golgi
YBL017C Golgi
YBL069W Golgi
YBL102W Golgi
YBR014C Golgi
YBR015C Golgi
YBR029C Golgi
YBR080C Golgi
YBR097W Golgi
YBR164C Golgi
YBR183W Golgi
YBR187W Golgi
YBR199W Golgi
YBR254C Golgi
YBR288C Golgi
YCL001W Golgi
YCL057W Golgi
YCR043C Golgi
YCR067C Golgi
YCR068W Golgi
YDL010W Golgi
YDL058W Golgi
YDL099W Golgi
YDL100C Golgi
YDL137W Golgi
YDL145C Golgi
YDL149W Golgi
YDL192W Golgi
YDL226C Golgi
YDR027C Golgi
YDR084C Golgi
YDR100W Golgi
YDR108W Golgi
YDR137W Golgi
YDR141C Golgi
YDR170C Golgi
YDR189W Golgi

YDR233C Golgi
YDR238C Golgi
YDR245W Golgi
YDR246W Golgi
YDR264C Golgi
YDR270W Golgi
YDR351W Golgi
YDR358W Golgi
YDR367W Golgi
YDR372C Golgi
YDR373W Golgi
YDR407C Golgi
YDR468C Golgi
YDR472W Golgi
YDR483W Golgi
YDR484W Golgi
YDR503C Golgi
YDR517W Golgi
YEL022W Golgi
YEL036C Golgi
YEL042W Golgi
YEL048C Golgi
YER001W Golgi
YER005W Golgi
YER031C Golgi
YER039C Golgi
YER074W-A Golgi
YER083C Golgi
YER113C Golgi
YER122C Golgi
YER125W Golgi
YER157W Golgi
YER166W Golgi
YFL038C Golgi
YFL048C Golgi
YFR051C Golgi
YGL005C Golgi
YGL020C Golgi
YGL038C Golgi
YGL051W Golgi
YGL054C Golgi
YGL095C Golgi
YGL137W Golgi
YGL161C Golgi
YGL167C Golgi

YGL198W Golgi
YGL200C Golgi
YGL203C Golgi
YGL206C Golgi
YGL210W Golgi
YGL223C Golgi
YGL225W Golgi
YGL257C Golgi
YGR120C Golgi
YGR166W Golgi
YGR167W Golgi
YGR170W Golgi
YGR172C Golgi
YGR202C Golgi
YGR209C Golgi
YGR247W Golgi
YGR261C Golgi
YHL017W Golgi
YHL019C Golgi
YHL031C Golgi
YHR098C Golgi
YHR103W Golgi
YHR108W Golgi
YHR123W Golgi
YHR181W Golgi
YIL004C Golgi
YIL014W Golgi
YIL041W Golgi
YIL044C Golgi
YIL048W Golgi
YIL076W Golgi
YIL085C Golgi
YIL109C Golgi
YIL173W Golgi
YJL004C Golgi
YJL024C Golgi
YJL029C Golgi
YJL094C Golgi
YJL099W Golgi
YJL123C Golgi
YJL139C Golgi
YJL178C Golgi
YJL183W Golgi
YJL186W Golgi
YJL204C Golgi

YJL207C Golgi
YJL222W Golgi
YJR031C Golgi
YJR040W Golgi
YJR066W Golgi
YJR075W Golgi
YJR125C Golgi
YJR134C Golgi
YKL004W Golgi
YKL006C-A Golgi
YKL034W Golgi
YKL039W Golgi
YKL063C Golgi
YKL135C Golgi
YKL165C Golgi
YKL174C Golgi
YKL179C Golgi
YKL196C Golgi
YKL209C Golgi
YKL212W Golgi
YKR020W Golgi
YKR027W Golgi
YKR030W Golgi
YKR061W Golgi
YKR068C Golgi
YKR088C Golgi
YLL040C Golgi
YLR026C Golgi
YLR039C Golgi
YLR043C Golgi
YLR078C Golgi
YLR080W Golgi
YLR170C Golgi
YLR220W Golgi
YLR240W Golgi
YLR242C Golgi
YLR260W Golgi
YLR262C Golgi
YLR268W Golgi
YLR309C Golgi
YLR330W Golgi
YLR360W Golgi
YML012W Golgi
YML038C Golgi
YML067C Golgi

YML071C Golgi
YML077W Golgi
YML115C Golgi
YMR052W Golgi
YMR054W Golgi
YMR071C Golgi
YMR079W Golgi
YMR162C Golgi
YMR192W Golgi
YMR197C Golgi
YMR218C Golgi
YMR237W Golgi
YMR292W Golgi
YNL006W Golgi
YNL024C-A Golgi
YNL029C Golgi
YNL041C Golgi
YNL044W Golgi
YNL049C Golgi
YNL051W Golgi
YNL127W Golgi
YNL130C Golgi
YNL183C Golgi
YNL238W Golgi
YNL263C Golgi
YNL287W Golgi
YNL297C Golgi
YNR026C Golgi
YOL018C Golgi
YOL107W Golgi
YOL137W Golgi
YOR034C Golgi
YOR036W Golgi
YOR069W Golgi
YOR070C Golgi
YOR079C Golgi
YOR094W Golgi
YOR099W Golgi
YOR115C Golgi
YOR171C Golgi
YOR216C Golgi
YOR299W Golgi
YOR311C Golgi
YOR320C Golgi
YOR322C Golgi

YOR357C Golgi
YPL010W Golgi
YPL050C Golgi
YPL051W Golgi
YPL085W Golgi
YPL087W Golgi
YPL145C Golgi
YPL195W Golgi
YPL218W Golgi
YPL246C Golgi
YPL249C Golgi
YPL259C Golgi
YPR028W Golgi
YPR029C Golgi
YPR079W Golgi
YPR089W Golgi
YPR095C Golgi
YPR105C Golgi
YPR113W Golgi
YPR159W Golgi
YPR165W Golgi
YPR181C Golgi
YPR194C Golgi
YAL035W Cytosol
YAL054C Cytosol
YAR002W Cytosol
YAR007C Cytosol
YBL015W Cytosol
YBL018C Cytosol
YBL027W Cytosol
YBL041W Cytosol
YBL050W Cytosol
YBL072C Cytosol
YBL075C Cytosol
YBL076C Cytosol
YBL078C Cytosol
YBL087C Cytosol
YBL092W Cytosol
YBR017C Cytosol
YBR019C Cytosol
YBR030W Cytosol
YBR031W Cytosol
YBR048W Cytosol
YBR084C-A Cytosol
YBR095C Cytosol

YBR097W Cytosol
YBR101C Cytosol
YBR128C Cytosol
YBR137W Cytosol
YBR143C Cytosol
YBR149W Cytosol
YBR152W Cytosol
YBR164C Cytosol
YBR181C Cytosol
YBR189W Cytosol
YBR191W Cytosol
YBR218C Cytosol
YBR231C Cytosol
YBR236C Cytosol
YBR261C Cytosol
YBR272C Cytosol
YBR276C Cytosol
YBR289W Cytosol
YCL011C Cytosol
YCL018W Cytosol
YCL028W Cytosol
YCL040W Cytosol
YCR020C Cytosol
YCR031C Cytosol
YCR032W Cytosol
YCR033W Cytosol
YCR073W-A Cytosol
YCR077C Cytosol
YDL005C Cytosol
YDL022W Cytosol
YDL061C Cytosol
YDL065C Cytosol
YDL075W Cytosol
YDL081C Cytosol
YDL082W Cytosol
YDL083C Cytosol
YDL087C Cytosol
YDL097C Cytosol
YDL106C Cytosol
YDL108W Cytosol
YDL126C Cytosol
YDL130W Cytosol
YDL133C-A Cytosol
YDL135C Cytosol
YDL136W Cytosol

YDL143W Cytosol
YDL147W Cytosol
YDL184C Cytosol
YDL191W Cytosol
YDR012W Cytosol
YDR025W Cytosol
YDR044W Cytosol
YDR049W Cytosol
YDR064W Cytosol
YDR073W Cytosol
YDR079C-A Cytosol
YDR091C Cytosol
YDR096W Cytosol
YDR098C Cytosol
YDR142C Cytosol
YDR165W Cytosol
YDR170C Cytosol
YDR174W Cytosol
YDR180W Cytosol
YDR188W Cytosol
YDR195W Cytosol
YDR212W Cytosol
YDR226W Cytosol
YDR228C Cytosol
YDR244W Cytosol
YDR255C Cytosol
YDR267C Cytosol
YDR283C Cytosol
YDR295C Cytosol
YDR311W Cytosol
YDR334W Cytosol
YDR353W Cytosol
YDR357C Cytosol
YDR363W-A Cytosol
YDR372C Cytosol
YDR382W Cytosol
YDR392W Cytosol
YDR409W Cytosol
YDR416W Cytosol
YDR418W Cytosol
YDR427W Cytosol
YDR447C Cytosol
YDR450W Cytosol
YDR464W Cytosol
YDR471W Cytosol

YDR487C Cytosol
YDR490C Cytosol
YDR500C Cytosol
YDR513W Cytosol
YEL003W Cytosol
YEL005C Cytosol
YEL018W Cytosol
YEL021W Cytosol
YEL022W Cytosol
YEL034W Cytosol
YEL046C Cytosol
YEL047C Cytosol
YEL054C Cytosol
YEL058W Cytosol
YER007C-A Cytosol
YER012W Cytosol
YER024W Cytosol
YER040W Cytosol
YER046W Cytosol
YER057C Cytosol
YER074W Cytosol
YER094C Cytosol
YER102W Cytosol
YER117W Cytosol
YER131W Cytosol
YER161C Cytosol
YER162C Cytosol
YER175C Cytosol
YFL007W Cytosol
YFL013C Cytosol
YFL014W Cytosol
YFL021W Cytosol
YFL023W Cytosol
YFL034C-A Cytosol
YFL045C Cytosol
YFL049W Cytosol
YFR004W Cytosol
YFR009W Cytosol
YFR021W Cytosol
YFR031C-A Cytosol
YFR032C-A Cytosol
YFR036W Cytosol
YFR050C Cytosol
YFR052W Cytosol
YFR053C Cytosol

YGL004C Cytosol
YGL009C Cytosol
YGL011C Cytosol
YGL013C Cytosol
YGL030W Cytosol
YGL031C Cytosol
YGL047W Cytosol
YGL048C Cytosol
YGL062W Cytosol
YGL066W Cytosol
YGL076C Cytosol
YGL079W Cytosol
YGL095C Cytosol
YGL099W Cytosol
YGL103W Cytosol
YGL112C Cytosol
YGL123W Cytosol
YGL124C Cytosol
YGL135W Cytosol
YGL147C Cytosol
YGL180W Cytosol
YGL189C Cytosol
YGL195W Cytosol
YGL211W Cytosol
YGL220W Cytosol
YGL246C Cytosol
YGL253W Cytosol
YGL258W Cytosol
YGR019W Cytosol
YGR027C Cytosol
YGR030C Cytosol
YGR034W Cytosol
YGR054W Cytosol
YGR058W Cytosol
YGR074W Cytosol
YGR078C Cytosol
YGR085C Cytosol
YGR093W Cytosol
YGR118W Cytosol
YGR129W Cytosol
YGR135W Cytosol
YGR142W Cytosol
YGR144W Cytosol
YGR148C Cytosol
YGR156W Cytosol

YGR198W Cytosol
YGR206W Cytosol
YGR209C Cytosol
YGR214W Cytosol
YGR232W Cytosol
YGR234W Cytosol
YGR239C Cytosol
YGR240C Cytosol
YGR248W Cytosol
YGR252W Cytosol
YGR253C Cytosol
YGR254W Cytosol
YGR256W Cytosol
YGR270W Cytosol
YGR274C Cytosol
YHL001W Cytosol
YHL015W Cytosol
YHL025W Cytosol
YHL033C Cytosol
YHR010W Cytosol
YHR018C Cytosol
YHR019C Cytosol
YHR021C Cytosol
YHR027C Cytosol
YHR035W Cytosol
YHR053C Cytosol
YHR055C Cytosol
YHR062C Cytosol
YHR085W Cytosol
YHR090C Cytosol
YHR109W Cytosol
YHR111W Cytosol
YHR133C Cytosol
YHR141C Cytosol
YHR160C Cytosol
YHR170W Cytosol
YHR171W Cytosol
YHR174W Cytosol
YHR197W Cytosol
YHR201C Cytosol
YHR202W Cytosol
YHR203C Cytosol
YHR206W Cytosol
YIL007C Cytosol
YIL008W Cytosol

YIL018W Cytosol
YIL026C Cytosol
YIL036W Cytosol
YIL052C Cytosol
YIL063C Cytosol
YIL069C Cytosol
YIL075C Cytosol
YIL084C Cytosol
YIL104C Cytosol
YIL113W Cytosol
YIL118W Cytosol
YIL131C Cytosol
YIL133C Cytosol
YIL142W Cytosol
YIL143C Cytosol
YIL148W Cytosol
YIR004W Cytosol
YIR012W Cytosol
YJL001W Cytosol
YJL006C Cytosol
YJL008C Cytosol
YJL011C Cytosol
YJL014W Cytosol
YJL036W Cytosol
YJL061W Cytosol
YJL068C Cytosol
YJL088W Cytosol
YJL111W Cytosol
YJL115W Cytosol
YJL121C Cytosol
YJL136C Cytosol
YJL145W Cytosol
YJL168C Cytosol
YJL176C Cytosol
YJL177W Cytosol
YJL179W Cytosol
YJL189W Cytosol
YJL190C Cytosol
YJL191W Cytosol
YJR006W Cytosol
YJR031C Cytosol
YJR032W Cytosol
YJR047C Cytosol
YJR064W Cytosol
YJR094W-A Cytosol

YJR103W Cytosol
YJR104C Cytosol
YJR123W Cytosol
YJR145C Cytosol
YKL006W Cytosol
YKL022C Cytosol
YKL056C Cytosol
YKL059C Cytosol
YKL060C Cytosol
YKL061W Cytosol
YKL062W Cytosol
YKL067W Cytosol
YKL113C Cytosol
YKL126W Cytosol
YKL127W Cytosol
YKL152C Cytosol
YKL156W Cytosol
YKL180W Cytosol
YKL182W Cytosol
YKL206C Cytosol
YKR057W Cytosol
YKR071C Cytosol
YKR080W Cytosol
YKR082W Cytosol
YKR094C Cytosol
YKR097W Cytosol
YLL009C Cytosol
YLL018C-A Cytosol
YLL024C Cytosol
YLL026W Cytosol
YLL029W Cytosol
YLL036C Cytosol
YLL045C Cytosol
YLR011W Cytosol
YLR027C Cytosol
YLR028C Cytosol
YLR029C Cytosol
YLR043C Cytosol
YLR044C Cytosol
YLR048W Cytosol
YLR061W Cytosol
YLR075W Cytosol
YLR116W Cytosol
YLR131C Cytosol
YLR153C Cytosol

YLR167W Cytosol
YLR174W Cytosol
YLR185W Cytosol
YLR195C Cytosol
YLR200W Cytosol
YLR211C Cytosol
YLR240W Cytosol
YLR244C Cytosol
YLR249W Cytosol
YLR262C Cytosol
YLR264W Cytosol
YLR287C-A Cytosol
YLR304C Cytosol
YLR309C Cytosol
YLR310C Cytosol
YLR325C Cytosol
YLR333C Cytosol
YLR340W Cytosol
YLR344W Cytosol
YLR360W Cytosol
YLR367W Cytosol
YLR377C Cytosol
YLR380W Cytosol
YLR388W Cytosol
YLR396C Cytosol
YLR406C Cytosol
YLR408C Cytosol
YLR421C Cytosol
YLR438W Cytosol
YLR441C Cytosol
YLR448W Cytosol
YLR455W Cytosol
YML024W Cytosol
YML026C Cytosol
YML028W Cytosol
YML063W Cytosol
YML073C Cytosol
YML092C Cytosol
YML094W Cytosol
YML097C Cytosol
YML102W Cytosol
YML113W Cytosol
YMR016C Cytosol
YMR018W Cytosol
YMR028W Cytosol

YMR037C Cytosol
YMR038C Cytosol
YMR043W Cytosol
YMR061W Cytosol
YMR070W Cytosol
YMR076C Cytosol
YMR079W Cytosol
YMR105C Cytosol
YMR116C Cytosol
YMR120C Cytosol
YMR121C Cytosol
YMR142C Cytosol
YMR143W Cytosol
YMR165C Cytosol
YMR182C Cytosol
YMR194W Cytosol
YMR205C Cytosol
YMR230W Cytosol
YMR235C Cytosol
YMR242C Cytosol
YMR277W Cytosol
YMR278W Cytosol
YMR314W Cytosol
YMR323W Cytosol
YNL007C Cytosol
YNL014W Cytosol
YNL035C Cytosol
YNL053W Cytosol
YNL064C Cytosol
YNL067W Cytosol
YNL068C Cytosol
YNL069C Cytosol
YNL086W Cytosol
YNL096C Cytosol
YNL117W Cytosol
YNL119W Cytosol
YNL141W Cytosol
YNL153C Cytosol
YNL162W Cytosol
YNL167C Cytosol
YNL178W Cytosol
YNL207W Cytosol
YNL216W Cytosol
YNL223W Cytosol
YNL227C Cytosol

YNL229C Cytosol
YNL240C Cytosol
YNL246W Cytosol
YNL247W Cytosol
YNL259C Cytosol
YNL264C Cytosol
YNL272C Cytosol
YNL273W Cytosol
YNL282W Cytosol
YNL290W Cytosol
YNL293W Cytosol
YNL297C Cytosol
YNL301C Cytosol
YNL302C Cytosol
YNL329C Cytosol
YNR007C Cytosol
YNR010W Cytosol
YNR023W Cytosol
YNR024W Cytosol
YNR031C Cytosol
YNR035C Cytosol
YNR043W Cytosol
YOL038W Cytosol
YOL039W Cytosol
YOL040C Cytosol
YOL055C Cytosol
YOL058W Cytosol
YOL059W Cytosol
YOL094C Cytosol
YOL111C Cytosol
YOL115W Cytosol
YOL120C Cytosol
YOL121C Cytosol
YOL126C Cytosol
YOL127W Cytosol
YOR007C Cytosol
YOR038C Cytosol
YOR063W Cytosol
YOR069W Cytosol
YOR096W Cytosol
YOR113W Cytosol
YOR122C Cytosol
YOR157C Cytosol
YOR164C Cytosol
YOR167C Cytosol

YOR168W Cytosol
YOR182C Cytosol
YOR197W Cytosol
YOR229W Cytosol
YOR234C Cytosol
YOR236W Cytosol
YOR261C Cytosol
YOR274W Cytosol
YOR293W Cytosol
YOR302W Cytosol
YOR312C Cytosol
YOR322C Cytosol
YOR357C Cytosol
YOR359W Cytosol
YOR362C Cytosol
YOR369C Cytosol
YOR387C Cytosol
YOR388C Cytosol
YOR393W Cytosol
YPL061W Cytosol
YPL070W Cytosol
YPL079W Cytosol
YPL081W Cytosol
YPL090C Cytosol
YPL091W Cytosol
YPL096W Cytosol
YPL100W Cytosol
YPL111W Cytosol
YPL120W Cytosol
YPL123C Cytosol
YPL131W Cytosol
YPL138C Cytosol
YPL143W Cytosol
YPL149W Cytosol
YPL153C Cytosol
YPL198W Cytosol
YPL201C Cytosol
YPL203W Cytosol
YPL214C Cytosol
YPL220W Cytosol
YPL231W Cytosol
YPL249C Cytosol
YPL249C-A Cytosol
YPL262W Cytosol
YPL281C Cytosol

YPR017C Cytosol
YPR032W Cytosol
YPR041W Cytosol
YPR043W Cytosol
YPR102C Cytosol
YPR103W Cytosol
YPR107C Cytosol
YPR132W Cytosol
YPR144C Cytosol
YPR179C Cytosol
YPR180W Cytosol
YAL005C Vacuole
YAL022C Vacuole
YAR028W Vacuole
YBL050W Vacuole
YBL078C Vacuole
YBL089W Vacuole
YBL104C Vacuole
YBR014C Vacuole
YBR074W Vacuole
YBR077C Vacuole
YBR097W Vacuole
YBR127C Vacuole
YBR128C Vacuole
YBR131W Vacuole
YBR132C Vacuole
YBR139W Vacuole
YBR147W Vacuole
YBR161W Vacuole
YBR162C Vacuole
YBR187W Vacuole
YBR205W Vacuole
YBR207W Vacuole
YBR235W Vacuole
YBR241C Vacuole
YBR286W Vacuole
YBR290W Vacuole
YBR293W Vacuole
YBR298C Vacuole
YBR302C Vacuole
YCL001W Vacuole
YCL005W-A Vacuole
YCL038C Vacuole
YCL057W Vacuole
YCL063W Vacuole

YCL069W Vacuole
YCL073C Vacuole
YCR010C Vacuole
YCR011C Vacuole
YCR023C Vacuole
YCR028C Vacuole
YCR044C Vacuole
YCR068W Vacuole
YCR075C Vacuole
YDL010W Vacuole
YDL046W Vacuole
YDL054C Vacuole
YDL077C Vacuole
YDL123W Vacuole
YDL128W Vacuole
YDL180W Vacuole
YDL185W Vacuole
YDL210W Vacuole
YDL211C Vacuole
YDR003W Vacuole
YDR080W Vacuole
YDR089W Vacuole
YDR105C Vacuole
YDR107C Vacuole
YDR119W Vacuole
YDR128W Vacuole
YDR131C Vacuole
YDR135C Vacuole
YDR229W Vacuole
YDR262W Vacuole
YDR281C Vacuole
YDR284C Vacuole
YDR313C Vacuole
YDR323C Vacuole
YDR352W Vacuole
YDR422C Vacuole
YDR452W Vacuole
YDR456W Vacuole
YDR477W Vacuole
YDR481C Vacuole
YDR486C Vacuole
YDR497C Vacuole
YDR525W-A Vacuole
YEL005C Vacuole
YEL013W Vacuole

YEL027W Vacuole
YEL051W Vacuole
YEL060C Vacuole
YEL062W Vacuole
YEL064C Vacuole
YER056C Vacuole
YER072W Vacuole
YER093C Vacuole
YER119C Vacuole
YER123W Vacuole
YFL004W Vacuole
YFL041W Vacuole
YFL048C Vacuole
YFL062W Vacuole
YFR019W Vacuole
YFR021W Vacuole
YGL006W Vacuole
YGL023C Vacuole
YGL095C Vacuole
YGL100W Vacuole
YGL114W Vacuole
YGL124C Vacuole
YGL156W Vacuole
YGL180W Vacuole
YGL190C Vacuole
YGL212W Vacuole
YGR020C Vacuole
YGR106C Vacuole
YGR125W Vacuole
YGR138C Vacuole
YGR141W Vacuole
YGR163W Vacuole
YGR170W Vacuole
YGR209C Vacuole
YGR223C Vacuole
YGR254W Vacuole
YGR279C Vacuole
YGR295C Vacuole
YHL008C Vacuole
YHL023C Vacuole
YHL035C Vacuole
YHR026W Vacuole
YHR028C Vacuole
YHR039C-A Vacuole
YHR050W Vacuole

YHR113W Vacuole
YHR132C Vacuole
YHR174W Vacuole
YHR175W Vacuole
YHR186C Vacuole
YHR202W Vacuole
YHR205W Vacuole
YHR215W Vacuole
YIL067C Vacuole
YIL088C Vacuole
YIL089W Vacuole
YIL099W Vacuole
YIL146C Vacuole
YIL158W Vacuole
YIR014W Vacuole
YJL012C Vacuole
YJL053W Vacuole
YJL058C Vacuole
YJL059W Vacuole
YJL079C Vacuole
YJL097W Vacuole
YJL100W Vacuole
YJL151C Vacuole
YJL154C Vacuole
YJL172W Vacuole
YJR001W Vacuole
YJR040W Vacuole
YJR054W Vacuole
YJR066W Vacuole
YJR124C Vacuole
YJR138W Vacuole
YJR161C Vacuole
YKL064W Vacuole
YKL077W Vacuole
YKL080W Vacuole
YKL103C Vacuole
YKL124W Vacuole
YKL146W Vacuole
YKL165C Vacuole
YKL175W Vacuole
YKL196C Vacuole
YKL203C Vacuole
YKR001C Vacuole
YKR007W Vacuole
YKR013W Vacuole

YKR039W Vacuole
YKR103W Vacuole
YKR105C Vacuole
YKR106W Vacuole
YLL015W Vacuole
YLL024C Vacuole
YLL028W Vacuole
YLL043W Vacuole
YLL048C Vacuole
YLL051C Vacuole
YLR001C Vacuole
YLR034C Vacuole
YLR043C Vacuole
YLR047C Vacuole
YLR083C Vacuole
YLR093C Vacuole
YLR148W Vacuole
YLR173W Vacuole
YLR178C Vacuole
YLR208W Vacuole
YLR220W Vacuole
YLR229C Vacuole
YLR240W Vacuole
YLR241W Vacuole
YLR297W Vacuole
YLR299W Vacuole
YLR360W Vacuole
YLR386W Vacuole
YLR396C Vacuole
YLR447C Vacuole
YML001W Vacuole
YML018C Vacuole
YML116W Vacuole
YML121W Vacuole
YML132W Vacuole
YMR054W Vacuole
YMR068W Vacuole
YMR077C Vacuole
YMR088C Vacuole
YMR160W Vacuole
YMR165C Vacuole
YMR171C Vacuole
YMR195W Vacuole
YMR221C Vacuole
YMR231W Vacuole

YMR243C Vacuole
YMR266W Vacuole
YMR297W Vacuole
YNL006W Vacuole
YNL015W Vacuole
YNL054W Vacuole
YNL058C Vacuole
YNL101W Vacuole
YNL115C Vacuole
YNL176C Vacuole
YNL180C Vacuole
YNL217W Vacuole
YNL275W Vacuole
YNL293W Vacuole
YNL298W Vacuole
YNL305C Vacuole
YNL321W Vacuole
YNL325C Vacuole
YNL326C Vacuole
YNL336W Vacuole
YNR013C Vacuole
YNR028W Vacuole
YNR061C Vacuole
YOL007C Vacuole
YOL019W Vacuole
YOL052C-A Vacuole
YOL060C Vacuole
YOL075C Vacuole
YOL078W Vacuole
YOL084W Vacuole
YOL092W Vacuole
YOL119C Vacuole
YOL129W Vacuole
YOL138C Vacuole
YOL154W Vacuole
YOR034C Vacuole
YOR068C Vacuole
YOR087W Vacuole
YOR099W Vacuole
YOR106W Vacuole
YOR219C Vacuole
YOR223W Vacuole
YOR247W Vacuole
YOR270C Vacuole
YOR273C Vacuole

YOR288C Vacuole
YOR291W Vacuole
YOR292C Vacuole
YOR316C Vacuole
YOR320C Vacuole
YOR332W Vacuole
YPL006W Vacuole
YPL019C Vacuole
YPL045W Vacuole
YPL057C Vacuole
YPL087W Vacuole
YPL100W Vacuole
YPL120W Vacuole
YPL123C Vacuole
YPL149W Vacuole
YPL154C Vacuole
YPL162C Vacuole
YPL163C Vacuole
YPL176C Vacuole
YPL180W Vacuole
YPL221W Vacuole
YPL232W Vacuole
YPL234C Vacuole
YPL236C Vacuole
YPL265W Vacuole
YPR026W Vacuole
YPR036W Vacuole
YPR049C Vacuole
YPR075C Vacuole
YPR091C Vacuole
YPR156C Vacuole
Q0045 Mitochondrion
Q0050 Mitochondrion
Q0055 Mitochondrion
Q0060 Mitochondrion
Q0065 Mitochondrion
Q0070 Mitochondrion
Q0075 Mitochondrion
Q0080 Mitochondrion
Q0085 Mitochondrion
Q0105 Mitochondrion
Q0110 Mitochondrion
Q0115 Mitochondrion
Q0120 Mitochondrion
Q0130 Mitochondrion

Q0140 Mitochondrion
Q0160 Mitochondrion
Q0250 Mitochondrion
Q0255 Mitochondrion
Q0275 Mitochondrion
YAL001C Mitochondrion
YAL008W Mitochondrion
YAL010C Mitochondrion
YAL011W Mitochondrion
YAL015C Mitochondrion
YAL019W Mitochondrion
YAL029C Mitochondrion
YAL035W Mitochondrion
YAL039C Mitochondrion
YAL044C Mitochondrion
YAL048C Mitochondrion
YAL054C Mitochondrion
YAL056W Mitochondrion
YAL062W Mitochondrion
YAR002C-A Mitochondrion
YAR008W Mitochondrion
YAR035W Mitochondrion
YBL013W Mitochondrion
YBL015W Mitochondrion
YBL016W Mitochondrion
YBL022C Mitochondrion
YBL030C Mitochondrion
YBL038W Mitochondrion
YBL045C Mitochondrion
YBL057C Mitochondrion
YBL059C-A Mitochondrion
YBL059W Mitochondrion
YBL064C Mitochondrion
YBL080C Mitochondrion
YBL088C Mitochondrion
YBL090W Mitochondrion
YBL095W Mitochondrion
YBL098W Mitochondrion
YBL099W Mitochondrion
YBL107C Mitochondrion
YBL111C Mitochondrion
YBR001C Mitochondrion
YBR003W Mitochondrion
YBR004C Mitochondrion
YBR024W Mitochondrion

YBR026C Mitochondrion
YBR035C Mitochondrion
YBR037C Mitochondrion
YBR039W Mitochondrion
YBR044C Mitochondrion
YBR047W Mitochondrion
YBR054W Mitochondrion
YBR056W Mitochondrion
YBR078W Mitochondrion
YBR081C Mitochondrion
YBR084W Mitochondrion
YBR085W Mitochondrion
YBR091C Mitochondrion
YBR097W Mitochondrion
YBR104W Mitochondrion
YBR106W Mitochondrion
YBR111C Mitochondrion
YBR120C Mitochondrion
YBR121C Mitochondrion
YBR122C Mitochondrion
YBR129C Mitochondrion
YBR136W Mitochondrion
YBR140C Mitochondrion
YBR146W Mitochondrion
YBR147W Mitochondrion
YBR150C Mitochondrion
YBR163W Mitochondrion
YBR170C Mitochondrion
YBR176W Mitochondrion
YBR177C Mitochondrion
YBR179C Mitochondrion
YBR185C Mitochondrion
YBR192W Mitochondrion
YBR196C Mitochondrion
YBR200W Mitochondrion
YBR203W Mitochondrion
YBR221C Mitochondrion
YBR227C Mitochondrion
YBR229C Mitochondrion
YBR230C Mitochondrion
YBR238C Mitochondrion
YBR244W Mitochondrion
YBR251W Mitochondrion
YBR262C Mitochondrion
YBR263W Mitochondrion

YBR265W Mitochondrion
YBR268W Mitochondrion
YBR269C Mitochondrion
YBR282W Mitochondrion
YBR291C Mitochondrion
YCL004W Mitochondrion
YCL005W Mitochondrion
YCL009C Mitochondrion
YCL017C Mitochondrion
YCL033C Mitochondrion
YCL044C Mitochondrion
YCL057C-A Mitochondrion
YCL057W Mitochondrion
YCL064C Mitochondrion
YCR003W Mitochondrion
YCR004C Mitochondrion
YCR005C Mitochondrion
YCR008W Mitochondrion
YCR010C Mitochondrion
YCR012W Mitochondrion
YCR024C Mitochondrion
YCR028C-A Mitochondrion
YCR032W Mitochondrion
YCR046C Mitochondrion
YCR071C Mitochondrion
YCR079W Mitochondrion
YCR083W Mitochondrion
YDL003W Mitochondrion
YDL004W Mitochondrion
YDL015C Mitochondrion
YDL027C Mitochondrion
YDL029W Mitochondrion
YDL033C Mitochondrion
YDL036C Mitochondrion
YDL040C Mitochondrion
YDL044C Mitochondrion
YDL045W-A Mitochondrion
YDL048C Mitochondrion
YDL066W Mitochondrion
YDL067C Mitochondrion
YDL069C Mitochondrion
YDL085W Mitochondrion
YDL086W Mitochondrion
YDL104C Mitochondrion
YDL107W Mitochondrion

YDL119C Mitochondrion
YDL120W Mitochondrion
YDL126C Mitochondrion
YDL130W-A Mitochondrion
YDL131W Mitochondrion
YDL140C Mitochondrion
YDL142C Mitochondrion
YDL148C Mitochondrion
YDL149W Mitochondrion
YDL157C Mitochondrion
YDL164C Mitochondrion
YDL168W Mitochondrion
YDL171C Mitochondrion
YDL174C Mitochondrion
YDL178W Mitochondrion
YDL181W Mitochondrion
YDL182W Mitochondrion
YDL183C Mitochondrion
YDL198C Mitochondrion
YDL202W Mitochondrion
YDL203C Mitochondrion
YDL207W Mitochondrion
YDL215C Mitochondrion
YDL217C Mitochondrion
YDL222C Mitochondrion
YDL226C Mitochondrion
YDL230W Mitochondrion
YDL240W Mitochondrion
YDL248W Mitochondrion
YDR011W Mitochondrion
YDR019C Mitochondrion
YDR027C Mitochondrion
YDR031W Mitochondrion
YDR032C Mitochondrion
YDR033W Mitochondrion
YDR035W Mitochondrion
YDR036C Mitochondrion
YDR039C Mitochondrion
YDR041W Mitochondrion
YDR046C Mitochondrion
YDR049W Mitochondrion
YDR050C Mitochondrion
YDR058C Mitochondrion
YDR061W Mitochondrion
YDR065W Mitochondrion

YDR069C Mitochondrion
YDR070C Mitochondrion
YDR074W Mitochondrion
YDR077W Mitochondrion
YDR079W Mitochondrion
YDR096W Mitochondrion
YDR115W Mitochondrion
YDR116C Mitochondrion
YDR119W-A Mitochondrion
YDR120C Mitochondrion
YDR125C Mitochondrion
YDR132C Mitochondrion
YDR141C Mitochondrion
YDR148C Mitochondrion
YDR150W Mitochondrion
YDR155C Mitochondrion
YDR175C Mitochondrion
YDR178W Mitochondrion
YDR185C Mitochondrion
YDR194C Mitochondrion
YDR196C Mitochondrion
YDR197W Mitochondrion
YDR204W Mitochondrion
YDR219C Mitochondrion
YDR226W Mitochondrion
YDR231C Mitochondrion
YDR232W Mitochondrion
YDR233C Mitochondrion
YDR234W Mitochondrion
YDR236C Mitochondrion
YDR237W Mitochondrion
YDR256C Mitochondrion
YDR258C Mitochondrion
YDR263C Mitochondrion
YDR268W Mitochondrion
YDR282C Mitochondrion
YDR296W Mitochondrion
YDR298C Mitochondrion
YDR301W Mitochondrion
YDR305C Mitochondrion
YDR316W Mitochondrion
YDR322C-A Mitochondrion
YDR322W Mitochondrion
YDR326C Mitochondrion
YDR332W Mitochondrion

YDR337W Mitochondrion
YDR341C Mitochondrion
YDR342C Mitochondrion
YDR343C Mitochondrion
YDR347W Mitochondrion
YDR350C Mitochondrion
YDR353W Mitochondrion
YDR375C Mitochondrion
YDR376W Mitochondrion
YDR377W Mitochondrion
YDR379C-A Mitochondrion
YDR381C-A Mitochondrion
YDR384C Mitochondrion
YDR393W Mitochondrion
YDR405W Mitochondrion
YDR419W Mitochondrion
YDR430C Mitochondrion
YDR438W Mitochondrion
YDR450W Mitochondrion
YDR462W Mitochondrion
YDR470C Mitochondrion
YDR477W Mitochondrion
YDR487C Mitochondrion
YDR493W Mitochondrion
YDR494W Mitochondrion
YDR505C Mitochondrion
YDR508C Mitochondrion
YDR511W Mitochondrion
YDR513W Mitochondrion
YDR514C Mitochondrion
YDR529C Mitochondrion
YDR538W Mitochondrion
YEL006W Mitochondrion
YEL020W-A Mitochondrion
YEL024W Mitochondrion
YEL030W Mitochondrion
YEL031W Mitochondrion
YEL034W Mitochondrion
YEL037C Mitochondrion
YEL039C Mitochondrion
YEL047C Mitochondrion
YEL050C Mitochondrion
YEL052W Mitochondrion
YEL059C-A Mitochondrion
YEL061C Mitochondrion

YEL063C Mitochondrion
YEL067C Mitochondrion
YER004W Mitochondrion
YER014W Mitochondrion
YER015W Mitochondrion
YER017C Mitochondrion
YER019W Mitochondrion
YER020W Mitochondrion
YER026C Mitochondrion
YER031C Mitochondrion
YER033C Mitochondrion
YER038W-A Mitochondrion
YER048W-A Mitochondrion
YER050C Mitochondrion
YER053C Mitochondrion
YER057C Mitochondrion
YER058W Mitochondrion
YER061C Mitochondrion
YER069W Mitochondrion
YER073W Mitochondrion
YER074W Mitochondrion
YER076C Mitochondrion
YER077C Mitochondrion
YER078C Mitochondrion
YER080W Mitochondrion
YER086W Mitochondrion
YER087W Mitochondrion
YER115C Mitochondrion
YER125W Mitochondrion
YER140W Mitochondrion
YER141W Mitochondrion
YER153C Mitochondrion
YER154W Mitochondrion
YER155C Mitochondrion
YER164W Mitochondrion
YER166W Mitochondrion
YER168C Mitochondrion
YER170W Mitochondrion
YER178W Mitochondrion
YER182W Mitochondrion
YER183C Mitochondrion
YFL005W Mitochondrion
YFL010C Mitochondrion
YFL011W Mitochondrion
YFL016C Mitochondrion

YFL018C Mitochondrion
YFL030W Mitochondrion
YFL036W Mitochondrion
YFL038C Mitochondrion
YFL046W Mitochondrion
YFR004W Mitochondrion
YFR011C Mitochondrion
YFR015C Mitochondrion
YFR019W Mitochondrion
YFR024C-A Mitochondrion
YFR031C Mitochondrion
YFR033C Mitochondrion
YFR044C Mitochondrion
YFR045W Mitochondrion
YFR049W Mitochondrion
YFR053C Mitochondrion
YGL002W Mitochondrion
YGL005C Mitochondrion
YGL008C Mitochondrion
YGL011C Mitochondrion
YGL018C Mitochondrion
YGL020C Mitochondrion
YGL023C Mitochondrion
YGL041W-A Mitochondrion
YGL057C Mitochondrion
YGL059W Mitochondrion
YGL063W Mitochondrion
YGL064C Mitochondrion
YGL068W Mitochondrion
YGL073W Mitochondrion
YGL080W Mitochondrion
YGL085W Mitochondrion
YGL093W Mitochondrion
YGL104C Mitochondrion
YGL107C Mitochondrion
YGL119W Mitochondrion
YGL120C Mitochondrion
YGL125W Mitochondrion
YGL129C Mitochondrion
YGL136C Mitochondrion
YGL139W Mitochondrion
YGL143C Mitochondrion
YGL151W Mitochondrion
YGL187C Mitochondrion
YGL191W Mitochondrion

YGL195W Mitochondrion
YGL197W Mitochondrion
YGL209W Mitochondrion
YGL210W Mitochondrion
YGL211W Mitochondrion
YGL219C Mitochondrion
YGL221C Mitochondrion
YGL225W Mitochondrion
YGL226W Mitochondrion
YGL228W Mitochondrion
YGL229C Mitochondrion
YGL230C Mitochondrion
YGL236C Mitochondrion
YGL240W Mitochondrion
YGL245W Mitochondrion
YGL249W Mitochondrion
YGL253W Mitochondrion
YGL256W Mitochondrion
YGR008C Mitochondrion
YGR012W Mitochondrion
YGR015C Mitochondrion
YGR021W Mitochondrion
YGR028W Mitochondrion
YGR029W Mitochondrion
YGR031W Mitochondrion
YGR033C Mitochondrion
YGR046W Mitochondrion
YGR049W Mitochondrion
YGR052W Mitochondrion
YGR062C Mitochondrion
YGR076C Mitochondrion
YGR082W Mitochondrion
YGR084C Mitochondrion
YGR086C Mitochondrion
YGR091W Mitochondrion
YGR094W Mitochondrion
YGR096W Mitochondrion
YGR098C Mitochondrion
YGR101W Mitochondrion
YGR102C Mitochondrion
YGR110W Mitochondrion
YGR112W Mitochondrion
YGR116W Mitochondrion
YGR132C Mitochondrion
YGR147C Mitochondrion

YGR150C Mitochondrion
YGR155W Mitochondrion
YGR159C Mitochondrion
YGR162W Mitochondrion
YGR165W Mitochondrion
YGR169C Mitochondrion
YGR171C Mitochondrion
YGR174C Mitochondrion
YGR178C Mitochondrion
YGR181W Mitochondrion
YGR183C Mitochondrion
YGR192C Mitochondrion
YGR193C Mitochondrion
YGR207C Mitochondrion
YGR215W Mitochondrion
YGR220C Mitochondrion
YGR222W Mitochondrion
YGR231C Mitochondrion
YGR234W Mitochondrion
YGR235C Mitochondrion
YGR236C Mitochondrion
YGR240C Mitochondrion
YGR243W Mitochondrion
YGR244C Mitochondrion
YGR254W Mitochondrion
YGR255C Mitochondrion
YGR257C Mitochondrion
YGR260W Mitochondrion
YGR266W Mitochondrion
YGR285C Mitochondrion
YGR286C Mitochondrion
YGR287C Mitochondrion
YGR296W Mitochondrion
YHL004W Mitochondrion
YHL014C Mitochondrion
YHL018W Mitochondrion
YHL021C Mitochondrion
YHL032C Mitochondrion
YHL035C Mitochondrion
YHL038C Mitochondrion
YHR001W-A Mitochondrion
YHR002W Mitochondrion
YHR003C Mitochondrion
YHR004C Mitochondrion
YHR005C-A Mitochondrion

YHR008C Mitochondrion
YHR011W Mitochondrion
YHR017W Mitochondrion
YHR024C Mitochondrion
YHR037W Mitochondrion
YHR038W Mitochondrion
YHR042W Mitochondrion
YHR050W Mitochondrion
YHR051W Mitochondrion
YHR059W Mitochondrion
YHR067W Mitochondrion
YHR070W Mitochondrion
YHR075C Mitochondrion
YHR076W Mitochondrion
YHR080C Mitochondrion
YHR083W Mitochondrion
YHR086W Mitochondrion
YHR091C Mitochondrion
YHR100C Mitochondrion
YHR106W Mitochondrion
YHR116W Mitochondrion
YHR117W Mitochondrion
YHR120W Mitochondrion
YHR135C Mitochondrion
YHR147C Mitochondrion
YHR155W Mitochondrion
YHR162W Mitochondrion
YHR168W Mitochondrion
YHR171W Mitochondrion
YHR174W Mitochondrion
YHR179W Mitochondrion
YHR183W Mitochondrion
YHR186C Mitochondrion
YHR189W Mitochondrion
YHR190W Mitochondrion
YHR194W Mitochondrion
YHR198C Mitochondrion
YHR199C Mitochondrion
YHR201C Mitochondrion
YHR208W Mitochondrion
YIL006W Mitochondrion
YIL016W Mitochondrion
YIL022W Mitochondrion
YIL042C Mitochondrion
YIL043C Mitochondrion

YIL047C Mitochondrion
YIL051C Mitochondrion
YIL055C Mitochondrion
YIL060W Mitochondrion
YIL062C Mitochondrion
YIL065C Mitochondrion
YIL066C Mitochondrion
YIL069C Mitochondrion
YIL070C Mitochondrion
YIL077C Mitochondrion
YIL078W Mitochondrion
YIL087C Mitochondrion
YIL093C Mitochondrion
YIL094C Mitochondrion
YIL098C Mitochondrion
YIL105C Mitochondrion
YIL111W Mitochondrion
YIL114C Mitochondrion
YIL124W Mitochondrion
YIL125W Mitochondrion
YIL129C Mitochondrion
YIL134W Mitochondrion
YIL136W Mitochondrion
YIL139C Mitochondrion
YIL146C Mitochondrion
YIL148W Mitochondrion
YIL149C Mitochondrion
YIL155C Mitochondrion
YIL157C Mitochondrion
YIL160C Mitochondrion
YIL162W Mitochondrion
YIR021W Mitochondrion
YIR024C Mitochondrion
YIR037W Mitochondrion
YIR038C Mitochondrion
YJL003W Mitochondrion
YJL005W Mitochondrion
YJL023C Mitochondrion
YJL043W Mitochondrion
YJL045W Mitochondrion
YJL046W Mitochondrion
YJL052W Mitochondrion
YJL054W Mitochondrion
YJL060W Mitochondrion
YJL062W-A Mitochondrion

YJL063C Mitochondrion
YJL066C Mitochondrion
YJL070C Mitochondrion
YJL071W Mitochondrion
YJL082W Mitochondrion
YJL094C Mitochondrion
YJL096W Mitochondrion
YJL098W Mitochondrion
YJL102W Mitochondrion
YJL104W Mitochondrion
YJL109C Mitochondrion
YJL112W Mitochondrion
YJL113W Mitochondrion
YJL116C Mitochondrion
YJL130C Mitochondrion
YJL131C Mitochondrion
YJL133C-A Mitochondrion
YJL133W Mitochondrion
YJL143W Mitochondrion
YJL147C Mitochondrion
YJL161W Mitochondrion
YJL166W Mitochondrion
YJL171C Mitochondrion
YJL178C Mitochondrion
YJL180C Mitochondrion
YJL200C Mitochondrion
YJL208C Mitochondrion
YJL209W Mitochondrion
YJR003C Mitochondrion
YJR009C Mitochondrion
YJR010W Mitochondrion
YJR016C Mitochondrion
YJR019C Mitochondrion
YJR034W Mitochondrion
YJR039W Mitochondrion
YJR045C Mitochondrion
YJR048W Mitochondrion
YJR051W Mitochondrion
YJR060W Mitochondrion
YJR062C Mitochondrion
YJR073C Mitochondrion
YJR077C Mitochondrion
YJR080C Mitochondrion
YJR085C Mitochondrion
YJR089W Mitochondrion

YJR091C Mitochondrion
YJR095W Mitochondrion
YJR098C Mitochondrion
YJR100C Mitochondrion
YJR101W Mitochondrion
YJR104C Mitochondrion
YJR111C Mitochondrion
YJR113C Mitochondrion
YJR117W Mitochondrion
YJR121W Mitochondrion
YJR122W Mitochondrion
YJR135W-A Mitochondrion
YJR144W Mitochondrion
YKL003C Mitochondrion
YKL010C Mitochondrion
YKL011C Mitochondrion
YKL016C Mitochondrion
YKL026C Mitochondrion
YKL027W Mitochondrion
YKL028W Mitochondrion
YKL029C Mitochondrion
YKL033W Mitochondrion
YKL037W Mitochondrion
YKL040C Mitochondrion
YKL053C-A Mitochondrion
YKL055C Mitochondrion
YKL056C Mitochondrion
YKL060C Mitochondrion
YKL067W Mitochondrion
YKL070W Mitochondrion
YKL081W Mitochondrion
YKL084W Mitochondrion
YKL085W Mitochondrion
YKL087C Mitochondrion
YKL093W Mitochondrion
YKL094W Mitochondrion
YKL106W Mitochondrion
YKL113C Mitochondrion
YKL114C Mitochondrion
YKL120W Mitochondrion
YKL132C Mitochondrion
YKL134C Mitochondrion
YKL137W Mitochondrion
YKL138C Mitochondrion
YKL141W Mitochondrion

YKL148C Mitochondrion
YKL150W Mitochondrion
YKL152C Mitochondrion
YKL155C Mitochondrion
YKL157W Mitochondrion
YKL162C Mitochondrion
YKL167C Mitochondrion
YKL170W Mitochondrion
YKL182W Mitochondrion
YKL187C Mitochondrion
YKL192C Mitochondrion
YKL194C Mitochondrion
YKL195W Mitochondrion
YKL196C Mitochondrion
YKL203C Mitochondrion
YKL205W Mitochondrion
YKL208W Mitochondrion
YKL212W Mitochondrion
YKL217W Mitochondrion
YKR001C Mitochondrion
YKR005C Mitochondrion
YKR006C Mitochondrion
YKR010C Mitochondrion
YKR016W Mitochondrion
YKR018C Mitochondrion
YKR019C Mitochondrion
YKR023W Mitochondrion
YKR027W Mitochondrion
YKR036C Mitochondrion
YKR042W Mitochondrion
YKR049C Mitochondrion
YKR052C Mitochondrion
YKR063C Mitochondrion
YKR064W Mitochondrion
YKR065C Mitochondrion
YKR066C Mitochondrion
YKR070W Mitochondrion
YKR071C Mitochondrion
YKR079C Mitochondrion
YKR085C Mitochondrion
YKR087C Mitochondrion
YKR094C Mitochondrion
YLL001W Mitochondrion
YLL006W Mitochondrion
YLL009C Mitochondrion

YLL013C Mitochondrion
YLL018C-A Mitochondrion
YLL024C Mitochondrion
YLL027W Mitochondrion
YLL036C Mitochondrion
YLL040C Mitochondrion
YLL041C Mitochondrion
YLL060C Mitochondrion
YLR001C Mitochondrion
YLR008C Mitochondrion
YLR038C Mitochondrion
YLR043C Mitochondrion
YLR059C Mitochondrion
YLR067C Mitochondrion
YLR069C Mitochondrion
YLR077W Mitochondrion
YLR084C Mitochondrion
YLR087C Mitochondrion
YLR089C Mitochondrion
YLR090W Mitochondrion
YLR091W Mitochondrion
YLR099W-A Mitochondrion
YLR100W Mitochondrion
YLR105C Mitochondrion
YLR106C Mitochondrion
YLR132C Mitochondrion
YLR139C Mitochondrion
YLR142W Mitochondrion
YLR154W-C Mitochondrion
YLR163C Mitochondrion
YLR164W Mitochondrion
YLR165C Mitochondrion
YLR167W Mitochondrion
YLR168C Mitochondrion
YLR183C Mitochondrion
YLR188W Mitochondrion
YLR190W Mitochondrion
YLR193C Mitochondrion
YLR201C Mitochondrion
YLR203C Mitochondrion
YLR204W Mitochondrion
YLR218C Mitochondrion
YLR239C Mitochondrion
YLR247C Mitochondrion
YLR251W Mitochondrion

YLR253W Mitochondrion
YLR259C Mitochondrion
YLR270W Mitochondrion
YLR281C Mitochondrion
YLR283W Mitochondrion
YLR289W Mitochondrion
YLR290C Mitochondrion
YLR291C Mitochondrion
YLR295C Mitochondrion
YLR304C Mitochondrion
YLR305C Mitochondrion
YLR312W-A Mitochondrion
YLR327C Mitochondrion
YLR335W Mitochondrion
YLR342W Mitochondrion
YLR346C Mitochondrion
YLR348C Mitochondrion
YLR351C Mitochondrion
YLR355C Mitochondrion
YLR356W Mitochondrion
YLR368W Mitochondrion
YLR369W Mitochondrion
YLR370C Mitochondrion
YLR380W Mitochondrion
YLR382C Mitochondrion
YLR383W Mitochondrion
YLR390W Mitochondrion
YLR390W-A Mitochondrion
YLR393W Mitochondrion
YLR395C Mitochondrion
YLR415C Mitochondrion
YLR419W Mitochondrion
YLR424W Mitochondrion
YLR426W Mitochondrion
YLR439W Mitochondrion
YLR442C Mitochondrion
YLR454W Mitochondrion
YML001W Mitochondrion
YML007C-A Mitochondrion
YML008C Mitochondrion
YML009C Mitochondrion
YML010W Mitochondrion
YML013W Mitochondrion
YML021C Mitochondrion
YML025C Mitochondrion

YML026C Mitochondrion
YML030W Mitochondrion
YML042W Mitochondrion
YML048W Mitochondrion
YML052W Mitochondrion
YML054C Mitochondrion
YML060W Mitochondrion
YML061C Mitochondrion
YML072C Mitochondrion
YML076C Mitochondrion
YML078W Mitochondrion
YML081C-A Mitochondrion
YML086C Mitochondrion
YML091C Mitochondrion
YML110C Mitochondrion
YML120C Mitochondrion
YML128C Mitochondrion
YML129C Mitochondrion
YML133C Mitochondrion
YMR002W Mitochondrion
YMR003W Mitochondrion
YMR023C Mitochondrion
YMR024W Mitochondrion
YMR030W Mitochondrion
YMR031C Mitochondrion
YMR035W Mitochondrion
YMR038C Mitochondrion
YMR056C Mitochondrion
YMR059W Mitochondrion
YMR060C Mitochondrion
YMR061W Mitochondrion
YMR062C Mitochondrion
YMR064W Mitochondrion
YMR065W Mitochondrion
YMR066W Mitochondrion
YMR072W Mitochondrion
YMR078C Mitochondrion
YMR083W Mitochondrion
YMR089C Mitochondrion
YMR097C Mitochondrion
YMR098C Mitochondrion
YMR108W Mitochondrion
YMR110C Mitochondrion
YMR115W Mitochondrion
YMR118C Mitochondrion

YMR128W Mitochondrion
YMR129W Mitochondrion
YMR145C Mitochondrion
YMR150C Mitochondrion
YMR152W Mitochondrion
YMR157C Mitochondrion
YMR158W Mitochondrion
YMR166C Mitochondrion
YMR167W Mitochondrion
YMR177W Mitochondrion
YMR186W Mitochondrion
YMR188C Mitochondrion
YMR189W Mitochondrion
YMR192W Mitochondrion
YMR193W Mitochondrion
YMR194C-B Mitochondrion
YMR203W Mitochondrion
YMR205C Mitochondrion
YMR207C Mitochondrion
YMR211W Mitochondrion
YMR212C Mitochondrion
YMR221C Mitochondrion
YMR224C Mitochondrion
YMR225C Mitochondrion
YMR228W Mitochondrion
YMR232W Mitochondrion
YMR241W Mitochondrion
YMR243C Mitochondrion
YMR244C-A Mitochondrion
YMR252C Mitochondrion
YMR256C Mitochondrion
YMR257C Mitochondrion
YMR264W Mitochondrion
YMR267W Mitochondrion
YMR282C Mitochondrion
YMR286W Mitochondrion
YMR287C Mitochondrion
YMR293C Mitochondrion
YMR301C Mitochondrion
YMR302C Mitochondrion
YMR306W Mitochondrion
YMR307W Mitochondrion
YNL003C Mitochondrion
YNL005C Mitochondrion
YNL009W Mitochondrion

YNL026W Mitochondrion
YNL036W Mitochondrion
YNL037C Mitochondrion
YNL052W Mitochondrion
YNL055C Mitochondrion
YNL063W Mitochondrion
YNL066W Mitochondrion
YNL070W Mitochondrion
YNL071W Mitochondrion
YNL073W Mitochondrion
YNL077W Mitochondrion
YNL081C Mitochondrion
YNL083W Mitochondrion
YNL088W Mitochondrion
YNL098C Mitochondrion
YNL100W Mitochondrion
YNL102W Mitochondrion
YNL104C Mitochondrion
YNL112W Mitochondrion
YNL121C Mitochondrion
YNL122C Mitochondrion
YNL125C Mitochondrion
YNL130C Mitochondrion
YNL130C-A Mitochondrion
YNL131W Mitochondrion
YNL135C Mitochondrion
YNL137C Mitochondrion
YNL144C Mitochondrion
YNL168C Mitochondrion
YNL169C Mitochondrion
YNL177C Mitochondrion
YNL185C Mitochondrion
YNL195C Mitochondrion
YNL200C Mitochondrion
YNL208W Mitochondrion
YNL211C Mitochondrion
YNL213C Mitochondrion
YNL227C Mitochondrion
YNL239W Mitochondrion
YNL249C Mitochondrion
YNL250W Mitochondrion
YNL252C Mitochondrion
YNL256W Mitochondrion
YNL268W Mitochondrion
YNL274C Mitochondrion

YNL284C Mitochondrion
YNL292W Mitochondrion
YNL304W Mitochondrion
YNL305C Mitochondrion
YNL306W Mitochondrion
YNL310C Mitochondrion
YNL315C Mitochondrion
YNL318C Mitochondrion
YNL320W Mitochondrion
YNL328C Mitochondrion
YNR001C Mitochondrion
YNR002C Mitochondrion
YNR003C Mitochondrion
YNR016C Mitochondrion
YNR017W Mitochondrion
YNR018W Mitochondrion
YNR020C Mitochondrion
YNR022C Mitochondrion
YNR036C Mitochondrion
YNR037C Mitochondrion
YNR040W Mitochondrion
YNR041C Mitochondrion
YNR045W Mitochondrion
YNR055C Mitochondrion
YNR070W Mitochondrion
YNR074C Mitochondrion
YOL008W Mitochondrion
YOL009C Mitochondrion
YOL021C Mitochondrion
YOL023W Mitochondrion
YOL025W Mitochondrion
YOL026C Mitochondrion
YOL027C Mitochondrion
YOL033W Mitochondrion
YOL038W Mitochondrion
YOL042W Mitochondrion
YOL045W Mitochondrion
YOL053W Mitochondrion
YOL059W Mitochondrion
YOL071W Mitochondrion
YOL077W-A Mitochondrion
YOL081W Mitochondrion
YOL089C Mitochondrion
YOL095C Mitochondrion
YOL096C Mitochondrion

YOL109W Mitochondrion
YOL129W Mitochondrion
YOL140W Mitochondrion
YOL141W Mitochondrion
YOL143C Mitochondrion
YOR004W Mitochondrion
YOR011W Mitochondrion
YOR017W Mitochondrion
YOR020C Mitochondrion
YOR020W-A Mitochondrion
YOR022C Mitochondrion
YOR037W Mitochondrion
YOR040W Mitochondrion
YOR045W Mitochondrion
YOR048C Mitochondrion
YOR065W Mitochondrion
YOR070C Mitochondrion
YOR086C Mitochondrion
YOR089C Mitochondrion
YOR090C Mitochondrion
YOR100C Mitochondrion
YOR108W Mitochondrion
YOR125C Mitochondrion
YOR130C Mitochondrion
YOR136W Mitochondrion
YOR142W Mitochondrion
YOR144C Mitochondrion
YOR147W Mitochondrion
YOR150W Mitochondrion
YOR151C Mitochondrion
YOR153W Mitochondrion
YOR158W Mitochondrion
YOR168W Mitochondrion
YOR176W Mitochondrion
YOR187W Mitochondrion
YOR188W Mitochondrion
YOR191W Mitochondrion
YOR196C Mitochondrion
YOR201C Mitochondrion
YOR205C Mitochondrion
YOR206W Mitochondrion
YOR211C Mitochondrion
YOR215C Mitochondrion
YOR221C Mitochondrion
YOR222W Mitochondrion

YOR226C Mitochondrion
YOR227W Mitochondrion
YOR228C Mitochondrion
YOR232W Mitochondrion
YOR236W Mitochondrion
YOR241W Mitochondrion
YOR251C Mitochondrion
YOR254C Mitochondrion
YOR266W Mitochondrion
YOR271C Mitochondrion
YOR274W Mitochondrion
YOR285W Mitochondrion
YOR286W Mitochondrion
YOR297C Mitochondrion
YOR298C-A Mitochondrion
YOR305W Mitochondrion
YOR316C Mitochondrion
YOR317W Mitochondrion
YOR330C Mitochondrion
YOR334W Mitochondrion
YOR335C Mitochondrion
YOR346W Mitochondrion
YOR347C Mitochondrion
YOR350C Mitochondrion
YOR354C Mitochondrion
YOR355W Mitochondrion
YOR356W Mitochondrion
YOR374W Mitochondrion
YOR384W Mitochondrion
YOR386W Mitochondrion
YPL004C Mitochondrion
YPL005W Mitochondrion
YPL013C Mitochondrion
YPL029W Mitochondrion
YPL033C Mitochondrion
YPL036W Mitochondrion
YPL040C Mitochondrion
YPL059W Mitochondrion
YPL060W Mitochondrion
YPL061W Mitochondrion
YPL063W Mitochondrion
YPL069C Mitochondrion
YPL072W Mitochondrion
YPL078C Mitochondrion
YPL082C Mitochondrion

YPL083C Mitochondrion
YPL091W Mitochondrion
YPL096W Mitochondrion
YPL097W Mitochondrion
YPL098C Mitochondrion
YPL099C Mitochondrion
YPL103C Mitochondrion
YPL104W Mitochondrion
YPL105C Mitochondrion
YPL107W Mitochondrion
YPL109C Mitochondrion
YPL118W Mitochondrion
YPL132W Mitochondrion
YPL134C Mitochondrion
YPL135W Mitochondrion
YPL137C Mitochondrion
YPL148C Mitochondrion
YPL154C Mitochondrion
YPL155C Mitochondrion
YPL159C Mitochondrion
YPL167C Mitochondrion
YPL168W Mitochondrion
YPL172C Mitochondrion
YPL173W Mitochondrion
YPL183W-A Mitochondrion
YPL186C Mitochondrion
YPL188W Mitochondrion
YPL189C-A Mitochondrion
YPL196W Mitochondrion
YPL202C Mitochondrion
YPL206C Mitochondrion
YPL215W Mitochondrion
YPL217C Mitochondrion
YPL222W Mitochondrion
YPL224C Mitochondrion
YPL226W Mitochondrion
YPL231W Mitochondrion
YPL252C Mitochondrion
YPL262W Mitochondrion
YPL270W Mitochondrion
YPL271W Mitochondrion
YPL283C Mitochondrion
YPR001W Mitochondrion
YPR002W Mitochondrion
YPR004C Mitochondrion

YPR006C Mitochondrion
YPR011C Mitochondrion
YPR020W Mitochondrion
YPR021C Mitochondrion
YPR024W Mitochondrion
YPR025C Mitochondrion
YPR033C Mitochondrion
YPR047W Mitochondrion
YPR048W Mitochondrion
YPR054W Mitochondrion
YPR058W Mitochondrion
YPR061C Mitochondrion
YPR067W Mitochondrion
YPR080W Mitochondrion
YPR083W Mitochondrion
YPR095C Mitochondrion
YPR097W Mitochondrion
YPR098C Mitochondrion
YPR100W Mitochondrion
YPR113W Mitochondrion
YPR116W Mitochondrion
YPR125W Mitochondrion
YPR133W-A Mitochondrion
YPR134W Mitochondrion
YPR140W Mitochondrion
YPR149W Mitochondrion
YPR151C Mitochondrion
YPR155C Mitochondrion
YPR165W Mitochondrion
YPR166C Mitochondrion
YPR183W Mitochondrion
YPR184W Mitochondrion
YPR191W Mitochondrion
YAL002W Endosome
YAL014C Endosome
YAL030W Endosome
YAR042W Endosome
YBL007C Endosome
YBL017C Endosome
YBL069W Endosome
YBR077C Endosome
YBR097W Endosome
YBR131W Endosome
YBR246W Endosome
YCL008C Endosome

YCR068W Endosome
YCR075C Endosome
YCR094W Endosome
YDL113C Endosome
YDL226C Endosome
YDR027C Endosome
YDR069C Endosome
YDR080W Endosome
YDR141C Endosome
YDR153C Endosome
YDR170C Endosome
YDR202C Endosome
YDR264C Endosome
YDR313C Endosome
YDR323C Endosome
YDR407C Endosome
YDR425W Endosome
YDR456W Endosome
YDR468C Endosome
YDR484W Endosome
YDR486C Endosome
YDR495C Endosome
YDR524C Endosome
YEL063C Endosome
YEL065W Endosome
YER031C Endosome
YER125W Endosome
YER128W Endosome
YER166W Endosome
YFL014W Endosome
YFR019W Endosome
YFR021W Endosome
YGL079W Endosome
YGL124C Endosome
YGL210W Endosome
YGR142W Endosome
YGR163W Endosome
YGR170W Endosome
YGR198W Endosome
YGR206W Endosome
YGR223C Endosome
YHL002W Endosome
YHL040C Endosome
YHL047C Endosome
YHR005C Endosome

YHR009C Endosome
YHR012W Endosome
YHR105W Endosome
YIL048W Endosome
YIL173W Endosome
YIR006C Endosome
YJL029C Endosome
YJL036W Endosome
YJL044C Endosome
YJL053W Endosome
YJL084C Endosome
YJL151C Endosome
YJL154C Endosome
YJL204C Endosome
YJR040W Endosome
YJR044C Endosome
YJR066W Endosome
YJR102C Endosome
YJR125C Endosome
YKL002W Endosome
YKL039W Endosome
YKL041W Endosome
YKL061W Endosome
YKL124W Endosome
YKL143W Endosome
YKL196C Endosome
YKR001C Endosome
YKR007W Endosome
YKR014C Endosome
YKR020W Endosome
YKR021W Endosome
YKR031C Endosome
YKR035W-A Endosome
YKR039W Endosome
YLR025W Endosome
YLR073C Endosome
YLR083C Endosome
YLR119W Endosome
YLR148W Endosome
YLR181C Endosome
YLR240W Endosome
YLR360W Endosome
YLR380W Endosome
YLR396C Endosome
YLR408C Endosome

YLR417W Endosome
YML121W Endosome
YMR054W Endosome
YMR077C Endosome
YMR110C Endosome
YMR171C Endosome
YMR197C Endosome
YMR218C Endosome
YMR231W Endosome
YNL006W Endosome
YNL084C Endosome
YNL086W Endosome
YNL093W Endosome
YNL264C Endosome
YNL265C Endosome
YNL287W Endosome
YNL293W Endosome
YNL297C Endosome
YNR006W Endosome
YOL018C Endosome
YOL129W Endosome
YOL158C Endosome
YOR036W Endosome
YOR069W Endosome
YOR089C Endosome
YOR132W Endosome
YOR171C Endosome
YOR327C Endosome
YOR357C Endosome
YPL002C Endosome
YPL045W Endosome
YPL065W Endosome
YPL084W Endosome
YPL100W Endosome
YPL120W Endosome
YPL170W Endosome
YPL183C Endosome
YPR079W Endosome
YPR165W Endosome
YPR173C Endosome
YAL005C Plasma
YAL030W Plasma
YAL038W Plasma
YAL056W Plasma
YAR027W Plasma

YAR031W Plasma
YAR033W Plasma
YAR050W Plasma
YAR066W Plasma
YBL007C Plasma
YBL029C-A Plasma
YBL037W Plasma
YBL042C Plasma
YBL060W Plasma
YBL061C Plasma
YBL069W Plasma
YBL085W Plasma
YBL105C Plasma
YBL106C Plasma
YBR008C Plasma
YBR016W Plasma
YBR021W Plasma
YBR041W Plasma
YBR043C Plasma
YBR068C Plasma
YBR069C Plasma
YBR078W Plasma
YBR086C Plasma
YBR132C Plasma
YBR140C Plasma
YBR196C Plasma
YBR207W Plasma
YBR264C Plasma
YBR294W Plasma
YBR295W Plasma
YBR296C Plasma
YBR298C Plasma
YBR299W Plasma
YCL008C Plasma
YCL024W Plasma
YCL025C Plasma
YCL027W Plasma
YCL040W Plasma
YCL048W Plasma
YCL048W-A Plasma
YCL058C Plasma
YCL073C Plasma
YCR004C Plasma
YCR010C Plasma
YCR012W Plasma

YCR017C Plasma
YCR021C Plasma
YCR024C-A Plasma
YCR027C Plasma
YCR028C Plasma
YCR037C Plasma
YCR098C Plasma
YDL012C Plasma
YDL019C Plasma
YDL035C Plasma
YDL124W Plasma
YDL135C Plasma
YDL138W Plasma
YDL194W Plasma
YDL222C Plasma
YDL223C Plasma
YDL229W Plasma
YDL245C Plasma
YDL247W Plasma
YDR011W Plasma
YDR032C Plasma
YDR033W Plasma
YDR038C Plasma
YDR039C Plasma
YDR040C Plasma
YDR046C Plasma
YDR050C Plasma
YDR055W Plasma
YDR090C Plasma
YDR093W Plasma
YDR099W Plasma
YDR103W Plasma
YDR122W Plasma
YDR129C Plasma
YDR144C Plasma
YDR158W Plasma
YDR160W Plasma
YDR164C Plasma
YDR208W Plasma
YDR210W Plasma
YDR212W Plasma
YDR261C Plasma
YDR276C Plasma
YDR309C Plasma
YDR342C Plasma

YDR343C Plasma
YDR345C Plasma
YDR348C Plasma
YDR373W Plasma
YDR384C Plasma
YDR420W Plasma
YDR459C Plasma
YDR461W Plasma
YDR463W Plasma
YDR497C Plasma
YDR508C Plasma
YDR522C Plasma
YDR524C-B Plasma
YDR536W Plasma
YDR539W Plasma
YEL017C-A Plasma
YEL047C Plasma
YEL063C Plasma
YEL069C Plasma
YER020W Plasma
YER056C Plasma
YER060W Plasma
YER060W-A Plasma
YER067W Plasma
YER091C Plasma
YER093C Plasma
YER118C Plasma
YER123W Plasma
YER125W Plasma
YER143W Plasma
YER145C Plasma
YER155C Plasma
YER166W Plasma
YER177W Plasma
YER185W Plasma
YFL005W Plasma
YFL011W Plasma
YFL014W Plasma
YFL026W Plasma
YFL041W Plasma
YFL050C Plasma
YFL051C Plasma
YFL055W Plasma
YFR029W Plasma
YGL008C Plasma

YGL045W Plasma
YGL051W Plasma
YGL053W Plasma
YGL077C Plasma
YGL082W Plasma
YGL084C Plasma
YGL100W Plasma
YGL108C Plasma
YGL115W Plasma
YGL186C Plasma
YGL206C Plasma
YGL208W Plasma
YGL233W Plasma
YGL255W Plasma
YGR009C Plasma
YGR014W Plasma
YGR023W Plasma
YGR026W Plasma
YGR031C-A Plasma
YGR032W Plasma
YGR041W Plasma
YGR055W Plasma
YGR060W Plasma
YGR065C Plasma
YGR086C Plasma
YGR121C Plasma
YGR122W Plasma
YGR131W Plasma
YGR138C Plasma
YGR152C Plasma
YGR167W Plasma
YGR191W Plasma
YGR192C Plasma
YGR197C Plasma
YGR198W Plasma
YGR213C Plasma
YGR217W Plasma
YGR221C Plasma
YGR224W Plasma
YGR241C Plasma
YGR254W Plasma
YGR256W Plasma
YGR260W Plasma
YGR266W Plasma
YGR281W Plasma

YGR289C Plasma
YGR292W Plasma
YHL007C Plasma
YHL016C Plasma
YHL040C Plasma
YHL044W Plasma
YHL047C Plasma
YHR005C Plasma
YHR006W Plasma
YHR042W Plasma
YHR048W Plasma
YHR092C Plasma
YHR094C Plasma
YHR096C Plasma
YHR114W Plasma
YHR126C Plasma
YHR135C Plasma
YHR161C Plasma
YHR174W Plasma
YHR186C Plasma
YHR201C Plasma
YHR214W Plasma
YIL033C Plasma
YIL034C Plasma
YIL047C Plasma
YIL088C Plasma
YIL105C Plasma
YIL118W Plasma
YIL120W Plasma
YIL121W Plasma
YIL140W Plasma
YIL147C Plasma
YIR006C Plasma
YIR019C Plasma
YIR038C Plasma
YIR039C Plasma
YJL005W Plasma
YJL052W Plasma
YJL058C Plasma
YJL062W Plasma
YJL085W Plasma
YJL093C Plasma
YJL100W Plasma
YJL129C Plasma
YJL138C Plasma

YJL145W Plasma
YJL156C Plasma
YJL158C Plasma
YJL170C Plasma
YJL171C Plasma
YJL198W Plasma
YJL212C Plasma
YJL214W Plasma
YJL219W Plasma
YJR005W Plasma
YJR009C Plasma
YJR040W Plasma
YJR054W Plasma
YJR058C Plasma
YJR059W Plasma
YJR065C Plasma
YJR066W Plasma
YJR086W Plasma
YJR152W Plasma
YJR158W Plasma
YJR160C Plasma
YKL007W Plasma
YKL035W Plasma
YKL046C Plasma
YKL051W Plasma
YKL092C Plasma
YKL094W Plasma
YKL105C Plasma
YKL126W Plasma
YKL135C Plasma
YKL178C Plasma
YKL196C Plasma
YKL203C Plasma
YKL209C Plasma
YKL217W Plasma
YKL220C Plasma
YKR014C Plasma
YKR039W Plasma
YKR050W Plasma
YKR055W Plasma
YKR059W Plasma
YKR093W Plasma
YKR100C Plasma
YKR105C Plasma
YKR106W Plasma

YLL010C Plasma
YLL024C Plasma
YLL028W Plasma
YLL043W Plasma
YLL050C Plasma
YLL052C Plasma
YLL053C Plasma
YLL055W Plasma
YLL061W Plasma
YLR004C Plasma
YLR019W Plasma
YLR020C Plasma
YLR058C Plasma
YLR081W Plasma
YLR084C Plasma
YLR092W Plasma
YLR096W Plasma
YLR109W Plasma
YLR120C Plasma
YLR121C Plasma
YLR130C Plasma
YLR138W Plasma
YLR187W Plasma
YLR194C Plasma
YLR214W Plasma
YLR219W Plasma
YLR229C Plasma
YLR237W Plasma
YLR262C Plasma
YLR303W Plasma
YLR305C Plasma
YLR310C Plasma
YLR332W Plasma
YLR342W Plasma
YLR343W Plasma
YLR353W Plasma
YLR373C Plasma
YLR411W Plasma
YLR413W Plasma
YLR414C Plasma
YLR432W Plasma
YLR452C Plasma
YML006C Plasma
YML013W Plasma
YML016C Plasma

YML047C Plasma
YML052W Plasma
YML116W Plasma
YML123C Plasma
YML125C Plasma
YML128C Plasma
YML132W Plasma
YMR008C Plasma
YMR011W Plasma
YMR017W Plasma
YMR031C Plasma
YMR058W Plasma
YMR063W Plasma
YMR068W Plasma
YMR086W Plasma
YMR120C Plasma
YMR183C Plasma
YMR186W Plasma
YMR192W Plasma
YMR212C Plasma
YMR215W Plasma
YMR238W Plasma
YMR251W-A Plasma
YMR275C Plasma
YMR306W Plasma
YMR307W Plasma
YMR319C Plasma
YNL006W Plasma
YNL019C Plasma
YNL033W Plasma
YNL047C Plasma
YNL065W Plasma
YNL084C Plasma
YNL087W Plasma
YNL090W Plasma
YNL093W Plasma
YNL098C Plasma
YNL142W Plasma
YNL145W Plasma
YNL154C Plasma
YNL173C Plasma
YNL180C Plasma
YNL183C Plasma
YNL192W Plasma
YNL194C Plasma

YNL209W Plasma
YNL231C Plasma
YNL243W Plasma
YNL257C Plasma
YNL268W Plasma
YNL270C Plasma
YNL271C Plasma
YNL275W Plasma
YNL279W Plasma
YNL283C Plasma
YNL291C Plasma
YNL293W Plasma
YNL294C Plasma
YNL318C Plasma
YNL322C Plasma
YNL323W Plasma
YNR002C Plasma
YNR047W Plasma
YNR049C Plasma
YNR055C Plasma
YNR056C Plasma
YNR060W Plasma
YNR070W Plasma
YNR072W Plasma
YNR074C Plasma
YOL002C Plasma
YOL009C Plasma
YOL011W Plasma
YOL019W Plasma
YOL020W Plasma
YOL062C Plasma
YOL078W Plasma
YOL081W Plasma
YOL084W Plasma
YOL086C Plasma
YOL103W Plasma
YOL109W Plasma
YOL113W Plasma
YOL122C Plasma
YOL130W Plasma
YOL132W Plasma
YOL152W Plasma
YOL156W Plasma
YOL158C Plasma
YOR008C Plasma

YOR011W Plasma
YOR018W Plasma
YOR030W Plasma
YOR047C Plasma
YOR049C Plasma
YOR071C Plasma
YOR086C Plasma
YOR101W Plasma
YOR104W Plasma
YOR107W Plasma
YOR122C Plasma
YOR129C Plasma
YOR153W Plasma
YOR161C Plasma
YOR171C Plasma
YOR188W Plasma
YOR212W Plasma
YOR273C Plasma
YOR301W Plasma
YOR306C Plasma
YOR317W Plasma
YOR322C Plasma
YOR328W Plasma
YOR348C Plasma
YOR371C Plasma
YOR378W Plasma
YOR381W Plasma
YOR384W Plasma
YPL004C Plasma
YPL036W Plasma
YPL056C Plasma
YPL058C Plasma
YPL092W Plasma
YPL176C Plasma
YPL180W Plasma
YPL204W Plasma
YPL232W Plasma
YPL249C Plasma
YPL265W Plasma
YPL274W Plasma
YPR032W Plasma
YPR055W Plasma
YPR075C Plasma
YPR124W Plasma
YPR138C Plasma

YPR149W Plasma
YPR156C Plasma
YPR159W Plasma
YPR165W Plasma
YPR171W Plasma
YPR192W Plasma
YPR194C Plasma
YPR198W Plasma
YPR201W Plasma
R0020C Nucleus
R0030W Nucleus
R0040C Nucleus
YAL001C Nucleus
YAL005C Nucleus
YAL009W Nucleus
YAL011W Nucleus
YAL012W Nucleus
YAL013W Nucleus
YAL015C Nucleus
YAL016W Nucleus
YAL019W Nucleus
YAL021C Nucleus
YAL025C Nucleus
YAL027W Nucleus
YAL032C Nucleus
YAL033W Nucleus
YAL034W-A Nucleus
YAL040C Nucleus
YAL041W Nucleus
YAL043C Nucleus
YAL051W Nucleus
YAL054C Nucleus
YAL059W Nucleus
YAL061W Nucleus
YAL062W Nucleus
YAR002W Nucleus
YAR003W Nucleus
YAR007C Nucleus
YAR008W Nucleus
YAR009C Nucleus
YAR010C Nucleus
YAR014C Nucleus
YAR015W Nucleus
YAR027W Nucleus
YAR042W Nucleus

YBL001C Nucleus
YBL002W Nucleus
YBL003C Nucleus
YBL004W Nucleus
YBL005W Nucleus
YBL005W-A Nucleus
YBL005W-B Nucleus
YBL006C Nucleus
YBL007C Nucleus
YBL008W Nucleus
YBL010C Nucleus
YBL014C Nucleus
YBL016W Nucleus
YBL018C Nucleus
YBL019W Nucleus
YBL021C Nucleus
YBL023C Nucleus
YBL024W Nucleus
YBL025W Nucleus
YBL026W Nucleus
YBL028C Nucleus
YBL029W Nucleus
YBL031W Nucleus
YBL032W Nucleus
YBL033C Nucleus
YBL034C Nucleus
YBL035C Nucleus
YBL041W Nucleus
YBL046W Nucleus
YBL052C Nucleus
YBL054W Nucleus
YBL056W Nucleus
YBL058W Nucleus
YBL059C-A Nucleus
YBL063W Nucleus
YBL066C Nucleus
YBL071W-A Nucleus
YBL074C Nucleus
YBL079W Nucleus
YBL084C Nucleus
YBL087C Nucleus
YBL088C Nucleus
YBL091C Nucleus
YBL093C Nucleus
YBL097W Nucleus

YBL100W-A Nucleus
YBL100W-B Nucleus
YBL103C Nucleus
YBL105C Nucleus
YBR008C Nucleus
YBR009C Nucleus
YBR010W Nucleus
YBR011C Nucleus
YBR012W-A Nucleus
YBR012W-B Nucleus
YBR030W Nucleus
YBR033W Nucleus
YBR034C Nucleus
YBR046C Nucleus
YBR049C Nucleus
YBR055C Nucleus
YBR057C Nucleus
YBR058C Nucleus
YBR060C Nucleus
YBR065C Nucleus
YBR066C Nucleus
YBR070C Nucleus
YBR072W Nucleus
YBR073W Nucleus
YBR081C Nucleus
YBR083W Nucleus
YBR085C-A Nucleus
YBR087W Nucleus
YBR088C Nucleus
YBR089C-A Nucleus
YBR090C Nucleus
YBR095C Nucleus
YBR097W Nucleus
YBR098W Nucleus
YBR103W Nucleus
YBR107C Nucleus
YBR111C Nucleus
YBR111W-A Nucleus
YBR112C Nucleus
YBR114W Nucleus
YBR117C Nucleus
YBR119W Nucleus
YBR123C Nucleus
YBR135W Nucleus
YBR136W Nucleus

YBR141C Nucleus
YBR142W Nucleus
YBR145W Nucleus
YBR150C Nucleus
YBR151W Nucleus
YBR152W Nucleus
YBR154C Nucleus
YBR156C Nucleus
YBR158W Nucleus
YBR160W Nucleus
YBR165W Nucleus
YBR167C Nucleus
YBR170C Nucleus
YBR173C Nucleus
YBR175W Nucleus
YBR181C Nucleus
YBR182C Nucleus
YBR186W Nucleus
YBR188C Nucleus
YBR189W Nucleus
YBR193C Nucleus
YBR195C Nucleus
YBR197C Nucleus
YBR198C Nucleus
YBR202W Nucleus
YBR205W Nucleus
YBR211C Nucleus
YBR215W Nucleus
YBR223C Nucleus
YBR228W Nucleus
YBR231C Nucleus
YBR233W Nucleus
YBR233W-A Nucleus
YBR236C Nucleus
YBR237W Nucleus
YBR239C Nucleus
YBR240C Nucleus
YBR242W Nucleus
YBR244W Nucleus
YBR245C Nucleus
YBR247C Nucleus
YBR249C Nucleus
YBR252W Nucleus
YBR253W Nucleus
YBR257W Nucleus

YBR258C Nucleus
YBR272C Nucleus
YBR273C Nucleus
YBR274W Nucleus
YBR275C Nucleus
YBR276C Nucleus
YBR278W Nucleus
YBR279W Nucleus
YBR281C Nucleus
YBR289W Nucleus
YBR297W Nucleus
YCL010C Nucleus
YCL011C Nucleus
YCL017C Nucleus
YCL019W Nucleus
YCL020W Nucleus
YCL026C-A Nucleus
YCL026C-B Nucleus
YCL028W Nucleus
YCL029C Nucleus
YCL031C Nucleus
YCL035C Nucleus
YCL039W Nucleus
YCL047C Nucleus
YCL050C Nucleus
YCL054W Nucleus
YCL055W Nucleus
YCL058W-A Nucleus
YCL059C Nucleus
YCL061C Nucleus
YCL066W Nucleus
YCL067C Nucleus
YCR014C Nucleus
YCR016W Nucleus
YCR018C Nucleus
YCR020W-B Nucleus
YCR031C Nucleus
YCR033W Nucleus
YCR035C Nucleus
YCR036W Nucleus
YCR039C Nucleus
YCR040W Nucleus
YCR042C Nucleus
YCR045C Nucleus
YCR047C Nucleus

YCR051W Nucleus
YCR052W Nucleus
YCR053W Nucleus
YCR054C Nucleus
YCR057C Nucleus
YCR059C Nucleus
YCR060W Nucleus
YCR063W Nucleus
YCR065W Nucleus
YCR066W Nucleus
YCR072C Nucleus
YCR077C Nucleus
YCR081W Nucleus
YCR082W Nucleus
YCR084C Nucleus
YCR086W Nucleus
YCR087C-A Nucleus
YCR090C Nucleus
YCR092C Nucleus
YCR093W Nucleus
YCR096C Nucleus
YCR097W Nucleus
YCR106W Nucleus
YDL002C Nucleus
YDL003W Nucleus
YDL005C Nucleus
YDL006W Nucleus
YDL007W Nucleus
YDL008W Nucleus
YDL013W Nucleus
YDL014W Nucleus
YDL017W Nucleus
YDL020C Nucleus
YDL022W Nucleus
YDL028C Nucleus
YDL030W Nucleus
YDL031W Nucleus
YDL036C Nucleus
YDL042C Nucleus
YDL043C Nucleus
YDL047W Nucleus
YDL048C Nucleus
YDL051W Nucleus
YDL053C Nucleus
YDL056W Nucleus

YDL059C Nucleus
YDL060W Nucleus
YDL063C Nucleus
YDL064W Nucleus
YDL070W Nucleus
YDL074C Nucleus
YDL076C Nucleus
YDL080C Nucleus
YDL084W Nucleus
YDL085C-A Nucleus
YDL087C Nucleus
YDL088C Nucleus
YDL089W Nucleus
YDL092W Nucleus
YDL098C Nucleus
YDL101C Nucleus
YDL102W Nucleus
YDL103C Nucleus
YDL105W Nucleus
YDL106C Nucleus
YDL108W Nucleus
YDL110C Nucleus
YDL111C Nucleus
YDL115C Nucleus
YDL116W Nucleus
YDL117W Nucleus
YDL124W Nucleus
YDL125C Nucleus
YDL126C Nucleus
YDL127W Nucleus
YDL129W Nucleus
YDL131W Nucleus
YDL132W Nucleus
YDL134C Nucleus
YDL139C Nucleus
YDL140C Nucleus
YDL141W Nucleus
YDL144C Nucleus
YDL147W Nucleus
YDL148C Nucleus
YDL150W Nucleus
YDL153C Nucleus
YDL154W Nucleus
YDL155W Nucleus
YDL156W Nucleus

YDL160C-A Nucleus
YDL164C Nucleus
YDL165W Nucleus
YDL166C Nucleus
YDL170W Nucleus
YDL175C Nucleus
YDL182W Nucleus
YDL188C Nucleus
YDL190C Nucleus
YDL193W Nucleus
YDL197C Nucleus
YDL200C Nucleus
YDL201W Nucleus
YDL204W Nucleus
YDL205C Nucleus
YDL207W Nucleus
YDL208W Nucleus
YDL209C Nucleus
YDL213C Nucleus
YDL216C Nucleus
YDL220C Nucleus
YDL227C Nucleus
YDL233W Nucleus
YDL235C Nucleus
YDL236W Nucleus
YDR002W Nucleus
YDR004W Nucleus
YDR005C Nucleus
YDR006C Nucleus
YDR009W Nucleus
YDR013W Nucleus
YDR014W Nucleus
YDR014W-A Nucleus
YDR016C Nucleus
YDR018C Nucleus
YDR020C Nucleus
YDR021W Nucleus
YDR026C Nucleus
YDR028C Nucleus
YDR030C Nucleus
YDR031W Nucleus
YDR034C Nucleus
YDR034C-C Nucleus
YDR034C-D Nucleus
YDR035W Nucleus

YDR043C Nucleus
YDR045C Nucleus
YDR047W Nucleus
YDR051C Nucleus
YDR052C Nucleus
YDR054C Nucleus
YDR060W Nucleus
YDR063W Nucleus
YDR066C Nucleus
YDR073W Nucleus
YDR075W Nucleus
YDR076W Nucleus
YDR078C Nucleus
YDR079C-A Nucleus
YDR081C Nucleus
YDR082W Nucleus
YDR083W Nucleus
YDR087C Nucleus
YDR088C Nucleus
YDR091C Nucleus
YDR092W Nucleus
YDR096W Nucleus
YDR097C Nucleus
YDR098C Nucleus
YDR098C-A Nucleus
YDR098C-B Nucleus
YDR099W Nucleus
YDR101C Nucleus
YDR103W Nucleus
YDR110W Nucleus
YDR111C Nucleus
YDR113C Nucleus
YDR115W Nucleus
YDR118W Nucleus
YDR120C Nucleus
YDR121W Nucleus
YDR123C Nucleus
YDR130C Nucleus
YDR132C Nucleus
YDR138W Nucleus
YDR140W Nucleus
YDR143C Nucleus
YDR145W Nucleus
YDR146C Nucleus
YDR151C Nucleus

YDR155C Nucleus
YDR156W Nucleus
YDR158W Nucleus
YDR159W Nucleus
YDR161W Nucleus
YDR162C Nucleus
YDR163W Nucleus
YDR165W Nucleus
YDR167W Nucleus
YDR169C Nucleus
YDR170W-A Nucleus
YDR173C Nucleus
YDR174W Nucleus
YDR176W Nucleus
YDR179C Nucleus
YDR180W Nucleus
YDR181C Nucleus
YDR184C Nucleus
YDR190C Nucleus
YDR191W Nucleus
YDR192C Nucleus
YDR195W Nucleus
YDR196C Nucleus
YDR201W Nucleus
YDR205W Nucleus
YDR206W Nucleus
YDR207C Nucleus
YDR208W Nucleus
YDR210C-C Nucleus
YDR210C-D Nucleus
YDR210W-A Nucleus
YDR210W-B Nucleus
YDR213W Nucleus
YDR216W Nucleus
YDR217C Nucleus
YDR223W Nucleus
YDR224C Nucleus
YDR225W Nucleus
YDR227W Nucleus
YDR228C Nucleus
YDR235W Nucleus
YDR240C Nucleus
YDR243C Nucleus
YDR252W Nucleus
YDR253C Nucleus

YDR254W Nucleus
YDR257C Nucleus
YDR259C Nucleus
YDR260C Nucleus
YDR261C-C Nucleus
YDR261C-D Nucleus
YDR261W-A Nucleus
YDR261W-B Nucleus
YDR263C Nucleus
YDR267C Nucleus
YDR279W Nucleus
YDR280W Nucleus
YDR285W Nucleus
YDR288W Nucleus
YDR289C Nucleus
YDR291W Nucleus
YDR293C Nucleus
YDR295C Nucleus
YDR296W Nucleus
YDR299W Nucleus
YDR301W Nucleus
YDR303C Nucleus
YDR305C Nucleus
YDR308C Nucleus
YDR310C Nucleus
YDR311W Nucleus
YDR312W Nucleus
YDR314C Nucleus
YDR315C Nucleus
YDR316W-A Nucleus
YDR316W-B Nucleus
YDR318W Nucleus
YDR320C-A Nucleus
YDR323C Nucleus
YDR324C Nucleus
YDR325W Nucleus
YDR328C Nucleus
YDR330W Nucleus
YDR334W Nucleus
YDR335W Nucleus
YDR339C Nucleus
YDR346C Nucleus
YDR354W Nucleus
YDR356W Nucleus
YDR359C Nucleus

YDR361C Nucleus
YDR362C Nucleus
YDR363W Nucleus
YDR363W-A Nucleus
YDR364C Nucleus
YDR365C Nucleus
YDR365W-A Nucleus
YDR365W-B Nucleus
YDR368W Nucleus
YDR369C Nucleus
YDR372C Nucleus
YDR378C Nucleus
YDR381W Nucleus
YDR383C Nucleus
YDR386W Nucleus
YDR390C Nucleus
YDR391C Nucleus
YDR392W Nucleus
YDR394W Nucleus
YDR395W Nucleus
YDR397C Nucleus
YDR398W Nucleus
YDR399W Nucleus
YDR400W Nucleus
YDR404C Nucleus
YDR408C Nucleus
YDR409W Nucleus
YDR410C Nucleus
YDR412W Nucleus
YDR416W Nucleus
YDR419W Nucleus
YDR421W Nucleus
YDR423C Nucleus
YDR427W Nucleus
YDR432W Nucleus
YDR437W Nucleus
YDR439W Nucleus
YDR440W Nucleus
YDR443C Nucleus
YDR446W Nucleus
YDR448W Nucleus
YDR449C Nucleus
YDR451C Nucleus
YDR452W Nucleus
YDR454C Nucleus

YDR457W Nucleus
YDR458C Nucleus
YDR460W Nucleus
YDR463W Nucleus
YDR464W Nucleus
YDR465C Nucleus
YDR469W Nucleus
YDR473C Nucleus
YDR477W Nucleus
YDR478W Nucleus
YDR480W Nucleus
YDR482C Nucleus
YDR485C Nucleus
YDR489W Nucleus
YDR496C Nucleus
YDR499W Nucleus
YDR501W Nucleus
YDR510W Nucleus
YDR514C Nucleus
YDR519W Nucleus
YDR520C Nucleus
YDR523C Nucleus
YDR530C Nucleus
YDR531W Nucleus
YDR532C Nucleus
YDR540C Nucleus
YDR545W Nucleus
YEL002C Nucleus
YEL007W Nucleus
YEL009C Nucleus
YEL013W Nucleus
YEL015W Nucleus
YEL017W Nucleus
YEL018W Nucleus
YEL019C Nucleus
YEL025C Nucleus
YEL026W Nucleus
YEL029C Nucleus
YEL032W Nucleus
YEL037C Nucleus
YEL038W Nucleus
YEL044W Nucleus
YEL055C Nucleus
YEL056W Nucleus
YEL058W Nucleus

YEL061C Nucleus
YEL066W Nucleus
YER002W Nucleus
YER003C Nucleus
YER006W Nucleus
YER009W Nucleus
YER012W Nucleus
YER013W Nucleus
YER018C Nucleus
YER022W Nucleus
YER027C Nucleus
YER028C Nucleus
YER029C Nucleus
YER030W Nucleus
YER034W Nucleus
YER035W Nucleus
YER036C Nucleus
YER037W Nucleus
YER038C Nucleus
YER040W Nucleus
YER041W Nucleus
YER042W Nucleus
YER044C-A Nucleus
YER045C Nucleus
YER048C Nucleus
YER049W Nucleus
YER051W Nucleus
YER057C Nucleus
YER059W Nucleus
YER062C Nucleus
YER063W Nucleus
YER064C Nucleus
YER067W Nucleus
YER068W Nucleus
YER070W Nucleus
YER074W Nucleus
YER075C Nucleus
YER078C Nucleus
YER079W Nucleus
YER082C Nucleus
YER084W Nucleus
YER088C Nucleus
YER089C Nucleus
YER092W Nucleus
YER094C Nucleus

YER095W Nucleus
YER103W Nucleus
YER104W Nucleus
YER105C Nucleus
YER106W Nucleus
YER107C Nucleus
YER109C Nucleus
YER110C Nucleus
YER111C Nucleus
YER112W Nucleus
YER116C Nucleus
YER117W Nucleus
YER120W Nucleus
YER123W Nucleus
YER125W Nucleus
YER126C Nucleus
YER127W Nucleus
YER130C Nucleus
YER133W Nucleus
YER134C Nucleus
YER137C-A Nucleus
YER138C Nucleus
YER139C Nucleus
YER142C Nucleus
YER146W Nucleus
YER147C Nucleus
YER148W Nucleus
YER152C Nucleus
YER156C Nucleus
YER159C Nucleus
YER159C-A Nucleus
YER160C Nucleus
YER161C Nucleus
YER162C Nucleus
YER163C Nucleus
YER164W Nucleus
YER165W Nucleus
YER167W Nucleus
YER168C Nucleus
YER169W Nucleus
YER171W Nucleus
YER172C Nucleus
YER173W Nucleus
YER174C Nucleus
YER177W Nucleus

YER179W Nucleus
YER184C Nucleus
YER190W Nucleus
YFL001W Nucleus
YFL002C Nucleus
YFL002W-A Nucleus
YFL002W-B Nucleus
YFL003C Nucleus
YFL007W Nucleus
YFL008W Nucleus
YFL009W Nucleus
YFL010C Nucleus
YFL013C Nucleus
YFL014W Nucleus
YFL017C Nucleus
YFL017W-A Nucleus
YFL021W Nucleus
YFL024C Nucleus
YFL028C Nucleus
YFL031W Nucleus
YFL033C Nucleus
YFL034C-B Nucleus
YFL037W Nucleus
YFL039C Nucleus
YFL044C Nucleus
YFL049W Nucleus
YFL052W Nucleus
YFL062W Nucleus
YFR001W Nucleus
YFR002W Nucleus
YFR003C Nucleus
YFR004W Nucleus
YFR005C Nucleus
YFR013W Nucleus
YFR023W Nucleus
YFR027W Nucleus
YFR028C Nucleus
YFR031C Nucleus
YFR034C Nucleus
YFR036W Nucleus
YFR037C Nucleus
YFR038W Nucleus
YFR046C Nucleus
YFR047C Nucleus
YFR050C Nucleus

YGL003C Nucleus
YGL004C Nucleus
YGL011C Nucleus
YGL013C Nucleus
YGL016W Nucleus
YGL019W Nucleus
YGL025C Nucleus
YGL026C Nucleus
YGL029W Nucleus
YGL033W Nucleus
YGL035C Nucleus
YGL037C Nucleus
YGL040C Nucleus
YGL043W Nucleus
YGL044C Nucleus
YGL047W Nucleus
YGL048C Nucleus
YGL056C Nucleus
YGL058W Nucleus
YGL060W Nucleus
YGL061C Nucleus
YGL066W Nucleus
YGL070C Nucleus
YGL071W Nucleus
YGL073W Nucleus
YGL075C Nucleus
YGL078C Nucleus
YGL082W Nucleus
YGL086W Nucleus
YGL087C Nucleus
YGL090W Nucleus
YGL091C Nucleus
YGL092W Nucleus
YGL093W Nucleus
YGL096W Nucleus
YGL097W Nucleus
YGL100W Nucleus
YGL101W Nucleus
YGL103W Nucleus
YGL111W Nucleus
YGL112C Nucleus
YGL113W Nucleus
YGL115W Nucleus
YGL116W Nucleus
YGL120C Nucleus

YGL122C Nucleus
YGL123W Nucleus
YGL127C Nucleus
YGL128C Nucleus
YGL130W Nucleus
YGL131C Nucleus
YGL133W Nucleus
YGL141W Nucleus
YGL150C Nucleus
YGL151W Nucleus
YGL157W Nucleus
YGL162W Nucleus
YGL163C Nucleus
YGL164C Nucleus
YGL166W Nucleus
YGL169W Nucleus
YGL171W Nucleus
YGL172W Nucleus
YGL173C Nucleus
YGL174W Nucleus
YGL175C Nucleus
YGL181W Nucleus
YGL183C Nucleus
YGL184C Nucleus
YGL190C Nucleus
YGL192W Nucleus
YGL194C Nucleus
YGL201C Nucleus
YGL207W Nucleus
YGL209W Nucleus
YGL213C Nucleus
YGL216W Nucleus
YGL220W Nucleus
YGL222C Nucleus
YGL227W Nucleus
YGL232W Nucleus
YGL237C Nucleus
YGL238W Nucleus
YGL240W Nucleus
YGL241W Nucleus
YGL243W Nucleus
YGL244W Nucleus
YGL246C Nucleus
YGL247W Nucleus
YGL249W Nucleus

YGL250W Nucleus
YGL251C Nucleus
YGL252C Nucleus
YGL253W Nucleus
YGL254W Nucleus
YGR002C Nucleus
YGR003W Nucleus
YGR005C Nucleus
YGR006W Nucleus
YGR007W Nucleus
YGR010W Nucleus
YGR013W Nucleus
YGR017W Nucleus
YGR024C Nucleus
YGR027W-A Nucleus
YGR027W-B Nucleus
YGR030C Nucleus
YGR037C Nucleus
YGR038C-A Nucleus
YGR038C-B Nucleus
YGR040W Nucleus
YGR042W Nucleus
YGR043C Nucleus
YGR044C Nucleus
YGR047C Nucleus
YGR048W Nucleus
YGR056W Nucleus
YGR058W Nucleus
YGR063C Nucleus
YGR067C Nucleus
YGR071C Nucleus
YGR072W Nucleus
YGR074W Nucleus
YGR075C Nucleus
YGR081C Nucleus
YGR085C Nucleus
YGR090W Nucleus
YGR091W Nucleus
YGR092W Nucleus
YGR093W Nucleus
YGR095C Nucleus
YGR097W Nucleus
YGR098C Nucleus
YGR099W Nucleus
YGR103W Nucleus

YGR104C Nucleus
YGR108W Nucleus
YGR109C Nucleus
YGR109W-A Nucleus
YGR109W-B Nucleus
YGR111W Nucleus
YGR113W Nucleus
YGR116W Nucleus
YGR119C Nucleus
YGR122W Nucleus
YGR123C Nucleus
YGR126W Nucleus
YGR128C Nucleus
YGR129W Nucleus
YGR134W Nucleus
YGR135W Nucleus
YGR136W Nucleus
YGR140W Nucleus
YGR142W Nucleus
YGR144W Nucleus
YGR145W Nucleus
YGR156W Nucleus
YGR158C Nucleus
YGR159C Nucleus
YGR161C Nucleus
YGR161C-C Nucleus
YGR161C-D Nucleus
YGR161W-A Nucleus
YGR161W-B Nucleus
YGR163W Nucleus
YGR178C Nucleus
YGR179C Nucleus
YGR180C Nucleus
YGR185C Nucleus
YGR186W Nucleus
YGR187C Nucleus
YGR188C Nucleus
YGR195W Nucleus
YGR200C Nucleus
YGR202C Nucleus
YGR203W Nucleus
YGR204W Nucleus
YGR205W Nucleus
YGR208W Nucleus
YGR209C Nucleus

YGR211W Nucleus
YGR218W Nucleus
YGR225W Nucleus
YGR229C Nucleus
YGR232W Nucleus
YGR233C Nucleus
YGR234W Nucleus
YGR245C Nucleus
YGR246C Nucleus
YGR248W Nucleus
YGR249W Nucleus
YGR251W Nucleus
YGR252W Nucleus
YGR253C Nucleus
YGR258C Nucleus
YGR262C Nucleus
YGR267C Nucleus
YGR270W Nucleus
YGR271C-A Nucleus
YGR274C Nucleus
YGR275W Nucleus
YGR276C Nucleus
YGR277C Nucleus
YGR278W Nucleus
YGR280C Nucleus
YGR283C Nucleus
YGR285C Nucleus
YGR288W Nucleus
YGR296W Nucleus
YHL006C Nucleus
YHL007C Nucleus
YHL009C Nucleus
YHL009W-A Nucleus
YHL009W-B Nucleus
YHL020C Nucleus
YHL022C Nucleus
YHL025W Nucleus
YHL027W Nucleus
YHL030W Nucleus
YHL034C Nucleus
YHL040C Nucleus
YHL048W Nucleus
YHR004C Nucleus
YHR006W Nucleus
YHR014W Nucleus

YHR027C Nucleus
YHR030C Nucleus
YHR031C Nucleus
YHR034C Nucleus
YHR036W Nucleus
YHR040W Nucleus
YHR041C Nucleus
YHR043C Nucleus
YHR046C Nucleus
YHR047C Nucleus
YHR049W Nucleus
YHR052W Nucleus
YHR056C Nucleus
YHR058C Nucleus
YHR061C Nucleus
YHR062C Nucleus
YHR065C Nucleus
YHR066W Nucleus
YHR069C Nucleus
YHR070W Nucleus
YHR071W Nucleus
YHR072W-A Nucleus
YHR074W Nucleus
YHR076W Nucleus
YHR079C Nucleus
YHR079C-A Nucleus
YHR081W Nucleus
YHR082C Nucleus
YHR084W Nucleus
YHR085W Nucleus
YHR086W Nucleus
YHR087W Nucleus
YHR088W Nucleus
YHR089C Nucleus
YHR090C Nucleus
YHR097C Nucleus
YHR099W Nucleus
YHR104W Nucleus
YHR112C Nucleus
YHR118C Nucleus
YHR119W Nucleus
YHR121W Nucleus
YHR122W Nucleus
YHR124W Nucleus
YHR127W Nucleus

YHR132W-A Nucleus
YHR133C Nucleus
YHR134W Nucleus
YHR137W Nucleus
YHR143W-A Nucleus
YHR146W Nucleus
YHR148W Nucleus
YHR152W Nucleus
YHR154W Nucleus
YHR156C Nucleus
YHR157W Nucleus
YHR163W Nucleus
YHR164C Nucleus
YHR165C Nucleus
YHR166C Nucleus
YHR167W Nucleus
YHR169W Nucleus
YHR170W Nucleus
YHR172W Nucleus
YHR177W Nucleus
YHR178W Nucleus
YHR179W Nucleus
YHR185C Nucleus
YHR187W Nucleus
YHR191C Nucleus
YHR193C Nucleus
YHR194W Nucleus
YHR195W Nucleus
YHR196W Nucleus
YHR197W Nucleus
YHR199C-A Nucleus
YHR205W Nucleus
YHR206W Nucleus
YHR207C Nucleus
YHR214C-B Nucleus
YHR214C-C Nucleus
YIL007C Nucleus
YIL008W Nucleus
YIL009C-A Nucleus
YIL010W Nucleus
YIL016W Nucleus
YIL017C Nucleus
YIL019W Nucleus
YIL020C Nucleus
YIL021W Nucleus

YIL026C Nucleus
YIL030C Nucleus
YIL031W Nucleus
YIL033C Nucleus
YIL035C Nucleus
YIL036W Nucleus
YIL038C Nucleus
YIL040W Nucleus
YIL046W Nucleus
YIL050W Nucleus
YIL053W Nucleus
YIL056W Nucleus
YIL061C Nucleus
YIL063C Nucleus
YIL069C Nucleus
YIL071C Nucleus
YIL072W Nucleus
YIL073C Nucleus
YIL075C Nucleus
YIL079C Nucleus
YIL082W-A Nucleus
YIL083C Nucleus
YIL084C Nucleus
YIL091C Nucleus
YIL092W Nucleus
YIL096C Nucleus
YIL097W Nucleus
YIL101C Nucleus
YIL104C Nucleus
YIL106W Nucleus
YIL110W Nucleus
YIL112W Nucleus
YIL113W Nucleus
YIL115C Nucleus
YIL119C Nucleus
YIL122W Nucleus
YIL126W Nucleus
YIL127C Nucleus
YIL128W Nucleus
YIL130W Nucleus
YIL131C Nucleus
YIL132C Nucleus
YIL143C Nucleus
YIL144W Nucleus
YIL145C Nucleus

YIL148W Nucleus
YIL149C Nucleus
YIL150C Nucleus
YIL153W Nucleus
YIL158W Nucleus
YIR002C Nucleus
YIR005W Nucleus
YIR006C Nucleus
YIR008C Nucleus
YIR009W Nucleus
YIR010W Nucleus
YIR011C Nucleus
YIR013C Nucleus
YIR015W Nucleus
YIR017C Nucleus
YIR018W Nucleus
YIR023W Nucleus
YIR025W Nucleus
YIR026C Nucleus
YIR033W Nucleus
YJL001W Nucleus
YJL006C Nucleus
YJL010C Nucleus
YJL011C Nucleus
YJL013C Nucleus
YJL019W Nucleus
YJL025W Nucleus
YJL026W Nucleus
YJL030W Nucleus
YJL033W Nucleus
YJL035C Nucleus
YJL039C Nucleus
YJL041W Nucleus
YJL047C Nucleus
YJL048C Nucleus
YJL050W Nucleus
YJL055W Nucleus
YJL056C Nucleus
YJL061W Nucleus
YJL065C Nucleus
YJL069C Nucleus
YJL072C Nucleus
YJL073W Nucleus
YJL074C Nucleus
YJL076W Nucleus

YJL079C Nucleus
YJL080C Nucleus
YJL081C Nucleus
YJL082W Nucleus
YJL087C Nucleus
YJL089W Nucleus
YJL090C Nucleus
YJL092W Nucleus
YJL103C Nucleus
YJL106W Nucleus
YJL109C Nucleus
YJL110C Nucleus
YJL113W Nucleus
YJL114W Nucleus
YJL115W Nucleus
YJL122W Nucleus
YJL124C Nucleus
YJL125C Nucleus
YJL127C Nucleus
YJL140W Nucleus
YJL141C Nucleus
YJL145W Nucleus
YJL146W Nucleus
YJL148W Nucleus
YJL157C Nucleus
YJL162C Nucleus
YJL164C Nucleus
YJL168C Nucleus
YJL173C Nucleus
YJL176C Nucleus
YJL184W Nucleus
YJL187C Nucleus
YJL191W Nucleus
YJL194W Nucleus
YJL197W Nucleus
YJL203W Nucleus
YJL206C Nucleus
YJL208C Nucleus
YJR002W Nucleus
YJR006W Nucleus
YJR008W Nucleus
YJR017C Nucleus
YJR021C Nucleus
YJR022W Nucleus
YJR025C Nucleus

YJR026W Nucleus
YJR027W Nucleus
YJR028W Nucleus
YJR029W Nucleus
YJR035W Nucleus
YJR036C Nucleus
YJR041C Nucleus
YJR042W Nucleus
YJR043C Nucleus
YJR045C Nucleus
YJR046W Nucleus
YJR049C Nucleus
YJR050W Nucleus
YJR052W Nucleus
YJR055W Nucleus
YJR056C Nucleus
YJR057W Nucleus
YJR059W Nucleus
YJR060W Nucleus
YJR063W Nucleus
YJR066W Nucleus
YJR067C Nucleus
YJR068W Nucleus
YJR069C Nucleus
YJR070C Nucleus
YJR074W Nucleus
YJR082C Nucleus
YJR084W Nucleus
YJR089W Nucleus
YJR090C Nucleus
YJR093C Nucleus
YJR094C Nucleus
YJR096W Nucleus
YJR097W Nucleus
YJR104C Nucleus
YJR105W Nucleus
YJR112W Nucleus
YJR117W Nucleus
YJR119C Nucleus
YJR127C Nucleus
YJR130C Nucleus
YJR132W Nucleus
YJR135C Nucleus
YJR136C Nucleus
YJR139C Nucleus

YJR140C Nucleus
YJR146W Nucleus
YJR147W Nucleus
YJR148W Nucleus
YKL005C Nucleus
YKL009W Nucleus
YKL010C Nucleus
YKL012W Nucleus
YKL014C Nucleus
YKL015W Nucleus
YKL017C Nucleus
YKL018W Nucleus
YKL020C Nucleus
YKL021C Nucleus
YKL022C Nucleus
YKL024C Nucleus
YKL028W Nucleus
YKL032C Nucleus
YKL033W Nucleus
YKL038W Nucleus
YKL042W Nucleus
YKL043W Nucleus
YKL045W Nucleus
YKL049C Nucleus
YKL052C Nucleus
YKL053C-A Nucleus
YKL054C Nucleus
YKL057C Nucleus
YKL058W Nucleus
YKL059C Nucleus
YKL062W Nucleus
YKL068W Nucleus
YKL069W Nucleus
YKL074C Nucleus
YKL078W Nucleus
YKL082C Nucleus
YKL086W Nucleus
YKL089W Nucleus
YKL091C Nucleus
YKL095W Nucleus
YKL099C Nucleus
YKL108W Nucleus
YKL109W Nucleus
YKL110C Nucleus
YKL112W Nucleus

YKL113C Nucleus
YKL114C Nucleus
YKL117W Nucleus
YKL122C Nucleus
YKL125W Nucleus
YKL128C Nucleus
YKL130C Nucleus
YKL138C-A Nucleus
YKL139W Nucleus
YKL143W Nucleus
YKL144C Nucleus
YKL145W Nucleus
YKL149C Nucleus
YKL160W Nucleus
YKL166C Nucleus
YKL172W Nucleus
YKL173W Nucleus
YKL183W Nucleus
YKL185W Nucleus
YKL186C Nucleus
YKL193C Nucleus
YKL205W Nucleus
YKL210W Nucleus
YKL213C Nucleus
YKL214C Nucleus
YKL222C Nucleus
YKR002W Nucleus
YKR008W Nucleus
YKR010C Nucleus
YKR011C Nucleus
YKR018C Nucleus
YKR022C Nucleus
YKR023W Nucleus
YKR024C Nucleus
YKR025W Nucleus
YKR029C Nucleus
YKR031C Nucleus
YKR034W Nucleus
YKR037C Nucleus
YKR038C Nucleus
YKR041W Nucleus
YKR043C Nucleus
YKR044W Nucleus
YKR048C Nucleus
YKR060W Nucleus

YKR062W Nucleus
YKR063C Nucleus
YKR064W Nucleus
YKR072C Nucleus
YKR075C Nucleus
YKR077W Nucleus
YKR079C Nucleus
YKR080W Nucleus
YKR081C Nucleus
YKR082W Nucleus
YKR083C Nucleus
YKR086W Nucleus
YKR092C Nucleus
YKR094C Nucleus
YKR095W Nucleus
YKR095W-A Nucleus
YKR096W Nucleus
YKR099W Nucleus
YKR101W Nucleus
YLL002W Nucleus
YLL004W Nucleus
YLL008W Nucleus
YLL011W Nucleus
YLL018C Nucleus
YLL019C Nucleus
YLL022C Nucleus
YLL023C Nucleus
YLL026W Nucleus
YLL034C Nucleus
YLL035W Nucleus
YLL036C Nucleus
YLL039C Nucleus
YLL050C Nucleus
YLL054C Nucleus
YLR002C Nucleus
YLR003C Nucleus
YLR005W Nucleus
YLR007W Nucleus
YLR009W Nucleus
YLR010C Nucleus
YLR011W Nucleus
YLR013W Nucleus
YLR014C Nucleus
YLR015W Nucleus
YLR016C Nucleus

YLR017W Nucleus
YLR018C Nucleus
YLR022C Nucleus
YLR032W Nucleus
YLR033W Nucleus
YLR035C Nucleus
YLR035C-A Nucleus
YLR039C Nucleus
YLR043C Nucleus
YLR044C Nucleus
YLR045C Nucleus
YLR051C Nucleus
YLR052W Nucleus
YLR055C Nucleus
YLR063W Nucleus
YLR064W Nucleus
YLR068W Nucleus
YLR071C Nucleus
YLR074C Nucleus
YLR079W Nucleus
YLR082C Nucleus
YLR085C Nucleus
YLR086W Nucleus
YLR090W Nucleus
YLR094C Nucleus
YLR095C Nucleus
YLR098C Nucleus
YLR102C Nucleus
YLR103C Nucleus
YLR105C Nucleus
YLR106C Nucleus
YLR107W Nucleus
YLR108C Nucleus
YLR113W Nucleus
YLR115W Nucleus
YLR116W Nucleus
YLR117C Nucleus
YLR118C Nucleus
YLR126C Nucleus
YLR127C Nucleus
YLR129W Nucleus
YLR131C Nucleus
YLR132C Nucleus
YLR134W Nucleus
YLR135W Nucleus

YLR136C Nucleus
YLR141W Nucleus
YLR145W Nucleus
YLR147C Nucleus
YLR150W Nucleus
YLR153C Nucleus
YLR154C Nucleus
YLR155C Nucleus
YLR157C-A Nucleus
YLR157C-B Nucleus
YLR160C Nucleus
YLR167W Nucleus
YLR175W Nucleus
YLR176C Nucleus
YLR179C Nucleus
YLR182W Nucleus
YLR183C Nucleus
YLR186W Nucleus
YLR196W Nucleus
YLR197W Nucleus
YLR200W Nucleus
YLR205C Nucleus
YLR208W Nucleus
YLR210W Nucleus
YLR218C Nucleus
YLR221C Nucleus
YLR222C Nucleus
YLR223C Nucleus
YLR226W Nucleus
YLR227W-A Nucleus
YLR227W-B Nucleus
YLR228C Nucleus
YLR229C Nucleus
YLR231C Nucleus
YLR233C Nucleus
YLR234W Nucleus
YLR240W Nucleus
YLR245C Nucleus
YLR247C Nucleus
YLR254C Nucleus
YLR256W Nucleus
YLR256W-A Nucleus
YLR258W Nucleus
YLR262C-A Nucleus
YLR263W Nucleus

YLR265C Nucleus
YLR266C Nucleus
YLR270W Nucleus
YLR271W Nucleus
YLR272C Nucleus
YLR274W Nucleus
YLR275W Nucleus
YLR276C Nucleus
YLR277C Nucleus
YLR278C Nucleus
YLR286C Nucleus
YLR288C Nucleus
YLR293C Nucleus
YLR297W Nucleus
YLR298C Nucleus
YLR301W Nucleus
YLR310C Nucleus
YLR315W Nucleus
YLR316C Nucleus
YLR318W Nucleus
YLR320W Nucleus
YLR321C Nucleus
YLR323C Nucleus
YLR327C Nucleus
YLR328W Nucleus
YLR329W Nucleus
YLR335W Nucleus
YLR336C Nucleus
YLR347C Nucleus
YLR354C Nucleus
YLR357W Nucleus
YLR363C Nucleus
YLR363W-A Nucleus
YLR373C Nucleus
YLR375W Nucleus
YLR376C Nucleus
YLR381W Nucleus
YLR383W Nucleus
YLR384C Nucleus
YLR385C Nucleus
YLR394W Nucleus
YLR399C Nucleus
YLR401C Nucleus
YLR403W Nucleus
YLR409C Nucleus

YLR410W-A Nucleus
YLR410W-B Nucleus
YLR418C Nucleus
YLR420W Nucleus
YLR421C Nucleus
YLR424W Nucleus
YLR430W Nucleus
YLR435W Nucleus
YLR437C Nucleus
YLR438C-A Nucleus
YLR438W Nucleus
YLR440C Nucleus
YLR442C Nucleus
YLR445W Nucleus
YLR449W Nucleus
YLR450W Nucleus
YLR451W Nucleus
YLR453C Nucleus
YLR455W Nucleus
YLR456W Nucleus
YLR457C Nucleus
YLR466W Nucleus
YLR467W Nucleus
YML004C Nucleus
YML007W Nucleus
YML010W Nucleus
YML011C Nucleus
YML014W Nucleus
YML015C Nucleus
YML016C Nucleus
YML021C Nucleus
YML022W Nucleus
YML023C Nucleus
YML027W Nucleus
YML031W Nucleus
YML032C Nucleus
YML034W Nucleus
YML036W Nucleus
YML039W Nucleus
YML040W Nucleus
YML041C Nucleus
YML043C Nucleus
YML045W Nucleus
YML045W-A Nucleus
YML046W Nucleus

YML049C Nucleus
YML051W Nucleus
YML053C Nucleus
YML058W Nucleus
YML058W-A Nucleus
YML060W Nucleus
YML061C Nucleus
YML062C Nucleus
YML065W Nucleus
YML069W Nucleus
YML074C Nucleus
YML075C Nucleus
YML076C Nucleus
YML079W Nucleus
YML080W Nucleus
YML081W Nucleus
YML082W Nucleus
YML085C Nucleus
YML088W Nucleus
YML091C Nucleus
YML092C Nucleus
YML093W Nucleus
YML095C Nucleus
YML098W Nucleus
YML099C Nucleus
YML102W Nucleus
YML103C Nucleus
YML106W Nucleus
YML107C Nucleus
YML108W Nucleus
YML109W Nucleus
YML112W Nucleus
YML113W Nucleus
YML114C Nucleus
YML121W Nucleus
YML124C Nucleus
YML126C Nucleus
YML127W Nucleus
YMR001C Nucleus
YMR002W Nucleus
YMR004W Nucleus
YMR005W Nucleus
YMR009W Nucleus
YMR014W Nucleus
YMR016C Nucleus

YMR019W Nucleus
YMR021C Nucleus
YMR025W Nucleus
YMR027W Nucleus
YMR030W Nucleus
YMR033W Nucleus
YMR036C Nucleus
YMR037C Nucleus
YMR038C Nucleus
YMR039C Nucleus
YMR042W Nucleus
YMR043W Nucleus
YMR044W Nucleus
YMR045C Nucleus
YMR046C Nucleus
YMR047C Nucleus
YMR048W Nucleus
YMR049C Nucleus
YMR050C Nucleus
YMR051C Nucleus
YMR053C Nucleus
YMR059W Nucleus
YMR061W Nucleus
YMR065W Nucleus
YMR067C Nucleus
YMR069W Nucleus
YMR070W Nucleus
YMR072W Nucleus
YMR074C Nucleus
YMR075W Nucleus
YMR076C Nucleus
YMR078C Nucleus
YMR080C Nucleus
YMR091C Nucleus
YMR092C Nucleus
YMR093W Nucleus
YMR094W Nucleus
YMR099C Nucleus
YMR104C Nucleus
YMR106C Nucleus
YMR111C Nucleus
YMR112C Nucleus
YMR114C Nucleus
YMR117C Nucleus
YMR119W Nucleus

YMR121C Nucleus
YMR125W Nucleus
YMR127C Nucleus
YMR128W Nucleus
YMR129W Nucleus
YMR131C Nucleus
YMR133W Nucleus
YMR134W Nucleus
YMR135C Nucleus
YMR136W Nucleus
YMR137C Nucleus
YMR144W Nucleus
YMR153W Nucleus
YMR156C Nucleus
YMR159C Nucleus
YMR164C Nucleus
YMR165C Nucleus
YMR167W Nucleus
YMR168C Nucleus
YMR172W Nucleus
YMR174C Nucleus
YMR176W Nucleus
YMR178W Nucleus
YMR179W Nucleus
YMR180C Nucleus
YMR182C Nucleus
YMR190C Nucleus
YMR198W Nucleus
YMR199W Nucleus
YMR201C Nucleus
YMR208W Nucleus
YMR213W Nucleus
YMR219W Nucleus
YMR220W Nucleus
YMR223W Nucleus
YMR224C Nucleus
YMR226C Nucleus
YMR227C Nucleus
YMR229C Nucleus
YMR232W Nucleus
YMR233W Nucleus
YMR234W Nucleus
YMR235C Nucleus
YMR236W Nucleus
YMR239C Nucleus

YMR240C Nucleus
YMR244C-A Nucleus
YMR247C Nucleus
YMR255W Nucleus
YMR258C Nucleus
YMR263W Nucleus
YMR268C Nucleus
YMR269W Nucleus
YMR270C Nucleus
YMR276W Nucleus
YMR277W Nucleus
YMR278W Nucleus
YMR280C Nucleus
YMR284W Nucleus
YMR285C Nucleus
YMR288W Nucleus
YMR290C Nucleus
YMR291W Nucleus
YMR298W Nucleus
YMR307W Nucleus
YMR308C Nucleus
YMR310C Nucleus
YMR311C Nucleus
YMR312W Nucleus
YMR314W Nucleus
YMR315W Nucleus
YNL002C Nucleus
YNL004W Nucleus
YNL007C Nucleus
YNL008C Nucleus
YNL010W Nucleus
YNL012W Nucleus
YNL015W Nucleus
YNL016W Nucleus
YNL021W Nucleus
YNL022C Nucleus
YNL023C Nucleus
YNL025C Nucleus
YNL027W Nucleus
YNL030W Nucleus
YNL031C Nucleus
YNL035C Nucleus
YNL036W Nucleus
YNL039W Nucleus
YNL042W Nucleus

YNL045W Nucleus
YNL053W Nucleus
YNL054W-A Nucleus
YNL059C Nucleus
YNL061W Nucleus
YNL062C Nucleus
YNL068C Nucleus
YNL072W Nucleus
YNL075W Nucleus
YNL076W Nucleus
YNL077W Nucleus
YNL078W Nucleus
YNL082W Nucleus
YNL088W Nucleus
YNL096C Nucleus
YNL097C Nucleus
YNL098C Nucleus
YNL102W Nucleus
YNL103W Nucleus
YNL107W Nucleus
YNL108C Nucleus
YNL110C Nucleus
YNL112W Nucleus
YNL113W Nucleus
YNL118C Nucleus
YNL123W Nucleus
YNL124W Nucleus
YNL126W Nucleus
YNL132W Nucleus
YNL133C Nucleus
YNL134C Nucleus
YNL135C Nucleus
YNL136W Nucleus
YNL138W-A Nucleus
YNL139C Nucleus
YNL141W Nucleus
YNL147W Nucleus
YNL148C Nucleus
YNL149C Nucleus
YNL151C Nucleus
YNL155W Nucleus
YNL157W Nucleus
YNL158W Nucleus
YNL159C Nucleus
YNL161W Nucleus

YNL162W-A Nucleus
YNL164C Nucleus
YNL167C Nucleus
YNL172W Nucleus
YNL173C Nucleus
YNL175C Nucleus
YNL180C Nucleus
YNL181W Nucleus
YNL182C Nucleus
YNL186W Nucleus
YNL187W Nucleus
YNL189W Nucleus
YNL196C Nucleus
YNL199C Nucleus
YNL201C Nucleus
YNL206C Nucleus
YNL207W Nucleus
YNL210W Nucleus
YNL215W Nucleus
YNL216W Nucleus
YNL218W Nucleus
YNL221C Nucleus
YNL222W Nucleus
YNL223W Nucleus
YNL224C Nucleus
YNL227C Nucleus
YNL230C Nucleus
YNL232W Nucleus
YNL236W Nucleus
YNL240C Nucleus
YNL245C Nucleus
YNL246W Nucleus
YNL248C Nucleus
YNL250W Nucleus
YNL251C Nucleus
YNL253W Nucleus
YNL254C Nucleus
YNL258C Nucleus
YNL260C Nucleus
YNL261W Nucleus
YNL262W Nucleus
YNL267W Nucleus
YNL273W Nucleus
YNL274C Nucleus
YNL278W Nucleus

YNL281W Nucleus
YNL282W Nucleus
YNL284C-A Nucleus
YNL284C-B Nucleus
YNL286W Nucleus
YNL288W Nucleus
YNL289W Nucleus
YNL290W Nucleus
YNL292W Nucleus
YNL298W Nucleus
YNL299W Nucleus
YNL308C Nucleus
YNL309W Nucleus
YNL312W Nucleus
YNL313C Nucleus
YNL314W Nucleus
YNL317W Nucleus
YNL330C Nucleus
YNL339C Nucleus
YNR003C Nucleus
YNR004W Nucleus
YNR009W Nucleus
YNR010W Nucleus
YNR011C Nucleus
YNR012W Nucleus
YNR015W Nucleus
YNR023W Nucleus
YNR024W Nucleus
YNR027W Nucleus
YNR032W Nucleus
YNR034W Nucleus
YNR038W Nucleus
YNR046W Nucleus
YNR049C Nucleus
YNR052C Nucleus
YNR053C Nucleus
YNR054C Nucleus
YNR063W Nucleus
YNR074C Nucleus
YNR075W Nucleus
YOL001W Nucleus
YOL004W Nucleus
YOL005C Nucleus
YOL006C Nucleus
YOL009C Nucleus

YOL010W Nucleus
YOL012C Nucleus
YOL017W Nucleus
YOL021C Nucleus
YOL028C Nucleus
YOL032W Nucleus
YOL034W Nucleus
YOL038W Nucleus
YOL041C Nucleus
YOL043C Nucleus
YOL051W Nucleus
YOL052C Nucleus
YOL054W Nucleus
YOL057W Nucleus
YOL064C Nucleus
YOL067C Nucleus
YOL068C Nucleus
YOL069W Nucleus
YOL072W Nucleus
YOL077C Nucleus
YOL080C Nucleus
YOL086W-A Nucleus
YOL089C Nucleus
YOL090W Nucleus
YOL093W Nucleus
YOL094C Nucleus
YOL100W Nucleus
YOL101C Nucleus
YOL102C Nucleus
YOL103W-A Nucleus
YOL103W-B Nucleus
YOL104C Nucleus
YOL108C Nucleus
YOL111C Nucleus
YOL113W Nucleus
YOL115W Nucleus
YOL116W Nucleus
YOL117W Nucleus
YOL123W Nucleus
YOL125W Nucleus
YOL133W Nucleus
YOL135C Nucleus
YOL139C Nucleus
YOL142W Nucleus
YOL143C Nucleus

YOL144W Nucleus
YOL145C Nucleus
YOL146W Nucleus
YOL148C Nucleus
YOL149W Nucleus
YOL151W Nucleus
YOL159C-A Nucleus
YOR001W Nucleus
YOR004W Nucleus
YOR005C Nucleus
YOR006C Nucleus
YOR014W Nucleus
YOR023C Nucleus
YOR025W Nucleus
YOR026W Nucleus
YOR028C Nucleus
YOR032C Nucleus
YOR033C Nucleus
YOR038C Nucleus
YOR039W Nucleus
YOR046C Nucleus
YOR047C Nucleus
YOR048C Nucleus
YOR051C Nucleus
YOR052C Nucleus
YOR056C Nucleus
YOR058C Nucleus
YOR060C Nucleus
YOR061W Nucleus
YOR062C Nucleus
YOR064C Nucleus
YOR066W Nucleus
YOR073W Nucleus
YOR074C Nucleus
YOR077W Nucleus
YOR078W Nucleus
YOR080W Nucleus
YOR083W Nucleus
YOR093C Nucleus
YOR095C Nucleus
YOR096W Nucleus
YOR098C Nucleus
YOR101W Nucleus
YOR107W Nucleus
YOR110W Nucleus

YOR113W Nucleus
YOR116C Nucleus
YOR117W Nucleus
YOR119C Nucleus
YOR120W Nucleus
YOR123C Nucleus
YOR129C Nucleus
YOR131C Nucleus
YOR138C Nucleus
YOR140W Nucleus
YOR141C Nucleus
YOR142W-A Nucleus
YOR142W-B Nucleus
YOR144C Nucleus
YOR145C Nucleus
YOR148C Nucleus
YOR151C Nucleus
YOR156C Nucleus
YOR157C Nucleus
YOR159C Nucleus
YOR160W Nucleus
YOR162C Nucleus
YOR163W Nucleus
YOR166C Nucleus
YOR172W Nucleus
YOR174W Nucleus
YOR179C Nucleus
YOR185C Nucleus
YOR189W Nucleus
YOR191W Nucleus
YOR192C-A Nucleus
YOR192C-B Nucleus
YOR194C Nucleus
YOR195W Nucleus
YOR197W Nucleus
YOR204W Nucleus
YOR206W Nucleus
YOR207C Nucleus
YOR208W Nucleus
YOR209C Nucleus
YOR210W Nucleus
YOR213C Nucleus
YOR217W Nucleus
YOR224C Nucleus
YOR229W Nucleus

YOR230W Nucleus
YOR243C Nucleus
YOR244W Nucleus
YOR249C Nucleus
YOR250C Nucleus
YOR252W Nucleus
YOR254C Nucleus
YOR257W Nucleus
YOR258W Nucleus
YOR259C Nucleus
YOR264W Nucleus
YOR269W Nucleus
YOR272W Nucleus
YOR274W Nucleus
YOR275C Nucleus
YOR279C Nucleus
YOR283W Nucleus
YOR287C Nucleus
YOR289W Nucleus
YOR290C Nucleus
YOR294W Nucleus
YOR295W Nucleus
YOR298C-A Nucleus
YOR304W Nucleus
YOR308C Nucleus
YOR310C Nucleus
YOR311C Nucleus
YOR315W Nucleus
YOR319W Nucleus
YOR323C Nucleus
YOR329C Nucleus
YOR337W Nucleus
YOR340C Nucleus
YOR341W Nucleus
YOR342C Nucleus
YOR343W-A Nucleus
YOR343W-B Nucleus
YOR344C Nucleus
YOR346W Nucleus
YOR351C Nucleus
YOR352W Nucleus
YOR355W Nucleus
YOR358W Nucleus
YOR359W Nucleus
YOR360C Nucleus

YOR362C Nucleus
YOR363C Nucleus
YOR368W Nucleus
YOR370C Nucleus
YOR372C Nucleus
YOR373W Nucleus
YOR375C Nucleus
YOR380W Nucleus
YOR386W Nucleus
YOR396W Nucleus
YPL001W Nucleus
YPL007C Nucleus
YPL008W Nucleus
YPL011C Nucleus
YPL012W Nucleus
YPL014W Nucleus
YPL015C Nucleus
YPL016W Nucleus
YPL018W Nucleus
YPL020C Nucleus
YPL022W Nucleus
YPL024W Nucleus
YPL026C Nucleus
YPL028W Nucleus
YPL031C Nucleus
YPL037C Nucleus
YPL038W Nucleus
YPL042C Nucleus
YPL043W Nucleus
YPL046C Nucleus
YPL047W Nucleus
YPL048W Nucleus
YPL049C Nucleus
YPL055C Nucleus
YPL064C Nucleus
YPL068C Nucleus
YPL071C Nucleus
YPL075W Nucleus
YPL081W Nucleus
YPL082C Nucleus
YPL083C Nucleus
YPL086C Nucleus
YPL089C Nucleus
YPL090C Nucleus
YPL091W Nucleus

YPL093W Nucleus
YPL096W Nucleus
YPL101W Nucleus
YPL111W Nucleus
YPL116W Nucleus
YPL117C Nucleus
YPL121C Nucleus
YPL122C Nucleus
YPL124W Nucleus
YPL125W Nucleus
YPL126W Nucleus
YPL127C Nucleus
YPL128C Nucleus
YPL129W Nucleus
YPL131W Nucleus
YPL133C Nucleus
YPL138C Nucleus
YPL139C Nucleus
YPL146C Nucleus
YPL151C Nucleus
YPL153C Nucleus
YPL157W Nucleus
YPL161C Nucleus
YPL164C Nucleus
YPL166W Nucleus
YPL167C Nucleus
YPL169C Nucleus
YPL177C Nucleus
YPL178W Nucleus
YPL181W Nucleus
YPL184C Nucleus
YPL186C Nucleus
YPL190C Nucleus
YPL192C Nucleus
YPL193W Nucleus
YPL194W Nucleus
YPL198W Nucleus
YPL200W Nucleus
YPL201C Nucleus
YPL202C Nucleus
YPL203W Nucleus
YPL204W Nucleus
YPL208W Nucleus
YPL209C Nucleus
YPL210C Nucleus

YPL211W Nucleus
YPL212C Nucleus
YPL213W Nucleus
YPL216W Nucleus
YPL217C Nucleus
YPL219W Nucleus
YPL226W Nucleus
YPL228W Nucleus
YPL230W Nucleus
YPL233W Nucleus
YPL235W Nucleus
YPL239W Nucleus
YPL243W Nucleus
YPL245W Nucleus
YPL247C Nucleus
YPL248C Nucleus
YPL253C Nucleus
YPL254W Nucleus
YPL256C Nucleus
YPL257W-A Nucleus
YPL257W-B Nucleus
YPL260W Nucleus
YPL266W Nucleus
YPL267W Nucleus
YPL268W Nucleus
YPL269W Nucleus
YPL273W Nucleus
YPL283C Nucleus
YPR007C Nucleus
YPR008W Nucleus
YPR009W Nucleus
YPR010C Nucleus
YPR016C Nucleus
YPR018W Nucleus
YPR019W Nucleus
YPR022C Nucleus
YPR023C Nucleus
YPR025C Nucleus
YPR030W Nucleus
YPR031W Nucleus
YPR034W Nucleus
YPR035W Nucleus
YPR040W Nucleus
YPR045C Nucleus
YPR046W Nucleus

YPR051W Nucleus
YPR052C Nucleus
YPR056W Nucleus
YPR057W Nucleus
YPR060C Nucleus
YPR062W Nucleus
YPR065W Nucleus
YPR068C Nucleus
YPR069C Nucleus
YPR070W Nucleus
YPR072W Nucleus
YPR073C Nucleus
YPR082C Nucleus
YPR085C Nucleus
YPR086W Nucleus
YPR091C Nucleus
YPR093C Nucleus
YPR094W Nucleus
YPR101W Nucleus
YPR102C Nucleus
YPR103W Nucleus
YPR104C Nucleus
YPR107C Nucleus
YPR108W Nucleus
YPR110C Nucleus
YPR112C Nucleus
YPR118W Nucleus
YPR119W Nucleus
YPR120C Nucleus
YPR124W Nucleus
YPR127W Nucleus
YPR133C Nucleus
YPR135W Nucleus
YPR137C-A Nucleus
YPR137C-B Nucleus
YPR137W Nucleus
YPR141C Nucleus
YPR143W Nucleus
YPR144C Nucleus
YPR152C Nucleus
YPR154W Nucleus
YPR158C-C Nucleus
YPR158C-D Nucleus
YPR158W Nucleus
YPR158W-A Nucleus

YPR158W-B Nucleus
YPR161C Nucleus
YPR162C Nucleus
YPR164W Nucleus
YPR168W Nucleus
YPR169W Nucleus
YPR172W Nucleus
YPR174C Nucleus
YPR175W Nucleus
YPR178W Nucleus
YPR179C Nucleus
YPR180W Nucleus
YPR182W Nucleus
YPR184W Nucleus
YPR186C Nucleus
YPR187W Nucleus
YPR189W Nucleus
YPR190C Nucleus
YPR196W Nucleus
YPR199C Nucleus
YAL055W Peroxisome
YBR041W Peroxisome
YBR168W Peroxisome
YBR222C Peroxisome
YCL056C Peroxisome
YCR005C Peroxisome
YDL022W Peroxisome
YDL065C Peroxisome
YDL078C Peroxisome
YDR142C Peroxisome
YDR244W Peroxisome
YDR256C Peroxisome
YDR265W Peroxisome
YDR329C Peroxisome
YDR479C Peroxisome
YER015W Peroxisome
YGL037C Peroxisome
YGL067W Peroxisome
YGL153W Peroxisome
YGL184C Peroxisome
YGL205W Peroxisome
YGR004W Peroxisome
YGR028W Peroxisome
YGR077C Peroxisome
YGR133W Peroxisome

YGR154C Peroxisome
YGR239C Peroxisome
YHR150W Peroxisome
YHR160C Peroxisome
YIL065C Peroxisome
YIL160C Peroxisome
YIR031C Peroxisome
YIR037W Peroxisome
YJL185C Peroxisome
YJL210W Peroxisome
YJR019C Peroxisome
YKL026C Peroxisome
YKL188C Peroxisome
YKL197C Peroxisome
YKR001C Peroxisome
YKR009C Peroxisome
YLL001W Peroxisome
YLR027C Peroxisome
YLR151C Peroxisome
YLR191W Peroxisome
YLR240W Peroxisome
YLR284C Peroxisome
YLR324W Peroxisome
YML042W Peroxisome
YMR018W Peroxisome
YMR026C Peroxisome
YMR163C Peroxisome
YMR204C Peroxisome
YMR304W Peroxisome
YNL009W Peroxisome
YNL117W Peroxisome
YNL202W Peroxisome
YNL214W Peroxisome
YNL329C Peroxisome
YOL044W Peroxisome
YOL147C Peroxisome
YOR084W Peroxisome
YOR180C Peroxisome
YOR193W Peroxisome
YPL112C Peroxisome
YPL147W Peroxisome
YPR128C Peroxisome
YPR165W Peroxisome
YPR194C Peroxisome
YAL005C Extracellular

YAL063C Extracellular
YAR050W Extracellular
YAR071W Extracellular
YBL008W-A Extracellular
YBR067C Extracellular
YBR078W Extracellular
YBR093C Extracellular
YBR162C Extracellular
YBR200W-A Extracellular
YBR301W Extracellular
YCL058C Extracellular
YCR089W Extracellular
YDL049C Extracellular
YDR032C Extracellular
YDR055W Extracellular
YDR077W Extracellular
YDR371W Extracellular
YDR461W Extracellular
YDR534C Extracellular
YEL040W Extracellular
YER011W Extracellular
YER150W Extracellular
YGL028C Extracellular
YGL089C Extracellular
YGR037C Extracellular
YGR189C Extracellular
YGR279C Extracellular
YGR282C Extracellular
YHR057C Extracellular
YHR143W Extracellular
YHR211W Extracellular
YIL011W Extracellular
YIL015W Extracellular
YIL123W Extracellular
YIL162W Extracellular
YIR019C Extracellular
YJL078C Extracellular
YJL079C Extracellular
YJL158C Extracellular
YJL159W Extracellular
YJL160C Extracellular
YJL174W Extracellular
YJR004C Extracellular
YJR150C Extracellular
YJR151C Extracellular

YJR153W Extracellular
YKL096W Extracellular
YKL096W-A Extracellular
YKL157W Extracellular
YKL163W Extracellular
YKL164C Extracellular
YKR013W Extracellular
YKR042W Extracellular
YKR102W Extracellular
YLL024C Extracellular
YLR037C Extracellular
YLR040C Extracellular
YLR042C Extracellular
YLR110C Extracellular
YLR155C Extracellular
YLR157C Extracellular
YLR158C Extracellular
YLR160C Extracellular
YLR286C Extracellular
YLR300W Extracellular
YLR390W-A Extracellular
YMR006C Extracellular
YMR215W Extracellular
YMR305C Extracellular
YMR307W Extracellular
YNL066W Extracellular
YNL145W Extracellular
YNL160W Extracellular
YNL190W Extracellular
YNL300W Extracellular
YNL322C Extracellular
YNL327W Extracellular
YNR044W Extracellular
YNR067C Extracellular
YOL011W Extracellular
YOL030W Extracellular
YOL155C Extracellular
YOR009W Extracellular
YOR010C Extracellular
YOR190W Extracellular
YOR214C Extracellular
YOR247W Extracellular
YOR382W Extracellular
YOR383C Extracellular
YOR389W Extracellular

YPL123C Extracellular
YPL130W Extracellular
YPL187W Extracellular
YPR121W Extracellular
